# Alterations in sperm DNA methylation, non-coding RNA and histone retention associate with DDT-induced epigenetic transgenerational inheritance of disease

**DOI:** 10.1186/s13072-018-0178-0

**Published:** 2018-02-27

**Authors:** Michael K. Skinner, Millissia Ben Maamar, Ingrid Sadler-Riggleman, Daniel Beck, Eric Nilsson, Margaux McBirney, Rachel Klukovich, Yeming Xie, Chong Tang, Wei Yan

**Affiliations:** 1 Center for Reproductive Biology, School of Biological Sciences, Washington State University, Pullman, WA 99164-4236 USA; 2 Department of Physiology and Cell Biology, University of Nevada, Reno School of Medicine, Reno, NV 89557 USA

**Keywords:** Epigenetic, Transgenerational, Inheritance, Sperm, DNA methylation, ncRNA, Histone, DDT, Disease etiology

## Abstract

**Background:**

Environmental toxicants such as DDT have been shown to induce the epigenetic transgenerational inheritance of disease (e.g., obesity) through the germline. The current study was designed to investigate the DDT-induced concurrent alterations of a number of different epigenetic processes including DNA methylation, non-coding RNA (ncRNA) and histone retention in sperm.

**Methods:**

Gestating females were exposed transiently to DDT during fetal gonadal development, and then, the directly exposed F1 generation, the directly exposed germline F2 generation and the transgenerational F3 generation sperm were investigated.

**Results:**

DNA methylation and ncRNA were altered in each generation sperm with the direct exposure F1 and F2 generations being predominantly distinct from the F3 generation epimutations. The piRNA and small tRNA were the most predominant classes of ncRNA altered. A highly conserved set of histone retention sites were found in the control lineage generations which was not significantly altered between generations, but a large number of new histone retention sites were found only in the transgenerational generation DDT lineage sperm.

**Conclusions:**

Therefore, all three different epigenetic processes were concurrently altered as DDT induced the epigenetic transgenerational inheritance of sperm epimutations. The direct exposure generations sperm epigenetic alterations were distinct from the transgenerational sperm epimutations. The genomic features and gene associations with the epimutations were investigated to help elucidate the integration of these different epigenetic processes. Observations demonstrate all three epigenetic processes are involved in transgenerational inheritance. The different epigenetic processes appear to be integrated in mediating the epigenetic transgenerational inheritance phenomenon.

**Electronic supplementary material:**

The online version of this article (10.1186/s13072-018-0178-0) contains supplementary material, which is available to authorized users.

## Background

A variety of environmental factors have been shown to promote the germline-mediated epigenetic transgenerational inheritance of disease and phenotypic variation [1, 2]. This includes abnormal nutrition (caloric restriction or high-fat diets), stress [3–5] and toxicants [1, 6]. A large number of environmental toxicants such as the agricultural fungicide vinclozolin [2], herbicide atrazine [7], plastic-derived bisphenol A (BPA) [8, 9] and phthalates [8, 10], hydrocarbons jet fuel JP8 [11], tributyltin [12] and pesticides permethrin and DEET (*N*,*N*-diethyl-meta-toluamide) [13], methoxychlor [2, 14] and DDT (dichlorodiphenyltrichloroethane) [15] have all been shown to promote transgenerational phenomena. Environmentally induced epigenetic transgenerational inheritance has been observed in a number of species investigated including plants [16], flies [17], worms [18], fish [19], birds [20], rodents [2], pigs [21] and humans [22]. Therefore, the epigenetic transgenerational inheritance phenomenon appears highly conserved and influenced by critical environmental factors associated with each species [1]. The ability of environmental factors to promote the epigenetic transgenerational inheritance of disease will have an important role in disease etiology [1, 2, 22]. Environmental impacts on the epigenetic transgenerational inheritance of phenotypic variation for subsequent generations also appear to have an important role in evolutionary biology [23]. Therefore, elucidation of the molecular mechanisms involved in environmentally induced epigenetic transgenerational inheritance is important to fully understand disease etiology and evolution.

**Fig. 1:**
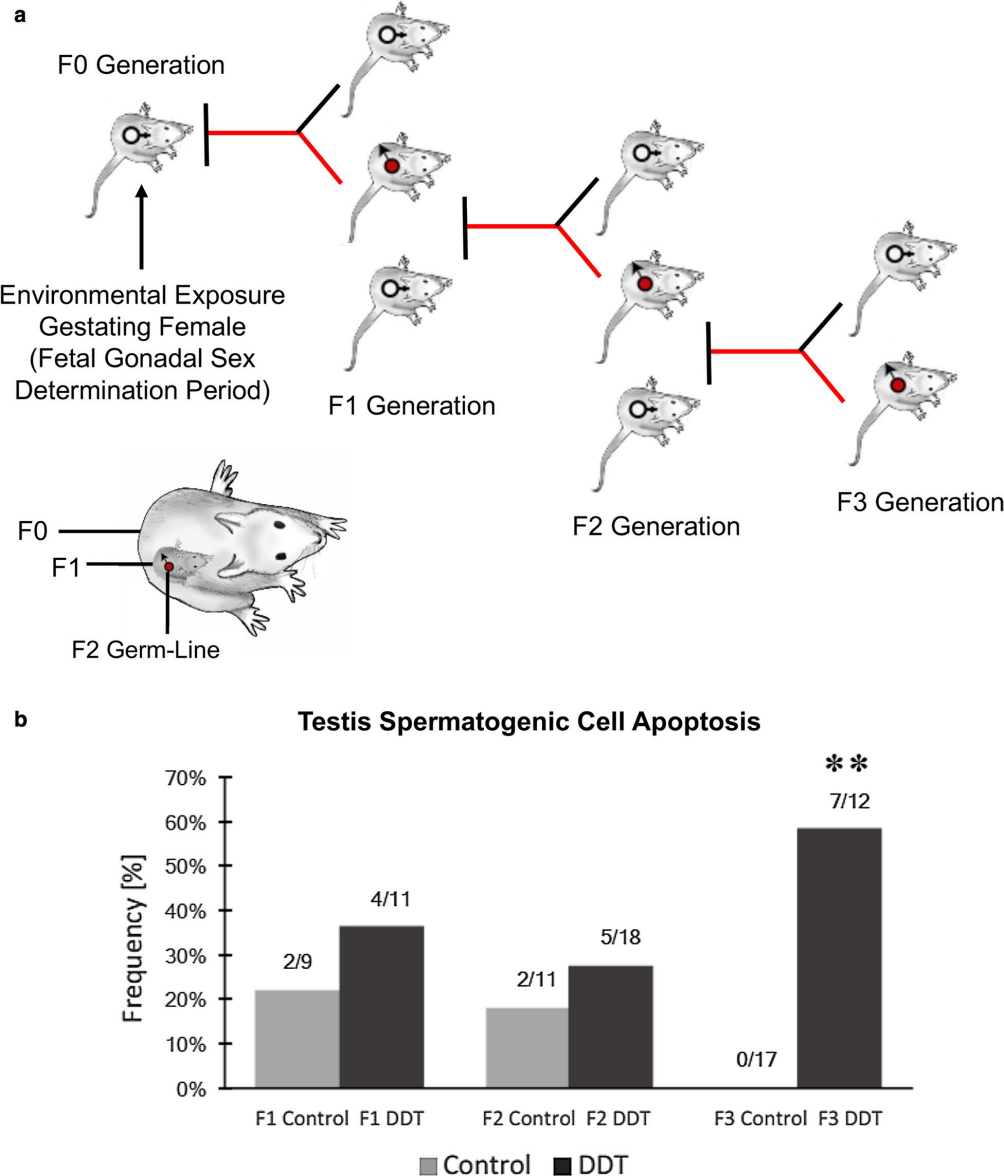
Animal breeding classification and disease. **a** Experimental design of F0 generation gestating female exposure and then F1, F2 and F3 generations being generated for sperm collection. The direct exposure of the F0 generation female, F1 generation fetus and F2 generation germline is also shown, **b** testis spermatogenic cell apoptosis as determined with TUNEL analysis with frequency (%) of apoptosis for each generation and lineage shown. Asterisk indicates statistical significance with control with a *p* < 0.01 with a Fisher’s exact *t* test

Epigenetic transgenerational inheritance is defined as the germline transmission of epigenetic information between generations in the absence of continued environmental exposures [24]. For example, exposure of a gestating female directly exposes the F0 generation female herself, the F1 generation fetus and its germline that will produce the F2 generation, such that the F3 generation (great grand-offspring) is the first transgenerational generation not being directly exposed [25] (Fig. 1a). In regard to a male or non-pregnant female, the male or female F0 generation and their respective germlines that will produce the F1 generation are directly exposed, so in this case the F2 generation is the first transgenerational generation [25]. Numerous studies in a number of different organisms exist for both the direct multigenerational exposure impacts and the subsequent transgenerational impacts [1]. In considering the molecular mechanisms involved in environmentally induced epigenetic transgenerational inheritance, it is critical to take into account the germline (sperm or egg) epigenetic alterations and the distinctions between the F1, F2 and F3 generations. No previous studies have reported the concurrent generational comparisons of the different epigenetic alterations in the germline.

The initial analysis of epigenetic transgenerational inheritance of alterations in the sperm involved the agricultural fungicide vinclozolin-induced transgenerational sperm DNA methylation changes [2]. Subsequently, the transgenerational sperm DNA methylation alterations from a wide variety of environmental toxicants were reported [2, 7–11, 13–15]. Interestingly, the transgenerational F3 generation sperm epigenetic alterations were predominantly exposure specific [26], identifying unique DNA methylation alterations termed epimutations. A variety of environmental impacts on transgenerational sperm DNA methylation have been observed including nutritional effects in mice and humans [27, 28] and mercury effects in fish [19]. A couple of studies have compared the F1 generation sperm and F3 generation sperm DNA methylation effects and have shown distinct changes [29, 30]. The initial analysis of epigenetic transgenerational inheritance of alterations in sperm non-coding RNA (ncRNA) involved stress-induced alterations in behavior and injection of the sperm ncRNA into eggs to generate the behavioral changes [31]. Subsequently, transgenerational ncRNA alterations in sperm have been reported involving vinclozolin [32] and stress [33]. Therefore, transgenerational sperm alterations in both DNA methylation and ncRNA have been observed, but minimal multiple generation studies have been conducted. Currently, no environmentally induced transgenerational alterations in histone modifications have been reported. Previous studies have established conserved gene-associated histone retention sites and modifications [34, 35] that suggest the potential of histone-modified transgenerational impacts [36]. A genetic mutation in a histone modification enzyme has been shown to alter generational impacts, also suggesting histones may have a role in epigenetic inheritance [37]. The current study was designed to investigate concurrent epigenetic modifications of DNA methylation, ncRNA and histones in sperm. Although the focus of this and previous studies has been on sperm due to the ability to isolate large numbers of sperm from most organisms, it is anticipated that epigenetic transgenerational inheritance will also involve epigenetic alterations in the egg. The inability to isolate sufficient numbers of eggs has restricted studies on egg, but new single cell technology will be helpful for this in the future.

The transgenerational model used for the current study involves DDT-induced epigenetic transgenerational inheritance of sperm epigenetic alterations and disease. DDT was the first major pesticide developed and used in the late 1940s and 1950s in agriculture and in populations to eliminate malaria in North America [38]. Although it was banned in the early 1970s in the USA and most of the world, it unfortunately has a long degradation half-life of over 25 years and is still today one of the contaminants found in 100% of pregnant women in the USA [39]. DDT is an estrogenic compound and can dramatically alter the endocrine system and development [40]. Previously we observed that DDT exposure of rats promotes the epigenetic transgenerational inheritance of disease [15]. One of the most predominant transgenerational diseases induced was obesity with approximately 50% of the population of males and females affected [15]. Interestingly, negligible obesity was observed in the F1 or F2 generation DDT lineage animals. Other transgenerational diseases included testis disease, ovary disease, kidney disease and prostate disease [15]. Nearly 90% of the F3 generation animals had at least one or more diseases. The DDT-induced epigenetic transgenerational inheritance of sperm DNA methylation changes in the F3 generation sperm was characterized [15]. The current study will use this DDT-induced epigenetic transgenerational inheritance model and a newly generated colony of rats to study the epigenetic changes in the F1, F2 and F3 generation sperm.

The objectives of the current study are to investigate the concurrent epigenetic alterations in sperm following DDT-induced epigenetic transgenerational inheritance of disease. The alterations in sperm DNA methylation, ncRNA and histones are investigated. The integration of this information and associated genes helps to elucidate the molecular processes involved in the epigenetic transgenerational inheritance phenomenon. Novel observations are provided on the role of modified histone retention sites, distinct DNA methylation and ncRNA alterations and the generational development of the germline-mediated transgenerational inheritance.

## Results

The experimental design (Fig. 1a) involved a daily transient exposure of gestating female F0 generation rats during postconception day 8–14 to 25 mg/kg body weight of DDT in DMSO using an intraperitoneal injection as previously described [1, 15]. A control generation lineage involved the exposure during day 8–14 of gestation to vehicle DMSO (dimethyl sulfoxide) alone. Six different gestating females from different litters for each control and DDT lineage were used. The F1 generation offspring were obtained and aged to 90-day postnatal age and selected males and females bred within the control or DDT lineage. The F2 generation offspring were obtained and aged to 90 days and selected males and females from different litters bred to generate the F3 generation. No sibling or cousin breeding was used to avoid any inbreeding artifacts. All the males were killed at 120 days of age for epididymal sperm collection. Previously, disease onset was primarily observed between 6 and 12 months of age [1, 41], so postnatal day 120 (P120) males were used to avoid any disease artifacts. The only disease detectable at P120 is testis spermatogenic cell apoptosis [2]. Selected male testis at each generation for both the control and DDT lineages was used for spermatogenic cell apoptosis analysis. A significant level of apoptosis was observed in the F3 generation DDT lineage (Fig. 1b) supporting the transgenerational phenotype of the DDT model used. The sperm samples were collected from the cauda epididymis and then sonication used to destroy any contaminating somatic (e.g., epididymal) cells and partially remove the tails from the sonication-resistant heads of the sperm as described in “Methods”. The sperm heads were isolated and then used for epigenetic analysis.

### Sperm DNA methylation alterations

**Fig. 2:**
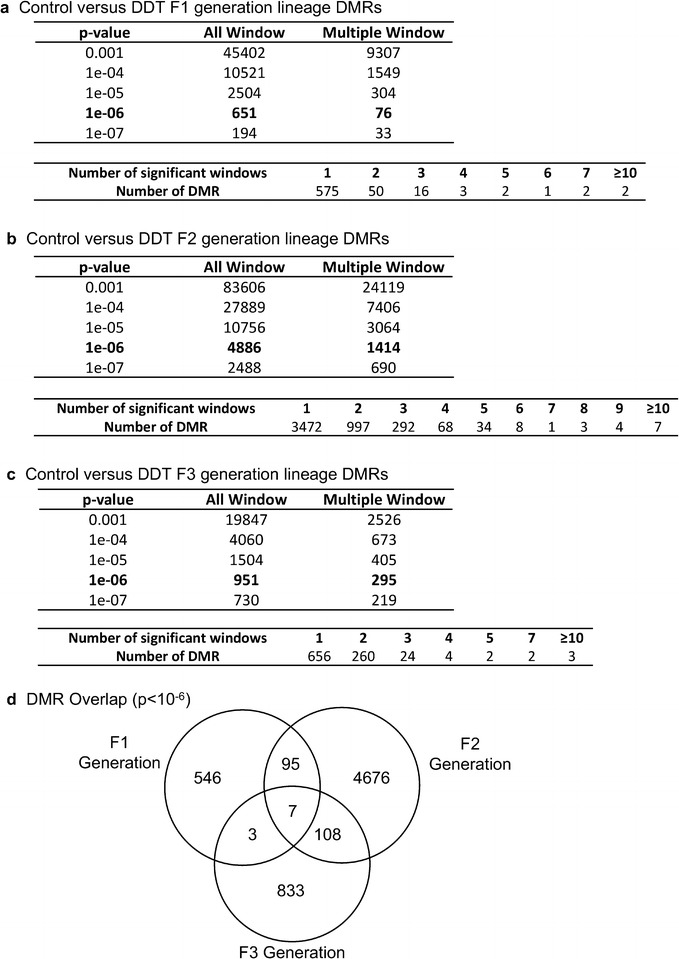
Differential DNA methylation regions (DMRs) analysis. **a** F1 generation control versus DDT lineage DMRs, **b** F2 generation control versus DDT lineage DMRs, **c** F3 generation control versus DDT lineage DMRs. The number of DMRs found using different *p* value cutoff thresholds is presented. The All Window column shows all DMRs. The Multiple Window column shows the number of DMRs containing at least two significant windows, **d** the DMR overlap (*p* ≤ 10^−6^) for the F1, F2 and F3 generation sperm

The differential DNA methylation regions (DMRs) between the control versus DDT lineages were determined using methylated DNA immunoprecipitation (MeDIP) followed by DNA sequencing (MeDIP-Seq) and bioinformatics analysis. The sperm DNA was isolated and then sonicated to 200–500-bp fragments and then a methyl-cytosine antibody used to immunoprecipitate methylated DNA fragments and a sequencing library generated to sequence 50-bp paired end (PE) regions of DNA to assess differential levels (read depths) of DNA methylation. The specific bioinformatics used involves edgeR and MEDIPS as described in “Methods”. A principle component analysis (PCA) demonstrated clustering separation of the control versus DDT lineage DMRs (Additional file 1: Figure S1A). In addition, a permutation analysis of the DMRs for the F1, F2 and F3 generations all showed the DMRs were not due to random chance (Additional file 2: Fig. S2). The DMRs for the F1, F2 and F3 generation sperm DNA were determined, and different threshold *p* values are shown in Fig. 2. A *p* value of < 10^−6^, corresponding FDR (false discovery rate) analysis < 0.05, was selected for comparison for all DMRs. The direct exposure F1 generation sperm had the lowest number of DMRs (Fig. 2a), the F2 generation the highest number of DMRs (Fig. 2b) and the transgenerational F3 generation an intermediate number of DMRs (Fig. 2c). A comparison of the DMRs at *p* < 10^−6^ demonstrated minimal overlap with a higher level of overlap between the F1 and F2 generations, as well as between the F2 and F3 generations (Fig. 2d). Therefore, the majority of the alterations in sperm DNA methylation were unique between the generations.

**Fig. 3:**
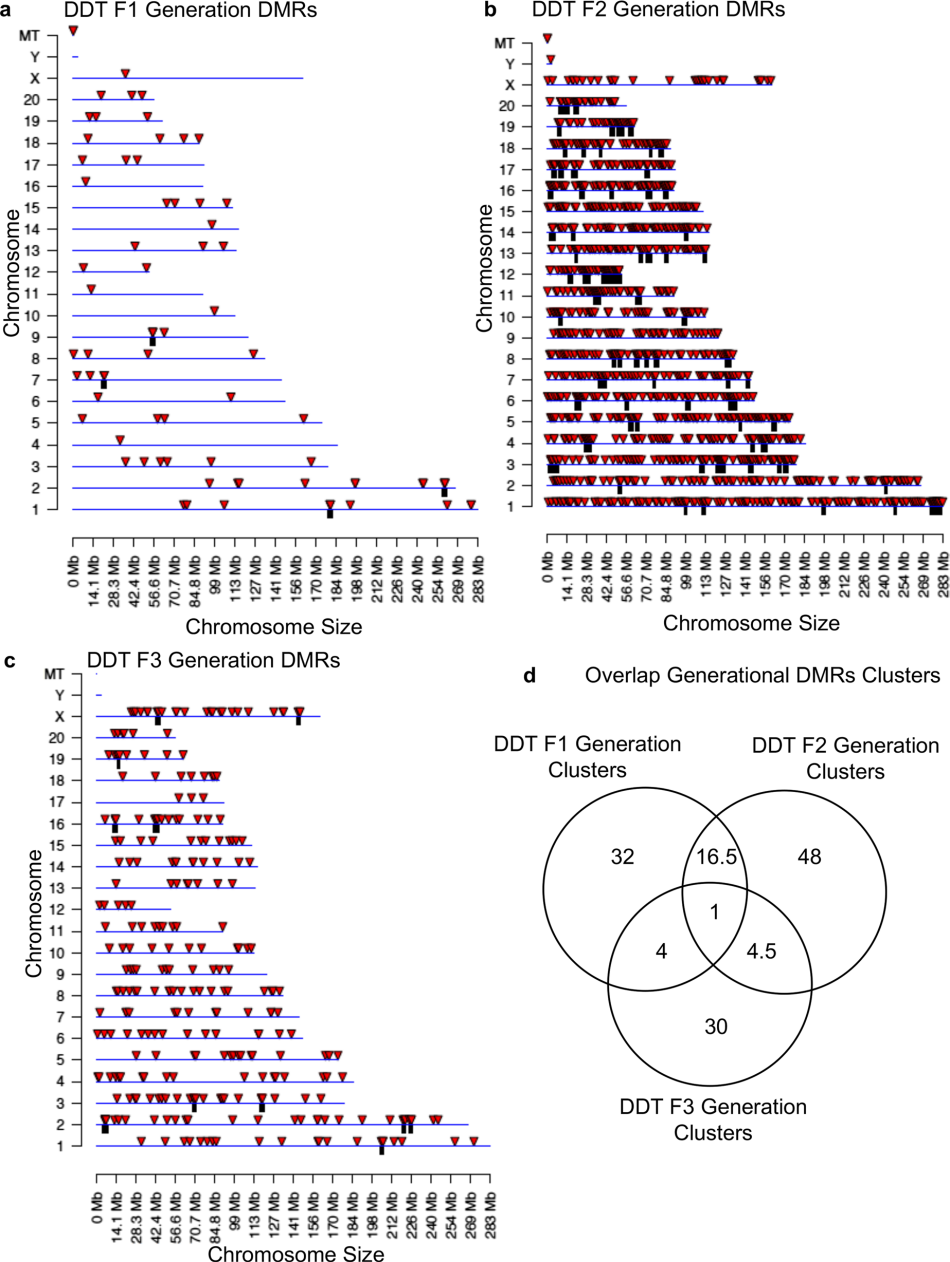
Chromosomal locations and overlaps of DMRs. **a** DDT F1 generation DMRs, **b** DDT F2 generation DMRs, **c** DDT F3 generation DMRs. The DMR locations on the individual chromosomes are shown with red arrowheads and clusters of DMRs with black boxes. Multiple Window DMRs at a *p* value threshold of 10^−6^ are shown, **d** overlap of the different generations DMR clusters

**Fig. 4:**
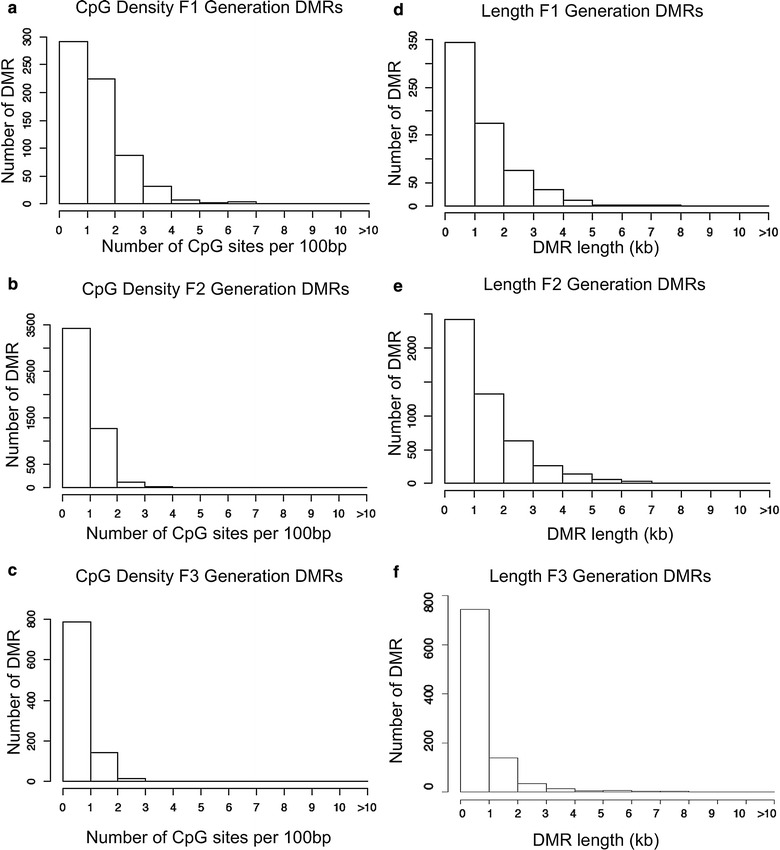
DMR CpG density for **a** F1 generation, **b** F2 generation and **c** F3 generation. Histograms of the number of DMRs at different CpG densities (CpG/100 bp). All DMRs at a *p* value threshold of 1e−06 are shown. DMR length (kb) for **d** F1 generation, **e** F2 generation and **f** F3 generation. Histograms of the number of DMR at different length (kb) are shown

The chromosomal locations of the DMRs with multiple statistically significant windows for each generation are shown in Fig. 3. All the chromosomes contain DMRs as indicated with red arrowheads, and some have over-represented clusters of DMRs indicated with black boxes. These different generation DMR clusters also are primarily distinct between the generations with minimal overlap (Fig. 3d). The genomic features of the DMRs identified were assessed and found to be similar to previously identified transgenerational DMRs [1, 42]. The CpG density of the DMRs for the F1, F2 and F3 generation sperm is shown in Fig. 4a–c. The CpG density of DMRs at *p* < 10^−6^ is presented and indicated that the predominant density is 1 CpG/100 bp with a range between 1 and 5 CpG. The lengths of the DMRs are presented in Fig. 4d–f and show the most predominant length of 1 kilobase and a range of 1–5 kb for each generation DMRs. Therefore, the DMRs are associated with CpG deserts with 10–20 CpG within the 1-kb DMRs [43]. The lists of DMRs with their chromosomal locations, size and CpG density are presented in Additional file 3: Table S1, Additional file 4: Table S2 and Additional file 5: Table S3 for the F1, F2 and F3 generation DMRs, respectively.

### Sperm ncRNA alterations

**Fig. 5:**
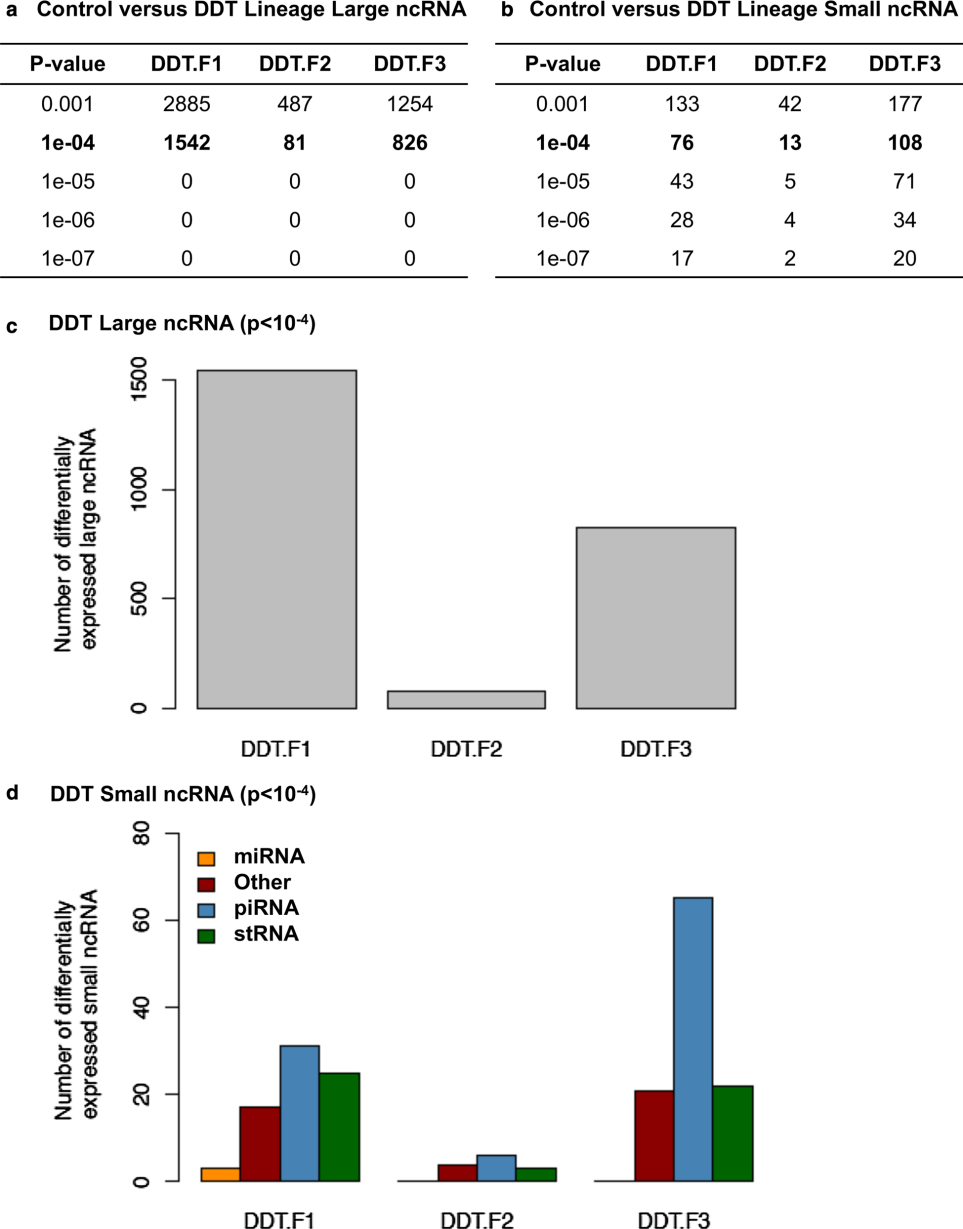
Non-coding RNA (ncRNA) differentially regulated in control versus DDT lineage F1, F2 and F3 generation sperm. **a** Long ncRNA and **b** small ncRNA numbers at different *p* value thresholds. **c** Long ncRNA (*p* < 10^−4^) for the F1, F2 and F3 generation correlated to the number of differential lncRNA. **d** Small ncRNA (*p* < 10^−4^) for the F1, F2 and F3 generation correlated to the number of miRNA, piRNA, stRNA and other sncRNA

The differential ncRNA present in sperm between the control versus DDT lineages was determined with next-generation sequencing using an RNA-Seq analysis previously described [32]. The sperm total RNA was collected and then separately small non-coding RNA (sncRNA) isolated and analyzed. The long non-coding RNA (lncRNA) was analyzed from the total RNA extraction and appropriate sequencing libraries prepared as described in “Methods”. The differential ncRNA levels (read depths) were determined for the F1, F2 and F3 generation sperm with a comparison of the control versus DDT lineage sperm. The long ncRNA for each generation with several *p* value thresholds is shown in Fig. 5a. The *p* value used for subsequent analysis selected was *p* < 10^−4^, with corresponding FDR < 0.1. The small ncRNA for each generation with several *p* value thresholds is shown in Fig. 5b. The *p* value used for subsequent small ncRNA analysis selected was also *p* < 10^−4^. The long ncRNA had an order of magnitude higher number of ncRNA than that observed with the small ncRNA. The F1 and F3 generation numbers were higher in number than the F2 generation for the long ncRNA (Fig. 5c). The small ncRNA was separated into categories with the piRNA being higher in number than others and the small tRNA (stRNA) being the next highest (Fig. 5d). The micro-RNA (miRNA) was low in number, and a mixture of uncategorized RNA (other) was present as well. Therefore, each generation had differential ncRNA present and differences are present between the generations.

**Fig. 6:**
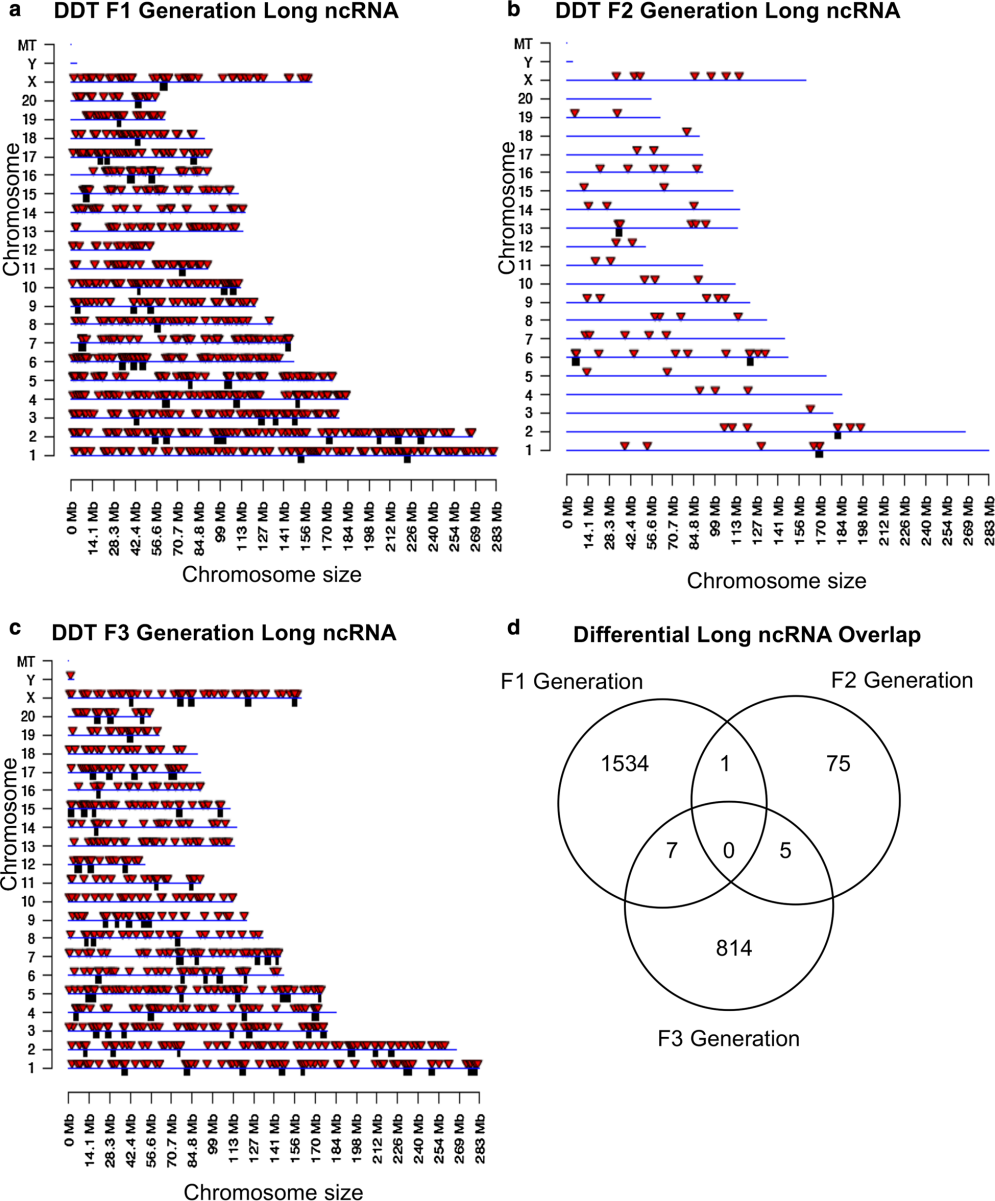
Chromosomal locations of long ncRNA for the **a** F1 generation, **b** F2 generation and **c** F3 generation sperm. The lncRNA locations on the individual chromosomes are indicated with red arrowheads and clusters with black boxes. The long ncRNA at a *p* value threshold of 10^−4^ is shown. **d** The overlap between the long ncRNA (*p* < 10^−4^) in the three DDT generations. Overlaps were determined based on common ncRNA names

**Fig. 7:**
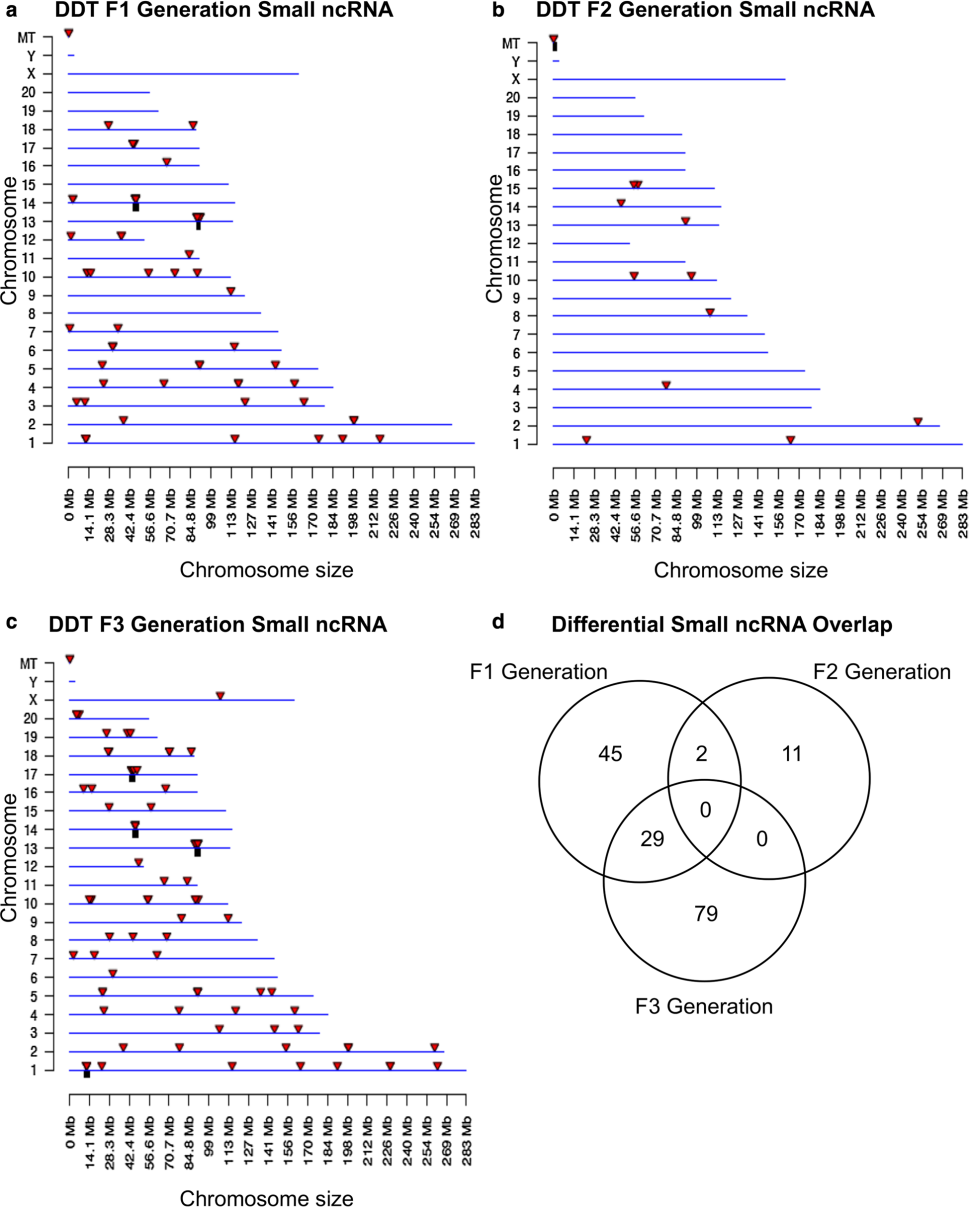
Chromosomal locations of small ncRNA for the **a** F1 generation, **b** F2 generation and **c** F3 generation. The sncRNA locations on the individual chromosomes are indicated with red arrowheads and clusters with black boxes. The sncRNA at an FDR-adjusted *p* value threshold of *p* < 10^−4^ is shown. There are four sncRNAs in the F1 generation and 6 sncRNAs in the F3 generation with unknown chromosome locations. **d** The overlap between the sncRNAs (*p* < 10^−4^) in the three DDT generations. Overlaps were determined based on common RNA names

The chromosomal locations of the differential ncRNA are presented for long ncRNA in Fig. 6. The red arrowheads identify individual lncRNA, and overlapping clusters of lncRNA are shown in black boxes. The F1, F2 and F3 generation long ncRNA is present on all chromosomes (Fig. 6a–c). The analysis of the long ncRNA overlap between the generations indicated the vast majority of differential ncRNA was distinct for each generation (Fig. 6d). The chromosomal locations of the differential ncRNA are presented for the small ncRNA in Fig. 7a–c. The overlap of specific small ncRNA between the generations is shown in Fig. 7d. The majority of differential sncRNA was unique for each generation with a reasonable overlap observed between the F1 and F3 generations for 29 small ncRNA. Therefore, both the long and small differential ncRNA was predominantly distinct from the transgenerational F3 generation ncRNA. The lists of differential ncRNA separated by category for identification, chromosomal location, size, statistics and gene associations are presented in Additional file 6: Table S4, Additional file 7: Table S5 and Additional file 8: Table S6 for the F1, F2 and F3 generations, respectively.

### Sperm histone alterations

**Fig. 8:**
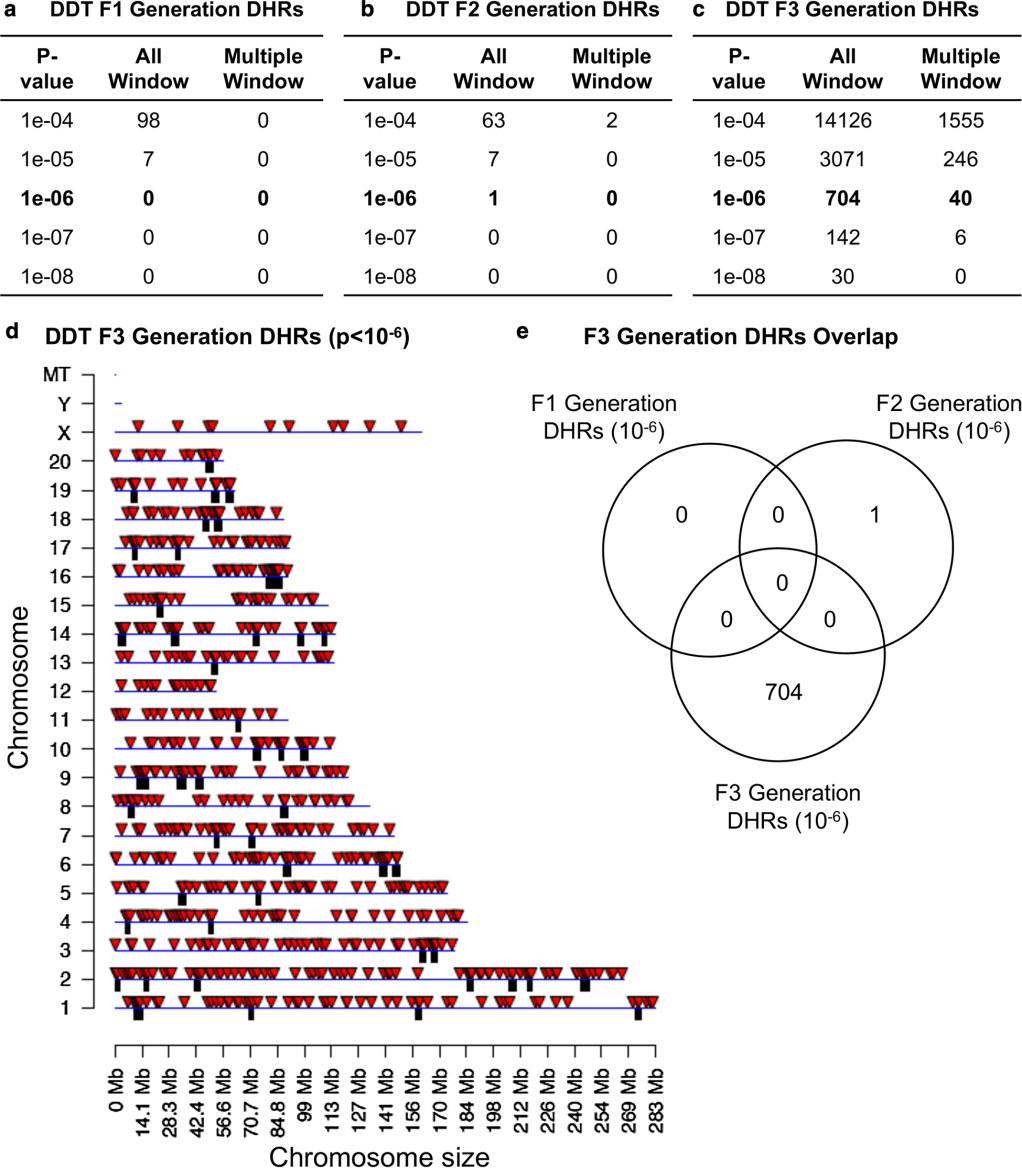
DDT differential histone retention sites (DHRs) in the **a** F1 generation, **b** F2 generation and **c** F3 generation. The number of DHRs found using different *p* value cutoff thresholds. The All Window column shows all DHRs. The Multiple Window column shows the number of DHRs containing at least two significant windows. **d** Chromosomal locations of F3 generation DHRs on individual chromosomes indicated by red arrowheads and DHR clusters with black boxes. All DHRs at a *p* value threshold of 1e-06 are shown. **e** The DMR overlap (*p* < 10^−6^) for the F1, F2 and F3 generation sperm

The differential histone retention regions (DHRs) for the F1, F2 and F3 generation for the control versus DDT lineage sperm were analyzed. The procedure involved assessment of differential histone retention sites (DHRs) through a comparison of the control versus DDT lineage sperm using the edgeR analysis similar to that used for the DNA methylation analysis. Interestingly, negligible DHRs were detected at *p* < 10^−6^ in the F1 or F2 generation sperm when the control versus DDT lineage histone H3 chromatin immunoprecipitation H3-ChIP-Seq data were compared (Fig. 8a, b). Only a small number were detected at *p* < 10^−4^, but were not confirmed with a false discovery rate (FDR) analysis (*p* < 0.1). In contrast, the F3 generation comparison of the control versus DDT lineage sperm identified a significant number of DHRs at a variety of *p* value thresholds that were supported by FDR analysis (Fig. 8c). A PCA of the F3 generation DHRs demonstrated a clustered separation of the control versus DDT lineages (Additional file 1: Figure S1B). The chromosomal locations of these F3 transgenerational DHRs (*p* < 10^−6^) are presented in Fig. 8d. An overlap of these transgenerational DHRs with the F1 and F2 generation DHRs demonstrated transgenerational F3 generation DHRs were unique (Fig. 8e). Therefore, additional histone retention sites were induced transgenerationally in the F3 generation lineage sperm. Although transgenerational alterations in histone retention sites are induced, no alterations are observed in the direct exposure F1 or F2 generation sperm.

**Fig. 9:**
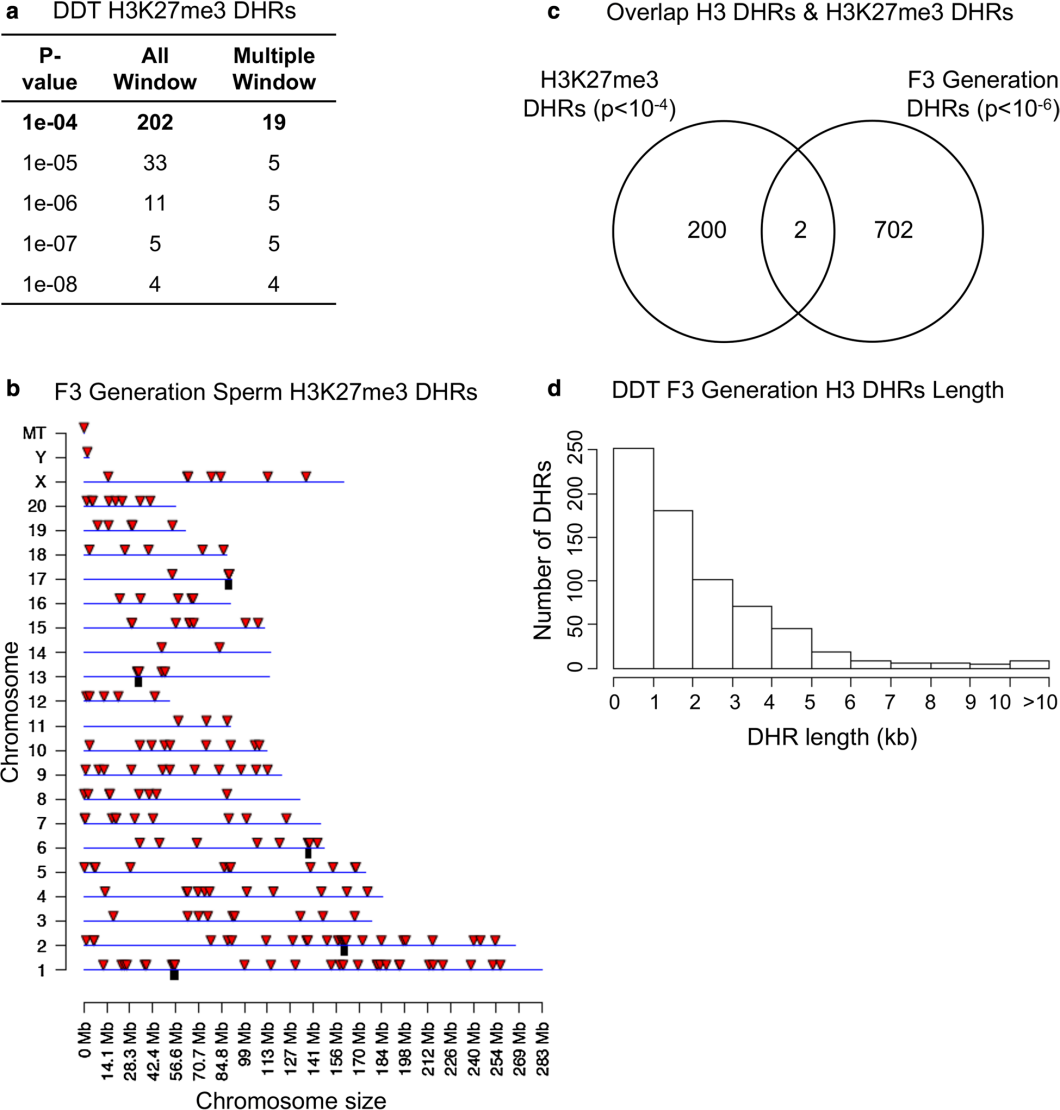
DDT F3 generation H3K27me3 DHRs. **a** The number of DHRs found using different *p* value cutoff thresholds. The All Window column shows all DHRs. The Multiple Window column shows the number of DHRs containing at least two significant windows. **b** The F3 generation sperm H3K27me3 DHR chromosomal locations on the individual chromosomes are indicated with red arrowheads and clusters with black boxes. All DHRs at a *p* value threshold of 1 × 10^−4^ are shown. **c** The overlap of F3 generation H3 DHRs (*p* < 10^−6^) and H3K27me3 DHRs (*p* < 10^−4^), **d** F3 generation H3 DHR lengths. All H3 DHRs at a *p* value threshold of < 10^−6^ are shown. F3 generation H3 DHR lengths (kb) versus the number of DHRs associated indicated

The analysis of total histone retention was calculated with the H3-ChIP-Seq analysis of sperm by determining the total amount (bp) having retained histones compared to the total length of the genome. The percent retained histone for the F1 and F2 generation control lineage sperm was 19%, while the F3 generation with increased DHRs for the control lineage sperm was 9% and DDT lineage was 18% histone retention. The length (kb) of the H3 DHRs varied between 1 and 6 kb, but was predominantly 1–2 kb (Fig. 9d). The DHRs for the F3 generation are listed with identification, location, size and associated genes in Additional file 9: Table S7 and Additional file 10: Table S8.

The final histone analysis assessed the potential alteration in histone modifications involving a histone H3K27me3 methylation alteration using an H3K27me3 ChIP-Seq analysis for the F3 generation control versus DDT lineage sperm. Analysis of the DHRs using the H3K27me3 analysis identified DHRs in the F3 generation sperm (Fig. 9a, b). The H3K27me3 histone modifications identified a smaller number of DHRs than the H3 histone DHRs, and they were distinct from each other in the F3 generation sperm (Fig. 9c). Therefore, transgenerational alterations in histone retention sites were more predominant than the histone modifications identified.

### Epimutation gene associations

**Fig. 10:**
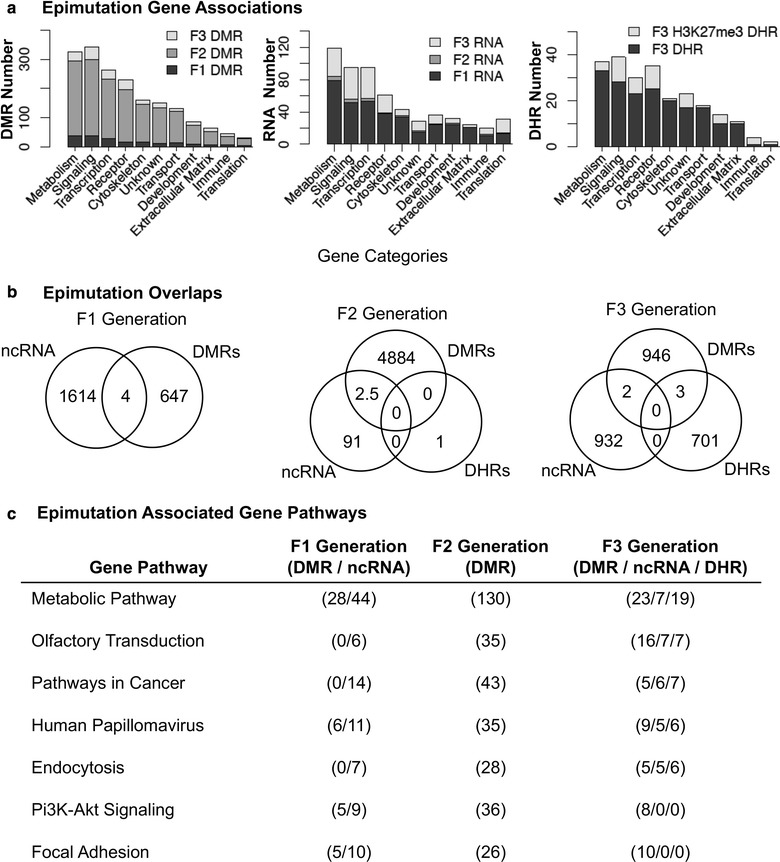
Epimutation overlaps and gene associations. **a** The number of DMR, DHR and ncRNA epimutation-associated genes is correlated to gene categories for the F1, F2 and F3 generations. **b** Overlaps of the F1, F2 and F3 generation epimutations between the DMRs, DHRs and ncRNAs. Gene pathways with epimutation-associated genes for the DMRs, DHRs and ncRNAs in the F1, F2 and F3 generations. The pathways with greater than five associated genes for each epimutation type are listed that were common between generations

The lists of DMRs, ncRNAs and DHRs for all the epigenetic alterations identified are presented in Additional file 1: Table S1, Additional file 2: Table S2, Additional file 3: Table S3, Additional file 4: Table S4, Additional file 5: Table S5, Additional file 6: Table S6, Additional file 7: Table S7 and Additional file 8: Table S8. The known gene associations for these epigenetic alterations (i.e., epimutations) are provided in these lists. Generally less than 20% of the epimutations had associated genes, so most were intergenic and not in an associated 10-kb proximity with genes. All the associated genes were categorized into relevant functions and the functional categories presented for each generation in Fig. 10a. For this analysis, all ncRNAs were combined. The top ten gene categories containing multiple genes for F1, F2 and F3 generations are presented for DMRs, ncRNAs and DHRs separately. Epimutations were found predominantly in the metabolism, transcription, signaling and receptor categories. The predominant gene categories for F1, F2 and F3 generation were similar for the DMRs, ncRNAs and DHRs (Fig. 10a). The number of epimutations between the generations was highest for DMRs in the F2 generation, highest for ncRNAs in the F1 generation and only present for DHRs in the F3 generation.

The integration of the different epigenetic alterations is presented in Fig. 10b. The overlap of DMRs, ncRNAs and DHRs suggests minimal overlap between the different epimutations within a generational comparison. Therefore, the different epigenetic alterations (DMRs, ncRNAs and DHRs) have different genomic locations with negligible overlap between epimutation types at each generation. A number of examples of epimutation associations were found within 100-kb regions, with one example provided in Additional file 11: Fig S3. The total number of associated genes with the DMRs, ncRNAs and DHRs is presented in Additional file 1: Table S1, Additional file 2: Table S2, Additional file 3: Table S3, Additional file 4: Table S4, Additional file 5: Table S5, Additional file 6: Table S6, Additional file 7: Table S7 and Additional file 8: Table S8, and due to the lack of overlap of the epimutations, there is negligible overlap between the genes. The final analysis used the epimutation-associated genes for DMRs, ncRNAs and DHRs separately and performed gene pathway analysis for each. The gene pathways with the highest number of associated genes for each epimutation and common between generations are listed and ranked in Fig. 10c. The analysis revealed seven pathways in common and each contained various numbers of epimutations. Therefore, the different epimutations targeted some common gene pathways. Examples of such pathways and associated genes are the cancer gene pathway and endocytosis pathway presented for the F3 generation DDT lineage in Additional file 12: Fig. S4 and Additional file 13: Fig. S5. Observations demonstrate 5 DMR, 5–6 ncRNA and 6–7 DHRs for each pathway, and all have the capacity to alter the pathways. Although the epimutations do not overlap, they can potentially target similar pathways.

## Discussion

The current study investigated several epigenetic processes in sperm correlated with DDT-induced epigenetic transgenerational inheritance of disease. DDT has previously been shown to promote a number of transgenerational diseases including obesity in 50% of the F3 generation male and female populations [15]. The analysis of DNA methylation, ncRNA and histone retention in the same purified sperm samples from F1, F2 and F3 generation control versus DDT lineage rats provides the most comprehensive analysis of epigenetic alterations associated with environmentally induced epigenetic transgenerational inheritance of germline epimutations. In addition, a comparison of the F1, F2 and F3 generations was made to assess the differences between the direct exposure F1 and F2 generations versus the transgenerational F3 generation. As discussed, the gestating female exposure during gonadal sex determination directly exposes the F1 generation fetus, and the germline that will generate the F2 generation, such that the transgenerational F3 generation is the first generation with no direct exposure. Although the F2 generation phenotype may be a combination of direct germline exposure and programmed transgenerational exposure [1, 25], it is not possible to distinguish between the two. Therefore, a detailed comparison of the different generations provides insights into the epigenetic transgenerational mechanisms.

The original epigenetic process associated with the F3 generation sperm identified was alterations in DNA methylation [2, 44]. Subsequently a large number of studies identified alterations in DNA methylation associated with epigenetic transgenerational inheritance of germline epimutations [1]. The exposures associated with altered sperm DNA methylation involve a number of environmental factors from nutrition to toxicants [1, 45]. One previous study investigated the difference between the F1 and F3 generation in alterations of sperm DNA methylation. A distinct set of DMRs was identified in vinclozolin-induced sperm DNA methylation [26]. Non-coding RNAs have also been shown to mediate the epigenetic transgenerational inheritance of sperm epimutations and pathologies [31–33, 46]. One of the initial studies identified alterations in ncRNA and used sperm ncRNA extracts to inject into oocytes and promote the transgenerational behavioral phenotype observed [31]. The ability to promote the transgenerational phenotype provides one of the first functional links with a sperm epigenetic alteration. Subsequently a number of studies have documented alterations in sperm ncRNA associated with the transgenerational inheritance of disease [31–33, 46]. Histone modifications were found to associate with environmentally induced inheritance of phenotypes in *C. elegans* and drosophila [18, 47], but have not been reported in mammals. Alterations in sperm histone modifications and retention have been shown in human and rodent sperm to associate with infertility [34, 48], but the role in transgenerational inheritance was only recently suggested [36]. The current study provides the first combined analysis to investigate the integration of the different epigenetic processes in environmentally induced transgenerational inheritance of disease.

Comparison of the F1, F2 and F3 generation sperm epimutations demonstrated primarily distinct alterations at each generation. For DNA methylation, the majority of DMRs were unique for each generation with more overlap between the F1 and F2 generations, as well as the F2 and F3 generations (Fig. 2d). Therefore, the direct exposure F1 generation sperm and transgenerational F3 generation sperm DMRs are distinct and associate with the often different pathologies observed between the generations [1, 2, 7–15]. The F2 generation appears to be a combination of direct exposure and transgenerational with some overlap with each, but again is primarily distinct. The ncRNA analyses examined both the small ncRNA and long ncRNA that have been shown to have distinct functions [46]. The small ncRNAs often target specific genes through alterations in mRNA stability [49]. The long ncRNAs often regulate transcriptional machinery to act on regions of the chromosome and target multiple genes. An example involves imprinted gene DNA methylation alterations that promote long ncRNA expression to act distally for megabases to influence the expression of multiple genes, termed imprinting control regions or epigenetic control regions [50]. The ncRNA analysis in the F1, F2 and F3 generation control versus DDT lineage sperm identified differential ncRNA levels. The long ncRNA was unique for each generation with negligible overlap (Fig. 6d). The number of long ncRNA for the F1 and F3 generation sperm was an order of magnitude higher than the small ncRNA. The small ncRNA had greater overlap between the F1 and F3 generations and F1 and F2 generations, but not the F2 and F3 generation (Fig. 7d). The piRNAs are the most common class of small ncRNA for each generation (Fig. 5d). Interestingly, vinclozolin-induced epigenetic transgenerational inheritance of sperm sncRNA alterations was found to predominantly promote small tRNA (stRNA) [32], so the current observations show exposure specificity. Therefore, concurrent DNA methylation and ncRNA sperm alterations are associated with epigenetic transgenerational inheritance.

During mammalian spermatogenesis, the histones are replaced by protamines to compact the DNA to fit into the head of the sperm [51]. A small percentage (5–10%) of histone retention sites are maintained [34] and are proposed to have a potential role in the zygote and early embryonic gene expression [52]. In the current study 9–19% histone retention was calculated for the DHRs. The potential that alterations in histone retention may be part of epigenetic transgenerational inheritance was investigated. Previously, the control lineage was found to contain a core set of histones that are highly reproducible and not changed between the F1, F2 and F3 generations [36]. This core set of histones was not influenced in the DDT lineage male sperm, so was not influenced during the F1, F2 or F3 generation sperm. A small number of alterations were observed in the F3 generation, but the majority of the core histone sites were not modified [36]. Due to the lack of variation and reproducibility, and lack of exposure effects, these core histone sites may be critical for development and appear to be highly conserved [36]. Interestingly, when differential histone retention regions (DHRs) were investigated, negligible DHRs were observed in either the F1 generation or F2 generation control versus DDT lineage sperm (Fig. 8). Therefore, the direct DDT exposure does not appear to alter the histone retention sites or promote DHRs. In contrast, the F3 generation control versus DDT lineage sperm DHRs was dramatically increased in comparison with the core histone retention sites. Since histone modifications (methylation or acetylation) have been the focus of most histone analyses, the potential transgenerational alterations in a common histone modification H3K27me3 were investigated, in addition to the histone H3 retention sites. The F3 generation control versus DDT lineage sperm comparison identified differential H3K27me3 DHRs that were reduced in number compared to the H3 DHRs (Fig. 9). Overlap of the H3 DHRs and H3K27me3 DHRs demonstrated negligible overlap at a similar statistical cutoff. Therefore, alterations in histone retention sites and to a lesser degree histone modifications (i.e., methylation) are associated with the DDT-induced transgenerational F3 generation sperm. In contrast to the DMRs and ncRNAs, direct exposure in the F1 and F2 generation sperm did not alter histone retention, while alterations in histone retention and modification were observed in the transgenerational F3 generation sperm. Therefore, alterations in DMRs, ncRNAs and DHRs are all present in the transgenerational F3 generation sperm and appear to be integrated to mediate the epigenetic transgenerational inheritance phenomenon.

In the investigation of the integration of the DMR, ncRNA and DHR epimutations, the initial analysis examined the potential overlap of the genomic sites between the epimutations. The chromosomal locations of each of the DMR, ncRNA and DHR epimutations were primarily distinct with negligible overlap. Clusters of epimutations were identified with DMRs, ncRNAs and DHRs, but overlap of the clusters was also negligible. In considering the genomic locations of the DMR methylation, ncRNA and DHRs alterations they are distinct. The genomic features of the epimutations demonstrated similar size and presence in CpG deserts [53] for the DMRs and DHRs. Therefore, the transgenerational sperm epimutations often are present in regions with similar genomic features, but localization was generally distinct. Since ncRNA can act distinctly within the genome, in particular long ncRNA [54], the ncRNA may help mediate the actions of the various epimutations that are distally located.

The DMRs, DHRs and ncRNA demonstrated negligible overlap between the different types of epimutations unless regions of 100 kb size are considered (Additional file 11: Fig. S3). The gene associations of the transgenerational epimutations identified similar categories of genes such that common cellular processes are influenced by each of the epigenetic alterations. A comparison of the F1, F2 and F3 generation epimutation-associated genes identified no overlap between the different types of epimutations. The analysis of associated gene pathways for the epimutations demonstrated the different epimutations (DMRs, ncRNA and DHRs) can influence common pathways so may be integrated on a functional level.

Comparison of the epimutations in the F1, F2 and F3 generations provided insights into the transient direct exposure effects of environmental exposures and transgenerational impacts of the exposures. The direct exposures of the F1 and F2 generation (Fig. 1a) were distinct from the transgenerational F3 generation sperm epimutations. Although DNA methylation and ncRNA were altered in the direct exposed F1 and F2 generation sperm, the histone retention sites or modifications were not significantly altered. Therefore, the histone retention and modification appears to be resistant to alterations from direct exposure. In contrast, the DNA methylation and ncRNA were altered with direct exposure, but were distinct between the different generations. The transgenerational F3 generation alterations in DNA methylation, ncRNA and histone alterations are distinct from the direct exposure. The proposal is that transgenerational epimutations will become permanent and mediate the epigenetic transgenerational inheritance in subsequent generations. Further studies are in progress to investigate this phenomenon. The mechanism of how the transgenerational epimutations are developed appears to be linked with the direct exposure alterations in the F1 and F2 generation to alter the development of the primordial germ cells (PGCs). This altered development of the PGCs appears to become permanently established so that the transgenerational epigenetic programming becomes imprinted and mediates the development of germline epimutations which will promote the epigenetic transgenerational inheritance phenomenon. Although epigenetic alterations in the transgenerational PGCs have been observed [55, 56], further investigation of PGC development in mediating the transgenerational mechanism is required. The current study demonstrates distinct epigenetic alterations between the direct exposed generations and the transgenerational generation sperm. It is important to understand that the mature sperm have condensed DNA to allow the DNA to fit into the head of the sperm, such that no expression of ncRNA or genes occurs nor is the expression machinery present. Therefore, the ncRNA in sperm is derived during spermatogenesis in the testis and then stored in the sperm. Some gene mRNA has been identified in sperm, but variability, stability and unclear function of these stored mRNAs have suggested no relation to sperm biology [57]. The developmental origins and functional role of these epimutations now need to be considered further regarding the unique transgenerational F3 generation germline epigenetic alterations. It is not simply the induction of an epigenetic alteration during direct exposure that is then maintained generationally, but instead a more complicated development of the transgenerational epimutations.

## Conclusions

Previous studies have suggested specific epigenetic processes are mediating the epigenetic transgenerational inheritance phenomenon. The current study provides the first concurrent analysis of DNA methylation, ncRNA and histone alterations to suggest all three are involved in transgenerational inheritance. Since each epigenetic process has a unique function, it is not surprising they would be integrated to facilitate transgenerational inheritance. The developmental role of DNA methylation and its ability to respond to environmental stressors, the distal actions of ncRNA to help integrate the other epigenetic marks and alter gene expression and the ability of sperm histone retention and modifications to alter early development of the zygote and early embryo all may work together to facilitate the epigenetic transgenerational phenomenon. Due to the potential impacts of this on disease etiology and other areas of biology such as evolution, further investigation and elucidation of these integrated epigenetic processes is required.

## Methods

### Animal studies and breeding

Female and male rats of an outbred strain Hsd:Sprague-Dawley SD^®^™ (Harlan) at about 70 and 100 days of age were fed ad lib with a standard rat diet and received ad lib tap water for drinking. To obtain time-pregnant females, the female rats in proestrus were pair-mated with male rats. The sperm-positive (day 0) rats were monitored for diestrus and body weight. On days 8 through 14 of gestation [58], the females received daily intraperitoneal injections of DDT (25 mg/kg BW/day) or dimethyl sulfoxide (DMSO) in oil (vehicle). The DDT was obtained from Chem Service Inc. (West Chester, PA) and was injected in a 20 µl DMSO/oil vehicle as previously described [26]. Treatment lineages are designated “control” or “DDT” lineages. The gestating female rats treated were designated as the F0 generation. The offspring of the F0 generation rats were the F1 generation. Non-littermate females and males aged 70–90 days from F1 generation of control or DDT lineages were bred to obtain F2 generation offspring. The F2 generation rats were bred to obtain F3 generation offspring. Individuals were maintained for 120 days and euthanized for sperm collection. The F1–F3 generation offspring were not themselves treated directly with DDT. The control and DDT lineages were housed in the same room and racks with lighting, food and water as previously described [26, 41, 59]. All experimental protocols for the procedures with rats were pre-approved by the Washington State University Animal Care and Use Committee (IACUC approval # 02568-39).

Testis sections were examined by transferase-mediated dUTP nick-end labeling (TUNEL) assay (In situ cell death detection kit, Fluorescein, Sigma, St. Louis, MO) to assess spermatogenic cell apoptosis. The frequency of spermatogenic cell apoptosis was determined histologically with a florescent microscope to identify apoptotic cells and count apoptotic cells per area of the slide. Multiple slides per animal were examined to assess the mean ± SD apoptotic cells per animal.

### Epididymal sperm collection and DNA and RNA isolation

The epididymis was dissected free of connective tissue, a small cut made to the cauda and tissue placed in 5 ml of 1 × PBS for 10 min at 37 °C and then kept at 4 °C to immobilize the sperm. The epididymal tissue was minced and the released sperm centrifuged at 13,000×*g* and the pellet stored at − 20 °C until processed further. The sample was resuspended and sonicated to destroy any contaminating somatic cells. This removed any somatic cell contamination due to the sonication resistance of the sperm head [60]. The pellet was resuspended in NIM buffer, and then one hundred microliters of sperm suspension was combined with 820 μl DNA extraction buffer and 80 μl 0.1 M DTT. The sample was incubated at 65 °C for 15 min. Following this incubation, 80 μl proteinase K (20 mg/ml) was added and the sample incubated at 55 °C for at least 2 h under constant rotation. Then 300 μl of protein precipitation solution (Promega Genomic DNA Purification Kit, A795A) was added, and the sample mixed thoroughly and incubated for 15 min on ice. The sample was centrifuged at 13,500 rpm for 20 min at 4 °C. One milliliter of the supernatant was transferred to a 2-ml tube, and 2 μl of glycoblue and 1 ml of cold 100% isopropanol were added. The sample was mixed well by inverting the tube several times and then left in – 20 °C freezer for at least 1 h. After precipitation, the sample was centrifuged at 13,500 rpm for 20 min at 4 °C. The supernatant was taken off and discarded without disturbing the (blue) pellet. The pellet was washed with 70% cold ethanol by adding 500 μl of 70% ethanol to the pellet and returning the tube to the freezer for 20 min. After the incubation, the tube was centrifuged for 10 min at 4 °C at 13,500 rpm and the supernatant discarded. The tube was spun again briefly to collect residual ethanol to bottom of tube, and then as much liquid as possible was removed with gel-loading tip. Pellet was air-dried at RT until it looked dry (about 5 min). Pellet was then resuspended in 100 μl of nuclease-free water. Equal amounts of DNA from several (3–6) individual sperm samples were used to produce three DNA pools per lineage and employed for methylated DNA immunoprecipitation (MeDIP).

### RNA isolation

The F1–F3 generation DDT and control lineage male epididymal sperm were collected and processed as previously described and stored at − 80 °C until use [50]. The total RNA (messenger RNA; long, non-coding RNA; ribosomal RNA; transfer RNA; sRNA) was isolated using the mirVana miRNA Isolation Kit (Life Technologies) following the manufacturer’s instructions with modifications at the lysis stage. In brief, after the addition of lysis buffer, the sperm pellets were manually homogenized, followed by a 20-min incubation at 65 °C. Samples were then placed on ice, and the default protocol was resumed. For quality control, RNA integrity numbers (RIN) were obtained by RNA 6000 Pico chips run on an Agilent 2100 Bioanalyzer (Agilent). A RIN of 2–4 indicates good sperm RNA quality. Concentration was determined using the Qubit RNA HS Assay Kit (ThermoFisher). Biological replicates of sperm were pooled by equal RNA content and were concentrated using Agencourt AMPure XP beads (Beckman Coulter). Some pools had underrepresented replicates due to low concentration. In this case, if the Agilent profile was normal, the maximum RNA content from the replicate was used in the pool and the pool was concentrated. Abnormal Agilent profiles excluded the following samples from the pools: F1 DDT pool 2, sample 2; F2 DDT pool 1, samples 3 and 4. The pools and samples that were underrepresented are as follows: F1 control pool 3, samples 1 and 2; F2 control pool 2, sample 3; F2 DDT pool 1, samples 1 and 2; and F2 DDT pool 2, samples 1 and 5. Equal amounts of each pool were used in the final analysis.

### Methylated DNA immunoprecipitation MeDIP

Methylated DNA immunoprecipitation (MeDIP) with genomic DNA was performed as follows: Rat sperm DNA pools were generated using the appropriate amount of genomic DNA from each individual for 3 pools each of control and DDT lineage animals. Genomic DNA was sonicated using the Covaris M220 the following way: The pooled genomic DNA was diluted to 130 μl with TE into the appropriate Covaris tube. Covaris was set to 300-bp program, and the program was run for each tube in the experiment. Ten microliters of each sonicated DNA was run on 1.5% agarose gel to verify fragment size. The sonicated DNA was transferred from the Covaris tube to a 1.7-ml microfuge tube and the volume measured. The sonicated DNA was then diluted with TE buffer (10 mM Tris-HCl, pH7.5; 1 mM EDTA) to 400 μl, heat-denatured for 10 min at 95 °C and then immediately cooled on ice for 10 min. Then 100 μl of 5 × IP buffer and 5 μg of antibody (monoclonal mouse anti-5-methyl cytidine; Diagenode #C15200006) were added to the denatured sonicated DNA. The DNA-antibody mixture was incubated overnight on a rotator at 4 °C.

The following day magnetic beads (Dynabeads M-280 sheep anti-mouse IgG; 11201D) were pre-washed as follows: The beads were resuspended in the vial; then, the appropriate volume (50 μl per sample) was transferred to a microfuge tube. The same volume of washing buffer (at least 1 mL) was added, and the bead sample was resuspended. Tube was then placed into a magnetic rack for 1–2 min, and the supernatant discarded. The tube was removed from the magnetic rack, and the beads washed once. The washed beads were resuspended in the same volume of 1 × IP buffer as the initial volume of beads. Fifty microliters of beads was added to 500 μl of DNA-antibody mixture from the overnight incubation and then incubated for 2 h on a rotator at 4 °C.

After the incubation the bead-antibody–DNA complex was washed three times with 1 × IP buffer as follows: The tube was placed into magnetic rack for 1–2 min and the supernatant discarded and then washed with 1 × IP buffer 3 times. The washed bead-DNA solution is then resuspended in 250 μl digestion buffer with 3.5 μl proteinase K (20 mg/ml). The sample was then incubated for 2–3 h on a rotator at 55°, and then 250 μl of buffered phenol–chloroform–isoamylalcohol solution was added to the supernatant and the tube vortexed for 30 s and then centrifuged at 14,000 rpm for 5 min at room temperature. The aqueous supernatant was carefully removed and transferred to a fresh microfuge tube. Then 250 μl of chloroform was added to the supernatant from the previous step, vortexed for 30 s and centrifuged at 14,000 rpm for 5 min at room temperature. The aqueous supernatant was removed and transferred to a fresh microfuge tube. To the supernatant 2 μl of glycoblue (20 mg/ml), 20 μl of 5 M NaCl and 500 μl ethanol were added and mixed well and then precipitated in − 20 °C freezer for 1 h to overnight.

The precipitate was centrifuged at 14,000 rpm for 20 min at 4 °C and the supernatant removed, while not disturbing the pellet. The pellet was washed with 500 μl cold 70% ethanol in − 20 °C freezer for 15 min and then centrifuged again at 14,000 rpm for 5 min at 4 °C, and the supernatant discarded. The tube was spun again briefly to collect residual ethanol to bottom of tube and as much liquid as possible was removed with gel-loading tip. Pellet was air-dried at RT until it looked dry (about 5 min) and then resuspended in 20 μl H_2_O or TE. DNA concentration was measured in Qubit (Life Technologies) with ssDNA kit (Molecular Probes Q10212).

### ncRNA sequencing analysis

Total RNA was used to construct large mRNA and ncRNA libraries for each pool. Libraries were constructed using the KAPA Stranded RNA-Seq Library Preparation Kit with RiboErase, according to the manufacturer’s instructions, with some modifications. The adaptor and barcodes used were from NEBNext Multiplex Oligos for Illumina. Prior to PCR amplification, libraries were incubated at 37 °C for 15 min with the USER enzyme (NEB). PCR cycle number was determined using qPCR with the KAPA RealTime Library Amplification Kit before final amplification. Size selection (300–700 bp) was performed using Agencourt AMPure XP beads (Beckman Coulter). Quality control was performed using Agilent DNA high sensitivity chips (Agilent) and Qubit dsDNA high sensitivity assay (ThermoFisher) (Additional file 14: Fig. S6). Libraries were pooled and loaded onto an Illumina NextSeq High Output v2 1X75 chip and sequenced on an Illumina NextSeq 500 sequencer. Bioinformatics analysis was used to separate mRNA libraries from ncRNA libraries (see “ncRNA bioinformatics” section).

Prior to small library preparation, pooled total sperm RNA samples were enriched for small RNAs using the supplemental protocol for miRNA enrichment with SPRIselect by Beckman Coulter. Small RNA-enriched samples were used for small RNA library preparation, using the NEBNext Multiplex Small RNA Library Prep Set for Illumina, and barcoded with NEBNext Multiplex Oligos for Illumina. Size selection (135–170 bp) was performed using the Pippin Prep (Sage Science). Quality control was performed using Agilent DNA high sensitivity chips (Agilent) and Qubit dsDNA high sensitivity assay (ThermoFisher). Libraries were pooled and loaded onto an Illumina NextSeq High Output v2 1 × 75 chip and sequenced on an Illumina NextSeq 500 sequencer.

### Histone chromatin immunoprecipitation ChIP-Seq-

Histone chromatin immunoprecipitation with genomic DNA was performed as follows: Rat sperm pools were generated using a total of 8 million sperm for 3 pools of control and DDT lineage animals. The control pools contained 5–6 individuals for a total of *n* = 17 rats, and the DDT pools contained 4 individuals for a total of *n* = 12 rats per exposure group. Sperm from each animal was sonicated 10 s using a Fisher Sonic Dismembrator Model 300 and then counted individually on a Neubauer improved cell prior to pooling. The sperm pools were filled up to 1 ml with 1 × PBS. To reduce disulfide bonds, 50 µl of 1 M DTT was added to each pool and incubated for 2 h at room temperature under constant rotation. To quench any residual DTT in the reaction, 120 µl of 1 M of NEM (*N*-ethylmaleimide, Thermo Scientific) was then added and incubated for 30 min at room temperature under constant rotation. The sperm cells were pelleted at 2000 g for 5 min at room temperature, and the supernatant discarded. Pellets were resuspended in 1 × PBS. The mixture was spinned again at 2000 g for 5 min at room temperature, and the supernatant was discarded.

The sperm cells were then resuspended in “complete buffer” in a ratio of 2 million sperm cells in 50 µl (as described by Hisano et al. [61]). “Complete buffer” was supplemented with Tergitol 0.5% and DOC (sodium deoxycholate, Sigma-Aldrich 30970). Fifty microliters of this mix was added to each aliquot. The tubes were homogenized and incubated for 20 min on ice. 10 Kuntz units of MNase (Roche, cat. no. 10107921001) were added, and the samples incubated for 5 min at 37 °C. The reaction was stopped by the addition of 2 µl of EDTA 0.5 M.

Whether the samples were treated using the MNase and 10 µl of each sample was run on a 1.5% agarose gel to verify fragment size. The aliquots from the same sample were pooled back together and centrifuged at 12,500 rpm for 10 min at room temperature. The supernatant was transferred to a fresh microfuge tube. Sixty-five microliters of protease inhibitors was added in each sample along with 3 µl of antibody (monoclonal rabbit anti-histone H3, Millipore Sigma 05-928). The DNA-antibody mixture was incubated overnight on a rotator at 4 °C. The following day, magnetic beads (ChIP-Grade protein G magnetic beads, Cell Signaling 9006) were pre-washed as follows: The beads were resuspended in the vial; then, the approximate volume (30 µl per sample) was transferred to a microfuge tube. The same volume of washing buffer (at least 1 ml) was added, and the bead sample was resuspended. Tube was then placed into a magnetic rack for 1–2 min, and the supernatant discarded. The tube was removed from the magnetic rack, and the beads washed once. The washed beads were resuspended in the same volume of 1 × IP buffer as the initial volume of beads. Thirty microliters of beads were added to the DNA-antibody mixture from the overnight incubation and then incubated for 2 h on a rotator at 4 °C. After the incubation, the beads-antibody–DNA complex was washed three times with 1 × IP buffer as follows: The tube was placed into a magnetic rack for 1–2 min and the supernatant discarded and then washed with 1 × IP buffer 3 times. The washed beads-DNA solution is then resuspended in 300 µl of digestion buffer and 3 µl proteinase K (20 mg/ml). The sample was then incubated for 3 h on a rotator at 56 °C. Then 300 µl of buffered phenol–chloroform–isoamylalcohol solution was added to the supernatant and the tube vortexed for 30 s and then centrifuged at 12,500 rpm for 10 min at room temperature. The aqueous supernatant was carefully removed and transferred to a fresh microfuge tube. Then 2 µl of glycoblue (20 mg/ml), a one-tenth volume of 3 M sodium acetate and two volumes of ethanol were added. The mixture was vortexed 30 s and then stored overnight in − 20 °C freezer.

The precipitate was centrifuged at 12,500 rpm for 30 min at 4 °C and the supernatant removed, while not disturbing the pellet. The pellet was washed with 500 µl cold 70% ethanol and then centrifuged again at 12,500 rpm for 10 min at 4 °C and the supernatant discarded. The tube was spun briefly to collect residual ethanol to the bottom of tube, and as much liquid as possible was removed with gel-loading tip. Pellet was air-dried at RT until it looked dry (about 5 min) and then resuspended in 20 µl H_2_0. DNA concentration was measured in Qubit (Life Technologies) with brDNA kit (Molecular Probes Q32853).

### MeDIP-Seq analysis

The MeDIP pools were used to create libraries for next-generation sequencing (NGS) using the NEBNext^®^ Ultra™ RNA Library Prep Kit for Illumina^®^ (NEB, San Diego, CA) starting at step 1.4 of the manufacturer’s protocol to generate double-stranded DNA. After this step, the manufacturer’s protocol was followed. Each pool received a separate index primer. NGS was performed at WSU Spokane Genomics Core using the Illumina HiSeq 2500 with a PE50 application, with a read size of approximately 50 bp and approximately 100 million reads per pool. Two to three libraries were run in one lane.

### Histone ChIP-Seq analysis

The ChIP pools were used to create libraries for next-generation sequencing (NGS) using the NEBNext^®^ Ultra™ II DNA Library Prep Kit for Illumina^®^ (NEB, San Diego, CA). The manufacturer protocol was followed. Each pool received a separate index primer. NGS was performed at WSU Spokane Genomics Core using Illumina HiSeq 2500 with a PE50 application, with a read size of approximately 50 bp and approximately 35 million reads per pool. Six libraries were run in one lane.

### Statistics and bioinformatics

The basic read quality was verified using summaries produced by the FastQC program http://www.bioinformatics.babraham.ac.uk/projects/fastqc/. The raw reads were trimmed and filtered using Trimmomatic [62]. The reads for each MeDIP and ChIP sample were mapped to the Rnor 6.0 rat genome using Bowtie2 [63] with default parameter options. The mapped read files were then converted to sorted BAM files using SAMtools [64]. To identify DMRs and DHRs, the reference genome was broken into 100-bp windows. Genomic windows with less than 40 mapped reads summed across all samples were removed prior to further analysis. The MEDIPS R package [65] was then used to calculate differential coverage between control and exposure sample groups. The edgeR *p* value [66] was used to determine the relative difference between the two groups for each genomic window. Windows with an edgeR *p* value less than 10^−6^ were considered DMRs or DHRs. The DMR/DHR edges were extended until no genomic window with a *p* value less than 0.1 remained within 1000 bp of the DMR/DHR. CpG density and other information were then calculated for the DMR/DHR based on the reference genome.

DMRs and DHRs were annotated using the biomaRt R package [67] to access the Ensembl database [68]. The genes that overlapped with DMR or DHR (within 10 kb) were then input into the KEGG pathway search [69, 70] to identify associated pathways. The DMR- and DHR-associated genes were then sorted into functional groups by consulting information provided by the DAVID [71], PANTHER [72] and Uniprot databases incorporated into an internal curated database (www.skinner.wsu.edu under genomic data). All molecular data have been deposited into the public database at NCBI (GEO # GSE109775 and GSE106125, NCBI SRA accession numbers: PRJNA430483 largeRNA (control and DTT), PRJNA430740 smallRNA (control and DTT)). The specific scripts used to perform the analysis can be accessed at github.com/skinnerlab and at www.skinner.wsu.edu/genomic-data-and-r-code-files.

### ncRNA bioinformatics

The small ncRNA data were annotated as follows: Low-quality reads and reads shorter than 15nt were discarded by Cutadapt [73]. The remaining reads were matched to known rat sncRNA, consisting of mature miRNA (miRBase, release 21), precursor miRNA (miRBase, release 21), tRNA (Genomic tRNA Database, rn5), piRNA (piRBase), rRNA (Ensembl, release 76) and mitochondrial RNA (Ensembl, release 76) using AASRA pipeline with default parameters [74]. Read counts generated by AASRA were statistically normalized by DESeq2 [75].

The long ncRNA data were annotated as follows: The FASTX-Toolkit was used to remove adaptor sequences and the low-quality reads from the RNA sequencing data of the mRNA libraries [62]. To identify all the transcripts, we used Tophat2 and Cufflinks to assemble the sequencing reads based on the Ensembl_Rnor_6.0 [76]. The differential expression analyses were performed by Cuffdiff. The coding and the non-coding genes were primarily annotated through rat CDS data ensembl_Rnor_6.0. The non-annotated genes were extracted through our in-house script and then analyzed by CPAT, indicating the true non-coding RNAs [77].

## Additional files

**Additional file 1: Fig. S1.** Principal component analysis (PCA) of control versus DDT genomic data sets for **(A)** DMRs and **(B)** DHRs in the F3 generation sperm. Separate clustering of the control and DDT data includes negligible overlap.

**Additional file 2: Fig. S2.** DMR permutation analysis. **(A)** F1 generation control versus DDT lineage, **(B)** F2 generation control versus DDT lineage, **(C)** F3 generation control versus DDT lineage analysis. The number of DMRs for all comparisons in the permutation analyses. The vertical red line shows the number of DMRs found in the original full analysis. All DMRs are defined using an edgeR p value threshold of 1e−06. The number of DMRs in the control versus DDT lineage analysis is higher than would be expected due to random chance (*p* ≤ 0.1).

**Additional file 3: Table S1.** F1 DMR *p* < 1e−06.

**Additional file 4: Table S2.** F2 DMR *p* < 1e−06.

**Additional file 5: Table S3.** F3 DMR *p* < 1e−06.

**Additional file 6: Table S4.** **(A)** Site Table F1 lncRNA p<1e-04, **(B)** F1 sncRNA *p* < 1e−04.

**Additional file 7: Table S5.** **(A)** F2 lncRNA p<1e-04, **(B)** F2 sncRNA *p* < 1e−04.

**Additional file 8: Table S6.** **(A)** F3 lncRNA p<1e-04, **(B)** F3 sncRNA *p* < 1e−04.

**Additional file 9: Table S7.** F3 DHR *p* < 1e−06.

**Additional file 10: Table S8.** F3 H3K27me3 DHR *p* < 1e−06.

**Additional file 11: Fig. S3.** Genome Browser representative map of site with two DMRs, one ncRNA and one histone retention site on chromosome 22q. One exposed sequence tag (EST) is present.

**Additional file 12: Fig. S4.** Epimutation-associated gene pathway for the pathways in cancer containing 5 DMRs (blue box), 6 ncRNA (red box) and 7 DHRs (pink box).

**Additional file 13: Fig. S5.** Epimutation-associated gene pathways for endocytosis containing 5 DMRs (blue box), 5 ncRNA (red box) and 6 DHRs (pink box).

**Additional file 14: Fig. S6.** Evaluation of the quality of the RNA-Seq data generated in this study. **(A)** Variations among biological replicates of the six RNA samples (pachytene RNP, pachytene polysome, round spermatid RNP, round spermatid polysome, elongating RNP and elongating polysome). The biological variation is reflected by the coefficient of variation to the power of two (CV^2^) of FPKM values for each gene. The CV^2^ represents a normalized measure of cross-replicate variability, which has been widely used for evaluating quality of RNA-Seq data. The data presented here show that the abundance of the genes varied between replicate RNA samples, especially for the ones with lower FPKM values, which is expected. **(B)** Scatterplot matrix showing the pairwise scatterplots of the log_10_-normalized FPKM scores across biological replicates of all six RNA samples. **(C)** Density plots showing the distribution of log_10_-normalized FPKM scores across biological replicates of all six RNA samples. **(D)** Overdispersion plots demonstrating the estimated overdispersion for each sample as a quality control measure.

## Abbreviations

BPA: bisphenol A
DDT: dichlorodiphenyltrichloroethane
DEET: *N*,*N*-diethyl-meta-toluamide
DHRs: differential histone retention regions
DMRs: differential DNA methylation regions
DMSO: dimethyl sulfoxide
FDR: false discovery rate
MeDIP: methylated DNA immunoprecipitation
ncRNA: non-coding RNA
P120: postnatal day 120
PGCs: primordial germ cells
Seq: DNA sequencing
TUNEL: Transferase-mediated dUTP nick-end labeling
