# Supplementary material for: Alterations in sperm DNA methylation, non-coding RNA and histone retention associate with DDT-induced epigenetic transgenerational inheritance of disease
Source: Epigenetics Chromatin. 2018 Feb 27;11:8. doi: 10.1186/s13072-018-0178-0 (PMC5827984; doi:10.1186/s13072-018-0178-0)

Supplemental Figure S1

(A) Principle Component Analysis (PCA) of F3 generation DMRs ($p<10^{-6}$) for control versus DDT lineage sperm.

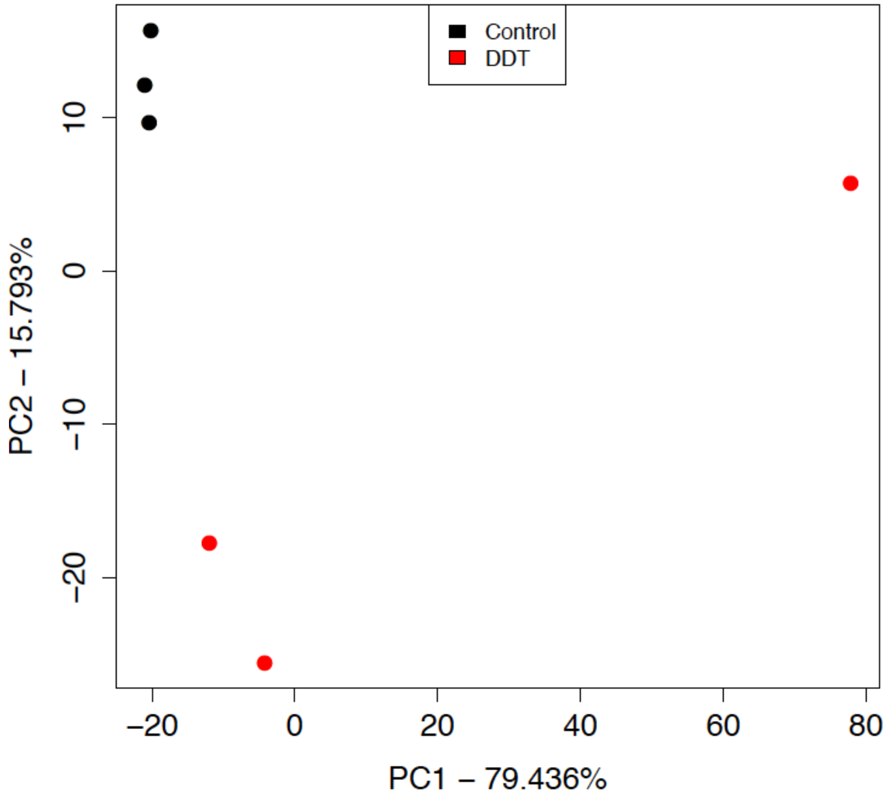

(B) Principle Component Analysis (PCA) of F3 generation DHRs ($p<10^{-6}$) for control versus DDT lineage sperm.

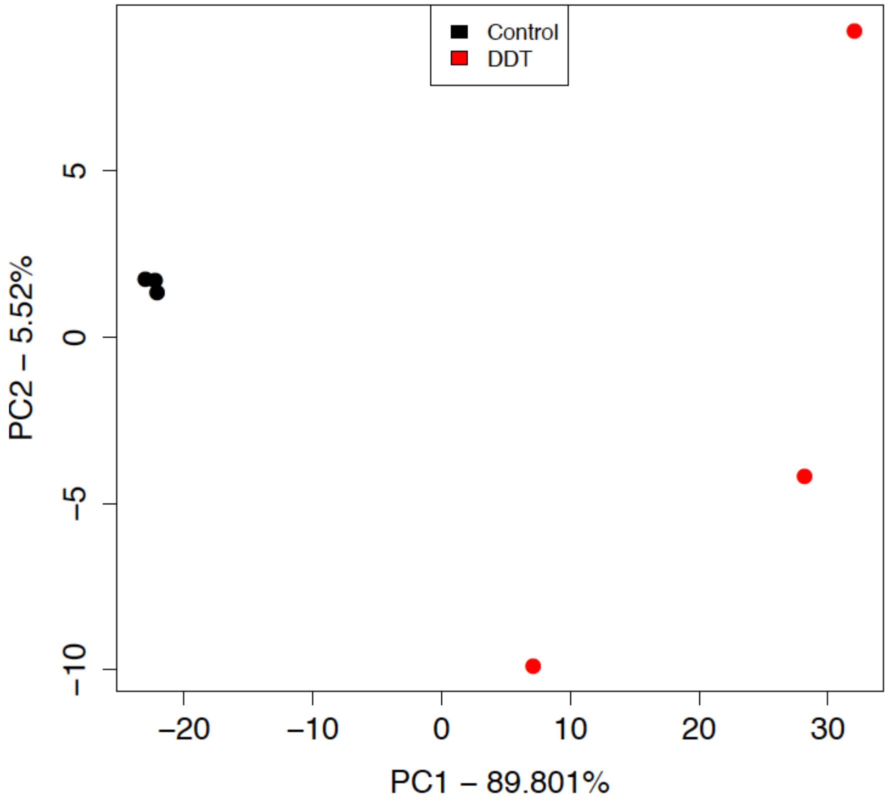

Supplement: Supplementary file 1 — Additional file 1: Fig. S1. Principal component analysis (PCA) of control versus DDT genomic data sets for (A) DMRs and (B) DHRs in the F3 generation sperm. Separate clustering of the control and DDT data includes negligible overlap. [file 13072_2018_178_MOESM1_ESM.pdf]
