# Supplementary material for: Alterations in sperm DNA methylation, non-coding RNA and histone retention associate with DDT-induced epigenetic transgenerational inheritance of disease
Source: Epigenetics Chromatin. 2018 Feb 27;11:8. doi: 10.1186/s13072-018-0178-0 (PMC5827984; doi:10.1186/s13072-018-0178-0)

Supplemental Figure S2

A DDT F1 Generation Lineage Permutation Analyses

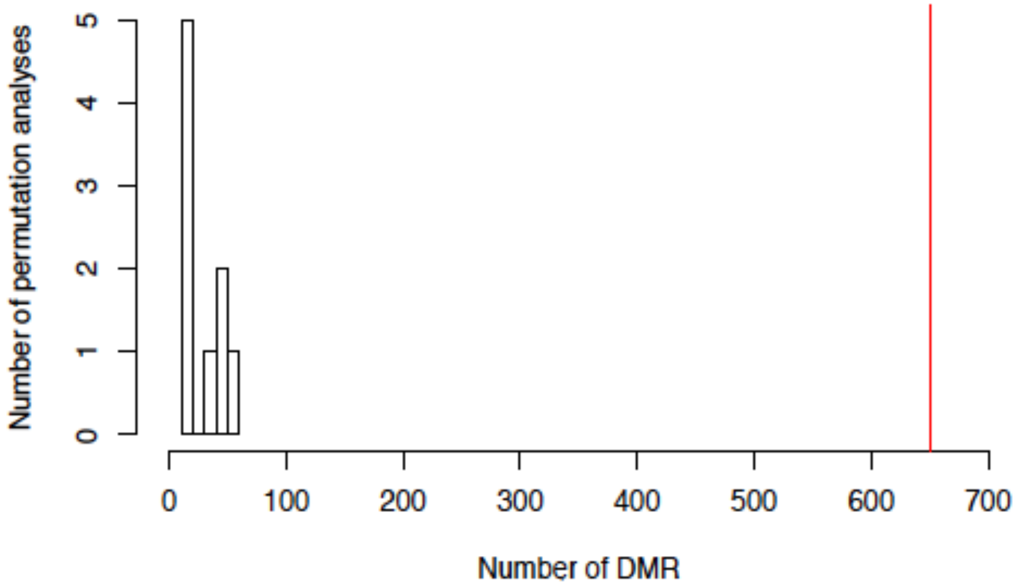

B DDT F2 Generation Lineage Permutation Analyses

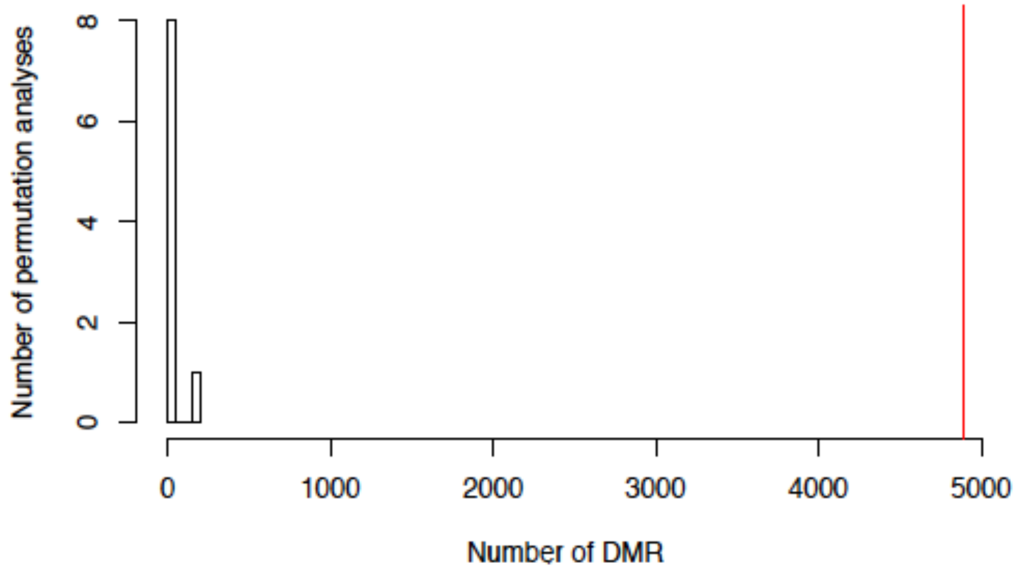

C DDT F3 Generation Lineage Permutation Analyses

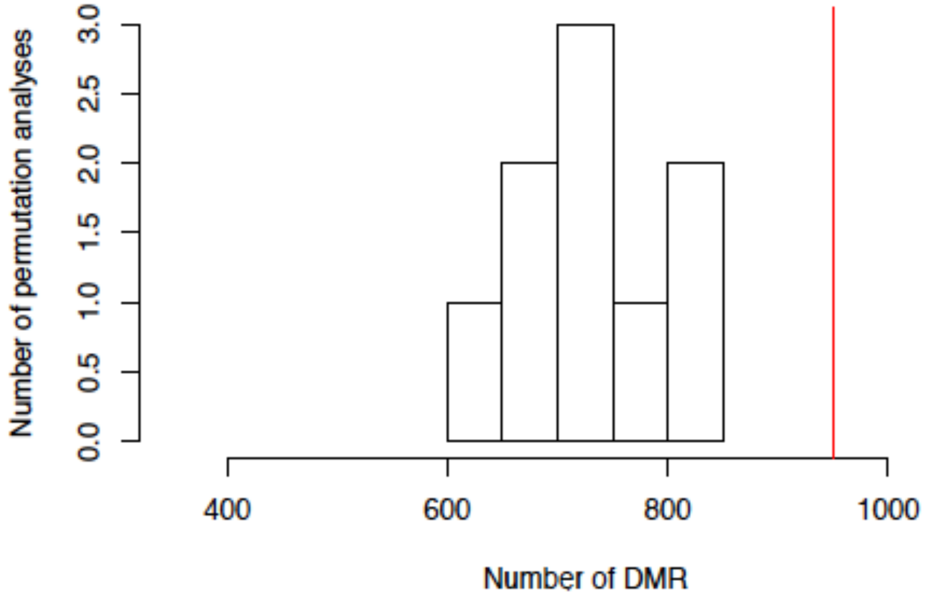

Supplement: Supplementary file 2 — Additional file 2: Fig. S2. DMR permutation analysis. (A) F1 generation control versus DDT lineage, (B) F2 generation control versus DDT lineage, (C) F3 generation control versus DDT lineage analysis. The number of DMRs for all comparisons in the permutation analyses. The vertical red line shows the number of DMRs found in the original full analysis. All DMRs are defined using an edgeR p value threshold of 1e−06. The number of DMRs in the control versus DDT lineage analysis is higher than would be expected due to random chance (p ≤ 0.1). [file 13072_2018_178_MOESM2_ESM.pdf]
