# Supplementary material for: Alterations in sperm DNA methylation, non-coding RNA and histone retention associate with DDT-induced epigenetic transgenerational inheritance of disease
Source: Epigenetics Chromatin. 2018 Feb 27;11:8. doi: 10.1186/s13072-018-0178-0 (PMC5827984; doi:10.1186/s13072-018-0178-0)
Supplement: Supplementary file 3 — Additional file 3: Table S1. F1 DMR p < 1e−06. [file 13072_2018_178_MOESM3_ESM.pdf]

Supplemental Table S1

F1 DMR p<1e-06									
DMR Name	Chr	Start	(bp) Length	# Sig Win	minP	CpG #	CpG Density	Gene Association	Gene Category
DMR1:2122601	1	2122601	600	1	1.31E-07	19	3.16		
DMR1:5431001	1	5431001	800	1	4.83E-08	10	1.25	SNORA70	
DMR1:13461701	1	13461701	1100	1	9.69E-07	14	1.27		
DMR1:17119101	1	17119101	1800	1	3.12E-07	21	1.16		
DMR1:26288501	1	26288501	2800	1	1.25E-07	82	2.92		
DMR1:29733301	1	29733301	500	1	2.46E-07	5	1		
DMR1:33501601	1	33501601	4200	1	5.62E-07	47	1.11		
DMR1:34949601	1	34949601	1800	1	4.67E-07	14	0.77		
DMR1:35252601	1	35252601	600	1	3.69E-07	0	0		
DMR1:35672001	1	35672001	300	1	3.36E-07	3	1		
DMR1:38083901	1	38083901	2300	1	1.78E-07	27	1.17		
DMR1:38399901	1	38399901	3400	1	2.10E-07	55	1.61		
DMR1:41983501	1	41983501	1500	1	9.64E-07	7	0.46	Syne1	Development
DMR1:46552001	1	46552001	2700	1	6.79E-07	42	1.55	Zdhhc14	Unknown
DMR1:48531001	1	48531001	800	1	3.66E-07	8	1	Slc22a3;Plg	Transport;Protease
DMR1:49896901	1	49896901	500	1	1.67E-07	9	1.8		
DMR1:52729801	1	52729801	700	1	1.05E-07	5	0.71		
DMR1:54393701	1	54393701	300	1	1.67E-07	19	6.33		
DMR1:54401901	1	54401901	700	1	8.17E-07	5	0.71		
DMR1:54440501	1	54440501	2300	1	7.77E-08	67	2.91	AABR07001634.1	
DMR1:57329801	1	57329801	1000	1	8.97E-07	20	2	Dll1	Receptor
DMR1:58123201	1	58123201	700	1	2.15E-07	4	0.57		
DMR1:59445801	1	59445801	500	1	4.94E-07	0	0		
DMR1:70857601	1	70857601	2300	1	1.25E-08	56	2.43		
DMR1:77158001	1	77158001	400	2	6.48E-08	3	0.75		
DMR1:79353601	1	79353601	600	2	4.34E-10	1	0.16	LOC102557244	
DMR1:79849201	1	79849201	1500	1	3.62E-08	15	1	Nova2	Transcription
DMR1:95985701	1	95985701	1800	1	4.91E-07	24	1.33		
DMR1:96048501	1	96048501	1000	1	7.69E-07	52	5.2		
DMR1:96102101	1	96102101	2000	1	6.04E-07	57	2.85		
DMR1:96435501	1	96435501	300	1	1.39E-07	3	1		
DMR1:100341801	1	100341801	800	1	2.56E-07	5	0.62	Shank1;RGD1309036	Development
DMR1:101164201	1	101164201	900	1	6.04E-07	15	1.66	Pih1d1;Slc17a7;Gfy	Signaling;Transport
DMR1:105461701	1	105461701	2000	2	7.21E-07	22	1.1	Nell1	Development
DMR1:123429101	1	123429101	400	1	6.42E-07	0	0		
DMR1:125447501	1	125447501	4000	1	2.72E-07	57	1.42	Apba2	Transport
DMR1:130135601	1	130135601	400	1	6.60E-07	3	0.75		
DMR1:143666601	1	143666601	1200	1	7.06E-07	20	1.66	7SK;Tm6sf1	
DMR1:166337301	1	166337301	1300	1	4.57E-07	9	0.69	Fchsd2	Cell Cycle
DMR1:167944501	1	167944501	500	1	2.17E-07	6	1.2	Olr57;Olr56	Receptor
DMR1:170546001	1	170546001	1500	1	3.25E-07	5	0.33	Dnhd1	
DMR1:170628201	1	170628201	500	1	7.65E-07	13	2.6	Dchs1	Signaling
DMR1:179108501	1	179108501	400	1	7.54E-08	6	1.5		
DMR1:179344001	1	179344001	2600	3	4.51E-10	75	2.88		
DMR1:179349701	1	179349701	1100	3	6.51E-08	35	3.18		
DMR1:179659501	1	179659501	2200	3	3.76E-08	40	1.81		
DMR1:179687901	1	179687901	3900	1	1.88E-07	46	1.17		
DMR1:180089501	1	180089501	1000	1	1.15E-07	24	2.4		
DMR1:181866901	1	181866901	1900	1	3.12E-07	48	2.52		
DMR1:182391601	1	182391601	2500	1	3.00E-07	19	0.76		

DMR1:182622101	1	182622101	3000	1	9.73E-07	60	2		
DMR1:189348901	1	189348901	1200	1	3.45E-07	11	0.91	Acsm1;RNaseP_nuc;RGD1559600	Metabolism
DMR1:193492901	1	193492901	1900	2	1.96E-09	26	1.36		
DMR1:194066201	1	194066201	300	1	2.17E-07	0	0		
DMR1:194283401	1	194283401	400	1	6.55E-07	4	1		
DMR1:198491601	1	198491601	1300	1	6.60E-09	26	2	Zg16	
DMR1:201100401	1	201100401	1000	1	9.73E-07	25	2.5	Tacc2;Nsmce4a	Unknown
DMR1:202699101	1	202699101	800	1	1.17E-09	1	0.12	AABR07005837.1;AABR07005837.2	
DMR1:210028101	1	210028101	500	1	6.64E-07	0	0		
DMR1:215835901	1	215835901	400	1	7.47E-07	28	7	Igf2;AC098563.1;Mir483	Growth Factors & Cytokines
DMR1:218366801	1	218366801	2000	1	5.19E-08	15	0.75	Rmt1	
DMR1:220694901	1	220694901	2900	1	1.20E-07	71	2.44		
DMR1:221778301	1	221778301	1000	1	8.83E-07	6	0.6	Pygm;Rasgrp2	Signaling
DMR1:225063801	1	225063801	300	1	4.47E-07	10	3.33	Ubxn1	
DMR1:235685201	1	235685201	200	1	2.37E-09	3	1.5	Vps13a	Proteolysis
DMR1:238252201	1	238252201	800	1	3.63E-07	10	1.25	Aldh1a1	Metabolism
DMR1:245322501	1	245322501	2200	1	8.29E-08	10	0.45		
DMR1:247543701	1	247543701	2300	1	2.37E-07	21	0.91	Cd274	Immune
DMR1:249350201	1	249350201	2300	1	2.05E-07	20	0.86		
DMR1:259696001	1	259696001	3300	1	1.55E-07	37	1.12	Tctn3	Unknown
DMR1:260072701	1	260072701	700	1	4.72E-08	6	0.85	AABR07072054.1	
DMR1:261252801	1	261252801	400	2	3.10E-07	3	0.75	Ubtd1	Metabolism
DMR1:263455101	1	263455101	2200	1	3.70E-07	25	1.13	Entpd7	Signaling
DMR1:272946401	1	272946401	1100	1	8.17E-07	22	2		
DMR1:273883901	1	273883901	400	1	1.61E-07	5	1.25	Add3	Cytoskeleton
DMR1:275677101	1	275677101	300	1	6.88E-07	8	2.66	AABR07006993.1	
DMR1:277917801	1	277917801	1700	2	6.28E-08	50	2.94	Ablim1	Cytoskeleton
DMR1:277931201	1	277931201	3500	2	3.11E-08	74	2.11	Ablim1	Cytoskeleton
DMR1:278447601	1	278447601	3400	1	3.43E-08	46	1.35		
DMR1:279839901	1	279839901	2500	1	1.87E-07	21	0.84		
DMR1:280488701	1	280488701	1500	1	6.67E-08	8	0.53	Pdzd8	Immune
DMR1:282130101	1	282130101	2000	1	4.35E-07	34	1.7	Nanos1;Eif3a	Transcription
DMR2:19766301	2	19766301	1200	1	3.33E-07	4	0.33		
DMR2:19809601	2	19809601	2200	1	6.88E-07	26	1.18	Atp6ap1l	Metabolism
DMR2:23388601	2	23388601	300	1	7.85E-07	3	1	Arsb	Metabolism
DMR2:25569301	2	25569301	3200	1	4.38E-07	49	1.53	AABR07007717.1	
DMR2:47204101	2	47204101	300	1	6.93E-07	0	0	Itga1	Signaling
DMR2:48424601	2	48424601	700	1	8.31E-07	6	0.85		
DMR2:57919701	2	57919701	400	1	8.67E-07	2	0.5	Slc1a3	Metabolism
DMR2:62798801	2	62798801	800	1	7.54E-07	3	0.37		
DMR2:73760001	2	73760001	500	1	6.16E-07	5	1		
DMR2:80861601	2	80861601	1900	1	1.23E-07	21	1.1	AABR07008948.1	
DMR2:80909901	2	80909901	400	1	2.40E-07	1	0.25		
DMR2:95212901	2	95212901	400	3	5.83E-09	17	4.25		
DMR2:111505501	2	111505501	600	1	4.05E-07	2	0.33	Nlgn1	Signaling
DMR2:114565501	2	114565501	200	1	2.32E-10	3	1.5		
DMR2:114591801	2	114591801	500	1	1.75E-07	17	3.4		
DMR2:114934301	2	114934301	2100	3	1.05E-09	33	1.57		
DMR2:115810201	2	115810201	500	5	2.09E-20	0	0	U1	
DMR2:135271101	2	135271101	300	1	6.64E-07	1	0.33		
DMR2:137919301	2	137919301	500	1	6.49E-08	3	0.6		
DMR2:140732801	2	140732801	900	1	4.44E-07	24	2.66	Mgst2;AABR07010563.1	Metabolism
DMR2:146453901	2	146453901	500	1	5.63E-07	0	0		

DMR2:147922601	2	147922601	300	1	3.74E-07	1	0.33	Rnf13	
DMR2:157942701	2	157942701	600	1	3.05E-07	3	0.5	Veph1	Unknown
DMR2:161772001	2	161772001	2400	1	5.30E-07	22	0.91		
DMR2:161942801	2	161942801	1100	2	8.22E-08	60	5.45		
DMR2:162805801	2	162805801	400	1	4.53E-07	3	0.75		
DMR2:165218201	2	165218201	500	1	1.35E-07	18	3.6		
DMR2:176203901	2	176203901	300	1	1.28E-08	1	0.33		
DMR2:177956801	2	177956801	900	1	1.52E-07	9	1	Rapgef2	
DMR2:183585401	2	183585401	1800	1	2.91E-07	17	0.94	Arfip1	Signaling
DMR2:183615501	2	183615501	600	1	6.93E-07	11	1.83		
DMR2:185327701	2	185327701	1300	1	8.73E-08	12	0.92	Sh3d19	
DMR2:194295201	2	194295201	1100	1	9.72E-08	51	4.63		
DMR2:196937501	2	196937501	300	2	6.46E-09	4	1.33		
DMR2:196940201	2	196940201	400	2	5.24E-08	6	1.5		
DMR2:206202501	2	206202501	1100	1	4.10E-07	52	4.72		
DMR2:214273101	2	214273101	300	1	2.26E-08	5	1.66		
DMR2:221154901	2	221154901	700	1	1.05E-07	0	0		
DMR2:225050301	2	225050301	800	1	6.46E-07	9	1.12	Slc44a3;AABR07013154.1	Metabolism
DMR2:225337601	2	225337601	400	1	2.11E-07	5	1.25	Abcd3	Transport
DMR2:225949201	2	225949201	1100	1	2.75E-07	9	0.81		
DMR2:244095901	2	244095901	2300	2	4.02E-08	49	2.13	Tspan5	Cytoskeleton
DMR2:244115301	2	244115301	7700	1	3.02E-07	155	2.01	Tspan5	Cytoskeleton
DMR2:244591501	2	244591501	500	2	6.66E-14	6	1.2	Stpg2	Development
DMR2:244910301	2	244910301	500	1	4.44E-07	1	0.2	Stpg2	Development
DMR2:245138101	2	245138101	1000	1	1.78E-07	9	0.9	AABR07013586.1	
DMR2:247454801	2	247454801	400	1	7.03E-07	5	1.25	Bmpr1b	Receptor
DMR2:251086601	2	251086601	1400	1	3.03E-07	27	1.92		
DMR2:254329301	2	254329301	2600	1	7.23E-07	41	1.57	Rn60_2_2544.1	
DMR2:257969601	2	257969601	700	1	2.24E-08	12	1.71	Pigk	Metabolism
DMR2:259022101	2	259022101	2700	1	1.26E-07	41	1.51		
DMR2:259146701	2	259146701	700	2	9.63E-11	2	0.28	AABR07013843.1	
DMR2:259591801	2	259591801	3300	2	7.57E-09	80	2.42	St6galnac3	Metabolism
DMR2:259625001	2	259625001	900	2	1.42E-09	2	0.22	St6galnac3	Metabolism
DMR3:2418401	3	2418401	1300	1	7.17E-08	8	0.61	Tor4a;Nelfb;U6	
DMR3:10321701	3	10321701	4000	1	1.78E-07	52	1.3	Ass1	Development
DMR3:32728501	3	32728501	200	1	1.60E-07	0	0		
DMR3:33626201	3	33626201	3500	1	5.48E-07	46	1.31		
DMR3:34102901	3	34102901	1300	1	6.46E-09	13	1		
DMR3:36579401	3	36579401	2100	2	3.34E-08	22	1.04	AABR07052091.1	
DMR3:37914701	3	37914701	400	1	2.28E-07	2	0.5	Arl5a	Translation
DMR3:43611201	3	43611201	1900	1	3.67E-09	25	1.31		
DMR3:49554201	3	49554201	300	3	6.36E-14	4	1.33		
DMR3:50572001	3	50572001	1200	1	4.19E-07	9	0.75		
DMR3:59578901	3	59578901	400	1	3.29E-07	5	1.25	AC120066.1	
DMR3:60962101	3	60962101	2100	4	2.09E-08	22	1.04		
DMR3:61170101	3	61170101	400	1	4.92E-07	0	0		
DMR3:63575601	3	63575601	3200	1	2.87E-07	98	3.06	AABR07052585.2	
DMR3:65649301	3	65649301	300	2	1.11E-07	2	0.66		
DMR3:67671201	3	67671201	1400	1	3.41E-07	19	1.35	Frzb;AABR07052643.1	Receptor
DMR3:72583701	3	72583701	1500	1	5.22E-07	19	1.26	Lrrc55	Unknown
DMR3:82683401	3	82683401	400	1	4.92E-07	2	0.5	Ext2	Metabolism
DMR3:83322801	3	83322801	300	1	6.68E-07	5	1.66	Api5	Apoptosis
DMR3:86806501	3	86806501	200	1	1.68E-07	1	0.5	Lrrc4c	Extracellular Matrix
DMR3:94326501	3	94326501	200	1	4.04E-07	1	0.5		

DMR3:95748801	3	95748801	1200	1	3.09E-07	7	0.58		
DMR3:96280601	3	96280601	4600	4	6.45E-08	60	1.3		
DMR3:104306001	3	104306001	600	1	6.74E-07	13	2.16	Ryr3	Receptor
DMR3:109212201	3	109212201	1900	1	2.98E-08	13	0.68		
DMR3:110793101	3	110793101	2800	1	4.71E-07	33	1.17		
DMR3:117680801	3	117680801	400	1	9.75E-07	3	0.75	Fbn1	Development
DMR3:123230901	3	123230901	600	1	8.44E-07	4	0.66	Itpa;Slc4a11	Signaling;Transport
DMR3:133520401	3	133520401	2100	1	4.12E-07	56	2.66		
DMR3:140691301	3	140691301	1000	1	8.77E-07	6	0.6	Ralgapa2	Signaling
DMR3:150379501	3	150379501	400	1	6.28E-08	3	0.75	Raly	Transcription
DMR3:150829401	3	150829401	2200	1	5.57E-07	17	0.77	Pigu;U6	Unknown
DMR3:153002701	3	153002701	1100	1	3.19E-07	18	1.63	Sla2;Ndrg3	Signaling;Transcription
DMR3:153044501	3	153044501	700	1	3.71E-07	5	0.71	Ndrg3	Transcription
DMR3:155197801	3	155197801	2300	1	4.12E-07	43	1.86	Ppp1r16b;RGD1563145	Signaling
DMR3:160629801	3	160629801	300	1	2.52E-07	6	2	Semg1	
DMR3:162938001	3	162938001	300	1	6.68E-07	3	1		
DMR3:164799401	3	164799401	1300	1	1.73E-07	22	1.69		
DMR3:165274701	3	165274701	1900	1	6.38E-07	26	1.36	Nfatc2	Transcription
DMR3:165794801	3	165794801	200	1	7.57E-09	7	3.5		
DMR3:165809201	3	165809201	200	1	1.70E-07	1	0.5		
DMR3:166178501	3	166178501	400	1	9.35E-07	12	3	U2	
DMR3:166220501	3	166220501	1000	2	2.12E-07	2	0.2		
DMR3:166480901	3	166480901	1400	1	1.09E-07	18	1.28		
DMR3:169328701	3	169328701	1200	1	5.24E-09	23	1.91		
DMR3:169428901	3	169428901	7500	1	5.04E-07	67	0.89		
DMR4:5486801	4	5486801	1400	1	1.58E-09	18	1.28		
DMR4:21657401	4	21657401	300	1	7.88E-07	0	0	RGD1563349	EST
DMR4:28043101	4	28043101	700	1	8.29E-07	5	0.71		
DMR4:29086401	4	29086401	500	1	2.84E-07	0	0	Bet1	Golgi
DMR4:29447201	4	29447201	2700	1	4.99E-07	24	0.88		
DMR4:32729601	4	32729601	1300	2	6.05E-08	39	3		
DMR4:32749201	4	32749201	3500	1	3.05E-07	51	1.45		
DMR4:37577401	4	37577401	100	1	1.70E-08	0	0		
DMR4:39235301	4	39235301	400	1	5.74E-07	1	0.25		
DMR4:46283301	4	46283301	300	1	6.42E-07	1	0.33		
DMR4:48519501	4	48519501	1500	1	4.31E-07	10	0.66		
DMR4:49087501	4	49087501	3200	1	1.58E-11	39	1.21	Cped1	
DMR4:64780401	4	64780401	4900	1	3.50E-07	72	1.46	Dgki	Signaling
DMR4:78516501	4	78516501	300	1	7.41E-07	1	0.33	Aoc1	Metabolism
DMR4:78770201	4	78770201	800	1	6.78E-07	22	2.75	Igf2bp3	Transcription
DMR4:80337001	4	80337001	1800	1	1.06E-07	12	0.66	LOC100363502;LOC500124	Binding Protein;EST
DMR4:83936201	4	83936201	1200	1	5.00E-08	21	1.75		
DMR4:85445001	4	85445001	400	1	2.01E-07	1	0.25	Fam188b	
DMR4:94785701	4	94785701	1200	1	1.63E-07	14	1.16	Grid2	Signaling
DMR4:100975601	4	100975601	1000	1	8.71E-07	19	1.9	Dnah6	
DMR4:116003001	4	116003001	1500	1	1.69E-07	8	0.53		
DMR4:116206401	4	116206401	1900	1	3.17E-07	12	0.63		
DMR4:120173701	4	120173701	1900	1	8.15E-07	22	1.15	SNORA29	
DMR4:124943701	4	124943701	400	1	3.85E-07	1	0.25		
DMR4:126766801	4	126766801	300	1	2.37E-07	6	2		
DMR4:130855401	4	130855401	1100	1	3.89E-07	12	1.09		
DMR4:132263601	4	132263601	500	1	7.80E-07	6	1.2		
DMR4:140305301	4	140305301	400	1	6.78E-08	0	0	Itpr1	Signaling

DMR4:146541001	4	146541001	2900	1	9.59E-07	92	3.17		
DMR4:157390201	4	157390201	900	1	1.91E-08	13	1.44	Gpr162;Cd4	Immune
DMR4:158770401	4	158770401	400	1	2.54E-07	2	0.5	AABR07062046.1	
DMR4:159432401	4	159432401	200	1	1.68E-07	1	0.5	Akap3;Dyrk4	Signaling
DMR4:171634101	4	171634101	400	1	4.58E-07	1	0.25		
DMR4:171654101	4	171654101	500	1	3.14E-07	1	0.2		
DMR4:177744501	4	177744501	500	1	3.09E-08	21	4.2		
DMR4:177863401	4	177863401	2200	1	5.66E-08	42	1.9		
DMR4:178444501	4	178444501	700	1	6.09E-08	10	1.42	Sox5	Transcription
DMR4:179152301	4	179152301	1200	1	4.62E-07	11	0.91		
DMR4:180274901	4	180274901	300	1	9.73E-07	6	2		
DMR4:180578701	4	180578701	200	1	2.76E-08	8	4	Itpr2	Receptor
DMR5:2822201	5	2822201	600	1	3.30E-07	5	0.83	Sbspon;7SK	
DMR5:4790901	5	4790901	1900	1	6.00E-07	27	1.42	Rbpj	Transcription
DMR5:6189201	5	6189201	1200	1	8.98E-07	8	0.66	Sulf1	Metabolism
DMR5:6372701	5	6372701	2000	3	5.92E-08	33	1.65	AABR07046778.1	
DMR5:16714201	5	16714201	1700	1	3.80E-09	11	0.64	Rps20;snoU54	Translation
DMR5:39710401	5	39710401	800	1	4.34E-07	5	0.62		
DMR5:57389501	5	57389501	2400	1	9.26E-07	25	1.04		
DMR5:58957901	5	58957901	3300	2	1.85E-09	43	1.3	Cd72;AC121204.1	
DMR5:62314501	5	62314501	1100	1	1.03E-08	13	1.18	Gabbr2	Receptor
DMR5:63459301	5	63459301	1100	1	1.44E-08	8	0.72		
DMR5:63771701	5	63771701	1700	2	1.89E-07	14	0.82	Nr4a3	Transcription
DMR5:67687601	5	67687601	300	1	4.04E-07	3	1		
DMR5:87513701	5	87513701	1400	1	2.75E-07	8	0.57		
DMR5:95682501	5	95682501	300	1	1.16E-07	0	0		
DMR5:97160301	5	97160301	300	1	1.47E-07	2	0.66		
DMR5:100387701	5	100387701	2600	1	2.48E-07	21	0.8		
DMR5:100576001	5	100576001	400	1	9.22E-07	11	2.75	Nfib	Transcription
DMR5:103111001	5	103111001	300	1	2.10E-07	0	0		
DMR5:106158301	5	106158301	1800	1	5.41E-09	31	1.72	Mllt3	Transcription
DMR5:107803601	5	107803601	800	1	1.61E-07	20	2.5		
DMR5:114867101	5	114867101	2700	1	7.25E-07	26	0.96	Fggy	Signaling
DMR5:132493901	5	132493901	400	1	7.23E-07	3	0.75		
DMR5:136975301	5	136975301	1400	1	3.80E-07	36	2.57	Kdm4a	Epigenetic
DMR5:145708101	5	145708101	500	1	9.92E-07	3	0.6		
DMR5:146007301	5	146007301	1000	1	2.49E-07	19	1.9		
DMR5:149841301	5	149841301	2000	1	1.79E-07	24	1.2		
DMR5:152600401	5	152600401	600	1	6.16E-08	8	1.33	7SK;Pafah2	Metabolism
DMR5:154384501	5	154384501	400	1	1.47E-07	4	1	Rpl11	Translation
DMR5:155506601	5	155506601	300	1	2.17E-07	12	4		
DMR5:160845901	5	160845901	500	1	3.02E-07	2	0.4		
DMR5:160945301	5	160945301	1300	2	5.83E-12	13	1		
DMR5:168054501	5	168054501	700	1	7.27E-07	11	1.57	U1	
DMR5:169598901	5	169598901	600	1	4.64E-07	4	0.66	Kcnab2	Transport
DMR5:171890301	5	171890301	2500	1	5.09E-08	34	1.36		
DMR5:172896001	5	172896001	600	1	3.60E-07	2	0.33	Tmem52;AC130035.2	
DMR6:1576701	6	1576701	600	1	2.52E-07	4	0.66	Prkd3	Signaling
DMR6:3857201	6	3857201	3500	1	9.65E-07	60	1.71		
DMR6:6712701	6	6712701	500	1	2.59E-07	1	0.2	Cox7a2l	Electron Transport
DMR6:9956801	6	9956801	300	1	1.39E-07	2	0.66	Prkce	Binding Protein
DMR6:10511801	6	10511801	2300	1	8.16E-07	40	1.73	Atp6v1e2	Transport
DMR6:15917201	6	15917201	1100	1	7.35E-08	8	0.72		
DMR6:16266201	6	16266201	4200	1	2.16E-08	58	1.38		
DMR6:16600501	6	16600501	1700	1	8.37E-07	32	1.88		
DMR6:17329101	6	17329101	700	3	5.03E-09	32	4.57		

DMR6:20148001	6	20148001	200	1	5.28E-07	1	0.5		
DMR6:31782101	6	31782101	300	1	4.12E-07	0	0		
DMR6:32573401	6	32573401	1700	1	2.08E-08	5	0.29	AABR07063511.2	
DMR6:34817401	6	34817401	900	1	8.56E-07	1	0.11		
DMR6:37884201	6	37884201	700	1	6.93E-07	10	1.42		
DMR6:41028301	6	41028301	1400	1	1.62E-07	15	1.07	Trib2;AABR07063672.1	Signaling
DMR6:49556701	6	49556701	1100	1	9.93E-08	3	0.27		
DMR6:54506001	6	54506001	300	1	6.04E-07	6	2	Snx13	Signaling
DMR6:62286601	6	62286601	800	1	3.82E-07	4	0.5	AABR07064048.2	
DMR6:65901401	6	65901401	500	1	9.73E-07	5	1		
DMR6:99239001	6	99239001	2900	1	7.41E-07	32	1.1	Tex21;Rn50_6_1087.2	
DMR6:102759501	6	102759501	900	1	1.75E-07	7	0.77		
DMR6:103640101	6	103640101	300	1	2.56E-07	9	3	AABR07064983.1	
DMR6:110127801	6	110127801	400	2	6.20E-08	2	0.5	Gpatch2l	
DMR6:121937101	6	121937101	300	1	9.30E-07	1	0.33		
DMR6:122667801	6	122667801	3300	1	2.77E-07	78	2.36	Ptpn21	Signaling
DMR6:130841901	6	130841901	1100	1	1.24E-07	8	0.72		
DMR6:134186001	6	134186001	500	1	6.77E-08	5	1		
DMR7:1404401	7	1404401	100	1	3.19E-07	6	6		
DMR7:2109001	7	2109001	6500	1	9.27E-07	127	1.95		
DMR7:2656901	7	2656901	3100	3	7.51E-14	97	3.12	Timeless;U6	Development
DMR7:8168601	7	8168601	300	1	5.41E-07	2	0.66		
DMR7:11762901	7	11762901	300	3	1.83E-09	6	2	Oaz1;Jsrp1	Signaling
DMR7:14447001	7	14447001	500	1	3.16E-07	20	4	Cyp4f39	Metabolism
DMR7:21449901	7	21449901	400	2	1.72E-09	10	2.5		
DMR7:21495001	7	21495001	8900	2	8.72E-09	95	1.06		
DMR7:21728201	7	21728201	4100	1	4.31E-07	26	0.63		
DMR7:21733501	7	21733501	2500	2	2.72E-08	14	0.56		
DMR7:21790801	7	21790801	600	1	4.67E-07	6	1		
DMR7:22025001	7	22025001	3600	1	8.17E-07	26	0.72		
DMR7:29387501	7	29387501	1200	1	8.61E-07	18	1.5	Utp20	
DMR7:31221601	7	31221601	1000	1	2.30E-07	16	1.6	Anks1b	Receptor
DMR7:33618401	7	33618401	600	1	6.36E-07	5	0.83		
DMR7:38602901	7	38602901	1100	1	4.34E-07	14	1.27		
DMR7:61554001	7	61554001	1300	1	1.09E-07	13	1		
DMR7:70267101	7	70267101	1300	1	1.29E-07	16	1.23	AABR07057421.2;AABR07057421.3	
DMR7:70866201	7	70866201	2400	1	2.57E-08	57	2.37	Lrp1	Receptor
DMR7:79362401	7	79362401	2300	1	2.12E-08	15	0.65		
DMR7:81412501	7	81412501	400	1	1.32E-07	1	0.25	Angpt1	Growth Factors & Cytokines
DMR7:81914201	7	81914201	1000	1	4.24E-07	10	1	Rspo2	
DMR7:91065301	7	91065301	400	1	7.28E-07	5	1.25		
DMR7:92216201	7	92216201	400	1	4.42E-07	2	0.5		
DMR7:112225501	7	112225501	500	1	6.85E-07	3	0.6		
DMR7:119308601	7	119308601	1100	1	8.80E-08	14	1.27	Cacng2	Transport
DMR7:119841401	7	119841401	600	1	6.03E-07	6	1	Cyth4	Signaling
DMR7:121525201	7	121525201	1800	1	6.94E-07	18	1	Cacna1i	Transport
DMR7:127161801	7	127161801	300	1	9.69E-07	4	1.33	Tbc1d22a	Signaling
DMR7:134169001	7	134169001	2500	1	6.49E-08	52	2.08		
DMR7:135125701	7	135125701	400	1	6.51E-07	11	2.75	AABR07058724.1	
DMR8:15101	8	15101	3300	1	2.30E-08	79	2.39		
DMR8:181001	8	181001	1300	3	5.78E-09	30	2.3		
DMR8:9474701	8	9474701	300	1	6.98E-08	0	0		
DMR8:10348801	8	10348801	4300	2	1.25E-07	77	1.79		

DMR8:10358101	8	10358101	2200	1	8.86E-08	30	1.36		
DMR8:10551801	8	10551801	2100	1	6.07E-07	46	2.19		
DMR8:19821301	8	19821301	9800	1	9.53E-07	231	2.35	Olr1156;U6	
DMR8:38511501	8	38511501	300	1	9.27E-07	1	0.33		
DMR8:39753001	8	39753001	1300	1	1.61E-07	3	0.23	Ccdc15	
DMR8:40443701	8	40443701	1900	1	6.40E-08	13	0.68	Olr1203	Receptor
DMR8:45009701	8	45009701	600	1	3.87E-07	17	2.83		
DMR8:45954301	8	45954301	300	1	7.91E-07	2	0.66		
DMR8:52243301	8	52243301	4100	14	1.03E-32	25	0.6	AABR07070099.1	
DMR8:52451901	8	52451901	600	1	4.72E-08	5	0.83		
DMR8:61091501	8	61091501	1500	1	2.05E-07	8	0.53	Hmg20a	
DMR8:62782101	8	62782101	1200	1	1.82E-07	9	0.75	Cyp11a1	Metabolism
DMR8:64109701	8	64109701	1000	1	8.44E-07	61	6.1	Adpgk	Metabolism
DMR8:64923501	8	64923501	1700	1	3.11E-07	25	1.47	Rn50_8_0647.2	
DMR8:68661301	8	68661301	1200	1	7.18E-09	28	2.33	Smad3	Transcription
DMR8:76117701	8	76117701	400	1	4.57E-07	0	0		
DMR8:80126001	8	80126001	300	1	4.10E-08	0	0		
DMR8:80830101	8	80830101	3200	1	2.07E-11	27	0.84		
DMR8:85137401	8	85137401	1300	1	1.47E-07	10	0.76		
DMR8:85871501	8	85871501	900	1	7.41E-07	8	0.88		
DMR8:90299301	8	90299301	1000	1	8.40E-07	9	0.9	AABR07070892.1	
DMR8:93401501	8	93401501	1400	1	1.26E-07	6	0.42		
DMR8:95179801	8	95179801	500	1	1.84E-07	3	0.6		
DMR8:103116201	8	103116201	600	1	1.15E-07	2	0.33		
DMR8:105996101	8	105996101	1900	1	4.16E-08	29	1.52		
DMR8:106201501	8	106201501	200	1	3.63E-07	3	1.5		
DMR8:115300301	8	115300301	2400	1	8.95E-08	13	0.54		
DMR8:115920401	8	115920401	1100	1	1.55E-07	24	2.18	Dock3;SNORA17	Signaling
DMR8:117258201	8	117258201	1300	1	9.27E-07	17	1.3	Lamb2;Ccdc71	Cytoskeleton;Transcription
DMR8:122313301	8	122313301	1700	1	1.11E-08	20	1.17	Fbxl2;AABR07071567.2	Proteolysis
DMR8:124770101	8	124770101	800	1	1.91E-07	14	1.75	Rbms3	Epigenetic
DMR8:125911801	8	125911801	1100	2	1.47E-09	6	0.54		
DMR8:127880801	8	127880801	1200	1	4.47E-07	17	1.41	Slc22a14;Slc22a13	Transport
DMR8:128073301	8	128073301	500	1	6.73E-07	3	0.6	Xylb	Metabolism
DMR8:129975301	8	129975301	600	1	4.53E-07	14	2.33		
DMR9:873601	9	873601	3800	1	4.92E-07	117	3.07		
DMR9:10235201	9	10235201	2400	1	2.26E-08	25	1.04	Rfx2	Transcription
DMR9:11750201	9	11750201	4500	1	2.87E-07	140	3.11		
DMR9:11760601	9	11760601	3800	1	3.84E-07	122	3.21		
DMR9:13739801	9	13739801	300	1	4.10E-07	12	4	AABR07066736.1	
DMR9:16051401	9	16051401	300	1	2.57E-07	7	2.33	Ubr2	Metabolism
DMR9:17890501	9	17890501	7000	1	2.63E-07	79	1.12	Aars2	Transcription
DMR9:21470301	9	21470301	3800	1	5.65E-07	16	0.42		
DMR9:21492201	9	21492201	2000	1	1.52E-07	9	0.45		
DMR9:21579701	9	21579701	3900	1	6.93E-07	12	0.3		
DMR9:21945401	9	21945401	1100	1	7.75E-08	37	3.36		
DMR9:30838001	9	30838001	4200	1	5.82E-09	55	1.3	Col19a1	Cytoskeleton
DMR9:34034401	9	34034401	500	1	4.86E-08	2	0.4		
DMR9:35208701	9	35208701	700	1	7.30E-07	5	0.71		
DMR9:37877601	9	37877601	4100	1	2.58E-07	76	1.85		
DMR9:38102301	9	38102301	400	1	2.17E-07	4	1	Dst	Cell Junction
DMR9:38129001	9	38129001	700	1	5.95E-07	13	1.85	Dst	Cell Junction
DMR9:42373001	9	42373001	2200	1	6.35E-07	31	1.4		
DMR9:45944301	9	45944301	300	1	1.43E-07	3	1	Npas2	Transcription
DMR9:46526401	9	46526401	500	1	3.11E-08	3	0.6		

DMR9:49547001	9	49547001	2300	1	3.86E-07	31	1.34		
DMR9:55001801	9	55001801	300	1	1.39E-07	4	1.33		
DMR9:55373801	9	55373801	2100	2	1.53E-10	31	1.47	Tmeff2	Signaling
DMR9:55574901	9	55574901	300	2	1.08E-09	1	0.33	Tmeff2	Signaling
DMR9:55607701	9	55607701	400	2	5.82E-09	6	1.5	Tmeff2	Signaling
DMR9:55650101	9	55650101	800	1	3.94E-07	1	0.12	Tmeff2	Signaling
DMR9:62304901	9	62304901	400	1	2.11E-08	1	0.25	Plcl1	Signaling
DMR9:63548201	9	63548201	3100	7	1.68E-12	38	1.22	Satb2	Epigenetic
DMR9:68188701	9	68188701	600	1	9.98E-08	1	0.16		
DMR9:70600701	9	70600701	200	1	4.10E-07	0	0		
DMR9:76174801	9	76174801	400	1	1.12E-07	2	0.5		
DMR9:76495401	9	76495401	500	1	3.11E-07	2	0.4		
DMR9:83338001	9	83338001	2100	1	2.55E-07	16	0.76		
DMR9:83528701	9	83528701	600	1	3.03E-07	4	0.66		
DMR9:84394401	9	84394401	400	1	8.57E-07	6	1.5		
DMR9:86304301	9	86304301	500	1	5.65E-07	6	1.2	Dock10	Signaling
DMR9:91896101	9	91896101	300	1	1.45E-07	1	0.33		
DMR9:94060801	9	94060801	1600	1	3.75E-07	13	0.81	Dis3l2	Transcription
DMR9:100269101	9	100269101	1200	1	1.48E-07	17	1.41	AABR07068350.1	
DMR9:106810801	9	106810801	200	1	3.09E-07	6	3		
DMR9:110539901	9	110539901	1100	1	6.93E-07	28	2.54	5S_rRNA;Fbxl17	Proteolysis
DMR9:114457101	9	114457101	300	1	1.45E-07	5	1.66		
DMR9:119547501	9	119547501	400	1	4.53E-07	8	2	Lpin2	
DMR10:1762801	10	1762801	500	1	4.16E-07	4	0.8	AABR07028998.1	
DMR10:2851301	10	2851301	3500	1	3.37E-07	18	0.51		
DMR10:7033401	10	7033401	200	1	3.36E-07	6	3	AC129395.1;Carhsp1	Translation
DMR10:7321101	10	7321101	600	1	1.23E-08	2	0.33		
DMR10:9870101	10	9870101	1300	1	8.54E-07	37	2.84		
DMR10:35222701	10	35222701	400	1	3.64E-07	4	1	AABR07029573.2	
DMR10:38078201	10	38078201	300	1	3.01E-07	11	3.66		
DMR10:39116501	10	39116501	500	1	5.05E-07	11	2.2	Irf1	Immune
DMR10:40696601	10	40696601	300	1	8.11E-07	3	1		
DMR10:40738001	10	40738001	2900	1	3.60E-08	42	1.44	AC127919.1;Sparc	Cytoskeleton
DMR10:41233601	10	41233601	1400	1	3.85E-08	6	0.42		
DMR10:44279201	10	44279201	300	1	8.43E-07	4	1.33	Trim58	Protease
DMR10:56129501	10	56129501	2300	1	7.02E-08	22	0.95	Dnah2	Cytoskeleton
DMR10:58294801	10	58294801	1400	1	4.01E-07	10	0.71		
DMR10:73041001	10	73041001	700	1	3.61E-07	4	0.57		
DMR10:74361601	10	74361601	2900	1	4.92E-07	39	1.34	Gdpd1	Metabolism
DMR10:88972901	10	88972901	3000	1	7.95E-07	55	1.83	Atp6v0a1;Naglu	Metabolism
DMR10:97113901	10	97113901	1800	1	7.85E-07	24	1.33	Cep112	
DMR10:98650501	10	98650501	2000	2	5.28E-09	35	1.75	Abca5	Transport
DMR10:101436901	10	101436901	3700	1	5.45E-07	76	2.05		
DMR10:103330801	10	103330801	1700	1	4.27E-07	23	1.35	Kif19;Btbd17	Cytoskeleton
DMR10:103587701	10	103587701	1200	1	2.08E-08	13	1.08	Cd300le	
DMR11:8748601	11	8748601	1200	1	1.70E-07	15	1.25		
DMR11:9321201	11	9321201	900	1	2.29E-08	9	1		
DMR11:9646201	11	9646201	700	1	1.33E-11	2	0.28	Robo1	Development
DMR11:10663601	11	10663601	400	1	1.41E-07	7	1.75		
DMR11:12598101	11	12598101	1300	3	6.50E-09	10	0.76		
DMR11:15661301	11	15661301	400	1	3.87E-09	1	0.25	AABR07033285.1	
DMR11:15835301	11	15835301	200	1	2.16E-10	4	2		
DMR11:16891201	11	16891201	1100	1	6.13E-07	4	0.36	Btg3	
DMR11:35788501	11	35788501	1100	1	1.51E-08	18	1.63		
DMR11:48430301	11	48430301	500	1	4.52E-08	1	0.2		
DMR11:53243601	11	53243601	200	1	3.45E-07	7	3.5		

DMR11:65886901	11	65886901	400	1	1.72E-07	1	0.25		
DMR11:73511201	11	73511201	3800	1	7.32E-07	68	1.78		
DMR11:73658001	11	73658001	700	1	9.47E-08	6	0.85		
DMR11:82553501	11	82553501	500	1	6.38E-07	3	0.6	Igf2bp2	Transcription
DMR12:1698901	12	1698901	1700	1	2.62E-07	9	0.52	Insr	Receptor
DMR12:7114701	12	7114701	1100	2	1.98E-07	5	0.45	Metazoa_SRP;AABR07035218.1	
DMR12:9938901	12	9938901	2100	1	2.66E-07	28	1.33		
DMR12:12619301	12	12619301	400	1	4.47E-07	5	1.25	Ocm2	Signaling
DMR12:16213801	12	16213801	3400	1	8.53E-07	55	1.61		
DMR12:22918701	12	22918701	6800	1	4.51E-08	82	1.2	Col26a1	Extracellular Matrix
DMR12:23541101	12	23541101	1000	1	3.49E-07	15	1.5	Prkrip1;Orai2	Proteolysis;Metabolism
DMR12:27989201	12	27989201	500	1	1.37E-09	9	1.8		
DMR12:29657801	12	29657801	2100	1	1.84E-07	47	2.23	Caln1	Signaling
DMR12:31212301	12	31212301	500	1	6.11E-08	3	0.6	Adgrd1	
DMR12:34381201	12	34381201	700	1	4.35E-07	1	0.14		
DMR12:36061201	12	36061201	3300	1	9.84E-07	37	1.12	Tmem132b	Unknown
DMR12:43227801	12	43227801	2500	1	9.18E-07	38	1.52	AABR07036435.1	
DMR12:43926001	12	43926001	2700	1	2.81E-08	69	2.55	SNORA17;RGD1562310	Unknown
DMR12:44314001	12	44314001	500	1	6.61E-08	12	2.4	Nos1	Metabolism
DMR12:48141001	12	48141001	2100	1	2.42E-07	34	1.61	Acacb	Metabolism
DMR12:51058601	12	51058601	1000	1	1.37E-08	18	1.8		
DMR12:51199901	12	51199901	2500	2	2.45E-07	41	1.64		
DMR12:52685801	12	52685801	300	1	2.87E-07	5	1.66	Plcxd1	
DMR13:19658001	13	19658001	2600	1	4.72E-08	36	1.38		
DMR13:38619201	13	38619201	300	1	8.16E-07	1	0.33		
DMR13:43068901	13	43068901	500	1	2.36E-08	0	0		
DMR13:43164201	13	43164201	1200	2	3.16E-07	7	0.58		
DMR13:45218201	13	45218201	500	1	1.80E-07	2	0.4	SNORA70	
DMR13:50281701	13	50281701	1000	1	1.43E-07	7	0.7		
DMR13:52755701	13	52755701	300	1	2.63E-07	11	3.66	Pkp1	
DMR13:54883601	13	54883601	600	1	3.05E-07	15	2.5		
DMR13:67323201	13	67323201	1600	1	9.44E-07	15	0.93		
DMR13:68699701	13	68699701	1000	1	9.04E-07	2	0.2	Ivns1abp	Receptor
DMR13:72693201	13	72693201	300	1	2.77E-07	6	2		
DMR13:82695101	13	82695101	5100	1	3.05E-07	48	0.94	Nme7	Signaling
DMR13:83351401	13	83351401	300	1	5.12E-07	1	0.33		
DMR13:83550201	13	83550201	3600	1	1.92E-07	30	0.83	Gpr161	Receptor
DMR13:88617101	13	88617101	500	1	1.82E-07	9	1.8	Sh2d1b	Signaling
DMR13:88840601	13	88840601	400	1	6.51E-07	10	2.5	LOC100361087	
DMR13:90144701	13	90144701	1400	1	5.56E-07	18	1.28	Cd48	Immune
DMR13:90286601	13	90286601	100	1	6.68E-08	2	2	Cd84	Immune
DMR13:90699801	13	90699801	300	2	1.70E-07	14	4.66	Igsf8;Kcnj9	Immune;Metabolism
DMR13:91047201	13	91047201	400	1	3.41E-09	5	1.25	Crp	
DMR13:94304501	13	94304501	1100	1	3.49E-07	7	0.63	Pld5	Signaling
DMR13:97565401	13	97565401	3000	1	4.57E-08	48	1.6		
DMR13:99248001	13	99248001	1000	1	5.71E-07	8	0.8	Tmem63a	Unknown
DMR13:104913101	13	104913101	1400	2	1.70E-07	10	0.71		
DMR13:108998901	13	108998901	400	1	4.92E-07	12	3		
DMR13:111044601	13	111044601	1000	1	8.57E-07	7	0.7	Kcnh1	Transport
DMR13:111936101	13	111936101	1100	1	1.33E-07	10	0.9	Hsd11b1	Metabolism
DMR13:113159801	13	113159801	400	1	3.09E-08	2	0.5		
DMR13:113766301	13	113766301	1200	1	2.25E-11	11	0.91	AC118802.1	

DMR14:10805401	14	10805401	2100	1	3.60E-07	41	1.95	Lin54	Cell Cycle
DMR14:13231601	14	13231601	400	1	9.69E-07	8	2	Antxr2	Receptor
DMR14:14841301	14	14841301	400	1	8.35E-09	12	3		
DMR14:14906501	14	14906501	1400	1	1.03E-08	19	1.35		
DMR14:16617701	14	16617701	400	1	4.38E-07	6	1.5	Shroom3	Cytoskeleton
DMR14:18202501	14	18202501	800	1	6.92E-07	3	0.37		
DMR14:19051501	14	19051501	800	1	2.53E-07	17	2.12	LOC360919	
DMR14:28802901	14	28802901	1800	1	5.39E-07	20	1.11	Adgrl3	
DMR14:31984701	14	31984701	200	1	8.16E-07	2	1		
DMR14:35162701	14	35162701	2900	1	5.45E-07	38	1.31	U6	
DMR14:37342601	14	37342601	2300	1	3.21E-07	35	1.52	Cwh43	Development
DMR14:42294301	14	42294301	800	1	3.69E-07	14	1.75		
DMR14:43227101	14	43227101	1700	1	6.00E-07	28	1.64		
DMR14:54837401	14	54837401	300	1	8.98E-08	3	1		
DMR14:67970401	14	67970401	1300	1	4.51E-07	2	0.15		
DMR14:75880701	14	75880701	200	1	5.42E-07	6	3	Hs3st1	Golgi
DMR14:81457501	14	81457501	1800	1	8.79E-07	16	0.88	Sh3bp2;U1	Translation
DMR14:91208801	14	91208801	300	1	3.79E-07	2	0.66	Vwc2	
DMR14:96487101	14	96487101	1600	1	5.74E-08	55	3.43		
DMR14:96738301	14	96738301	1100	1	7.30E-07	23	2.09		
DMR14:96899601	14	96899601	1300	2	3.19E-07	43	3.3	Rn50_14_0967.1	
DMR14:97299301	14	97299301	300	1	8.82E-08	8	2.66		
DMR14:106825001	14	106825001	1100	1	6.88E-07	19	1.72		
DMR14:107126701	14	107126701	1100	1	6.55E-08	8	0.72	Ehbp1	Unknown
DMR14:112172501	14	112172501	300	1	5.80E-08	0	0		
DMR14:114656701	14	114656701	300	1	7.06E-07	2	0.66	Sptbn1	Cytoskeleton
DMR15:143301	15	143301	600	1	7.91E-08	1	0.16	Dlg5	Extracellular Matrix
DMR15:2929001	15	2929001	1900	1	6.70E-07	14	0.73	Kat6b	Epigenetic
DMR15:3466801	15	3466801	4400	1	1.87E-07	75	1.7	Vcl	Extracellular Matrix
DMR15:7402301	15	7402301	1300	1	1.51E-07	22	1.69	AABR07017045.1	
DMR15:10734601	15	10734601	400	1	3.73E-07	6	1.5		
DMR15:12051801	15	12051801	2800	1	6.38E-08	30	1.07		
DMR15:15354501	15	15354501	200	1	3.11E-08	1	0.5	Cadps	Metabolism
DMR15:15746201	15	15746201	400	1	2.89E-07	3	0.75		
DMR15:25204301	15	25204301	400	1	2.28E-07	8	2		
DMR15:26005801	15	26005801	1200	1	1.70E-07	16	1.33		
DMR15:27801901	15	27801901	300	1	9.20E-07	8	2.66	Tep1	Transcription
DMR15:47617701	15	47617701	500	1	8.56E-08	10	2	Msra	Metabolism
DMR15:49745501	15	49745501	2900	1	5.00E-07	78	2.68		
DMR15:50974001	15	50974001	800	1	1.58E-07	2	0.25		
DMR15:53896901	15	53896901	500	1	7.62E-07	0	0		
DMR15:57018501	15	57018501	1200	1	8.93E-07	15	1.25		
DMR15:63615801	15	63615801	200	1	1.04E-07	1	0.5		
DMR15:65278201	15	65278201	1600	1	2.75E-07	38	2.37		
DMR15:65280901	15	65280901	1500	3	8.40E-09	35	2.33		
DMR15:67561301	15	67561301	100	1	1.71E-10	4	4	Pcdh17	Cytoskeleton
DMR15:70972501	15	70972501	400	2	1.16E-11	1	0.25		
DMR15:72298801	15	72298801	200	1	7.49E-07	3	1.5		
DMR15:73753301	15	73753301	1000	1	2.58E-07	3	0.3		
DMR15:82875201	15	82875201	500	1	9.77E-08	1	0.2		
DMR15:86918901	15	86918901	500	1	6.04E-08	6	1.2		
DMR15:88517001	15	88517001	1600	2	6.48E-08	24	1.5	Mycbp2	Metabolism
DMR15:92675301	15	92675301	500	1	6.16E-07	5	1	Mycbp2	Metabolism
DMR15:103091201	15	103091201	1100	1	6.14E-08	27	2.45	Gpc6	Extracellular Matrix

DMR15:107289801	15	107289801	1300	3	4.79E-11	41	3.15		
DMR16:8717801	16	8717801	500	2	5.24E-09	1	0.2		
DMR16:11115801	16	11115801	2200	1	5.10E-07	34	1.54	Wapl	
DMR16:16521301	16	16521301	500	1	9.14E-07	4	0.8		
DMR16:17679501	16	17679501	600	1	1.53E-08	3	0.5	Sh2d4b	
DMR16:20002301	16	20002301	400	1	2.78E-07	4	1	Nxnl1;Slc27a1	Metabolism
DMR16:23508501	16	23508501	400	1	3.49E-07	11	2.75	Psd3	Signaling
DMR16:30239801	16	30239801	1300	1	1.01E-07	35	2.69		
DMR16:31118101	16	31118101	1700	1	6.93E-07	46	2.7		
DMR16:37149501	16	37149501	300	1	1.52E-07	4	1.33	Fbxo8	Signaling
DMR16:47487901	16	47487901	2900	1	1.29E-08	51	1.75	Wwc2	Unknown
DMR16:47793101	16	47793101	1500	1	2.13E-07	31	2.06		
DMR16:56583301	16	56583301	400	1	5.18E-07	2	0.5		
DMR16:59593401	16	59593401	2200	1	5.42E-07	44	2	Lonrf1	
DMR16:60372701	16	60372701	400	1	9.68E-08	4	1		
DMR16:71000401	16	71000401	1000	1	9.44E-07	27	2.7	Kcnu1;Ash2l	Transcription
DMR16:71883201	16	71883201	1800	1	3.64E-07	23	1.27	Adam9;Adam32	Protease
DMR16:72270401	16	72270401	700	1	1.37E-07	0	0	Ido2	
DMR16:74439501	16	74439501	300	1	1.88E-07	13	4.33		
DMR16:74514901	16	74514901	500	1	2.21E-07	8	1.6	AABR07026377.1	
DMR16:79325301	16	79325301	1900	1	2.42E-07	32	1.68		
DMR16:80999901	16	80999901	1300	1	7.81E-08	31	2.38	Tmco3;Dcun1d2	Transport;Proteolysis
DMR16:82506001	16	82506001	1300	1	5.10E-07	20	1.53	7SK	
DMR16:84735401	16	84735401	1400	1	2.08E-07	19	1.357	Myo16	Cytoskeleton
DMR17:4704901	17	4704901	2800	1	2.55E-07	35	1.25	AABR07026912.1	
DMR17:6427401	17	6427401	1100	2	1.77E-07	15	1.36		
DMR17:8655301	17	8655301	2100	1	1.57E-07	39	1.85		
DMR17:13905901	17	13905901	1500	1	1.95E-07	33	2.2		
DMR17:18565401	17	18565401	1200	1	9.73E-07	26	2.16	Cap2	Cytoskeleton
DMR17:18972601	17	18972601	2100	1	4.22E-07	19	0.9		
DMR17:19412501	17	19412501	4400	1	1.98E-07	57	1.29	Atxn1	Transcription
DMR17:23652001	17	23652001	600	1	8.12E-08	2	0.33	7SK;Phactr1	Signaling
DMR17:28789101	17	28789101	200	1	7.81E-07	2	1		
DMR17:33348201	17	33348201	300	1	6.28E-07	2	0.66		
DMR17:35863901	17	35863901	1600	1	3.86E-07	14	0.87		
DMR17:36158601	17	36158601	1400	1	2.56E-07	10	0.71	Mboat1	Metabolism
DMR17:36934301	17	36934301	300	2	1.44E-09	1	0.33	Cdkal1	Cell Cycle
DMR17:41658701	17	41658701	400	1	8.71E-07	12	3		
DMR17:43163301	17	43163301	600	1	8.19E-08	0	0	Carmil1	
DMR17:44784401	17	44784401	500	2	4.33E-07	3	0.6	Hist1h2bo;Hist1h2ac;AABR07072559.1	Transcription
DMR17:55628201	17	55628201	300	1	3.33E-08	21	7		
DMR17:60463701	17	60463701	1100	1	2.00E-07	2	0.18	Armc4	
DMR17:65283601	17	65283601	900	1	5.25E-07	5	0.55		
DMR17:65295101	17	65295101	500	1	6.51E-07	6	1.2		
DMR17:79560001	17	79560001	1500	1	3.62E-07	18	1.2	Itga8	Extracellular Matrix
DMR17:85800601	17	85800601	600	1	4.75E-07	6	1		
DMR18:6539601	18	6539601	1000	1	6.90E-08	16	1.6		
DMR18:7035701	18	7035701	400	1	5.22E-07	1	0.25	Chst9	Metabolism
DMR18:8442401	18	8442401	300	1	8.93E-07	0	0		
DMR18:10304701	18	10304701	1600	2	1.35E-07	20	1.25		
DMR18:17279001	18	17279001	1400	1	6.42E-07	23	1.64	Fhod3	Cytoskeleton
DMR18:31722901	18	31722901	2400	1	2.57E-07	34	1.41	Nr3c1	Transcription
DMR18:51741101	18	51741101	1300	1	2.03E-07	24	1.84	AC118858.1	

DMR18:51877901	18	51877901	400	1	6.04E-08	12	3	3-Mar	Metabolism
DMR18:52177001	18	52177001	600	1	9.11E-11	3	0.5		
DMR18:58434001	18	58434001	300	1	1.12E-07	10	3.33	Piezo2	
DMR18:60511801	18	60511801	400	1	3.41E-08	1	0.25	Nedd4l	Protease
DMR18:60532401	18	60532401	2300	7	1.22E-24	38	1.65	Nedd4l	Protease
DMR18:62686201	18	62686201	3300	1	7.21E-09	34	1.03		
DMR18:64423601	18	64423601	1100	1	4.05E-07	19	1.72		
DMR18:73747801	18	73747801	1700	1	4.52E-07	31	1.82	Loxhd1	
DMR18:74897901	18	74897901	200	1	3.11E-07	4	2		
DMR18:77216101	18	77216101	600	2	8.85E-10	16	2.66	Nfatc1	Transcription
DMR18:77488301	18	77488301	400	1	2.90E-08	1	0.25	Atp9b	Transport
DMR18:78406701	18	78406701	1000	1	3.26E-09	10	1		
DMR18:79240401	18	79240401	1300	1	2.63E-07	15	1.15	Galr1	Receptor
DMR18:87853401	18	87853401	800	2	2.50E-08	40	5		
DMR19:11439101	19	11439101	1400	2	6.22E-08	22	1.57	Ogfod1	Metabolism
DMR19:15937001	19	15937001	1500	2	5.46E-07	13	0.86		
DMR19:28304301	19	28304301	1800	1	8.74E-07	52	2.88	AABR07043429.1	
DMR19:28370301	19	28370301	2500	1	9.05E-07	63	2.52	Vom1r21	Receptor
DMR19:34900401	19	34900401	400	1	1.35E-07	1	0.25		
DMR19:38423401	19	38423401	300	1	1.78E-07	11	3.66	Nqo1	Metabolism
DMR19:38794601	19	38794601	1400	1	9.96E-09	11	0.78	Cdh1	Cytoskeleton
DMR19:44508401	19	44508401	200	1	7.89E-08	7	3.5		
DMR19:46231601	19	46231601	1100	1	5.10E-07	24	2.18		
DMR19:47512101	19	47512101	800	1	3.07E-07	6	0.75		
DMR19:47608901	19	47608901	1000	1	2.50E-07	21	2.1		
DMR19:51890901	19	51890901	300	2	3.87E-08	5	1.66	Cdh13	Extracellular Matrix
DMR19:52295501	19	52295501	900	1	1.39E-08	9	1		
DMR19:57903401	19	57903401	1700	1	7.59E-07	35	2.05	Disc1	
DMR19:59662501	19	59662501	800	1	5.83E-07	14	1.75	AABR07044148.1	
DMR19:59942301	19	59942301	400	1	9.43E-08	4	1		
DMR20:57001	20	57001	1300	1	3.11E-07	29	2.23	7SK	
DMR20:5229401	20	5229401	200	1	1.65E-07	6	3	RT1-DMb	
DMR20:6310401	20	6310401	1500	1	6.30E-09	18	1.2	SCARNA14;Rn60_20_0064.1	
DMR20:6785001	20	6785001	500	1	3.79E-08	4	0.8	Ppil1;Grm4	Immune;Receptor
DMR20:8596701	20	8596701	700	1	4.04E-07	16	2.28		
DMR20:10283601	20	10283601	1200	1	9.52E-07	21	1.75	Ndufv3;SNORA36	Metabolism
DMR20:12360901	20	12360901	1200	1	2.57E-07	41	3.41	Slc19a1;AABR07044596.1	Transport
DMR20:18932301	20	18932301	500	1	4.62E-08	16	3.2	Bicc1	Transcription
DMR20:19484001	20	19484001	1900	6	1.02E-11	24	1.26	Fam13c	
DMR20:19852601	20	19852601	4000	1	7.91E-08	57	1.42	AABR07044765.1	
DMR20:20981401	20	20981401	600	1	1.64E-07	3	0.5	AABR07044799.1	
DMR20:33543501	20	33543501	1500	1	5.42E-07	28	1.86	Nepn	
DMR20:40898401	20	40898401	600	4	4.38E-11	22	3.66		
DMR20:42206301	20	42206301	500	1	5.43E-07	3	0.6		
DMR20:44638501	20	44638501	1500	1	7.69E-07	37	2.46	Fyn	Transcription
DMR20:47584201	20	47584201	5400	1	5.78E-07	88	1.62	Scml4	Transcription
DMR20:47897901	20	47897901	600	1	5.68E-07	14	2.33	Sobp	
DMR20:48054301	20	48054301	3200	3	2.94E-09	61	1.9	Pdss2	Metabolism
DMRMT:1	MT	1	12900	33	8.09E-13	241	1.86	AY172581.13;AY172581.9;AY172581.3;	

DMRMT:14001	MT	14001	2313	5	2.28E-09	47	2.03	Mt-nd2;AY172581.6;AY172581.22;AY172581.18;AY172581.10;AY172581.7;	
DMRX:290501	X	290501	1400	1	4.32E-07	38	2.71		
DMRX:10934701	X	10934701	1800	1	1.03E-07	17	0.94	Rn60_X_0110.2;Rn60_X_0110.1	
DMRX:36450301	X	36450301	800	3	1.12E-08	30	3.75		
DMRX:36466401	X	36466401	2800	1	8.79E-07	60	2.14		
DMRX:40976801	X	40976801	500	1	6.33E-07	4	0.8		
DMRX:46749301	X	46749301	800	1	3.63E-07	4	0.5		
DMRX:61107301	X	61107301	600	1	3.66E-09	13	2.16	Mageb4	
DMRX:117456201	X	117456201	500	1	5.68E-07	7	1.4		
DMRX:123467901	X	123467901	300	1	3.69E-07	2	0.66	RGD1564541	Unknown
DMRX:150474201	X	150474201	7700	1	9.34E-07	65	0.84		
DMRX:150492601	X	150492601	1300	1	2.85E-07	32	2.46		
DMRY:1208001	Y	1208001	500	1	8.72E-08	1	0.2	Ddx3;Rn60_Y_0010.2	
