# Supplementary material for: Alterations in sperm DNA methylation, non-coding RNA and histone retention associate with DDT-induced epigenetic transgenerational inheritance of disease
Source: Epigenetics Chromatin. 2018 Feb 27;11:8. doi: 10.1186/s13072-018-0178-0 (PMC5827984; doi:10.1186/s13072-018-0178-0)
Supplement: Supplementary file 4 — Additional file 4: Table S2. F2 DMR p < 1e−06. [file 13072_2018_178_MOESM4_ESM.pdf]

Supplemental Table S2
F2 DMR p<1e-06

DMR Name	Chr	Start	(bp) Length	# SigWin	minP	CpG #	CpG Density	Gene Association	Gene Category
DMR1:617901	1	617901	4200	3	1.93E-09	47	1.119047619		
DMR1:2151701	1	2151701	400	1	3.87E-07	0	0		
DMR1:2635101	1	2635101	1900	1	1.41E-07	38	2	Ust	Metabolism
DMR1:2724301	1	2724301	300	1	5.25E-07	0	0	U6	
DMR1:2900401	1	2900401	500	2	3.17E-07	1	0.2		
DMR1:6121201	1	6121201	1600	2	2.34E-07	6	0.375		
DMR1:6145601	1	6145601	900	4	8.00E-11	9	1		
DMR1:6386901	1	6386901	600	2	6.08E-09	0	0		
DMR1:7041801	1	7041801	1000	1	1.57E-08	26	2.6	Stx11	Transport
DMR1:7071801	1	7071801	3300	1	4.52E-07	35	1.060606061	Stx11	Transport
DMR1:7881501	1	7881501	2000	1	9.88E-07	46	2.3	Aig1	
DMR1:7891901	1	7891901	6100	1	1.23E-07	81	1.327868852	Aig1	
DMR1:8147801	1	8147801	800	1	9.82E-07	15	1.875		
DMR1:8358101	1	8358101	1600	1	7.67E-07	14	0.875		
DMR1:8805901	1	8805901	2000	1	2.27E-07	15	0.75		
DMR1:9136301	1	9136301	5300	1	8.13E-07	44	0.830188679		
DMR1:9169001	1	9169001	1400	3	2.15E-11	9	0.642857143		
DMR1:9606501	1	9606501	1400	2	9.47E-08	10	0.714285714		
DMR1:10539001	1	10539001	300	1	3.28E-07	2	0.666666667		
DMR1:10929101	1	10929101	400	1	7.29E-08	1	0.25		
DMR1:11053901	1	11053901	4100	1	6.08E-08	40	0.975609756		
DMR1:11638101	1	11638101	400	2	1.23E-10	1	0.25		
DMR1:11908601	1	11908601	11800	1	7.83E-08	878	7.440677966	5_8S_rRNA;pRNA	

DMR1:11959301	1	11959301	8300	2	1.91E-07	668	8.048192771	pRNA;AABR07000398.1;5_8S_rRNA;AABR07000402.1;AABR07000404.1	
DMR1:14646701	1	14646701	1700	2	2.69E-07	17	1		
DMR1:15047301	1	15047301	4700	2	7.95E-08	79	1.680851064		
DMR1:15247501	1	15247501	2300	1	8.33E-08	33	1.434782609	AC136053.1	
DMR1:15470101	1	15470101	1500	2	8.02E-09	14	0.933333333	Map3k5	Signaling
DMR1:16118901	1	16118901	1800	1	7.21E-11	12	0.666666667	Pde7b	Signaling
DMR1:16663401	1	16663401	1900	1	6.30E-07	25	1.315789474	Myb	Transcription
DMR1:16748101	1	16748101	500	2	1.28E-12	0	0		
DMR1:18441101	1	18441101	3300	1	8.86E-09	34	1.03030303		
DMR1:20455001	1	20455001	2300	1	1.21E-07	16	0.695652174		
DMR1:20489801	1	20489801	300	1	2.35E-07	0	0		
DMR1:20562001	1	20562001	1100	1	2.70E-07	2	0.181818182		
DMR1:20629601	1	20629601	4700	3	4.94E-09	27	0.574468085		
DMR1:20728001	1	20728001	500	1	4.81E-08	10	2	AABR07000639.1	
DMR1:20806101	1	20806101	1300	1	8.28E-08	5	0.384615385		
DMR1:21651101	1	21651101	2400	1	5.89E-07	27	1.125	Enpp3	
DMR1:21784401	1	21784401	400	2	4.49E-12	3	0.75	Enpp1	Signaling
DMR1:21917001	1	21917001	2100	1	2.54E-07	10	0.476190476		
DMR1:22238401	1	22238401	2400	2	1.32E-07	11	0.458333333	Stx7	Transcription
DMR1:23129101	1	23129101	700	1	5.13E-10	2	0.285714286		
DMR1:23539601	1	23539601	1900	1	9.06E-07	2	0.105263158	Eya4;LOC100910446	Transcription
DMR1:23803001	1	23803001	400	1	7.89E-07	2	0.5		
DMR1:24417001	1	24417001	1500	1	4.52E-10	27	1.8		
DMR1:24657501	1	24657501	500	1	6.99E-08	3	0.6		
DMR1:24782601	1	24782601	1500	1	4.34E-07	5	0.333333333		

DMR1:25114901	1	25114901	2300	1	1.63E-07	16	0.695652174		
DMR1:26633001	1	26633001	400	1	9.43E-08	1	0.25		
DMR1:26641101	1	26641101	3700	4	1.64E-13	34	0.918918919		
DMR1:26651501	1	26651501	200	1	3.11E-07	2	1		
DMR1:26657501	1	26657501	3200	1	6.90E-07	51	1.59375		
DMR1:26804001	1	26804001	900	1	1.81E-07	9	1		
DMR1:27119101	1	27119101	300	1	2.12E-07	0	0		
DMR1:28908601	1	28908601	3400	1	1.08E-07	19	0.558823529		
DMR1:29077401	1	29077401	2400	1	7.38E-07	59	2.458333333		
DMR1:29715601	1	29715601	2200	1	7.48E-08	4	0.181818182		
DMR1:29976601	1	29976601	1800	1	6.06E-07	9	0.5		
DMR1:31259901	1	31259901	400	1	1.54E-07	1	0.25	Mrpl36;LOC679739	Translation;Metabolism
DMR1:31679901	1	31679901	1700	1	4.92E-08	11	0.647058824	Ahrr	Transcription
DMR1:32468301	1	32468301	800	1	1.58E-07	6	0.75	AABR07001003.1	
DMR1:33501601	1	33501601	800	1	6.62E-07	2	0.25		
DMR1:33730101	1	33730101	500	2	5.54E-10	0	0	AABR07001018.5;AABR07001018.3	
DMR1:34025601	1	34025601	800	2	1.02E-08	7	0.875		
DMR1:34243201	1	34243201	300	1	2.07E-07	1	0.333333333		
DMR1:34389801	1	34389801	900	1	1.13E-07	5	0.555555556		
DMR1:34882001	1	34882001	2500	1	3.52E-07	27	1.08		
DMR1:34950001	1	34950001	1600	1	1.78E-07	17	1.0625		
DMR1:35998801	1	35998801	3100	1	5.43E-08	22	0.709677419	AABR07001054.2	
DMR1:36610801	1	36610801	400	3	9.17E-11	3	0.75	AABR07001061.1	
DMR1:37549001	1	37549001	1100	1	8.06E-07	7	0.636363636	Adcy2	Metabolism
DMR1:38081301	1	38081301	3700	1	6.40E-07	28	0.756756757		
DMR1:38350101	1	38350101	400	2	2.70E-07	2	0.5		
DMR1:38442101	1	38442101	1500	1	3.80E-07	19	1.266666667		

DMR1:39248301	1	39248301	1500	1	2.55E-08	13	0.866666667		
DMR1:39748001	1	39748001	1000	1	1.58E-07	5	0.5		
DMR1:40483001	1	40483001	1000	1	2.14E-07	18	1.8	Plekhg1	Signaling
DMR1:40825201	1	40825201	300	1	9.13E-07	1	0.333333333	Akap12	
DMR1:41389901	1	41389901	1100	1	3.79E-08	9	0.818181818	Esr1;SNORD88	Receptor
DMR1:42311501	1	42311501	1500	1	2.98E-07	5	0.333333333		
DMR1:42392701	1	42392701	800	1	1.03E-07	2	0.25		
DMR1:43412101	1	43412101	1000	3	6.37E-09	3	0.3		
DMR1:43851701	1	43851701	2900	1	1.61E-07	34	1.172413793	Cnksr3	Signaling
DMR1:44142401	1	44142401	2300	1	5.01E-07	27	1.173913043		
DMR1:44727901	1	44727901	300	1	7.03E-09	0	0		
DMR1:44731401	1	44731401	400	1	3.72E-07	1	0.25		
DMR1:45037901	1	45037901	700	3	2.97E-10	1	0.142857143		
DMR1:45091801	1	45091801	1000	1	3.30E-07	7	0.7		
DMR1:45158501	1	45158501	300	1	9.18E-07	1	0.333333333		
DMR1:45614001	1	45614001	800	2	3.16E-10	7	0.875	AABR07001455.1	
DMR1:45743101	1	45743101	2500	1	8.14E-07	27	1.08		
DMR1:46756801	1	46756801	1500	1	1.01E-07	9	0.6		
DMR1:47275301	1	47275301	400	1	7.79E-07	0	0	Sytl3	Transport
DMR1:47829801	1	47829801	1300	3	1.04E-14	11	0.846153846		
DMR1:48077501	1	48077501	1600	1	4.41E-07	19	1.1875	Mas1l;Mas1	Receptor
DMR1:48331101	1	48331101	1100	1	1.09E-09	0	0	Slc22a2;AC114389.1	Transport
DMR1:48884201	1	48884201	1000	1	8.25E-07	11	1.1	AC135026.1	
DMR1:50277001	1	50277001	2000	1	1.01E-08	23	1.15		
DMR1:51268801	1	51268801	2000	1	4.83E-07	13	0.65		
DMR1:51386801	1	51386801	1900	1	1.17E-07	17	0.894736842		
DMR1:51995401	1	51995401	300	1	1.19E-09	0	0		
DMR1:52201701	1	52201701	200	1	2.29E-07	0	0		

DMR1:52730001	1	52730001	600	2	3.96E-10	2	0.333333333		
DMR1:53045901	1	53045901	1200	2	4.00E-08	13	1.083333333	Mpc1	
DMR1:53095501	1	53095501	300	2	5.77E-09	0	0	Ccr6	
DMR1:53202901	1	53202901	300	1	4.47E-07	0	0		
DMR1:53344501	1	53344501	800	1	8.33E-07	2	0.25		
DMR1:53542401	1	53542401	4100	1	7.04E-07	52	1.268292683	Tcp10b	
DMR1:53670101	1	53670101	900	1	5.23E-07	6	0.666666667		
DMR1:54243001	1	54243001	2300	1	1.97E-09	23	1	AABR07001610.1	
DMR1:54824701	1	54824701	2300	1	1.81E-07	26	1.130434783		
DMR1:54948501	1	54948501	2400	1	1.89E-08	13	0.541666667		
DMR1:54989801	1	54989801	3400	1	5.70E-08	22	0.647058824		
DMR1:55448801	1	55448801	1200	1	5.29E-08	8	0.666666667		
DMR1:55469801	1	55469801	3000	1	5.71E-08	15	0.5		
DMR1:55732001	1	55732001	1600	1	9.77E-07	8	0.5	AABR07001734.1	
DMR1:55754401	1	55754401	3300	2	2.13E-08	16	0.484848485	AABR07001734.1	
DMR1:55996801	1	55996801	500	1	2.14E-07	3	0.6		
DMR1:57456001	1	57456001	1200	4	1.63E-09	2	0.166666667		
DMR1:57484401	1	57484401	800	1	6.54E-07	11	1.375	Psmb1;Tbp	Protease;Transcription
DMR1:58231501	1	58231501	2200	1	9.07E-07	16	0.727272727		
DMR1:59091501	1	59091501	600	1	9.70E-07	5	0.833333333		
DMR1:61511401	1	61511401	3100	1	2.66E-07	41	1.322580645	LOC108348215;Zfp51	
DMR1:63029401	1	63029401	6800	3	3.05E-09	85	1.25		
DMR1:63063601	1	63063601	1500	2	2.77E-07	7	0.466666667		
DMR1:64502201	1	64502201	1000	1	2.55E-07	12	1.2	Olr386;Nlrp12	
DMR1:65207701	1	65207701	2900	1	6.14E-07	25	0.862068966	Vom2r80	Receptor
DMR1:65355101	1	65355101	1100	3	3.92E-09	4	0.363636364		
DMR1:65946501	1	65946501	300	2	3.29E-09	0	0	Vom2r34;Vom2r33	

DMR1:66278901	1	66278901	1700	1	2.73E-08	5	0.294117647	AABR07002093.1	
DMR1:67862701	1	67862701	300	2	6.64E-09	1	0.333333333	Vom2r32	
DMR1:69108201	1	69108201	400	1	2.48E-07	0	0		
DMR1:70009801	1	70009801	1400	1	4.80E-07	7	0.5	Usp29	Proteolysis
DMR1:70167401	1	70167401	200	1	9.29E-07	1	0.5		
DMR1:70399401	1	70399401	700	1	3.74E-07	9	1.285714286	AABR07002262.1	
DMR1:70428401	1	70428401	1500	1	9.86E-07	25	1.666666667	Olr3	
DMR1:71093501	1	71093501	400	1	2.24E-07	1	0.25		
DMR1:71323801	1	71323801	3900	3	3.35E-10	37	0.948717949	Zscan5b	Transcription
DMR1:71768801	1	71768801	400	1	6.32E-07	2	0.5	AABR07002351.2	
DMR1:71780001	1	71780001	800	1	1.87E-07	5	0.625	Obox2	Transcription
DMR1:72084801	1	72084801	300	1	2.46E-08	4	1.333333333		
DMR1:72649001	1	72649001	4200	1	4.87E-07	31	0.738095238	Il11;Fam71e2;Cox6b2	Electron Transport
DMR1:72723101	1	72723101	2400	2	7.40E-11	28	1.166666667	Brsk1;Hspbp1	Signaling
DMR1:72776701	1	72776701	500	1	9.29E-08	4	0.8	Ppp6r1;Tmem86b	Unknown
DMR1:77294601	1	77294601	1000	2	2.64E-09	2	0.2		
DMR1:77583401	1	77583401	1300	2	1.25E-07	8	0.615384615		
DMR1:78062401	1	78062401	1400	2	4.85E-08	3	0.214285714	Slc8a2;Meis3	Metabolism;Transcription
DMR1:78605201	1	78605201	400	1	2.51E-07	4	1		
DMR1:79798401	1	79798401	600	1	7.19E-07	30	5	Pglyrp1;Ccdc61	Receptor
DMR1:79824801	1	79824801	1000	1	5.62E-08	9	0.9	Ccdc61;Nova2	Transcription
DMR1:81321701	1	81321701	400	2	4.44E-15	7	1.75	Plaur	Receptor
DMR1:81446001	1	81446001	1800	1	3.11E-08	25	1.388888889	Xrcc1;Zfp575;Ethe1	Transcription;Metabolism
DMR1:81802101	1	81802101	3200	1	5.61E-08	85	2.65625	Arhgef1	Signaling
DMR1:82046401	1	82046401	2200	1	3.58E-07	59	2.681818182		
DMR1:82446301	1	82446301	900	5	3.30E-08	0	0	Bckdha;Exosc5	Metabolism

DMR1:83519401	1	83519401	2000	1	9.17E-08	5	0.25	Cyp2b12	Metabolism
DMR1:83611601	1	83611601	1300	1	5.97E-07	5	0.384615385		
DMR1:83982401	1	83982401	5100	1	2.54E-07	77	1.509803922	Egln2;Rab4b;Mia	Development;Signaling;Transcription
DMR1:84079101	1	84079101	1600	1	6.76E-07	22	1.375	Numbl	Signaling
DMR1:84434601	1	84434601	500	1	1.47E-07	6	1.2	Akt2	Signaling
DMR1:84996901	1	84996901	3000	1	1.65E-07	42	1.4	U2;Fcgbp	Extracellular Matrix
DMR1:85259101	1	85259101	4200	1	7.91E-07	29	0.69047619	Ifnl1	
DMR1:85935901	1	85935901	300	1	2.28E-07	1	0.333333333		
DMR1:86847901	1	86847901	1100	2	1.37E-10	14	1.272727273	AABR07002848.2	
DMR1:86911701	1	86911701	2100	1	3.88E-07	24	1.142857143	Fbxo17;Mrps12;Sars2	Transcription
DMR1:90150601	1	90150601	1200	1	2.37E-07	12	1	RGD1308428	
DMR1:91398401	1	91398401	2700	1	6.59E-07	45	1.666666667		
DMR1:91403201	1	91403201	1900	2	3.06E-08	22	1.157894737		
DMR1:92147101	1	92147101	600	1	2.26E-07	6	1		
DMR1:92681401	1	92681401	600	1	4.02E-07	2	0.333333333		
DMR1:92805601	1	92805601	600	2	1.18E-08	3	0.5		
DMR1:92858201	1	92858201	5300	1	3.66E-09	81	1.528301887		
DMR1:92917001	1	92917001	900	1	3.69E-07	12	1.333333333	AABR07003007.1	
DMR1:93356901	1	93356901	800	2	1.64E-07	11	1.375		
DMR1:93757001	1	93757001	1900	3	1.70E-12	24	1.263157895		
DMR1:93808801	1	93808801	1200	1	1.84E-08	12	1		
DMR1:93957901	1	93957901	400	1	2.52E-07	3	0.75	Zfp536	Transcription
DMR1:94508101	1	94508101	4400	1	1.97E-08	53	1.204545455		
DMR1:95207701	1	95207701	1400	4	4.09E-11	2	0.142857143		
DMR1:95219001	1	95219001	800	1	7.46E-10	9	1.125		

DMR1:95906701	1	95906701	300	1	3.75E-07	1	0.333333333		
DMR1:96119401	1	96119401	1900	4	1.62E-07	21	1.105263158		
DMR1:97011101	1	97011101	600	1	1.86E-07	2	0.333333333		
DMR1:97904801	1	97904801	3400	5	4.17E-14	21	0.617647059		
DMR1:97909401	1	97909401	2400	3	4.64E-09	5	0.208333333		
DMR1:98561401	1	98561401	800	5	1.54E-18	0	0	Siglec5	Immune
DMR1:99043001	1	99043001	4600	1	4.75E-08	45	0.97826087	Vom2r38	Receptor
DMR1:99507001	1	99507001	1300	1	6.60E-07	10	0.769230769	Zfp719	
DMR1:99579501	1	99579501	3300	1	1.42E-09	44	1.333333333	Ceacam18;Siglec8	Extracellular Matrix
DMR1:99622701	1	99622701	600	2	1.31E-09	1	0.166666667	Klk14;LOC103691107	Protease
DMR1:100174601	1	1E+08	300	1	2.09E-07	4	1.333333333	Klk1;LOC108348116	Metabolism
DMR1:101095801	1	1.01E+08	2300	1	5.05E-07	30	1.304347826	Rcn3;Fcgrt	Metabolism;Receptor
DMR1:101403101	1	1.01E+08	1600	1	6.19E-07	14	0.875	Kcna7;Ntf4;Lhb;Ruvbl2	Transport;Signaling;Transcription
DMR1:101588901	1	1.02E+08	1200	1	4.16E-07	11	0.916666667	Fgf21;Fut1	Metabolism
DMR1:101641401	1	1.02E+08	400	1	2.67E-07	5	1.25	Fut2	Metabolism
DMR1:101647501	1	1.02E+08	4000	1	2.08E-07	62	1.55	Fut2	Metabolism
DMR1:101709301	1	1.02E+08	1400	1	3.17E-07	20	1.428571429	Rpl18;Fam83e;Spaca4;Sult2b1	Transcription;Metabolism
DMR1:101853101	1	1.02E+08	1700	1	1.05E-08	27	1.588235294	Grin2d;Kdelr1	Receptor
DMR1:101988601	1	1.02E+08	4200	1	9.68E-07	64	1.523809524	Abcc6	Transport
DMR1:102306301	1	1.02E+08	1300	1	3.28E-09	23	1.769230769	Otog	Unknown
DMR1:102961701	1	1.03E+08	300	1	1.03E-07	1	0.333333333	Tsg101	Metabolism
DMR1:103087001	1	1.03E+08	500	1	2.84E-09	5	1		
DMR1:103346601	1	1.03E+08	1500	1	4.59E-07	12	0.8		

DMR1:103732301	1	1.04E+08	2900	3	1.05E-11	12	0.413793103	Mrgprx2	Receptor
DMR1:105083701	1	1.05E+08	300	1	3.84E-08	1	0.333333333		
DMR1:105129701	1	1.05E+08	2100	1	2.10E-09	8	0.380952381	Prmt3	Metabolism
DMR1:105456501	1	1.05E+08	6000	2	1.67E-08	66	1.1	Nell1	Development
DMR1:105858401	1	1.06E+08	500	1	6.36E-07	2	0.4	Nell1	Development
DMR1:106011801	1	1.06E+08	400	1	1.21E-08	1	0.25	Nell1	Development
DMR1:108041301	1	1.08E+08	300	1	4.50E-09	2	0.666666667		
DMR1:108763201	1	1.09E+08	800	2	2.91E-13	2	0.25		
DMR1:108918501	1	1.09E+08	500	1	9.07E-08	0	0	AABR07003398.1	
DMR1:109237501	1	1.09E+08	400	1	1.93E-07	0	0		
DMR1:109278401	1	1.09E+08	600	1	9.07E-07	0	0		
DMR1:110029001	1	1.1E+08	600	1	2.60E-07	1	0.166666667	AABR07003449.1	
DMR1:110272901	1	1.1E+08	1100	1	4.49E-07	2	0.181818182		
DMR1:110568101	1	1.11E+08	400	1	3.99E-08	4	1		
DMR1:110781601	1	1.11E+08	400	2	9.81E-09	4	1		
DMR1:110983801	1	1.11E+08	600	1	5.20E-07	8	1.333333333		
DMR1:111793801	1	1.12E+08	400	1	6.54E-07	1	0.25		
DMR1:111867001	1	1.12E+08	700	2	4.21E-11	0	0	Luzp2	
DMR1:112329301	1	1.12E+08	1200	3	3.50E-09	2	0.166666667	Luzp2;Gabrg3	Receptor
DMR1:112723401	1	1.13E+08	5300	2	7.85E-09	65	1.226415094	Luzp2;Gabrg3	Receptor
DMR1:114687501	1	1.15E+08	1200	2	4.65E-10	5	0.416666667	Oca2	Transport
DMR1:115003401	1	1.15E+08	900	2	1.51E-07	8	0.888888889		
DMR1:118484101	1	1.18E+08	300	1	7.79E-07	1	0.333333333		
DMR1:121300201	1	1.21E+08	5600	2	2.01E-12	52	0.928571429		
DMR1:123391701	1	1.23E+08	4500	3	1.23E-12	40	0.888888889		
DMR1:125447701	1	1.25E+08	400	1	7.88E-10	3	0.75	Apba2	Transport
DMR1:125602001	1	1.26E+08	1300	2	1.17E-08	12	0.923076923	Fam189a1	Unknown
DMR1:126380401	1	1.26E+08	900	1	9.63E-07	19	2.111111111	AABR07004130.1	
DMR1:127250301	1	1.27E+08	2300	1	9.66E-13	32	1.391304348	Lrrk1	Unknown

DMR1:127912601	1	1.28E+08	1100	1	9.20E-07	9	0.818181818	Adamts17	Proteolysis
DMR1:128406401	1	1.28E+08	1100	1	6.14E-07	3	0.272727273	LOC103691157	
DMR1:128826901	1	1.29E+08	1000	1	2.46E-07	16	1.6		
DMR1:128861601	1	1.29E+08	1300	1	4.26E-07	17	1.307692308		
DMR1:129691901	1	1.3E+08	700	1	1.51E-08	4	0.571428571		
DMR1:130827901	1	1.31E+08	400	1	4.02E-07	1	0.25		
DMR1:131257101	1	1.31E+08	900	1	6.26E-07	4	0.444444444		
DMR1:131325901	1	1.31E+08	200	1	3.67E-07	4	2		
DMR1:131601801	1	1.32E+08	900	1	8.68E-08	3	0.333333333		
DMR1:131794601	1	1.32E+08	1300	3	1.39E-16	6	0.461538462		
DMR1:131897501	1	1.32E+08	1400	1	5.55E-07	14	1		
DMR1:133950001	1	1.34E+08	1300	1	9.19E-08	5	0.384615385		
DMR1:135135301	1	1.35E+08	400	1	5.23E-09	2	0.5	Fam174b;Rn50_1_1362.2	
DMR1:136554701	1	1.37E+08	300	1	2.96E-08	0	0		
DMR1:136692201	1	1.37E+08	1600	2	3.82E-09	25	1.5625	Sv2b	Binding Protein
DMR1:136702501	1	1.37E+08	800	3	2.32E-10	1	0.125	Sv2b	Binding Protein
DMR1:136999001	1	1.37E+08	1900	1	6.85E-07	18	0.947368421		
DMR1:137515501	1	1.38E+08	600	1	4.57E-07	3	0.5		
DMR1:138682901	1	1.39E+08	500	3	4.97E-10	3	0.6	Agbl1	Signaling
DMR1:140669701	1	1.41E+08	500	2	1.41E-07	1	0.2		
DMR1:141296201	1	1.41E+08	600	1	3.93E-08	0	0		
DMR1:141838701	1	1.42E+08	4400	2	1.28E-07	60	1.363636364	Zfp710;Echs1;Fuom	Transcription;Metabolism
DMR1:141959701	1	1.42E+08	3900	1	4.02E-07	46	1.179487179	LOC102548889	
DMR1:142778601	1	1.43E+08	1800	4	6.26E-10	19	1.055555556		
DMR1:142898801	1	1.43E+08	2300	3	1.13E-09	22	0.956521739	Alpk3;AABR07004437.1	Cytoskeleton

DMR1:144456101	1	1.44E+08	1200	1	9.49E-09	8	0.666666667	Adamtsl3	Extracellular Matrix
DMR1:144637701	1	1.45E+08	800	1	1.73E-07	4	0.5	Efl1	
DMR1:145498101	1	1.45E+08	3200	1	6.14E-08	21	0.65625		
DMR1:145505801	1	1.46E+08	2000	1	2.17E-08	22	1.1		
DMR1:145820001	1	1.46E+08	500	1	6.76E-07	2	0.4	Il16	Growth Factors & Cytokines
DMR1:145932001	1	1.46E+08	400	1	3.17E-07	3	0.75	Cfap161	
DMR1:146783901	1	1.47E+08	1800	2	3.09E-07	7	0.388888889	Zfand6	Signaling
DMR1:146866301	1	1.47E+08	400	1	9.84E-07	0	0		
DMR1:147742201	1	1.48E+08	3500	1	9.57E-09	30	0.857142857	Cyp2c6v1	Metabolism
DMR1:147797101	1	1.48E+08	900	1	5.62E-07	5	0.555555556	Cyp2c6v1	Metabolism
DMR1:147836301	1	1.48E+08	1600	1	4.47E-07	9	0.5625	Cyp2c6v1	Metabolism
DMR1:148426701	1	1.48E+08	3900	1	7.84E-07	240	6.153846154	Vbp1	Protein Binding
DMR1:148975601	1	1.49E+08	1200	1	2.00E-07	2	0.166666667	Vom2r42	
DMR1:150011801	1	1.5E+08	700	2	8.20E-13	9	1.285714286		
DMR1:150954401	1	1.51E+08	300	1	3.53E-07	0	0	Nox4	Metabolism
DMR1:151581601	1	1.52E+08	3100	1	1.39E-07	21	0.677419355	Grm5	Receptor
DMR1:151657701	1	1.52E+08	1800	1	2.83E-09	8	0.444444444	Grm5	Receptor
DMR1:151776001	1	1.52E+08	400	1	1.27E-08	0	0	Grm5	Receptor
DMR1:152098901	1	1.52E+08	1900	1	4.57E-07	18	0.947368421	Rab38	Signaling
DMR1:152174701	1	1.52E+08	3400	1	1.20E-10	35	1.029411765		
DMR1:153319901	1	1.53E+08	800	2	9.88E-10	0	0		
DMR1:154393001	1	1.54E+08	500	2	7.89E-08	3	0.6	Picalm	Transport
DMR1:156238101	1	1.56E+08	800	1	7.24E-07	5	0.625		
DMR1:156447901	1	1.56E+08	5000	1	7.30E-07	61	1.22		
DMR1:156546701	1	1.57E+08	1100	1	6.73E-09	5	0.454545455	Dlg2	
DMR1:157188101	1	1.57E+08	300	2	1.30E-07	1	0.333333333	Dlg2	
DMR1:157513401	1	1.58E+08	1300	2	1.55E-07	6	0.461538462		

DMR1:159566101	1	1.6E+08	1700	3	1.61E-15	12	0.705882353		
DMR1:159829401	1	1.6E+08	300	2	1.05E-09	0	0		
DMR1:161237301	1	1.61E+08	1400	1	8.95E-07	5	0.357142857		
DMR1:162725101	1	1.63E+08	1400	1	8.52E-07	20	1.428571429	AC133383.1	
DMR1:164506901	1	1.65E+08	800	1	1.31E-07	5	0.625	Arrb1	Signaling
DMR1:165034501	1	1.65E+08	500	1	3.11E-07	1	0.2	Chrdl2	Signaling
DMR1:165576101	1	1.66E+08	3600	1	2.24E-08	40	1.111111111	Mrpl48	Translation
DMR1:166477601	1	1.66E+08	4300	2	1.91E-08	62	1.441860465	Arap1	Signaling
DMR1:166547101	1	1.67E+08	700	3	5.23E-09	2	0.285714286	Pde2a	Signaling
DMR1:166692301	1	1.67E+08	2300	1	4.10E-07	17	0.739130435		
DMR1:167952401	1	1.68E+08	2000	1	1.06E-08	5	0.25	Olr56;AC096030.2	
DMR1:168380601	1	1.68E+08	600	1	8.39E-07	22	3.666666667	Olr86;Olr87	Receptor
DMR1:168571401	1	1.69E+08	200	1	9.39E-07	3	1.5	Olr103	Receptor
DMR1:169363101	1	1.69E+08	3700	1	1.78E-10	62	1.675675676	RGD1310717;Olr149	
DMR1:169377101	1	1.69E+08	2500	1	7.53E-08	43	1.72	Olr149;Olr150	
DMR1:169838001	1	1.7E+08	200	2	5.32E-11	0	0	Olr179	
DMR1:170545601	1	1.71E+08	1100	4	5.12E-09	1	0.090909091	Dnhd1	
DMR1:171688201	1	1.72E+08	1000	1	3.58E-07	9	0.9	Syt9	Transport
DMR1:172427401	1	1.72E+08	500	1	6.93E-07	3	0.6		
DMR1:173047001	1	1.73E+08	1400	1	4.68E-07	19	1.357142857	LOC687119	
DMR1:173249901	1	1.73E+08	1400	1	6.36E-07	4	0.285714286	LOC499229	
DMR1:173307801	1	1.73E+08	700	1	1.90E-07	3	0.428571429		
DMR1:173326601	1	1.73E+08	900	1	8.31E-08	2	0.222222222		
DMR1:173330201	1	1.73E+08	400	1	1.29E-07	1	0.25		
DMR1:173966801	1	1.74E+08	1300	2	1.81E-10	7	0.538461538	Stk33	Signaling
DMR1:174099801	1	1.74E+08	400	1	2.11E-08	1	0.25	Trim66	
DMR1:175403201	1	1.75E+08	400	2	5.84E-13	0	0	Sbf2	Epigenetic
DMR1:175469001	1	1.75E+08	1600	1	7.75E-08	9	0.5625		

DMR1:176510101	1	1.77E+08	1400	2	1.18E-08	7	0.5	Galnt18	Unknown
DMR1:177944101	1	1.78E+08	800	1	9.37E-07	4	0.5	5S_rRNA	
DMR1:178801601	1	1.79E+08	600	2	5.35E-07	8	1.333333333	Spon1	Growth Factors & Cytokines
DMR1:179026001	1	1.79E+08	1300	1	3.85E-08	4	0.307692308		
DMR1:179103901	1	1.79E+08	1000	1	1.49E-07	12	1.2		
DMR1:179590001	1	1.8E+08	9800	1	2.71E-07	50	0.510204082		
DMR1:179684301	1	1.8E+08	700	2	1.73E-09	3	0.428571429		
DMR1:179820601	1	1.8E+08	1900	1	5.72E-09	9	0.473684211		
DMR1:179887401	1	1.8E+08	1200	1	4.56E-07	5	0.416666667		
DMR1:180411101	1	1.8E+08	1400	1	7.70E-08	17	1.214285714		
DMR1:181725701	1	1.82E+08	3900	1	1.06E-07	42	1.076923077		
DMR1:181730801	1	1.82E+08	300	1	3.78E-08	7	2.333333333		
DMR1:181769001	1	1.82E+08	900	1	6.27E-08	5	0.555555556		
DMR1:181943401	1	1.82E+08	15000	1	4.49E-09	366	2.44		
DMR1:182577401	1	1.83E+08	400	1	5.31E-09	0	0		
DMR1:182609601	1	1.83E+08	1700	1	1.47E-08	16	0.941176471		
DMR1:182733701	1	1.83E+08	1100	1	8.96E-08	7	0.636363636		
DMR1:183347301	1	1.83E+08	500	1	9.78E-08	1	0.2		
DMR1:183570501	1	1.84E+08	1900	1	3.30E-07	19	1		
DMR1:183620101	1	1.84E+08	2700	1	8.72E-07	22	0.814814815	AABR07005506.1	
DMR1:184454901	1	1.84E+08	1300	1	1.40E-07	11	0.846153846		
DMR1:185849401	1	1.86E+08	600	1	3.72E-07	6	1	Sox6	Development
DMR1:186237901	1	1.86E+08	2200	1	3.37E-08	15	0.681818182		
DMR1:188756501	1	1.89E+08	3100	2	1.50E-08	22	0.709677419		
DMR1:189065201	1	1.89E+08	1400	1	1.81E-08	6	0.428571429		
DMR1:189190401	1	1.89E+08	900	3	7.82E-09	3	0.333333333	Gp2;Umod	
DMR1:189348801	1	1.89E+08	600	1	4.58E-07	5	0.833333333	Acsm1;RNaseP_nuc;RGD1559600	Metabolism

DMR1:190986601	1	1.91E+08	400	1	6.69E-07	3	0.75	Mettl9	
DMR1:191192601	1	1.91E+08	800	1	4.20E-07	12	1.5		
DMR1:192262301	1	1.92E+08	800	1	2.00E-07	5	0.625	Prkcb	Signaling
DMR1:192349301	1	1.92E+08	4200	1	8.77E-07	55	1.30952381	Prkcb	Signaling
DMR1:192757001	1	1.93E+08	500	1	5.40E-07	4	0.8		
DMR1:192796701	1	1.93E+08	1200	1	3.32E-07	14	1.166666667		
DMR1:192871301	1	1.93E+08	2700	1	6.41E-08	23	0.851851852		
DMR1:192980601	1	1.93E+08	1000	2	1.50E-09	6	0.6		
DMR1:194567301	1	1.95E+08	5000	1	5.51E-07	25	0.5		
DMR1:196235901	1	1.96E+08	300	1	3.87E-07	0	0		
DMR1:196361201	1	1.96E+08	700	1	9.11E-08	7	1		
DMR1:196596401	1	1.97E+08	1600	2	3.18E-08	1	0.0625		
DMR1:197318501	1	1.97E+08	600	2	1.71E-08	5	0.833333333		
DMR1:197339601	1	1.97E+08	1500	1	2.09E-07	21	1.4		
DMR1:198259201	1	1.98E+08	400	2	1.33E-08	8	2	Fam57b;RGD1563217	Unknown
DMR1:198268301	1	1.98E+08	2200	1	5.46E-09	23	1.045454545	Fam57b;RGD1563217	Unknown
DMR1:198355101	1	1.98E+08	800	2	2.20E-09	5	0.625	Tmem219;Kctd13	
DMR1:199003901	1	1.99E+08	1200	1	3.21E-07	23	1.916666667	Zfp689;Srcap	Transcription
DMR1:199137901	1	1.99E+08	5000	1	7.67E-07	75	1.5		
DMR1:200547201	1	2.01E+08	1200	1	3.44E-07	8	0.666666667	AABR07005806.1	
DMR1:201317901	1	2.01E+08	2100	2	9.85E-09	27	1.285714286	Tacc2	Unknown
DMR1:202173401	1	2.02E+08	300	1	2.00E-07	0	0		
DMR1:202982101	1	2.03E+08	400	1	5.62E-07	3	0.75		
DMR1:203753501	1	2.04E+08	400	1	7.53E-07	5	1.25		
DMR1:204044201	1	2.04E+08	1300	1	1.29E-08	4	0.307692308	Cpxm2	Proteolysis
DMR1:204396401	1	2.04E+08	300	1	1.12E-07	1	0.333333333		
DMR1:204583201	1	2.05E+08	200	1	2.00E-10	2	1	Oat	Metabolism

DMR1:205926601	1	2.06E+08	600	1	6.27E-07	1	0.166666667	Fank1	
DMR1:206572301	1	2.07E+08	700	2	5.02E-10	5	0.714285714	LOC100909609	
DMR1:206961401	1	2.07E+08	400	1	1.39E-07	2	0.5	Dock1	Signaling
DMR1:207138801	1	2.07E+08	500	1	9.86E-07	0	0	Dock1;Fam196a	Signaling
DMR1:207352701	1	2.07E+08	1500	1	9.14E-09	13	0.866666667	Dock1	Signaling
DMR1:207436301	1	2.07E+08	1700	1	7.98E-07	6	0.352941176		
DMR1:207493301	1	2.07E+08	1000	2	1.22E-08	9	0.9		
DMR1:207495501	1	2.07E+08	400	2	1.52E-08	0	0		
DMR1:207592401	1	2.08E+08	1400	1	1.34E-07	79	5.642857143		
DMR1:209751001	1	2.1E+08	2600	1	6.73E-11	17	0.653846154	LOC100362342	
DMR1:209828901	1	2.1E+08	1400	1	3.71E-07	17	1.214285714		
DMR1:210027701	1	2.1E+08	900	2	6.35E-08	3	0.333333333		
DMR1:210156101	1	2.1E+08	1800	1	1.92E-08	6	0.333333333		
DMR1:210195001	1	2.1E+08	1100	2	2.04E-11	7	0.636363636		
DMR1:210948101	1	2.11E+08	200	1	8.58E-07	4	2		
DMR1:212528701	1	2.13E+08	1800	4	3.59E-12	48	2.666666667	Caly	
DMR1:212617701	1	2.13E+08	300	1	2.83E-07	4	1.333333333	Mtg1;Sprn	Signaling;Development
DMR1:213584901	1	2.14E+08	300	1	1.63E-07	2	0.666666667	RGD1309350;AC109844.1	
DMR1:215050001	1	2.15E+08	1500	1	5.45E-08	8	0.533333333	Krtap5-2	
DMR1:215093601	1	2.15E+08	300	1	2.24E-07	2	0.666666667	Krtap5-2	
DMR1:215360601	1	2.15E+08	400	2	2.45E-09	3	0.75	Krtap5-2;Gm4559	
DMR1:215375101	1	2.15E+08	300	1	7.19E-07	1	0.333333333	Krtap5-2;Gm4559	
DMR1:215466101	1	2.15E+08	1900	1	2.58E-07	24	1.263157895	LOC685544	
DMR1:215520601	1	2.16E+08	12400	1	4.70E-08	117	0.943548387	Ifitm10;Ctsd	Protease
DMR1:215633301	1	2.16E+08	1900	2	1.68E-09	5	0.263157895	Lsp1	Cytoskeleton
DMR1:216645801	1	2.17E+08	1300	1	6.12E-07	11	0.846153846		
DMR1:217893501	1	2.18E+08	3300	1	1.55E-07	25	0.757575758	Ano1	Unknown

DMR1:218031701	1	2.18E+08	1300	1	3.11E-07	10	0.769230769	Fgf4	Signaling
DMR1:218038801	1	2.18E+08	2400	1	2.70E-07	22	0.916666667		
DMR1:218368501	1	2.18E+08	600	1	2.81E-07	5	0.833333333	Rmt1	
DMR1:218812601	1	2.19E+08	1300	1	3.28E-07	6	0.461538462	Ppp6r3;Lrp5	Receptor
DMR1:219022301	1	2.19E+08	300	1	8.10E-08	0	0	Kmt5b	
DMR1:219320801	1	2.19E+08	400	1	5.91E-07	1	0.25	RGD1307603;Cabp2	Signaling
DMR1:219990101	1	2.2E+08	300	1	3.87E-08	4	1.333333333	Sptbn2	Cytoskeleton
DMR1:220858901	1	2.21E+08	1300	1	1.61E-07	23	1.769230769	Efemp2;Mus81;Cfl1	Signaling;Transcription;Cytoskeleton
DMR1:221778301	1	2.22E+08	3100	3	3.18E-09	37	1.193548387	Pygm;Rasgrp2	Signaling
DMR1:221799401	1	2.22E+08	900	1	7.74E-07	12	1.333333333	Nrxn2	Receptor
DMR1:221942401	1	2.22E+08	500	3	3.10E-11	0	0		
DMR1:222057301	1	2.22E+08	2700	1	2.40E-08	25	0.925925926		
DMR1:222240301	1	2.22E+08	1000	1	4.11E-07	8	0.8	Ppp1r14b;Fkbp2;Vegfb;Dnajc4;Nudt22;Trpt1	Signaling;Metabolism;Protein Binding;Translation
DMR1:223740901	1	2.24E+08	1700	1	2.96E-10	22	1.294117647		
DMR1:224036601	1	2.24E+08	900	3	3.60E-08	11	1.222222222		
DMR1:224090501	1	2.24E+08	700	2	1.34E-08	3	0.428571429		
DMR1:224211401	1	2.24E+08	2400	1	4.22E-07	23	0.958333333		
DMR1:224410101	1	2.24E+08	400	1	1.29E-07	1	0.25		
DMR1:225219001	1	2.25E+08	1100	1	4.77E-07	11	1	Ahnak	Cytoskeleton
DMR1:225258101	1	2.25E+08	800	2	2.70E-08	7	0.875		
DMR1:225694801	1	2.26E+08	600	1	3.94E-07	1	0.166666667	Psbpc2	
DMR1:226123901	1	2.26E+08	400	1	1.29E-07	2	0.5	Fads2	
DMR1:226169801	1	2.26E+08	1600	1	2.62E-07	17	1.0625		
DMR1:226415001	1	2.26E+08	1000	1	1.21E-08	4	0.4		

DMR1:226422101	1	2.26E+08	3000	2	5.98E-08	30	1		
DMR1:226582901	1	2.27E+08	1500	1	3.02E-07	16	1.066666667	Cpsf7	Translation
DMR1:226699901	1	2.27E+08	700	1	4.55E-09	0	0	Vwce	Development
DMR1:228869701	1	2.29E+08	1400	1	7.86E-07	12	0.857142857		
DMR1:229473401	1	2.29E+08	2200	2	2.76E-08	12	0.545454545	Olr387	
DMR1:231282801	1	2.31E+08	1500	1	4.48E-09	11	0.733333333		
DMR1:232223901	1	2.32E+08	400	1	8.43E-07	0	0		
DMR1:233203901	1	2.33E+08	6100	2	1.54E-07	74	1.213114754	Cep78	
DMR1:233563701	1	2.34E+08	500	1	9.49E-07	2	0.4	Gnaq	Signaling
DMR1:234215401	1	2.34E+08	300	1	5.24E-07	0	0		
DMR1:234505601	1	2.35E+08	2000	1	4.44E-07	24	1.2	Trpm6	Receptor
DMR1:235308501	1	2.35E+08	1500	2	1.53E-08	3	0.2	Gna14	Signaling
DMR1:237061301	1	2.37E+08	100	1	1.23E-08	0	0	Foxb2	
DMR1:238946001	1	2.39E+08	1600	1	1.22E-08	14	0.875		
DMR1:239067501	1	2.39E+08	1200	1	9.99E-08	9	0.75	Gda	Metabolism
DMR1:239153001	1	2.39E+08	1000	1	2.51E-08	8	0.8	Rn50_1_2465.1	
DMR1:239376201	1	2.39E+08	2900	1	7.30E-10	27	0.931034483	AABR07006544.1	
DMR1:240833401	1	2.41E+08	400	2	9.20E-13	0	0		
DMR1:240888001	1	2.41E+08	1500	1	4.75E-07	9	0.6		
DMR1:241003701	1	2.41E+08	1000	1	1.05E-07	5	0.5	Smc5	Transcription
DMR1:241641501	1	2.42E+08	300	1	1.94E-07	1	0.333333333	Apba1	Development
DMR1:242475501	1	2.42E+08	5000	1	6.88E-11	33	0.66		
DMR1:243739801	1	2.44E+08	600	1	8.29E-07	2	0.333333333		
DMR1:244500601	1	2.45E+08	400	2	3.91E-07	1	0.25	AABR07006654.1	
DMR1:244578301	1	2.45E+08	500	1	4.77E-11	2	0.4		
DMR1:244595501	1	2.45E+08	4200	1	9.58E-07	38	0.904761905		
DMR1:244849901	1	2.45E+08	300	1	7.82E-07	2	0.666666667		
DMR1:244955701	1	2.45E+08	1000	4	4.88E-10	1	0.1		
DMR1:245177701	1	2.45E+08	1200	2	1.05E-07	8	0.666666667		

DMR1:245347701	1	2.45E+08	3100	1	7.88E-08	22	0.709677419		
DMR1:247196801	1	2.47E+08	4800	1	3.27E-07	101	2.104166667		
DMR1:247543401	1	2.48E+08	2700	2	5.23E-09	28	1.037037037	Cd274	Immune
DMR1:247657301	1	2.48E+08	2100	2	9.04E-11	36	1.714285714		
DMR1:248381601	1	2.48E+08	1500	2	1.24E-08	7	0.466666667	Gldc	Metabolism
DMR1:249007501	1	2.49E+08	300	1	3.32E-07	4	1.333333333	AABR07006713.1	
DMR1:249014401	1	2.49E+08	1700	1	2.72E-08	18	1.058823529	AABR07006713.1	
DMR1:249351901	1	2.49E+08	1700	4	6.15E-11	7	0.411764706		
DMR1:250129701	1	2.5E+08	200	1	7.43E-07	1	0.5	AABR07006724.1	
DMR1:250896801	1	2.51E+08	900	1	3.69E-07	6	0.666666667	Sgms1	Metabolism
DMR1:251515001	1	2.52E+08	500	1	1.90E-10	0	0		
DMR1:251546001	1	2.52E+08	1200	1	7.80E-08	13	1.083333333		
DMR1:251736901	1	2.52E+08	800	1	4.68E-07	3	0.375		
DMR1:252464801	1	2.52E+08	300	1	6.65E-07	0	0	Ankrd22	Transcription
DMR1:253076701	1	2.53E+08	2100	1	8.39E-07	21	1		
DMR1:253172301	1	2.53E+08	300	1	9.97E-07	1	0.333333333	Pank1	Signaling
DMR1:253224101	1	2.53E+08	1500	2	2.62E-09	10	0.666666667	Kif20b	Cytoskeleton
DMR1:253495101	1	2.53E+08	300	1	6.67E-07	3	1		
DMR1:254018301	1	2.54E+08	1700	2	3.01E-07	24	1.411764706		
DMR1:254797101	1	2.55E+08	600	1	6.39E-08	0	0		
DMR1:255731401	1	2.56E+08	1600	1	9.19E-08	1	0.0625	Cpeb3	MicroRNA
DMR1:256004701	1	2.56E+08	1200	1	3.02E-07	5	0.416666667	Ide	Protease
DMR1:256360901	1	2.56E+08	700	1	5.75E-07	2	0.285714286	Exoc6;Cyp26c1	Transport;Metabolism
DMR1:256444101	1	2.56E+08	800	2	7.49E-11	7	0.875	AC096353.1	
DMR1:256535301	1	2.57E+08	800	3	2.31E-09	1	0.125		
DMR1:256749101	1	2.57E+08	2400	1	9.08E-07	19	0.791666667	Cep55	
DMR1:258905201	1	2.59E+08	1100	1	5.97E-07	8	0.727272727		
DMR1:259696401	1	2.6E+08	1100	1	6.53E-07	5	0.454545455	Tctn3	Unknown

DMR1:260358201	1	2.6E+08	1400	2	4.87E-10	20	1.428571429	Tll2	Protease
DMR1:260584201	1	2.61E+08	1000	1	3.37E-08	1	0.1	Pik3ap1	
DMR1:261880101	1	2.62E+08	4900	2	5.91E-08	70	1.428571429		
DMR1:261925401	1	2.62E+08	500	3	6.96E-09	3	0.6	AC096317.1	
DMR1:262100801	1	2.62E+08	1800	1	1.89E-08	19	1.055555556		
DMR1:262118501	1	2.62E+08	2000	1	4.59E-07	30	1.5		
DMR1:262775401	1	2.63E+08	3900	1	1.78E-07	39	1		
DMR1:263164701	1	2.63E+08	1900	2	2.65E-09	24	1.263157895		
DMR1:263342301	1	2.63E+08	400	1	1.05E-08	0	0		
DMR1:263601601	1	2.64E+08	1400	1	1.81E-07	25	1.785714286	Abcc2	Transport
DMR1:264414701	1	2.64E+08	3800	1	2.03E-07	123	3.236842105		
DMR1:264932101	1	2.65E+08	400	2	4.22E-10	1	0.25	AC105485.1	
DMR1:265802101	1	2.66E+08	2200	1	8.65E-07	19	0.863636364	Ldb1;Pprc1	Transcription;Signaling
DMR1:266443201	1	2.66E+08	1100	1	1.82E-07	11	1	Borcs7	
DMR1:266458301	1	2.66E+08	2000	1	9.79E-07	25	1.25	Borcs7	
DMR1:266885801	1	2.67E+08	1200	2	1.29E-08	2	0.166666667	Pdcd11	Apoptosis
DMR1:266938701	1	2.67E+08	1400	2	2.50E-07	13	0.928571429	Calhm3	
DMR1:267332501	1	2.67E+08	400	1	4.70E-07	1	0.25		
DMR1:267345101	1	2.67E+08	500	1	7.88E-08	9	1.8		
DMR1:268523301	1	2.69E+08	1300	2	1.04E-08	11	0.846153846	Sorcs3	Receptor
DMR1:269529601	1	2.7E+08	500	1	8.37E-09	0	0		
DMR1:271117401	1	2.71E+08	1200	1	4.83E-07	2	0.166666667		
DMR1:272298501	1	2.72E+08	2000	1	6.55E-07	14	0.7		
DMR1:272509701	1	2.73E+08	500	1	6.04E-07	3	0.6		
DMR1:273029601	1	2.73E+08	300	1	3.38E-07	1	0.333333333		
DMR1:273450001	1	2.73E+08	900	1	4.47E-07	10	1.111111111		
DMR1:273628301	1	2.74E+08	3400	3	3.77E-08	23	0.676470588		
DMR1:274616801	1	2.75E+08	1700	3	2.57E-09	10	0.588235294	Pdcd4	Transcription

DMR1:274981201	1	2.75E+08	500	1	9.25E-07	0	0		
DMR1:275007801	1	2.75E+08	1200	2	2.08E-08	3	0.25	AABR07006978.1	
DMR1:275355901	1	2.75E+08	100	1	6.19E-18	0	0		
DMR1:275512601	1	2.76E+08	1700	2	8.96E-09	3	0.176470588		
DMR1:275939801	1	2.76E+08	2400	3	1.17E-08	8	0.333333333		
DMR1:276052001	1	2.76E+08	600	2	2.15E-14	1	0.166666667		
DMR1:276189701	1	2.76E+08	1700	1	3.56E-08	25	1.470588235	Gucy2g	
DMR1:277100001	1	2.77E+08	600	2	4.25E-08	2	0.333333333	Habp2;Nrap	Protease;Cytoskeleton
DMR1:277452701	1	2.77E+08	1400	1	9.85E-08	25	1.785714286	AABR07007023.1	
DMR1:277884001	1	2.78E+08	1700	3	1.14E-10	17	1	Ablim1	Cytoskeleton
DMR1:278006901	1	2.78E+08	3500	1	3.02E-08	66	1.885714286	Ablim1	Cytoskeleton
DMR1:278210101	1	2.78E+08	500	2	2.57E-07	3	0.6	Fam160b1	Unknown
DMR1:278245001	1	2.78E+08	1800	3	1.39E-18	23	1.277777778		
DMR1:278449701	1	2.78E+08	2400	5	5.04E-19	18	0.75		
DMR1:278456701	1	2.78E+08	3000	3	9.42E-14	37	1.233333333		
DMR1:278725801	1	2.79E+08	1700	2	9.57E-15	7	0.411764706	Atrnl1	Signaling
DMR1:278822601	1	2.79E+08	1600	1	6.08E-08	13	0.8125	Atrnl1	Signaling
DMR1:278891501	1	2.79E+08	500	1	1.78E-09	2	0.4		
DMR1:279247201	1	2.79E+08	1000	1	4.47E-07	14	1.4	Gfra1	Receptor
DMR1:279303901	1	2.79E+08	300	1	4.15E-07	6	2		
DMR1:279652401	1	2.8E+08	800	1	5.62E-07	39	4.875	Ccdc172	
DMR1:279748301	1	2.8E+08	200	1	8.14E-07	3	1.5		
DMR1:279840901	1	2.8E+08	400	2	3.40E-14	7	1.75		
DMR1:279920201	1	2.8E+08	1400	2	4.03E-08	10	0.714285714	Pnliprp2	Unknown
DMR1:280030601	1	2.8E+08	1500	2	4.00E-09	9	0.6		
DMR1:280309501	1	2.8E+08	800	1	7.31E-07	6	0.75		
DMR1:280922701	1	2.81E+08	4200	1	6.07E-07	56	1.333333333		
DMR1:281700001	1	2.82E+08	1200	4	3.42E-16	5	0.416666667		

DMR1:282037301	1	2.82E+08	2500	1	2.30E-10	13	0.52		
DMR1:282439701	1	2.82E+08	1500	3	9.72E-11	6	0.4	Grk5	Signaling
DMR2:451901	2	451901	1800	1	4.47E-07	12	0.666666667		
DMR2:1484901	2	1484901	1300	1	3.37E-11	13	1	Cast	Signaling
DMR2:2371601	2	2371601	500	1	8.18E-07	2	0.4		
DMR2:3871801	2	3871801	300	1	8.35E-09	2	0.666666667	Mctp1	Unknown
DMR2:4241001	2	4241001	600	5	2.78E-14	0	0	RGD1560883	Unknown
DMR2:4440201	2	4440201	200	1	1.19E-07	0	0	RGD1560883	Unknown
DMR2:5895101	2	5895101	1500	2	3.86E-11	7	0.466666667		
DMR2:6575201	2	6575201	1900	4	6.02E-08	3	0.157894737		
DMR2:6802001	2	6802001	400	1	1.28E-08	1	0.25		
DMR2:7298701	2	7298701	600	2	3.37E-08	6	1		
DMR2:8942201	2	8942201	1200	2	2.83E-07	3	0.25	Adgrv1	
DMR2:9254001	2	9254001	400	1	1.93E-10	3	0.75	Adgrv1	
DMR2:9879101	2	9879101	1600	1	1.26E-08	7	0.4375		
DMR2:11180301	2	11180301	300	1	1.92E-07	1	0.333333333		
DMR2:11316001	2	11316001	800	3	5.70E-10	1	0.125		
DMR2:11416201	2	11416201	5000	1	2.98E-07	95	1.9		
DMR2:11925201	2	11925201	200	1	4.09E-07	2	1		
DMR2:13362901	2	13362901	4000	1	9.36E-07	38	0.95		
DMR2:13812601	2	13812601	1600	2	6.38E-09	10	0.625		
DMR2:15489101	2	15489101	200	1	6.58E-07	3	1.5		
DMR2:16163401	2	16163401	1200	2	1.41E-08	8	0.666666667		
DMR2:16977001	2	16977001	600	1	4.93E-08	5	0.833333333		
DMR2:17095301	2	17095301	300	1	2.70E-07	1	0.333333333		
DMR2:18597601	2	18597601	800	2	5.25E-09	3	0.375		
DMR2:19267201	2	19267201	2400	1	1.97E-08	44	1.833333333		
DMR2:20633201	2	20633201	1200	1	9.43E-07	7	0.583333333	Ssbp2	Transcription
DMR2:21074801	2	21074801	1500	1	7.85E-08	13	0.866666667		

DMR2:21898801	2	21898801	2100	1	7.89E-07	10	0.476190476	Msh3	DNA Repair
DMR2:22040301	2	22040301	2600	1	4.70E-07	79	3.038461538	Fam151b	
DMR2:23281201	2	23281201	2600	1	6.00E-09	26	1	Bhmt2;Dmgdh	Metabolism
DMR2:23577901	2	23577901	1100	2	3.51E-08	9	0.818181818		
DMR2:24286401	2	24286401	1100	1	8.50E-07	7	0.636363636		
DMR2:25266101	2	25266101	2200	1	6.40E-09	24	1.090909091		
DMR2:25602301	2	25602301	900	1	1.78E-07	10	1.111111111		
DMR2:25749801	2	25749801	500	1	3.25E-07	1	0.2	Arhgef28;SNORA40	Signaling
DMR2:26273401	2	26273401	300	1	2.50E-07	5	1.666666667	Iqgap2	Signaling
DMR2:27225001	2	27225001	800	1	2.32E-07	5	0.625	Arhgef26	
DMR2:28979401	2	28979401	3400	2	2.88E-11	27	0.794117647	Fcho2	Cell Cycle
DMR2:29468601	2	29468601	500	1	1.92E-07	3	0.6	Zfp366	Transcription
DMR2:30973201	2	30973201	600	3	1.18E-11	0	0		
DMR2:31467001	2	31467001	1500	3	4.32E-10	10	0.666666667		
DMR2:31814401	2	31814401	600	1	3.82E-08	6	1	Pik3r1	Signaling
DMR2:32088401	2	32088401	1400	1	9.20E-07	10	0.714285714		
DMR2:32119101	2	32119101	3300	2	8.67E-10	25	0.757575758		
DMR2:32553701	2	32553701	1500	1	7.17E-08	7	0.466666667	AABR07007878.1	
DMR2:33381401	2	33381401	1100	1	6.81E-07	9	0.818181818		
DMR2:33437801	2	33437801	400	1	8.96E-07	0	0		
DMR2:34699501	2	34699501	2300	1	3.74E-09	40	1.739130435		
DMR2:34747901	2	34747901	200	1	6.92E-08	1	0.5	Cwc27	Metabolism
DMR2:34825101	2	34825101	400	1	2.53E-08	2	0.5	Cwc27	Metabolism
DMR2:35085201	2	35085201	1600	1	9.74E-07	32	2	Rgs7bp	
DMR2:36723401	2	36723401	400	1	3.30E-09	1	0.25		
DMR2:37362101	2	37362101	600	1	6.55E-07	0	0		
DMR2:38874301	2	38874301	1600	1	2.12E-07	16	1		
DMR2:39172201	2	39172201	2500	1	4.37E-07	13	0.52		
DMR2:39268001	2	39268001	300	1	8.22E-07	0	0		

DMR2:40012801	2	40012801	1800	3	8.30E-13	15	0.833333333	Depdc1b	Transcription
DMR2:40963901	2	40963901	1300	1	5.04E-07	4	0.307692308	Pde4d	Metabolism
DMR2:42171501	2	42171501	700	1	2.76E-08	5	0.714285714		
DMR2:43161001	2	43161001	1200	1	1.50E-08	12	1		
DMR2:44777901	2	44777901	800	2	2.75E-12	7	0.875	Skiv2l2;Dhx29	Transcription
DMR2:47492601	2	47492601	600	1	5.07E-07	7	1.166666667		
DMR2:49437401	2	49437401	3500	1	4.71E-08	50	1.428571429		
DMR2:50245101	2	50245101	500	1	2.04E-10	2	0.4	Hcn1	Signaling
DMR2:51034001	2	51034001	900	2	4.89E-09	7	0.777777778	Mrps30	
DMR2:51121701	2	51121701	600	2	1.77E-07	3	0.5		
DMR2:51657301	2	51657301	800	1	1.45E-08	3	0.375		
DMR2:52097401	2	52097401	600	1	2.51E-07	4	0.666666667		
DMR2:52274601	2	52274601	2300	4	2.12E-08	21	0.913043478	Nnt	Metabolism
DMR2:52487201	2	52487201	2600	2	1.02E-08	24	0.923076923	Nim1k	
DMR2:52928601	2	52928601	600	1	5.64E-07	5	0.833333333		
DMR2:52970401	2	52970401	400	1	4.51E-08	9	2.25		
DMR2:54200601	2	54200601	400	1	2.36E-08	2	0.5	Plcxd3	
DMR2:54590401	2	54590401	300	1	1.77E-12	2	0.666666667		
DMR2:54785401	2	54785401	1300	1	1.77E-07	23	1.769230769	C7	Immune
DMR2:55612501	2	55612501	1900	1	1.87E-07	18	0.947368421		
DMR2:56276201	2	56276201	400	1	2.31E-07	2	0.5		
DMR2:56749801	2	56749801	1200	2	1.66E-09	2	0.166666667		
DMR2:56982201	2	56982201	2600	1	4.29E-07	23	0.884615385	Wdr70	Unknown
DMR2:57279101	2	57279101	900	3	1.57E-12	1	0.111111111	RGD1310081	
DMR2:57492601	2	57492601	700	4	1.37E-15	4	0.571428571		
DMR2:58384501	2	58384501	1300	1	1.14E-07	11	0.846153846		
DMR2:59999501	2	59999501	1100	1	2.03E-09	15	1.363636364	7SK	
DMR2:60674001	2	60674001	4600	1	6.75E-07	78	1.695652174	Rai14	Transcription
DMR2:60791401	2	60791401	400	1	8.45E-07	0	0		

DMR2:60950601	2	60950601	500	1	1.19E-08	2	0.4	C1qtnf3;Amacr	Metabolism
DMR2:61193201	2	61193201	1700	1	4.18E-08	15	0.882352941	Adamts12	Protease
DMR2:61298401	2	61298401	1300	2	3.38E-08	10	0.769230769	Adamts12	Protease
DMR2:61592001	2	61592001	1100	2	2.02E-07	11	1		
DMR2:63145801	2	63145801	5800	2	6.78E-09	53	0.913793103	Cdh6	Cytoskeleton
DMR2:65039001	2	65039001	400	1	3.41E-07	1	0.25		
DMR2:67833201	2	67833201	300	1	6.88E-08	0	0		
DMR2:69282401	2	69282401	2400	1	3.57E-09	10	0.416666667		
DMR2:69369501	2	69369501	2200	1	5.72E-07	20	0.909090909		
DMR2:69735901	2	69735901	4300	1	2.18E-07	51	1.186046512	AABR07008670.1	
DMR2:69741701	2	69741701	600	3	3.17E-11	1	0.166666667	AABR07008670.1	
DMR2:71987701	2	71987701	300	1	7.06E-07	1	0.333333333		
DMR2:73224201	2	73224201	400	1	8.91E-08	4	1		
DMR2:73379101	2	73379101	1400	1	7.70E-07	8	0.571428571		
DMR2:77669101	2	77669101	1300	1	2.60E-07	8	0.615384615	AABR07008898.1	
DMR2:79282101	2	79282101	300	1	6.23E-07	2	0.666666667		
DMR2:80452601	2	80452601	400	1	3.10E-07	4	1		
DMR2:80909901	2	80909901	600	1	8.75E-07	3	0.5		
DMR2:81542801	2	81542801	400	1	2.75E-07	2	0.5		
DMR2:81685701	2	81685701	1400	1	4.48E-08	19	1.357142857		
DMR2:82183301	2	82183301	1200	1	1.14E-08	5	0.416666667		
DMR2:82448701	2	82448701	1300	1	7.78E-07	8	0.615384615		
DMR2:82501901	2	82501901	1500	1	1.37E-09	9	0.6	AABR07008991.1	
DMR2:83089101	2	83089101	600	1	2.79E-07	6	1		
DMR2:84171801	2	84171801	1000	3	6.46E-11	3	0.3		
DMR2:85688001	2	85688001	300	1	8.78E-07	2	0.666666667	Sema5a	Signaling
DMR2:85783101	2	85783101	400	1	4.92E-08	2	0.5	Sema5a	Signaling
DMR2:87356301	2	87356301	500	1	7.95E-08	0	0		
DMR2:88138901	2	88138901	400	2	1.08E-08	0	0	Car3	Metabolism

DMR2:89360501	2	89360501	1100	1	5.75E-07	9	0.818181818		
DMR2:89542201	2	89542201	500	3	1.05E-11	1	0.2		
DMR2:90064001	2	90064001	2000	1	8.56E-07	6	0.3		
DMR2:92017901	2	92017901	300	1	2.73E-07	0	0		
DMR2:93471401	2	93471401	1300	1	3.68E-10	7	0.538461538	AABR07009338.1	
DMR2:93801901	2	93801901	900	1	4.33E-07	7	0.777777778	Fabp4;Fabp9;U6	Binding Protein;Receptor
DMR2:94780301	2	94780301	800	1	5.92E-08	13	1.625		
DMR2:95212001	2	95212001	1300	1	5.48E-08	46	3.538461538		
DMR2:95702801	2	95702801	600	1	4.49E-09	2	0.333333333		
DMR2:95872001	2	95872001	1600	3	5.48E-13	3	0.1875		
DMR2:97264401	2	97264401	1400	1	3.93E-07	4	0.285714286	LOC108351601	
DMR2:98862201	2	98862201	500	1	5.13E-11	1	0.2		
DMR2:100549601	2	1.01E+08	200	1	6.28E-07	0	0		
DMR2:101537601	2	1.02E+08	2100	2	3.69E-10	8	0.380952381		
DMR2:101568701	2	1.02E+08	5900	2	8.35E-09	80	1.355932203		
DMR2:103276501	2	1.03E+08	800	1	1.97E-07	3	0.375		
DMR2:104239101	2	1.04E+08	400	1	5.15E-08	2	0.5		
DMR2:104391301	2	1.04E+08	2200	1	3.59E-07	17	0.772727273	Dnajc5b	Protein Binding
DMR2:104707301	2	1.05E+08	1000	5	6.51E-09	1	0.1		
DMR2:104833301	2	1.05E+08	400	1	2.94E-08	0	0	Hps3	Development
DMR2:104835201	2	1.05E+08	4300	1	2.10E-07	16	0.372093023	Hps3	Development
DMR2:105022401	2	1.05E+08	2700	2	2.62E-08	24	0.888888889	Cpa3	Proteolysis
DMR2:105064901	2	1.05E+08	1900	1	4.04E-07	11	0.578947368	Cpb1	Protease
DMR2:106836301	2	1.07E+08	600	1	2.78E-09	2	0.333333333		
DMR2:108694201	2	1.09E+08	1600	1	6.41E-07	6	0.375		
DMR2:108807601	2	1.09E+08	2400	2	3.67E-12	21	0.875		
DMR2:109397801	2	1.09E+08	1200	1	8.23E-08	7	0.583333333		

DMR2:110307101	2	1.1E+08	700	1	3.26E-07	4	0.571428571	LOC499584	
DMR2:111763601	2	1.12E+08	700	3	8.29E-08	1	0.142857143	Nlgn1	Signaling
DMR2:111807901	2	1.12E+08	300	1	9.08E-07	0	0		
DMR2:112488501	2	1.12E+08	500	2	6.79E-10	2	0.4	Spata16	
DMR2:112710201	2	1.13E+08	2500	1	9.95E-07	20	0.8		
DMR2:113008801	2	1.13E+08	1200	1	1.47E-09	6	0.5	Tnfsf10	Apoptosis
DMR2:113191601	2	1.13E+08	300	1	7.89E-07	7	2.333333333	Fndc3b	Cytoskeleton
DMR2:113430101	2	1.13E+08	800	1	5.64E-07	14	1.75		
DMR2:113476501	2	1.13E+08	800	1	3.32E-08	1	0.125		
DMR2:114342701	2	1.14E+08	2000	1	6.49E-07	12	0.6	Tnik	Signaling
DMR2:114869001	2	1.15E+08	1300	1	1.76E-08	8	0.615384615		
DMR2:115541101	2	1.16E+08	500	1	5.09E-07	16	3.2		
DMR2:116773301	2	1.17E+08	200	1	3.35E-07	1	0.5		
DMR2:117975701	2	1.18E+08	1700	1	6.61E-07	19	1.117647059		
DMR2:118678501	2	1.19E+08	1000	1	1.79E-08	4	0.4		
DMR2:118886101	2	1.19E+08	2400	1	8.44E-07	38	1.583333333	Kcnmb3	Transport
DMR2:119015401	2	1.19E+08	200	1	1.01E-07	5	2.5	Gnb4	Signaling
DMR2:119030301	2	1.19E+08	700	1	6.41E-07	6	0.857142857		
DMR2:119298501	2	1.19E+08	500	1	9.13E-10	4	0.8	Usp13	Protease
DMR2:120368901	2	1.2E+08	800	2	5.76E-13	5	0.625		
DMR2:121071701	2	1.21E+08	2900	3	7.40E-11	26	0.896551724		
DMR2:121990701	2	1.22E+08	400	1	2.75E-07	4	1		
DMR2:122944201	2	1.23E+08	3900	1	1.05E-08	44	1.128205128	Qrfpr	Receptor
DMR2:123118301	2	1.23E+08	400	1	4.42E-08	2	0.5		
DMR2:123176001	2	1.23E+08	1200	2	1.01E-09	10	0.833333333	Anxa5	Signaling
DMR2:123815601	2	1.24E+08	2900	1	1.25E-07	22	0.75862069	Adad1	Transcription

DMR2:124132201	2	1.24E+08	1000	1	3.59E-07	3	0.3	Fgf2;Nudt6	Growth Factors & Cytokines;Signaling
DMR2:124573701	2	1.25E+08	300	1	7.57E-07	4	1.333333333		
DMR2:125930701	2	1.26E+08	500	1	4.28E-07	4	0.8		
DMR2:126003601	2	1.26E+08	3000	1	4.62E-07	27	0.9		
DMR2:126340801	2	1.26E+08	400	1	2.55E-07	1	0.25		
DMR2:126843201	2	1.27E+08	500	3	1.02E-10	1	0.2		
DMR2:128017201	2	1.28E+08	1300	2	1.42E-09	8	0.615384615		
DMR2:128384801	2	1.28E+08	500	1	5.02E-08	7	1.4	AABR07010183.1	
DMR2:128772201	2	1.29E+08	5200	1	3.72E-07	176	3.384615385		
DMR2:129040901	2	1.29E+08	700	1	7.23E-07	1	0.142857143		
DMR2:131158601	2	1.31E+08	1500	4	1.04E-12	3	0.2		
DMR2:132074601	2	1.32E+08	1200	1	5.86E-07	2	0.166666667		
DMR2:132158901	2	1.32E+08	500	1	9.02E-09	4	0.8		
DMR2:132196201	2	1.32E+08	2000	1	1.41E-08	10	0.5		
DMR2:132598201	2	1.33E+08	100	1	3.63E-07	0	0		
DMR2:132842001	2	1.33E+08	600	2	5.46E-07	1	0.166666667	AABR07010342.1	
DMR2:134450201	2	1.34E+08	300	1	1.59E-07	1	0.333333333		
DMR2:134701701	2	1.35E+08	300	1	1.27E-08	0	0		
DMR2:134799801	2	1.35E+08	3600	1	3.29E-09	36	1		
DMR2:135901401	2	1.36E+08	1700	1	3.37E-08	11	0.647058824		
DMR2:138103001	2	1.38E+08	2400	3	1.82E-09	23	0.958333333		
DMR2:139090401	2	1.39E+08	600	1	7.43E-07	5	0.833333333		
DMR2:139154701	2	1.39E+08	300	1	1.37E-07	3	1		
DMR2:139264701	2	1.39E+08	300	1	6.26E-07	1	0.333333333		
DMR2:141727801	2	1.42E+08	900	1	9.37E-07	10	1.111111111		
DMR2:141949901	2	1.42E+08	1000	2	3.17E-07	10	1		

DMR2:142113601	2	1.42E+08	400	1	1.14E-07	5	1.25		
DMR2:142336801	2	1.42E+08	700	1	2.46E-07	1	0.142857143	Lhfp	Transcription
DMR2:142471501	2	1.42E+08	1400	1	7.60E-07	6	0.428571429	Lhfp	Transcription
DMR2:143009701	2	1.43E+08	2500	1	2.62E-09	23	0.92		
DMR2:143395201	2	1.43E+08	400	1	1.52E-07	5	1.25	AABR07010609.1	
DMR2:144081901	2	1.44E+08	400	1	1.37E-07	3	0.75		
DMR2:145932901	2	1.46E+08	1500	2	3.06E-09	6	0.4	AABR07010649.1	
DMR2:146285501	2	1.46E+08	1300	1	4.11E-07	5	0.384615385		
DMR2:146453801	2	1.46E+08	500	2	4.12E-09	0	0		
DMR2:146670701	2	1.47E+08	4500	2	1.05E-07	41	0.911111111		
DMR2:146741701	2	1.47E+08	700	1	1.14E-07	1	0.142857143		
DMR2:147258601	2	1.47E+08	4600	1	5.35E-07	33	0.717391304		
DMR2:147269501	2	1.47E+08	800	1	6.68E-07	6	0.75		
DMR2:147406501	2	1.47E+08	200	1	3.28E-07	1	0.5		
DMR2:147600701	2	1.48E+08	700	1	6.10E-07	3	0.428571429	Wwtr1	Transcription
DMR2:147844301	2	1.48E+08	1000	1	1.99E-07	6	0.6	Rnf13	
DMR2:148000201	2	1.48E+08	600	1	3.38E-07	5	0.833333333	Rnf13;LOC100909840	Cytoskeleton
DMR2:148160601	2	1.48E+08	1400	2	6.73E-09	13	0.928571429		
DMR2:148491501	2	1.48E+08	300	1	7.18E-07	0	0		
DMR2:148810401	2	1.49E+08	400	1	1.24E-11	0	0	AC112531.1;AC112531.2;Erich6	
DMR2:149053001	2	1.49E+08	400	1	3.34E-09	0	0	Clrn1	
DMR2:149525101	2	1.5E+08	600	1	2.42E-07	7	1.166666667	Med12l;Igsf10	Transcription;Immune
DMR2:149577001	2	1.5E+08	800	1	1.36E-08	2	0.25		
DMR2:151436901	2	1.51E+08	2100	2	2.04E-07	37	1.761904762	7SK	
DMR2:152324601	2	1.52E+08	400	2	2.21E-07	0	0		
DMR2:153512301	2	1.54E+08	1700	1	2.04E-07	25	1.470588235		

DMR2:153572601	2	1.54E+08	2600	1	4.33E-08	29	1.115384615		
DMR2:153975801	2	1.54E+08	1200	1	2.69E-08	6	0.5		
DMR2:155783501	2	1.56E+08	2800	1	6.05E-07	13	0.464285714	Kcnab1	Transport
DMR2:155982301	2	1.56E+08	1200	1	4.68E-07	7	0.583333333	Kcnab1	Transport
DMR2:157565901	2	1.58E+08	700	1	9.51E-08	0	0	Lekr1	
DMR2:158909801	2	1.59E+08	1000	1	8.62E-08	9	0.9		
DMR2:159003601	2	1.59E+08	300	1	1.17E-07	10	3.333333333		
DMR2:159009301	2	1.59E+08	2300	3	1.22E-10	60	2.608695652		
DMR2:159138401	2	1.59E+08	1800	1	9.15E-07	24	1.333333333	AABR07011152.1	
DMR2:159163801	2	1.59E+08	700	1	9.73E-08	12	1.714285714		
DMR2:159267201	2	1.59E+08	800	1	2.79E-07	4	0.5		
DMR2:159981801	2	1.6E+08	1200	1	6.77E-08	13	1.083333333		
DMR2:161899501	2	1.62E+08	5000	1	3.32E-07	85	1.7		
DMR2:162150401	2	1.62E+08	1700	1	3.39E-09	20	1.176470588		
DMR2:162411301	2	1.62E+08	7600	1	1.61E-07	140	1.842105263		
DMR2:162780101	2	1.63E+08	400	1	3.81E-08	0	0		
DMR2:163095801	2	1.63E+08	700	1	5.75E-07	6	0.857142857		
DMR2:163325001	2	1.63E+08	3900	2	7.40E-09	35	0.897435897	AABR07011545.1	
DMR2:163606501	2	1.64E+08	700	1	8.87E-07	9	1.285714286		
DMR2:165166401	2	1.65E+08	2900	1	5.42E-07	51	1.75862069		
DMR2:165215101	2	1.65E+08	1700	1	2.83E-07	10	0.588235294		
DMR2:165895601	2	1.66E+08	1900	1	6.62E-08	15	0.789473684		
DMR2:167480301	2	1.67E+08	300	1	1.44E-07	2	0.666666667		
DMR2:168006301	2	1.68E+08	400	1	2.52E-07	1	0.25	AABR07011746.1	
DMR2:168814601	2	1.69E+08	500	1	1.48E-07	0	0		
DMR2:169428101	2	1.69E+08	2100	2	2.27E-07	27	1.285714286		
DMR2:169697501	2	1.7E+08	2100	1	2.01E-07	8	0.380952381		
DMR2:173702201	2	1.74E+08	1800	1	2.79E-07	8	0.444444444	Wdr49	
DMR2:174100301	2	1.74E+08	300	1	1.27E-08	0	0	Serpini1	Proteolysis

DMR2:175283801	2	1.75E+08	1900	1	5.71E-07	19	1		
DMR2:176204301	2	1.76E+08	400	1	2.29E-08	0	0		
DMR2:177369301	2	1.77E+08	700	1	7.08E-08	2	0.285714286		
DMR2:178450601	2	1.78E+08	700	2	1.11E-08	2	0.285714286	Rxfp1	Receptor
DMR2:178908501	2	1.79E+08	2900	2	8.30E-09	34	1.172413793		
DMR2:179514801	2	1.8E+08	1100	1	1.68E-07	5	0.454545455		
DMR2:179744101	2	1.8E+08	1600	1	9.23E-09	4	0.25		
DMR2:180317701	2	1.8E+08	2500	2	2.26E-10	9	0.36		
DMR2:181562501	2	1.82E+08	300	1	5.03E-07	1	0.333333333		
DMR2:182003701	2	1.82E+08	800	1	1.50E-07	5	0.625	Fgg;Fga	Cytoskeleton;Signaling
DMR2:182341501	2	1.82E+08	2300	2	2.44E-07	10	0.434782609		
DMR2:182374501	2	1.82E+08	800	5	5.36E-11	4	0.5		
DMR2:182504101	2	1.83E+08	1900	2	3.81E-10	18	0.947368421		
DMR2:184520001	2	1.85E+08	1900	1	5.70E-07	21	1.105263158		
DMR2:185328201	2	1.85E+08	800	2	1.54E-07	7	0.875	Sh3d19	
DMR2:186498701	2	1.86E+08	5400	1	8.23E-08	63	1.166666667	Kirrel	Metabolism
DMR2:186538101	2	1.87E+08	500	3	3.31E-13	2	0.4		
DMR2:186567401	2	1.87E+08	200	1	1.79E-08	1	0.5	Fcrl1	Receptor
DMR2:187179201	2	1.87E+08	900	4	4.53E-08	3	0.333333333	Insrr	Receptor
DMR2:187974601	2	1.88E+08	800	1	5.98E-09	2	0.25	U6;Arhgef2	Signaling
DMR2:188181301	2	1.88E+08	3200	1	1.43E-07	42	1.3125	Gon4l	Transcription
DMR2:189199401	2	1.89E+08	1400	3	2.77E-08	9	0.642857143	She;Il6r	Receptor
DMR2:190195901	2	1.9E+08	2200	2	5.98E-08	22	1		
DMR2:190304601	2	1.9E+08	3600	1	1.42E-10	35	0.972222222		
DMR2:192531501	2	1.93E+08	300	1	3.05E-07	0	0	LOC102550416	
DMR2:192779101	2	1.93E+08	300	1	1.99E-07	1	0.333333333	Smcp	
DMR2:194003101	2	1.94E+08	2700	1	8.95E-07	17	0.62962963	RGD1564513	Unknown
DMR2:194715401	2	1.95E+08	1800	2	2.48E-07	9	0.5	LOC100911679	Unknown

DMR2:194881201	2	1.95E+08	1000	1	1.64E-08	1	0.1	RGD1566337	Unknown
DMR2:194943701	2	1.95E+08	500	1	5.03E-08	1	0.2		
DMR2:195037801	2	1.95E+08	300	1	2.15E-07	0	0	LOC100911679	Unknown
DMR2:196012601	2	1.96E+08	800	1	6.05E-07	9	1.125	Pogz	Transcription
DMR2:196319401	2	1.96E+08	2400	1	1.01E-07	16	0.666666667	Lysmd1;Tnfaip8l2	Unknown
DMR2:197628401	2	1.98E+08	700	1	5.44E-07	4	0.571428571		
DMR2:199150701	2	1.99E+08	1900	1	1.50E-07	32	1.684210526		
DMR2:199866301	2	2E+08	3200	1	5.02E-09	36	1.125	Pde4dip	
DMR2:201332701	2	2.01E+08	300	1	2.61E-07	1	0.333333333	Tbx15	Transcription
DMR2:201906601	2	2.02E+08	300	1	1.79E-08	4	1.333333333		
DMR2:202259201	2	2.02E+08	1500	1	7.23E-07	6	0.4	Spag17	
DMR2:203558401	2	2.04E+08	2400	2	1.00E-10	46	1.916666667		
DMR2:204508601	2	2.05E+08	3400	1	6.49E-07	54	1.588235294	Casq2	Signaling
DMR2:204647401	2	2.05E+08	300	1	2.50E-07	6	2		
DMR2:205072701	2	2.05E+08	900	2	8.73E-10	3	0.333333333		
DMR2:205452601	2	2.05E+08	800	1	1.45E-07	1	0.125	Nr1h5;U1	
DMR2:206032901	2	2.06E+08	800	1	6.17E-07	6	0.75		
DMR2:206335301	2	2.06E+08	3600	1	4.81E-09	39	1.083333333	Ptpn22	Signaling
DMR2:206896901	2	2.07E+08	4800	1	8.53E-11	65	1.354166667	RGD1564469	
DMR2:207183101	2	2.07E+08	1900	1	1.01E-07	20	1.052631579		
DMR2:207478901	2	2.07E+08	1800	1	7.29E-07	27	1.5	St7l	Unknown
DMR2:208344101	2	2.08E+08	1100	1	2.85E-07	12	1.090909091	LOC100911347;Tmigd3	
DMR2:210660301	2	2.11E+08	1900	1	1.99E-07	14	0.736842105	Eps8l3	Unknown
DMR2:211687201	2	2.12E+08	800	1	9.30E-07	2	0.25	Fndc7;Mir6314	
DMR2:211958701	2	2.12E+08	6300	1	4.09E-07	82	1.301587302	Slc25a24	Binding Protein
DMR2:212018601	2	2.12E+08	800	1	4.12E-07	9	1.125		
DMR2:214101601	2	2.14E+08	400	1	2.93E-07	15	3.75		
DMR2:215354901	2	2.15E+08	3700	1	9.37E-07	35	0.945945946		

DMR2:216643201	2	2.17E+08	800	2	4.44E-08	1	0.125		
DMR2:216968401	2	2.17E+08	500	1	4.26E-07	2	0.4	Col11a1	Cytoskeleton
DMR2:216985301	2	2.17E+08	300	1	3.97E-07	4	1.333333333	Col11a1	Cytoskeleton
DMR2:220013201	2	2.2E+08	1100	2	4.29E-14	8	0.727272727	Plppr5	
DMR2:220420701	2	2.2E+08	2400	1	7.75E-10	16	0.666666667	Frrs1	Metabolism
DMR2:221155101	2	2.21E+08	400	1	2.33E-07	0	0		
DMR2:224665401	2	2.25E+08	1500	1	1.04E-22	21	1.4		
DMR2:227182701	2	2.27E+08	1200	1	1.35E-07	8	0.666666667	Myoz2	Unknown
DMR2:227255001	2	2.27E+08	3000	1	3.88E-07	35	1.166666667	Synpo2	Development
DMR2:227425101	2	2.27E+08	3000	1	2.67E-07	23	0.766666667		
DMR2:228016601	2	2.28E+08	1000	1	1.84E-08	1	0.1		
DMR2:229266901	2	2.29E+08	300	2	7.70E-09	3	1	Ndst4	Metabolism
DMR2:230354301	2	2.3E+08	1000	2	3.15E-09	11	1.1	Sec24b	Transport
DMR2:230703101	2	2.31E+08	1400	3	1.51E-12	13	0.928571429		
DMR2:231112501	2	2.31E+08	400	1	8.68E-07	3	0.75	Camk2d	Signaling
DMR2:232118901	2	2.32E+08	600	2	1.59E-08	0	0	Alpk1;Tifa	Signaling;Apoptosis
DMR2:233476801	2	2.33E+08	200	1	5.20E-07	0	0		
DMR2:233734401	2	2.34E+08	400	2	9.69E-08	3	0.75	Enpep	Metabolism
DMR2:234716401	2	2.35E+08	300	1	9.36E-07	10	3.333333333		
DMR2:235738101	2	2.36E+08	300	1	3.57E-07	4	1.333333333	Etnppl	Metabolism
DMR2:235783301	2	2.36E+08	1500	1	7.72E-07	16	1.066666667		
DMR2:235991101	2	2.36E+08	2100	1	2.97E-07	32	1.523809524		
DMR2:236186801	2	2.36E+08	200	1	2.93E-07	1	0.5		
DMR2:236412201	2	2.36E+08	900	1	9.43E-07	15	1.666666667	Cyp2u1	Metabolism
DMR2:236507801	2	2.37E+08	3200	1	1.08E-07	48	1.5		
DMR2:237908801	2	2.38E+08	1200	1	1.84E-07	11	0.916666667	Tbck	Unknown
DMR2:237940801	2	2.38E+08	300	1	2.69E-11	0	0	Tbck	Unknown
DMR2:239073201	2	2.39E+08	700	1	5.05E-07	2	0.285714286	AABR07013477.2	

DMR2:239115801	2	2.39E+08	3300	1	7.24E-07	45	1.363636364		
DMR2:239475801	2	2.39E+08	500	1	5.55E-07	1	0.2		
DMR2:239494601	2	2.39E+08	400	2	5.17E-08	3	0.75		
DMR2:239498301	2	2.39E+08	1700	1	4.11E-09	13	0.764705882		
DMR2:240521601	2	2.41E+08	1700	1	2.11E-08	12	0.705882353	Slc9b2;Slc9b1	
DMR2:240906601	2	2.41E+08	300	2	2.51E-09	0	0	U2	
DMR2:241036201	2	2.41E+08	700	2	2.82E-09	3	0.428571429	Slc39a8	Transport
DMR2:242209301	2	2.42E+08	300	1	1.15E-08	0	0		
DMR2:242265501	2	2.42E+08	3700	2	2.50E-08	44	1.189189189		
DMR2:242590001	2	2.43E+08	1800	2	1.49E-07	6	0.333333333		
DMR2:242623501	2	2.43E+08	800	2	7.81E-09	0	0		
DMR2:243238801	2	2.43E+08	2000	1	6.17E-07	4	0.2		
DMR2:243475801	2	2.43E+08	300	1	5.16E-09	5	1.666666667	RGD1309170	
DMR2:245137801	2	2.45E+08	900	1	4.53E-09	1	0.111111111	AABR07013586.1	
DMR2:245160401	2	2.45E+08	1500	1	4.80E-07	26	1.733333333		
DMR2:245628801	2	2.46E+08	2100	2	1.30E-10	10	0.476190476		
DMR2:245633601	2	2.46E+08	300	2	4.53E-10	1	0.333333333		
DMR2:246494701	2	2.46E+08	900	1	4.47E-07	8	0.888888889		
DMR2:246579901	2	2.47E+08	700	1	1.19E-08	2	0.285714286		
DMR2:247093401	2	2.47E+08	300	1	2.95E-07	6	2		
DMR2:248577901	2	2.49E+08	4900	1	1.13E-08	71	1.448979592	AABR07013654.1	
DMR2:249977101	2	2.5E+08	5100	1	2.04E-07	31	0.607843137		
DMR2:250205901	2	2.5E+08	2800	3	2.55E-11	30	1.071428571	Lmo4	Cytoskeleton
DMR2:250683701	2	2.51E+08	2300	3	8.42E-09	22	0.956521739		
DMR2:250796601	2	2.51E+08	800	1	4.02E-08	20	2.5	Clca4l	Transport
DMR2:251780301	2	2.52E+08	400	1	1.27E-07	4	1		
DMR2:252027301	2	2.52E+08	1600	2	7.38E-11	12	0.75	Mcoln2	Transport
DMR2:252395601	2	2.52E+08	1800	1	4.92E-07	11	0.611111111		
DMR2:252456901	2	2.52E+08	2200	1	1.41E-08	50	2.272727273	Dnase2b;Uox	Transcription

DMR2:253262901	2	2.53E+08	1200	1	4.21E-09	7	0.583333333		
DMR2:254330701	2	2.54E+08	4200	2	2.27E-08	67	1.595238095	Rn60_2_2544.1	
DMR2:254934601	2	2.55E+08	6300	1	5.51E-07	71	1.126984127		
DMR2:255056301	2	2.55E+08	1200	3	2.69E-11	4	0.333333333	AABR07013762.1	
DMR2:255490701	2	2.55E+08	300	1	4.88E-07	0	0		
DMR2:255615301	2	2.56E+08	500	1	3.49E-09	3	0.6		
DMR2:256337401	2	2.56E+08	500	1	2.93E-07	1	0.2		
DMR2:257104201	2	2.57E+08	3700	1	8.49E-07	33	0.891891892		
DMR2:257117401	2	2.57E+08	500	2	1.55E-10	1	0.2		
DMR2:257734701	2	2.58E+08	700	1	3.65E-07	10	1.428571429	Ak5	Signaling
DMR2:258829901	2	2.59E+08	9200	1	5.90E-07	206	2.239130435	Adgrl2	
DMR2:259023101	2	2.59E+08	1500	1	3.17E-08	26	1.733333333		
DMR2:259146901	2	2.59E+08	1300	1	9.86E-07	10	0.769230769	AABR07013843.1	
DMR2:259334901	2	2.59E+08	1500	2	1.95E-08	9	0.6		
DMR2:261168601	2	2.61E+08	400	1	3.11E-07	6	1.5	Tnni3k	Transcription
DMR2:261329401	2	2.61E+08	600	1	2.59E-08	4	0.666666667	Tnni3k	Transcription
DMR2:261988201	2	2.62E+08	300	1	1.01E-09	2	0.666666667		
DMR2:264006501	2	2.64E+08	400	2	1.65E-07	2	0.5		
DMR2:264701001	2	2.65E+08	2400	2	5.24E-11	7	0.291666667	Depdc1	Transcription
DMR2:265633701	2	2.66E+08	1200	1	2.68E-12	6	0.5		
DMR3:601	3	601	500	1	3.31E-07	1	0.2	AABR07051177.1	
DMR3:457301	3	457301	400	1	6.48E-07	3	0.75	Spopl	Transcription
DMR3:1566001	3	1566001	1600	1	3.36E-07	13	0.8125	Pax8	Transcription
DMR3:2243801	3	2243801	400	2	7.45E-09	0	0	Pnpla7	Metabolism
DMR3:2350001	3	2350001	3600	1	5.30E-07	50	1.388888889		
DMR3:2357901	3	2357901	5800	2	6.12E-09	120	2.068965517		
DMR3:2418201	3	2418201	1200	3	4.42E-09	6	0.5	Tor4a;Nelfb;U6	
DMR3:2599401	3	2599401	2000	2	2.26E-08	16	0.8	Sapcd2	
DMR3:2927801	3	2927801	2700	1	1.25E-07	20	0.740740741	LOC108348105	

DMR3:3762901	3	3762901	1300	2	2.54E-08	11	0.846153846	Gpsm1	Signaling
DMR3:4113901	3	4113901	1500	1	5.19E-10	33	2.2	AABR07051251.1	
DMR3:5766801	3	5766801	1100	1	4.24E-09	12	1.090909091	Sardh;AABR07051353.1	Metabolism
DMR3:5934701	3	5934701	800	1	9.92E-07	9	1.125	Vav2	Signaling
DMR3:5991501	3	5991501	700	2	1.74E-08	13	1.857142857	Brd3;Mir7578	Transcription
DMR3:6132401	3	6132401	5800	2	1.24E-07	71	1.224137931		
DMR3:6300401	3	6300401	1500	2	3.54E-07	16	1.066666667	Rxra	Receptor
DMR3:6393901	3	6393901	3200	2	4.92E-08	60	1.875		
DMR3:6403401	3	6403401	4000	1	3.19E-08	68	1.7		
DMR3:6449701	3	6449701	3700	1	9.20E-08	56	1.513513514	Col5a1	Extracellular Matrix
DMR3:6842901	3	6842901	300	1	7.12E-07	1	0.333333333		
DMR3:8414001	3	8414001	300	1	1.38E-07	1	0.333333333	Urm1;Mir2964	
DMR3:8697501	3	8697501	500	3	1.62E-12	0	0	Zer1;Tbc1d13	Signaling
DMR3:9519901	3	9519901	1200	1	2.41E-07	20	1.666666667		
DMR3:9995701	3	9995701	600	1	3.32E-07	1	0.166666667	Fibcd1	Cytoskeleton
DMR3:10502101	3	10502101	3200	1	4.23E-07	42	1.3125		
DMR3:12548101	3	12548101	300	1	4.99E-07	2	0.666666667		
DMR3:12674201	3	12674201	600	2	4.21E-10	2	0.333333333	Lmx1b	
DMR3:13223201	3	13223201	1500	1	2.15E-07	23	1.533333333	Pbx3	Transcription
DMR3:14061401	3	14061401	2000	1	5.98E-07	21	1.05	C5	Immune
DMR3:15970001	3	15970001	1300	1	3.92E-08	16	1.230769231		
DMR3:16398601	3	16398601	600	1	3.75E-08	3	0.5		
DMR3:18137301	3	18137301	400	2	4.26E-09	1	0.25		
DMR3:18287801	3	18287801	400	1	1.07E-08	1	0.25		
DMR3:20788601	3	20788601	600	1	2.83E-07	23	3.833333333		
DMR3:20870501	3	20870501	300	1	8.67E-07	2	0.666666667	Olr413	
DMR3:22732201	3	22732201	1300	3	1.15E-11	10	0.769230769		

DMR3:23107001	3	23107001	600	1	1.97E-10	0	0		
DMR3:25157701	3	25157701	300	1	5.99E-07	1	0.333333333	SNORA70	
DMR3:26911001	3	26911001	2300	1	2.97E-08	28	1.217391304		
DMR3:29411901	3	29411901	600	1	1.78E-07	7	1.166666667	Gtdc1	Metabolism
DMR3:30067801	3	30067801	1300	1	4.99E-07	9	0.692307692		
DMR3:30150201	3	30150201	1200	1	2.82E-07	6	0.5		
DMR3:30186201	3	30186201	1700	2	1.54E-09	10	0.588235294		
DMR3:31148901	3	31148901	1800	1	6.02E-07	13	0.722222222		
DMR3:31624501	3	31624501	500	2	6.43E-09	2	0.4		
DMR3:32384101	3	32384101	500	1	6.64E-07	12	2.4		
DMR3:33878601	3	33878601	900	3	9.72E-12	0	0		
DMR3:34034601	3	34034601	300	1	6.36E-07	3	1		
DMR3:34636801	3	34636801	300	1	4.29E-07	0	0		
DMR3:35224201	3	35224201	2900	1	1.26E-07	37	1.275862069	Kif5c	Cytoskeleton
DMR3:36530301	3	36530301	600	1	3.74E-07	6	1		
DMR3:37498201	3	37498201	2000	1	1.59E-07	15	0.75		
DMR3:37980401	3	37980401	2500	1	5.58E-09	26	1.04	Cacnb4	Transport
DMR3:38196101	3	38196101	1500	1	6.65E-09	12	0.8		
DMR3:39672201	3	39672201	1100	1	7.47E-19	5	0.454545455		
DMR3:41240501	3	41240501	3400	2	3.25E-08	30	0.882352941		
DMR3:42637701	3	42637701	300	1	9.79E-07	1	0.333333333		
DMR3:43266801	3	43266801	2800	1	1.39E-07	31	1.107142857	Gpd2	Metabolism
DMR3:44569701	3	44569701	1900	1	1.15E-16	37	1.947368421		
DMR3:45442701	3	45442701	300	1	4.41E-07	5	1.666666667		
DMR3:46937501	3	46937501	300	2	1.27E-07	0	0	Rbms1	Transcription
DMR3:48353501	3	48353501	400	1	1.06E-08	2	0.5	Dpp4	Protease
DMR3:50156801	3	50156801	600	1	2.23E-10	2	0.333333333		
DMR3:50195401	3	50195401	1400	1	8.74E-07	10	0.714285714		
DMR3:50557201	3	50557201	900	2	4.35E-12	4	0.444444444		

DMR3:51025101	3	51025101	2800	1	7.98E-08	24	0.857142857	Grb14;U4	Receptor
DMR3:51140801	3	51140801	500	1	1.03E-07	3	0.6	Cobll1	Development
DMR3:51311301	3	51311301	2000	1	4.89E-09	19	0.95		
DMR3:51357901	3	51357901	300	1	9.28E-07	3	1		
DMR3:52053801	3	52053801	2600	1	9.70E-09	14	0.538461538	Csrnp3	Unknown
DMR3:52706001	3	52706001	1100	2	6.48E-07	11	1	7SK	
DMR3:54394301	3	54394301	2000	1	3.04E-08	17	0.85	Stk39	
DMR3:56334901	3	56334901	1500	1	8.47E-07	8	0.533333333		
DMR3:56492801	3	56492801	1300	3	2.13E-10	5	0.384615385	Myo3b	Cytoskeleton
DMR3:56557301	3	56557301	300	2	6.92E-10	0	0	Myo3b	Cytoskeleton
DMR3:56640901	3	56640901	1600	1	1.73E-07	18	1.125		
DMR3:56726201	3	56726201	400	1	6.05E-07	0	0		
DMR3:56955501	3	56955501	900	2	1.59E-17	1	0.111111111		
DMR3:57780001	3	57780001	2300	1	1.21E-07	29	1.260869565	AC107446.2	
DMR3:58937001	3	58937001	3800	2	2.52E-07	47	1.236842105		
DMR3:59134401	3	59134401	400	1	2.67E-09	1	0.25		
DMR3:59187501	3	59187501	2200	1	1.37E-08	27	1.227272727		
DMR3:59202101	3	59202101	1600	1	4.03E-07	23	1.4375		
DMR3:59223801	3	59223801	1000	1	3.35E-07	11	1.1		
DMR3:59275601	3	59275601	600	1	1.31E-07	10	1.666666667	AABR07052521.1	
DMR3:61043201	3	61043201	400	2	1.11E-09	0	0		
DMR3:61479601	3	61479601	500	1	1.49E-07	1	0.2	Lnpk	
DMR3:62336401	3	62336401	1500	1	1.55E-09	20	1.333333333		
DMR3:62593501	3	62593501	2300	2	2.14E-08	28	1.217391304		
DMR3:62960701	3	62960701	400	3	1.94E-12	0	0	Pde11a	Signaling
DMR3:63252701	3	63252701	2400	1	1.67E-14	30	1.25		
DMR3:63286601	3	63286601	1000	1	5.92E-07	7	0.7		
DMR3:64210101	3	64210101	1100	1	6.96E-09	7	0.636363636		
DMR3:64790401	3	64790401	1600	1	1.17E-07	11	0.6875		

DMR3:65077401	3	65077401	900	2	1.09E-08	8	0.888888889		
DMR3:65231501	3	65231501	500	1	6.50E-07	5	1		
DMR3:65273401	3	65273401	1400	2	4.93E-09	10	0.714285714		
DMR3:66006501	3	66006501	800	1	4.27E-07	17	2.125	5S_rRNA	
DMR3:67312201	3	67312201	700	2	4.24E-07	2	0.285714286		
DMR3:67679701	3	67679701	500	1	7.60E-07	1	0.2	AABR07052643.1	
DMR3:70394501	3	70394501	600	1	1.49E-08	3	0.5		
DMR3:71507701	3	71507701	300	1	8.13E-07	3	1		
DMR3:72158401	3	72158401	1200	1	8.05E-08	10	0.833333333	AC096003.1;U6;Serping1	Proteolysis
DMR3:73166401	3	73166401	400	1	4.43E-07	0	0	Olr458	
DMR3:73441801	3	73441801	3200	1	2.56E-09	21	0.65625	AABR07052755.2	
DMR3:75645401	3	75645401	5800	1	4.50E-07	60	1.034482759	AABR07052795.1;Actg1;AABR07052795.2	Cytoskeleton
DMR3:76511401	3	76511401	200	2	2.16E-16	0	0	Olr623	Receptor
DMR3:76984101	3	76984101	2000	1	6.44E-07	43	2.15	AC105624.1	
DMR3:77496501	3	77496501	300	1	3.23E-10	0	0	Olr662	
DMR3:80044301	3	80044301	1300	1	9.54E-07	10	0.769230769	Ddb2	Transcription
DMR3:80303701	3	80303701	1400	2	1.01E-07	11	0.785714286	RGD1309540	Unknown
DMR3:81617501	3	81617501	1300	1	1.60E-07	13	1		
DMR3:82160801	3	82160801	400	1	6.33E-07	2	0.5	Tspan18	Cytoskeleton
DMR3:82395501	3	82395501	2200	3	2.12E-10	26	1.181818182		
DMR3:82632201	3	82632201	800	1	5.57E-07	4	0.5	Ext2;AABR07052895.1	Metabolism
DMR3:83384901	3	83384901	700	1	7.93E-07	6	0.857142857		
DMR3:83948901	3	83948901	500	1	4.20E-07	1	0.2		
DMR3:84108501	3	84108501	400	1	5.72E-07	0	0		
DMR3:85048601	3	85048601	300	1	9.46E-08	4	1.333333333		

DMR3:85115501	3	85115501	1100	1	1.05E-07	7	0.636363636		
DMR3:85298301	3	85298301	300	1	7.49E-09	2	0.666666667		
DMR3:85637001	3	85637001	300	1	3.76E-09	0	0	Lrrc4c	Extracellular Matrix
DMR3:86894601	3	86894601	400	2	2.49E-11	1	0.25		
DMR3:87442001	3	87442001	1200	5	2.02E-12	14	1.166666667		
DMR3:88818901	3	88818901	500	1	3.34E-09	1	0.2		
DMR3:90029701	3	90029701	1700	3	8.25E-11	9	0.529411765		
DMR3:91033001	3	91033001	800	1	4.41E-07	6	0.75		
DMR3:91794701	3	91794701	500	1	6.51E-08	2	0.4	Ldlrad3	Receptor
DMR3:91856301	3	91856301	1200	1	4.01E-07	9	0.75		
DMR3:92119501	3	92119501	600	2	7.00E-11	2	0.333333333	LOC102551100	
DMR3:92682301	3	92682301	500	1	7.33E-07	1	0.2		
DMR3:93137901	3	93137901	500	2	2.39E-08	0	0		
DMR3:93768901	3	93768901	1400	1	7.02E-07	21	1.5	Caprin1	Transport
DMR3:95153101	3	95153101	3500	1	5.44E-10	34	0.971428571	Wt1	Transcription
DMR3:95486901	3	95486901	700	1	2.44E-08	3	0.428571429		
DMR3:95749301	3	95749301	700	2	1.51E-08	3	0.428571429		
DMR3:96290201	3	96290201	1400	1	4.35E-08	6	0.428571429		
DMR3:96810601	3	96810601	900	2	1.76E-10	5	0.555555556		
DMR3:97219601	3	97219601	1200	2	1.40E-08	7	0.583333333	AABR07053293.1	
DMR3:97449201	3	97449201	300	1	6.41E-07	4	1.333333333		
DMR3:99050601	3	99050601	200	1	2.79E-07	0	0		
DMR3:100249801	3	1E+08	2600	5	1.38E-08	12	0.461538462	Mettl15	
DMR3:100535901	3	1.01E+08	200	1	1.01E-07	1	0.5		
DMR3:100889501	3	1.01E+08	700	1	5.50E-07	2	0.285714286		
DMR3:102584601	3	1.03E+08	1000	1	4.34E-10	3	0.3	Olr754;AC121203.1	
DMR3:103750501	3	1.04E+08	700	2	1.18E-08	1	0.142857143	Nutm1;Nop10;Slc12a6	Transcription;Transport

DMR3:104243301	3	1.04E+08	1500	2	8.72E-08	21	1.4	Ryr3	Receptor
DMR3:104384301	3	1.04E+08	600	1	6.53E-07	3	0.5	Ryr3	Receptor
DMR3:104678001	3	1.05E+08	5200	1	1.19E-09	51	0.980769231	Hmgn4	
DMR3:106893201	3	1.07E+08	1600	1	5.99E-08	28	1.75		
DMR3:106926201	3	1.07E+08	500	1	5.45E-09	2	0.4	AABR07053435.1	
DMR3:107758401	3	1.08E+08	1000	1	2.23E-07	37	3.7	Meis2	Transcription
DMR3:109402201	3	1.09E+08	600	1	5.59E-07	1	0.166666667	AABR07053480.1	
DMR3:109734401	3	1.1E+08	400	2	9.31E-09	1	0.25		
DMR3:110316801	3	1.1E+08	600	3	2.15E-07	0	0	Bmf	Apoptosis
DMR3:110742801	3	1.11E+08	900	4	7.83E-10	3	0.333333333	Chst14	Metabolism
DMR3:110813101	3	1.11E+08	1100	3	5.29E-08	11	1	Ccdc32	Transcription
DMR3:111152601	3	1.11E+08	2000	3	2.91E-07	22	1.1	Dll4;Chac1	Growth Factors & Cytokines;Metabolism
DMR3:113030101	3	1.13E+08	1600	4	1.97E-09	4	0.25	Tgm7l1	
DMR3:113299801	3	1.13E+08	400	1	1.05E-09	2	0.5	Mfap1a;AC116071.1	Extracellular Matrix
DMR3:113726101	3	1.14E+08	900	3	2.92E-08	9	1		
DMR3:115027501	3	1.15E+08	1100	1	4.91E-07	14	1.272727273		
DMR3:115454501	3	1.15E+08	4400	1	9.24E-08	52	1.181818182		
DMR3:116032801	3	1.16E+08	1200	1	5.99E-08	3	0.25		
DMR3:116364501	3	1.16E+08	1500	1	7.15E-08	9	0.6		
DMR3:117301301	3	1.17E+08	3200	1	1.21E-10	31	0.96875		
DMR3:117751501	3	1.18E+08	2400	1	4.21E-07	30	1.25	Fbn1	Development
DMR3:117960901	3	1.18E+08	2300	3	1.43E-07	10	0.434782609	Shc4	Signaling
DMR3:117997601	3	1.18E+08	1900	1	2.41E-08	26	1.368421053	Shc4;Secisbp2l	Signaling;Receptor
DMR3:118149201	3	1.18E+08	1300	2	1.20E-09	10	0.769230769	Galk2	Signaling

DMR3:118384101	3	1.18E+08	800	1	8.36E-09	5	0.625	Fam227b	
DMR3:118643401	3	1.19E+08	300	1	7.56E-07	0	0	Atp8b4	Transport
DMR3:118918501	3	1.19E+08	1700	1	4.59E-07	27	1.588235294	Atp8b4	Transport
DMR3:119097601	3	1.19E+08	1700	1	8.68E-07	5	0.294117647	Gabpb1;U1	Transcription
DMR3:119858301	3	1.2E+08	300	1	6.08E-08	5	1.666666667		
DMR3:121269001	3	1.21E+08	300	1	2.11E-07	1	0.333333333	Mertk	Signaling
DMR3:121322901	3	1.21E+08	2600	4	6.86E-11	24	0.923076923	Mertk;SNORA25	Signaling
DMR3:122145101	3	1.22E+08	2700	2	2.53E-08	41	1.518518519	Sirpa	Signaling
DMR3:122258101	3	1.22E+08	1500	2	2.97E-11	8	0.533333333		
DMR3:122552301	3	1.23E+08	900	2	4.09E-11	4	0.444444444	Tgm3	Translation
DMR3:123333601	3	1.23E+08	400	2	1.09E-08	3	0.75	RGD1565616	EST
DMR3:123379701	3	1.23E+08	300	1	5.48E-08	4	1.333333333	RGD1565616;U6	EST
DMR3:123667401	3	1.24E+08	1500	4	3.09E-14	3	0.2	Hspa12b	Signaling
DMR3:123975601	3	1.24E+08	1500	1	1.54E-07	10	0.666666667		
DMR3:124032801	3	1.24E+08	2000	1	3.74E-07	17	0.85		
DMR3:124498601	3	1.24E+08	800	2	1.59E-09	12	1.5		
DMR3:125309801	3	1.25E+08	500	3	7.59E-12	2	0.4		
DMR3:125593501	3	1.26E+08	2200	4	6.15E-20	17	0.772727273	Fermt1	Signaling
DMR3:126133301	3	1.26E+08	1200	2	3.19E-08	5	0.416666667		
DMR3:126823301	3	1.27E+08	500	3	3.45E-08	0	0		
DMR3:126875701	3	1.27E+08	400	1	8.13E-08	2	0.5		
DMR3:126935901	3	1.27E+08	300	1	4.26E-07	4	1.333333333		
DMR3:129446801	3	1.29E+08	700	1	8.97E-07	3	0.428571429		
DMR3:130358201	3	1.3E+08	400	1	7.80E-07	0	0		
DMR3:131511701	3	1.32E+08	500	1	1.87E-09	1	0.2		
DMR3:132065501	3	1.32E+08	1400	1	7.04E-07	7	0.5		
DMR3:132612001	3	1.33E+08	400	3	2.73E-13	0	0	Sptlc3	Metabolism
DMR3:132804801	3	1.33E+08	300	1	3.48E-07	1	0.333333333		
DMR3:133455301	3	1.33E+08	2400	1	9.17E-08	20	0.833333333		

DMR3:133497901	3	1.33E+08	2500	1	6.07E-07	29	1.16		
DMR3:135503801	3	1.36E+08	700	1	4.69E-07	3	0.428571429		
DMR3:136440201	3	1.36E+08	300	1	8.43E-07	0	0		
DMR3:137796701	3	1.38E+08	1600	1	4.87E-07	12	0.75	Pcsk2	Proteolysis
DMR3:138093201	3	1.38E+08	1500	2	2.69E-09	4	0.266666667	Rrbp1	Receptor
DMR3:138144101	3	1.38E+08	1500	2	1.06E-08	18	1.2		
DMR3:138886101	3	1.39E+08	400	2	4.61E-09	4	1	Dtd1	Transcription
DMR3:140449701	3	1.4E+08	300	1	1.09E-07	1	0.333333333		
DMR3:140764101	3	1.41E+08	2500	2	2.79E-08	41	1.64		
DMR3:141711101	3	1.42E+08	300	1	3.03E-12	1	0.333333333		
DMR3:141853501	3	1.42E+08	600	1	1.19E-07	3	0.5		
DMR3:142515601	3	1.43E+08	500	2	1.10E-07	3	0.6		
DMR3:142701401	3	1.43E+08	1200	1	7.82E-07	9	0.75		
DMR3:142872901	3	1.43E+08	300	1	8.08E-07	1	0.333333333		
DMR3:143237701	3	1.43E+08	4900	1	2.58E-07	35	0.714285714		
DMR3:144253201	3	1.44E+08	500	2	5.57E-07	13	2.6		
DMR3:145256801	3	1.45E+08	3300	3	7.65E-09	35	1.060606061		
DMR3:145337601	3	1.45E+08	1900	2	2.52E-10	10	0.526315789		
DMR3:145514901	3	1.46E+08	100	1	3.43E-10	0	0		
DMR3:145566701	3	1.46E+08	700	1	1.71E-07	3	0.428571429		
DMR3:145669901	3	1.46E+08	500	3	6.41E-08	8	1.6		
DMR3:146269001	3	1.46E+08	2000	3	1.64E-14	32	1.6		
DMR3:146600801	3	1.47E+08	3900	3	2.53E-12	49	1.256410256	Pygb	Metabolism
DMR3:146643201	3	1.47E+08	1000	1	9.16E-09	8	0.8	Pygb;Abhd12	Metabolism
DMR3:147099101	3	1.47E+08	2000	1	6.72E-07	28	1.4	Sdcbp2;Snph	Transport
DMR3:148431701	3	1.48E+08	700	1	6.69E-07	13	1.857142857	Dusp15;Ttll9	Signaling;Cytoskeleton
DMR3:148669001	3	1.49E+08	1400	3	1.54E-14	18	1.285714286	Tm9sf4	Metabolism
DMR3:149024701	3	1.49E+08	2700	1	1.94E-07	22	0.814814815		

DMR3:149521601	3	1.5E+08	1000	1	4.74E-07	6	0.6	Bpifa2f	
DMR3:150518101	3	1.51E+08	3700	1	8.10E-08	60	1.621621622		
DMR3:150569501	3	1.51E+08	300	2	6.54E-14	1	0.333333333	Asip	Signaling
DMR3:151058501	3	1.51E+08	600	1	1.36E-09	0	0	Acss2	Metabolism
DMR3:151776801	3	1.52E+08	2900	1	6.23E-07	31	1.068965517		
DMR3:151812501	3	1.52E+08	300	2	2.31E-09	0	0		
DMR3:152113401	3	1.52E+08	3100	2	1.94E-08	22	0.709677419		
DMR3:152426201	3	1.52E+08	2200	1	6.95E-08	32	1.454545455	Cnbd2	
DMR3:154223001	3	1.54E+08	3900	1	2.18E-08	68	1.743589744	Ctnnbl1;AABR07054456.2	
DMR3:154718501	3	1.55E+08	400	1	7.15E-07	4	1	RGD1563354;AABR07054460.3	
DMR3:155199701	3	1.55E+08	400	2	9.70E-08	3	0.75	Ppp1r16b;RGD1563145	Signaling
DMR3:156351301	3	1.56E+08	400	1	6.44E-07	11	2.75		
DMR3:158009001	3	1.58E+08	200	1	6.54E-07	2	1	Ptprt	Receptor
DMR3:158089601	3	1.58E+08	2200	1	2.30E-07	24	1.090909091	Ptprt	Receptor
DMR3:158420501	3	1.58E+08	2200	1	3.12E-08	23	1.045454545		
DMR3:158731501	3	1.59E+08	2300	1	8.98E-08	26	1.130434783	SNORA17	
DMR3:159003901	3	1.59E+08	200	1	6.12E-07	0	0	LOC108348048	
DMR3:159694301	3	1.6E+08	1200	4	3.48E-12	8	0.666666667	Tox2	
DMR3:159770301	3	1.6E+08	500	1	1.17E-07	4	0.8	Jph2	
DMR3:159898701	3	1.6E+08	500	1	1.40E-07	15	3	R3hdml;Hnf4a	
DMR3:160315501	3	1.6E+08	2500	1	2.81E-08	30	1.2		
DMR3:160420901	3	1.6E+08	4600	2	9.62E-09	71	1.543478261	Pabpc1l	Transcription
DMR3:160822001	3	1.61E+08	2700	1	3.30E-09	23	0.851851852		
DMR3:160969601	3	1.61E+08	300	1	5.34E-07	1	0.333333333	AABR07054564.1	
DMR3:161075401	3	1.61E+08	2400	3	3.73E-11	21	0.875	Wfdc8;Wfdc6b;SNORA17	Signaling

DMR3:162240801	3	1.62E+08	1600	4	1.06E-08	4	0.25	AABR07054583.1	
DMR3:163753701	3	1.64E+08	1700	1	6.81E-07	12	0.705882353	Stau1	Transcription
DMR3:164522501	3	1.65E+08	1400	1	8.97E-07	18	1.285714286		
DMR3:164772001	3	1.65E+08	3900	1	3.49E-07	42	1.076923077		
DMR3:165295401	3	1.65E+08	2100	2	5.79E-15	31	1.476190476	Nfatc2	Transcription
DMR3:165410801	3	1.65E+08	1200	1	2.66E-07	8	0.666666667	Atp9a	Transport
DMR3:165759601	3	1.66E+08	3000	1	6.61E-08	49	1.633333333		
DMR3:166220501	3	1.66E+08	2400	2	5.42E-11	30	1.25		
DMR3:166326401	3	1.66E+08	1500	1	7.75E-10	28	1.866666667		
DMR3:166478501	3	1.66E+08	3600	2	4.15E-08	65	1.805555556		
DMR3:166510101	3	1.67E+08	400	1	9.75E-08	1	0.25		
DMR3:166683601	3	1.67E+08	500	2	6.00E-13	1	0.2		
DMR3:167561101	3	1.68E+08	1000	1	9.58E-07	2	0.2		
DMR3:167986401	3	1.68E+08	4000	1	4.47E-07	70	1.75	Bcas1	
DMR3:168389401	3	1.68E+08	1600	1	5.13E-08	17	1.0625	Dok5	Unknown
DMR3:168781601	3	1.69E+08	2000	1	4.41E-07	25	1.25	LOC102552273	
DMR3:168923901	3	1.69E+08	2900	2	3.59E-08	38	1.310344828		
DMR3:169032401	3	1.69E+08	1000	1	8.36E-07	11	1.1		
DMR3:169185401	3	1.69E+08	500	1	1.43E-07	7	1.4		
DMR3:169440601	3	1.69E+08	800	2	3.53E-09	2	0.25		
DMR3:169730901	3	1.7E+08	1900	1	1.51E-07	14	0.736842105		
DMR3:170110001	3	1.7E+08	500	1	2.49E-09	1	0.2		
DMR3:170195901	3	1.7E+08	1000	1	1.60E-08	7	0.7		
DMR3:170283601	3	1.7E+08	3600	3	1.25E-07	65	1.805555556		
DMR3:170412701	3	1.7E+08	5100	1	1.16E-07	73	1.431372549	Cass4	Transcription
DMR3:170428401	3	1.7E+08	2800	1	1.68E-07	60	2.142857143	Cass4	Transcription
DMR3:170518801	3	1.71E+08	6300	1	4.42E-07	110	1.746031746		
DMR3:170568701	3	1.71E+08	2100	2	1.05E-08	17	0.80952381		
DMR3:170572401	3	1.71E+08	1000	1	1.29E-11	6	0.6		

DMR3:170874401	3	1.71E+08	700	1	6.49E-07	5	0.714285714	Bmp7	Growth Factors & Cytokines
DMR3:171406301	3	1.71E+08	800	2	6.29E-09	7	0.875		
DMR3:171535301	3	1.72E+08	4000	1	3.91E-07	58	1.45		
DMR3:171910801	3	1.72E+08	500	1	9.73E-08	2	0.4	Apcdd1l	
DMR3:171918501	3	1.72E+08	800	2	1.77E-08	14	1.75	Apcdd1l	
DMR3:171984401	3	1.72E+08	400	1	5.62E-07	0	0		
DMR3:173427101	3	1.73E+08	1400	1	3.37E-07	16	1.142857143		
DMR3:174284501	3	1.74E+08	700	3	7.28E-09	5	0.714285714		
DMR3:174422401	3	1.74E+08	900	1	2.20E-08	4	0.444444444		
DMR3:174853401	3	1.75E+08	1200	1	1.67E-07	5	0.416666667		
DMR3:175866901	3	1.76E+08	600	2	9.42E-09	2	0.333333333	Slco4a1	Transport
DMR3:176266401	3	1.76E+08	900	5	1.13E-14	0	0		
DMR3:176846901	3	1.77E+08	1300	1	3.53E-08	8	0.615384615	Rtel1	Cell Cycle
DMR3:177219101	3	1.77E+08	2800	1	9.74E-08	10	0.357142857	Oprl1	Receptor
DMR4:467301	4	467301	1400	2	4.67E-13	13	0.928571429	Cnpy1	
DMR4:798601	4	798601	500	1	5.33E-10	2	0.4		
DMR4:806401	4	806401	1400	1	2.50E-10	9	0.642857143		
DMR4:1501301	4	1501301	400	1	9.02E-07	1	0.25	Olr1237;LOC103692039	Receptor
DMR4:2170901	4	2170901	800	1	2.08E-07	3	0.375	Lmbr1	Receptor
DMR4:2439801	4	2439801	200	1	3.95E-07	6	3		
DMR4:3139001	4	3139001	300	1	1.79E-07	1	0.333333333		
DMR4:3208801	4	3208801	300	1	8.64E-07	1	0.333333333		
DMR4:3701601	4	3701601	300	1	3.31E-09	1	0.333333333		
DMR4:4311301	4	4311301	300	1	8.51E-07	1	0.333333333	Dpp6	Proteolysis
DMR4:4778801	4	4778801	800	2	1.72E-07	3	0.375		
DMR4:4806901	4	4806901	400	2	3.85E-09	3	0.75		
DMR4:4856601	4	4856601	500	1	3.17E-07	1	0.2		

DMR4:6760301	4	6760301	2100	1	1.08E-07	37	1.761904762	Prkag2	Signaling
DMR4:8277301	4	8277301	500	1	6.15E-07	4	0.8		
DMR4:8498801	4	8498801	300	1	8.50E-07	3	1	AABR07059201.1	
DMR4:9331701	4	9331701	2700	1	4.94E-07	23	0.851851852	SNORA17	
DMR4:9366701	4	9366701	1700	1	3.21E-07	22	1.294117647	Reln	Protease
DMR4:10501201	4	10501201	700	1	2.25E-08	10	1.428571429	Gsap	Receptor
DMR4:11061001	4	11061001	2700	1	5.90E-08	39	1.444444444	Magi2	Metabolism
DMR4:12069801	4	12069801	500	2	5.40E-08	0	0		
DMR4:12111201	4	12111201	700	1	2.98E-08	2	0.285714286		
DMR4:12464801	4	12464801	800	1	3.15E-08	1	0.125	AABR07059258.1	
DMR4:12690501	4	12690501	2200	2	5.74E-08	27	1.227272727		
DMR4:14170901	4	14170901	300	1	4.12E-07	1	0.333333333	Cd36	Immune
DMR4:14225901	4	14225901	300	1	1.02E-07	1	0.333333333	Cd36;LOC103690020	Immune
DMR4:15007701	4	15007701	500	2	4.26E-08	1	0.2		
DMR4:15675601	4	15675601	1600	1	8.10E-07	10	0.625		
DMR4:16066901	4	16066901	1400	2	5.02E-09	20	1.428571429	Cacna2d1	Metabolism
DMR4:16121501	4	16121501	2000	1	9.37E-07	19	0.95	Cacna2d1	Metabolism
DMR4:16312301	4	16312301	1900	1	9.29E-07	35	1.842105263		
DMR4:17634101	4	17634101	700	1	1.40E-07	6	0.857142857		
DMR4:17770101	4	17770101	600	1	7.29E-07	4	0.666666667		
DMR4:17811401	4	17811401	2700	1	7.41E-07	17	0.62962963	7SK	
DMR4:18650301	4	18650301	400	1	2.46E-07	3	0.75		
DMR4:18927701	4	18927701	2100	1	9.97E-09	10	0.476190476		
DMR4:19244001	4	19244001	800	2	2.79E-07	0	0	Sema3d	Growth Factors & Cytokines
DMR4:19289601	4	19289601	1300	1	1.23E-07	9	0.692307692	Sema3d	Growth Factors & Cytokines
DMR4:21657301	4	21657301	300	1	5.73E-07	0	0	RGD1563349	EST

DMR4:22087001	4	22087001	3500	1	1.82E-07	40	1.142857143	Crot	Metabolism
DMR4:22376901	4	22376901	600	1	2.52E-08	2	0.333333333	Abcb1a	Transport
DMR4:24576201	4	24576201	1400	2	8.27E-08	4	0.285714286		
DMR4:25821601	4	25821601	800	1	8.85E-07	10	1.25	Cdk14	Cell Cycle
DMR4:25892901	4	25892901	3700	1	1.74E-11	43	1.162162162	Cdk14	Cell Cycle
DMR4:26098501	4	26098501	300	1	9.70E-07	1	0.333333333	Cdk14	Cell Cycle
DMR4:26646901	4	26646901	1900	2	1.77E-12	9	0.473684211		
DMR4:27396601	4	27396601	300	2	5.60E-07	1	0.333333333	Lrrd1	
DMR4:27892001	4	27892001	300	1	6.35E-07	7	2.333333333	Cdk6	Cell Cycle
DMR4:28043101	4	28043101	700	2	5.27E-08	5	0.714285714		
DMR4:28155501	4	28155501	4400	2	1.30E-07	58	1.318181818		
DMR4:28817701	4	28817701	1200	1	6.40E-07	3	0.25		
DMR4:29086401	4	29086401	700	2	1.42E-11	6	0.857142857	Bet1	Golgi
DMR4:29447101	4	29447101	7000	6	2.91E-12	79	1.128571429		
DMR4:29478501	4	29478501	1200	1	1.35E-08	16	1.333333333		
DMR4:29569901	4	29569901	1600	1	8.32E-07	20	1.25	NEWGENE_621351	
DMR4:30505401	4	30505401	1700	2	4.55E-09	6	0.352941176	Asb4	Protein Binding
DMR4:30608101	4	30608101	900	1	6.84E-08	8	0.888888889		
DMR4:30695401	4	30695401	300	1	1.88E-08	2	0.666666667		
DMR4:30797701	4	30797701	2000	2	1.50E-09	15	0.75	Dync1i1	Cytoskeleton
DMR4:31145601	4	31145601	2200	1	8.99E-07	27	1.227272727		
DMR4:31575501	4	31575501	300	1	4.30E-08	0	0	NEWGENE_621351	
DMR4:31740601	4	31740601	300	2	1.26E-07	0	0		
DMR4:32634901	4	32634901	300	1	5.67E-07	0	0		
DMR4:32784901	4	32784901	1500	2	4.39E-07	9	0.6		
DMR4:34717801	4	34717801	300	1	1.78E-09	2	0.666666667	Ica1	Immune
DMR4:36132701	4	36132701	200	1	9.36E-09	1	0.5		
DMR4:37577401	4	37577401	100	1	1.10E-12	0	0		
DMR4:37725201	4	37725201	2800	1	8.58E-07	16	0.571428571		

DMR4:39090801	4	39090801	1400	1	7.17E-07	8	0.571428571	Thsd7a;5S_rRNA	Extracellular Matrix
DMR4:39218901	4	39218901	2600	1	2.58E-07	14	0.538461538		
DMR4:41240401	4	41240401	1100	1	7.00E-07	4	0.363636364		
DMR4:41881201	4	41881201	500	1	3.32E-07	6	1.2	Foxp2;AC115440.1	Transcription
DMR4:42282701	4	42282701	300	1	8.39E-07	3	1	Mdfic	Transcription
DMR4:42487901	4	42487901	500	1	2.33E-07	7	1.4		
DMR4:42963801	4	42963801	300	1	6.04E-09	0	0	Cttnbp2	Receptor
DMR4:44366901	4	44366901	1300	1	7.15E-08	10	0.769230769	Tes	Cytoskeleton
DMR4:44408301	4	44408301	300	1	4.91E-07	1	0.333333333		
DMR4:45487001	4	45487001	3200	1	1.38E-07	27	0.84375		
DMR4:46344401	4	46344401	300	1	5.36E-07	3	1		
DMR4:47029301	4	47029301	1300	1	3.59E-07	12	0.923076923		
DMR4:47986001	4	47986001	600	1	4.82E-08	0	0		
DMR4:48937401	4	48937401	3100	2	6.21E-08	39	1.258064516	Tspan12;AC116288.1	Cytoskeleton
DMR4:49087301	4	49087301	2300	1	1.02E-07	30	1.304347826	Cped1	
DMR4:49474601	4	49474601	1500	1	7.00E-08	6	0.4		
DMR4:50905801	4	50905801	400	1	3.36E-07	0	0		
DMR4:51768301	4	51768301	100	1	1.06E-08	0	0		
DMR4:51895501	4	51895501	700	1	7.29E-07	9	1.285714286	Pot1	Cell Cycle
DMR4:52343601	4	52343601	900	1	8.02E-12	3	0.333333333	Tmem229a	
DMR4:52709801	4	52709801	1100	1	9.35E-10	4	0.363636364		
DMR4:52752101	4	52752101	2800	1	1.48E-09	12	0.428571429		
DMR4:52870901	4	52870901	1200	1	4.29E-07	4	0.333333333		
DMR4:53559101	4	53559101	800	1	3.70E-07	1	0.125		
DMR4:54166301	4	54166301	300	2	7.98E-09	3	1		
DMR4:54304801	4	54304801	400	1	5.53E-07	4	1		
DMR4:55555401	4	55555401	13800	1	9.00E-07	223	1.615942029		

DMR4:56232401	4	56232401	400	1	9.24E-08	3	0.75		
DMR4:56455901	4	56455901	1100	1	6.42E-07	8	0.727272727	Prrt4	
DMR4:56668301	4	56668301	1400	1	5.25E-08	20	1.428571429	Ccdc136;AABR07072995.1	
DMR4:58530601	4	58530601	1400	1	3.43E-07	18	1.285714286		
DMR4:59395701	4	59395701	800	1	3.10E-07	5	0.625	AABR07060154.1	
DMR4:59435601	4	59435601	2100	1	7.16E-07	11	0.523809524	Plxna4	Receptor
DMR4:62168201	4	62168201	600	2	8.34E-07	2	0.333333333		
DMR4:62209201	4	62209201	3600	2	1.26E-07	50	1.388888889	AC103335.1;Cald1	Metabolism
DMR4:62472301	4	62472301	4500	1	4.53E-07	53	1.177777778	Stra8;RGD1565367	
DMR4:62524101	4	62524101	600	1	9.34E-07	5	0.833333333	RGD1565367	
DMR4:63695201	4	63695201	1300	1	9.84E-07	4	0.307692308	AABR07060235.1	
DMR4:63775201	4	63775201	400	1	5.66E-09	1	0.25		
DMR4:63830501	4	63830501	900	2	5.77E-10	2	0.222222222		
DMR4:64462801	4	64462801	900	1	6.87E-07	4	0.444444444	Dgki	Signaling
DMR4:64738601	4	64738601	600	2	5.91E-10	6	1	Dgki	Signaling
DMR4:65496201	4	65496201	400	1	3.31E-07	1	0.25		
DMR4:67097801	4	67097801	2500	1	2.88E-07	33	1.32	AABR07060318.1	
DMR4:67622101	4	67622101	800	3	5.67E-08	0	0		
DMR4:67842201	4	67842201	600	1	6.81E-07	0	0		
DMR4:68367001	4	68367001	3400	1	2.70E-07	17	0.5		
DMR4:69882401	4	69882401	2300	1	9.68E-07	18	0.782608696		
DMR4:70326201	4	70326201	1700	1	6.49E-07	15	0.882352941	Chl1	Extracellular Matrix
DMR4:70682501	4	70682501	1600	1	6.43E-09	9	0.5625	AC142181.1;Prss2	Proteolysis
DMR4:70731101	4	70731101	300	1	1.36E-07	3	1	AC142181.1	
DMR4:70858601	4	70858601	300	1	6.35E-12	0	0	U6	
DMR4:71948401	4	71948401	100	1	1.01E-20	0	0	Olr804	
DMR4:73307101	4	73307101	1000	1	7.52E-07	8	0.8		

DMR4:73733101	4	73733101	1500	1	5.52E-07	5	0.333333333		
DMR4:74407201	4	74407201	2200	2	2.50E-07	11	0.5		
DMR4:74868501	4	74868501	400	1	4.09E-07	2	0.5		
DMR4:76010701	4	76010701	500	2	4.90E-08	3	0.6	Cntnap2	Receptor
DMR4:76666901	4	76666901	700	1	7.70E-08	5	0.714285714	Cntnap2	Receptor
DMR4:77792901	4	77792901	300	1	1.97E-07	0	0		
DMR4:78444801	4	78444801	1000	1	1.04E-07	5	0.5	Tmem176b	Receptor
DMR4:78646301	4	78646301	300	1	4.25E-07	0	0	Svs1	
DMR4:78719101	4	78719101	3700	2	3.35E-10	32	0.864864865	Gpnmb	Cytoskeleton
DMR4:78836501	4	78836501	2300	1	2.92E-07	27	1.173913043	Igf2bp3	Transcription
DMR4:80338101	4	80338101	500	3	6.38E-08	1	0.2	LOC100363502;LOC500124	Binding Protein;EST
DMR4:81333901	4	81333901	1000	1	1.52E-07	12	1.2	Snx10	Transport
DMR4:82364401	4	82364401	1300	1	3.15E-09	9	0.692307692	NEWGENE_1565788	
DMR4:82500901	4	82500901	600	3	3.64E-08	0	0		
DMR4:82634901	4	82634901	500	2	1.69E-08	0	0	Hibadh;LOC685406	Metabolism
DMR4:82890501	4	82890501	700	1	2.46E-08	6	0.857142857	Jazf1	
DMR4:84314701	4	84314701	400	1	8.84E-09	1	0.25	Chn2	Signaling
DMR4:84523401	4	84523401	2300	3	5.89E-08	12	0.52173913		
DMR4:85309401	4	85309401	300	1	1.66E-07	3	1	Crhr2	Receptor
DMR4:85465601	4	85465601	3400	1	1.15E-07	70	2.058823529	Fam188b	
DMR4:85768601	4	85768601	600	2	8.37E-08	0	0		
DMR4:86155601	4	86155601	300	1	1.14E-11	0	0	Ccdc129	
DMR4:87139501	4	87139501	400	1	8.86E-08	1	0.25		
DMR4:87198801	4	87198801	800	2	1.51E-08	7	0.875	Fkbp9	Signaling
DMR4:87754001	4	87754001	100	1	1.32E-11	0	0	Vom1r73	Receptor
DMR4:87756101	4	87756101	200	1	3.94E-13	0	0	Vom1r73	Receptor
DMR4:88155601	4	88155601	6900	1	8.13E-07	91	1.31884058		

DMR4:88639001	4	88639001	500	1	8.29E-08	1	0.2	Herc6	Translation
DMR4:88664601	4	88664601	300	1	4.89E-07	7	2.333333333		
DMR4:89314301	4	89314301	900	1	9.17E-08	17	1.888888889		
DMR4:91569401	4	91569401	200	1	8.84E-07	2	1	Ccser1	
DMR4:92961601	4	92961601	600	1	2.63E-07	2	0.333333333		
DMR4:95314501	4	95314501	300	1	2.35E-08	1	0.333333333	Grid2	Signaling
DMR4:97793601	4	97793601	2400	1	8.75E-08	25	1.041666667	Gadd45a	Epigenetic
DMR4:97880601	4	97880601	1600	1	8.31E-08	11	0.6875		
DMR4:97953101	4	97953101	600	3	1.11E-07	4	0.666666667		
DMR4:98437101	4	98437101	1100	1	3.35E-07	4	0.363636364	AABR07060872.1	
DMR4:98525901	4	98525901	400	1	7.16E-07	2	0.5	AABR07060872.1;AABR07060886.1	
DMR4:99812401	4	99812401	800	3	3.67E-12	1	0.125	Ptcd3	Unknown
DMR4:99928601	4	99928601	700	1	4.50E-07	6	0.857142857	St3gal5	Metabolism
DMR4:100597001	4	1.01E+08	800	1	3.87E-07	12	1.5	Tcf7l1;Metazoa_SRP;AABR07060913.1	Transcription
DMR4:101188201	4	1.01E+08	2700	1	2.76E-07	20	0.740740741	Suclg1	Metabolism
DMR4:101280901	4	1.01E+08	2100	2	3.50E-09	16	0.761904762		
DMR4:105696101	4	1.06E+08	500	1	3.70E-08	2	0.4		
DMR4:106148201	4	1.06E+08	200	1	3.10E-08	1	0.5		
DMR4:106804201	4	1.07E+08	600	1	4.75E-08	8	1.333333333		
DMR4:107693101	4	1.08E+08	1200	1	3.19E-07	2	0.166666667		
DMR4:108568201	4	1.09E+08	2700	1	3.86E-07	16	0.592592593		
DMR4:109353901	4	1.09E+08	1400	2	1.56E-08	4	0.285714286		
DMR4:109681301	4	1.1E+08	1600	2	1.14E-07	10	0.625		
DMR4:110763701	4	1.11E+08	300	2	6.49E-09	2	0.666666667	Lrrtm4	Receptor
DMR4:111079501	4	1.11E+08	400	1	7.06E-07	1	0.25	Lrrtm4	Receptor
DMR4:112574901	4	1.13E+08	2600	1	4.84E-08	24	0.923076923	SNORA17	
DMR4:113453201	4	1.13E+08	500	1	4.02E-08	2	0.4		

DMR4:113615101	4	1.14E+08	1200	1	5.41E-08	4	0.333333333	Hk2	Metabolism
DMR4:115291401	4	1.15E+08	1400	1	1.10E-07	18	1.285714286		
DMR4:115412501	4	1.15E+08	2100	2	3.30E-10	17	0.80952381	Vax2;Atp6v1b1	Transcription;Transport
DMR4:115997001	4	1.16E+08	1900	1	8.70E-07	19	1		
DMR4:116084801	4	1.16E+08	1000	2	9.19E-08	14	1.4		
DMR4:116121501	4	1.16E+08	1000	2	1.09E-07	8	0.8		
DMR4:116205601	4	1.16E+08	1500	1	4.34E-07	8	0.533333333		
DMR4:116392901	4	1.16E+08	300	1	1.42E-07	2	0.666666667	Exoc6b	Transport
DMR4:116441201	4	1.16E+08	300	1	2.79E-07	2	0.666666667	Exoc6b	Transport
DMR4:116888101	4	1.17E+08	300	1	9.29E-08	2	0.666666667	SNORA17	
DMR4:118011001	4	1.18E+08	600	1	9.71E-09	4	0.666666667	Tgfa	Growth Factors & Cytokines
DMR4:118110501	4	1.18E+08	4200	2	1.89E-09	49	1.166666667		
DMR4:118117301	4	1.18E+08	1800	2	8.87E-09	10	0.555555556		
DMR4:118155701	4	1.18E+08	2200	1	5.45E-08	22	1	Fam136a;LOC687679	Transcription
DMR4:119133301	4	1.19E+08	600	1	1.95E-09	2	0.333333333	Antxr1;Gkn1	Receptor
DMR4:119310201	4	1.19E+08	400	2	1.05E-07	4	1	Arhgap25	Unknown
DMR4:120118401	4	1.2E+08	500	1	9.95E-07	5	1		
DMR4:121016501	4	1.21E+08	4100	1	1.40E-07	60	1.463414634		
DMR4:121499001	4	1.21E+08	400	2	5.89E-10	5	1.25	Chchd6;7SK	Transcription
DMR4:124359601	4	1.24E+08	1200	2	9.04E-10	6	0.5		
DMR4:124769501	4	1.25E+08	1700	4	5.92E-13	13	0.764705882	Adamts9	Proteolysis
DMR4:124943401	4	1.25E+08	800	2	7.47E-09	2	0.25		
DMR4:125040501	4	1.25E+08	300	1	6.79E-07	1	0.333333333		
DMR4:128197001	4	1.28E+08	1500	1	2.15E-07	9	0.6		
DMR4:128728901	4	1.29E+08	400	1	6.03E-07	1	0.25		
DMR4:129905201	4	1.3E+08	400	1	2.04E-07	4	1	SNORA63	

DMR4:130477401	4	1.3E+08	2200	5	5.52E-10	15	0.681818182		
DMR4:131331001	4	1.31E+08	1500	1	5.32E-07	16	1.066666667		
DMR4:132003901	4	1.32E+08	500	1	7.99E-07	0	0	7SK	
DMR4:132252501	4	1.32E+08	6800	2	1.67E-07	83	1.220588235		
DMR4:132421001	4	1.32E+08	700	2	1.39E-07	5	0.714285714	AABR07061535.1	
DMR4:132936001	4	1.33E+08	1400	2	5.84E-08	9	0.642857143		
DMR4:132967901	4	1.33E+08	1600	1	8.79E-09	11	0.6875		
DMR4:133361201	4	1.33E+08	3800	1	7.13E-07	36	0.947368421		
DMR4:134237901	4	1.34E+08	1200	1	5.05E-08	14	1.166666667		
DMR4:135190201	4	1.35E+08	400	1	1.16E-07	0	0	7SK	
DMR4:138348701	4	1.38E+08	300	1	1.65E-08	3	1	Cntn4	Cytoskeleton
DMR4:142714801	4	1.43E+08	2100	1	6.87E-07	25	1.19047619		
DMR4:143416001	4	1.43E+08	500	2	4.39E-09	1	0.2	U6	
DMR4:143456901	4	1.43E+08	1400	1	1.69E-10	11	0.785714286		
DMR4:144071301	4	1.44E+08	4000	1	2.90E-10	23	0.575		
DMR4:144162901	4	1.44E+08	400	1	3.22E-07	0	0		
DMR4:145925401	4	1.46E+08	700	2	3.39E-10	8	1.142857143	Atp2b2	Metabolism
DMR4:146258101	4	1.46E+08	800	1	2.24E-07	11	1.375		
DMR4:146296801	4	1.46E+08	300	2	4.76E-07	7	2.333333333	Slc6a1;LOC102548867;AABR07061807.1;AABR07061807.2	Transport
DMR4:146511801	4	1.47E+08	300	1	2.94E-08	1	0.333333333		
DMR4:146793901	4	1.47E+08	1700	2	1.76E-07	18	1.058823529	Vgll4	
DMR4:147108901	4	1.47E+08	400	1	2.27E-07	2	0.5	Syn2	Cytoskeleton
DMR4:147433901	4	1.47E+08	3400	3	2.27E-08	54	1.588235294		
DMR4:148677901	4	1.49E+08	5300	1	1.79E-08	42	0.79245283	Olr831	
DMR4:148686201	4	1.49E+08	1100	1	1.88E-07	12	1.090909091	Olr831	
DMR4:148810301	4	1.49E+08	600	1	6.60E-07	3	0.5	Rassf4;Tmem72	Signaling;Unknown

DMR4:148919401	4	1.49E+08	2500	1	1.30E-09	23	0.92		
DMR4:150258201	4	1.5E+08	200	1	2.56E-07	1	0.5		
DMR4:150268401	4	1.5E+08	1000	1	4.28E-07	10	1		
DMR4:151211501	4	1.51E+08	900	1	4.41E-07	10	1.111111111		
DMR4:151324301	4	1.51E+08	4500	2	3.32E-08	58	1.288888889	Cacna2d4	Transport
DMR4:151658701	4	1.52E+08	500	1	6.36E-08	1	0.2		
DMR4:152703801	4	1.53E+08	400	2	4.33E-09	0	0	Ninj2	Extracellular Matrix
DMR4:152843401	4	1.53E+08	400	1	1.86E-07	8	2	B4galnt3	Metabolism
DMR4:153723901	4	1.54E+08	300	2	1.14E-08	1	0.333333333		
DMR4:153996201	4	1.54E+08	2700	1	8.76E-09	36	1.333333333	Iqsec3	Signaling
DMR4:154018601	4	1.54E+08	400	2	1.02E-07	0	0	Iqsec3	Signaling
DMR4:154554301	4	1.55E+08	700	1	5.11E-10	4	0.571428571	Mug1	Immune
DMR4:154586301	4	1.55E+08	1000	2	7.21E-07	5	0.5	Mug1	Immune
DMR4:154625001	4	1.55E+08	300	1	1.63E-07	2	0.666666667	Mug1	Immune
DMR4:154715501	4	1.55E+08	700	2	2.75E-08	6	0.857142857	Mug1	Immune
DMR4:155569001	4	1.56E+08	900	2	1.99E-09	1	0.111111111	Slc2a3	Transport
DMR4:155670501	4	1.56E+08	500	1	3.50E-07	2	0.4	Foxj2	Transcription
DMR4:155767701	4	1.56E+08	2600	3	2.01E-08	38	1.461538462	LOC100909595	Transport
DMR4:155814801	4	1.56E+08	2100	1	3.59E-07	15	0.714285714	Clec4a2	Receptor
DMR4:155966701	4	1.56E+08	4600	2	4.65E-08	33	0.717391304	Clec4a1	Cytoskeleton
DMR4:157148201	4	1.57E+08	2000	1	4.12E-09	26	1.3	C1s	Immune
DMR4:157157101	4	1.57E+08	300	1	4.54E-08	2	0.666666667	C1s	Immune
DMR4:157930501	4	1.58E+08	3300	2	4.04E-09	38	1.151515152	U6	
DMR4:158337201	4	1.58E+08	3100	1	3.79E-09	43	1.387096774	Ano2	
DMR4:158614201	4	1.59E+08	400	2	4.55E-09	1	0.25		
DMR4:158737401	4	1.59E+08	700	1	4.45E-08	3	0.428571429		
DMR4:159557601	4	1.6E+08	400	2	8.03E-08	2	0.5	Fgf6	Signaling
DMR4:159807701	4	1.6E+08	2600	1	1.77E-07	41	1.576923077		

DMR4:161049901	4	1.61E+08	300	2	1.68E-08	2	0.666666667	Senp17	Proteolysis
DMR4:161272701	4	1.61E+08	400	2	1.66E-08	3	0.75	AABR07062108.1	
DMR4:161517901	4	1.62E+08	900	1	1.85E-07	11	1.222222222	LOC689519;AABR07062112.1	
DMR4:161857801	4	1.62E+08	300	1	3.67E-07	1	0.333333333	Pzp;A2ml1	Development
DMR4:161890101	4	1.62E+08	800	1	7.08E-07	8	1	A2ml1	
DMR4:162813801	4	1.63E+08	1200	1	1.75E-07	1	0.083333333	AABR07062154.2	
DMR4:163349501	4	1.63E+08	1100	1	4.45E-07	7	0.636363636	Klre1;Klrd1	Receptor
DMR4:164069001	4	1.64E+08	600	1	1.60E-07	3	0.5		
DMR4:164647201	4	1.65E+08	2900	1	2.05E-08	12	0.413793103	Ly49s4;Ly49i2	Receptor
DMR4:165447701	4	1.65E+08	1100	1	3.20E-07	10	0.909090909	Klra2	Immune
DMR4:165759801	4	1.66E+08	300	1	7.52E-07	0	0		
DMR4:165796301	4	1.66E+08	900	1	5.44E-07	10	1.111111111	Tas2r106	
DMR4:165843101	4	1.66E+08	700	2	8.81E-09	3	0.428571429		
DMR4:165953801	4	1.66E+08	3300	1	2.74E-07	35	1.060606061		
DMR4:167415101	4	1.67E+08	4200	1	8.58E-07	36	0.857142857	Grpcb	
DMR4:167603301	4	1.68E+08	2300	1	1.45E-08	13	0.565217391		
DMR4:167624401	4	1.68E+08	1100	1	8.65E-07	11	1		
DMR4:167957901	4	1.68E+08	500	1	2.22E-07	9	1.8	Etv6	Transcription
DMR4:168539501	4	1.69E+08	200	1	4.90E-07	0	0		
DMR4:168715601	4	1.69E+08	500	1	3.75E-07	8	1.6		
DMR4:169949701	4	1.7E+08	2600	1	3.04E-10	40	1.538461538	Grin2b	Receptor
DMR4:170725501	4	1.71E+08	4400	2	1.08E-07	57	1.295454545	Gucy2c	Metabolism
DMR4:171633901	4	1.72E+08	1200	2	1.02E-09	8	0.666666667		
DMR4:171897501	4	1.72E+08	400	1	1.68E-07	1	0.25		
DMR4:172140101	4	1.72E+08	500	3	1.02E-09	0	0	Mgst1	Metabolism
DMR4:173643601	4	1.74E+08	1100	1	1.87E-07	10	0.909090909	Rergl	Signaling
DMR4:173671101	4	1.74E+08	1100	3	1.38E-10	2	0.181818182		
DMR4:173843601	4	1.74E+08	1500	1	5.27E-10	10	0.666666667	Pik3c2g	Signaling

DMR4:174512301	4	1.75E+08	1100	1	7.65E-07	1	0.090909091		
DMR4:174861001	4	1.75E+08	2000	3	1.81E-09	19	0.95	Aebp2	Transcription
DMR4:174929401	4	1.75E+08	300	2	5.73E-09	3	1		
DMR4:176352701	4	1.76E+08	700	1	7.81E-10	3	0.428571429	Slco1a6	
DMR4:176450901	4	1.76E+08	500	1	2.79E-07	0	0	Slco1a2;7SK	Transport
DMR4:176559401	4	1.77E+08	500	1	9.21E-07	9	1.8	Pyroxd1	Metabolism
DMR4:176637301	4	1.77E+08	600	2	2.51E-11	0	0	Spx;Gys2	Metabolism
DMR4:178524601	4	1.79E+08	300	1	2.13E-07	1	0.333333333		
DMR4:178599701	4	1.79E+08	4800	1	1.14E-07	77	1.604166667		
DMR4:179636001	4	1.8E+08	1900	1	4.01E-08	55	2.894736842		
DMR4:180316601	4	1.8E+08	1100	1	6.45E-09	7	0.636363636	Sspn	Development
DMR4:180340001	4	1.8E+08	2000	1	6.06E-07	15	0.75		
DMR4:180599401	4	1.81E+08	1400	1	3.68E-07	8	0.571428571	Itpr2	Receptor
DMR4:180734201	4	1.81E+08	1600	2	1.86E-07	17	1.0625		
DMR4:181248101	4	1.81E+08	400	1	1.24E-08	6	1.5	Smco2	
DMR4:181528801	4	1.82E+08	2500	1	9.99E-09	14	0.56	AABR07062535.2	
DMR4:181701101	4	1.82E+08	1800	1	1.19E-08	20	1.111111111	AABR07062539.3	
DMR4:181764401	4	1.82E+08	600	1	1.55E-07	3	0.5		
DMR4:182256701	4	1.82E+08	1900	1	4.82E-07	0	0		
DMR4:182549601	4	1.83E+08	2000	1	5.37E-08	19	0.95	Far2	Metabolism
DMR4:183093301	4	1.83E+08	400	1	5.59E-08	3	0.75		
DMR5:491301	5	491301	5500	1	5.48E-08	56	1.018181818		
DMR5:2219501	5	2219501	1400	2	1.44E-09	7	0.5		
DMR5:2821701	5	2821701	1100	3	4.81E-10	7	0.636363636	Sbspon;7SK	
DMR5:4013701	5	4013701	2100	4	6.32E-12	2	0.095238095		
DMR5:4258301	5	4258301	1300	1	3.85E-08	7	0.538461538		
DMR5:4505901	5	4505901	3200	2	8.49E-07	20	0.625	Eya1	Metabolism
DMR5:4930301	5	4930301	300	1	1.94E-07	1	0.333333333	AC127076.1	
DMR5:5393901	5	5393901	2300	1	3.68E-08	30	1.304347826		

DMR5:5828701	5	5828701	400	2	9.67E-08	0	0	U6	
DMR5:6372801	5	6372801	2100	1	9.30E-07	35	1.666666667	AABR07046778.1	
DMR5:7223101	5	7223101	1400	1	3.91E-07	6	0.428571429	RGD1564053	Unknown
DMR5:7241101	5	7241101	400	1	8.29E-07	1	0.25	RGD1564053	Unknown
DMR5:7477401	5	7477401	1500	1	3.21E-07	18	1.2		
DMR5:7932701	5	7932701	600	1	3.88E-08	4	0.666666667	Prex2	Signaling
DMR5:9939901	5	9939901	400	1	1.03E-07	4	1		
DMR5:10018301	5	10018301	1300	1	8.40E-07	4	0.307692308		
DMR5:10169601	5	10169601	800	1	1.23E-07	3	0.375	Sntg1	Development
DMR5:10374501	5	10374501	600	2	1.28E-08	2	0.333333333	Sntg1	Development
DMR5:14644401	5	14644401	400	2	2.95E-07	1	0.25		
DMR5:14772801	5	14772801	400	1	1.40E-07	0	0		
DMR5:15704801	5	15704801	500	3	1.20E-14	2	0.4		
DMR5:16553401	5	16553401	1300	1	2.37E-11	10	0.769230769	Lyn	Transcription
DMR5:16714301	5	16714301	1100	3	6.13E-10	1	0.090909091	Rps20;snoU54	Translation
DMR5:16907201	5	16907201	900	1	2.25E-07	3	0.333333333	Sdr16c5	Metabolism
DMR5:17716801	5	17716801	500	2	1.47E-10	1	0.2		
DMR5:18985601	5	18985601	700	1	6.90E-07	5	0.714285714		
DMR5:21043801	5	21043801	300	1	2.67E-07	15	5	Gm5471	
DMR5:21518501	5	21518501	1600	3	1.78E-08	31	1.9375		
DMR5:22323301	5	22323301	1700	2	1.99E-09	10	0.588235294	U6	
DMR5:22414001	5	22414001	1100	1	1.84E-07	15	1.363636364	Clvs1	
DMR5:23671401	5	23671401	1100	1	1.78E-08	4	0.363636364		
DMR5:25838201	5	25838201	300	1	1.22E-08	2	0.666666667		
DMR5:26068701	5	26068701	1600	1	1.77E-07	14	0.875		
DMR5:27980501	5	27980501	600	1	1.90E-08	0	0	Slc26a7	Transport
DMR5:28242601	5	28242601	1000	1	8.21E-09	9	0.9	Lrrc69	
DMR5:28434301	5	28434301	300	1	7.79E-07	1	0.333333333	Tmem55a;Rn60_5_0285.4	Unknown

DMR5:28662201	5	28662201	4000	1	6.72E-07	56	1.4	Necab1	
DMR5:28725601	5	28725601	1400	1	3.38E-08	6	0.428571429	Necab1	
DMR5:29177701	5	29177701	300	1	6.42E-08	2	0.666666667		
DMR5:29193601	5	29193601	1300	1	2.33E-07	3	0.230769231		
DMR5:29717301	5	29717301	400	1	2.16E-07	3	0.75		
DMR5:29870501	5	29870501	300	1	7.26E-07	19	6.333333333	Ripk2	Signaling
DMR5:32382001	5	32382001	300	1	9.04E-07	2	0.666666667		
DMR5:32754001	5	32754001	1700	1	1.24E-07	6	0.352941176	Cnbd1	
DMR5:33186601	5	33186601	700	2	4.69E-09	4	0.571428571	Cngb3;Maged2	Receptor;Extracellular Matrix
DMR5:34586801	5	34586801	400	1	1.71E-08	1	0.25	Nkain3	Transport
DMR5:35136001	5	35136001	700	3	3.33E-10	0	0		
DMR5:35361901	5	35361901	2900	2	5.72E-11	26	0.896551724		
DMR5:35366401	5	35366401	2100	1	1.45E-08	31	1.476190476		
DMR5:35785601	5	35785601	500	1	1.75E-07	1	0.2		
DMR5:36440301	5	36440301	1800	2	5.95E-16	9	0.5	AABR07047528.1	
DMR5:36480801	5	36480801	600	1	3.83E-08	5	0.833333333		
DMR5:36846501	5	36846501	600	1	2.29E-11	6	1		
DMR5:39989201	5	39989201	200	1	9.09E-10	3	1.5		
DMR5:41827301	5	41827301	500	1	2.30E-07	0	0		
DMR5:42462001	5	42462001	2800	1	1.42E-08	14	0.5		
DMR5:43771401	5	43771401	1000	1	3.20E-09	5	0.5		
DMR5:44051601	5	44051601	4100	1	3.82E-08	48	1.170731707		
DMR5:44249601	5	44249601	700	1	7.13E-07	3	0.428571429		
DMR5:44965001	5	44965001	900	2	5.69E-10	3	0.333333333		
DMR5:45650401	5	45650401	100	1	8.19E-14	1	1		
DMR5:45695601	5	45695601	2800	1	7.60E-07	28	1		
DMR5:46213801	5	46213801	2000	1	3.54E-08	15	0.75		
DMR5:46543301	5	46543301	2600	1	3.42E-07	26	1		

DMR5:47345901	5	47345901	4100	1	2.10E-07	52	1.268292683		
DMR5:48135901	5	48135901	2100	1	7.75E-07	27	1.285714286	Ankrd6	
DMR5:48652201	5	48652201	2700	1	4.69E-07	16	0.592592593	Rngtt	Translation
DMR5:49014201	5	49014201	600	2	1.95E-09	3	0.5		
DMR5:49815401	5	49815401	2100	1	5.20E-07	56	2.666666667	AABR07047835.1	
DMR5:49909101	5	49909101	4600	2	5.21E-09	40	0.869565217		
DMR5:50257901	5	50257901	2900	1	1.42E-08	26	0.896551724	SNORA17	
DMR5:50537401	5	50537401	500	1	3.29E-09	2	0.4	Mob3b	Signaling
DMR5:52017501	5	52017501	400	1	1.07E-07	3	0.75		
DMR5:52084701	5	52084701	900	2	1.72E-07	3	0.333333333		
DMR5:52156001	5	52156001	600	1	6.68E-07	2	0.333333333		
DMR5:52809301	5	52809301	1500	1	1.48E-08	4	0.266666667		
DMR5:53289401	5	53289401	400	2	5.12E-10	0	0		
DMR5:53789101	5	53789101	600	2	5.57E-08	11	1.833333333		
DMR5:53827601	5	53827601	1400	1	1.93E-07	15	1.071428571		
DMR5:54409401	5	54409401	300	1	1.01E-07	2	0.666666667		
DMR5:54624601	5	54624601	1100	1	3.16E-07	7	0.636363636		
DMR5:55137601	5	55137601	800	1	2.41E-07	7	0.875		
DMR5:58068401	5	58068401	300	1	5.79E-07	7	2.333333333	Cntfr	Receptor
DMR5:58234201	5	58234201	900	1	1.71E-07	9	1		
DMR5:58446901	5	58446901	200	1	1.98E-08	1	0.5	Vcp;Fancg	Cytoskeleton
DMR5:58462101	5	58462101	900	3	6.55E-10	3	0.333333333	Fancg;Pigo;Stoml2	Metabolism;Cytoskeleton
DMR5:58662901	5	58662901	300	1	5.96E-07	3	1	Unc13b	Receptor
DMR5:59429001	5	59429001	2100	2	8.17E-12	26	1.238095238	Glipr2	
DMR5:59882701	5	59882701	2400	1	8.51E-08	27	1.125	SNORA17	
DMR5:59901201	5	59901201	2600	2	2.57E-12	35	1.346153846	LOC100359916	Unknown
DMR5:60198301	5	60198301	3500	1	5.82E-08	42	1.2	Pax5	Transcription
DMR5:60342801	5	60342801	700	2	3.24E-13	2	0.285714286	Zcchc7	Transcription

DMR5:60519401	5	60519401	1300	1	6.24E-07	13	1	AABR07048075.1;AABR07048075.2;Grhpr	
DMR5:60727901	5	60727901	300	1	4.74E-09	2	0.666666667		
DMR5:61095001	5	61095001	1600	1	3.57E-08	19	1.1875		
DMR5:61183001	5	61183001	3800	2	1.85E-09	42	1.105263158		
DMR5:61497401	5	61497401	2900	1	1.91E-07	41	1.413793103	Ccdc180	
DMR5:61650601	5	61650601	300	1	3.68E-07	7	2.333333333	Tmod1	Cytoskeleton
DMR5:62116601	5	62116601	1900	1	1.79E-07	26	1.368421053	Nans	Metabolism
DMR5:62792501	5	62792501	600	1	9.44E-07	2	0.333333333		
DMR5:62872001	5	62872001	2100	2	3.16E-10	26	1.238095238	Col15a1	Extracellular Matrix
DMR5:63221001	5	63221001	3600	1	1.88E-08	47	1.305555556		
DMR5:63294901	5	63294901	600	1	3.95E-08	3	0.5		
DMR5:63459401	5	63459401	900	2	3.11E-09	7	0.777777778		
DMR5:63668101	5	63668101	400	1	5.71E-07	2	0.5		
DMR5:63771801	5	63771801	1400	2	7.47E-10	12	0.857142857	Nr4a3	Transcription
DMR5:64470101	5	64470101	2400	1	7.27E-07	28	1.166666667	LOC108348074	
DMR5:64675101	5	64675101	900	4	3.83E-10	5	0.555555556	Acnat2	
DMR5:64704101	5	64704101	1100	2	1.46E-08	7	0.636363636	Acnat2;Acnat1	
DMR5:64738101	5	64738101	1900	1	1.49E-07	10	0.526315789		
DMR5:66399401	5	66399401	300	1	6.67E-07	0	0		
DMR5:67259401	5	67259401	1400	1	8.33E-07	4	0.285714286		
DMR5:67911301	5	67911301	1300	1	1.04E-07	18	1.384615385	Plppr1	
DMR5:68458601	5	68458601	2900	1	4.30E-07	19	0.655172414		
DMR5:68982301	5	68982301	400	1	4.55E-07	1	0.25		
DMR5:69098101	5	69098101	2000	1	2.50E-07	20	1	AABR07048253.1	
DMR5:70128301	5	70128301	700	1	7.51E-07	2	0.285714286		
DMR5:70435401	5	70435401	3500	1	5.79E-07	64	1.828571429	Fsd1l	

DMR5:70801101	5	70801101	500	1	7.03E-07	0	0		
DMR5:71166501	5	71166501	1000	1	2.66E-07	1	0.1		
DMR5:71591301	5	71591301	1600	1	4.79E-07	9	0.5625		
DMR5:71843101	5	71843101	900	1	8.37E-07	6	0.666666667		
DMR5:72436901	5	72436901	400	1	7.57E-07	3	0.75		
DMR5:72560701	5	72560701	700	1	4.93E-07	2	0.285714286		
DMR5:72935001	5	72935001	2300	1	2.14E-07	8	0.347826087	AABR07048321.1	
DMR5:74218001	5	74218001	1600	1	5.24E-07	15	0.9375		
DMR5:74636401	5	74636401	200	1	7.65E-07	0	0		
DMR5:75673501	5	75673501	2800	1	2.02E-08	38	1.357142857	Lpar1;AABR07048379.1	Receptor
DMR5:75915101	5	75915101	300	1	7.13E-07	2	0.666666667	Olr854	Receptor
DMR5:76121101	5	76121101	400	1	7.21E-07	0	0	Ptgr1	Metabolism
DMR5:77667801	5	77667801	5300	1	4.32E-07	90	1.698113208	LOC100912565;Rn50_5_0814.4	Immune
DMR5:77679601	5	77679601	6800	1	7.15E-07	74	1.088235294	LOC100912565;Rn50_5_0814.4	Immune
DMR5:78423901	5	78423901	1100	1	3.35E-08	9	0.818181818	Rgs3	
DMR5:78461201	5	78461201	1900	2	6.42E-08	17	0.894736842	Rgs3	
DMR5:78749101	5	78749101	1200	2	2.84E-08	16	1.333333333		
DMR5:79285301	5	79285301	3000	1	5.50E-08	28	0.933333333	Whrn	Cytoskeleton
DMR5:80001001	5	80001001	200	1	2.37E-07	1	0.5		
DMR5:80681301	5	80681301	300	1	7.55E-08	0	0		
DMR5:80684101	5	80684101	500	1	5.84E-08	1	0.2		
DMR5:81228501	5	81228501	800	2	1.23E-09	2	0.25	Astn2	Unknown
DMR5:82442601	5	82442601	1300	1	2.08E-08	4	0.307692308		
DMR5:83920001	5	83920001	600	2	7.41E-08	0	0		
DMR5:86819701	5	86819701	2100	1	5.67E-08	6	0.285714286		
DMR5:87869901	5	87869901	300	1	7.48E-08	0	0		

DMR5:88048601	5	88048601	800	1	4.96E-08	7	0.875		
DMR5:89160801	5	89160801	800	1	3.54E-08	0	0		
DMR5:90868101	5	90868101	2000	1	6.60E-08	16	0.8	Kdm4c	Epigenetic
DMR5:91585501	5	91585501	500	2	7.76E-09	0	0		
DMR5:95341401	5	95341401	3900	1	3.14E-07	53	1.358974359	AABR07048878.1	
DMR5:95388401	5	95388401	700	2	1.53E-09	4	0.571428571	AABR07048878.1	
DMR5:95682501	5	95682501	1000	1	9.49E-08	2	0.2		
DMR5:96100801	5	96100801	1800	1	3.04E-09	20	1.111111111		
DMR5:97588901	5	97588901	1800	1	2.27E-07	7	0.388888889		
DMR5:98488401	5	98488401	400	2	3.69E-08	2	0.5	Lurap1l	
DMR5:98840301	5	98840301	4000	1	7.44E-07	39	0.975	AABR07048977.1	
DMR5:99171601	5	99171601	600	2	1.31E-10	1	0.166666667	RGD1306186	EST
DMR5:99920001	5	99920001	1400	1	4.41E-07	9	0.642857143	U4	
DMR5:100389801	5	1E+08	2500	3	7.09E-08	34	1.36		
DMR5:100865401	5	1.01E+08	900	1	3.21E-10	3	0.333333333	AABR07049035.1	
DMR5:100889501	5	1.01E+08	2300	3	2.23E-15	26	1.130434783	AABR07049035.1	
DMR5:101775001	5	1.02E+08	2400	2	2.93E-08	34	1.416666667	Ccdc171	
DMR5:102239401	5	1.02E+08	700	1	1.51E-07	5	0.714285714		
DMR5:102290201	5	1.02E+08	200	1	8.58E-07	3	1.5		
DMR5:103814501	5	1.04E+08	400	1	3.26E-08	0	0		
DMR5:103919801	5	1.04E+08	1200	1	2.43E-07	9	0.75		
DMR5:104134401	5	1.04E+08	900	2	1.07E-08	8	0.888888889		
DMR5:104414301	5	1.04E+08	400	2	2.29E-08	3	0.75	Adamtsl1	Extracellular Matrix
DMR5:106376001	5	1.06E+08	1500	1	4.15E-07	5	0.333333333		
DMR5:106420701	5	1.06E+08	300	1	2.77E-07	1	0.333333333	Focad;7SK	
DMR5:106471901	5	1.06E+08	1500	2	1.79E-08	10	0.666666667	Focad	
DMR5:107157401	5	1.07E+08	1300	2	1.71E-12	7	0.538461538		
DMR5:107301301	5	1.07E+08	1800	2	6.44E-07	16	0.888888889	AABR07049134.4	

DMR5:107992701	5	1.08E+08	300	1	1.93E-07	2	0.666666667		
DMR5:109220401	5	1.09E+08	600	1	9.85E-07	3	0.5	AABR07049190.1	
DMR5:109753301	5	1.1E+08	300	1	7.68E-07	1	0.333333333		
DMR5:110875701	5	1.11E+08	1800	3	1.70E-12	15	0.833333333		
DMR5:110991301	5	1.11E+08	1700	1	4.30E-10	18	1.058823529		
DMR5:112371801	5	1.12E+08	1200	1	4.24E-07	12	1		
DMR5:114167601	5	1.14E+08	500	1	1.10E-10	2	0.4		
DMR5:114555801	5	1.15E+08	1300	1	5.20E-08	5	0.384615385	Fggy	Signaling
DMR5:114817701	5	1.15E+08	600	2	4.03E-09	1	0.166666667	Fggy	Signaling
DMR5:116614301	5	1.17E+08	1500	1	8.10E-07	22	1.466666667	Nfia	Transcription
DMR5:116976101	5	1.17E+08	400	1	5.87E-07	9	2.25	LOC100912024	
DMR5:119319401	5	1.19E+08	1600	1	9.85E-07	11	0.6875		
DMR5:119857101	5	1.2E+08	400	1	2.07E-09	0	0		
DMR5:120483701	5	1.2E+08	1200	1	9.74E-07	16	1.333333333	Dnajc6	Protein Binding
DMR5:120704701	5	1.21E+08	4300	1	1.59E-09	49	1.139534884		
DMR5:121388901	5	1.21E+08	1200	1	4.81E-08	18	1.5		
DMR5:122149801	5	1.22E+08	300	1	5.20E-09	1	0.333333333		
DMR5:122168401	5	1.22E+08	600	2	1.69E-09	3	0.5		
DMR5:123113301	5	1.23E+08	400	1	4.22E-07	1	0.25		
DMR5:123244101	5	1.23E+08	400	1	7.38E-09	1	0.25		
DMR5:123860401	5	1.24E+08	6300	1	6.00E-07	90	1.428571429		
DMR5:123941101	5	1.24E+08	1900	1	6.14E-07	8	0.421052632	Dab1	Signaling
DMR5:124771201	5	1.25E+08	1100	1	1.03E-07	19	1.727272727	Plpp3;AABR07049503.1	
DMR5:124954501	5	1.25E+08	2500	2	3.81E-07	28	1.12		
DMR5:125663801	5	1.26E+08	2000	2	2.96E-07	20	1		
DMR5:125867701	5	1.26E+08	2100	1	9.33E-07	15	0.714285714	AABR07049524.1	
DMR5:126295001	5	1.26E+08	1300	1	5.29E-07	15	1.153846154	Mroh7	
DMR5:126957601	5	1.27E+08	800	1	3.64E-08	8	1	Yipf1	

DMR5:128287501	5	1.28E+08	900	1	2.09E-07	4	0.444444444	Zfyve9	Transcription
DMR5:128860801	5	1.29E+08	1800	3	2.07E-08	9	0.5	Calr4	
DMR5:129720501	5	1.3E+08	800	3	1.64E-08	0	0	Faf1	Apoptosis
DMR5:130710801	5	1.31E+08	500	1	7.47E-09	3	0.6		
DMR5:130764301	5	1.31E+08	1200	2	8.79E-09	7	0.583333333		
DMR5:132783501	5	1.33E+08	400	2	2.31E-11	0	0	AABR07049682.1	
DMR5:133240701	5	1.33E+08	600	1	8.48E-09	8	1.333333333	Trabd2b	
DMR5:133674301	5	1.34E+08	1400	2	1.60E-09	17	1.214285714		
DMR5:134040901	5	1.34E+08	300	1	2.40E-08	0	0		
DMR5:134160801	5	1.34E+08	800	1	2.57E-08	3	0.375		
DMR5:134227001	5	1.34E+08	5100	1	6.12E-07	64	1.254901961		
DMR5:134431201	5	1.34E+08	600	1	8.72E-07	3	0.5		
DMR5:136859901	5	1.37E+08	600	2	1.62E-08	3	0.5	St3gal3	Metabolism
DMR5:136928401	5	1.37E+08	4500	3	5.16E-09	62	1.377777778	St3gal3	Metabolism
DMR5:137958601	5	1.38E+08	3200	1	1.23E-07	26	0.8125		
DMR5:138190901	5	1.38E+08	2700	2	2.82E-07	42	1.555555556	Slc2a1	Metabolism
DMR5:138485501	5	1.38E+08	1600	1	7.07E-07	15	0.9375	Zmynd12	Transcription
DMR5:138503401	5	1.39E+08	200	1	6.12E-07	1	0.5	Zmynd12	Transcription
DMR5:138697901	5	1.39E+08	6800	10	3.01E-13	86	1.264705882	Guca2b	Signaling
DMR5:138739901	5	1.39E+08	200	1	9.43E-07	3	1.5		
DMR5:139165901	5	1.39E+08	1900	1	3.39E-09	19	1		
DMR5:139516701	5	1.4E+08	3700	1	8.36E-09	43	1.162162162		
DMR5:141427201	5	1.41E+08	500	1	1.99E-07	3	0.6	Akirin1	Signaling
DMR5:141617301	5	1.42E+08	1200	2	4.54E-08	10	0.833333333		
DMR5:141718601	5	1.42E+08	4000	1	9.56E-09	48	1.2		
DMR5:141982101	5	1.42E+08	2400	1	1.28E-07	35	1.458333333		
DMR5:141989701	5	1.42E+08	4100	1	3.49E-07	49	1.195121951		
DMR5:142257201	5	1.42E+08	700	1	1.97E-08	17	2.428571429		
DMR5:142541801	5	1.43E+08	600	1	3.32E-07	3	0.5		

DMR5:143125001	5	1.43E+08	700	2	1.79E-08	4	0.571428571	Zc3h12a	Transcription
DMR5:144295301	5	1.44E+08	2600	1	5.51E-08	20	0.769230769	Trappc3	
DMR5:144653201	5	1.45E+08	400	1	1.68E-13	3	0.75		
DMR5:144975901	5	1.45E+08	1400	2	6.42E-08	5	0.357142857	Zmym4	Transcription
DMR5:145342601	5	1.45E+08	3600	1	6.62E-09	48	1.333333333	U6	
DMR5:145759301	5	1.46E+08	500	3	2.48E-12	3	0.6		
DMR5:145821901	5	1.46E+08	800	1	2.26E-08	1	0.125		
DMR5:146120801	5	1.46E+08	300	1	2.45E-07	5	1.666666667		
DMR5:146354601	5	1.46E+08	500	1	9.37E-08	9	1.8	AABR07049962.1	
DMR5:146425401	5	1.46E+08	300	1	2.08E-10	0	0	Csmd2	Unknown
DMR5:148204201	5	1.48E+08	1800	1	5.43E-08	19	1.055555556	Adgrb2	
DMR5:148304301	5	1.48E+08	2100	2	2.41E-07	21	1	Col16a1	Extracellular Matrix
DMR5:148504601	5	1.49E+08	1500	3	2.54E-09	15	1	AABR07050017.2;AABR07050017.1;SCARNA16	
DMR5:148540601	5	1.49E+08	1800	1	9.69E-07	25	1.388888889	Fabp3;Zcchc17	Binding Protein;Receptor
DMR5:148935101	5	1.49E+08	1300	1	1.05E-07	14	1.076923077	Sdc3	Signaling
DMR5:149225801	5	1.49E+08	1400	1	9.47E-07	19	1.357142857		
DMR5:149238001	5	1.49E+08	2800	1	4.15E-08	30	1.071428571		
DMR5:149313501	5	1.49E+08	1500	1	1.95E-07	13	0.866666667		
DMR5:149745001	5	1.5E+08	1100	2	2.20E-08	12	1.090909091		
DMR5:149939201	5	1.5E+08	3100	1	2.56E-09	62	2	Ptpru	Signaling
DMR5:149984801	5	1.5E+08	1700	1	2.11E-07	27	1.588235294	Ptpru	Signaling
DMR5:150613301	5	1.51E+08	1200	3	6.89E-10	11	0.916666667	Phactr4	Signaling
DMR5:151001701	5	1.51E+08	3400	1	7.84E-07	39	1.147058824	RGD1561465;Ppp1r8	Transcription

DMR5:151246001	5	1.51E+08	2000	2	4.35E-09	26	1.3	Ahdc1	
DMR5:151724101	5	1.52E+08	1700	1	8.00E-07	9	0.529411765		
DMR5:152430101	5	1.52E+08	1400	1	3.34E-09	17	1.214285714	Umodl;Catsper4	Transport
DMR5:152434601	5	1.52E+08	2300	2	2.90E-07	18	0.782608696	Catsper4;Cnksr1	Transport;Signaling
DMR5:152613101	5	1.53E+08	900	1	6.72E-07	17	1.888888889	Pafah2	Metabolism
DMR5:152642101	5	1.53E+08	4100	2	5.80E-09	70	1.707317073	Pafah2	Metabolism
DMR5:153310801	5	1.53E+08	1400	2	2.03E-10	17	1.214285714		
DMR5:153949701	5	1.54E+08	800	1	4.39E-07	5	0.625		
DMR5:154561501	5	1.55E+08	1700	1	5.69E-08	25	1.470588235	Asap3	Transcription
DMR5:154628601	5	1.55E+08	300	1	5.86E-09	6	2	Tcea3	Transcription
DMR5:154759001	5	1.55E+08	1600	2	2.27E-08	18	1.125		
DMR5:155072301	5	1.55E+08	4600	1	4.27E-07	94	2.043478261	Ephb2	Receptor
DMR5:155184601	5	1.55E+08	700	1	3.80E-07	6	0.857142857	Ephb2	Receptor
DMR5:156076101	5	1.56E+08	500	1	1.93E-08	0	0	Rap1gap;Alpl	Signaling;Metabolism
DMR5:156779401	5	1.57E+08	1100	1	4.24E-07	12	1.090909091	Cda	Metabolism
DMR5:156825501	5	1.57E+08	2700	1	5.10E-08	40	1.481481481		
DMR5:157173701	5	1.57E+08	1300	1	3.27E-07	18	1.384615385	Ubxn10;Pla2g2c	Metabolism
DMR5:157208101	5	1.57E+08	1900	1	2.04E-07	36	1.894736842	Pla2g2f	Metabolism
DMR5:157236801	5	1.57E+08	2700	1	9.52E-07	42	1.555555556	Pla2g2d;Pla2g5	Metabolism
DMR5:157997201	5	1.58E+08	2500	1	2.42E-08	35	1.4		
DMR5:158186501	5	1.58E+08	1600	1	6.96E-08	13	0.8125		
DMR5:158315701	5	1.58E+08	2800	2	3.21E-12	46	1.642857143	Pax7	Transcription
DMR5:158730901	5	1.59E+08	500	1	5.32E-07	2	0.4	AABR07050265.1	
DMR5:159329301	5	1.59E+08	300	1	5.48E-07	2	0.666666667	Padi3;Padi1	Metabolism
DMR5:160166901	5	1.6E+08	800	1	1.08E-07	13	1.625	Fblim1;Tmem82;Slc25a34	Cytoskeleton;Unknown;Binding Protein

DMR5:160945901	5	1.61E+08	600	3	8.00E-10	0	0		
DMR5:161327801	5	1.61E+08	400	2	7.17E-10	1	0.25		
DMR5:161775901	5	1.62E+08	1600	2	7.48E-08	22	1.375	Prdm2	Transcription
DMR5:161942101	5	1.62E+08	700	1	4.73E-07	17	2.428571429	Pdpn	Development
DMR5:162155101	5	1.62E+08	1800	4	4.03E-09	12	0.666666667	Pramef27;Oog1	
DMR5:162564101	5	1.63E+08	4800	1	2.07E-07	58	1.208333333		
DMR5:162680801	5	1.63E+08	1500	2	6.30E-09	8	0.533333333	Aadacl4	Metabolism
DMR5:162852801	5	1.63E+08	900	1	1.27E-09	9	1	Dhrs3	Metabolism
DMR5:163200501	5	1.63E+08	1300	3	3.90E-10	14	1.076923077	Tnfrsf8	Receptor
DMR5:164205601	5	1.64E+08	700	1	6.07E-07	10	1.428571429	U6	
DMR5:164599701	5	1.65E+08	2000	1	5.04E-07	61	3.05		
DMR5:164933701	5	1.65E+08	700	1	9.89E-10	11	1.571428571	Draxin	
DMR5:164988601	5	1.65E+08	5100	5	3.48E-12	77	1.509803922		
DMR5:165526301	5	1.66E+08	800	5	4.52E-10	2	0.25		
DMR5:165582201	5	1.66E+08	2500	6	1.69E-13	34	1.36		
DMR5:165690501	5	1.66E+08	1200	1	2.11E-07	28	2.333333333		
DMR5:166322401	5	1.66E+08	1600	1	6.50E-09	12	0.75	LOC691196	
DMR5:166660801	5	1.67E+08	1200	1	8.46E-07	6	0.5		
DMR5:166917001	5	1.67E+08	600	1	2.19E-09	5	0.833333333	Spsb1	Proteolysis
DMR5:167423901	5	1.67E+08	300	1	3.24E-08	2	0.666666667	Rere	Unknown
DMR5:167820801	5	1.68E+08	500	1	3.80E-09	8	1.6		
DMR5:167830301	5	1.68E+08	2100	1	7.34E-10	35	1.666666667		
DMR5:168303001	5	1.68E+08	1100	1	1.81E-07	13	1.181818182	Camta1	Transcription
DMR5:169032501	5	1.69E+08	400	2	1.70E-09	3	0.75	Rn60_5_1691.2	
DMR5:169040601	5	1.69E+08	900	1	3.91E-07	19	2.111111111	Rn60_5_1691.2	
DMR5:169598901	5	1.7E+08	800	1	4.63E-07	16	2	Kcnab2	Transport
DMR5:169616901	5	1.7E+08	600	1	9.89E-09	13	2.166666667	Kcnab2	Transport
DMR5:169788001	5	1.7E+08	1000	1	4.17E-07	6	0.6		
DMR5:169815301	5	1.7E+08	3900	1	6.44E-08	66	1.692307692		

DMR5:169882501	5	1.7E+08	2200	6	3.67E-14	15	0.681818182		
DMR5:169983901	5	1.7E+08	2700	1	7.91E-07	23	0.851851852		
DMR5:170233001	5	1.7E+08	500	1	2.07E-07	4	0.8		
DMR5:170514801	5	1.71E+08	600	3	1.62E-11	2	0.333333333		
DMR5:170545801	5	1.71E+08	2100	1	3.21E-07	25	1.19047619		
DMR5:170745001	5	1.71E+08	300	1	2.01E-07	4	1.333333333		
DMR5:170775101	5	1.71E+08	2400	1	6.35E-07	42	1.75	AABR07050600.1	
DMR5:171388201	5	1.71E+08	2300	1	1.57E-07	36	1.565217391	Tp73	Transcription
DMR5:171429701	5	1.71E+08	2200	1	7.75E-08	28	1.272727273	Wrap73	
DMR5:171757701	5	1.72E+08	2900	1	3.31E-08	26	0.896551724		
DMR5:171889201	5	1.72E+08	3400	2	3.00E-08	48	1.411764706		
DMR5:172668501	5	1.73E+08	4300	2	3.84E-09	64	1.488372093	Prkcz	Signaling
DMR5:172712001	5	1.73E+08	2700	1	7.24E-07	43	1.592592593	Prkcz	Signaling
DMR5:172882801	5	1.73E+08	1800	1	8.88E-07	26	1.444444444	AC130035.1;Tmem52;AC130035.2	
DMR5:172985801	5	1.73E+08	500	1	5.84E-07	15	3	Gnb1;Nadk	Signaling
DMR5:173200901	5	1.73E+08	800	1	3.63E-07	6	0.75	Atad3a	Metabolism
DMR5:173233801	5	1.73E+08	200	1	7.72E-07	0	0	Tmem88b;Ankrd65	Unknown
DMR5:173245601	5	1.73E+08	300	1	3.60E-07	2	0.666666667	Ankrd65;Mrpl20	Translation
DMR5:173637201	5	1.74E+08	1600	1	5.49E-09	13	0.8125	Perm1;Plekhn1	
DMR6:75101	6	75101	1000	1	8.29E-08	6	0.6	AABR07062615.1	
DMR6:153701	6	153701	1900	2	9.36E-11	18	0.947368421		
DMR6:292701	6	292701	1600	2	3.33E-09	12	0.75		
DMR6:1152501	6	1152501	2800	1	1.18E-09	52	1.857142857	Vit	Extracellular Matrix
DMR6:1321801	6	1321801	1000	1	1.44E-07	10	1	Strn;Heatr5b	Receptor
DMR6:1385501	6	1385501	2300	1	3.92E-08	27	1.173913043	Heatr5b	Receptor
DMR6:2028101	6	2028101	400	2	8.18E-11	1	0.25		
DMR6:2642401	6	2642401	1500	1	3.75E-07	7	0.466666667		

DMR6:2701801	6	2701801	300	1	8.59E-07	5	1.666666667	Hnrnpll;U6	
DMR6:2961301	6	2961301	1700	2	4.11E-09	19	1.117647059	Dhx57;Morn2	Transcription
DMR6:3835301	6	3835301	500	1	3.04E-07	3	0.6		
DMR6:4800301	6	4800301	100	1	2.79E-07	1	1		
DMR6:5122501	6	5122501	1300	3	4.57E-12	3	0.230769231		
DMR6:6710301	6	6710301	400	1	6.81E-08	2	0.5	Cox7a2l	Electron Transport
DMR6:6712601	6	6712601	500	3	4.14E-09	1	0.2	Cox7a2l	Electron Transport
DMR6:7083101	6	7083101	500	1	5.94E-09	3	0.6		
DMR6:8536801	6	8536801	2800	1	8.38E-07	49	1.75		
DMR6:8787501	6	8787501	4500	2	1.01E-07	64	1.422222222		
DMR6:9620501	6	9620501	1700	9	1.40E-09	7	0.411764706	AABR07062800.1	
DMR6:9956801	6	9956801	400	2	1.37E-10	2	0.5	Prkce	Binding Protein
DMR6:10805901	6	10805901	8800	1	7.47E-07	379	4.306818182		
DMR6:12857501	6	12857501	200	1	7.24E-07	0	0	Fshr	Receptor
DMR6:13828101	6	13828101	400	2	6.41E-09	1	0.25	Rn60_6_0139.1	
DMR6:13857101	6	13857101	2200	1	3.74E-07	15	0.681818182		
DMR6:15136001	6	15136001	1300	1	1.45E-07	4	0.307692308	Nrxn1	Receptor
DMR6:15704201	6	15704201	300	1	1.31E-07	2	0.666666667		
DMR6:16541301	6	16541301	1300	1	3.24E-08	11	0.846153846		
DMR6:17306901	6	17306901	1000	1	4.96E-09	8	0.8		
DMR6:20428301	6	20428301	800	2	3.18E-09	6	0.75		
DMR6:21666801	6	21666801	4400	1	7.52E-07	24	0.545454545		
DMR6:21673601	6	21673601	1500	5	1.63E-08	20	1.333333333		
DMR6:21677601	6	21677601	300	2	2.50E-07	4	1.333333333		
DMR6:21697201	6	21697201	2000	8	2.79E-17	28	1.4	SNORA48;AABR07063197.1	

DMR6:21707601	6	21707601	400	1	1.17E-08	1	0.25	SNORA48;AABR07063197.1	
DMR6:21941801	6	21941801	1300	2	1.06E-31	9	0.692307692	Birc6	Unknown
DMR6:22310901	6	22310901	3600	2	2.18E-10	36	1	Dpy30	
DMR6:22545001	6	22545001	400	1	5.90E-07	0	0		
DMR6:23171401	6	23171401	1100	2	1.82E-08	11	1	Alk	Receptor
DMR6:24102701	6	24102701	800	2	7.10E-09	7	0.875		
DMR6:25233501	6	25233501	600	2	3.01E-07	4	0.666666667		
DMR6:25337501	6	25337501	300	1	1.81E-07	1	0.333333333		
DMR6:25517101	6	25517101	1000	3	4.46E-09	5	0.5		
DMR6:26342101	6	26342101	400	2	8.18E-11	0	0		
DMR6:26769301	6	26769301	3700	1	3.44E-07	67	1.810810811	Tcf23;Prr30	
DMR6:27290601	6	27290601	700	1	2.34E-07	4	0.571428571	Cib4	Signaling
DMR6:27343101	6	27343101	1500	2	3.71E-11	22	1.466666667	Otof	Transport
DMR6:29361501	6	29361501	2700	1	2.58E-07	46	1.703703704	Klhl29	Transcription
DMR6:29504501	6	29504501	900	1	1.68E-07	5	0.555555556		
DMR6:29951901	6	29951901	1700	1	3.81E-08	20	1.176470588	Wdcp	
DMR6:30260301	6	30260301	3200	1	5.34E-07	27	0.84375		
DMR6:30593501	6	30593501	6200	1	4.53E-08	44	0.709677419		
DMR6:30629501	6	30629501	3400	1	5.17E-08	480	14.11764706	5_8S_rRNA;AABR07063421.1;AABR07063424.1;LOC257642;AABR07063425.2;AABR07063425.1	
DMR6:32377801	6	32377801	700	1	5.84E-08	3	0.428571429	AABR07063508.1	
DMR6:32572801	6	32572801	1400	2	2.50E-09	2	0.142857143	AABR07063511.2	
DMR6:32914701	6	32914701	400	1	9.56E-08	0	0		
DMR6:33662901	6	33662901	400	1	1.24E-07	1	0.25		
DMR6:33965301	6	33965301	400	1	2.77E-08	2	0.5		

DMR6:34163801	6	34163801	400	2	6.46E-09	3	0.75	Ttc32	
DMR6:34342001	6	34342001	2700	1	6.03E-07	19	0.703703704	7SK	
DMR6:35287001	6	35287001	400	1	5.94E-07	0	0	AABR07063558.1	
DMR6:35445901	6	35445901	1500	1	4.72E-08	13	0.866666667	AABR07063565.1	
DMR6:35740101	6	35740101	700	1	9.44E-07	0	0		
DMR6:35746101	6	35746101	300	1	3.63E-07	1	0.333333333		
DMR6:36073101	6	36073101	500	1	2.13E-07	0	0	AABR07063581.1	
DMR6:36154901	6	36154901	1100	2	1.21E-13	7	0.636363636		
DMR6:36588001	6	36588001	600	1	7.12E-07	2	0.333333333		
DMR6:38470201	6	38470201	1000	1	8.19E-08	1	0.1	Nbas	Unknown
DMR6:38572801	6	38572801	1700	1	7.17E-07	25	1.470588235	Nbas;AABR07063638.1	Unknown
DMR6:40009601	6	40009601	2300	1	5.74E-08	23	1		
DMR6:40934201	6	40934201	1500	1	1.56E-09	14	0.933333333		
DMR6:41054701	6	41054701	700	3	4.06E-10	5	0.714285714		
DMR6:41560401	6	41560401	700	2	4.29E-14	7	1		
DMR6:42087201	6	42087201	1500	1	1.19E-09	2	0.133333333	E2f6	Transcription
DMR6:42481301	6	42481301	400	1	9.95E-08	0	0	Kcnf1	Metabolism
DMR6:42682301	6	42682301	1200	1	2.30E-08	12	1	Nol10	Transcription
DMR6:42763901	6	42763901	600	1	6.27E-08	11	1.833333333		
DMR6:43861401	6	43861401	900	1	1.11E-08	37	4.111111111	Cys1	
DMR6:44549701	6	44549701	1300	1	6.86E-07	10	0.769230769		
DMR6:45814601	6	45814601	300	1	2.41E-07	1	0.333333333		
DMR6:48075301	6	48075301	600	1	4.36E-07	7	1.166666667	Tssc1	
DMR6:48416101	6	48416101	1300	1	1.02E-09	14	1.076923077		
DMR6:48471501	6	48471501	300	1	2.13E-07	3	1	Myt1l	Transcription
DMR6:48568101	6	48568101	5100	1	2.95E-08	55	1.078431373	Myt1l	Transcription
DMR6:49144701	6	49144701	400	1	2.43E-11	0	0	Sntg2	Cytoskeleton
DMR6:49556501	6	49556501	2300	3	6.50E-10	9	0.391304348		

DMR6:50377701	6	50377701	400	2	1.29E-07	4	1		
DMR6:50438701	6	50438701	200	1	8.81E-07	3	1.5		
DMR6:50640701	6	50640701	5200	1	1.86E-08	61	1.173076923		
DMR6:50755801	6	50755801	400	1	3.08E-10	0	0	Slc26a3	Transport
DMR6:52017701	6	52017701	400	1	8.42E-07	6	1.5		
DMR6:55104901	6	55104901	500	2	7.02E-11	0	0		
DMR6:55593201	6	55593201	2000	1	8.29E-08	7	0.35	Bzw2	Transcription
DMR6:55776901	6	55776901	2200	2	3.29E-08	21	0.954545455		
DMR6:56565701	6	56565701	2100	2	4.49E-08	7	0.333333333		
DMR6:57050501	6	57050501	400	2	7.75E-09	0	0	Agmo	Metabolism
DMR6:57064301	6	57064301	800	3	9.89E-08	0	0	Agmo	Metabolism
DMR6:57319701	6	57319701	300	1	3.10E-07	0	0		
DMR6:59082001	6	59082001	900	2	1.99E-08	2	0.222222222		
DMR6:60245801	6	60245801	1500	1	5.65E-08	14	0.933333333	Zfp277	Transcription
DMR6:61993701	6	61993701	3300	1	8.19E-07	35	1.060606061		
DMR6:62044901	6	62044901	500	3	5.13E-10	0	0		
DMR6:63663901	6	63663901	500	1	3.02E-07	0	0		
DMR6:64005101	6	64005101	1700	1	3.94E-14	6	0.352941176		
DMR6:68039201	6	68039201	400	3	6.09E-11	0	0		
DMR6:68385501	6	68385501	400	1	7.15E-07	0	0		
DMR6:70481401	6	70481401	300	1	2.22E-08	0	0		
DMR6:72630801	6	72630801	2400	1	7.98E-08	27	1.125		
DMR6:72943001	6	72943001	200	1	4.09E-07	0	0	Nubpl	Metabolism
DMR6:72981501	6	72981501	1000	1	3.25E-08	28	2.8	Nubpl	Metabolism
DMR6:73635201	6	73635201	1500	3	2.69E-13	12	0.8	Akap6	Signaling
DMR6:74051801	6	74051801	1800	2	9.86E-10	29	1.611111111		
DMR6:75010701	6	75010701	6300	1	7.95E-07	73	1.158730159		
DMR6:75450101	6	75450101	300	1	1.98E-07	2	0.666666667		
DMR6:77149001	6	77149001	2000	1	6.49E-07	18	0.9	7SK	

DMR6:77548201	6	77548201	1000	2	3.36E-07	24	2.4		
DMR6:78706201	6	78706201	1600	2	2.08E-08	18	1.125		
DMR6:78733401	6	78733401	1300	2	3.39E-08	8	0.615384615		
DMR6:80663401	6	80663401	400	2	2.19E-07	0	0		
DMR6:83586001	6	83586001	600	1	1.24E-07	2	0.333333333		
DMR6:84214701	6	84214701	1300	1	5.05E-08	7	0.538461538		
DMR6:85120401	6	85120401	500	2	7.55E-12	1	0.2		
DMR6:86293901	6	86293901	4200	1	8.78E-07	28	0.666666667		
DMR6:86362601	6	86362601	6600	1	6.25E-08	91	1.378787879		
DMR6:87969201	6	87969201	600	1	8.29E-08	3	0.5		
DMR6:88315801	6	88315801	300	2	4.57E-09	0	0		
DMR6:89303101	6	89303101	500	1	2.72E-08	2	0.4		
DMR6:90221301	6	90221301	2500	4	9.33E-11	10	0.4		
DMR6:91187401	6	91187401	500	3	5.66E-12	1	0.2		
DMR6:91587901	6	91587901	6000	2	5.43E-08	77	1.283333333	Klhdc1;AABR07064716.1;Klhdc2	
DMR6:92061101	6	92061101	1700	1	6.84E-07	5	0.294117647	L2hgdh;Atp5s	Metabolism;Electron Transport
DMR6:92669501	6	92669501	1300	1	1.16E-07	29	2.230769231	Trim9	Unknown
DMR6:93400401	6	93400401	1700	1	2.39E-08	27	1.588235294	Actr10	Cytoskeleton
DMR6:93575701	6	93575701	1700	1	1.99E-07	13	0.764705882	Tomm20l;LOC690035	Transport
DMR6:94068401	6	94068401	2100	1	1.46E-07	35	1.666666667		
DMR6:96659601	6	96659601	2900	3	5.87E-07	27	0.931034483	Prkch	Binding Protein
DMR6:97343601	6	97343601	900	1	1.45E-07	4	0.444444444	AABR07064835.1	
DMR6:97800201	6	97800201	1900	1	1.65E-08	7	0.368421053		
DMR6:98231901	6	98231901	300	1	5.54E-10	0	0		
DMR6:98282701	6	98282701	300	1	3.82E-07	1	0.333333333	Rhoj	Signaling

DMR6:98918601	6	98918601	2200	1	3.67E-08	28	1.272727273	Syne2	
DMR6:98960101	6	98960101	2800	1	9.10E-09	21	0.75	Syne2	
DMR6:99445301	6	99445301	2400	1	3.28E-10	24	1	Hspa2;Ppp1r36	Protein Binding
DMR6:99646201	6	99646201	1900	1	9.03E-07	27	1.421052632	Plekhg3	Signaling
DMR6:99812301	6	99812301	300	1	9.49E-07	5	1.666666667	Churc1;U6	
DMR6:99976601	6	99976601	700	3	8.43E-12	0	0	Fntb;Max	Metabolism;Transcription
DMR6:100121001	6	1E+08	2000	3	1.43E-07	9	0.45		
DMR6:100322301	6	1E+08	700	2	9.39E-13	5	0.714285714		
DMR6:100963101	6	1.01E+08	1000	2	2.43E-08	6	0.6		
DMR6:101729401	6	1.02E+08	500	1	7.71E-08	2	0.4	Gphn	Receptor
DMR6:102104301	6	1.02E+08	600	1	3.93E-07	6	1	Plek2	Cytoskeleton
DMR6:102896001	6	1.03E+08	2800	1	8.55E-09	27	0.964285714		
DMR6:103475401	6	1.03E+08	500	2	1.34E-07	0	0	Actn1	Cytoskeleton
DMR6:104107701	6	1.04E+08	400	1	4.85E-07	3	0.75	Galnt16	Metabolism
DMR6:104805901	6	1.05E+08	1500	1	8.00E-10	7	0.466666667	Smoc1	Receptor
DMR6:104967101	6	1.05E+08	300	1	2.89E-08	3	1	Slc8a3	Transport
DMR6:107587901	6	1.08E+08	800	1	6.23E-07	1	0.125	Rn50_6_1181.1;Acot6;Dnal1	Cytoskeleton
DMR6:107980601	6	1.08E+08	900	1	4.07E-09	2	0.222222222		
DMR6:108542001	6	1.09E+08	1000	1	1.73E-08	8	0.8	Ltbp2	Receptor
DMR6:108585601	6	1.09E+08	2500	1	2.47E-07	24	0.96	Ltbp2	Receptor
DMR6:109542901	6	1.1E+08	300	2	1.23E-11	0	0		
DMR6:110264301	6	1.1E+08	1000	5	3.05E-11	10	1	AABR07065113.2	
DMR6:110279401	6	1.1E+08	1600	1	6.54E-07	20	1.25		
DMR6:110814101	6	1.11E+08	1100	1	6.95E-07	18	1.636363636	7SK	
DMR6:110934901	6	1.11E+08	1000	1	8.08E-08	8	0.8		
DMR6:111065901	6	1.11E+08	1600	1	1.73E-07	18	1.125	Tmem63c	
DMR6:112181701	6	1.12E+08	500	1	9.31E-07	3	0.6	Nrxn3	Receptor

DMR6:112516901	6	1.13E+08	300	2	2.59E-10	0	0	Nrxn3	Receptor
DMR6:112664501	6	1.13E+08	400	2	2.15E-07	0	0	Nrxn3	Receptor
DMR6:113243801	6	1.13E+08	300	1	7.88E-07	0	0	Nrxn3	Receptor
DMR6:113377801	6	1.13E+08	900	1	2.79E-07	4	0.444444444	Nrxn3	Receptor
DMR6:113481701	6	1.13E+08	200	1	4.43E-07	6	3	Nrxn3	Receptor
DMR6:113767201	6	1.14E+08	300	1	1.99E-07	6	2	Nrxn3	Receptor
DMR6:114466901	6	1.14E+08	300	1	5.14E-07	0	0	Dio2	Metabolism
DMR6:114500501	6	1.15E+08	1000	1	3.35E-08	5	0.5		
DMR6:115384701	6	1.15E+08	3500	1	2.43E-07	45	1.285714286		
DMR6:117182401	6	1.17E+08	1400	2	2.23E-09	6	0.428571429		
DMR6:120158101	6	1.2E+08	700	2	3.26E-08	4	0.571428571		
DMR6:120696601	6	1.21E+08	1800	1	8.85E-08	18	1		
DMR6:122913701	6	1.23E+08	300	1	3.67E-07	0	0	Ttc8	Metabolism
DMR6:122916701	6	1.23E+08	800	1	8.63E-08	7	0.875	Ttc8	Metabolism
DMR6:122994901	6	1.23E+08	700	1	4.12E-07	3	0.428571429		
DMR6:124472501	6	1.24E+08	1600	1	3.11E-09	2	0.125		
DMR6:125533501	6	1.26E+08	2500	1	4.76E-07	10	0.4	Tc2n	Unknown
DMR6:125916501	6	1.26E+08	4900	2	5.55E-09	83	1.693877551	AABR07065406.2	
DMR6:126376001	6	1.26E+08	400	1	4.51E-07	3	0.75	Golga5	Golgi
DMR6:127218901	6	1.27E+08	5200	1	3.61E-07	82	1.576923077	Asb2	Transcription
DMR6:128002201	6	1.28E+08	500	2	9.19E-08	1	0.2	LOC500712;Serpina3c	Proteolysis
DMR6:128326901	6	1.28E+08	2200	3	5.58E-11	18	0.818181818		
DMR6:129131401	6	1.29E+08	1000	1	1.82E-07	12	1.2		
DMR6:129779501	6	1.3E+08	3600	1	5.48E-07	63	1.75		
DMR6:130037701	6	1.3E+08	1700	2	3.31E-08	19	1.117647059	SNORA17	
DMR6:130075501	6	1.3E+08	400	1	1.09E-07	0	0		
DMR6:130704501	6	1.31E+08	1500	1	1.13E-07	19	1.266666667		
DMR6:130774601	6	1.31E+08	700	2	5.47E-08	4	0.571428571		

DMR6:131006501	6	1.31E+08	400	2	7.18E-11	2	0.5		
DMR6:131160801	6	1.31E+08	1300	2	7.00E-10	14	1.076923077		
DMR6:131522801	6	1.32E+08	2200	1	2.34E-07	30	1.363636364		
DMR6:131580901	6	1.32E+08	400	1	5.05E-08	1	0.25		
DMR6:131771301	6	1.32E+08	600	2	3.97E-09	4	0.666666667		
DMR6:132008401	6	1.32E+08	1300	5	2.01E-12	6	0.461538462		
DMR6:132222101	6	1.32E+08	400	1	3.34E-07	10	2.5	Hhipl1	Receptor
DMR6:132395201	6	1.32E+08	2300	2	7.82E-07	16	0.695652174	Eml1	
DMR6:132909401	6	1.33E+08	1600	1	5.77E-08	22	1.375	Wdr25	
DMR6:133596801	6	1.34E+08	3900	1	1.18E-07	38	0.974358974		
DMR6:133612001	6	1.34E+08	2100	3	2.68E-09	29	1.380952381		
DMR6:134241301	6	1.34E+08	3200	1	9.30E-09	52	1.625		
DMR6:134503301	6	1.35E+08	1900	1	1.71E-07	48	2.526315789		
DMR6:134654901	6	1.35E+08	700	1	1.77E-07	1	0.142857143		
DMR6:135019201	6	1.35E+08	3000	2	4.25E-08	77	2.566666667	Dync1h1;RGD1305298	Cytoskeleton
DMR6:135082301	6	1.35E+08	2900	2	1.51E-07	77	2.655172414	Dync1h1;RGD1305298	Cytoskeleton
DMR6:135139101	6	1.35E+08	1700	3	1.43E-07	18	1.058823529	Rpl35al1	
DMR6:135261001	6	1.35E+08	3200	1	1.03E-08	57	1.78125	Mok;AC121220.1;Zfp839	Signaling
DMR6:136406501	6	1.36E+08	1500	1	9.19E-08	15	1	Zfyve21;Ppp1r13b	Transport;Signaling
DMR6:136580001	6	1.37E+08	1700	1	1.23E-07	17	1	Tdrd9	Transcription
DMR6:137061501	6	1.37E+08	2000	1	4.75E-07	24	1.2	LOC691485	Unknown
DMR6:137124801	6	1.37E+08	800	1	1.13E-08	8	1	AABR07065602.2	
DMR6:137542901	6	1.38E+08	1300	1	2.27E-08	19	1.461538462		
DMR6:137561801	6	1.38E+08	500	3	4.81E-13	0	0		

DMR6:137955701	6	1.38E+08	1000	1	3.32E-07	17	1.7	Mta1;Crip2;Crip1	Transcription;Unknown;Cytoskeleton
DMR6:138040501	6	1.38E+08	1700	1	5.79E-08	9	0.529411765		
DMR6:139985201	6	1.4E+08	100	1	4.04E-11	0	0		
DMR6:141673201	6	1.42E+08	800	1	5.22E-07	0	0		
DMR6:141694401	6	1.42E+08	500	2	6.94E-12	0	0		
DMR6:143379701	6	1.43E+08	800	1	2.62E-07	22	2.75		
DMR6:143571601	6	1.44E+08	1200	1	1.76E-07	13	1.083333333		
DMR6:143737701	6	1.44E+08	700	2	1.93E-07	4	0.571428571		
DMR6:144541301	6	1.45E+08	2900	1	3.56E-07	33	1.137931034	Ptprn2;7SK	Signaling
DMR6:145444801	6	1.45E+08	1000	1	5.62E-07	5	0.5		
DMR6:145762401	6	1.46E+08	2200	1	2.60E-07	33	1.5	Cdca7l	
DMR6:146031101	6	1.46E+08	2500	1	1.34E-10	39	1.56	Dnah11	Cytoskeleton
DMR6:146859801	6	1.47E+08	500	2	7.00E-09	34	6.8	Abcb5	Transcription
DMR7:56501	7	56501	1100	1	9.39E-07	11	1		
DMR7:500401	7	500401	2500	1	4.79E-08	7	0.28		
DMR7:791901	7	791901	300	2	4.67E-09	2	0.666666667		
DMR7:1018901	7	1018901	800	1	7.29E-07	13	1.625		
DMR7:2625401	7	2625401	700	1	1.53E-07	11	1.571428571	Gls2;Spryd4;Mip	Metabolism;Unknown;Transport
DMR7:2788401	7	2788401	1400	1	2.27E-08	15	1.071428571	Cs;Coq10a;Ankrd52	Metabolism;Transcription
DMR7:5641701	7	5641701	2700	1	1.00E-06	10	0.37037037		
DMR7:5860801	7	5860801	900	2	3.55E-13	10	1.111111111		
DMR7:8168501	7	8168501	600	1	7.43E-08	3	0.5		
DMR7:9753801	7	9753801	2200	1	1.32E-07	13	0.590909091	LOC102549817	
DMR7:9811001	7	9811001	3000	1	1.08E-08	59	1.966666667	LOC102549817	

DMR7:10228701	7	10228701	3800	1	1.92E-07	33	0.868421053		
DMR7:10257001	7	10257001	700	1	1.63E-08	1	0.142857143	AABR07055812.1	
DMR7:10270501	7	10270501	6600	6	2.52E-10	47	0.712121212	AABR07055812.1	
DMR7:11307401	7	11307401	900	1	9.41E-07	18	2	Tjp3;Apba3;AC094643.1	Cell Junction;Receptor
DMR7:12209401	7	12209401	800	5	4.48E-09	0	0	Plk5	Unknown
DMR7:12647501	7	12647501	700	1	3.75E-08	10	1.428571429	Arhgap45;Elane;Prtn3;Plppr3	Protease
DMR7:13058601	7	13058601	1900	1	5.39E-08	41	2.157894737	Mier2;AC115214.1;Plpp2	Development
DMR7:13656601	7	13656601	1800	1	4.37E-07	20	1.111111111		
DMR7:13769401	7	13769401	2000	3	1.24E-09	11	0.55		
DMR7:15796301	7	15796301	4000	1	2.95E-07	35	0.875	Zfp955a	
DMR7:17988601	7	17988601	2200	1	5.60E-07	7	0.318181818		
DMR7:18346401	7	18346401	1800	2	5.81E-08	12	0.666666667	AABR07056014.1	
DMR7:18476501	7	18476501	700	1	9.46E-08	6	0.857142857	Myo1f	Cytoskeleton
DMR7:18943901	7	18943901	600	1	9.31E-08	1	0.166666667		
DMR7:19368901	7	19368901	300	1	2.33E-08	2	0.666666667		
DMR7:20242201	7	20242201	2600	1	2.29E-07	15	0.576923077		
DMR7:21334101	7	21334101	400	1	8.03E-08	1	0.25		
DMR7:21496501	7	21496501	300	1	9.94E-07	7	2.333333333		
DMR7:21709901	7	21709901	1300	3	5.24E-12	4	0.307692308		
DMR7:21714001	7	21714001	7800	1	1.57E-08	65	0.833333333		
DMR7:22452201	7	22452201	3300	1	2.72E-07	136	4.121212121	5_8S_rRNA	
DMR7:23005301	7	23005301	1200	1	2.17E-07	5	0.416666667	LOC300308	
DMR7:23653301	7	23653301	900	1	9.01E-07	11	1.222222222	Syn3	Development
DMR7:23727101	7	23727101	3500	2	2.61E-11	23	0.657142857	Syn3	Development
DMR7:23777701	7	23777701	600	2	7.78E-08	4	0.666666667	Syn3	Development

DMR7:24253201	7	24253201	1200	2	1.72E-10	8	0.666666667	Btbd11	Signaling
DMR7:24629001	7	24629001	300	1	5.32E-07	5	1.666666667	Cry1;Mterf2	DNA Repair
DMR7:24641101	7	24641101	1600	1	1.26E-07	21	1.3125	Cry1;Mterf2;Tmem263	DNA Repair
DMR7:25353601	7	25353601	4700	1	3.93E-07	53	1.127659574		
DMR7:25429201	7	25429201	1800	1	9.21E-07	24	1.333333333		
DMR7:25660201	7	25660201	1300	1	5.72E-07	19	1.461538462		
DMR7:26167901	7	26167901	1400	1	4.93E-09	14	1		
DMR7:26224301	7	26224301	300	1	9.41E-09	0	0		
DMR7:26555601	7	26555601	800	3	2.04E-08	2	0.25	Slc41a2	Metabolism
DMR7:27006801	7	27006801	900	1	7.46E-07	7	0.777777778		
DMR7:27419701	7	27419701	200	1	1.18E-07	0	0	Stab2	Extracellular Matrix
DMR7:29732301	7	29732301	500	2	3.05E-08	1	0.2		
DMR7:29743601	7	29743601	1700	1	2.32E-07	14	0.823529412		
DMR7:30926801	7	30926801	500	1	1.53E-10	0	0	Anks1b	Receptor
DMR7:31044901	7	31044901	400	1	3.21E-07	1	0.25	Anks1b	Receptor
DMR7:31670501	7	31670501	300	1	8.49E-08	2	0.666666667	Anks1b	Receptor
DMR7:32002101	7	32002101	2500	1	9.39E-07	21	0.84	AABR07056605.1	
DMR7:32853301	7	32853301	100	1	9.43E-07	0	0		
DMR7:33022401	7	33022401	1900	1	1.24E-07	13	0.684210526		
DMR7:33643901	7	33643901	500	2	2.27E-08	4	0.8		
DMR7:34199801	7	34199801	1900	1	7.19E-07	18	0.947368421		
DMR7:34288701	7	34288701	300	1	7.35E-07	9	3	Lta4h	Metabolism
DMR7:34569901	7	34569901	4000	1	7.94E-07	32	0.8	Ntn4	Extracellular Matrix
DMR7:34996401	7	34996401	2900	1	4.47E-07	39	1.344827586	Fgd6	Signaling
DMR7:35170001	7	35170001	1000	1	2.34E-07	5	0.5	Ndufa12	Metabolism
DMR7:35388001	7	35388001	400	1	6.52E-08	1	0.25	Tmcc3	

DMR7:35708701	7	35708701	1600	2	1.37E-13	4	0.25	AABR07056680.1;AABR07056678.1	
DMR7:36912701	7	36912701	1600	1	9.58E-07	19	1.1875	AABR07056688.1	
DMR7:37519601	7	37519601	1200	1	4.59E-07	4	0.333333333		
DMR7:37561801	7	37561801	900	2	2.72E-08	2	0.222222222		
DMR7:37591401	7	37591401	500	1	1.32E-07	2	0.4		
DMR7:37931801	7	37931801	1300	3	3.46E-11	21	1.615384615		
DMR7:37949201	7	37949201	500	2	4.34E-08	17	3.4		
DMR7:38670701	7	38670701	300	2	1.96E-09	3	1	AABR07056730.1	
DMR7:39757901	7	39757901	300	1	1.46E-07	6	2		
DMR7:39945601	7	39945601	1100	1	8.27E-07	7	0.636363636		
DMR7:40490001	7	40490001	1500	2	1.04E-09	13	0.866666667		
DMR7:40751401	7	40751401	1200	5	1.26E-10	3	0.25		
DMR7:40947901	7	40947901	1100	2	6.77E-10	12	1.090909091		
DMR7:41241301	7	41241301	800	1	5.34E-07	4	0.5		
DMR7:41509601	7	41509601	3900	1	6.56E-07	34	0.871794872		
DMR7:41731101	7	41731101	1400	2	1.14E-07	4	0.285714286		
DMR7:41957701	7	41957701	900	1	1.96E-08	2	0.222222222		
DMR7:43276601	7	43276601	1800	2	3.37E-08	14	0.777777778		
DMR7:44485201	7	44485201	4300	1	1.38E-07	41	0.953488372		
DMR7:44804101	7	44804101	400	1	7.82E-07	2	0.5		
DMR7:45315901	7	45315901	700	1	1.23E-08	1	0.142857143		
DMR7:46106301	7	46106301	500	1	6.96E-07	3	0.6		
DMR7:46884301	7	46884301	1300	1	3.05E-07	11	0.846153846		
DMR7:46969601	7	46969601	1300	2	5.10E-10	7	0.538461538		
DMR7:49253101	7	49253101	600	2	6.36E-09	1	0.166666667	Acss3;SNORA17	Metabolism
DMR7:49557301	7	49557301	700	2	8.54E-09	1	0.142857143	Lin7a	Cell Junction
DMR7:50290501	7	50290501	2800	1	1.47E-07	38	1.357142857	Syt1	Transport
DMR7:51254401	7	51254401	300	1	1.09E-07	1	0.333333333		

DMR7:52131001	7	52131001	300	1	7.67E-07	0	0		
DMR7:52342101	7	52342101	700	1	7.80E-15	7	1	Nav3	Development
DMR7:52570801	7	52570801	600	1	3.67E-07	2	0.333333333		
DMR7:53542601	7	53542601	1400	1	1.20E-07	13	0.928571429		
DMR7:53858901	7	53858901	400	2	1.71E-07	2	0.5		
DMR7:54125701	7	54125701	200	1	3.53E-07	0	0		
DMR7:54627201	7	54627201	1500	3	2.23E-12	7	0.466666667		
DMR7:57208101	7	57208101	400	2	1.40E-08	1	0.25		
DMR7:58101401	7	58101401	700	1	5.25E-09	0	0	Tph2	Metabolism
DMR7:58744601	7	58744601	2100	1	1.64E-08	13	0.619047619		
DMR7:59708001	7	59708001	2600	5	1.73E-08	11	0.423076923		
DMR7:59879901	7	59879901	2500	2	1.69E-07	18	0.72	Myrfl	
DMR7:61886901	7	61886901	1700	1	3.95E-07	14	0.823529412		
DMR7:61975301	7	61975301	1900	2	2.41E-07	7	0.368421053		
DMR7:62039201	7	62039201	300	1	3.35E-07	1	0.333333333		
DMR7:63132901	7	63132901	2000	1	3.28E-09	28	1.4	Wif1	Signaling
DMR7:63288701	7	63288701	1900	1	2.04E-07	19	1		
DMR7:63438501	7	63438501	2100	1	3.26E-12	15	0.714285714		
DMR7:63961101	7	63961101	2700	2	2.33E-09	15	0.555555556	Srgap1	Signaling
DMR7:64005801	7	64005801	200	1	6.02E-08	0	0	Srgap1	Signaling
DMR7:65030901	7	65030901	3600	1	7.50E-07	54	1.5		
DMR7:65135101	7	65135101	700	1	6.75E-07	7	1		
DMR7:65528301	7	65528301	400	1	2.19E-08	1	0.25		
DMR7:65981701	7	65981701	800	1	5.81E-08	4	0.5		
DMR7:66063301	7	66063301	400	1	3.21E-07	1	0.25	Fam19a2	Growth Factors & Cytokines
DMR7:66367801	7	66367801	1100	1	1.75E-09	8	0.727272727		
DMR7:66636701	7	66636701	900	1	4.16E-07	5	0.555555556	Usp15	Proteolysis
DMR7:66714401	7	66714401	400	2	9.56E-09	0	0	Mon2	Proteolysis

DMR7:66752201	7	66752201	400	1	5.48E-07	0	0	Mon2	Proteolysis
DMR7:66770201	7	66770201	400	1	3.45E-07	4	1	Mon2	Proteolysis
DMR7:66872201	7	66872201	400	1	5.57E-07	1	0.25	Ppm1h	Signaling
DMR7:68611101	7	68611101	800	1	1.65E-07	8	1		
DMR7:68677001	7	68677001	400	1	1.90E-08	1	0.25		
DMR7:69383001	7	69383001	400	1	5.91E-07	0	0		
DMR7:69710001	7	69710001	700	1	9.01E-07	2	0.285714286		
DMR7:69855001	7	69855001	4600	1	6.65E-08	50	1.086956522		
DMR7:70311201	7	70311201	2400	1	5.42E-07	42	1.75	Avil;Tsfm;Mettl21b	Cytoskeleton;Transcription
DMR7:70382701	7	70382701	2000	1	3.91E-07	36	1.8	Agap2;Os9	Transcription
DMR7:70505001	7	70505001	1000	1	2.34E-07	2	0.2	Pip4k2c;Kif5a	Signaling;Cytoskeleton
DMR7:70866201	7	70866201	600	1	6.91E-08	0	0	Lrp1	Receptor
DMR7:71835401	7	71835401	1500	1	3.44E-07	14	0.933333333	Cpq	Metabolism
DMR7:71979601	7	71979601	1300	3	2.55E-09	2	0.153846154	Cpq	Metabolism
DMR7:72002801	7	72002801	1700	1	2.17E-08	7	0.411764706	Cpq	Metabolism
DMR7:72933301	7	72933301	1300	1	4.48E-07	10	0.769230769	Laptm4b	Proteolysis
DMR7:73133001	7	73133001	1300	1	3.07E-07	4	0.307692308	Matn2;9430069I07Rik	Cytoskeleton
DMR7:74506101	7	74506101	400	1	1.70E-08	1	0.25		
DMR7:75435101	7	75435101	4000	2	1.79E-09	42	1.05		
DMR7:76484901	7	76484901	2800	1	8.78E-07	33	1.178571429	Ncald	Signaling
DMR7:76563801	7	76563801	500	1	1.60E-08	2	0.4		
DMR7:76924001	7	76924001	2700	2	3.69E-09	18	0.666666667		
DMR7:77062301	7	77062301	300	1	7.80E-07	0	0	Odf1	Cytoskeleton
DMR7:77114401	7	77114401	2700	2	1.81E-07	22	0.814814815		
DMR7:77150801	7	77150801	700	1	1.45E-07	10	1.428571429	Klf10	Transcription
DMR7:77308801	7	77308801	400	1	7.56E-09	2	0.5		

DMR7:77321201	7	77321201	1400	2	6.98E-08	2	0.142857143		
DMR7:77944301	7	77944301	1100	1	4.52E-07	12	1.090909091		
DMR7:78268601	7	78268601	700	1	5.85E-07	11	1.571428571	Rims2	Signaling
DMR7:79128801	7	79128801	2700	1	1.22E-09	17	0.62962963	AABR07057586.1	
DMR7:79274401	7	79274401	1800	1	9.82E-07	22	1.222222222		
DMR7:82188501	7	82188501	3100	1	5.84E-07	39	1.258064516		
DMR7:83339601	7	83339601	500	2	2.84E-10	1	0.2	Nudcd1;Eny2	
DMR7:84650101	7	84650101	400	1	2.13E-08	0	0		
DMR7:85134801	7	85134801	2100	1	3.33E-07	46	2.19047619		
DMR7:86178601	7	86178601	600	1	4.61E-10	3	0.5		
DMR7:86491101	7	86491101	100	1	2.67E-07	0	0		
DMR7:87178101	7	87178101	400	2	1.01E-09	2	0.5	Csmd3	
DMR7:87935501	7	87935501	2600	1	2.31E-08	48	1.846153846	Csmd3	
DMR7:88925701	7	88925701	300	1	1.21E-07	1	0.333333333		
DMR7:89954001	7	89954001	3100	1	3.66E-08	7	0.225806452		
DMR7:91868701	7	91868701	1300	1	1.39E-07	6	0.461538462	Slc30a8	Transport
DMR7:92059201	7	92059201	1400	1	1.58E-08	4	0.285714286		
DMR7:92676401	7	92676401	2600	2	2.41E-09	22	0.846153846	Ext1	Metabolism
DMR7:93318001	7	93318001	3900	1	1.68E-07	46	1.179487179	Samd12	Unknown
DMR7:93981401	7	93981401	800	3	1.77E-07	2	0.25	Colec10	Metabolism
DMR7:94641401	7	94641401	500	1	1.47E-07	1	0.2		
DMR7:95312901	7	95312901	2200	2	9.28E-08	12	0.545454545	Mrpl13;Mtbp	Transcription
DMR7:95325901	7	95325901	4100	1	4.47E-07	43	1.048780488	Mtbp	
DMR7:95509801	7	95509801	300	1	5.74E-07	4	1.333333333	Sntb1	Development
DMR7:95856601	7	95856601	800	1	5.78E-08	2	0.25		
DMR7:96654401	7	96654401	500	2	9.18E-10	2	0.4		
DMR7:97359301	7	97359301	500	1	7.01E-07	1	0.2	AABR07057997.1	
DMR7:98586601	7	98586601	900	1	2.90E-07	6	0.666666667		
DMR7:99226401	7	99226401	300	1	3.62E-07	4	1.333333333		

DMR7:99422701	7	99422701	800	1	6.65E-07	2	0.25		
DMR7:99521801	7	99521801	4400	1	4.99E-07	332	7.545454545		
DMR7:99983501	7	99983501	400	1	2.35E-07	5	1.25		
DMR7:100014001	7	1E+08	2900	3	4.62E-10	29	1		
DMR7:100512501	7	1.01E+08	3100	1	7.98E-11	9	0.290322581		
DMR7:100573001	7	1.01E+08	200	1	2.80E-08	1	0.5		
DMR7:101172101	7	1.01E+08	400	1	3.74E-07	0	0		
DMR7:101759101	7	1.02E+08	600	1	4.24E-07	3	0.5		
DMR7:101934801	7	1.02E+08	800	1	4.25E-10	7	0.875	Rn60_7_1020.1	
DMR7:101967301	7	1.02E+08	4300	1	2.82E-07	30	0.697674419		
DMR7:102904401	7	1.03E+08	400	1	6.48E-08	1	0.25		
DMR7:103114201	7	1.03E+08	700	1	2.98E-07	8	1.142857143		
DMR7:107739701	7	1.08E+08	300	1	1.61E-07	1	0.333333333	Ndrg1	Transcription
DMR7:108555801	7	1.09E+08	2200	1	3.81E-10	11	0.5	Rn60_7_1086.1	
DMR7:108663801	7	1.09E+08	1200	2	6.85E-23	11	0.916666667	Phf20l1	Epigenetic
DMR7:109218701	7	1.09E+08	3400	1	6.83E-07	32	0.941176471		
DMR7:109721701	7	1.1E+08	300	1	7.81E-07	0	0		
DMR7:110226601	7	1.1E+08	2200	1	4.17E-07	7	0.318181818		
DMR7:110257901	7	1.1E+08	400	3	4.14E-13	0	0		
DMR7:111437001	7	1.11E+08	800	2	3.71E-08	2	0.25		
DMR7:111670501	7	1.12E+08	500	1	8.54E-07	1	0.2	AABR07058360.2	
DMR7:112655801	7	1.13E+08	500	3	1.47E-09	0	0		
DMR7:113004101	7	1.13E+08	700	1	3.25E-09	1	0.142857143		
DMR7:113541601	7	1.14E+08	600	1	3.35E-07	5	0.833333333		
DMR7:113943501	7	1.14E+08	400	1	4.59E-08	0	0	Trappc9;Kcnk9	Transport
DMR7:114121701	7	1.14E+08	700	1	6.53E-07	0	0	Trappc9;U7	
DMR7:114926301	7	1.15E+08	1200	1	2.67E-07	15	1.25	AABR07058410.3	
DMR7:116809401	7	1.17E+08	4100	1	1.17E-07	62	1.512195122	Zc3h3	Transcription
DMR7:117334901	7	1.17E+08	1400	1	6.26E-08	17	1.214285714	AC107096.1;Spatc1	

DMR7:118037201	7	1.18E+08	1500	1	3.67E-07	10	0.666666667		
DMR7:118841401	7	1.19E+08	2400	2	7.84E-22	21	0.875	Apol11a	
DMR7:119308801	7	1.19E+08	700	3	3.75E-08	5	0.714285714	Cacng2	Transport
DMR7:119662301	7	1.2E+08	2000	1	8.53E-07	29	1.45	Kctd17;Tmprss6	Transport;Protease
DMR7:119734501	7	1.2E+08	2600	1	1.86E-07	24	0.923076923	C1qtnf6	Growth Factors & Cytokines
DMR7:119766201	7	1.2E+08	2200	1	5.86E-07	45	2.045454545	Sstr3	Receptor
DMR7:119770001	7	1.2E+08	2600	1	7.91E-07	17	0.653846154	Sstr3	Receptor
DMR7:119899901	7	1.2E+08	4600	1	2.41E-07	81	1.760869565	Elfn2	Receptor
DMR7:119922801	7	1.2E+08	1000	1	7.58E-08	5	0.5		
DMR7:121001501	7	1.21E+08	4300	2	7.32E-12	37	0.860465116	Dnal4;Nptxr	Cytoskeleton
DMR7:121631101	7	1.22E+08	200	1	8.12E-07	6	3		
DMR7:121942501	7	1.22E+08	2300	3	2.26E-08	26	1.130434783	Tnrc6b;Rpl26-ps1	Apoptosis
DMR7:122509001	7	1.23E+08	300	1	1.04E-07	2	0.666666667		
DMR7:123483001	7	1.23E+08	2300	3	2.06E-12	28	1.217391304	Cenpm;LOC688613;LOC108351520	
DMR7:123933601	7	1.24E+08	2000	3	5.81E-10	21	1.05		
DMR7:124353901	7	1.24E+08	2700	1	6.36E-07	48	1.777777778	Ttll1	Cytoskeleton
DMR7:124563801	7	1.25E+08	2800	1	1.40E-09	40	1.428571429	Scube1	Signaling
DMR7:124616201	7	1.25E+08	400	1	1.67E-07	2	0.5	Scube1;AABR07058554.1	Signaling
DMR7:124853301	7	1.25E+08	3400	1	4.55E-07	57	1.676470588	Efcab6	Signaling
DMR7:126211001	7	1.26E+08	2000	1	1.63E-07	10	0.5		
DMR7:126261901	7	1.26E+08	1400	1	3.79E-07	8	0.571428571	Atxn10	
DMR7:127862701	7	1.28E+08	2000	2	1.83E-07	18	0.9		
DMR7:128033501	7	1.28E+08	300	1	3.15E-07	2	0.666666667		
DMR7:128162501	7	1.28E+08	900	2	3.62E-08	3	0.333333333		
DMR7:128273501	7	1.28E+08	400	1	1.50E-07	1	0.25		

DMR7:128847901	7	1.29E+08	900	3	5.87E-09	10	1.111111111		
DMR7:129413901	7	1.29E+08	900	2	1.40E-10	8	0.888888889	LOC108351524	
DMR7:129527801	7	1.3E+08	4300	1	6.85E-09	47	1.093023256		
DMR7:129832501	7	1.3E+08	300	2	7.08E-07	2	0.666666667	AABR07058656.1	
DMR7:129836601	7	1.3E+08	1700	1	4.35E-07	16	0.941176471	AABR07058656.1	
DMR7:131721701	7	1.32E+08	800	2	2.75E-07	5	0.625	Cpne8	Unknown
DMR7:132299001	7	1.32E+08	3800	1	2.30E-10	72	1.894736842	Abcd2	Transport
DMR7:132578901	7	1.33E+08	1600	2	2.67E-08	13	0.8125	Slc2a13	Transport
DMR7:132634301	7	1.33E+08	2500	1	2.64E-07	14	0.56	Slc2a13	Transport
DMR7:132866101	7	1.33E+08	2200	1	2.73E-07	26	1.181818182	Lrrk2	Unknown
DMR7:134069401	7	1.34E+08	500	1	7.72E-07	6	1.2		
DMR7:134233301	7	1.34E+08	400	3	7.16E-15	2	0.5		
DMR7:135057001	7	1.35E+08	2300	1	1.96E-07	27	1.173913043		
DMR7:135662601	7	1.36E+08	2500	1	4.65E-07	30	1.2		
DMR7:136717401	7	1.37E+08	500	2	5.95E-09	0	0	Nell2	Signaling
DMR7:136886601	7	1.37E+08	200	1	9.43E-07	0	0		
DMR7:137221001	7	1.37E+08	500	1	1.63E-08	3	0.6	Ano6	Signaling
DMR7:138174401	7	1.38E+08	2000	1	5.20E-10	23	1.15		
DMR7:140019401	7	1.4E+08	3800	1	3.24E-07	40	1.052631579	AC103129.1;Olr1107	
DMR7:140121501	7	1.4E+08	500	1	6.29E-07	0	0		
DMR7:140264501	7	1.4E+08	2100	1	6.18E-07	32	1.523809524	RGD1305928;Adcy6	Metabolism
DMR7:140360601	7	1.4E+08	300	1	1.33E-07	4	1.333333333	Rnd1	Signaling
DMR7:141260401	7	1.41E+08	3900	1	9.52E-08	40	1.025641026	Aqp5;Aqp6	Transport;Metabolism
DMR7:141311201	7	1.41E+08	1600	1	2.31E-08	16	1	Racgap1	Binding Protein
DMR7:141496701	7	1.41E+08	500	1	6.26E-07	7	1.4	Lima1	Cytoskeleton
DMR7:142800601	7	1.43E+08	3100	2	1.38E-07	36	1.161290323		
DMR7:143243601	7	1.43E+08	3700	4	1.29E-08	21	0.567567568	Rn50_7_1411.3	

DMR7:143611401	7	1.44E+08	400	2	2.96E-09	1	0.25	Krt8;AABR07058925.1	Cytoskeleton
DMR7:143755101	7	1.44E+08	1600	2	1.51E-08	27	1.6875	Igfbp6;Soat2	Receptor;Metabolism
DMR8:1546901	8	1546901	500	2	4.10E-08	1	0.2	Gria4	Signaling
DMR8:2341001	8	2341001	1200	2	2.89E-10	5	0.416666667		
DMR8:3366601	8	3366601	3500	2	3.27E-09	29	0.828571429		
DMR8:4315601	8	4315601	900	1	1.59E-08	3	0.333333333	Vom2r18;Vom2r22	
DMR8:5773901	8	5773901	1300	1	2.39E-07	6	0.461538462	Mmp8	Protease
DMR8:6281001	8	6281001	1200	1	2.72E-08	10	0.833333333	Cep126	
DMR8:7530701	8	7530701	600	1	4.24E-07	3	0.5		
DMR8:7774001	8	7774001	900	2	1.08E-12	8	0.888888889		
DMR8:8400501	8	8400501	500	1	4.71E-09	2	0.4	Cntn5	Extracellular Matrix
DMR8:8594001	8	8594001	1000	2	4.03E-07	17	1.7		
DMR8:9304501	8	9304501	300	1	2.26E-07	0	0		
DMR8:9774701	8	9774701	200	1	4.67E-07	1	0.5		
DMR8:10712901	8	10712901	2800	2	1.27E-07	23	0.821428571		
DMR8:10729601	8	10729601	1000	1	5.47E-07	6	0.6		
DMR8:11946001	8	11946001	700	1	3.91E-07	8	1.142857143	Maml2	Transcription
DMR8:12109201	8	12109201	1700	3	1.38E-07	11	0.647058824	Maml2	Transcription
DMR8:13356601	8	13356601	1200	2	9.17E-09	5	0.416666667	Mre11a;Gpr83	Transcription;Signaling
DMR8:13442001	8	13442001	400	1	4.24E-07	4	1		
DMR8:13581001	8	13581001	4400	1	2.07E-07	78	1.772727273	Panx1	Cell Junction
DMR8:14000501	8	14000501	1200	1	4.48E-10	16	1.333333333		
DMR8:14291901	8	14291901	900	2	1.36E-07	6	0.666666667	AABR07069238.1	
DMR8:14531901	8	14531901	1900	1	2.35E-07	12	0.631578947	Fat3	Transcription
DMR8:14661901	8	14661901	300	1	2.11E-07	1	0.333333333	Fat3	Transcription

DMR8:14989401	8	14989401	400	1	9.68E-07	1	0.25		
DMR8:15288301	8	15288301	1500	1	5.43E-07	3	0.2		
DMR8:15291901	8	15291901	1400	1	1.89E-07	13	0.928571429		
DMR8:15955901	8	15955901	900	1	5.74E-07	5	0.555555556		
DMR8:16670401	8	16670401	1600	1	5.94E-08	8	0.5		
DMR8:16971501	8	16971501	400	2	4.07E-11	1	0.25		
DMR8:17106501	8	17106501	300	1	1.37E-07	2	0.666666667		
DMR8:17651901	8	17651901	600	1	3.33E-07	0	0	7SK	
DMR8:18723801	8	18723801	400	1	1.84E-07	0	0	Olr1121	
DMR8:18887101	8	18887101	2600	1	6.35E-07	45	1.730769231		
DMR8:19064601	8	19064601	100	1	3.91E-07	0	0		
DMR8:19569501	8	19569501	700	5	5.50E-14	2	0.285714286		
DMR8:21833601	8	21833601	3400	1	1.96E-07	28	0.823529412	Col5a3;Rdh8	Extracellular Matrix;Metabolism
DMR8:22323301	8	22323301	3600	1	3.16E-10	55	1.527777778	Kri1;Ap1m2	Transport
DMR8:23009801	8	23009801	1500	4	1.47E-11	7	0.466666667	Rgl3;Ccdc151;Prkcsh	Signaling
DMR8:23282801	8	23282801	400	1	1.53E-07	2	0.5	LOC103690166;Zfp717	
DMR8:23411401	8	23411401	200	2	5.94E-11	1	0.5	RGD1561444	
DMR8:23804001	8	23804001	3700	2	5.12E-08	56	1.513513514	Bbs9	Development
DMR8:23844901	8	23844901	700	1	1.39E-07	1	0.142857143	Bbs9	Development
DMR8:24253601	8	24253601	300	1	7.98E-07	3	1		
DMR8:24892401	8	24892401	1000	1	2.17E-07	4	0.4		
DMR8:26069001	8	26069001	400	2	1.20E-07	1	0.25		
DMR8:26552301	8	26552301	1100	1	7.57E-07	2	0.181818182		
DMR8:26774501	8	26774501	4000	1	6.34E-07	37	0.925		
DMR8:27904701	8	27904701	500	1	1.10E-07	1	0.2	Glb1l3	Golgi

DMR8:28155301	8	28155301	500	2	6.78E-09	2	0.4	Jam3	Cytoskeleton
DMR8:28355401	8	28355401	800	1	3.87E-09	2	0.25	Igsf9b	
DMR8:28896001	8	28896001	1200	1	6.08E-10	19	1.583333333		
DMR8:30676401	8	30676401	5200	1	9.85E-09	78	1.5		
DMR8:31184201	8	31184201	400	1	1.32E-10	0	0		
DMR8:31200101	8	31200101	500	1	1.30E-08	0	0		
DMR8:31989501	8	31989501	2500	2	2.37E-09	25	1	Adamts15	Protease
DMR8:32399401	8	32399401	2100	1	1.65E-07	10	0.476190476		
DMR8:33141101	8	33141101	900	1	1.11E-07	15	1.666666667		
DMR8:33610601	8	33610601	900	1	2.71E-07	14	1.555555556	Fli1	Transcription
DMR8:33696301	8	33696301	2300	1	2.37E-07	23	1		
DMR8:33788801	8	33788801	500	1	3.53E-07	5	1		
DMR8:33836401	8	33836401	2600	1	7.24E-07	38	1.461538462	Ets1	Transcription
DMR8:34060801	8	34060801	600	1	1.70E-10	6	1		
DMR8:34397101	8	34397101	400	2	1.41E-08	1	0.25		
DMR8:35791301	8	35791301	500	1	2.75E-07	9	1.8		
DMR8:36205601	8	36205601	2500	1	1.02E-07	33	1.32	Kirrel3	Extracellular Matrix
DMR8:36675201	8	36675201	3800	1	7.79E-07	33	0.868421053	Cdon;U1	Cytoskeleton
DMR8:36748201	8	36748201	1600	2	1.36E-07	29	1.8125	Ddx25	Transcription
DMR8:36894001	8	36894001	300	1	8.70E-07	2	0.666666667		
DMR8:39313501	8	39313501	800	1	2.21E-07	2	0.25	Fez1	
DMR8:40038601	8	40038601	500	1	2.01E-11	2	0.4	AABR07069791.4;AABR07069791.3	
DMR8:44607901	8	44607901	1700	1	1.21E-10	14	0.823529412		
DMR8:44619601	8	44619601	700	1	4.98E-07	1	0.142857143		
DMR8:45033001	8	45033001	2500	3	1.61E-13	28	1.12		
DMR8:45106101	8	45106101	700	2	1.62E-11	4	0.571428571	RGD1309108	Unknown

DMR8:46597201	8	46597201	2200	2	4.52E-12	27	1.227272727	SNORA17;Tecta	Extracellular Matrix
DMR8:47208201	8	47208201	400	2	1.46E-12	0	0		
DMR8:47663201	8	47663201	400	1	3.46E-08	3	0.75		
DMR8:47828601	8	47828601	2800	4	6.25E-11	44	1.571428571		
DMR8:48071701	8	48071701	400	1	1.21E-07	1	0.25		
DMR8:48280101	8	48280101	1600	2	9.16E-09	22	1.375		
DMR8:48794201	8	48794201	2300	2	8.09E-08	12	0.52173913	U6;AC105645.4;Bcl9l	
DMR8:48850301	8	48850301	2300	1	3.96E-07	25	1.086956522	Cxcr5;AC105645.1	Receptor
DMR8:49536001	8	49536001	1000	1	9.70E-07	2	0.2	LOC102546809	
DMR8:50360601	8	50360601	300	1	5.87E-07	0	0	Sik3	Receptor
DMR8:50600201	8	50600201	7400	1	5.35E-08	138	1.864864865	LOC500990;AC135409.1	
DMR8:50629701	8	50629701	800	2	5.37E-08	0	0		
DMR8:50802301	8	50802301	2200	5	4.22E-09	12	0.545454545		
DMR8:51011201	8	51011201	1300	1	4.57E-07	7	0.538461538		
DMR8:51885501	8	51885501	1100	1	1.13E-11	26	2.363636364		
DMR8:52211201	8	52211201	3400	1	1.95E-07	58	1.705882353		
DMR8:52242101	8	52242101	5000	12	2.22E-24	42	0.84	AABR07070099.1	
DMR8:52435901	8	52435901	900	2	2.05E-08	10	1.111111111		
DMR8:52844901	8	52844901	900	2	2.28E-07	9	1		
DMR8:53035001	8	53035001	1700	1	7.18E-07	33	1.941176471	Zbtb16	Transcription
DMR8:53067101	8	53067101	1200	1	9.99E-07	23	1.916666667	Zbtb16	Transcription
DMR8:53119001	8	53119001	200	1	3.32E-08	1	0.5	Zbtb16	Transcription
DMR8:53196601	8	53196601	3000	1	1.90E-07	26	0.866666667		
DMR8:53278701	8	53278701	2100	1	4.37E-07	28	1.333333333	Htr3b	Receptor
DMR8:53637601	8	53637601	300	1	9.94E-07	1	0.333333333		

DMR8:53835001	8	53835001	1600	1	5.29E-07	19	1.1875	AABR07070129.1;AABR07070131.1;Ncam1	Extracellular Matrix
DMR8:54881001	8	54881001	4100	1	9.19E-12	51	1.243902439	AC141541.1	
DMR8:54915401	8	54915401	600	1	1.74E-07	1	0.166666667	Plet1	
DMR8:56526401	8	56526401	1200	1	9.99E-07	6	0.5		
DMR8:57003001	8	57003001	500	1	2.00E-08	21	4.2		
DMR8:57482401	8	57482401	1400	2	1.58E-09	12	0.857142857		
DMR8:57534501	8	57534501	700	1	6.66E-10	3	0.428571429		
DMR8:58682501	8	58682501	400	1	3.21E-07	7	1.75		
DMR8:58922101	8	58922101	1700	1	1.05E-09	10	0.588235294	Gldn;Dmxl2	Unknown
DMR8:59543301	8	59543301	2500	1	3.48E-07	25	1	Psma4;Rn60_8_0596.7	Protease
DMR8:59874201	8	59874201	600	1	1.27E-07	7	1.166666667		
DMR8:60248501	8	60248501	1600	1	9.04E-07	17	1.0625	Scaper	
DMR8:61320701	8	61320701	3800	1	9.69E-07	42	1.105263158		
DMR8:62958701	8	62958701	2000	1	7.51E-08	23	1.15	Islr	Receptor
DMR8:63121201	8	63121201	7100	2	3.38E-07	71	1	LOC685963	Transcription
DMR8:63189001	8	63189001	1500	2	9.79E-12	17	1.133333333		
DMR8:63795901	8	63795901	500	2	5.78E-09	4	0.8		
DMR8:64302501	8	64302501	200	2	1.08E-08	0	0	Tmem202;SNORA40	
DMR8:64916901	8	64916901	500	1	6.54E-07	3	0.6	Rn50_8_0647.2	
DMR8:65095701	8	65095701	300	1	4.85E-11	2	0.666666667		
DMR8:65132201	8	65132201	1200	2	6.49E-11	8	0.666666667	Rn50_8_0651.1	
DMR8:66506401	8	66506401	300	1	1.01E-08	0	0		
DMR8:66546201	8	66546201	2200	2	5.71E-07	16	0.727272727		
DMR8:66721701	8	66721701	1700	1	8.78E-08	23	1.352941176	LOC102552398	
DMR8:66954001	8	66954001	2800	1	1.85E-07	45	1.607142857	Paqr5	Receptor

DMR8:68885401	8	68885401	300	1	2.33E-07	0	0		
DMR8:69859501	8	69859501	300	1	1.79E-07	15	5		
DMR8:69870201	8	69870201	2000	2	6.26E-08	16	0.8		
DMR8:70116201	8	70116201	600	2	4.85E-09	2	0.333333333	Megf11	Extracellular Matrix
DMR8:70413601	8	70413601	1600	2	1.20E-07	15	0.9375	Dennd4a;Slc24a1	Transport
DMR8:71023801	8	71023801	2100	1	8.14E-07	17	0.80952381	Spg21;Ankdd1a	Unknown
DMR8:71027401	8	71027401	400	1	3.00E-07	0	0	Spg21;Ankdd1a	Unknown
DMR8:71600201	8	71600201	500	2	7.50E-09	6	1.2	Csnk1g1;SNORA17	Signaling
DMR8:71998001	8	71998001	2400	2	2.46E-08	15	0.625		
DMR8:72364701	8	72364701	500	3	1.23E-09	4	0.8		
DMR8:74250801	8	74250801	1100	1	4.56E-07	4	0.363636364		
DMR8:74266701	8	74266701	600	1	3.92E-07	5	0.833333333	AABR07070532.1	
DMR8:75714001	8	75714001	900	1	6.67E-07	17	1.888888889	Anxa2	Signaling
DMR8:76111601	8	76111601	700	1	3.03E-07	7	1	AABR07070587.1	
DMR8:76117801	8	76117801	400	1	1.60E-08	0	0		
DMR8:76170801	8	76170801	400	2	2.48E-09	1	0.25		
DMR8:76182401	8	76182401	500	1	9.14E-07	0	0		
DMR8:76248701	8	76248701	100	1	7.84E-07	3	3	LOC100912027	
DMR8:76650401	8	76650401	400	1	1.53E-08	2	0.5		
DMR8:77166301	8	77166301	500	1	1.57E-07	2	0.4	Adam10	Protease
DMR8:77683901	8	77683901	1000	1	6.99E-07	10	1	Aldh1a2	Metabolism
DMR8:77727601	8	77727601	300	2	8.51E-08	0	0	Aldh1a2	Metabolism
DMR8:77752601	8	77752601	400	1	6.11E-12	0	0		
DMR8:77791301	8	77791301	2900	3	2.69E-08	21	0.724137931		
DMR8:77911501	8	77911501	2700	2	4.13E-08	26	0.962962963		
DMR8:78626101	8	78626101	1400	3	1.08E-07	8	0.571428571	Tcf12	Transcription
DMR8:79519301	8	79519301	2600	1	4.07E-08	13	0.5	Prtg	Immune
DMR8:79758601	8	79758601	1100	2	1.13E-07	15	1.363636364	Rab27a	Signaling

DMR8:79855901	8	79855901	400	1	4.85E-08	0	0	AC128582.1	
DMR8:80441801	8	80441801	300	1	1.47E-07	1	0.333333333	Unc13c	Development
DMR8:80696501	8	80696501	500	1	8.62E-07	2	0.4		
DMR8:81064901	8	81064901	600	1	5.62E-07	5	0.833333333		
DMR8:81305601	8	81305601	500	1	4.67E-07	4	0.8		
DMR8:81840801	8	81840801	1600	1	7.64E-07	13	0.8125		
DMR8:82131301	8	82131301	3700	1	1.33E-09	28	0.756756757	Myo5a	Cytoskeleton
DMR8:82176801	8	82176801	700	1	2.57E-09	7	1	Myo5c	Cytoskeleton
DMR8:82204601	8	82204601	1500	1	6.56E-07	17	1.133333333	Myo5c	Cytoskeleton
DMR8:82314801	8	82314801	1300	1	5.41E-07	14	1.076923077		
DMR8:83469601	8	83469601	300	2	7.48E-08	1	0.333333333		
DMR8:83710401	8	83710401	1400	1	3.71E-09	9	0.642857143		
DMR8:84682201	8	84682201	300	1	5.70E-08	1	0.333333333	AABR07070799.1	
DMR8:86044301	8	86044301	2500	1	4.24E-07	15	0.6	Cd109	Immune
DMR8:86615801	8	86615801	400	1	4.85E-07	1	0.25		
DMR8:87172101	8	87172101	1400	1	3.09E-09	4	0.285714286		
DMR8:88265101	8	88265101	1500	2	2.58E-07	12	0.8		
DMR8:88282901	8	88282901	500	1	3.99E-07	2	0.4		
DMR8:88802801	8	88802801	1700	1	3.80E-07	14	0.823529412		
DMR8:92854901	8	92854901	500	1	4.87E-07	0	0		
DMR8:94005901	8	94005901	600	1	5.02E-07	2	0.333333333	Ube3d	Proteolysis
DMR8:94140101	8	94140101	1900	2	1.18E-14	14	0.736842105	Dopey1	
DMR8:94764001	8	94764001	1500	2	3.74E-09	15	1	Cyb5r4	Metabolism
DMR8:95770301	8	95770301	4300	3	3.03E-08	50	1.162790698		
DMR8:97117501	8	97117501	1500	1	1.01E-09	12	0.8	Ankrd34c	
DMR8:97298801	8	97298801	300	1	7.60E-07	0	0	Rasgrf1	Signaling
DMR8:97356501	8	97356501	900	1	4.88E-07	1	0.111111111	Rasgrf1	Signaling
DMR8:97693201	8	97693201	500	1	8.29E-07	2	0.4		
DMR8:97775501	8	97775501	900	1	3.14E-08	4	0.444444444		

DMR8:98096101	8	98096101	300	1	1.79E-08	0	0		
DMR8:99255201	8	99255201	700	1	3.39E-07	1	0.142857143		
DMR8:99517901	8	99517901	1400	1	3.96E-08	8	0.571428571		
DMR8:101521601	8	1.02E+08	1600	1	1.03E-07	8	0.5		
DMR8:102232701	8	1.02E+08	1200	1	8.96E-08	10	0.833333333		
DMR8:102378201	8	1.02E+08	300	2	6.68E-11	1	0.333333333	Slc9a9	Transport
DMR8:102663901	8	1.03E+08	1700	1	8.00E-07	5	0.294117647	Slc9a9	Transport
DMR8:104406901	8	1.04E+08	700	1	7.70E-07	5	0.714285714	Rasa2	
DMR8:104594201	8	1.05E+08	2600	1	5.76E-07	19	0.730769231	Zbtb38	Transcription
DMR8:105194801	8	1.05E+08	700	1	5.36E-07	7	1	Trim42	Metabolism
DMR8:105468801	8	1.05E+08	300	1	5.20E-08	2	0.666666667	Clstn2	Metabolism
DMR8:105594801	8	1.06E+08	4300	1	3.86E-07	49	1.139534884		
DMR8:106293801	8	1.06E+08	1400	1	6.54E-07	20	1.428571429		
DMR8:106367101	8	1.06E+08	2400	3	2.26E-09	19	0.791666667	Nmnat3	Metabolism
DMR8:106729301	8	1.07E+08	1800	1	9.06E-07	12	0.666666667		
DMR8:109831101	8	1.1E+08	600	1	3.58E-07	1	0.166666667		
DMR8:110107301	8	1.1E+08	900	1	6.21E-07	2	0.222222222	AABR07071335.1	
DMR8:110508801	8	1.11E+08	1500	1	3.94E-07	15	1	Ephb1	Receptor
DMR8:110590901	8	1.11E+08	800	1	4.44E-09	9	1.125	Ephb1	Receptor
DMR8:110646301	8	1.11E+08	1000	1	1.01E-07	4	0.4	Ephb1	Receptor
DMR8:110809701	8	1.11E+08	200	1	9.26E-07	1	0.5	Ephb1	Receptor
DMR8:111389601	8	1.11E+08	3100	2	1.87E-09	27	0.870967742	Ryk	Receptor
DMR8:111521801	8	1.12E+08	3000	1	7.17E-07	41	1.366666667	Slco2a1;SNORA17	Metabolism
DMR8:112196501	8	1.12E+08	500	2	1.05E-10	0	0		
DMR8:112679001	8	1.13E+08	3100	1	3.58E-09	14	0.451612903	Acad11	Cytoskeleton
DMR8:113015901	8	1.13E+08	900	1	5.20E-07	6	0.666666667		
DMR8:114651701	8	1.15E+08	400	1	1.53E-07	3	0.75		
DMR8:114656901	8	1.15E+08	300	1	2.92E-07	1	0.333333333		
DMR8:114975801	8	1.15E+08	200	2	5.71E-10	1	0.5		

DMR8:115300201	8	1.15E+08	1800	2	2.86E-09	7	0.388888889		
DMR8:116024301	8	1.16E+08	1100	1	2.25E-07	11	1	Mapkapk3	Signaling
DMR8:116114201	8	1.16E+08	1000	1	4.45E-08	10	1	RGD1307461	
DMR8:116272701	8	1.16E+08	2300	1	9.61E-07	42	1.826086957	Cacna2d2	Transport
DMR8:117179801	8	1.17E+08	3900	1	3.70E-09	56	1.435897436	Usp4;Ccdc36;LOC498675	Proteolysis
DMR8:118155501	8	1.18E+08	3400	1	1.89E-09	41	1.205882353	Map4;Dhx30	Cytoskeleton;Transcription
DMR8:118716201	8	1.19E+08	3500	1	1.68E-07	53	1.514285714		
DMR8:119676701	8	1.2E+08	700	2	1.13E-07	3	0.428571429		
DMR8:119745601	8	1.2E+08	1000	1	3.51E-08	4	0.4	Dclk3	Cytoskeleton
DMR8:119813101	8	1.2E+08	1100	2	3.34E-08	6	0.545454545	Stac	Unknown
DMR8:120162301	8	1.2E+08	600	1	4.17E-08	12	2		
DMR8:120686501	8	1.21E+08	1800	1	5.87E-07	9	0.5		
DMR8:121073801	8	1.21E+08	2400	1	9.10E-07	16	0.666666667		
DMR8:122460601	8	1.22E+08	800	1	7.47E-07	8	1	Glb1	Metabolism
DMR8:123027301	8	1.23E+08	2700	1	1.86E-07	46	1.703703704		
DMR8:123341701	8	1.23E+08	300	1	4.36E-07	3	1	Stt3b	
DMR8:123534801	8	1.24E+08	1400	1	9.68E-07	26	1.857142857		
DMR8:123935501	8	1.24E+08	2100	2	4.66E-12	23	1.095238095		
DMR8:123975701	8	1.24E+08	1500	1	6.58E-07	5	0.333333333		
DMR8:124067301	8	1.24E+08	1800	2	5.47E-08	16	0.888888889		
DMR8:125551401	8	1.26E+08	1000	3	6.60E-08	5	0.5	Rbms3	Epigenetic
DMR8:125839501	8	1.26E+08	300	1	5.10E-08	0	0		
DMR8:125944801	8	1.26E+08	1600	1	6.57E-07	16	1		
DMR8:126642901	8	1.27E+08	900	1	1.31E-15	12	1.333333333	7SK	
DMR8:126718701	8	1.27E+08	3600	1	4.18E-09	26	0.722222222		
DMR8:126912301	8	1.27E+08	2400	1	5.81E-07	31	1.291666667		
DMR8:127127801	8	1.27E+08	1700	2	1.94E-07	12	0.705882353		

DMR8:127258201	8	1.27E+08	4400	1	1.41E-07	65	1.477272727	Itga9;Golga4	Extracellular Matrix;Binding Protein
DMR8:128296401	8	1.28E+08	2300	1	8.87E-08	32	1.391304348	Scn10a	Transport
DMR8:128381601	8	1.28E+08	1700	4	8.59E-14	18	1.058823529	Scn10a	Transport
DMR8:128390801	8	1.28E+08	2200	1	8.01E-07	23	1.045454545	Scn10a	Transport
DMR8:128422101	8	1.28E+08	400	2	1.21E-10	0	0	Scn10a	Transport
DMR8:128912201	8	1.29E+08	1900	3	2.75E-08	20	1.052631579		
DMR8:128990301	8	1.29E+08	400	2	1.67E-12	2	0.5	Myrip	Metabolism
DMR8:129587901	8	1.3E+08	3600	1	2.22E-07	47	1.305555556		
DMR8:129734101	8	1.3E+08	500	2	3.48E-07	1	0.2	Ulk4	
DMR8:129979601	8	1.3E+08	2500	2	2.17E-07	21	0.84		
DMR8:132523101	8	1.33E+08	600	2	2.03E-11	4	0.666666667	Lars2	Metabolism
DMR8:132550501	8	1.33E+08	400	1	5.27E-07	1	0.25	Limd1	Transcription
DMR8:132688901	8	1.33E+08	3700	1	5.62E-11	47	1.27027027	RGD1566368	
DMR9:2130001	9	2130001	1000	1	2.47E-07	7	0.7	AABR07066096.1	
DMR9:4001401	9	4001401	300	1	6.37E-08	2	0.666666667		
DMR9:4454801	9	4454801	800	2	1.24E-07	2	0.25	Kat2b	Epigenetic
DMR9:4676301	9	4676301	1000	1	1.17E-07	2	0.2	Sult1c2	Metabolism
DMR9:4761601	9	4761601	600	1	2.71E-07	5	0.833333333		
DMR9:5084601	9	5084601	4400	1	1.50E-08	51	1.159090909		
DMR9:5451201	9	5451201	300	1	8.94E-07	1	0.333333333		
DMR9:7518801	9	7518801	1400	1	1.97E-08	11	0.785714286		
DMR9:7769801	9	7769801	600	2	6.83E-08	2	0.333333333	Vom2r77	
DMR9:9437401	9	9437401	1000	2	2.34E-08	7	0.7	Adgre1	
DMR9:9504701	9	9504701	400	1	5.89E-07	2	0.5	Adgre1	
DMR9:9946401	9	9946401	1600	1	8.14E-07	15	0.9375	U1	
DMR9:10062601	9	10062601	1900	2	5.21E-08	18	0.947368421	Clpp;Acer1	EST;Metabolism

DMR9:10577101	9	10577101	1400	1	9.85E-08	7	0.5		
DMR9:10908501	9	10908501	400	2	2.07E-09	1	0.25	AABR07066532.1	
DMR9:10918001	9	10918001	300	1	7.80E-08	0	0		
DMR9:11304401	9	11304401	1000	1	6.84E-09	2	0.2	Uxs1	Metabolism
DMR9:11556701	9	11556701	300	1	3.11E-08	0	0		
DMR9:12031401	9	12031401	300	1	5.64E-07	2	0.666666667		
DMR9:12125501	9	12125501	200	1	7.89E-08	0	0	AABR07066648.1	
DMR9:12824801	9	12824801	2700	2	1.26E-08	37	1.37037037	Rftn1	Unknown
DMR9:13800601	9	13800601	1000	1	3.96E-07	2	0.2		
DMR9:14132801	9	14132801	900	1	2.74E-08	10	1.111111111		
DMR9:14328301	9	14328301	1200	1	7.67E-07	7	0.583333333	SNORA17	
DMR9:14435901	9	14435901	5500	1	1.88E-07	64	1.163636364		
DMR9:15150501	9	15150501	1400	1	3.18E-07	12	0.857142857		
DMR9:15201401	9	15201401	1300	1	2.90E-08	8	0.615384615	Tfeb	Transcription
DMR9:15282701	9	15282701	1100	2	1.84E-08	7	0.636363636	Pgc	Proteolysis
DMR9:15827001	9	15827001	1200	2	5.96E-09	19	1.583333333	U6	
DMR9:15945701	9	15945701	900	1	3.52E-10	11	1.222222222		
DMR9:16377401	9	16377401	1400	1	5.53E-07	15	1.071428571	Gltscr1l	
DMR9:16620201	9	16620201	3900	1	1.70E-09	49	1.256410256	Mea1;Klhdc3;Rrp36;LOC108348250	Unknown;Transcription
DMR9:16834901	9	16834901	3000	1	3.87E-07	64	2.133333333	Cul9;LOC100910410;Dnph1	
DMR9:16856001	9	16856001	6100	1	4.46E-08	160	2.62295082	Dnph1;Ttbk1	Signaling
DMR9:16973701	9	16973701	2300	1	6.13E-07	19	0.826086957	Zfp318	
DMR9:17007101	9	17007101	2700	1	2.01E-07	41	1.518518519		
DMR9:17033201	9	17033201	2000	1	4.61E-08	40	2	Abcc10	Transport
DMR9:17707901	9	17707901	4100	5	1.36E-10	41	1	Mrpl14;Tmem63b	Transcription;EST
DMR9:17891801	9	17891801	1700	1	4.14E-09	19	1.117647059		

DMR9:18656201	9	18656201	5600	1	1.75E-07	76	1.357142857	Runx2	Transcription
DMR9:19110701	9	19110701	1400	1	7.24E-07	6	0.428571429	Clic5	Transport
DMR9:19757801	9	19757801	900	1	8.72E-07	10	1.111111111	Rcan2	Development
DMR9:20000001	9	20000001	1000	1	4.22E-07	8	0.8	Ankrd66	
DMR9:20437101	9	20437101	600	3	8.44E-10	3	0.5		
DMR9:21131101	9	21131101	100	1	6.75E-15	0	0	Ptchd4	Unknown
DMR9:21464301	9	21464301	200	1	5.84E-07	2	1		
DMR9:21476001	9	21476001	500	1	8.74E-07	1	0.2		
DMR9:22322701	9	22322701	1600	1	5.73E-12	8	0.5		
DMR9:25375901	9	25375901	1000	2	1.72E-10	5	0.5		
DMR9:27243901	9	27243901	2500	1	3.71E-07	23	0.92		
DMR9:28290801	9	28290801	200	2	4.52E-08	0	0		
DMR9:28436001	9	28436001	300	1	8.44E-07	2	0.666666667	Rims1	Signaling
DMR9:28825501	9	28825501	1000	3	3.30E-09	7	0.7	Rims1	Signaling
DMR9:29154901	9	29154901	2500	1	1.88E-07	10	0.4		
DMR9:29822301	9	29822301	2700	1	3.15E-07	17	0.62962963		
DMR9:29853601	9	29853601	1200	1	5.36E-09	6	0.5		
DMR9:29871901	9	29871901	3800	1	1.07E-08	40	1.052631579		
DMR9:30373901	9	30373901	1600	1	3.51E-07	13	0.8125		
DMR9:30493401	9	30493401	700	1	1.42E-07	0	0	Col9a1	Extracellular Matrix
DMR9:30837801	9	30837801	1600	2	1.42E-07	14	0.875	Col19a1	Cytoskeleton
DMR9:32010601	9	32010601	300	1	8.19E-07	6	2	Adgrb3	
DMR9:33480501	9	33480501	300	1	1.88E-07	2	0.666666667		
DMR9:35406601	9	35406601	300	1	5.25E-07	1	0.333333333		
DMR9:36082501	9	36082501	400	1	4.99E-07	1	0.25		
DMR9:36552101	9	36552101	300	1	4.99E-07	3	1		
DMR9:36892801	9	36892801	2000	1	1.37E-08	12	0.6		
DMR9:37091801	9	37091801	5600	2	7.23E-10	57	1.017857143	Phf3	

DMR9:39114401	9	39114401	1200	1	1.14E-07	13	1.083333333		
DMR9:39383601	9	39383601	700	1	8.75E-07	3	0.428571429		
DMR9:40327501	9	40327501	400	1	2.79E-07	2	0.5		
DMR9:41645101	9	41645101	100	1	7.26E-07	0	0		
DMR9:41793501	9	41793501	400	1	5.94E-08	8	2		
DMR9:42328901	9	42328901	900	1	6.81E-08	6	0.666666667		
DMR9:42382601	9	42382601	600	2	5.81E-10	1	0.166666667		
DMR9:42547801	9	42547801	2500	1	4.91E-08	25	1		
DMR9:42669801	9	42669801	400	1	8.95E-07	7	1.75		
DMR9:42822101	9	42822101	400	1	9.84E-08	7	1.75	Neurl3	Metabolism
DMR9:43650501	9	43650501	1700	1	2.76E-08	13	0.764705882	Vwa3b	Development
DMR9:43805901	9	43805901	900	2	3.61E-10	5	0.555555556	Cnga3	Receptor
DMR9:44278101	9	44278101	2200	1	9.98E-07	28	1.272727273		
DMR9:45967501	9	45967501	500	2	4.12E-11	1	0.2	Npas2	Transcription
DMR9:46228001	9	46228001	3800	1	1.39E-07	47	1.236842105		
DMR9:46299601	9	46299601	400	1	3.10E-07	5	1.25	Rnf149	Proteolysis
DMR9:46539101	9	46539101	800	1	4.55E-07	5	0.625		
DMR9:46839301	9	46839301	600	1	3.87E-07	4	0.666666667	Il1r2	Receptor
DMR9:47029601	9	47029601	2100	1	2.83E-09	20	0.952380952	Il1r1	Receptor
DMR9:47214901	9	47214901	600	1	4.79E-07	8	1.333333333	Il18r1	Receptor
DMR9:47466201	9	47466201	2000	2	3.98E-09	28	1.4	Slc9a2	Transport
DMR9:47611001	9	47611001	1900	2	1.28E-07	46	2.421052632		
DMR9:48109901	9	48109901	1600	1	9.44E-07	15	0.9375	AABR07067471.2	
DMR9:48147101	9	48147101	800	1	1.36E-07	3	0.375		
DMR9:48984401	9	48984401	1900	1	5.27E-10	13	0.684210526		
DMR9:48994901	9	48994901	1600	1	4.00E-07	14	0.875		
DMR9:49099101	9	49099101	1600	1	8.46E-07	9	0.5625		
DMR9:49998301	9	49998301	1500	2	1.29E-07	12	0.8	AABR07067506.2	
DMR9:51201401	9	51201401	1600	1	2.85E-08	4	0.25		

DMR9:52145401	9	52145401	600	2	1.99E-09	4	0.666666667	Col5a2	Extracellular Matrix
DMR9:53531901	9	53531901	2400	1	9.20E-07	17	0.708333333	Hibch	Metabolism
DMR9:53847001	9	53847001	1600	1	2.45E-08	7	0.4375		
DMR9:55520001	9	55520001	400	1	2.92E-08	2	0.5	Tmeff2	Signaling
DMR9:55646501	9	55646501	900	1	1.32E-08	2	0.222222222	Tmeff2	Signaling
DMR9:56733901	9	56733901	1400	1	9.03E-08	6	0.428571429		
DMR9:57643401	9	57643401	300	1	1.44E-07	2	0.666666667		
DMR9:59737001	9	59737001	400	1	1.29E-07	0	0		
DMR9:60629001	9	60629001	400	1	4.39E-08	3	0.75	Hecw2	Proteolysis
DMR9:62138601	9	62138601	200	1	1.77E-07	0	0		
DMR9:62520101	9	62520101	1200	1	3.30E-07	4	0.333333333		
DMR9:62976001	9	62976001	300	1	2.13E-07	0	0		
DMR9:63548101	9	63548101	3500	1	5.14E-07	40	1.142857143	Satb2	Epigenetic
DMR9:63671801	9	63671801	400	1	3.91E-08	3	0.75		
DMR9:63721701	9	63721701	1100	2	8.28E-10	6	0.545454545		
DMR9:63870001	9	63870001	1300	1	6.32E-07	16	1.230769231	AABR07067788.1	
DMR9:64054501	9	64054501	2600	1	4.26E-07	18	0.692307692	RGD1306941	
DMR9:64674301	9	64674301	1500	1	9.20E-07	9	0.6	Spats2l	Development
DMR9:64758101	9	64758101	300	1	2.09E-08	4	1.333333333	Spats2l	Development
DMR9:65677001	9	65677001	2200	4	5.63E-10	18	0.818181818	Als2cr12	
DMR9:67514701	9	67514701	1400	4	8.15E-12	13	0.928571429		
DMR9:68533601	9	68533601	1200	3	6.09E-14	3	0.25	Pard3b	Cell Junction
DMR9:71451401	9	71451401	2200	1	2.77E-07	44	2	Fzd5	Receptor
DMR9:71717801	9	71717801	1000	1	9.69E-07	3	0.3		
DMR9:72022501	9	72022501	2100	1	5.93E-07	12	0.571428571		
DMR9:72278301	9	72278301	200	1	1.37E-07	0	0		
DMR9:72936501	9	72936501	700	2	8.23E-08	4	0.571428571	Crygf	Unknown
DMR9:73936901	9	73936901	400	1	3.10E-07	1	0.25	Myl1	Cytoskeleton

DMR9:74333101	9	74333101	200	1	5.15E-08	2	1		
DMR9:75242501	9	75242501	1000	2	7.86E-07	5	0.5	Erbb4	Signaling
DMR9:76343901	9	76343901	1400	2	4.24E-08	11	0.785714286		
DMR9:77436501	9	77436501	1600	1	4.30E-07	10	0.625	Spag16	Cytoskeleton
DMR9:78532701	9	78532701	2500	1	2.18E-07	23	0.92	Abca12	Transport
DMR9:78592601	9	78592601	1300	1	2.64E-11	9	0.692307692	Abca12	Transport
DMR9:79092501	9	79092501	500	2	7.87E-09	1	0.2		
DMR9:79807301	9	79807301	400	1	4.20E-07	2	0.5	4-Mar	Metabolism
DMR9:79823301	9	79823301	300	1	2.51E-08	3	1	4-Mar	Metabolism
DMR9:80032901	9	80032901	1300	1	9.46E-07	10	0.769230769		
DMR9:80178401	9	80178401	500	2	3.13E-09	2	0.4		
DMR9:80643801	9	80643801	300	1	2.94E-08	1	0.333333333		
DMR9:80836801	9	80836801	1200	1	2.08E-09	5	0.416666667		
DMR9:82029901	9	82029901	3300	1	6.34E-07	66	2	Wnt6	Signaling
DMR9:82182001	9	82182001	600	1	1.73E-11	4	0.666666667	Cfap65	
DMR9:82284201	9	82284201	600	1	3.87E-07	3	0.5	Nhej1	
DMR9:82536901	9	82536901	300	1	1.24E-09	0	0		
DMR9:82955001	9	82955001	900	2	3.33E-10	3	0.333333333		
DMR9:83557401	9	83557401	1400	1	5.61E-07	13	0.928571429		
DMR9:83978501	9	83978501	2400	2	1.58E-14	17	0.708333333		
DMR9:84238301	9	84238301	700	1	2.70E-08	5	0.714285714	Sgpp2	Signaling
DMR9:86871501	9	86871501	300	1	7.30E-07	0	0	NEWGENE_1305560	
DMR9:90234401	9	90234401	700	3	6.89E-10	4	0.571428571		
DMR9:90508901	9	90508901	5400	1	4.25E-07	66	1.222222222		
DMR9:91390601	9	91390601	300	1	1.81E-07	0	0		
DMR9:92023201	9	92023201	2100	1	6.59E-09	14	0.666666667	Dner	Growth Factors & Cytokines
DMR9:93455101	9	93455101	800	2	5.24E-11	4	0.5	LOC501180	

DMR9:94781201	9	94781201	500	1	3.34E-08	1	0.2	Inpp5d	Signaling
DMR9:94915701	9	94915701	900	1	6.12E-07	14	1.555555556	Atg16l1;SCARNA6	Unknown
DMR9:95272201	9	95272201	1500	1	2.33E-08	15	1	Ugt1a5	
DMR9:95456001	9	95456001	400	2	8.49E-10	1	0.25	Trpm8	Receptor
DMR9:95603201	9	95603201	800	2	8.70E-12	2	0.25		
DMR9:95629201	9	95629201	1500	1	6.82E-07	13	0.866666667		
DMR9:95831201	9	95831201	1500	1	2.67E-09	20	1.333333333		
DMR9:95881401	9	95881401	1100	1	2.26E-08	6	0.545454545		
DMR9:97055201	9	97055201	300	1	4.75E-07	0	0	Gbx2	Transcription
DMR9:97190901	9	97190901	4600	1	6.79E-10	85	1.847826087	Iqca1	
DMR9:97240001	9	97240001	4200	1	3.87E-07	48	1.142857143	Iqca1	
DMR9:97589501	9	97589501	1300	2	2.16E-10	19	1.461538462		
DMR9:98087401	9	98087401	1100	1	6.06E-07	6	0.545454545	Mlph	Metabolism
DMR9:98627701	9	98627701	700	1	3.35E-07	17	2.428571429	U6;Traf3ip1	
DMR9:98712501	9	98712501	2600	4	2.72E-11	32	1.230769231		
DMR9:98804701	9	98804701	2800	1	3.73E-07	47	1.678571429		
DMR9:100269201	9	1E+08	400	1	1.18E-08	0	0	AABR07068350.1	
DMR9:101651601	9	1.02E+08	300	1	5.40E-07	2	0.666666667		
DMR9:104021401	9	1.04E+08	3700	2	1.02E-08	31	0.837837838		
DMR9:105218701	9	1.05E+08	300	1	6.00E-09	0	0		
DMR9:105568101	9	1.06E+08	2000	1	2.60E-08	11	0.55	RGD1560925	
DMR9:106323001	9	1.06E+08	600	1	2.94E-07	4	0.666666667		
DMR9:106557601	9	1.07E+08	1300	1	8.71E-08	7	0.538461538		
DMR9:107207601	9	1.07E+08	1200	2	2.39E-09	6	0.5		
DMR9:109693001	9	1.1E+08	300	1	1.90E-07	0	0		
DMR9:109850301	9	1.1E+08	600	1	1.09E-09	2	0.333333333		
DMR9:109955701	9	1.1E+08	1500	1	2.24E-07	11	0.733333333		
DMR9:110059001	9	1.1E+08	2100	2	8.11E-11	27	1.285714286	Efna5;AABR07068587.1	Signaling

DMR9:110320001	9	1.1E+08	2100	1	1.46E-07	29	1.380952381		
DMR9:112212101	9	1.12E+08	2800	1	9.70E-07	50	1.785714286	AABR07068614.1	
DMR9:113058401	9	1.13E+08	600	2	1.03E-07	7	1.166666667	Tmem232	
DMR9:114731001	9	1.15E+08	1100	1	2.21E-10	15	1.363636364	AABR07068694.1	
DMR9:114981001	9	1.15E+08	900	1	3.85E-07	14	1.555555556	Ptprm	Receptor
DMR9:115820901	9	1.16E+08	1000	1	1.15E-07	7	0.7		
DMR9:115859901	9	1.16E+08	2000	1	2.82E-07	24	1.2	Lrrc30	Unknown
DMR9:116293901	9	1.16E+08	1200	1	5.72E-09	8	0.666666667	AABR07068712.2;AABR07068712.1	
DMR9:116855201	9	1.17E+08	1500	5	3.55E-15	9	0.6	L3mbtl4	Epigenetic
DMR9:117075201	9	1.17E+08	400	1	3.70E-07	1	0.25		
DMR9:119001301	9	1.19E+08	2900	1	5.34E-07	26	0.896551724	Dlgap1	Signaling
DMR9:119286001	9	1.19E+08	1700	1	1.08E-07	12	0.705882353		
DMR9:119301101	9	1.19E+08	900	2	4.61E-08	12	1.333333333	Myl12b	Cytoskeleton
DMR9:120150101	9	1.2E+08	1100	1	1.87E-07	9	0.818181818		
DMR9:120328901	9	1.2E+08	1400	1	2.98E-07	7	0.5	SNORA17	
DMR9:121649601	9	1.22E+08	700	4	9.90E-11	3	0.428571429		
DMR9:121672901	9	1.22E+08	1800	4	5.79E-11	8	0.444444444		
DMR10:1094501	10	1094501	5400	1	2.32E-08	46	0.851851852		
DMR10:2073301	10	2073301	500	2	5.47E-11	1	0.2	RGD1562055	
DMR10:2224601	10	2224601	300	1	1.68E-08	3	1	LOC688899	
DMR10:2770801	10	2770801	900	1	8.51E-08	8	0.888888889		
DMR10:2871201	10	2871201	3300	1	2.26E-07	21	0.636363636		
DMR10:4109501	10	4109501	1900	2	2.98E-07	25	1.315789474	Snx29	Cytoskeleton
DMR10:4112401	10	4112401	600	1	2.99E-09	3	0.5	Snx29	Cytoskeleton
DMR10:4768001	10	4768001	800	4	4.58E-11	4	0.5	Litaf	Transcription
DMR10:5280901	10	5280901	3000	1	4.10E-07	36	1.2		
DMR10:5412301	10	5412301	900	1	1.55E-09	13	1.444444444		
DMR10:6114001	10	6114001	2300	2	1.76E-07	19	0.826086957	Grin2a	Receptor

DMR10:6684601	10	6684601	3000	1	3.61E-07	41	1.366666667	7SK	
DMR10:6944701	10	6944701	800	2	8.77E-09	8	1		
DMR10:7090101	10	7090101	200	1	6.00E-07	5	2.5	Tmem186;Abat	Metabolism
DMR10:7143501	10	7143501	1500	1	6.60E-09	17	1.133333333	Abat	Metabolism
DMR10:7319901	10	7319901	1900	1	6.71E-08	23	1.210526316		
DMR10:7546401	10	7546401	1100	2	1.55E-09	5	0.454545455		
DMR10:8000501	10	8000501	1800	1	9.52E-10	15	0.833333333		
DMR10:8016201	10	8016201	1500	1	1.81E-09	8	0.533333333		
DMR10:8658901	10	8658901	1100	1	1.15E-07	6	0.545454545	Rbfox1	Unknown
DMR10:8721601	10	8721601	600	2	4.29E-10	0	0		
DMR10:8918901	10	8918901	400	1	9.68E-07	0	0		
DMR10:9004401	10	9004401	700	1	1.82E-07	4	0.571428571		
DMR10:9055201	10	9055201	700	2	2.75E-09	4	0.571428571		
DMR10:9378201	10	9378201	1000	1	1.77E-07	10	1		
DMR10:9565701	10	9565701	600	2	4.08E-10	2	0.333333333		
DMR10:9590901	10	9590901	600	1	9.44E-07	5	0.833333333		
DMR10:9946101	10	9946101	800	1	3.13E-08	5	0.625		
DMR10:9986701	10	9986701	1100	1	2.22E-07	36	3.272727273		
DMR10:10139301	10	10139301	2100	1	2.84E-09	23	1.095238095		
DMR10:10228601	10	10228601	2700	2	1.47E-13	36	1.333333333		
DMR10:12248801	10	12248801	600	1	2.41E-08	10	1.666666667		
DMR10:14069801	10	14069801	3300	1	5.57E-08	87	2.636363636	Gfer;Noxo1;Tbl3	Growth Factors & Cytokines;Signaling
DMR10:15070901	10	15070901	400	1	1.29E-08	1	0.25	AABR07029217.4	
DMR10:15472901	10	15472901	5600	9	1.80E-10	41	0.732142857	Decr2;Tmem8a	Metabolism
DMR10:15775401	10	15775401	400	1	5.17E-09	0	0	AABR07029217.2	
DMR10:16136501	10	16136501	1400	1	8.08E-07	17	1.214285714		

DMR10:16487001	10	16487001	400	1	5.84E-07	2	0.5		
DMR10:17679701	10	17679701	1700	3	3.62E-12	14	0.823529412		
DMR10:17863401	10	17863401	800	1	2.29E-09	12	1.5		
DMR10:18735501	10	18735501	2800	1	1.93E-09	27	0.964285714	Kcnip1	Signaling
DMR10:18868301	10	18868301	2100	1	7.30E-09	24	1.142857143	Kcnip1	Signaling
DMR10:20125701	10	20125701	400	1	1.90E-10	1	0.25		
DMR10:20327701	10	20327701	3300	1	6.18E-09	49	1.484848485	Slit3	Development
DMR10:20696501	10	20696501	300	1	4.37E-07	0	0	Wwc1	Unknown
DMR10:22372201	10	22372201	1300	1	9.16E-07	13	1		
DMR10:22460101	10	22460101	2300	1	5.91E-07	9	0.391304348		
DMR10:23046201	10	23046201	1000	2	8.92E-09	9	0.9		
DMR10:23315701	10	23315701	1700	3	5.43E-13	10	0.588235294		
DMR10:24695601	10	24695601	2600	1	8.09E-08	15	0.576923077		
DMR10:26036201	10	26036201	3500	1	3.58E-07	45	1.285714286		
DMR10:27379901	10	27379901	600	1	1.36E-08	4	0.666666667		
DMR10:27425601	10	27425601	3100	3	1.16E-08	14	0.451612903	AABR07029410.1	
DMR10:27647301	10	27647301	600	1	6.43E-07	0	0		
DMR10:28058101	10	28058101	2000	1	3.00E-09	8	0.4	Gabrb2	Receptor
DMR10:28924901	10	28924901	300	1	3.35E-07	1	0.333333333		
DMR10:29216101	10	29216101	1200	1	6.63E-07	14	1.166666667		
DMR10:29923401	10	29923401	700	3	4.04E-10	7	1		
DMR10:30638601	10	30638601	2200	1	6.51E-07	28	1.272727273		
DMR10:30667601	10	30667601	1200	1	7.63E-07	11	0.916666667		
DMR10:30765801	10	30765801	3000	2	1.41E-07	28	0.933333333		
DMR10:31579201	10	31579201	1100	3	7.72E-09	7	0.636363636	Havcr2	Receptor
DMR10:32020201	10	32020201	600	3	2.39E-08	1	0.166666667		
DMR10:32536501	10	32536501	1400	1	3.14E-08	4	0.285714286		
DMR10:33870601	10	33870601	300	2	2.91E-11	0	0		
DMR10:34124601	10	34124601	1900	1	1.04E-10	37	1.947368421		

DMR10:34270901	10	34270901	400	1	4.34E-07	3	0.75	Ifi47	Immune
DMR10:34801801	10	34801801	900	1	7.03E-07	3	0.333333333	Olr1401	
DMR10:35386001	10	35386001	1500	1	1.33E-07	14	0.933333333	Rasgef1c	Signaling
DMR10:36047901	10	36047901	1200	1	2.37E-07	15	1.25		
DMR10:37359801	10	37359801	2900	1	1.24E-07	42	1.448275862		
DMR10:37381201	10	37381201	400	1	9.11E-07	0	0		
DMR10:37859101	10	37859101	300	1	1.19E-09	1	0.333333333		
DMR10:38094001	10	38094001	1200	1	8.78E-07	6	0.5	Fstl4	Hormone
DMR10:38474701	10	38474701	3400	1	4.28E-07	55	1.617647059	Fstl4	Hormone
DMR10:39136401	10	39136401	2200	1	3.60E-07	28	1.272727273		
DMR10:39687401	10	39687401	200	1	7.52E-08	4	2	Acsl6	Metabolism
DMR10:40460501	10	40460501	3300	1	7.54E-07	44	1.333333333	Gm2a;Slc36a3	
DMR10:40960401	10	40960401	200	1	7.70E-08	1	0.5	Glra1	Receptor
DMR10:41267501	10	41267501	1200	1	8.33E-08	6	0.5		
DMR10:41394201	10	41394201	1500	1	3.58E-07	27	1.8	Nmur2	Signaling
DMR10:41982501	10	41982501	300	1	1.30E-07	1	0.333333333		
DMR10:42423401	10	42423401	1400	1	8.10E-08	3	0.214285714		
DMR10:42961301	10	42961301	2800	1	7.12E-08	39	1.392857143		
DMR10:43136301	10	43136301	1100	1	1.91E-07	11	1	Galnt10	Metabolism
DMR10:43272701	10	43272701	4400	1	3.59E-08	56	1.272727273		
DMR10:43298501	10	43298501	4000	4	1.02E-15	35	0.875		
DMR10:43422901	10	43422901	1700	2	6.58E-14	13	0.764705882		
DMR10:43735901	10	43735901	900	2	4.59E-07	9	1	RGD1308564;Zfp692	Unknown;Transcription
DMR10:43908201	10	43908201	1300	1	6.56E-08	10	0.769230769	Olr1414	
DMR10:44815301	10	44815301	3100	1	2.04E-07	37	1.193548387	Olr1460	Receptor
DMR10:45239201	10	45239201	300	1	1.28E-07	4	1.333333333	Zfp39	Transcription
DMR10:45362801	10	45362801	300	1	1.80E-07	1	0.333333333	Obscn	Unknown
DMR10:45464201	10	45464201	800	1	7.36E-07	7	0.875	Obscn	Unknown

DMR10:45906901	10	45906901	800	1	3.68E-09	10	1.25	Nlrp3	Unknown
DMR10:46040401	10	46040401	300	1	4.13E-07	2	0.666666667	Mprip	
DMR10:46859001	10	46859001	4000	1	4.40E-08	76	1.9	Myo15a	Cytoskeleton
DMR10:47304201	10	47304201	2300	1	3.55E-07	25	1.086956522	Kcnj12	Transport
DMR10:48078001	10	48078001	1900	1	1.70E-08	16	0.842105263	Ulk2	Signaling
DMR10:48809501	10	48809501	1700	1	4.29E-08	11	0.647058824	Pigl	Metabolism
DMR10:49075001	10	49075001	1400	1	2.78E-07	12	0.857142857		
DMR10:49470101	10	49470101	1200	1	9.08E-07	10	0.833333333	Tekt3	Cytoskeleton
DMR10:50989701	10	50989701	1300	1	2.90E-07	10	0.769230769	Hs3st3a1	Metabolism
DMR10:52208201	10	52208201	700	1	2.84E-08	9	1.285714286	Map2k4	Signaling
DMR10:52959401	10	52959401	4400	1	1.63E-07	33	0.75	Shisa6	Development
DMR10:53558501	10	53558501	1500	1	1.00E-07	19	1.266666667		
DMR10:53811701	10	53811701	800	1	3.15E-08	5	0.625	Myh2;Myh8	Cytoskeleton
DMR10:53942301	10	53942301	1500	1	1.16E-07	6	0.4		
DMR10:54246801	10	54246801	300	1	4.66E-09	1	0.333333333	Rcvrn	Signaling
DMR10:54871701	10	54871701	2500	2	3.67E-08	29	1.16	Ntn1	Development
DMR10:56869401	10	56869401	2800	1	7.47E-09	28	1	Alox12	Metabolism
DMR10:56891101	10	56891101	1600	1	9.86E-07	5	0.3125		
DMR10:57454001	10	57454001	3000	2	1.36E-08	32	1.066666667	Rabep1	
DMR10:57748401	10	57748401	2100	1	3.63E-07	19	0.904761905	Nlrp1a	
DMR10:58038301	10	58038301	1800	1	2.14E-08	18	1		
DMR10:58294601	10	58294601	2200	1	3.47E-13	16	0.727272727		
DMR10:59016701	10	59016701	800	1	8.29E-07	9	1.125	Mybbp1a;Spns2	Transcription
DMR10:61140001	10	61140001	400	1	4.02E-07	1	0.25	AABR07029955.1	
DMR10:62842601	10	62842601	600	2	5.47E-08	0	0		
DMR10:64893101	10	64893101	800	2	2.24E-14	3	0.375	Sez6	Development
DMR10:65605101	10	65605101	1500	1	2.36E-07	25	1.666666667	Pigs;Unc119	Metabolism;Receptor

DMR10:65733501	10	65733501	300	1	7.46E-07	1	0.333333333	Slc46a1;Sarm1	Metabolism;Signaling
DMR10:66235201	10	66235201	300	1	4.57E-07	0	0	LOC497963;Lgals5	Metabolism
DMR10:66989901	10	66989901	2200	3	1.04E-13	29	1.318181818	Rab11fip4;U6	Unknown
DMR10:67264201	10	67264201	1200	3	1.52E-12	11	0.916666667	Utp6	Transcription
DMR10:67491501	10	67491501	400	1	3.52E-07	6	1.5	SNORA43;SNORA17;Adap2	Transcription
DMR10:68697801	10	68697801	2800	1	5.04E-08	27	0.964285714		
DMR10:69209401	10	69209401	2100	3	2.53E-08	16	0.761904762		
DMR10:69816101	10	69816101	2100	1	4.96E-09	22	1.047619048		
DMR10:70352101	10	70352101	4600	11	3.89E-12	9	0.195652174	Slfn13;Rn50_10_0700.5	Translation
DMR10:70403701	10	70403701	3500	1	4.30E-10	31	0.885714286	Rn50_10_0701.2;Slfn4	Translation
DMR10:70873501	10	70873501	400	1	5.45E-10	0	0	Ccl3;Rn50_10_0706.2	Signaling
DMR10:71024801	10	71024801	800	1	6.39E-07	11	1.375		
DMR10:71980201	10	71980201	400	2	1.28E-07	1	0.25		
DMR10:72076101	10	72076101	400	1	5.00E-07	2	0.5		
DMR10:72393001	10	72393001	1200	1	1.07E-07	7	0.583333333	Usp32	
DMR10:74453701	10	74453701	1500	1	7.26E-07	22	1.466666667	Trim37	Development
DMR10:74558401	10	74558401	800	1	4.84E-09	12	1.5	Trim37;Ppm1e	Development;Signaling
DMR10:74928801	10	74928801	300	1	9.82E-13	0	0	Hsf5	Transcription
DMR10:76173601	10	76173601	1200	1	2.97E-07	2	0.166666667	Akap1	Signaling
DMR10:76823001	10	76823001	5000	1	5.14E-07	88	1.76	Nog	Signaling
DMR10:77017001	10	77017001	2800	1	4.03E-09	32	1.142857143		
DMR10:77072601	10	77072601	2600	1	1.70E-07	22	0.846153846	AABR07030235.1	
DMR10:77557301	10	77557301	2200	1	3.88E-08	28	1.272727273		

DMR10:80004901	10	80004901	3700	1	6.36E-08	35	0.945945946		
DMR10:82718301	10	82718301	3300	1	3.16E-08	38	1.151515152		
DMR10:83217401	10	83217401	2700	1	1.11E-07	27	1	Slc35b1	Transport
DMR10:83559001	10	83559001	1500	1	4.18E-08	3	0.2	Zfp652	Transcription
DMR10:83793801	10	83793801	2400	1	2.21E-07	23	0.958333333	Igf2bp1	Transcription
DMR10:83887001	10	83887001	1200	1	3.59E-07	18	1.5	Ube2z;Atp5g1	Metabolism
DMR10:83966701	10	83966701	1400	1	2.60E-08	10	0.714285714	Ttll6	Cytoskeleton
DMR10:84159001	10	84159001	900	1	1.04E-08	19	2.111111111	Hoxb6;Hoxb5;Mir10a;Hoxb4	Transcription
DMR10:84878001	10	84878001	1500	1	2.82E-07	29	1.933333333	Prr15l;Pnpo	Metabolism
DMR10:85292101	10	85292101	3600	2	3.51E-07	69	1.916666667	Gpr179;Socs7	Receptor;Signaling
DMR10:85429701	10	85429701	1300	1	1.53E-07	16	1.230769231	Arhgap23;4933428G20Rik	Signaling
DMR10:85993701	10	85993701	2000	2	3.96E-10	44	2.2	Stac2	Signaling
DMR10:86305601	10	86305601	5700	1	2.97E-07	110	1.929824561	Ppp1r1b;Stard3	Signaling;Metabolism
DMR10:86682501	10	86682501	600	1	5.71E-07	11	1.833333333	Thra;Nr1d1	Receptor;Transcription
DMR10:87207401	10	87207401	1600	1	2.01E-08	13	0.8125		
DMR10:87342801	10	87342801	900	2	3.26E-09	8	0.888888889	Krt12;Krt20	Cytoskeleton
DMR10:87929001	10	87929001	6500	3	2.45E-08	76	1.169230769	Krtap16-1;Rn50_10_0878.7;Krtap17-1;Rn50_10_0878.6	
DMR10:87983501	10	87983501	400	1	2.80E-07	0	0	Krt32	Cytoskeleton
DMR10:88102301	10	88102301	1000	1	1.98E-07	8	0.8	Krt14;Krt9	Cytoskeleton
DMR10:88163301	10	88163301	1600	1	8.38E-07	35	2.1875	Krt17;Krt42	Cytoskeleton

DMR10:88235901	10	88235901	2200	1	4.02E-07	32	1.454545455	Eif1;Gast	Translation;Signaling
DMR10:88259601	10	88259601	1900	1	3.03E-07	33	1.736842105	Hap1	Transcription
DMR10:88661101	10	88661101	3800	1	8.36E-08	73	1.921052632	Kcnh4;Hcrt;Ghdc	Transport;Signaling
DMR10:89745901	10	89745901	2800	1	1.08E-07	24	0.857142857		
DMR10:89979301	10	89979301	200	1	7.97E-10	2	1	Cd300lg	Receptor
DMR10:89980801	10	89980801	1000	1	4.65E-11	7	0.7	Cd300lg	Receptor
DMR10:90570301	10	90570301	1800	1	4.56E-07	27	1.5	AABR07030494.1	
DMR10:90590401	10	90590401	500	3	1.94E-10	0	0		
DMR10:92851201	10	92851201	600	1	5.95E-09	5	0.833333333		
DMR10:95844501	10	95844501	1700	1	3.04E-07	17	1	Helz	Transcription
DMR10:96127001	10	96127001	2300	1	1.47E-07	37	1.608695652	Cacng5	Transport
DMR10:96145001	10	96145001	1900	1	4.00E-07	12	0.631578947		
DMR10:96643601	10	96643601	1700	1	2.13E-07	24	1.411764706	Apoh	Binding Protein
DMR10:97245701	10	97245701	700	1	9.52E-07	11	1.571428571	Axin2	Signaling
DMR10:97360901	10	97360901	2300	1	1.94E-07	25	1.086956522		
DMR10:97771501	10	97771501	800	1	1.33E-07	8	1	Arsg	Metabolism
DMR10:97798101	10	97798101	1000	3	3.84E-15	7	0.7	Arsg	Metabolism
DMR10:97972401	10	97972401	1900	2	2.33E-07	20	1.052631579	Fam20a	Unknown
DMR10:98045101	10	98045101	600	1	9.84E-07	1	0.166666667		
DMR10:98218801	10	98218801	600	2	2.95E-08	0	0		
DMR10:98466301	10	98466301	1800	2	1.37E-09	21	1.166666667	Abca9;Abca6	Transport
DMR10:98749501	10	98749501	1200	1	2.10E-07	11	0.916666667	Map2k6	Signaling
DMR10:99738801	10	99738801	3800	6	5.55E-10	22	0.578947368	AABR07030690.1	
DMR10:99763901	10	99763901	300	1	6.33E-07	0	0	AABR07030690.1	
DMR10:99781701	10	99781701	1700	1	8.28E-07	5	0.294117647		
DMR10:10050170	10	1.01E+08	1400	2	1.13E-07	23	1.642857143		
DMR10:10095390	10	1.01E+08	2100	1	6.03E-07	33	1.571428571		

DMR10:10130000	10	1.01E+08	1700	1	2.86E-07	18	1.058823529	Sox9	Transcription
DMR10:10142580	10	1.01E+08	400	1	1.44E-08	0	0		
DMR10:10158720	10	1.02E+08	4300	1	5.98E-07	78	1.813953488		
DMR10:10182860	10	1.02E+08	1400	1	1.77E-09	16	1.142857143	Slc39a11	Metabolism
DMR10:10183150	10	1.02E+08	2800	3	3.15E-15	71	2.535714286	Slc39a11	Metabolism
DMR10:10185250	10	1.02E+08	5100	1	4.50E-08	73	1.431372549	Slc39a11	Metabolism
DMR10:10199030	10	1.02E+08	300	1	7.51E-07	4	1.333333333	Slc39a11;AABR07072200.1	Metabolism
DMR10:10223450	10	1.02E+08	700	1	2.42E-09	4	0.571428571	;Cdc42ep4	Signaling
DMR10:10255000	10	1.03E+08	1900	3	2.87E-13	36	1.894736842	Sdk2	Development
DMR10:10283820	10	1.03E+08	1200	1	1.70E-08	10	0.833333333		
DMR10:10287090	10	1.03E+08	2300	1	3.19E-07	36	1.565217391	AC118434.2	
DMR10:10297330	10	1.03E+08	2200	1	7.21E-07	22	1		
DMR10:10344250	10	1.03E+08	300	1	2.72E-08	0	0	Cd300a	Receptor
DMR10:10365530	10	1.04E+08	600	2	1.26E-09	10	1.666666667		
DMR10:10411020	10	1.04E+08	900	1	1.28E-07	14	1.555555556	Nup85	Unknown
DMR10:10661270	10	1.07E+08	300	1	1.67E-07	1	0.333333333		
DMR10:10690380	10	1.07E+08	400	1	2.64E-07	0	0		
DMR10:10704820	10	1.07E+08	400	1	8.19E-07	2	0.5	Dnah17	Cytoskeleton
DMR10:10710740	10	1.07E+08	1900	1	2.36E-07	29	1.526315789	Dnah17	Cytoskeleton
DMR10:10745390	10	1.07E+08	1100	1	1.26E-07	16	1.454545455	Cant1;C1qtnf1	Metabolism;Growth Factors & Cytokines
DMR10:10752160	10	1.08E+08	1800	2	4.06E-10	30	1.666666667	Engase;Rbfox3	Metabolism
DMR10:10754100	10	1.08E+08	900	2	1.56E-10	4	0.444444444	Rbfox3	
DMR10:10784190	10	1.08E+08	5600	2	2.04E-07	107	1.910714286		
DMR10:10874770	10	1.09E+08	1400	1	6.54E-07	14	1	Rptor	
DMR10:10887380	10	1.09E+08	400	1	7.03E-08	1	0.25	Rptor	
DMR10:10926140	10	1.09E+08	2200	1	3.12E-08	28	1.272727273	Cep131;Tepsin	

DMR10:10982810	10	1.1E+08	900	1	1.61E-11	2	0.222222222	Pycr1;Myadml2;Notum	Metabolism
DMR10:11023940	10	1.1E+08	1600	1	3.43E-08	13	0.8125	Cd7	Receptor
DMR10:11132420	10	1.11E+08	1600	1	5.29E-07	13	0.8125		
DMR10:11140070	10	1.11E+08	1800	1	2.38E-08	12	0.666666667	AABR07030977.1	
DMR10:11196650	10	1.12E+08	1200	2	1.04E-11	7	0.583333333		
DMR11:65101	11	65101	1500	2	1.00E-09	15	1		
DMR11:584901	11	584901	1100	1	8.39E-07	0	0	Epha3	Receptor
DMR11:1193701	11	1193701	400	1	3.00E-08	2	0.5		
DMR11:6089001	11	6089001	2300	4	1.75E-09	20	0.869565217		
DMR11:6277101	11	6277101	2500	1	5.29E-07	16	0.64		
DMR11:6973501	11	6973501	500	1	1.85E-08	0	0		
DMR11:7280801	11	7280801	800	3	7.43E-09	0	0	Gbe1	Metabolism
DMR11:8842801	11	8842801	600	1	6.92E-07	2	0.333333333		
DMR11:9512101	11	9512101	1600	1	1.11E-08	4	0.25		
DMR11:10330501	11	10330501	1500	1	3.94E-07	11	0.733333333		
DMR11:10413401	11	10413401	900	1	5.54E-09	5	0.555555556		
DMR11:11115001	11	11115001	700	3	1.95E-09	4	0.571428571	Robo2	Receptor
DMR11:11730501	11	11730501	1000	1	3.85E-07	6	0.6		
DMR11:11829901	11	11829901	8000	1	5.55E-07	88	1.1		
DMR11:14683001	11	14683001	1800	3	5.25E-08	21	1.166666667	Nrip1	Transcription
DMR11:15505801	11	15505801	1900	1	8.94E-07	16	0.842105263	Usp25	Protease
DMR11:16103001	11	16103001	500	1	8.12E-07	4	0.8	Mir3588	
DMR11:16272901	11	16272901	600	1	5.63E-07	1	0.166666667	AABR07033292.1	
DMR11:17023701	11	17023701	1800	1	3.58E-07	6	0.333333333		
DMR11:18850901	11	18850901	500	3	6.70E-10	0	0		
DMR11:18873301	11	18873301	1500	1	6.44E-07	21	1.4		
DMR11:19423501	11	19423501	500	2	6.12E-10	2	0.4		
DMR11:21388101	11	21388101	800	1	1.41E-07	12	1.5		

DMR11:21585401	11	21585401	2400	1	8.14E-07	19	0.791666667		
DMR11:21772301	11	21772301	1800	1	4.28E-07	15	0.833333333		
DMR11:22092101	11	22092101	1000	1	1.39E-08	10	1		
DMR11:23012401	11	23012401	300	1	7.28E-08	3	1		
DMR11:23261701	11	23261701	300	1	8.14E-07	4	1.333333333	U4	
DMR11:24337901	11	24337901	1000	2	3.24E-08	2	0.2		
DMR11:25708501	11	25708501	1200	3	1.96E-12	5	0.416666667		
DMR11:25828601	11	25828601	700	1	5.05E-08	0	0		
DMR11:26519501	11	26519501	300	1	2.70E-07	1	0.333333333		
DMR11:27864801	11	27864801	2700	1	4.82E-07	28	1.037037037	Grik1	Receptor
DMR11:28704401	11	28704401	1200	2	3.54E-08	7	0.583333333		
DMR11:29314401	11	29314401	1200	1	9.68E-07	8	0.666666667		
DMR11:29918001	11	29918001	3200	2	7.01E-09	45	1.40625		
DMR11:30449001	11	30449001	5000	1	4.20E-08	63	1.26		
DMR11:30725401	11	30725401	4000	5	7.51E-09	30	0.75		
DMR11:30768301	11	30768301	3900	1	9.95E-07	26	0.666666667		
DMR11:30824801	11	30824801	800	1	5.64E-08	8	1		
DMR11:32060701	11	32060701	700	2	3.21E-08	19	2.714285714	Itsn1	Signaling
DMR11:33050901	11	33050901	1500	1	9.54E-08	8	0.533333333		
DMR11:33117101	11	33117101	3200	2	3.24E-07	45	1.40625		
DMR11:33335801	11	33335801	5500	1	9.86E-07	73	1.327272727		
DMR11:33347001	11	33347001	300	2	1.11E-09	1	0.333333333		
DMR11:33649401	11	33649401	1800	1	4.95E-07	18	1		
DMR11:34155201	11	34155201	2500	3	7.69E-09	29	1.16		
DMR11:34194001	11	34194001	300	1	8.96E-08	5	1.666666667		
DMR11:35033801	11	35033801	3100	2	7.82E-10	50	1.612903226	Kcnj6	Metabolism
DMR11:35060601	11	35060601	600	4	1.67E-09	1	0.166666667	Kcnj6	Metabolism
DMR11:35095501	11	35095501	2800	2	1.09E-08	31	1.107142857	Kcnj6	Metabolism
DMR11:35326501	11	35326501	900	1	4.46E-07	7	0.777777778	AABR07033688.1	

DMR11:35542701	11	35542701	400	1	9.33E-09	0	0		
DMR11:35871801	11	35871801	300	1	4.71E-07	0	0		
DMR11:36005501	11	36005501	1000	1	2.43E-07	3	0.3		
DMR11:36160001	11	36160001	900	3	9.97E-08	11	1.222222222		
DMR11:36176401	11	36176401	900	3	1.87E-08	5	0.555555556		
DMR11:36193501	11	36193501	1200	1	6.84E-07	4	0.333333333		
DMR11:36215801	11	36215801	2200	1	1.18E-09	10	0.454545455		
DMR11:36310701	11	36310701	800	1	4.60E-07	7	0.875		
DMR11:36655401	11	36655401	700	1	9.21E-07	5	0.714285714	B3galt5	Metabolism
DMR11:36872401	11	36872401	500	2	2.34E-08	0	0	Pcp4	
DMR11:36986101	11	36986101	1600	1	6.84E-08	22	1.375		
DMR11:37499301	11	37499301	700	1	1.46E-08	5	0.714285714	AABR07033720.1	
DMR11:37607001	11	37607001	1700	1	4.19E-07	4	0.235294118	AABR07033720.1	
DMR11:37882901	11	37882901	1600	2	2.46E-08	14	0.875	Bace2;Mx1	Metabolism
DMR11:38208301	11	38208301	400	2	6.42E-07	1	0.25		
DMR11:39639901	11	39639901	500	2	2.05E-08	0	0	AABR07033829.1	
DMR11:39827401	11	39827401	400	1	3.62E-07	1	0.25		
DMR11:40668101	11	40668101	500	2	6.80E-09	0	0		
DMR11:41619301	11	41619301	400	1	3.15E-08	1	0.25		
DMR11:42175701	11	42175701	600	2	3.93E-12	0	0		
DMR11:43343701	11	43343701	1600	1	2.11E-07	7	0.4375	Olr1541	Receptor
DMR11:43693201	11	43693201	100	1	9.53E-19	0	0	Olr1558;Olr1559	Receptor
DMR11:43782301	11	43782301	800	1	2.03E-08	1	0.125	Olr1561	
DMR11:44167901	11	44167901	1900	1	3.27E-07	11	0.578947368		
DMR11:44774801	11	44774801	100	1	1.29E-09	0	0		
DMR11:44813101	11	44813101	1100	2	6.65E-08	4	0.363636364		
DMR11:46139901	11	46139901	4100	1	1.91E-07	40	0.975609756	Adgrg7	
DMR11:46453901	11	46453901	800	1	4.63E-08	3	0.375		
DMR11:46509301	11	46509301	400	2	4.28E-09	1	0.25		

DMR11:47867201	11	47867201	300	1	5.55E-07	4	1.333333333		
DMR11:48430301	11	48430301	600	2	1.36E-08	1	0.166666667		
DMR11:49444601	11	49444601	300	1	1.28E-07	0	0		
DMR11:49716601	11	49716601	2300	1	1.50E-08	11	0.47826087	Hmgb2	Epigenetic
DMR11:51221201	11	51221201	700	1	6.84E-07	1	0.142857143	AABR07034073.1	
DMR11:51291301	11	51291301	1100	2	3.04E-07	7	0.636363636		
DMR11:52139101	11	52139101	1000	2	5.24E-09	8	0.8		
DMR11:52159801	11	52159801	200	1	2.98E-07	0	0		
DMR11:52261601	11	52261601	1400	1	9.27E-07	6	0.428571429		
DMR11:52517001	11	52517001	300	1	4.26E-07	1	0.333333333		
DMR11:52737101	11	52737101	1400	1	1.99E-07	16	1.142857143		
DMR11:54323601	11	54323601	300	1	8.06E-07	0	0	Myh15	Cytoskeleton
DMR11:54769701	11	54769701	1000	1	3.49E-07	3	0.3	Morc1	
DMR11:54887601	11	54887601	200	1	1.77E-07	1	0.5	Morc1	
DMR11:55647701	11	55647701	100	1	4.01E-07	0	0		
DMR11:55735701	11	55735701	800	1	1.33E-07	9	1.125		
DMR11:56490301	11	56490301	1800	1	2.67E-08	4	0.222222222		
DMR11:56887301	11	56887301	1300	1	3.09E-08	11	0.846153846		
DMR11:56989601	11	56989601	1000	1	2.88E-07	12	1.2		
DMR11:57325301	11	57325301	400	2	7.82E-10	0	0	Phldb2	
DMR11:57350601	11	57350601	200	2	3.51E-28	0	0	Phldb2	
DMR11:57858301	11	57858301	300	1	7.54E-08	3	1		
DMR11:59539101	11	59539101	400	2	7.18E-09	0	0		
DMR11:59971601	11	59971601	400	3	3.40E-12	0	0		
DMR11:60121601	11	60121601	300	1	9.85E-08	1	0.333333333	Tmprss7	Protease
DMR11:60204701	11	60204701	300	1	3.46E-07	0	0	Slc9c1	
DMR11:60557901	11	60557901	2900	1	1.50E-07	25	0.862068966		
DMR11:60649801	11	60649801	2600	1	3.62E-07	36	1.384615385	Ccdc80	Transcription
DMR11:62037201	11	62037201	800	1	1.87E-07	4	0.5	Gramd1c;Zbtb20	Transcription

DMR11:62341801	11	62341801	400	1	1.21E-07	3	0.75	Gramd1c;Zbtb20	Transcription
DMR11:63090201	11	63090201	1400	1	1.33E-10	6	0.428571429		
DMR11:64047701	11	64047701	600	1	3.75E-07	2	0.333333333		
DMR11:64481001	11	64481001	400	3	1.94E-15	4	1	RGD1563835;RGD1306995	Translation
DMR11:64959701	11	64959701	2300	2	3.35E-10	21	0.913043478	Popdc2;Cox17	Development;Binding Protein
DMR11:64968801	11	64968801	2300	1	3.97E-07	22	0.956521739	Cox17;Maats1	Binding Protein
DMR11:65014601	11	65014601	1900	2	1.80E-07	18	0.947368421	Maats1;Nr1i2	Receptor
DMR11:65033101	11	65033101	400	2	2.85E-07	0	0	Nr1i2	Receptor
DMR11:65055301	11	65055301	2400	1	2.58E-07	46	1.916666667	Nr1i2;Gsk3b	Receptor;Signaling
DMR11:65684501	11	65684501	700	4	5.00E-08	1	0.142857143		
DMR11:66627601	11	66627601	700	2	5.36E-11	3	0.428571429	Polq	Transcription
DMR11:67053601	11	67053601	300	1	9.49E-07	5	1.666666667		
DMR11:67240401	11	67240401	500	1	8.78E-07	0	0	Casr;7SK	Receptor
DMR11:67644001	11	67644001	2200	1	8.94E-07	27	1.227272727	Fam162a;Wdr5b;Kpna1	Unknown;Metabolism
DMR11:68242801	11	68242801	300	1	5.67E-07	3	1	Dirc2	Development
DMR11:68844701	11	68844701	1900	1	2.50E-08	29	1.526315789	Adcy5	Signaling
DMR11:69944901	11	69944901	1800	1	2.67E-07	20	1.111111111	Kalrn	Signaling
DMR11:70328401	11	70328401	2100	1	2.35E-07	17	0.80952381	Heg1	
DMR11:70459301	11	70459301	400	1	2.82E-10	2	0.5	Slc12a8	Transport
DMR11:71487601	11	71487601	300	1	2.06E-08	3	1		
DMR11:72677401	11	72677401	1800	1	8.43E-08	21	1.166666667		
DMR11:73044601	11	73044601	300	1	8.94E-07	9	3	Ppp1r2	Signaling
DMR11:73319301	11	73319301	2800	1	4.67E-07	41	1.464285714	Xxylt1	
DMR11:74572801	11	74572801	1700	1	3.58E-08	24	1.411764706		
DMR11:74993601	11	74993601	2300	1	1.61E-07	23	1	Atp13a5	Transport

DMR11:77871501	11	77871501	300	1	2.69E-08	0	0		
DMR11:77977201	11	77977201	700	1	6.99E-07	7	1		
DMR11:78590801	11	78590801	2400	5	1.75E-15	28	1.166666667		
DMR11:78723501	11	78723501	800	3	1.81E-10	0	0	Tprg1	Unknown
DMR11:79134101	11	79134101	1900	1	2.03E-07	17	0.894736842	AABR07034573.1	
DMR11:80842101	11	80842101	1500	1	1.73E-09	8	0.533333333		
DMR11:81011301	11	81011301	800	2	5.69E-08	9	1.125		
DMR11:81558701	11	81558701	1200	1	4.67E-07	8	0.666666667		
DMR11:81599701	11	81599701	500	1	2.00E-07	1	0.2		
DMR11:81976601	11	81976601	6500	1	6.99E-07	99	1.523076923	Dgkg	Signaling
DMR11:82153201	11	82153201	800	4	7.49E-11	0	0	Dgkg	Signaling
DMR11:82687001	11	82687001	2900	1	4.25E-08	50	1.724137931	Liph	
DMR11:83418701	11	83418701	1900	1	8.51E-07	17	0.894736842	AABR07034669.1	
DMR11:84518401	11	84518401	400	1	8.53E-08	2	0.5	Yeats2;Cyp2ab1;Parl	Transcription;Protease
DMR11:84759601	11	84759601	300	1	7.79E-07	1	0.333333333	Klhl6	Unknown
DMR11:84909101	11	84909101	900	2	3.30E-09	1	0.111111111	B3gnt5	Metabolism
DMR11:86269201	11	86269201	1300	3	6.79E-08	6	0.461538462	Hira;Mrpl40	Transcription;Translation
DMR11:86472301	11	86472301	400	1	2.06E-07	0	0		
DMR11:86930801	11	86930801	2000	3	6.63E-09	11	0.55	AABR07072264.2;AABR07072264.3	
DMR11:88955801	11	88955801	300	1	6.41E-07	0	0	Pkp2	Cytoskeleton
DMR11:89455501	11	89455501	1000	1	4.70E-07	11	1.1	Prkdc	Signaling
DMR11:90027501	11	90027501	1000	2	4.67E-07	4	0.4		
DMR11:90447901	11	90447901	900	1	6.42E-07	12	1.333333333		
DMR12:75201	12	75201	700	1	9.26E-07	2	0.285714286		
DMR12:115201	12	115201	600	1	9.32E-08	4	0.666666667		
DMR12:138601	12	138601	1500	1	1.54E-08	11	0.733333333		

DMR12:825601	12	825601	400	1	2.37E-07	4	1		
DMR12:1341001	12	1341001	900	1	3.91E-08	9	1		
DMR12:1403101	12	1403101	300	1	3.33E-07	10	3.333333333		
DMR12:1799901	12	1799901	3600	2	3.53E-07	45	1.25	Insr	Receptor
DMR12:1877901	12	1877901	500	1	5.02E-08	0	0		
DMR12:1996101	12	1996101	2700	2	3.28E-12	56	2.074074074	Arhgef18;Pex11g	Signaling
DMR12:2547701	12	2547701	300	1	1.70E-07	5	1.666666667	Snapc2;Tgfbr3l;Map2k7;Lrrc8e	Translation;Signaling;Development
DMR12:2592201	12	2592201	400	1	5.12E-09	4	1	Prr36;Evi5l;AABR07034980.1	
DMR12:4544601	12	4544601	1000	1	6.91E-08	3	0.3	Higd2al1	Apoptosis
DMR12:5628901	12	5628901	1000	1	2.40E-07	9	0.9	Fry	Development
DMR12:6589301	12	6589301	300	1	6.07E-07	7	2.333333333	AABR07035193.1	
DMR12:6713901	12	6713901	700	1	3.11E-07	17	2.428571429	Tex26;Medag	
DMR12:6912401	12	6912401	300	1	8.68E-07	8	2.666666667		
DMR12:7114201	12	7114201	1500	1	8.62E-10	9	0.6	Metazoa_SRP;AABR07035218.1	
DMR12:7352601	12	7352601	1200	3	4.21E-10	12	1	Metazoa_SRP;AABR07035224.1	
DMR12:7684201	12	7684201	1300	1	3.03E-07	11	0.846153846		
DMR12:7847301	12	7847301	2200	1	8.78E-07	44	2	AC123253.1	
DMR12:8849801	12	8849801	3800	1	2.17E-07	75	1.973684211		
DMR12:9633901	12	9633901	1100	1	7.17E-07	4	0.363636364		
DMR12:10111701	12	10111701	800	2	8.89E-09	5	0.625		
DMR12:10124201	12	10124201	800	1	1.46E-07	8	1	AABR07035309.1	
DMR12:11891701	12	11891701	7800	1	2.44E-08	114	1.461538462		
DMR12:12157201	12	12157201	900	2	2.02E-08	7	0.777777778		

DMR12:12627301	12	12627301	5300	1	6.67E-07	92	1.735849057	Ocm2;Rn60_12_0127.3;Ccz1b	Signaling
DMR12:13379801	12	13379801	3100	2	4.09E-08	53	1.709677419	Zfp12;Spdye4	Cell Cycle
DMR12:13497601	12	13497601	3200	1	5.64E-07	38	1.1875	Rnf216	Metabolism
DMR12:14024101	12	14024101	2700	1	7.31E-12	36	1.333333333	Mmd2	Development
DMR12:14447401	12	14447401	300	1	7.83E-07	1	0.333333333	Sdk1	Unknown
DMR12:15478601	12	15478601	4800	1	6.08E-09	57	1.1875		
DMR12:15503201	12	15503201	1700	2	1.24E-07	23	1.352941176		
DMR12:15679301	12	15679301	1000	6	1.67E-14	0	0	Rn60_12_0157.1	
DMR12:16107101	12	16107101	3600	1	5.90E-07	75	2.083333333	Ttyh3;Lfng	Unknown;Signaling
DMR12:16242201	12	16242201	600	2	8.92E-10	1	0.166666667	AC117065.1	
DMR12:16363201	12	16363201	1500	2	2.63E-10	9	0.6	Snx8	Transport
DMR12:16393201	12	16393201	400	1	9.83E-07	6	1.5	Snx8;Nudt1;Mrm2	Transport;Metabolism
DMR12:16500101	12	16500101	800	3	9.62E-12	7	0.875	Mad1l1	
DMR12:16630801	12	16630801	400	1	7.98E-09	2	0.5	Mad1l1	
DMR12:17078701	12	17078701	1500	1	7.59E-07	5	0.333333333		
DMR12:17186801	12	17186801	900	1	4.94E-07	15	1.666666667	Uncx	Transcription
DMR12:17290501	12	17290501	2400	1	1.17E-07	40	1.666666667	LOC498154	Unknown
DMR12:17925201	12	17925201	500	1	1.39E-08	0	0	Fam20c	
DMR12:18339101	12	18339101	300	1	8.96E-07	11	3.666666667		
DMR12:19432901	12	19432901	2400	2	3.28E-07	25	1.041666667	Nxpe5	Unknown
DMR12:22142401	12	22142401	1000	1	3.35E-07	22	2.2	Lrch4;Fbxo24	Unknown
DMR12:22577701	12	22577701	2300	1	1.56E-07	31	1.347826087		
DMR12:23302301	12	23302301	500	2	2.53E-08	1	0.2	Cux1	Development
DMR12:23627901	12	23627901	300	1	4.84E-07	0	0	Rasa4	Signaling
DMR12:23849901	12	23849901	2000	1	4.18E-07	26	1.3	Hspb1;Srrm3	Signaling
DMR12:24642701	12	24642701	1800	3	2.32E-09	26	1.444444444	Vps37d	

DMR12:24769501	12	24769501	1700	1	1.27E-07	17	1	Cldn4;Wbscr27	Cytoskeleton;Development
DMR12:24976501	12	24976501	500	1	6.57E-07	1	0.2	Eln	
DMR12:25674901	12	25674901	900	3	2.47E-10	3	0.333333333		
DMR12:25776001	12	25776001	1500	1	5.51E-07	24	1.6	U6	
DMR12:26226501	12	26226501	500	1	2.50E-08	4	0.8		
DMR12:26281301	12	26281301	500	1	7.88E-07	3	0.6		
DMR12:26362701	12	26362701	500	1	6.14E-08	17	3.4		
DMR12:26673901	12	26673901	1200	1	4.59E-07	4	0.333333333		
DMR12:26793001	12	26793001	400	1	4.96E-07	4	1		
DMR12:26806801	12	26806801	800	2	4.37E-08	7	0.875		
DMR12:27105301	12	27105301	3600	2	1.10E-08	40	1.111111111		
DMR12:27318901	12	27318901	500	2	2.18E-15	2	0.4	AABR07035916.1	
DMR12:27463601	12	27463601	4700	1	6.88E-07	64	1.361702128	AABR07035916.1	
DMR12:27575101	12	27575101	400	1	2.62E-07	9	2.25	AABR07035916.1	
DMR12:28323501	12	28323501	1500	2	6.07E-10	10	0.666666667		
DMR12:28482401	12	28482401	2400	2	1.71E-07	7	0.291666667	Wbscr17;Rn50_12_0305.1	Development
DMR12:28636301	12	28636301	500	1	5.47E-07	4	0.8	Wbscr17	Development
DMR12:28759801	12	28759801	1000	3	5.20E-14	6	0.6	Wbscr17	Development
DMR12:28834001	12	28834001	1700	2	1.48E-09	12	0.705882353	Wbscr17	Development
DMR12:28959901	12	28959901	1700	1	9.22E-07	24	1.411764706	Wbscr17	Development
DMR12:29109901	12	29109901	400	2	4.33E-07	0	0	Wbscr17	Development
DMR12:29656701	12	29656701	1500	3	1.83E-08	12	0.8	Caln1	Signaling
DMR12:31014501	12	31014501	1100	1	2.09E-07	9	0.818181818		
DMR12:31178201	12	31178201	4100	1	8.46E-08	85	2.073170732	Adgrd1	
DMR12:31380701	12	31380701	2100	1	6.65E-08	24	1.142857143		
DMR12:31392501	12	31392501	3600	1	1.02E-08	66	1.833333333		
DMR12:31431401	12	31431401	800	2	2.92E-09	11	1.375		

DMR12:31542801	12	31542801	1400	2	8.35E-08	23	1.642857143	Rimbp2	Unknown
DMR12:31670701	12	31670701	800	1	7.49E-07	15	1.875		
DMR12:31739501	12	31739501	400	2	2.78E-09	1	0.25		
DMR12:32008901	12	32008901	1000	1	1.71E-07	11	1.1	Tmem132d	Unknown
DMR12:32230901	12	32230901	400	1	2.49E-07	1	0.25	Tmem132d	Unknown
DMR12:32941101	12	32941101	600	1	5.34E-07	19	3.166666667	Tmem132c	Unknown
DMR12:33381401	12	33381401	1100	1	8.70E-09	6	0.545454545		
DMR12:33891601	12	33891601	800	1	9.25E-07	3	0.375		
DMR12:34381101	12	34381101	2500	2	1.91E-07	15	0.6		
DMR12:34725501	12	34725501	1400	1	2.10E-07	12	0.857142857		
DMR12:34921601	12	34921601	1800	1	9.25E-07	15	0.833333333		
DMR12:35086201	12	35086201	800	1	2.85E-07	1	0.125		
DMR12:35460001	12	35460001	2300	1	1.51E-07	26	1.130434783		
DMR12:35697601	12	35697601	1300	1	5.70E-07	12	0.923076923		
DMR12:36277601	12	36277601	1500	3	2.72E-13	0	0	Tmem132b	Unknown
DMR12:36480201	12	36480201	1400	2	1.74E-08	11	0.785714286		
DMR12:36656401	12	36656401	500	1	1.15E-07	11	2.2		
DMR12:36660701	12	36660701	2600	3	3.66E-09	30	1.153846154		
DMR12:36733401	12	36733401	3500	1	3.19E-08	57	1.628571429	Scarb1	Receptor
DMR12:38060701	12	38060701	900	1	2.43E-08	10	1.111111111	Hip1r;Ccdc62	Cytoskeleton;Transcription
DMR12:39119501	12	39119501	900	1	7.46E-07	12	1.333333333	Kdm2b	
DMR12:39703901	12	39703901	400	1	1.63E-07	2	0.5	Vps29;Rad9b	Transport;DNA Repair
DMR12:39714801	12	39714801	900	1	7.72E-08	10	1.111111111	Rad9b	DNA Repair
DMR12:39958701	12	39958701	1200	1	6.98E-08	16	1.333333333	Ccdc63;Myl2	Cytoskeleton
DMR12:39969001	12	39969001	2200	3	5.53E-09	41	1.863636364		
DMR12:39988201	12	39988201	600	2	1.54E-09	3	0.5		
DMR12:40094801	12	40094801	3600	2	1.68E-09	99	2.75	Cux2	Development

DMR12:40184001	12	40184001	2100	1	9.33E-07	40	1.904761905	Cux2	Development
DMR12:41033601	12	41033601	2700	1	1.01E-07	22	0.814814815		
DMR12:41063901	12	41063901	2000	2	5.86E-12	19	0.95	Rph3a	Unknown
DMR12:41251801	12	41251801	300	1	3.04E-11	0	0	Oas1b	
DMR12:41641201	12	41641201	1400	4	5.63E-11	16	1.142857143	Sdsl	Metabolism
DMR12:41764501	12	41764501	2000	4	1.52E-08	15	0.75		
DMR12:42159001	12	42159001	2200	1	9.82E-08	23	1.045454545		
DMR12:42192801	12	42192801	2000	1	5.09E-08	14	0.7	AABR07036406.1	
DMR12:42408401	12	42408401	2000	2	9.27E-10	32	1.6		
DMR12:42433301	12	42433301	400	1	9.32E-07	0	0		
DMR12:42830701	12	42830701	1700	2	7.14E-10	30	1.764705882		
DMR12:42861401	12	42861401	500	1	8.58E-11	2	0.4		
DMR12:42940301	12	42940301	1000	2	9.21E-12	14	1.4		
DMR12:43035501	12	43035501	500	1	1.83E-07	2	0.4		
DMR12:43228301	12	43228301	3400	2	4.28E-10	39	1.147058824	AABR07036435.1	
DMR12:43292601	12	43292601	1100	1	4.71E-07	18	1.636363636		
DMR12:43818401	12	43818401	4700	1	5.80E-08	133	2.829787234		
DMR12:43824101	12	43824101	1900	2	7.59E-12	14	0.736842105		
DMR12:43940101	12	43940101	500	1	8.15E-07	9	1.8	RGD1562310;Rnft2	Unknown
DMR12:43954601	12	43954601	1600	1	1.42E-07	22	1.375	Rnft2	
DMR12:44375001	12	44375001	1400	1	7.68E-07	26	1.857142857	Nos1	Metabolism
DMR12:44708401	12	44708401	500	1	3.37E-07	5	1	Ksr2	Signaling
DMR12:44754201	12	44754201	3800	2	1.11E-10	43	1.131578947	Ksr2	Signaling
DMR12:44914401	12	44914401	2000	1	1.26E-07	20	1	Ksr2	Signaling
DMR12:45352001	12	45352001	400	1	7.33E-07	2	0.5		
DMR12:45471201	12	45471201	900	1	6.81E-07	11	1.222222222		
DMR12:45634501	12	45634501	1900	2	1.34E-07	29	1.526315789		
DMR12:45719301	12	45719301	400	2	1.28E-08	1	0.25	Srrm4	Translation
DMR12:46183901	12	46183901	800	1	6.09E-11	2	0.25	Ccdc60	

DMR12:46238301	12	46238301	5100	1	2.78E-08	63	1.235294118	7SK	
DMR12:46270801	12	46270801	2000	2	1.54E-08	18	0.9	Tmem233	
DMR12:47712501	12	47712501	2100	2	7.82E-10	15	0.714285714	Trpv4	Transport
DMR12:47737801	12	47737801	900	1	2.91E-08	12	1.333333333	Trpv4;Fam222a	Transport;Unknown
DMR12:48138501	12	48138501	1100	1	1.93E-08	26	2.363636364	Acacb	Metabolism
DMR12:48166601	12	48166601	1600	2	1.02E-10	19	1.1875	Acacb	Metabolism
DMR12:48499401	12	48499401	800	1	5.12E-09	20	2.5	Coro1c	Cytoskeleton
DMR12:48582401	12	48582401	700	1	8.22E-08	14	2	Selplg	Receptor
DMR12:48621801	12	48621801	1700	3	2.26E-13	35	2.058823529	Iscu;Sart3	Transcription
DMR12:49155401	12	49155401	3100	1	6.51E-07	51	1.64516129		
DMR12:49453901	12	49453901	200	2	9.06E-14	2	1	RGD1306556	
DMR12:49474101	12	49474101	1700	1	5.80E-11	37	2.176470588	RGD1306556	
DMR12:49480801	12	49480801	4700	1	7.23E-07	129	2.744680851	RGD1306556	
DMR12:49799101	12	49799101	400	1	2.58E-07	9	2.25	Myo18b	Cytoskeleton
DMR12:49827301	12	49827301	1800	2	8.57E-08	19	1.055555556	Myo18b	Cytoskeleton
DMR12:50065101	12	50065101	900	2	5.72E-09	7	0.777777778		
DMR12:50071601	12	50071601	2200	1	3.84E-09	31	1.409090909		
DMR12:50121101	12	50121101	3600	3	3.79E-11	33	0.916666667	Sez6l	Receptor
DMR12:50187601	12	50187601	1300	1	5.64E-07	12	0.923076923	Sez6l	Receptor
DMR12:50224401	12	50224401	1000	1	2.16E-11	18	1.8	Sez6l	Receptor
DMR12:50310201	12	50310201	1500	1	5.34E-07	15	1	Hps4;AC123358.1;Srrd;Tfip11	Translation
DMR12:50339801	12	50339801	700	3	3.97E-16	3	0.428571429	Tfip11;Tpst2	Translation;Metabolism
DMR12:50398301	12	50398301	6000	3	3.68E-09	81	1.35	Crybb1;Cryba4	Unknown
DMR12:50485301	12	50485301	1300	1	1.53E-07	5	0.384615385		
DMR12:50565101	12	50565101	3700	5	6.48E-11	47	1.27027027		
DMR12:50627301	12	50627301	600	2	4.35E-07	1	0.166666667	AABR07036626.1	

DMR12:50709301	12	50709301	2300	1	1.86E-07	11	0.47826087		
DMR12:50874501	12	50874501	600	1	4.47E-07	3	0.5		
DMR12:50880701	12	50880701	4100	3	8.92E-11	27	0.658536585		
DMR12:50890501	12	50890501	1200	2	3.79E-09	4	0.333333333		
DMR12:50922301	12	50922301	1900	1	5.89E-07	24	1.263157895		
DMR12:50958801	12	50958801	1800	3	2.28E-10	18	1		
DMR12:51058701	12	51058701	1900	2	2.53E-08	29	1.526315789		
DMR12:51085201	12	51085201	3300	1	1.26E-07	25	0.757575758		
DMR12:51095801	12	51095801	900	1	2.09E-07	15	1.666666667		
DMR12:51106401	12	51106401	1600	2	1.09E-07	26	1.625		
DMR12:51149101	12	51149101	2300	2	5.61E-09	21	0.913043478		
DMR12:51682501	12	51682501	400	1	3.24E-08	3	0.75	Ttc28;AABR07036642.1	Unknown
DMR13:2326501	13	2326501	600	1	2.01E-07	8	1.333333333		
DMR13:2988401	13	2988401	2800	3	2.71E-09	26	0.928571429		
DMR13:3605901	13	3605901	3700	2	2.72E-10	25	0.675675676		
DMR13:4478001	13	4478001	400	1	9.61E-09	0	0		
DMR13:4670101	13	4670101	1000	1	6.34E-07	7	0.7		
DMR13:9513401	13	9513401	4100	2	8.09E-08	41	1		
DMR13:12353401	13	12353401	2500	1	4.50E-10	20	0.8		
DMR13:13372801	13	13372801	1400	1	9.44E-08	8	0.571428571		
DMR13:13750601	13	13750601	400	1	1.03E-07	1	0.25		
DMR13:14380601	13	14380601	400	1	4.13E-07	1	0.25	LOC304725	
DMR13:16372701	13	16372701	1500	1	4.78E-07	16	1.066666667		
DMR13:16466001	13	16466001	800	3	5.95E-10	7	0.875		
DMR13:18905301	13	18905301	300	1	8.51E-07	4	1.333333333		
DMR13:19470001	13	19470001	300	1	7.19E-07	1	0.333333333		
DMR13:19578201	13	19578201	800	1	1.19E-07	2	0.25		
DMR13:19653001	13	19653001	600	2	8.34E-07	3	0.5		

DMR13:19698801	13	19698801	2000	1	7.82E-07	9	0.45		
DMR13:19790101	13	19790101	3100	2	4.11E-09	19	0.612903226		
DMR13:20504801	13	20504801	400	2	9.78E-09	0	0		
DMR13:21134401	13	21134401	1400	1	3.97E-09	7	0.5		
DMR13:21521701	13	21521701	2000	2	5.20E-09	10	0.5		
DMR13:22311301	13	22311301	400	2	1.16E-07	0	0	Cntnap5b	Cytoskeleton
DMR13:25321901	13	25321901	300	1	6.21E-07	0	0		
DMR13:26016601	13	26016601	1200	1	2.41E-08	21	1.75	Zcchc2	Transcription
DMR13:26553001	13	26553001	1800	2	5.18E-08	26	1.444444444		
DMR13:27069901	13	27069901	500	1	2.86E-07	1	0.2	Serpinb13	Protease
DMR13:29881501	13	29881501	1500	3	2.88E-07	12	0.8		
DMR13:30537801	13	30537801	600	2	4.11E-08	4	0.666666667		
DMR13:30576401	13	30576401	4300	3	1.32E-10	36	0.837209302		
DMR13:31750101	13	31750101	500	1	3.40E-09	1	0.2	AC109707.1	
DMR13:32295201	13	32295201	2300	3	2.58E-17	23	1		
DMR13:32993701	13	32993701	4300	1	5.51E-08	37	0.860465116		
DMR13:34018401	13	34018401	200	2	3.21E-07	0	0		
DMR13:34021201	13	34021201	600	1	1.40E-08	7	1.166666667		
DMR13:34127201	13	34127201	600	2	2.48E-08	3	0.5		
DMR13:34622101	13	34622101	900	1	1.95E-07	6	0.666666667	Tfcp2l1;5S_rRNA	
DMR13:35377301	13	35377301	400	1	1.64E-07	11	2.75		
DMR13:37203701	13	37203701	400	2	1.08E-07	0	0		
DMR13:37316501	13	37316501	2700	12	5.62E-11	5	0.185185185		
DMR13:37585101	13	37585101	2800	1	9.76E-10	18	0.642857143		
DMR13:37964301	13	37964301	600	1	1.90E-07	0	0		
DMR13:38428301	13	38428301	700	1	7.17E-07	4	0.571428571		
DMR13:40176501	13	40176501	600	2	8.22E-08	1	0.166666667		
DMR13:41802501	13	41802501	1100	1	4.95E-12	11	1	LOC100362110	
DMR13:42098501	13	42098501	300	1	5.78E-08	0	0		

DMR13:42187601	13	42187601	1300	1	8.55E-07	5	0.384615385		
DMR13:43081101	13	43081101	1500	3	2.65E-09	4	0.266666667		
DMR13:43393101	13	43393101	2900	3	5.80E-11	19	0.655172414	AABR07020835.1	
DMR13:44337201	13	44337201	1900	2	1.29E-07	11	0.578947368	Tmem163	Unknown
DMR13:44438801	13	44438801	2400	1	1.05E-07	30	1.25	Acmsd	
DMR13:44521701	13	44521701	1500	1	4.04E-09	23	1.533333333	Ccnt2;Map3k19	Cell Cycle;Signaling
DMR13:44818501	13	44818501	200	1	9.68E-07	0	0	R3hdm1	Unknown
DMR13:44920401	13	44920401	1300	1	9.26E-08	12	0.923076923	R3hdm1;Mir128-1	Unknown
DMR13:45228401	13	45228401	600	2	1.11E-10	7	1.166666667	SNORA70	
DMR13:45350901	13	45350901	1200	2	1.08E-14	17	1.416666667		
DMR13:46225701	13	46225701	600	1	1.69E-10	4	0.666666667	Thsd7b	Extracellular Matrix
DMR13:46340401	13	46340401	700	1	5.12E-07	2	0.285714286	Thsd7b	Extracellular Matrix
DMR13:48027801	13	48027801	1100	1	6.88E-07	10	0.909090909	Rassf5;Ikbke	Signaling
DMR13:48211301	13	48211301	200	1	6.24E-07	3	1.5	Srgap2	Signaling
DMR13:48477801	13	48477801	900	1	1.18E-07	2	0.222222222	Rab7b	Signaling
DMR13:48910501	13	48910501	3800	2	3.54E-09	44	1.157894737	Cdk18	Cell Cycle
DMR13:50095001	13	50095001	1400	3	1.91E-10	15	1.071428571	Atp2b4	Transport
DMR13:50182201	13	50182201	900	1	4.95E-07	7	0.777777778	Lax1	
DMR13:50751701	13	50751701	5100	2	6.41E-09	71	1.392156863	Optc;Prelp	Extracellular Matrix
DMR13:51243601	13	51243601	400	1	1.28E-07	2	0.5	Cyb5r1;Adipor1	Electron Transport;Receptor
DMR13:51661001	13	51661001	900	5	1.01E-12	5	0.555555556	Ppp1r12b	Signaling
DMR13:52626701	13	52626701	1800	1	5.14E-07	18	1	Tnni1	Cytoskeleton
DMR13:52698001	13	52698001	3600	1	2.11E-07	31	0.861111111	Pkp1	

DMR13:52882701	13	52882701	700	1	2.84E-07	0	0	Ascl5;Cacna1s	Transcription;Transport
DMR13:53668201	13	53668201	400	1	2.89E-07	0	0	AABR07021018.1	
DMR13:53715801	13	53715801	800	1	1.17E-09	9	1.125	AABR07021022.1	
DMR13:54080301	13	54080301	2300	1	5.91E-07	15	0.652173913		
DMR13:54188701	13	54188701	700	2	1.83E-09	1	0.142857143		
DMR13:54211601	13	54211601	600	1	5.53E-07	2	0.333333333		
DMR13:55597001	13	55597001	2400	2	5.78E-08	23	0.958333333		
DMR13:55923201	13	55923201	2500	3	6.36E-19	15	0.6		
DMR13:56000001	13	56000001	1100	1	4.26E-07	7	0.636363636		
DMR13:57063101	13	57063101	2400	1	1.25E-07	12	0.5	Cfh	Immune
DMR13:59223601	13	59223601	500	2	1.58E-08	0	0		
DMR13:59620701	13	59620701	1900	2	7.72E-08	3	0.157894737		
DMR13:59963601	13	59963601	800	2	9.85E-10	6	0.75		
DMR13:60909001	13	60909001	1200	1	9.43E-09	30	2.5		
DMR13:61940801	13	61940801	1500	1	7.74E-09	9	0.6		
DMR13:62244601	13	62244601	1000	1	9.42E-07	1	0.1		
DMR13:62412501	13	62412501	1200	1	1.38E-07	7	0.583333333		
DMR13:62759101	13	62759101	1000	1	7.24E-07	12	1.2		
DMR13:66123201	13	66123201	500	2	9.28E-10	4	0.8		
DMR13:66613201	13	66613201	3300	2	4.58E-07	19	0.575757576	SNORA17	
DMR13:67291901	13	67291901	700	2	8.31E-09	2	0.285714286		
DMR13:67533701	13	67533701	2000	2	3.63E-08	16	0.8	Pdc	Protein Binding
DMR13:68568401	13	68568401	300	1	1.08E-10	0	0		
DMR13:68668201	13	68668201	1900	3	3.47E-15	26	1.368421053		
DMR13:69083101	13	69083101	500	1	6.26E-07	13	2.6	Fam129a	Unknown
DMR13:69636101	13	69636101	1600	1	9.69E-07	20	1.25	AABR07021392.1	
DMR13:69836301	13	69836301	1800	1	7.72E-07	20	1.111111111	Colgalt2	

DMR13:70232701	13	70232701	500	1	3.72E-08	2	0.4	Rn60_13_0703.2;Ncf2	Development
DMR13:70415601	13	70415601	1300	1	4.57E-09	7	0.538461538	Nmnat2	Metabolism
DMR13:70572601	13	70572601	1700	2	3.49E-09	20	1.176470588	Lamc2	Cytoskeleton
DMR13:70755101	13	70755101	1100	1	4.57E-08	20	1.818181818	Lamc1	Extracellular Matrix
DMR13:70796601	13	70796601	1100	1	7.24E-07	7	0.636363636		
DMR13:70854401	13	70854401	300	1	4.47E-07	2	0.666666667	Shcbp1l	
DMR13:70875401	13	70875401	1000	1	1.05E-10	20	2	Shcbp1l;Dhx9	Transcription
DMR13:71009501	13	71009501	300	1	4.63E-09	2	0.666666667		
DMR13:71110001	13	71110001	600	1	8.15E-07	9	1.5	Rgs8	Signaling
DMR13:72386701	13	72386701	400	2	3.35E-08	2	0.5		
DMR13:72523701	13	72523701	1900	3	4.65E-16	9	0.473684211		
DMR13:72554801	13	72554801	1300	2	1.68E-12	7	0.538461538		
DMR13:72588701	13	72588701	2300	2	9.18E-08	27	1.173913043		
DMR13:72953801	13	72953801	2600	1	8.19E-07	28	1.076923077	Xpr1	Receptor
DMR13:73122001	13	73122001	1000	1	1.23E-08	8	0.8		
DMR13:75079201	13	75079201	5900	1	9.99E-07	53	0.898305085		
DMR13:75386501	13	75386501	1000	1	2.18E-07	9	0.9	AABR07021508.1	
DMR13:75693001	13	75693001	2100	1	3.78E-09	20	0.952380952		
DMR13:75962601	13	75962601	3200	1	1.72E-07	30	0.9375	Brinp2	
DMR13:76244001	13	76244001	300	1	1.81E-07	4	1.333333333	Astn1	
DMR13:76662601	13	76662601	1400	1	1.67E-07	19	1.357142857	Pappa2	
DMR13:76700401	13	76700401	1800	1	2.23E-07	9	0.5		
DMR13:77112201	13	77112201	2900	1	2.87E-07	28	0.965517241		
DMR13:77818201	13	77818201	300	2	1.11E-08	1	0.333333333	Rn50_13_0828.1;AABR07021544.1	
DMR13:78484601	13	78484601	1300	1	1.37E-08	10	0.769230769	Rabgap1l	Signaling
DMR13:80102101	13	80102101	400	1	3.00E-07	2	0.5	Dnm3	Cytoskeleton

DMR13:81081601	13	81081601	1400	1	1.39E-07	13	0.928571429		
DMR13:81139601	13	81139601	1300	1	4.53E-08	4	0.307692308	Prrx1	Transcription
DMR13:82098901	13	82098901	300	1	8.43E-12	1	0.333333333	Kifap3	Cytoskeleton
DMR13:82392101	13	82392101	1600	2	1.76E-09	14	0.875	Sell	Extracellular Matrix
DMR13:82422001	13	82422001	3400	1	7.06E-07	38	1.117647059	Selp	Receptor
DMR13:82581801	13	82581801	2800	1	3.32E-07	24	0.857142857	Ccdc181;Blzf1	Transcription
DMR13:83552801	13	83552801	700	3	1.29E-10	2	0.285714286	Gpr161	Receptor
DMR13:83565801	13	83565801	1900	1	6.98E-08	13	0.684210526	Gpr161	Receptor
DMR13:83901501	13	83901501	2600	2	3.57E-10	44	1.692307692	Rcsd1	Unknown
DMR13:84743201	13	84743201	300	1	6.91E-07	1	0.333333333	LOC685351	
DMR13:85040601	13	85040601	300	1	3.58E-08	0	0		
DMR13:85085101	13	85085101	1800	2	4.40E-10	29	1.611111111		
DMR13:85482601	13	85482601	2000	2	1.58E-08	23	1.15	Tmco1	
DMR13:85563601	13	85563601	5000	4	2.18E-14	74	1.48	Tmco1	
DMR13:85613801	13	85613801	1300	1	8.36E-08	26	2	Mgst3;AABR07021691.2	Metabolism
DMR13:86913701	13	86913701	1400	1	6.27E-07	17	1.214285714		
DMR13:88143701	13	88143701	300	1	2.15E-08	0	0		
DMR13:88158401	13	88158401	2600	3	1.63E-07	16	0.615384615		
DMR13:88699101	13	88699101	400	1	2.79E-10	2	0.5	LOC100361087	
DMR13:89374401	13	89374401	2500	1	3.06E-07	18	0.72	Fcgr2b;Fcgr3a	Immune
DMR13:90714901	13	90714901	400	1	2.00E-07	1	0.25	Kcnj9;Kcnj10	Metabolism
DMR13:91097301	13	91097301	4300	1	9.20E-07	50	1.162790698	Crp	
DMR13:92312601	13	92312601	5200	1	4.64E-07	66	1.269230769	Spta1	
DMR13:92953701	13	92953701	900	2	2.15E-08	0	0	Grem2	
DMR13:95727501	13	95727501	800	1	2.55E-08	4	0.5	LOC103692343	
DMR13:96058801	13	96058801	700	1	2.55E-07	0	0	NA	
DMR13:97420101	13	97420101	1600	2	9.21E-25	25	1.5625		

DMR13:97566001	13	97566001	2600	1	2.22E-07	41	1.576923077		
DMR13:98629101	13	98629101	1400	1	2.84E-09	19	1.357142857	Itpkb	Signaling
DMR13:98911401	13	98911401	200	2	1.89E-07	0	0	U6	
DMR13:99165501	13	99165501	1200	1	1.88E-07	8	0.666666667	Lefty2	Signaling
DMR13:99247901	13	99247901	2700	1	1.53E-07	35	1.296296296	Tmem63a	Unknown
DMR13:99284801	13	99284801	500	1	7.52E-08	4	0.8	Ephx1	Metabolism
DMR13:99745401	13	99745401	900	1	5.07E-07	4	0.444444444	Cnih3	Signaling
DMR13:10007320	13	1E+08	200	1	1.08E-07	1	0.5	LOC690288;AABR07021946.1	
DMR13:10016620	13	1E+08	500	2	4.74E-08	10	2	Eif3el1	
DMR13:10108430	13	1.01E+08	4200	1	7.22E-07	69	1.642857143		
DMR13:10139540	13	1.01E+08	500	2	9.98E-09	1	0.2	Tlr5	Receptor
DMR13:10170310	13	1.02E+08	2800	1	1.01E-07	29	1.035714286	Brox;Aida	
DMR13:10230610	13	1.02E+08	900	4	9.77E-09	9	1		
DMR13:10231430	13	1.02E+08	3200	2	3.01E-09	29	0.90625		
DMR13:10267480	13	1.03E+08	1800	1	6.81E-07	18	1		
DMR13:10311690	13	1.03E+08	1000	1	2.97E-07	5	0.5		
DMR13:10422770	13	1.04E+08	2200	1	5.61E-08	13	0.590909091		
DMR13:10448940	13	1.04E+08	1200	1	6.67E-07	12	1		
DMR13:10465130	13	1.05E+08	1000	1	5.33E-09	8	0.8	AABR07022055.1	
DMR13:10525950	13	1.05E+08	900	1	3.15E-08	8	0.888888889		
DMR13:10593420	13	1.06E+08	1800	5	7.66E-14	15	0.833333333		
DMR13:10602890	13	1.06E+08	3900	2	2.34E-09	40	1.025641026		
DMR13:10607380	13	1.06E+08	300	1	6.02E-07	2	0.666666667		
DMR13:10642710	13	1.06E+08	2200	2	9.75E-10	22	1		
DMR13:10664460	13	1.07E+08	300	1	1.97E-08	5	1.666666667	Esrrg	Receptor
DMR13:10680340	13	1.07E+08	300	1	4.70E-07	0	0	Ush2a	Extracellular Matrix

DMR13:10733380	13	1.07E+08	2300	1	1.49E-09	57	2.47826087	Ush2a	Extracellular Matrix
DMR13:10769600	13	1.08E+08	3200	1	6.37E-07	32	1	Kcnk2	Transport
DMR13:10773600	13	1.08E+08	2600	1	4.91E-07	37	1.423076923	Kcnk2	Transport
DMR13:10795030	13	1.08E+08	2000	3	4.28E-10	15	0.75		
DMR13:10827080	13	1.08E+08	200	1	1.67E-07	2	1	AABR07022108.1	
DMR13:10859970	13	1.09E+08	400	1	1.11E-07	0	0	AABR07022113.1	
DMR13:10940900	13	1.09E+08	2400	3	3.01E-11	26	1.083333333	Rps6kc1	Signaling
DMR13:10941310	13	1.09E+08	1400	1	8.14E-07	19	1.357142857	Rps6kc1	Signaling
DMR13:11047090	13	1.1E+08	1500	1	3.65E-07	6	0.4	Lpgat1;AABR07022162.1	Metabolism
DMR13:11136970	13	1.11E+08	800	1	3.79E-08	1	0.125	Hhat	Metabolism
DMR13:11142670	13	1.11E+08	500	2	3.92E-09	1	0.2	Hhat	Metabolism
DMR13:11214020	13	1.12E+08	1800	2	1.11E-09	23	1.277777778		
DMR13:11227450	13	1.12E+08	400	2	7.28E-08	2	0.5		
DMR13:11236300	13	1.12E+08	1300	1	1.27E-07	3	0.230769231		
DMR13:11243160	13	1.12E+08	400	1	4.99E-08	0	0		
DMR13:11289320	13	1.13E+08	400	1	5.20E-07	1	0.25		
DMR13:11303670	13	1.13E+08	400	1	8.08E-08	5	1.25		
DMR13:11309570	13	1.13E+08	1400	2	7.43E-07	10	0.714285714		
DMR13:11371880	13	1.14E+08	200	1	4.48E-08	0	0	Cd34	Immune
DMR13:11376700	13	1.14E+08	1300	1	4.23E-07	6	0.461538462	AC118802.1	
DMR13:11400020	13	1.14E+08	200	1	5.54E-07	1	0.5		
DMR14:448501	14	448501	2000	1	1.51E-08	24	1.2		
DMR14:483701	14	483701	1700	1	8.68E-08	7	0.411764706		
DMR14:860201	14	860201	300	1	8.69E-07	4	1.333333333		
DMR14:2828001	14	2828001	1500	1	5.91E-10	14	0.933333333	Fam69a	
DMR14:3415801	14	3415801	2400	3	1.03E-09	17	0.708333333	Brdt	Epigenetic
DMR14:3430501	14	3430501	600	2	9.40E-09	5	0.833333333	Brdt	Epigenetic

DMR14:3530801	14	3530801	1900	1	2.92E-08	16	0.842105263	Tgfbr3	Receptor
DMR14:3549401	14	3549401	700	1	4.57E-08	5	0.714285714	Tgfbr3	Receptor
DMR14:3642801	14	3642801	2900	2	6.73E-09	47	1.620689655	Tgfbr3	Receptor
DMR14:3730901	14	3730901	2000	2	1.80E-09	18	0.9		
DMR14:4217901	14	4217901	200	1	2.77E-07	2	1		
DMR14:4391601	14	4391601	300	1	6.67E-07	1	0.333333333		
DMR14:4702601	14	4702601	300	1	1.26E-09	2	0.666666667		
DMR14:5813801	14	5813801	1100	1	7.19E-07	9	0.818181818		
DMR14:5988601	14	5988601	2500	1	6.31E-07	36	1.44		
DMR14:6076401	14	6076401	1200	1	6.50E-07	7	0.583333333	Abcg3l3	
DMR14:6811301	14	6811301	2100	1	7.89E-07	18	0.857142857	Ibsp	Signaling
DMR14:8522801	14	8522801	2400	1	1.38E-08	26	1.083333333	Arhgap24	Unknown
DMR14:8559701	14	8559701	900	2	4.84E-10	8	0.888888889	Arhgap24	Unknown
DMR14:9474401	14	9474401	400	2	1.31E-08	3	0.75		
DMR14:9681501	14	9681501	300	1	7.86E-07	0	0		
DMR14:10297801	14	10297801	1200	3	5.45E-09	18	1.5	AABR07014342.1	
DMR14:10367701	14	10367701	1800	1	4.79E-08	29	1.611111111	Gpat3	
DMR14:11332701	14	11332701	1900	1	7.33E-07	27	1.421052632	U6	
DMR14:11840701	14	11840701	300	1	3.56E-09	0	0		
DMR14:11929701	14	11929701	800	1	7.05E-08	13	1.625		
DMR14:12105501	14	12105501	600	1	1.70E-08	0	0		
DMR14:12209001	14	12209001	1000	1	9.28E-10	9	0.9	Prkg2	Signaling
DMR14:12450301	14	12450301	600	1	2.01E-10	15	2.5		
DMR14:12548901	14	12548901	2300	2	2.61E-09	30	1.304347826		
DMR14:13143401	14	13143401	1300	1	4.67E-07	20	1.538461538		
DMR14:13447201	14	13447201	700	1	1.33E-09	1	0.142857143		
DMR14:13502801	14	13502801	800	1	7.17E-07	14	1.75		
DMR14:13660601	14	13660601	1000	1	4.70E-07	14	1.4	U6	
DMR14:14057901	14	14057901	2600	1	9.24E-07	19	0.730769231		

DMR14:14081501	14	14081501	800	1	2.47E-10	5	0.625		
DMR14:14304901	14	14304901	1200	1	1.34E-08	13	1.083333333		
DMR14:16610301	14	16610301	3700	1	3.76E-07	46	1.243243243	Shroom3	Cytoskeleton
DMR14:16879301	14	16879301	1200	1	1.70E-08	20	1.666666667	Shroom3	Cytoskeleton
DMR14:17068301	14	17068301	200	1	4.77E-08	1	0.5	Scarb2	Receptor
DMR14:17298901	14	17298901	2400	1	1.42E-07	41	1.708333333	Naaa;Ppef2	Metabolism;Signaling
DMR14:17419801	14	17419801	2900	4	3.70E-09	16	0.551724138	Uso1	Golgi
DMR14:17503901	14	17503901	800	3	1.30E-16	20	2.5	G3bp2	Signaling
DMR14:18059201	14	18059201	800	1	8.51E-07	3	0.375	Parm1	Apoptosis
DMR14:18535101	14	18535101	2900	1	1.59E-08	27	0.931034483	Areg	Growth Factors & Cytokines
DMR14:18948601	14	18948601	500	3	6.37E-11	0	0	SNORA25	
DMR14:18991301	14	18991301	2000	2	4.26E-09	31	1.55	Rassf6	Signaling
DMR14:19220601	14	19220601	900	1	4.43E-08	8	0.888888889		
DMR14:20013501	14	20013501	500	1	3.18E-07	2	0.4	Adamts3	Protease
DMR14:20262701	14	20262701	1800	1	1.06E-07	15	0.833333333	Gc	Binding Protein
DMR14:20826901	14	20826901	1700	1	5.58E-15	25	1.470588235	Slc4a4	Transport
DMR14:21886201	14	21886201	1800	1	1.03E-07	20	1.111111111	Dzip1-ps1;Prr27	
DMR14:22571001	14	22571001	2300	1	9.67E-09	21	0.913043478	Ugt2a1;Ugt2b35	Metabolism
DMR14:23337501	14	23337501	4200	1	2.09E-07	62	1.476190476	Tmprss11d	Protease
DMR14:23738701	14	23738701	600	1	5.51E-09	4	0.666666667		
DMR14:26847201	14	26847201	1300	1	9.28E-08	10	0.769230769		
DMR14:26870701	14	26870701	500	1	8.62E-09	4	0.8		
DMR14:26960001	14	26960001	700	1	1.82E-08	0	0		
DMR14:27121101	14	27121101	800	1	1.08E-09	3	0.375		
DMR14:27332901	14	27332901	400	1	1.88E-09	0	0	U6	
DMR14:28008501	14	28008501	800	3	6.51E-10	4	0.5		
DMR14:28803001	14	28803001	1200	1	2.95E-07	12	1	Adgrl3	

DMR14:28810401	14	28810401	1300	2	2.16E-10	19	1.461538462	Adgrl3	
DMR14:29587701	14	29587701	2700	1	3.45E-07	17	0.62962963		
DMR14:33656001	14	33656001	800	1	4.34E-07	10	1.25	Aasdh;RGD1311575	Metabolism
DMR14:34312101	14	34312101	1200	2	1.12E-11	9	0.75		
DMR14:35214101	14	35214101	200	1	4.07E-08	1	0.5		
DMR14:35848701	14	35848701	2100	1	5.91E-10	12	0.571428571		
DMR14:36043901	14	36043901	2000	1	1.22E-09	38	1.9	Lnx1	Cytoskeleton
DMR14:36589601	14	36589601	300	1	1.87E-08	0	0	AC114452.1	
DMR14:36669101	14	36669101	600	1	3.05E-08	3	0.5	SNORA26;SNORA27	
DMR14:36997801	14	36997801	3000	1	2.01E-07	36	1.2	AABR07014897.2	
DMR14:37346701	14	37346701	1600	1	4.33E-08	13	0.8125	Cwh43	Development
DMR14:37852501	14	37852501	3000	1	3.86E-08	38	1.266666667	Slain2	
DMR14:38094001	14	38094001	800	3	7.81E-10	3	0.375	Txk;Nipal1	Signaling
DMR14:38224301	14	38224301	1300	2	6.47E-08	7	0.538461538	Nfxl1	Transcription
DMR14:39600201	14	39600201	3400	1	3.70E-07	39	1.147058824		
DMR14:41326901	14	41326901	1400	1	5.00E-07	6	0.428571429		
DMR14:41725901	14	41725901	900	2	5.78E-09	7	0.777777778	Grxcr1	Metabolism
DMR14:43086901	14	43086901	1200	2	3.60E-10	7	0.583333333		
DMR14:43717901	14	43717901	1400	3	4.50E-13	17	1.214285714		
DMR14:43999901	14	43999901	1800	1	5.83E-08	18	1	Rhoh	Signaling
DMR14:44174901	14	44174901	2100	1	1.95E-09	25	1.19047619	Pds5a	Transcription
DMR14:44487601	14	44487601	500	1	1.88E-07	1	0.2	Ugdh	Metabolism
DMR14:45094501	14	45094501	1500	4	1.89E-08	16	1.066666667		
DMR14:45333501	14	45333501	400	1	3.41E-08	1	0.25	Mir328b	
DMR14:46429501	14	46429501	1300	1	5.54E-07	1	0.076923077		
DMR14:46601701	14	46601701	13300	1	9.44E-08	212	1.593984962	LOC257642	
DMR14:46627801	14	46627801	12100	5	3.21E-11	839	6.933884298	pRNA;Rn5-8s	
DMR14:46678401	14	46678401	5200	1	1.31E-07	560	10.76923077	pRNA;5_8S_rRNA	
DMR14:48293401	14	48293401	400	1	2.77E-08	0	0		

DMR14:48475901	14	48475901	300	1	3.34E-11	1	0.333333333		
DMR14:49505601	14	49505601	300	1	6.67E-07	0	0		
DMR14:49558501	14	49558501	500	1	1.35E-07	7	1.4		
DMR14:49914201	14	49914201	1400	1	8.16E-09	14	1		
DMR14:50271301	14	50271301	2500	1	8.43E-07	20	0.8		
DMR14:50392001	14	50392001	1200	1	8.69E-07	4	0.333333333		
DMR14:52553601	14	52553601	400	1	2.52E-07	1	0.25		
DMR14:52933301	14	52933301	400	3	2.45E-10	0	0		
DMR14:53057501	14	53057501	3800	1	1.76E-07	13	0.342105263	AABR07015358.1	
DMR14:53843801	14	53843801	1100	1	9.75E-07	9	0.818181818		
DMR14:54565601	14	54565601	900	1	1.91E-08	2	0.222222222		
DMR14:54732101	14	54732101	1000	1	1.37E-09	71	7.1		
DMR14:54881001	14	54881001	500	1	2.11E-07	5	1		
DMR14:54980201	14	54980201	300	1	8.92E-07	0	0		
DMR14:56095101	14	56095101	300	1	4.43E-07	1	0.333333333		
DMR14:56803901	14	56803901	1000	1	2.47E-07	7	0.7		
DMR14:56871701	14	56871701	4100	1	6.22E-08	35	0.853658537		
DMR14:58110501	14	58110501	1100	1	1.35E-07	15	1.363636364		
DMR14:58730701	14	58730701	700	2	2.11E-10	8	1.142857143		
DMR14:59083301	14	59083301	2000	1	1.11E-07	27	1.35		
DMR14:59901201	14	59901201	1500	4	1.09E-18	5	0.333333333		
DMR14:60964301	14	60964301	500	2	2.25E-09	5	1	Sod3	Metabolism
DMR14:61049301	14	61049301	1500	1	5.67E-08	3	0.2		
DMR14:61595801	14	61595801	800	1	4.52E-07	6	0.75		
DMR14:61682001	14	61682001	1500	2	1.80E-10	18	1.2		
DMR14:62780401	14	62780401	700	2	3.54E-08	5	0.714285714		
DMR14:62983301	14	62983301	800	1	8.08E-07	8	1		
DMR14:63246301	14	63246301	400	2	8.56E-09	0	0		
DMR14:63262301	14	63262301	400	1	1.77E-08	0	0		

DMR14:63503501	14	63503501	700	1	2.17E-07	0	0		
DMR14:63902401	14	63902401	800	3	8.74E-12	2	0.25		
DMR14:64563701	14	64563701	300	1	1.33E-07	0	0		
DMR14:65149001	14	65149001	700	2	3.45E-09	1	0.142857143	AABR07015596.1	
DMR14:66666201	14	66666201	300	1	9.74E-08	0	0	Kcnip4	Metabolism
DMR14:67680601	14	67680601	1800	1	3.05E-09	21	1.166666667		
DMR14:67689501	14	67689501	1800	1	4.27E-07	42	2.333333333		
DMR14:67741401	14	67741401	3400	2	7.52E-07	29	0.852941176		
DMR14:67960501	14	67960501	500	1	1.77E-08	3	0.6		
DMR14:69494701	14	69494701	300	1	3.60E-09	1	0.333333333		
DMR14:69513901	14	69513901	800	1	2.75E-07	2	0.25	AABR07015674.1	
DMR14:69580101	14	69580101	800	2	4.64E-10	10	1.25		
DMR14:70086701	14	70086701	400	2	1.18E-07	0	0	Fam184b;Med28	
DMR14:70132901	14	70132901	1300	1	1.14E-08	11	0.846153846		
DMR14:70843201	14	70843201	600	1	1.28E-07	0	0	Ldb2	Transcription
DMR14:71058201	14	71058201	3300	2	6.96E-08	29	0.878787879	Ldb2	Transcription
DMR14:71583501	14	71583501	3300	1	9.78E-07	23	0.696969697	Prom1	Transport
DMR14:75613301	14	75613301	2200	2	7.35E-08	8	0.363636364		
DMR14:76061501	14	76061501	1400	2	3.34E-07	8	0.571428571		
DMR14:76588901	14	76588901	2100	2	1.40E-08	11	0.523809524		
DMR14:77578401	14	77578401	1700	1	2.33E-07	10	0.588235294		
DMR14:77597001	14	77597001	300	1	2.29E-08	2	0.666666667		
DMR14:77659901	14	77659901	2000	1	4.57E-08	27	1.35	AABR07015800.1;AABR07015800.2	
DMR14:77676801	14	77676801	2700	4	5.56E-11	18	0.666666667	AABR07015800.1	
DMR14:78228601	14	78228601	300	1	3.84E-09	2	0.666666667	Evc	Development
DMR14:78872001	14	78872001	700	3	1.98E-08	2	0.285714286	Ppp2r2c	Signaling
DMR14:80039301	14	80039301	1400	1	3.36E-07	4	0.285714286	Ablim2	Cytoskeleton
DMR14:81456801	14	81456801	1200	2	2.19E-09	8	0.666666667	Sh3bp2;U1	Translation

DMR14:81764001	14	81764001	2100	2	9.90E-10	25	1.19047619	Zfyve28	Transcription
DMR14:82500101	14	82500101	1800	1	9.38E-07	25	1.388888889		
DMR14:83652201	14	83652201	1900	1	2.82E-07	17	0.894736842	Rnf185	Transcription
DMR14:83720901	14	83720901	300	1	6.26E-07	4	1.333333333	Pla2g3;Inpp5j	Metabolism;Signaling
DMR14:84558101	14	84558101	800	3	9.19E-08	5	0.625		
DMR14:84842801	14	84842801	500	1	6.26E-08	1	0.2	U6	
DMR14:84849301	14	84849301	1200	2	1.12E-08	7	0.583333333	U6	
DMR14:85258001	14	85258001	1000	1	5.69E-08	10	1	Ap1b1	Metabolism
DMR14:85813801	14	85813801	400	1	6.31E-07	2	0.5	Ankrd36	
DMR14:85917801	14	85917801	4900	1	4.50E-07	51	1.040816327		
DMR14:86360801	14	86360801	1300	2	2.86E-07	2	0.153846154	Npc1l1;U4	Receptor
DMR14:86969801	14	86969801	500	2	5.70E-15	2	0.4	U4;AABR07015941.2	
DMR14:87495101	14	87495101	300	1	2.33E-10	1	0.333333333		
DMR14:88239701	14	88239701	900	1	1.37E-07	9	1		
DMR14:89079201	14	89079201	300	1	5.56E-10	0	0	Pkd1l1	Signaling
DMR14:89138301	14	89138301	400	1	5.70E-09	3	0.75	Pkd1l1	Signaling
DMR14:89489701	14	89489701	1200	2	7.54E-12	8	0.666666667	Abca13	Transport
DMR14:91779901	14	91779901	900	3	8.49E-10	0	0	Ikzf1	Transcription
DMR14:91835601	14	91835601	3600	3	9.09E-09	53	1.472222222	Ikzf1	Transcription
DMR14:92388901	14	92388901	800	2	6.53E-09	2	0.25	Cobl	Development
DMR14:93473201	14	93473201	3300	1	8.13E-07	18	0.545454545		
DMR14:94612601	14	94612601	1400	1	2.52E-07	15	1.071428571		
DMR14:95106601	14	95106601	800	2	3.61E-10	4	0.5		
DMR14:95111501	14	95111501	500	1	5.65E-09	2	0.4		
DMR14:95245801	14	95245801	1200	1	7.61E-08	27	2.25		
DMR14:95427001	14	95427001	1600	1	6.23E-07	13	0.8125		
DMR14:96331701	14	96331701	1000	1	3.02E-07	10	1		

DMR14:96573401	14	96573401	500	2	5.09E-08	1	0.2		
DMR14:96936401	14	96936401	800	2	4.52E-08	4	0.5		
DMR14:96958701	14	96958701	900	1	4.88E-07	13	1.444444444		
DMR14:97246401	14	97246401	800	1	5.87E-07	9	1.125		
DMR14:97276601	14	97276601	100	1	4.20E-34	0	0		
DMR14:98515401	14	98515401	700	2	3.11E-11	3	0.428571429		
DMR14:98692601	14	98692601	300	2	5.41E-07	2	0.666666667		
DMR14:99832801	14	99832801	500	2	1.93E-10	1	0.2		
DMR14:10025200	14	1E+08	1200	3	4.57E-11	3	0.25	Cnrip1;Rn50_14_1003.1	
DMR14:10141240	14	1.01E+08	4400	1	2.08E-08	48	1.090909091		
DMR14:10170710	14	1.02E+08	300	1	9.69E-07	0	0		
DMR14:10173140	14	1.02E+08	800	1	3.95E-08	16	2		
DMR14:10198760	14	1.02E+08	6900	18	4.63E-11	112	1.623188406		
DMR14:10234170	14	1.02E+08	400	2	1.94E-08	5	1.25		
DMR14:10276590	14	1.03E+08	800	1	7.65E-08	4	0.5		
DMR14:10335210	14	1.03E+08	600	2	1.56E-09	0	0	AABR07016556.1	
DMR14:10368160	14	1.04E+08	3200	1	3.49E-07	21	0.65625		
DMR14:10572310	14	1.06E+08	300	1	1.12E-07	1	0.333333333		
DMR14:10684770	14	1.07E+08	300	1	2.12E-07	0	0		
DMR14:10696510	14	1.07E+08	2800	1	7.41E-07	33	1.178571429	Ehbp1	Unknown
DMR14:10810840	14	1.08E+08	300	2	1.17E-09	0	0		
DMR14:10817150	14	1.08E+08	2100	3	1.06E-11	30	1.428571429	Usp34	Protease
DMR14:10849530	14	1.08E+08	1200	1	1.92E-07	15	1.25	Rel	Transcription
DMR14:10857560	14	1.09E+08	1600	2	9.42E-08	13	0.8125		
DMR14:10875300	14	1.09E+08	7200	1	7.31E-07	113	1.569444444		
DMR14:10892150	14	1.09E+08	2500	1	8.69E-07	33	1.32	Bcl11a;AABR07016640.1	Transcription
DMR14:10956700	14	1.1E+08	2200	1	1.68E-07	18	0.818181818	LOC108348154	

DMR14:11008020	14	1.1E+08	800	1	1.11E-07	5	0.625		
DMR14:11086540	14	1.11E+08	700	1	3.29E-09	4	0.571428571	Vrk2	Signaling
DMR14:11103480	14	1.11E+08	1800	1	7.50E-07	10	0.555555556		
DMR14:11296020	14	1.13E+08	900	1	7.56E-07	8	0.888888889	AABR07016731.2	
DMR14:11314450	14	1.13E+08	300	1	4.06E-08	2	0.666666667	AABR07016731.1	
DMR14:11376540	14	1.14E+08	300	1	7.46E-07	1	0.333333333		
DMR14:11402850	14	1.14E+08	200	2	3.62E-09	0	0	AABR07016779.1	
DMR14:11476810	14	1.15E+08	1200	1	7.21E-07	10	0.833333333	U6	
DMR14:11543170	14	1.15E+08	2000	1	3.75E-07	18	0.9	Asb3	
DMR15:400501	15	400501	1300	2	1.66E-07	11	0.846153846	Kcnma1	Metabolism
DMR15:535201	15	535201	500	1	2.97E-09	1	0.2	Kcnma1	Metabolism
DMR15:1881801	15	1881801	500	2	8.03E-10	3	0.6		
DMR15:2093901	15	2093901	2200	2	8.22E-09	39	1.772727273		
DMR15:2193401	15	2193401	300	1	9.90E-07	0	0		
DMR15:2742301	15	2742301	300	1	3.15E-07	0	0	LOC108348179;Dusp13	Signaling
DMR15:3067501	15	3067501	1100	1	2.60E-07	8	0.727272727	Adk	Signaling
DMR15:3106101	15	3106101	1300	1	1.82E-07	25	1.923076923	Adk	Signaling
DMR15:3530701	15	3530701	1200	1	3.72E-09	7	0.583333333	Vcl	Extracellular Matrix
DMR15:4130801	15	4130801	2100	1	3.60E-07	22	1.047619048	Usp54;Rn50_15_0083.1	
DMR15:4977301	15	4977301	5700	1	3.81E-07	74	1.298245614		
DMR15:5531701	15	5531701	1200	1	4.76E-07	11	0.916666667	AABR07016976.1	
DMR15:5811901	15	5811901	3900	1	2.18E-07	23	0.58974359	Cd99l2;LOC102550314	Extracellular Matrix
DMR15:5904701	15	5904701	1900	1	6.70E-07	10	0.526315789	Cd99l2;LOC102546376	Extracellular Matrix;EST
DMR15:6614101	15	6614101	2200	2	6.73E-10	14	0.636363636		

DMR15:6734001	15	6734001	100	1	6.92E-17	0	0		
DMR15:8153201	15	8153201	2200	3	9.02E-09	27	1.227272727	Ube2e1	Metabolism
DMR15:8660701	15	8660701	400	1	6.17E-09	0	0		
DMR15:8687301	15	8687301	200	1	4.31E-07	2	1		
DMR15:8730301	15	8730301	600	2	1.18E-07	46	7.666666667	Nr1d2	Receptor
DMR15:8966601	15	8966601	1600	1	1.18E-07	19	1.1875	Thrb	
DMR15:9350801	15	9350801	1200	1	7.91E-07	11	0.916666667		
DMR15:9882401	15	9882401	2500	2	1.09E-08	14	0.56		
DMR15:10465701	15	10465701	800	1	2.80E-11	0	0	Ngly1;Oxsm	Metabolism
DMR15:10665801	15	10665801	600	2	3.23E-07	5	0.833333333		
DMR15:11495701	15	11495701	1400	1	5.34E-07	12	0.857142857		
DMR15:12201201	15	12201201	300	1	8.83E-08	1	0.333333333		
DMR15:12487001	15	12487001	2400	2	7.46E-07	35	1.458333333	Atxn7	Development
DMR15:12607601	15	12607601	1000	2	7.89E-10	2	0.2	LOC102550375	
DMR15:12614001	15	12614001	1000	1	6.54E-07	6	0.6		
DMR15:13281101	15	13281101	400	1	8.78E-07	0	0		
DMR15:13431401	15	13431401	1000	2	4.46E-08	4	0.4		
DMR15:14110601	15	14110601	1700	1	4.25E-07	9	0.529411765		
DMR15:14849201	15	14849201	600	1	4.56E-07	2	0.333333333		
DMR15:15245401	15	15245401	1300	1	1.99E-07	6	0.461538462		
DMR15:15263301	15	15263301	400	1	9.06E-07	3	0.75		
DMR15:16337601	15	16337601	500	1	8.16E-07	1	0.2		
DMR15:16954201	15	16954201	2000	1	1.09E-07	11	0.55		
DMR15:16960901	15	16960901	1300	1	3.16E-10	7	0.538461538		
DMR15:18083301	15	18083301	500	1	9.31E-08	3	0.6	Ptgdr	Receptor
DMR15:18339701	15	18339701	2200	1	2.12E-08	14	0.636363636	Fam3d	Unknown
DMR15:18472901	15	18472901	700	1	2.43E-07	14	2	Acox2	Metabolism
DMR15:19384201	15	19384201	1300	2	6.94E-09	8	0.615384615		
DMR15:20500801	15	20500801	2100	2	1.29E-08	17	0.80952381		

DMR15:20635301	15	20635301	600	1	3.32E-07	7	1.166666667		
DMR15:21076401	15	21076401	2900	1	2.28E-07	30	1.034482759		
DMR15:21175401	15	21175401	1900	1	7.35E-08	25	1.315789474		
DMR15:21807401	15	21807401	400	1	7.68E-08	0	0		
DMR15:22146101	15	22146101	500	1	3.32E-07	2	0.4		
DMR15:22409901	15	22409901	1000	1	7.46E-07	6	0.6		
DMR15:22793901	15	22793901	1400	1	9.92E-08	20	1.428571429		
DMR15:23224601	15	23224601	900	1	1.50E-31	3	0.333333333		
DMR15:23230801	15	23230801	300	1	2.68E-08	0	0		
DMR15:23427401	15	23427401	3000	1	1.24E-07	36	1.2		
DMR15:23870801	15	23870801	1700	1	9.43E-07	19	1.117647059	Samd4a	Signaling
DMR15:24127701	15	24127701	3200	1	8.57E-07	51	1.59375		
DMR15:25855501	15	25855501	2400	1	3.23E-09	37	1.541666667		
DMR15:26318801	15	26318801	600	1	7.96E-07	4	0.666666667		
DMR15:26838801	15	26838801	1300	1	1.84E-07	6	0.461538462		
DMR15:27753701	15	27753701	800	1	2.77E-07	9	1.125	Parp2;Tep1	Metabolism;Transcription
DMR15:28093301	15	28093301	1600	3	4.28E-09	15	0.9375	Eddm3b;Ang2	Signaling;Transcription
DMR15:28558501	15	28558501	900	1	2.75E-07	8	0.888888889	Rpgrip1	Signaling
DMR15:29268801	15	29268801	800	1	2.79E-07	1	0.125		
DMR15:29287701	15	29287701	1300	1	1.45E-07	6	0.461538462	AABR07017617.2	
DMR15:29782201	15	29782201	700	1	9.78E-08	1	0.142857143	AABR07017658.1	
DMR15:30501001	15	30501001	300	2	1.20E-07	0	0		
DMR15:30546301	15	30546301	2400	1	9.92E-07	48	2	AABR07017745.3;AABR07017745.4	
DMR15:30814601	15	30814601	1200	1	4.40E-08	5	0.416666667	AABR07017768.1;AABR07017768.7	
DMR15:31199601	15	31199601	1200	1	7.70E-08	2	0.166666667	AABR07017804.1	

DMR15:31235601	15	31235601	3000	1	7.47E-07	13	0.433333333	LOC100360891	
DMR15:31403101	15	31403101	300	1	9.30E-09	1	0.333333333	AABR07017824.1	
DMR15:31448801	15	31448801	1700	1	4.79E-08	18	1.058823529	AABR07017825.6;AABR07017825.1	
DMR15:31581801	15	31581801	1300	1	2.74E-07	13	1	AABR07017830.1	
DMR15:31595301	15	31595301	1900	2	5.56E-09	18	0.947368421		
DMR15:31793701	15	31793701	1600	1	2.86E-07	8	0.5		
DMR15:31976901	15	31976901	2400	1	4.95E-07	11	0.458333333	AABR07017868.2	
DMR15:32119701	15	32119701	300	2	5.40E-08	1	0.333333333		
DMR15:33608301	15	33608301	1100	1	4.76E-07	9	0.818181818	Il25;Cmtm5;Myh6;AC115371.1;Mir3546	Signaling;Cytoskeleton
DMR15:33672101	15	33672101	1800	3	1.01E-12	21	1.166666667	Ngdn	
DMR15:33860901	15	33860901	1300	1	7.24E-07	11	0.846153846		
DMR15:34123701	15	34123701	900	1	5.64E-08	1	0.111111111		
DMR15:34139801	15	34139801	300	1	1.12E-07	8	2.666666667		
DMR15:34374701	15	34374701	2200	1	3.08E-09	28	1.272727273	Tgm1	Metabolism
DMR15:34432901	15	34432901	1400	2	2.55E-09	3	0.214285714	Dhrs1;Nop9;Cideb	Metabolism;Unknown
DMR15:34744701	15	34744701	300	1	2.90E-07	2	0.666666667	Mcpt4	Proteolysis
DMR15:36341501	15	36341501	400	1	8.16E-07	2	0.5	LOC102553861	
DMR15:36544301	15	36544301	1200	2	3.74E-07	6	0.5		
DMR15:36824801	15	36824801	1400	1	1.53E-08	17	1.214285714	Parp4	
DMR15:38158301	15	38158301	700	1	9.54E-08	9	1.285714286	Zdhhc20	Unknown
DMR15:38790401	15	38790401	700	4	1.19E-11	1	0.142857143		
DMR15:39088901	15	39088901	400	1	4.75E-09	3	0.75		
DMR15:39118101	15	39118101	1800	2	7.68E-12	19	1.055555556		
DMR15:40208101	15	40208101	5500	9	1.50E-10	56	1.018181818	Atp8a2	Transport
DMR15:41480101	15	41480101	4900	1	4.95E-07	27	0.551020408	Sacs	
DMR15:41610001	15	41610001	600	1	4.04E-07	0	0	AC111687.1	

DMR15:42439501	15	42439501	1000	2	6.45E-10	10	1		
DMR15:42701201	15	42701201	100	1	5.96E-08	1	1	Gulo;Adam2	Protease
DMR15:42930701	15	42930701	3400	1	8.21E-08	37	1.088235294	Ptk2b	Signaling
DMR15:43082901	15	43082901	700	1	4.21E-08	8	1.142857143		
DMR15:43878801	15	43878801	300	1	2.72E-08	0	0		
DMR15:43940301	15	43940301	1300	1	6.03E-07	11	0.846153846	Ebf2	Immune
DMR15:44339901	15	44339901	2000	1	4.08E-08	19	0.95		
DMR15:44867301	15	44867301	1300	1	2.17E-07	5	0.384615385	Nefm	
DMR15:44899201	15	44899201	1800	1	2.07E-07	23	1.277777778		
DMR15:45743301	15	45743301	4000	2	4.74E-09	67	1.675	Fam124a	
DMR15:47658901	15	47658901	2500	2	1.57E-10	20	0.8	Msra	Metabolism
DMR15:47982801	15	47982801	2900	2	6.87E-11	28	0.965517241		
DMR15:48015701	15	48015701	2200	1	2.21E-07	26	1.181818182		
DMR15:50718101	15	50718101	300	1	4.28E-07	0	0		
DMR15:50721801	15	50721801	200	1	9.97E-07	1	0.5		
DMR15:50972701	15	50972701	2800	1	3.79E-07	11	0.392857143		
DMR15:51888601	15	51888601	500	2	2.78E-08	0	0	Sorbs3;Ppp3cc	Cytoskeleton;Signaling
DMR15:53119301	15	53119301	400	1	5.99E-07	2	0.5		
DMR15:53136101	15	53136101	3700	1	1.32E-08	40	1.081081081		
DMR15:53463601	15	53463601	800	1	9.43E-07	2	0.25		
DMR15:53897001	15	53897001	400	1	1.08E-07	0	0		
DMR15:54193901	15	54193901	2000	1	7.24E-07	17	0.85		
DMR15:54501301	15	54501301	4500	1	1.16E-07	55	1.222222222	Fndc3a	
DMR15:55010801	15	55010801	1700	3	1.95E-11	12	0.705882353		
DMR15:56459401	15	56459401	1200	2	4.02E-08	5	0.416666667		
DMR15:56844001	15	56844001	1300	2	1.30E-12	6	0.461538462	Lrch1	Unknown
DMR15:57728101	15	57728101	2800	3	2.23E-10	27	0.964285714		
DMR15:59811901	15	59811901	1100	1	6.72E-07	7	0.636363636	Enox1	Transcription

DMR15:60077301	15	60077301	2200	2	1.48E-08	22	1	Epsti1	Development
DMR15:60351501	15	60351501	1400	1	1.81E-07	22	1.571428571		
DMR15:60441101	15	60441101	5900	1	7.56E-07	70	1.186440678		
DMR15:61854201	15	61854201	4900	2	7.38E-10	42	0.857142857	Elf1	Transcription
DMR15:63626401	15	63626401	1200	1	6.03E-07	7	0.583333333		
DMR15:64791101	15	64791101	1600	1	5.42E-07	27	1.6875		
DMR15:65038001	15	65038001	1200	1	1.62E-07	17	1.416666667		
DMR15:65061901	15	65061901	2900	1	2.38E-08	13	0.448275862		
DMR15:65159201	15	65159201	2800	2	5.86E-08	7	0.25		
DMR15:65296301	15	65296301	400	1	9.08E-07	3	0.75		
DMR15:65388201	15	65388201	1800	1	1.55E-07	15	0.833333333		
DMR15:65601501	15	65601501	500	2	6.98E-08	2	0.4		
DMR15:67067101	15	67067101	300	1	5.36E-08	2	0.666666667		
DMR15:67340701	15	67340701	3100	1	5.58E-07	64	2.064516129		
DMR15:67560501	15	67560501	900	1	2.35E-14	18	2	Pcdh17	Cytoskeleton
DMR15:67693401	15	67693401	400	1	2.03E-09	1	0.25		
DMR15:68193401	15	68193401	2500	1	7.24E-07	16	0.64		
DMR15:69426701	15	69426701	400	1	5.21E-07	6	1.5		
DMR15:69538301	15	69538301	1300	1	2.79E-07	11	0.846153846		
DMR15:69982201	15	69982201	300	1	7.46E-08	0	0		
DMR15:71939901	15	71939901	500	1	2.23E-08	1	0.2		
DMR15:72281601	15	72281601	2000	1	6.52E-11	16	0.8		
DMR15:73334201	15	73334201	300	1	1.39E-08	0	0		
DMR15:73884701	15	73884701	800	1	9.32E-07	0	0		
DMR15:74662101	15	74662101	300	1	1.76E-07	0	0		
DMR15:74687601	15	74687601	700	1	3.22E-07	4	0.571428571		
DMR15:74962001	15	74962001	6100	1	3.81E-08	76	1.245901639		
DMR15:74996001	15	74996001	500	3	8.11E-12	0	0		
DMR15:76829701	15	76829701	700	2	2.30E-07	4	0.571428571		

DMR15:77123601	15	77123601	400	2	1.88E-07	0	0		
DMR15:77457801	15	77457801	800	1	7.01E-09	4	0.5		
DMR15:77547101	15	77547101	600	1	9.52E-07	2	0.333333333		
DMR15:78505001	15	78505001	3600	1	3.30E-08	47	1.305555556		
DMR15:78862201	15	78862201	1000	1	7.28E-08	4	0.4		
DMR15:79525201	15	79525201	1400	1	1.20E-07	8	0.571428571		
DMR15:79694801	15	79694801	3300	2	6.54E-10	30	0.909090909		
DMR15:81309101	15	81309101	3200	1	5.89E-08	47	1.46875		
DMR15:82453901	15	82453901	500	2	2.09E-09	2	0.4	Dach1	Transcription
DMR15:83194301	15	83194301	700	1	6.47E-08	3	0.428571429		
DMR15:84327801	15	84327801	700	1	3.65E-07	0	0	Klf12	Transcription
DMR15:86084701	15	86084701	2200	1	5.27E-08	17	0.772727273	Tbc1d4	
DMR15:86248001	15	86248001	500	1	4.82E-08	1	0.2	Lmo7	Cytoskeleton
DMR15:87261601	15	87261601	200	1	8.07E-09	1	0.5		
DMR15:87698401	15	87698401	600	2	3.02E-09	2	0.333333333	Mycbp2;Scel	Metabolism
DMR15:88514201	15	88514201	400	1	6.68E-07	1	0.25	Mycbp2	Metabolism
DMR15:88627101	15	88627101	300	1	2.16E-08	4	1.333333333	Mycbp2;Pou4f1;Rnf219	Metabolism;Transcription
DMR15:88632001	15	88632001	800	1	7.16E-09	19	2.375	Mycbp2;Pou4f1;Rnf219	Metabolism;Transcription
DMR15:88694101	15	88694101	1000	1	3.51E-08	8	0.8	Mycbp2	Metabolism
DMR15:88981201	15	88981201	3000	2	4.35E-07	34	1.133333333	Mycbp2	Metabolism
DMR15:89020901	15	89020901	2300	1	4.58E-07	19	0.826086957	Mycbp2;AABR07019099.1	Metabolism
DMR15:90265801	15	90265801	500	1	9.33E-08	4	0.8	Mycbp2	Metabolism
DMR15:91578601	15	91578601	800	2	8.81E-09	3	0.375	Mycbp2	Metabolism
DMR15:92049501	15	92049501	600	3	4.58E-13	0	0	Mycbp2	Metabolism
DMR15:93003601	15	93003601	2600	1	1.01E-09	17	0.653846154	Mycbp2	Metabolism
DMR15:93234401	15	93234401	600	1	1.99E-08	7	1.166666667	Mycbp2	Metabolism

DMR15:94765401	15	94765401	5700	2	3.69E-10	60	1.052631579		
DMR15:96087901	15	96087901	1800	1	6.76E-07	14	0.777777778		
DMR15:96670401	15	96670401	1500	2	5.23E-09	9	0.6		
DMR15:97034801	15	97034801	500	2	2.04E-08	2	0.4		
DMR15:97619301	15	97619301	1100	1	8.67E-07	9	0.818181818		
DMR15:98438801	15	98438801	1100	1	2.40E-07	5	0.454545455		
DMR15:98445901	15	98445901	500	2	1.68E-08	3	0.6		
DMR15:10007630	15	1E+08	200	2	4.21E-15	0	0		
DMR15:10022320	15	1E+08	400	1	7.24E-07	0	0		
DMR15:10036800	15	1E+08	2800	1	6.56E-09	16	0.571428571		
DMR15:10123770	15	1.01E+08	500	2	4.07E-08	0	0		
DMR15:10191860	15	1.02E+08	400	1	7.65E-07	1	0.25		
DMR15:10326810	15	1.03E+08	900	1	2.00E-08	21	2.333333333		
DMR15:10404900	15	1.04E+08	3000	2	8.82E-08	33	1.1	Cldn10	Cell Junction
DMR15:10470110	15	1.05E+08	1000	1	7.78E-07	4	0.4	Hs6st3	Metabolism
DMR15:10525980	15	1.05E+08	600	1	1.19E-10	5	0.833333333	Hs6st3	Metabolism
DMR15:10551460	15	1.06E+08	1300	1	7.87E-10	14	1.076923077	AABR07019437.6;AABR07019437.7;AABR07019437.4	
DMR15:10554600	15	1.06E+08	1600	2	4.16E-08	26	1.625	AABR07019437.4	
DMR15:10556180	15	1.06E+08	1900	1	4.42E-08	8	0.421052632	AABR07019437.4	
DMR15:10564980	15	1.06E+08	3100	1	1.15E-08	42	1.35483871	Mbnl2	Transcription
DMR15:10635850	15	1.06E+08	3300	2	1.65E-12	36	1.090909091		
DMR15:10646340	15	1.06E+08	800	1	3.74E-07	6	0.75	Farp1	Signaling
DMR15:10651440	15	1.07E+08	2800	1	2.26E-07	75	2.678571429	Farp1	Signaling
DMR15:10678110	15	1.07E+08	700	1	1.63E-07	3	0.428571429		
DMR15:10847700	15	1.08E+08	1000	1	2.21E-07	7	0.7		
DMR15:10908850	15	1.09E+08	500	1	4.00E-09	5	1	Pcca	Metabolism
DMR15:10992910	15	1.1E+08	2100	1	2.29E-12	21	1	Nalcn	Transport

DMR15:11008370	15	1.1E+08	300	1	5.16E-09	0	0		
DMR16:124501	16	124501	500	1	3.23E-09	2	0.4		
DMR16:1565701	16	1565701	600	1	3.61E-07	3	0.5		
DMR16:1611601	16	1611601	2800	2	4.41E-11	18	0.642857143		
DMR16:1682801	16	1682801	2400	1	2.04E-07	18	0.75		
DMR16:1843701	16	1843701	4000	9	8.18E-11	19	0.475	Zmiz1	Metabolism
DMR16:2018101	16	2018101	400	3	2.64E-13	0	0	Zcchc24	
DMR16:2185701	16	2185701	700	4	1.90E-12	1	0.142857143	Slmap;SNORA71	Protein Binding
DMR16:3302901	16	3302901	1300	1	2.13E-07	6	0.461538462	Erc2	Cytoskeleton
DMR16:3399701	16	3399701	900	3	6.31E-09	3	0.333333333	Erc2	Cytoskeleton
DMR16:4183101	16	4183101	400	1	2.28E-08	1	0.25		
DMR16:4567801	16	4567801	4400	3	1.77E-08	28	0.636363636		
DMR16:5555701	16	5555701	400	1	7.56E-07	4	1	Cacna2d3	Transport
DMR16:5576101	16	5576101	600	4	1.61E-12	1	0.166666667	Cacna2d3	Transport
DMR16:5659001	16	5659001	1100	1	4.31E-07	22	2	Cacna2d3	Transport
DMR16:6344801	16	6344801	1000	1	9.38E-07	10	1	Cacna1d	Metabolism
DMR16:6516001	16	6516001	500	1	3.53E-09	2	0.4		
DMR16:6608301	16	6608301	900	1	9.28E-08	7	0.777777778	Tkt	Metabolism
DMR16:6979801	16	6979801	2900	2	1.89E-08	35	1.206896552	Itih4;Itih3	Proteolysis;Signaling
DMR16:8195701	16	8195701	1600	1	3.09E-07	25	1.5625	Dph3;Oxnad1	Unknown;Metabolism
DMR16:8940201	16	8940201	1000	1	2.50E-09	2	0.2		
DMR16:9018401	16	9018401	400	1	5.01E-12	2	0.5	RGD1561145	
DMR16:9409101	16	9409101	800	1	4.97E-10	3	0.375	Wdfy4	
DMR16:9748701	16	9748701	3300	1	6.33E-07	23	0.696969697		
DMR16:9980601	16	9980601	1500	2	4.03E-07	15	1	Ptpn20	Signaling
DMR16:11166001	16	11166001	1100	3	8.91E-11	9	0.818181818		
DMR16:11460301	16	11460301	400	1	1.21E-07	2	0.5		

DMR16:11607801	16	11607801	300	1	8.88E-10	1	0.333333333	Grid1	Receptor
DMR16:11693101	16	11693101	1900	2	3.74E-08	20	1.052631579	Grid1	Receptor
DMR16:12369401	16	12369401	400	1	7.79E-07	0	0	uc_338	
DMR16:12664301	16	12664301	3100	1	9.40E-07	20	0.64516129	RGD1559508	Unknown
DMR16:12830101	16	12830101	2300	1	6.08E-07	2	0.086956522		
DMR16:12834701	16	12834701	1000	1	4.45E-08	8	0.8		
DMR16:13041801	16	13041801	1100	2	2.11E-12	5	0.454545455	AABR07024735.1	
DMR16:15103001	16	15103001	8000	1	2.68E-07	78	0.975	LOC683469	Translation
DMR16:15901701	16	15901701	300	1	1.33E-07	0	0		
DMR16:16706601	16	16706601	500	1	2.72E-07	4	0.8		
DMR16:17678801	16	17678801	1500	1	1.07E-08	13	0.866666667	Sh2d4b	
DMR16:17749501	16	17749501	300	1	1.58E-07	1	0.333333333		
DMR16:17838901	16	17838901	2700	1	1.36E-07	19	0.703703704		
DMR16:20048401	16	20048401	800	2	4.29E-10	3	0.375	Fam129c;Colgalt1;AC122603.2;Unc13a	Receptor
DMR16:20233901	16	20233901	2000	2	4.46E-09	11	0.55	AABR07024870.1	
DMR16:20851501	16	20851501	3800	1	6.12E-08	70	1.842105263	Upf1;Gdf1;Cers1;Cope	Transcription;Growth Factors & Cytokines;Transport
DMR16:21742501	16	21742501	1400	1	4.04E-07	5	0.357142857		
DMR16:22302001	16	22302001	500	1	8.41E-08	1	0.2	Lzts1;AABR07024951.1	
DMR16:22382301	16	22382301	1600	1	3.82E-08	11	0.6875	Slc18a1	Transport
DMR16:22960301	16	22960301	700	1	4.72E-10	3	0.428571429		
DMR16:23379201	16	23379201	800	2	4.30E-10	2	0.25		
DMR16:24109601	16	24109601	300	2	1.67E-07	0	0		
DMR16:24572001	16	24572001	2300	1	1.92E-10	13	0.565217391	Naf1	

DMR16:24903201	16	24903201	500	2	2.30E-07	2	0.4		
DMR16:25027901	16	25027901	1500	2	3.22E-07	5	0.333333333	1-Mar	Metabolism
DMR16:25126501	16	25126501	500	1	8.91E-08	1	0.2	1-Mar	Metabolism
DMR16:25157401	16	25157401	800	1	8.69E-08	2	0.25	1-Mar	Metabolism
DMR16:25525401	16	25525401	300	1	7.13E-07	7	2.333333333		
DMR16:25717501	16	25717501	4200	3	9.10E-09	39	0.928571429		
DMR16:26912701	16	26912701	400	1	7.48E-09	3	0.75	Cpe	Protease
DMR16:26941401	16	26941401	800	2	3.26E-07	4	0.5	Cpe	Protease
DMR16:28017601	16	28017601	500	1	8.49E-07	2	0.4		
DMR16:28990701	16	28990701	1700	1	1.18E-09	12	0.705882353		
DMR16:30180601	16	30180601	1800	1	1.10E-08	13	0.722222222		
DMR16:31650501	16	31650501	300	1	3.19E-07	4	1.333333333		
DMR16:32648701	16	32648701	3000	5	1.29E-11	25	0.833333333	AABR07025303.1	
DMR16:32802201	16	32802201	1800	1	6.67E-07	12	0.666666667		
DMR16:33089201	16	33089201	700	1	8.60E-08	0	0		
DMR16:34729301	16	34729301	2700	2	4.95E-08	21	0.777777778		
DMR16:35834801	16	35834801	1400	1	2.77E-08	16	1.142857143		
DMR16:36019901	16	36019901	600	1	2.75E-07	6	1	Galnt7	Metabolism
DMR16:36846501	16	36846501	1500	1	8.60E-08	5	0.333333333		
DMR16:37022901	16	37022901	500	2	4.52E-09	0	0		
DMR16:37043601	16	37043601	300	1	6.03E-07	2	0.666666667	7SK	
DMR16:37914401	16	37914401	400	2	2.99E-09	2	0.5	Glra3	Receptor
DMR16:39204701	16	39204701	800	1	1.99E-07	4	0.5		
DMR16:39228901	16	39228901	1800	1	1.75E-07	11	0.611111111		
DMR16:39418101	16	39418101	300	1	6.54E-07	1	0.333333333	Gpm6a	Cytoskeleton
DMR16:39915101	16	39915101	300	1	1.37E-07	3	1	Wdr17	
DMR16:40925401	16	40925401	700	3	5.14E-09	0	0		
DMR16:41056001	16	41056001	3500	1	1.52E-09	27	0.771428571		
DMR16:42781401	16	42781401	1800	1	7.98E-07	13	0.722222222		

DMR16:44790901	16	44790901	1400	2	9.66E-11	3	0.214285714	AABR07025662.1	
DMR16:45232701	16	45232701	300	1	4.57E-07	0	0		
DMR16:45369101	16	45369101	400	1	7.74E-07	2	0.5		
DMR16:45491201	16	45491201	400	1	2.58E-07	1	0.25		
DMR16:45514801	16	45514801	3300	2	1.97E-09	22	0.666666667		
DMR16:45599001	16	45599001	400	1	3.64E-09	0	0		
DMR16:46150401	16	46150401	400	2	8.08E-08	0	0		
DMR16:46280501	16	46280501	1000	2	1.28E-07	8	0.8		
DMR16:46427601	16	46427601	1200	1	1.78E-09	19	1.583333333		
DMR16:46500601	16	46500601	1100	1	3.50E-07	10	0.909090909		
DMR16:46723501	16	46723501	700	1	6.93E-08	1	0.142857143	Tenm3	
DMR16:46746801	16	46746801	300	2	1.88E-08	0	0	Tenm3	
DMR16:49114801	16	49114801	500	1	1.99E-07	1	0.2		
DMR16:49213501	16	49213501	1600	1	9.07E-08	13	0.8125		
DMR16:49875401	16	49875401	800	1	1.97E-07	7	0.875	SNORA17;SNORA43	
DMR16:50044901	16	50044901	400	1	9.23E-07	3	0.75	Fam149a	
DMR16:50113501	16	50113501	2000	1	5.34E-07	30	1.5	Fam149a;Cyp4v3	Metabolism
DMR16:51239101	16	51239101	3700	4	6.66E-12	31	0.837837838	U6	
DMR16:51428001	16	51428001	700	1	4.45E-10	1	0.142857143		
DMR16:51497801	16	51497801	1200	3	2.95E-15	2	0.166666667		
DMR16:51544101	16	51544101	300	1	5.85E-10	1	0.333333333		
DMR16:52648101	16	52648101	6200	1	2.65E-10	62	1		
DMR16:52943701	16	52943701	3700	1	4.82E-07	33	0.891891892		
DMR16:53956801	16	53956801	600	1	8.67E-07	5	0.833333333	Frg1	Transcription
DMR16:54195601	16	54195601	3200	1	6.13E-08	24	0.75	Fgl1	Signaling
DMR16:54872701	16	54872701	600	2	3.58E-09	0	0	Vps37a	
DMR16:55756701	16	55756701	6600	1	5.05E-09	66	1		
DMR16:55852801	16	55852801	300	1	8.69E-08	2	0.666666667		
DMR16:56583201	16	56583201	400	1	2.79E-07	1	0.25		

DMR16:56833001	16	56833001	200	2	1.26E-14	1	0.5	Msr1	Receptor
DMR16:57672701	16	57672701	800	1	4.79E-07	3	0.375		
DMR16:57753501	16	57753501	2900	2	5.84E-10	20	0.689655172		
DMR16:57947501	16	57947501	700	1	6.54E-08	1	0.142857143		
DMR16:58119501	16	58119501	500	1	6.00E-07	1	0.2		
DMR16:58331201	16	58331201	3400	2	4.02E-11	29	0.852941176		
DMR16:59163801	16	59163801	1000	1	1.13E-07	6	0.6	Dlc1	Signaling
DMR16:59919501	16	59919501	1500	1	7.95E-08	8	0.533333333		
DMR16:60211401	16	60211401	1800	3	1.30E-12	22	1.222222222	Mfhas1	Unknown
DMR16:60490801	16	60490801	400	1	1.64E-08	1	0.25		
DMR16:60883001	16	60883001	400	1	7.85E-08	8	2		
DMR16:60888301	16	60888301	300	1	1.09E-07	0	0		
DMR16:60900901	16	60900901	4200	1	7.94E-07	34	0.80952381		
DMR16:61576601	16	61576601	800	1	5.09E-07	48	6		
DMR16:63506701	16	63506701	3700	1	4.01E-07	40	1.081081081		
DMR16:63846801	16	63846801	700	1	2.91E-08	2	0.285714286	Nrg1	Signaling
DMR16:64896401	16	64896401	500	1	4.90E-07	8	1.6		
DMR16:64898101	16	64898101	4200	1	1.06E-07	19	0.452380952		
DMR16:64926601	16	64926601	9000	8	2.28E-08	58	0.644444444	AABR07026120.1	
DMR16:64939701	16	64939701	17900	1	7.21E-10	93	0.519553073		
DMR16:66061801	16	66061801	1700	3	1.43E-09	20	1.176470588		
DMR16:67045101	16	67045101	300	1	5.11E-07	0	0	LOC100910854	
DMR16:67134301	16	67134301	2000	2	7.57E-10	21	1.05	snoR12	
DMR16:67138301	16	67138301	3500	1	2.96E-08	32	0.914285714	snoR12	
DMR16:68841101	16	68841101	600	2	5.41E-13	5	0.833333333	Poteg	
DMR16:68975401	16	68975401	1400	1	1.14E-08	8	0.571428571	Eif4ebp1;Rn60_16_0690.3	Transcription
DMR16:69120701	16	69120701	2500	3	4.69E-08	17	0.68	Adgra2	
DMR16:69156201	16	69156201	500	1	1.65E-07	1	0.2	Prosc	Metabolism

DMR16:69439601	16	69439601	2300	1	5.14E-08	30	1.304347826		
DMR16:69481801	16	69481801	400	1	1.71E-07	0	0		
DMR16:69517601	16	69517601	400	2	4.43E-08	1	0.25		
DMR16:70402201	16	70402201	300	1	1.37E-09	3	1		
DMR16:70613201	16	70613201	3800	1	1.68E-08	58	1.526315789		
DMR16:71438301	16	71438301	500	1	3.60E-07	29	5.8	AABR07026311.1	
DMR16:71468401	16	71468401	300	1	2.37E-10	0	0		
DMR16:71765301	16	71765301	2500	1	5.19E-08	51	2.04	Plekha2	
DMR16:71869201	16	71869201	400	1	4.92E-08	0	0	Adam9	Protease
DMR16:71883001	16	71883001	2400	2	6.49E-07	25	1.041666667	Adam9;Adam32	Protease
DMR16:72201901	16	72201901	1400	2	3.70E-08	18	1.285714286	Adam18	Protease
DMR16:72241601	16	72241601	1000	4	7.56E-16	7	0.7		
DMR16:72250101	16	72250101	1100	1	8.79E-07	7	0.636363636		
DMR16:72270301	16	72270301	800	1	2.56E-08	0	0	Ido2	
DMR16:72493501	16	72493501	400	1	9.62E-08	2	0.5		
DMR16:72808801	16	72808801	1800	4	3.41E-10	12	0.666666667	Zmat4	Transcription
DMR16:73274901	16	73274901	900	2	1.02E-07	5	0.555555556		
DMR16:74061401	16	74061401	2000	1	6.60E-07	41	2.05	AABR07026361.1;AABR07026361.2	
DMR16:75007001	16	75007001	1100	1	3.32E-08	6	0.545454545	Fam90a1a	
DMR16:75431601	16	75431601	2900	1	7.23E-07	24	0.827586207	Defa7;Defa8	
DMR16:75981001	16	75981001	300	2	3.24E-09	0	0	Mcph1;Angpt2	DNA Repair;Signaling
DMR16:76532701	16	76532701	700	1	8.00E-07	15	2.142857143		
DMR16:77377501	16	77377501	900	3	1.06E-09	6	0.666666667		
DMR16:77986801	16	77986801	2700	1	2.73E-09	15	0.555555556	AABR07026473.1	
DMR16:78015501	16	78015501	1400	1	2.84E-08	16	1.142857143	AABR07026473.1	
DMR16:78495301	16	78495301	300	1	8.19E-07	0	0		

DMR16:78553701	16	78553701	1200	1	3.30E-08	11	0.916666667	Csmd1	Signaling
DMR16:79628301	16	79628301	400	2	1.08E-08	0	0	Myom2	Cytoskeleton
DMR16:79686001	16	79686001	4700	1	1.25E-09	57	1.212765957	Kbtbd11	Transcription
DMR16:80532601	16	80532601	2900	1	1.70E-07	43	1.482758621		
DMR16:81024401	16	81024401	900	1	5.22E-08	6	0.666666667	Tmco3;Dcun1d2;U6	Transport;Proteolysis
DMR16:81271701	16	81271701	1200	1	3.67E-08	9	0.75	AABR07026534.2	
DMR16:81336201	16	81336201	5100	1	4.24E-09	60	1.176470588	Rasa3	Signaling
DMR16:81597001	16	81597001	900	1	8.62E-09	4	0.444444444	LOC103693999;NEWGENE_1582994;U6	
DMR16:82339201	16	82339201	4400	1	2.63E-07	61	1.386363636	AABR07072528.2;AABR07072528.4	
DMR16:82367301	16	82367301	1100	3	1.29E-10	15	1.363636364	AABR07072528.1	
DMR16:82749201	16	82749201	800	3	2.56E-10	3	0.375		
DMR16:83505501	16	83505501	1000	3	7.38E-10	6	0.6		
DMR16:84031701	16	84031701	1600	1	1.74E-08	19	1.1875		
DMR16:84350601	16	84350601	400	1	2.63E-07	2	0.5		
DMR16:84562001	16	84562001	600	1	1.89E-07	9	1.5		
DMR16:84577401	16	84577401	300	1	4.21E-07	0	0	Myo16	Cytoskeleton
DMR16:84615601	16	84615601	600	2	2.45E-08	3	0.5	Myo16	Cytoskeleton
DMR16:84652701	16	84652701	5800	3	1.72E-09	91	1.568965517	Myo16	Cytoskeleton
DMR16:84688501	16	84688501	2300	1	8.55E-07	47	2.043478261	Myo16	Cytoskeleton
DMR16:84752301	16	84752301	2500	1	1.68E-07	7	0.28	Myo16	Cytoskeleton
DMR16:85113901	16	85113901	4100	1	1.95E-07	67	1.634146341	Marco	Receptor
DMR16:85271201	16	85271201	1100	1	4.91E-09	6	0.545454545	Tnfsf13b	
DMR16:85279801	16	85279801	1300	1	6.19E-07	15	1.153846154	Tnfsf13b	
DMR16:85820001	16	85820001	3100	1	6.26E-07	27	0.870967742		
DMR16:85851501	16	85851501	200	1	5.09E-07	3	1.5		

DMR16:85857101	16	85857101	1600	3	9.53E-10	9	0.5625		
DMR16:86262101	16	86262101	600	2	2.79E-08	13	2.166666667		
DMR16:86268801	16	86268801	300	1	5.58E-07	0	0		
DMR16:86439501	16	86439501	500	1	1.40E-07	1	0.2		
DMR16:86499101	16	86499101	600	2	1.56E-08	0	0		
DMR16:86860601	16	86860601	800	1	4.01E-08	1	0.125		
DMR16:88084201	16	88084201	1000	1	4.37E-07	23	2.3		
DMR16:88186601	16	88186601	400	2	5.62E-08	1	0.25		
DMR16:88672601	16	88672601	300	2	6.36E-07	0	0		
DMR16:88892601	16	88892601	600	2	2.82E-10	5	0.833333333		
DMR16:89021801	16	89021801	200	1	1.02E-08	2	1		
DMR16:89956101	16	89956101	300	1	1.33E-09	1	0.333333333		
DMR17:39101	17	39101	1700	1	3.25E-07	8	0.470588235		
DMR17:2176801	17	2176801	500	3	1.74E-07	1	0.2		
DMR17:2300501	17	2300501	300	1	3.13E-07	1	0.333333333		
DMR17:2946701	17	2946701	300	1	7.48E-07	1	0.333333333		
DMR17:3004001	17	3004001	400	1	1.41E-07	2	0.5		
DMR17:3084701	17	3084701	1900	1	7.93E-09	15	0.789473684		
DMR17:3142901	17	3142901	400	1	3.50E-07	2	0.5		
DMR17:3683201	17	3683201	2600	1	1.14E-07	18	0.692307692		
DMR17:3721001	17	3721001	2700	1	5.46E-09	23	0.851851852	AABR07026893.1	
DMR17:3861301	17	3861301	400	1	9.39E-07	5	1.25		
DMR17:4056201	17	4056201	500	2	7.04E-13	1	0.2	LOC100364523;Ctsql2	
DMR17:4210101	17	4210101	500	2	5.72E-09	2	0.4	Testin	
DMR17:4484601	17	4484601	1200	2	4.34E-09	6	0.5		
DMR17:4626601	17	4626601	600	1	7.65E-08	16	2.666666667		
DMR17:4959301	17	4959301	1400	1	3.61E-08	19	1.357142857		
DMR17:5163501	17	5163501	1300	2	3.66E-10	8	0.615384615		

DMR17:5354401	17	5354401	1900	2	4.59E-09	3	0.157894737	Spata31d3;LOC100912252	
DMR17:6107901	17	6107901	1200	1	8.69E-08	10	0.833333333	Ntrk2	Receptor
DMR17:6561601	17	6561601	2300	1	5.24E-07	16	0.695652174		
DMR17:7538401	17	7538401	3500	1	1.51E-10	48	1.371428571		
DMR17:8361501	17	8361501	1700	1	5.75E-07	30	1.764705882		
DMR17:8684601	17	8684601	1800	2	2.23E-07	18	1	AABR07026989.1	
DMR17:8844001	17	8844001	1000	3	1.69E-08	5	0.5		
DMR17:9058201	17	9058201	2000	3	7.78E-09	29	1.45	U6	
DMR17:9742801	17	9742801	500	2	1.29E-11	2	0.4	F12;Pfn3;Slc34a1	Protease;Cytoskeleton;Metabolism
DMR17:10241601	17	10241601	2300	2	1.21E-08	30	1.304347826		
DMR17:10300401	17	10300401	3100	1	5.56E-07	51	1.64516129		
DMR17:11005401	17	11005401	2700	1	7.21E-09	51	1.888888889		
DMR17:11193801	17	11193801	300	1	7.46E-07	2	0.666666667		
DMR17:11328601	17	11328601	900	3	1.48E-10	6	0.666666667		
DMR17:11504101	17	11504101	6200	2	4.64E-08	95	1.532258065		
DMR17:11528601	17	11528601	4200	1	7.57E-07	47	1.119047619		
DMR17:11756401	17	11756401	7700	1	7.08E-07	84	1.090909091		
DMR17:12302501	17	12302501	2700	1	1.61E-07	47	1.740740741	Auh	Metabolism
DMR17:12977201	17	12977201	4200	1	9.69E-07	53	1.261904762		
DMR17:13031201	17	13031201	1300	1	7.84E-07	10	0.769230769		
DMR17:13041701	17	13041701	800	1	2.45E-08	8	1		
DMR17:13446001	17	13446001	900	2	7.16E-08	10	1.111111111		
DMR17:13965701	17	13965701	1600	1	9.43E-07	27	1.6875		
DMR17:15765701	17	15765701	500	1	7.12E-08	4	0.8	Fgd3	
DMR17:16604001	17	16604001	700	1	5.80E-07	8	1.142857143		
DMR17:17152401	17	17152401	6000	1	1.38E-07	76	1.266666667		

DMR17:17623701	17	17623701	4100	1	4.62E-08	51	1.243902439		
DMR17:17779701	17	17779701	1200	1	4.48E-07	12	1		
DMR17:18708401	17	18708401	4700	1	1.39E-07	55	1.170212766		
DMR17:18831101	17	18831101	1800	1	7.92E-07	25	1.388888889	LOC102554838	
DMR17:18963801	17	18963801	4700	1	4.37E-07	64	1.361702128		
DMR17:18973001	17	18973001	1600	2	1.91E-09	14	0.875		
DMR17:18992201	17	18992201	700	5	6.20E-17	0	0		
DMR17:19219301	17	19219301	2200	1	2.87E-07	19	0.863636364		
DMR17:19370601	17	19370601	2100	1	1.25E-07	24	1.142857143	Atxn1	Transcription
DMR17:19414701	17	19414701	2200	3	2.44E-08	20	0.909090909	Atxn1	Transcription
DMR17:19522801	17	19522801	1600	2	7.48E-08	11	0.6875	Atxn1	Transcription
DMR17:19616701	17	19616701	1900	1	3.41E-07	18	0.947368421		
DMR17:19877501	17	19877501	1300	1	1.01E-07	10	0.769230769		
DMR17:19914101	17	19914101	400	1	7.12E-09	2	0.5		
DMR17:21168801	17	21168801	1000	1	4.97E-07	19	1.9	AABR07027244.1	
DMR17:22557201	17	22557201	1600	3	5.26E-13	14	0.875		
DMR17:22905401	17	22905401	1700	2	2.35E-08	13	0.764705882		
DMR17:23308601	17	23308601	700	1	3.11E-07	2	0.285714286		
DMR17:23355801	17	23355801	200	1	7.24E-07	0	0		
DMR17:24283801	17	24283801	400	1	1.03E-08	0	0	Rnf182	
DMR17:25328301	17	25328301	1800	1	3.26E-07	12	0.666666667	AABR07027342.1	
DMR17:25645301	17	25645301	300	1	7.28E-08	1	0.333333333		
DMR17:26269001	17	26269001	1000	1	4.13E-07	12	1.2		
DMR17:26657501	17	26657501	1200	1	9.68E-07	17	1.416666667		
DMR17:26686101	17	26686101	900	1	9.70E-07	7	0.777777778		
DMR17:27382501	17	27382501	400	1	7.40E-09	1	0.25	LOC102546383	
DMR17:27801701	17	27801701	1200	1	3.11E-07	5	0.416666667		
DMR17:27850301	17	27850301	1100	1	4.83E-07	23	2.090909091	AABR07027390.2	

DMR17:27860501	17	27860501	500	1	7.77E-07	4	0.8	AABR07027390.1;AABR07027390.3	
DMR17:27909701	17	27909701	500	1	1.09E-09	3	0.6		
DMR17:29305501	17	29305501	400	1	2.28E-07	0	0	Fars2	
DMR17:29837501	17	29837501	700	1	4.36E-09	3	0.428571429	Cdyl	Metabolism
DMR17:31587301	17	31587301	300	1	6.66E-08	2	0.666666667		
DMR17:33206201	17	33206201	1500	2	9.43E-08	13	0.866666667		
DMR17:34542601	17	34542601	700	1	7.46E-09	2	0.285714286	AABR07027501.1	
DMR17:35395901	17	35395901	300	1	2.72E-08	1	0.333333333	Exoc2	
DMR17:35860801	17	35860801	400	1	6.07E-07	2	0.5		
DMR17:35952101	17	35952101	1000	1	4.43E-08	17	1.7	Agtr1a	Receptor
DMR17:36075001	17	36075001	400	2	1.31E-07	0	0	Mboat1	Metabolism
DMR17:36445601	17	36445601	3300	3	2.23E-09	38	1.151515152	RGD1561310;Cdkal1	Cell Cycle
DMR17:37707801	17	37707801	1000	3	9.80E-10	1	0.1	AABR07027581.1	
DMR17:38154101	17	38154101	6100	1	5.04E-08	80	1.31147541		
DMR17:38409001	17	38409001	1000	1	6.81E-07	18	1.8		
DMR17:40783701	17	40783701	200	1	1.70E-07	2	1		
DMR17:42162101	17	42162101	400	1	6.12E-09	1	0.25	Aldh5a1;RGD1307443;LOC100360654	Metabolism
DMR17:43163401	17	43163401	1200	3	2.96E-10	2	0.166666667	Carmil1	
DMR17:43285401	17	43285401	1000	2	4.53E-12	3	0.3	Carmil1	
DMR17:43533101	17	43533101	500	1	6.26E-07	0	0	Slc17a3	Transport
DMR17:43883301	17	43883301	2000	1	1.03E-07	16	0.8	Btn2a2	
DMR17:44403801	17	44403801	5100	1	2.06E-08	119	2.333333333		
DMR17:46073301	17	46073301	900	2	2.44E-08	4	0.444444444	Aoah	Signaling
DMR17:46655701	17	46655701	1600	2	4.67E-08	10	0.625	Elmo1	Signaling
DMR17:47633901	17	47633901	500	1	7.98E-09	3	0.6	LOC498759	
DMR17:49759301	17	49759301	700	3	2.09E-10	0	0		

DMR17:51512101	17	51512101	2700	1	1.04E-07	18	0.666666667		
DMR17:51707801	17	51707801	500	1	5.55E-10	1	0.2		
DMR17:51887601	17	51887601	100	1	4.04E-11	0	0		
DMR17:52191601	17	52191601	3300	1	7.28E-08	31	0.939393939		
DMR17:52612501	17	52612501	400	1	2.44E-07	0	0		
DMR17:53030401	17	53030401	600	1	1.32E-07	5	0.833333333		
DMR17:54566101	17	54566101	300	2	3.26E-13	0	0		
DMR17:55595401	17	55595401	2300	2	2.84E-08	23	1		
DMR17:55614701	17	55614701	500	2	1.76E-08	1	0.2		
DMR17:56489001	17	56489001	700	1	2.82E-07	2	0.285714286		
DMR17:56580301	17	56580301	2200	1	3.53E-07	18	0.818181818		
DMR17:56881201	17	56881201	1000	1	2.48E-07	5	0.5	AABR07028094.1	
DMR17:57478901	17	57478901	300	1	6.32E-07	0	0	AABR07028113.1	
DMR17:57762001	17	57762001	1300	1	3.54E-07	22	1.692307692	RGD1564999	Metabolism
DMR17:58278001	17	58278001	500	2	5.61E-08	0	0	Adarb2	Metabolism
DMR17:58360101	17	58360101	400	2	6.34E-09	0	0		
DMR17:58684201	17	58684201	1200	1	9.89E-07	4	0.333333333	AABR07028172.1	
DMR17:60463501	17	60463501	1200	3	2.04E-07	4	0.333333333	Armc4	
DMR17:60528701	17	60528701	1500	1	3.89E-07	29	1.933333333	Mkx	Epigenetic
DMR17:61695001	17	61695001	300	1	2.87E-07	2	0.666666667		
DMR17:62350801	17	62350801	1700	1	5.44E-07	18	1.058823529	Ccny	Cell Cycle
DMR17:63510701	17	63510701	500	3	3.85E-11	4	0.8		
DMR17:63767601	17	63767601	900	5	1.96E-09	1	0.111111111	Dip2c	
DMR17:64003501	17	64003501	700	2	2.35E-08	3	0.428571429	Chrm3	Receptor
DMR17:64167801	17	64167801	500	1	5.83E-07	1	0.2		
DMR17:64793801	17	64793801	600	1	3.16E-09	0	0		
DMR17:64897901	17	64897901	700	1	7.41E-08	5	0.714285714		
DMR17:65038501	17	65038501	400	1	1.08E-08	1	0.25		
DMR17:65423101	17	65423101	900	1	7.57E-07	6	0.666666667		

DMR17:65580501	17	65580501	300	1	7.40E-09	1	0.333333333	Ryr2	Receptor
DMR17:65582301	17	65582301	500	1	9.59E-08	4	0.8	Ryr2	Receptor
DMR17:65678501	17	65678501	500	1	6.26E-09	1	0.2	Ryr2	Receptor
DMR17:65836101	17	65836101	600	1	9.21E-09	4	0.666666667	Ryr2	Receptor
DMR17:65907601	17	65907601	1000	3	6.29E-08	7	0.7	Ryr2	Receptor
DMR17:66297601	17	66297601	800	1	2.57E-07	2	0.25	Mtr;Actn2	Epigenetic;Cytoskeleton
DMR17:66395801	17	66395801	2700	2	3.74E-07	72	2.666666667	Actn2	Cytoskeleton
DMR17:68467701	17	68467701	300	1	2.60E-07	3	1	Pitrm1	Protease
DMR17:69654301	17	69654301	500	1	1.47E-08	1	0.2	Akr1c13;Akr1c19	Metabolism
DMR17:70470701	17	70470701	500	1	2.62E-07	0	0	Il15ra	Immune
DMR17:70569101	17	70569101	200	2	1.33E-10	0	0		
DMR17:70731501	17	70731501	1200	1	1.04E-07	9	0.75		
DMR17:70968801	17	70968801	600	4	6.24E-13	1	0.166666667	Prkcq	Binding Protein
DMR17:71120601	17	71120601	1700	3	9.64E-08	3	0.176470588		
DMR17:71124601	17	71124601	500	1	5.55E-08	0	0		
DMR17:71317501	17	71317501	700	5	5.14E-13	0	0		
DMR17:71347401	17	71347401	1500	2	1.07E-07	16	1.066666667		
DMR17:71708701	17	71708701	4000	2	1.94E-08	43	1.075		
DMR17:72872401	17	72872401	800	1	7.50E-09	5	0.625		
DMR17:73453501	17	73453501	400	1	2.21E-08	3	0.75		
DMR17:74820401	17	74820401	1300	1	8.33E-07	6	0.461538462		
DMR17:75048901	17	75048901	800	1	3.83E-07	5	0.625		
DMR17:75086801	17	75086801	700	1	7.16E-09	2	0.285714286		
DMR17:75130901	17	75130901	700	1	7.55E-08	5	0.714285714		
DMR17:75171201	17	75171201	700	1	2.86E-07	9	1.285714286		
DMR17:75915001	17	75915001	900	3	1.56E-10	1	0.111111111		
DMR17:76302701	17	76302701	2900	1	9.98E-07	35	1.206896552	AC141220.1;Dhtkd1	Metabolism
DMR17:76510601	17	76510601	4100	2	4.29E-14	69	1.682926829		

DMR17:76548801	17	76548801	700	2	3.64E-08	11	1.571428571	Camk1d	Signaling
DMR17:76706301	17	76706301	3500	1	7.28E-09	46	1.314285714	Camk1d	Signaling
DMR17:77093801	17	77093801	300	1	9.70E-07	3	1	Ccdc3;AC105577.1	Transcription
DMR17:77122501	17	77122501	1200	1	4.07E-07	5	0.416666667		
DMR17:77277101	17	77277101	1600	1	5.13E-07	32	2	Phyh	
DMR17:77612101	17	77612101	500	1	2.45E-07	1	0.2	Prpf18	Transcription
DMR17:77838801	17	77838801	300	1	7.96E-10	3	1		
DMR17:79024201	17	79024201	1700	1	2.42E-07	21	1.235294118	Fam171a1	
DMR17:79640001	17	79640001	3900	2	4.07E-07	32	0.820512821	Itga8	Extracellular Matrix
DMR17:80063301	17	80063301	600	1	3.19E-07	3	0.5	LOC102548534	
DMR17:80507701	17	80507701	800	1	2.76E-08	1	0.125	Rsu1	Signaling
DMR17:80872901	17	80872901	700	1	4.37E-07	3	0.428571429	Vim	Cytoskeleton
DMR17:81182401	17	81182401	1000	1	3.87E-07	17	1.7	Hacd1	
DMR17:81346601	17	81346601	2900	1	4.84E-09	26	0.896551724	Tmem236;Mrc1	Receptor
DMR17:81403801	17	81403801	900	3	4.11E-09	15	1.666666667	Mrc1	Receptor
DMR17:81680501	17	81680501	1100	1	3.29E-08	9	0.818181818		
DMR17:82445601	17	82445601	1100	2	1.13E-07	2	0.181818182	Malrd1	
DMR17:83143701	17	83143701	600	2	2.58E-28	8	1.333333333		
DMR17:84018701	17	84018701	800	2	7.93E-08	6	0.75		
DMR17:84692301	17	84692301	400	1	2.51E-07	9	2.25		
DMR17:85797701	17	85797701	4200	1	7.65E-07	33	0.785714286		
DMR17:85944301	17	85944301	3400	1	4.90E-07	32	0.941176471	Armc3;AABR07028779.1	
DMR17:86312901	17	86312901	1400	1	2.34E-09	12	0.857142857	LOC681241	
DMR17:86755501	17	86755501	700	2	1.12E-07	4	0.571428571		
DMR17:87911001	17	87911001	300	1	5.55E-07	1	0.333333333		
DMR17:88082101	17	88082101	2400	1	4.50E-07	42	1.75	Enkur;7SK	Metabolism
DMR17:88267601	17	88267601	400	1	6.08E-10	0	0	Gpr158	Receptor

DMR17:88438101	17	88438101	1600	3	9.88E-14	16	1	Gpr158	Receptor
DMR17:88901601	17	88901601	600	1	8.40E-07	0	0		
DMR17:89241101	17	89241101	700	1	8.36E-08	4	0.571428571	Gad2	Metabolism
DMR17:89990801	17	89990801	300	1	7.13E-07	0	0	Abi1	Signaling
DMR17:90758901	17	90758901	800	1	5.93E-07	1	0.125	Ero1b	
DMR18:833701	18	833701	1500	1	4.31E-07	14	0.933333333		
DMR18:1527901	18	1527901	1500	1	7.77E-07	6	0.4		
DMR18:2508401	18	2508401	300	1	1.11E-07	0	0	AABR07031164.1	
DMR18:2803501	18	2803501	1400	1	1.64E-08	17	1.214285714		
DMR18:3731101	18	3731101	2100	1	2.75E-07	37	1.761904762	Lama3	Extracellular Matrix
DMR18:4039701	18	4039701	3100	1	6.93E-08	40	1.290322581	Ttc39c	Unknown
DMR18:4158501	18	4158501	900	1	7.62E-07	5	0.555555556	Osbpl1a	Binding Protein
DMR18:4389601	18	4389601	300	1	1.52E-07	4	1.333333333	Hrh4	Receptor
DMR18:4438801	18	4438801	6100	2	7.65E-08	60	0.983606557		
DMR18:4513701	18	4513701	300	1	1.35E-07	0	0		
DMR18:4708501	18	4708501	700	2	1.04E-10	4	0.571428571		
DMR18:6033301	18	6033301	600	2	7.82E-09	3	0.5	Ss18	Transcription
DMR18:6529901	18	6529901	2800	2	4.75E-09	26	0.928571429		
DMR18:8379601	18	8379601	800	3	2.45E-09	0	0		
DMR18:10559201	18	10559201	2500	2	1.74E-08	32	1.28		
DMR18:11155201	18	11155201	3500	1	1.38E-09	49	1.4		
DMR18:11502401	18	11502401	2800	1	7.98E-08	20	0.714285714		
DMR18:11652301	18	11652301	700	2	2.16E-08	28	4		
DMR18:12480901	18	12480901	600	4	4.12E-12	1	0.166666667		
DMR18:12810101	18	12810101	400	3	2.95E-08	2	0.5	Ccdc178	
DMR18:13448501	18	13448501	300	1	3.64E-07	0	0	Asxl3	
DMR18:13487401	18	13487401	600	2	1.29E-13	2	0.333333333	Asxl3	
DMR18:14549101	18	14549101	400	1	4.37E-07	3	0.75	Dtna	Cytoskeleton

DMR18:14692301	18	14692301	1900	1	1.00E-07	33	1.736842105		
DMR18:15398701	18	15398701	2900	1	1.29E-07	38	1.310344828		
DMR18:16257501	18	16257501	2500	1	1.56E-07	22	0.88		
DMR18:16314701	18	16314701	2500	1	2.27E-09	26	1.04		
DMR18:17176801	18	17176801	600	2	3.53E-13	0	0	Fhod3;Rn60_18_0172.2	Cytoskeleton
DMR18:17648201	18	17648201	200	1	7.54E-07	4	2	RGD1562608	EST
DMR18:17998901	18	17998901	1600	1	3.14E-07	14	0.875	AABR07031533.1	
DMR18:19982501	18	19982501	400	1	9.68E-07	4	1		
DMR18:20617401	18	20617401	500	2	4.73E-09	1	0.2		
DMR18:21184601	18	21184601	500	1	2.13E-08	2	0.4		
DMR18:21543801	18	21543801	500	2	7.97E-10	0	0		
DMR18:23070801	18	23070801	600	3	1.18E-08	0	0		
DMR18:23455501	18	23455501	600	1	1.03E-07	6	1		
DMR18:24212701	18	24212701	400	1	1.54E-07	8	2	SNORA17	
DMR18:24663001	18	24663001	1400	1	1.75E-07	26	1.857142857	Wdr33	Unknown
DMR18:24845401	18	24845401	800	2	1.06E-07	3	0.375	Iws1	
DMR18:24913201	18	24913201	5000	2	4.76E-07	59	1.18	Proc	
DMR18:25403901	18	25403901	300	1	6.28E-07	0	0	SNORA17	
DMR18:25480501	18	25480501	500	2	1.09E-11	2	0.4	SNORA62	
DMR18:26201801	18	26201801	2500	1	3.44E-08	39	1.56	Nrep	Development
DMR18:26418301	18	26418301	500	2	3.47E-08	2	0.4	U6	
DMR18:26893901	18	26893901	1000	2	2.47E-10	9	0.9		
DMR18:26971501	18	26971501	700	1	9.07E-07	10	1.428571429	U7	
DMR18:28722301	18	28722301	1400	2	1.08E-07	11	0.785714286		
DMR18:30007201	18	30007201	900	1	2.87E-08	0	0	Pcdha4	
DMR18:31953601	18	31953601	400	1	6.67E-07	9	2.25	Arhgap26	Signaling
DMR18:32819601	18	32819601	1400	1	6.34E-07	9	0.642857143		
DMR18:32851701	18	32851701	1600	1	2.74E-07	15	0.9375		

DMR18:32941201	18	32941201	6300	2	6.64E-09	70	1.111111111		
DMR18:33046001	18	33046001	1200	2	1.32E-10	6	0.5		
DMR18:34248801	18	34248801	2400	1	3.02E-07	22	0.916666667		
DMR18:34687401	18	34687401	400	2	7.44E-10	0	0		
DMR18:36019801	18	36019801	300	1	1.78E-09	2	0.666666667		
DMR18:36366701	18	36366701	2400	1	7.92E-10	31	1.291666667	Sh3rf2	
DMR18:37096901	18	37096901	1300	2	1.13E-12	12	0.923076923	Ppp2r2b	Signaling
DMR18:38075501	18	38075501	300	2	2.72E-09	5	1.666666667	Jakmip2	
DMR18:38919801	18	38919801	1700	1	7.52E-07	16	0.941176471		
DMR18:38925501	18	38925501	1000	2	1.92E-08	5	0.5		
DMR18:38972701	18	38972701	600	4	3.39E-12	1	0.166666667		
DMR18:43364001	18	43364001	5600	1	8.14E-07	61	1.089285714		
DMR18:44018301	18	44018301	200	1	1.23E-07	0	0		
DMR18:44485601	18	44485601	400	1	2.34E-07	1	0.25	Dmxl1	
DMR18:44749701	18	44749701	1200	2	1.68E-07	19	1.583333333	Tnfaip8	Unknown
DMR18:44752201	18	44752201	1500	2	8.46E-08	21	1.4	Tnfaip8	Unknown
DMR18:45587801	18	45587801	400	1	5.91E-07	2	0.5		
DMR18:46078401	18	46078401	1400	1	4.49E-07	8	0.571428571		
DMR18:46505501	18	46505501	1100	1	1.99E-09	19	1.727272727		
DMR18:47573501	18	47573501	1400	1	4.16E-08	12	0.857142857	Lox;Y_RNA;Zfp474	Metabolism
DMR18:48416501	18	48416501	2200	1	7.68E-07	16	0.727272727		
DMR18:50422101	18	50422101	1600	1	1.98E-07	7	0.4375	AABR07032162.1	
DMR18:52546201	18	52546201	2300	1	1.26E-07	28	1.217391304	Ctxn3	
DMR18:52557001	18	52557001	600	1	4.26E-07	0	0	Ctxn3	
DMR18:52880901	18	52880901	3100	1	2.10E-07	24	0.774193548		
DMR18:53481901	18	53481901	800	1	7.56E-08	3	0.375	AC107505.1	
DMR18:53970701	18	53970701	2800	1	7.73E-07	27	0.964285714	Adamts19	Proteolysis
DMR18:53977001	18	53977001	2200	3	1.56E-09	21	0.954545455	Adamts19	Proteolysis
DMR18:54308901	18	54308901	1800	1	1.86E-07	10	0.555555556	Chsy3	Metabolism

DMR18:55945401	18	55945401	1800	1	6.89E-07	23	1.277777778	AC095289.1;Ndst1	Metabolism
DMR18:56368901	18	56368901	1900	2	5.55E-08	23	1.210526316	Pdgfrb	Receptor
DMR18:56874601	18	56874601	1000	1	4.96E-07	6	0.6	Arhgef37	
DMR18:57175401	18	57175401	3000	1	6.36E-07	17	0.566666667	Ablim3	Cytoskeleton
DMR18:57230101	18	57230101	2400	1	1.08E-08	33	1.375	Ablim3	Cytoskeleton
DMR18:57262201	18	57262201	400	1	8.81E-07	1	0.25		
DMR18:58108901	18	58108901	1900	1	9.68E-07	21	1.105263158		
DMR18:58204901	18	58204901	1100	1	3.78E-07	7	0.636363636		
DMR18:58529201	18	58529201	400	2	8.85E-12	0	0		
DMR18:59431901	18	59431901	2500	1	2.50E-07	18	0.72		
DMR18:59439901	18	59439901	800	1	3.96E-07	1	0.125		
DMR18:59616201	18	59616201	900	1	6.02E-07	9	1		
DMR18:60446701	18	60446701	2000	1	3.21E-07	45	2.25	Nedd4l	Protease
DMR18:62418501	18	62418501	800	1	1.24E-09	6	0.75		
DMR18:63103201	18	63103201	700	3	3.24E-11	4	0.571428571	Cidea	Unknown
DMR18:63976201	18	63976201	1200	1	2.04E-07	25	2.083333333	Ldlrad4	Receptor
DMR18:64361001	18	64361001	600	1	8.75E-08	4	0.666666667		
DMR18:64461301	18	64461301	400	2	5.59E-11	0	0	U4	
DMR18:64670501	18	64670501	5500	1	2.97E-07	30	0.545454545		
DMR18:64723601	18	64723601	2400	1	3.46E-07	20	0.833333333		
DMR18:65027201	18	65027201	1600	2	1.67E-12	25	1.5625		
DMR18:65127101	18	65127101	300	1	2.44E-07	5	1.666666667		
DMR18:65903601	18	65903601	200	1	8.28E-08	0	0		
DMR18:67837701	18	67837701	500	2	3.98E-13	0	0		
DMR18:68032401	18	68032401	2400	1	4.23E-07	19	0.791666667		
DMR18:68637801	18	68637801	1300	1	4.47E-08	9	0.692307692	AABR07032496.1	
DMR18:69420501	18	69420501	3500	1	6.91E-08	48	1.371428571		
DMR18:69865101	18	69865101	9500	7	3.20E-12	71	0.747368421	AABR07032520.2	
DMR18:70508901	18	70508901	4100	1	1.30E-08	63	1.536585366	Myo5b	Cytoskeleton

DMR18:70810901	18	70810901	1800	1	3.02E-08	19	1.055555556		
DMR18:70892501	18	70892501	2100	1	2.96E-10	40	1.904761905	Lipg	Metabolism
DMR18:70937201	18	70937201	2900	3	2.67E-08	53	1.827586207		
DMR18:70941901	18	70941901	2900	1	8.29E-07	41	1.413793103		
DMR18:72267901	18	72267901	2300	1	7.02E-09	35	1.52173913	Zbtb7c	Transcription
DMR18:72813601	18	72813601	1200	1	2.11E-07	4	0.333333333		
DMR18:72940401	18	72940401	500	3	3.07E-08	1	0.2		
DMR18:73065601	18	73065601	2200	1	6.38E-07	27	1.227272727		
DMR18:73338801	18	73338801	900	3	3.30E-08	3	0.333333333	Katnal2	Cytoskeleton
DMR18:73614701	18	73614701	400	1	3.61E-07	2	0.5	St8sia5	Metabolism
DMR18:73689801	18	73689801	1900	1	7.29E-07	31	1.631578947	Loxhd1	
DMR18:73732201	18	73732201	2000	2	2.13E-07	35	1.75	Loxhd1	
DMR18:74483001	18	74483001	600	1	3.89E-07	6	1	Slc14a1	Metabolism
DMR18:74593301	18	74593301	2800	1	5.32E-07	33	1.178571429	Slc14a2	Transport
DMR18:74841201	18	74841201	5500	10	3.72E-15	70	1.272727273		
DMR18:74920401	18	74920401	100	1	8.14E-07	2	2		
DMR18:75130901	18	75130901	200	1	7.33E-07	0	0	Setbp1	Transcription
DMR18:75618001	18	75618001	800	1	1.48E-07	2	0.25		
DMR18:77215601	18	77215601	1100	2	4.74E-16	24	2.181818182	Nfatc1	Transcription
DMR18:77488201	18	77488201	1100	1	9.71E-07	5	0.454545455	Atp9b	Transport
DMR18:77992201	18	77992201	700	2	4.11E-10	4	0.571428571		
DMR18:78251101	18	78251101	1300	1	3.80E-08	5	0.384615385		
DMR18:78869101	18	78869101	700	1	7.30E-07	4	0.571428571		
DMR18:78952101	18	78952101	1300	4	2.19E-11	7	0.538461538	AABR07032747.1	
DMR18:79429401	18	79429401	600	1	2.94E-10	3	0.5	Mbp	Unknown
DMR18:79461401	18	79461401	1200	1	1.07E-08	10	0.833333333	Zfp236	Transcription
DMR18:79514401	18	79514401	1200	1	7.42E-07	0	0	Zfp236	Transcription
DMR18:79823401	18	79823401	600	1	2.67E-07	18	3	Zfp516	Transcription
DMR18:80003701	18	80003701	1000	3	1.26E-09	2	0.2		

DMR18:80058401	18	80058401	1400	1	5.99E-07	14	1		
DMR18:80248001	18	80248001	300	1	7.45E-07	2	0.666666667		
DMR18:80369901	18	80369901	500	1	3.00E-07	3	0.6		
DMR18:81197701	18	81197701	700	1	8.45E-08	4	0.571428571	Zfp407	Transcription
DMR18:81318201	18	81318201	600	3	2.41E-10	0	0	Zfp407	Transcription
DMR18:81382801	18	81382801	1800	2	1.51E-07	21	1.166666667	Zfp407	Transcription
DMR18:81400501	18	81400501	300	1	5.12E-07	3	1	Zfp407	Transcription
DMR18:81459101	18	81459101	3700	2	9.81E-08	44	1.189189189	Cndp1	Protease
DMR18:81612501	18	81612501	1100	2	4.53E-11	5	0.454545455		
DMR18:81769801	18	81769801	1600	1	8.21E-07	24	1.5		
DMR18:81893001	18	81893001	300	1	4.21E-07	3	1		
DMR18:82763501	18	82763501	400	1	7.64E-07	2	0.5	AABR07032806.1	
DMR18:83057201	18	83057201	2000	2	2.75E-07	18	0.9		
DMR18:83554601	18	83554601	500	1	3.49E-08	1	0.2	Neto1	
DMR18:83640301	18	83640301	2500	2	6.55E-10	10	0.4		
DMR18:85098901	18	85098901	600	2	3.26E-08	2	0.333333333		
DMR18:87417301	18	87417301	600	1	1.85E-09	1	0.166666667	AABR07032888.1	
DMR18:87452601	18	87452601	500	1	2.21E-09	0	0		
DMR18:87946901	18	87946901	3000	1	1.32E-07	16	0.533333333		
DMR18:87991001	18	87991001	1000	1	5.28E-07	8	0.8		
DMR18:88146901	18	88146901	300	1	3.00E-07	3	1		
DMR19:148401	19	148401	1300	1	2.56E-07	9	0.692307692		
DMR19:837801	19	837801	800	1	7.95E-07	30	3.75	Cmtm3;LOC102554842	Signaling
DMR19:975701	19	975701	500	1	2.86E-07	1	0.2	Bean1	
DMR19:1169801	19	1169801	800	1	1.67E-08	1	0.125		
DMR19:1609701	19	1609701	500	1	2.30E-07	1	0.2	LOC102556295	
DMR19:2670901	19	2670901	1200	1	3.26E-07	6	0.5		
DMR19:3651601	19	3651601	1300	1	7.82E-07	10	0.769230769		

DMR19:3666101	19	3666101	1800	1	7.84E-07	10	0.555555556		
DMR19:4736801	19	4736801	900	1	2.75E-07	3	0.333333333		
DMR19:4858801	19	4858801	1100	1	3.46E-08	8	0.727272727		
DMR19:6856201	19	6856201	300	1	1.92E-07	2	0.666666667		
DMR19:7711301	19	7711301	400	2	4.90E-08	6	1.5		
DMR19:7972501	19	7972501	1100	2	1.36E-08	4	0.363636364		
DMR19:8263501	19	8263501	1000	2	3.56E-08	5	0.5	AABR07042780.1	
DMR19:8629701	19	8629701	1200	1	6.23E-12	2	0.166666667		
DMR19:9499601	19	9499601	1900	1	6.03E-08	24	1.263157895		
DMR19:9562901	19	9562901	3000	2	4.84E-08	45	1.5	AABR07042802.1	
DMR19:10464901	19	10464901	1500	1	7.40E-11	12	0.8	Adgrg1	
DMR19:10685701	19	10685701	700	1	5.34E-07	2	0.285714286	Ccl22	Signaling
DMR19:11612301	19	11612301	400	1	1.67E-07	4	1	Gnao1	Signaling
DMR19:13188701	19	13188701	2300	1	3.79E-07	19	0.826086957		
DMR19:13936901	19	13936901	900	1	1.80E-07	4	0.444444444		
DMR19:14656101	19	14656101	700	1	7.03E-07	15	2.142857143	AABR07042911.1;Rasd2	Signaling
DMR19:15148601	19	15148601	700	1	3.16E-07	7	1	Ces1d;Ces1f	Metabolism
DMR19:15286901	19	15286901	4400	2	2.98E-08	17	0.386363636	Ces1f	
DMR19:15386401	19	15386401	800	1	8.29E-07	0	0	Slc6a2	Metabolism
DMR19:15775701	19	15775701	2200	1	8.51E-10	27	1.227272727		
DMR19:15937801	19	15937801	500	1	2.14E-08	0	0		
DMR19:16350901	19	16350901	200	2	8.19E-11	0	0		
DMR19:16640601	19	16640601	2000	1	2.64E-07	6	0.3		
DMR19:17347601	19	17347601	4300	1	3.58E-07	44	1.023255814	Rbl2;SNORA17	Development
DMR19:17540001	19	17540001	700	1	1.60E-07	9	1.285714286		
DMR19:18111601	19	18111601	600	1	1.07E-07	3	0.5	Tox3	Transcription
DMR19:18953901	19	18953901	300	1	6.28E-07	2	0.666666667		
DMR19:19074701	19	19074701	2600	1	7.14E-09	32	1.230769231		

DMR19:19640101	19	19640101	3500	1	6.54E-07	44	1.257142857		
DMR19:19943401	19	19943401	200	1	7.67E-07	0	0		
DMR19:20007401	19	20007401	700	1	5.54E-07	3	0.428571429		
DMR19:20057701	19	20057701	300	1	4.87E-08	1	0.333333333		
DMR19:21294301	19	21294301	1100	2	4.50E-09	6	0.545454545		
DMR19:21310701	19	21310701	2100	1	2.13E-08	25	1.19047619		
DMR19:21500601	19	21500601	2800	2	1.60E-09	32	1.142857143	Lonp2	Protease
DMR19:22287901	19	22287901	200	1	1.71E-07	6	3	Phkb;Itfg1	Signaling
DMR19:22652801	19	22652801	500	1	8.06E-10	0	0		
DMR19:23158601	19	23158601	700	1	8.42E-07	2	0.285714286		
DMR19:23564901	19	23564901	2000	1	1.97E-07	9	0.45		
DMR19:24004001	19	24004001	2700	1	6.40E-08	21	0.777777778		
DMR19:24606501	19	24606501	500	2	1.58E-08	0	0	Scoc	Golgi
DMR19:25957301	19	25957301	300	1	3.54E-08	3	1	Gadd45gip1;Rad23a;Calr	Epigenetic;Proteolysis;Signaling
DMR19:27756501	19	27756501	1200	1	8.60E-09	6	0.5	AABR07043358.1	
DMR19:28672501	19	28672501	2300	1	8.78E-07	21	0.913043478	AABR07043453.1	
DMR19:28929001	19	28929001	3100	1	5.93E-07	37	1.193548387	U6	
DMR19:29283801	19	29283801	2200	1	2.64E-07	20	0.909090909	AABR07043564.1	
DMR19:30222901	19	30222901	1100	1	9.71E-07	4	0.363636364		
DMR19:30315501	19	30315501	600	1	8.78E-07	6	1		
DMR19:31579601	19	31579601	500	1	3.48E-07	0	0	Hhip	Receptor
DMR19:32135701	19	32135701	700	1	5.74E-07	7	1		
DMR19:32383701	19	32383701	3800	2	9.05E-11	36	0.947368421	LOC498933	
DMR19:33122601	19	33122601	2500	1	5.45E-07	23	0.92	LOC102550668	
DMR19:33703101	19	33703101	600	1	8.97E-07	4	0.666666667		
DMR19:34010901	19	34010901	800	1	5.56E-07	7	0.875	AABR07043652.1	
DMR19:34223001	19	34223001	4400	2	4.07E-09	130	2.954545455	Arhgap10	Signaling

DMR19:36472401	19	36472401	400	1	7.82E-07	2	0.5		
DMR19:36682901	19	36682901	4100	3	3.25E-08	28	0.682926829		
DMR19:37848301	19	37848301	300	1	2.75E-07	2	0.666666667	Nutf2;Nrn1l	Unknown
DMR19:39070201	19	39070201	300	1	2.51E-08	3	1	Has3;Chtf8	Metabolism
DMR19:39268201	19	39268201	2700	1	7.05E-07	35	1.296296296	Nip7;Tmed6;Terf2	Transcription;Binding Protein
DMR19:39798201	19	39798201	1900	2	3.35E-08	23	1.210526316		
DMR19:41080501	19	41080501	700	1	7.94E-07	6	0.857142857	Hydin	Unknown
DMR19:41093401	19	41093401	1300	1	8.52E-08	11	0.846153846	Hydin	Unknown
DMR19:41511301	19	41511301	1200	2	2.33E-08	13	1.083333333	Calb2	Signaling
DMR19:41516801	19	41516801	1000	1	4.30E-08	9	0.9	Calb2	Signaling
DMR19:42319001	19	42319001	300	1	5.55E-08	5	1.666666667		
DMR19:42618801	19	42618801	2400	1	9.10E-07	24	1		
DMR19:42976101	19	42976101	2800	2	1.29E-08	29	1.035714286	Rfwd3;Glg1	Metabolism;Signaling
DMR19:43460701	19	43460701	2100	1	4.70E-07	22	1.047619048		
DMR19:44292801	19	44292801	2800	1	2.56E-07	31	1.107142857	U6	
DMR19:44328901	19	44328901	2600	1	3.04E-07	32	1.230769231		
DMR19:44455201	19	44455201	700	1	1.72E-07	3	0.428571429		
DMR19:44921801	19	44921801	1300	1	2.48E-07	15	1.153846154	Cntnap4	Signaling
DMR19:45457901	19	45457901	300	2	1.70E-08	2	0.666666667	AABR07043877.3	
DMR19:46136101	19	46136101	600	3	7.67E-13	0	0		
DMR19:46367701	19	46367701	500	2	2.57E-11	0	0		
DMR19:46517901	19	46517901	3800	1	7.95E-07	61	1.605263158	Vat1l	Metabolism
DMR19:46967501	19	46967501	1800	1	7.76E-08	22	1.222222222		
DMR19:47015701	19	47015701	700	3	5.45E-12	10	1.428571429		
DMR19:47084101	19	47084101	3100	1	1.82E-07	30	0.967741935		
DMR19:47151301	19	47151301	8200	2	2.83E-08	115	1.402439024		
DMR19:47271101	19	47271101	3800	1	1.12E-08	49	1.289473684		

DMR19:47518901	19	47518901	300	1	6.47E-08	5	1.666666667		
DMR19:47522601	19	47522601	2600	1	7.33E-08	26	1		
DMR19:47796201	19	47796201	400	1	3.93E-07	2	0.5		
DMR19:48538701	19	48538701	600	1	4.62E-08	1	0.166666667		
DMR19:48572501	19	48572501	600	1	2.14E-08	2	0.333333333	AABR07043929.1	
DMR19:48943801	19	48943801	1000	2	1.55E-07	8	0.8		
DMR19:49016101	19	49016101	2400	1	2.77E-07	37	1.541666667	Dynlrb2	Cytoskeleton
DMR19:49054601	19	49054601	6600	1	3.91E-07	61	0.924242424		
DMR19:49122001	19	49122001	1500	1	1.01E-07	28	1.866666667	Cdyl2	Metabolism
DMR19:49261901	19	49261901	1400	1	1.66E-07	8	0.571428571		
DMR19:49282601	19	49282601	300	1	8.64E-07	1	0.333333333		
DMR19:49393501	19	49393501	1500	1	2.18E-07	22	1.466666667	Cenpn	Cell Cycle
DMR19:49837001	19	49837001	400	1	3.91E-07	7	1.75	Cmip	Signaling
DMR19:50052101	19	50052101	1000	2	5.86E-10	11	1.1	Plcg2	Signaling
DMR19:50186201	19	50186201	3100	1	8.03E-07	38	1.225806452		
DMR19:50429901	19	50429901	2700	2	6.81E-08	37	1.37037037		
DMR19:50561401	19	50561401	700	1	9.56E-08	6	0.857142857		
DMR19:51000301	19	51000301	900	2	7.74E-13	11	1.222222222	Cdh13	Extracellular Matrix
DMR19:51453301	19	51453301	600	3	2.85E-12	2	0.333333333	Cdh13	Extracellular Matrix
DMR19:51729701	19	51729701	300	1	9.20E-07	2	0.666666667	Cdh13	Extracellular Matrix
DMR19:51835601	19	51835601	2300	2	1.39E-09	21	0.913043478	Cdh13	Extracellular Matrix
DMR19:52054401	19	52054401	2100	4	2.54E-08	6	0.285714286	Mlycd;AABR07044001.4;AABR07044001.2;AABR07044001.3	

DMR19:52294701	19	52294701	2400	2	3.11E-08	33	1.375		
DMR19:52378101	19	52378101	800	3	1.07E-08	6	0.75	Atp2c2	Transport
DMR19:52420901	19	52420901	3400	1	6.57E-09	50	1.470588235	Tldc1	Unknown
DMR19:52491701	19	52491701	1300	3	1.32E-10	2	0.153846154	Cotl1	Cytoskeleton
DMR19:53232701	19	53232701	2200	1	2.60E-07	37	1.681818182		
DMR19:53850601	19	53850601	3200	2	6.15E-09	35	1.09375	AABR07044049.1	
DMR19:53891801	19	53891801	4400	2	2.59E-08	59	1.340909091	AABR07044049.1	
DMR19:54053501	19	54053501	2900	1	3.65E-07	31	1.068965517	Gse1	Transcription
DMR19:54348701	19	54348701	2200	1	4.15E-07	23	1.045454545		
DMR19:54528201	19	54528201	1200	2	1.12E-08	5	0.416666667	SNORA72;AABR07044063.2	
DMR19:56045301	19	56045301	1900	2	2.55E-08	32	1.684210526	Spata2L;Vps9d1;Zfp276	Development;Transcription
DMR19:56227001	19	56227001	2500	1	1.17E-07	69	2.76	Rn60_19_0563.2;Tubb3;Rn60_19_0563.3	
DMR19:56452001	19	56452001	1000	1	7.36E-07	14	1.4	AABR07044080.2	
DMR19:56899201	19	56899201	3500	1	2.85E-08	75	2.142857143		
DMR19:57292501	19	57292501	2600	1	3.65E-07	42	1.615384615	Cog2	
DMR19:57325501	19	57325501	1500	1	4.49E-08	11	0.733333333	Cog2;Agt	Proteolysis
DMR19:57371601	19	57371601	3000	2	2.43E-07	41	1.366666667	Capn9	Protease
DMR19:58101901	19	58101901	1100	1	1.89E-07	3	0.272727273		
DMR19:58415001	19	58415001	1100	3	7.93E-10	11	1	AABR07044111.1;Set	Receptor
DMR19:58457101	19	58457101	3800	1	6.24E-07	37	0.973684211		
DMR19:58703001	19	58703001	2200	1	3.09E-07	62	2.818181818	Pcnx2	
DMR19:59018901	19	59018901	4200	1	4.60E-08	57	1.357142857		
DMR19:59674101	19	59674101	2400	2	2.94E-08	23	0.958333333		
DMR19:59685601	19	59685601	2400	1	8.51E-08	15	0.625	AABR07072667.1	

DMR19:59728101	19	59728101	2900	2	1.52E-08	29	1		
DMR19:60273101	19	60273101	1100	2	4.72E-09	6	0.545454545	Pard3	Cell Junction
DMR19:60617901	19	60617901	1500	1	2.53E-07	8	0.533333333		
DMR19:60652601	19	60652601	300	1	2.58E-10	1	0.333333333		
DMR19:61194001	19	61194001	700	1	4.21E-09	3	0.428571429		
DMR19:61514501	19	61514501	400	2	5.61E-08	1	0.25	AABR07044173.1	
DMR20:1819401	20	1819401	300	2	6.00E-09	3	1	Olr1739;RT1-M3-1;Olr1740-ps;Olr1741-ps	Immune
DMR20:1906901	20	1906901	2100	1	7.52E-07	17	0.80952381	Ubd;Rn50_20_0040.5;Olr1747-ps	Transcription
DMR20:2558201	20	2558201	900	1	8.61E-08	5	0.555555556	7SK	
DMR20:3053901	20	3053901	200	1	2.74E-07	1	0.5		
DMR20:3071401	20	3071401	600	1	3.12E-08	2	0.333333333	Rn60_20_0031.1	
DMR20:4664501	20	4664501	400	1	1.01E-07	6	1.5	RT1-CE1;Rn60_20_0047.3;RT1-CE4;RT1-CE6	Immune
DMR20:5597101	20	5597101	3700	1	1.85E-08	43	1.162162162	Bak1	Signaling
DMR20:5655801	20	5655801	2300	1	5.86E-08	44	1.913043478	Itpr3	Signaling
DMR20:6496201	20	6496201	1500	1	6.15E-08	29	1.933333333	Cpne5	Development
DMR20:8045001	20	8045001	1300	1	2.82E-11	13	1		
DMR20:8073101	20	8073101	1600	1	2.56E-09	7	0.4375		
DMR20:8080801	20	8080801	900	1	9.58E-07	9	1		
DMR20:8408601	20	8408601	1100	1	5.62E-07	12	1.090909091		
DMR20:9180101	20	9180101	4900	1	1.14E-07	78	1.591836735	Btbd9;AABR07044520.1	Unknown
DMR20:9316201	20	9316201	1300	3	5.41E-09	7	0.538461538	Dnah8	Cytoskeleton
DMR20:9339801	20	9339801	1700	1	1.16E-07	18	1.058823529	Dnah8	Cytoskeleton
DMR20:9350001	20	9350001	1100	3	7.27E-11	15	1.363636364	Dnah8	Cytoskeleton

DMR20:9576201	20	9576201	400	2	4.21E-10	1	0.25	Glp1r	Receptor
DMR20:9659501	20	9659501	2700	3	1.07E-07	26	0.962962963	Umodl1	
DMR20:10122901	20	10122901	600	2	3.53E-08	12	2	Pde9a	Signaling
DMR20:10280601	20	10280601	4300	1	9.35E-07	59	1.372093023	Ndufv3;SNORA36	Metabolism
DMR20:10705101	20	10705101	4500	1	6.43E-07	105	2.333333333		
DMR20:12403301	20	12403301	2600	1	1.99E-09	26	1		
DMR20:12517001	20	12517001	1300	1	7.35E-08	1	0.076923077	Pcbp3	Translation
DMR20:12754901	20	12754901	1400	3	2.62E-12	18	1.285714286		
DMR20:13377501	20	13377501	2500	2	7.72E-09	30	1.2		
DMR20:13446801	20	13446801	1700	4	6.83E-10	14	0.823529412	Slc5a4	Transport
DMR20:13524501	20	13524501	600	2	3.31E-09	0	0	Slc5a4b	
DMR20:14171001	20	14171001	1600	1	1.65E-07	27	1.6875	7SK;Upb1	Metabolism
DMR20:14401501	20	14401501	2400	1	3.54E-07	23	0.958333333	Specc1l	
DMR20:14569801	20	14569801	1700	1	3.56E-08	18	1.058823529	Rab36;Rtdr1	Signaling
DMR20:14846901	20	14846901	500	1	4.57E-11	1	0.2		
DMR20:15052501	20	15052501	4000	1	1.27E-07	45	1.125	Pcdh15	Extracellular Matrix
DMR20:15103801	20	15103801	1200	2	4.08E-11	11	0.916666667	Pcdh15	Extracellular Matrix
DMR20:15287201	20	15287201	300	1	2.99E-07	0	0	Pcdh15	Extracellular Matrix
DMR20:15437901	20	15437901	1000	2	3.02E-09	3	0.3		
DMR20:15781301	20	15781301	800	1	2.83E-07	5	0.625		
DMR20:15797901	20	15797901	1600	1	1.55E-10	15	0.9375		
DMR20:16022601	20	16022601	300	1	1.67E-07	7	2.333333333		
DMR20:16673401	20	16673401	1900	1	1.15E-08	12	0.631578947		
DMR20:18229001	20	18229001	300	1	5.57E-07	0	0		
DMR20:18369901	20	18369901	2100	2	1.63E-08	27	1.285714286		
DMR20:18749701	20	18749701	2900	1	2.83E-07	39	1.344827586		

DMR20:19484001	20	19484001	1300	3	9.21E-08	17	1.307692308	Fam13c	
DMR20:19855601	20	19855601	4100	1	2.02E-08	64	1.56097561	AABR07044765.1	
DMR20:19918501	20	19918501	700	1	1.45E-07	7	1		
DMR20:20365101	20	20365101	1600	2	2.83E-07	17	1.0625	Ank3	Cytoskeleton
DMR20:20502201	20	20502201	1900	1	9.72E-08	26	1.368421053		
DMR20:20610901	20	20610901	600	3	5.15E-11	1	0.166666667		
DMR20:20871401	20	20871401	5600	4	2.15E-08	69	1.232142857		
DMR20:20981401	20	20981401	300	1	1.39E-07	0	0	AABR07044799.1	
DMR20:20995901	20	20995901	500	1	2.28E-08	4	0.8		
DMR20:21099701	20	21099701	200	1	7.50E-07	0	0		
DMR20:21184101	20	21184101	800	2	2.42E-08	7	0.875	AABR07044804.1	
DMR20:21510001	20	21510001	1400	1	6.12E-07	14	1		
DMR20:21761501	20	21761501	700	1	3.11E-07	7	1	Arid5b	Transcription
DMR20:22138701	20	22138701	600	2	4.65E-09	0	0		
DMR20:22365601	20	22365601	1600	1	3.11E-07	5	0.3125		
DMR20:22858901	20	22858901	500	2	1.64E-11	4	0.8	Jmjd1c	Epigenetic
DMR20:23391301	20	23391301	1500	8	3.45E-09	3	0.2		
DMR20:24887901	20	24887901	1100	1	4.41E-07	5	0.454545455		
DMR20:25393701	20	25393701	400	1	1.54E-08	1	0.25	Ctnna3	Cytoskeleton
DMR20:25442301	20	25442301	300	1	1.87E-07	1	0.333333333	Ctnna3	Cytoskeleton
DMR20:25584101	20	25584101	800	1	4.44E-07	12	1.5	Ctnna3	Cytoskeleton
DMR20:25661401	20	25661401	300	1	5.09E-07	1	0.333333333	Ctnna3	Cytoskeleton
DMR20:26048801	20	26048801	900	1	1.71E-07	3	0.333333333	Lrrtm3	Receptor
DMR20:26588101	20	26588101	700	2	1.80E-08	2	0.285714286	Ctnna3	Cytoskeleton
DMR20:27157901	20	27157901	2800	2	2.04E-10	19	0.678571429	Hnrnph3	Translation
DMR20:28381201	20	28381201	1100	1	8.75E-07	8	0.727272727	AABR07044962.1	
DMR20:29845601	20	29845601	1400	1	6.49E-07	20	1.428571429	Psap	Signaling
DMR20:30266701	20	30266701	1400	1	5.98E-08	26	1.857142857	AABR07044980.1	
DMR20:30521901	20	30521901	1300	1	8.92E-08	23	1.769230769	AABR07044988.2	

DMR20:30842601	20	30842601	500	2	7.91E-08	0	0	Adamts14	Protease
DMR20:31140501	20	31140501	800	1	7.37E-07	6	0.75	Lrrc20	Development
DMR20:31255501	20	31255501	1300	1	1.51E-08	6	0.461538462	Npffr1;Ppa1	Receptor;Metabolism
DMR20:31703801	20	31703801	800	2	1.75E-12	0	0	AABR07045015.1	
DMR20:32350701	20	32350701	2600	3	1.46E-09	47	1.807692308	Stox1;U6	
DMR20:32653801	20	32653801	600	1	1.77E-08	1	0.166666667		
DMR20:32812901	20	32812901	400	1	3.59E-11	0	0		
DMR20:33506801	20	33506801	400	1	2.20E-08	7	1.75	Gopc;AABR07045071.1	Transport
DMR20:34612801	20	34612801	3200	5	9.22E-13	15	0.46875	Cep85l	Epigenetic
DMR20:35817401	20	35817401	300	1	9.55E-07	0	0		
DMR20:37459501	20	37459501	2500	1	5.41E-08	35	1.4	Tbc1d32	Unknown
DMR20:38304901	20	38304901	500	2	1.84E-09	0	0		
DMR20:39194201	20	39194201	2900	1	2.56E-07	39	1.344827586		
DMR20:40144201	20	40144201	1600	1	7.68E-07	11	0.6875		
DMR20:41256001	20	41256001	200	1	2.19E-07	0	0		
DMR20:41495201	20	41495201	300	1	3.60E-07	3	1		
DMR20:42050701	20	42050701	400	1	9.44E-10	1	0.25		
DMR20:42363601	20	42363601	100	1	4.43E-07	1	1		
DMR20:43847601	20	43847601	400	1	1.77E-08	0	0		
DMR20:44324301	20	44324301	2600	2	1.92E-13	23	0.884615385		
DMR20:44786901	20	44786901	1200	2	9.77E-16	7	0.583333333		
DMR20:45048501	20	45048501	2500	1	6.27E-08	24	0.96	RGD1561777	Metabolism
DMR20:45271301	20	45271301	1600	1	8.04E-07	18	1.125		
DMR20:45313401	20	45313401	3300	1	3.28E-07	57	1.727272727	Rpf2	Transcription
DMR20:45620201	20	45620201	1300	1	6.56E-09	15	1.153846154		
DMR20:45742101	20	45742101	2600	1	1.71E-07	25	0.961538462		
DMR20:45925901	20	45925901	2700	1	2.35E-14	47	1.740740741	Fig4	Signaling

DMR20:45966501	20	45966501	1800	2	3.79E-07	23	1.277777778	Fig4;AABR07045391.1	Signaling
DMR20:46367301	20	46367301	1000	1	2.46E-07	12	1.2		
DMR20:46815901	20	46815901	500	1	6.65E-09	3	0.6	Armc2	Transcription
DMR20:46930101	20	46930101	900	2	8.44E-09	11	1.222222222	AABR07045416.1	
DMR20:46936001	20	46936001	1400	1	3.92E-07	19	1.357142857	AABR07045416.1	
DMR20:47309801	20	47309801	2300	1	7.28E-08	27	1.173913043	Nr2e1	Receptor
DMR20:47621801	20	47621801	300	2	7.18E-09	5	1.666666667	Scml4	Transcription
DMR20:47884401	20	47884401	2100	1	1.04E-07	40	1.904761905	Sobp	
DMR20:48055901	20	48055901	1300	2	1.09E-08	20	1.538461538	Pdss2	Metabolism
DMR20:48143901	20	48143901	3500	1	1.15E-07	28	0.8	Pdss2	Metabolism
DMR20:48278101	20	48278101	1800	1	2.34E-07	40	2.222222222	Bend3;LOC683897	
DMR20:48415301	20	48415301	600	1	2.63E-07	3	0.5		
DMR20:48624101	20	48624101	2800	1	9.46E-07	37	1.321428571	Mettl24	Epigenetic
DMR20:49692701	20	49692701	1100	1	5.85E-07	3	0.272727273		
DMR20:50136801	20	50136801	1600	1	7.13E-07	25	1.5625		
DMR20:50604601	20	50604601	1100	1	6.11E-07	5	0.454545455		
DMR20:50696901	20	50696901	1300	1	3.70E-08	16	1.230769231	Hace1	Translation
DMR20:51124701	20	51124701	700	1	3.07E-07	9	1.285714286		
DMR20:52153801	20	52153801	200	1	4.12E-07	1	0.5		
DMR20:53168701	20	53168701	2500	1	9.21E-07	30	1.2		
DMR20:53748501	20	53748501	400	1	1.04E-08	1	0.25		
DMR20:55421301	20	55421301	900	1	4.04E-07	16	1.777777778	Ascc3	Signaling

DMRMT:1	MT	1	13000	17	3.45E-11	245	1.884615385	AY172581.13;AY172581.9;AY172581.3;AY172581.24;AY172581.14;Mt-nd1;AY172581.4;AY172581.21;AY172581.15;Mt-nd2;AY172581.6;AY172581.22;AY172581.18;AY172581.10;AY172581.7;Mt-co1;AY172581.19;AY172581.12;Mt-co2;AY172581.1;Mt-atp8;Mt-atp6;Mt-cox3;AY172581.5;Mt-nd3;AY172581.16;Mt-nd4l;Mt-nd4;AY172581.23;AY172581.17;AY172581.11;Mt-nd5;Mt-nd6;AY172581.20;Mt-cyb;AY172581.8;AY172581.2	

DMRMT:14001	MT	14001	2300	6	5.87E-12	47	2.043478261	Mt-nd2;AY172581.6;AY172581.22;AY172581.18;AY172581.10;AY172581.7;Mt-co1;AY172581.19;AY172581.12;Mt-co2;AY172581.1;Mt-atp8;Mt-atp6;Mt-cox3;AY172581.5;Mt-nd3;AY172581.16;Mt-nd4l;Mt-nd4;AY172581.23;AY172581.17;AY172581.11;Mt-nd5;Mt-nd6;AY172581.20;Mt-cyb;AY172581.8;AY172581.2	
DMRX:136801	X	136801	800	1	2.07E-07	12	1.5		
DMRX:235801	X	235801	1100	1	5.19E-07	10	0.909090909		
DMRX:321301	X	321301	400	1	2.08E-08	1	0.25		
DMRX:341701	X	341701	2100	2	3.06E-08	11	0.523809524		
DMRX:713701	X	713701	1400	1	4.95E-08	5	0.357142857	Ssx2	
DMRX:2200001	X	2200001	6200	1	1.59E-07	32	0.516129032		
DMRX:2339801	X	2339801	900	1	2.25E-08	9	1		
DMRX:2341701	X	2341701	8800	1	5.94E-09	46	0.522727273		
DMRX:2431901	X	2431901	600	1	1.00E-07	4	0.666666667	Slc9a7	Metabolism

DMRX:3025301	X	3025301	3500	1	8.13E-07	35	1		
DMRX:3592901	X	3592901	300	1	8.52E-07	0	0		
DMRX:3604801	X	3604801	1100	2	4.95E-07	12	1.090909091		
DMRX:4339001	X	4339001	2200	1	7.50E-08	20	0.909090909		
DMRX:4979601	X	4979601	1100	1	2.43E-10	2	0.181818182		
DMRX:6213501	X	6213501	4700	1	5.04E-09	38	0.808510638		
DMRX:7887101	X	7887101	600	1	3.39E-10	5	0.833333333		
DMRX:8037801	X	8037801	400	1	1.70E-07	0	0		
DMRX:9185801	X	9185801	1300	1	3.11E-07	6	0.461538462		
DMRX:10177201	X	10177201	900	1	8.19E-07	3	0.333333333		
DMRX:11231201	X	11231201	700	1	1.89E-07	3	0.428571429		
DMRX:11589401	X	11589401	1500	1	2.61E-07	17	1.133333333		
DMRX:14252001	X	14252001	4100	1	2.38E-08	64	1.56097561		
DMRX:14287201	X	14287201	2200	1	2.07E-07	36	1.636363636	Rpgr	Signaling
DMRX:14951401	X	14951401	4200	4	2.33E-09	21	0.5	SNORA26;Slc38a5	Unknown
DMRX:15665501	X	15665501	400	1	4.08E-07	0	0	Magix	
DMRX:18032201	X	18032201	1500	2	5.92E-09	14	0.933333333		
DMRX:18460601	X	18460601	1600	2	8.29E-08	6	0.375		
DMRX:20754801	X	20754801	3100	1	4.22E-07	31	1	FAM120C;Fam104b	
DMRX:21137001	X	21137001	1000	3	1.22E-09	3	0.3	Phf8	Cell Cycle
DMRX:23822401	X	23822401	1900	1	1.94E-10	22	1.157894737		
DMRX:25009701	X	25009701	500	2	1.86E-09	5	1	Clcn4	Metabolism
DMRX:28947201	X	28947201	300	1	6.95E-08	1	0.333333333	AABR07037715.1	
DMRX:29772001	X	29772001	1100	1	7.13E-07	19	1.727272727		
DMRX:30244601	X	30244601	6000	1	2.11E-08	62	1.033333333		
DMRX:30968701	X	30968701	400	2	3.89E-11	2	0.5		
DMRX:31504201	X	31504201	900	1	3.11E-11	5	0.555555556		
DMRX:32496801	X	32496801	2000	1	7.43E-08	7	0.35	RGD1565844	
DMRX:36223301	X	36223301	200	2	1.67E-42	1	0.5		

DMRX:37825701	X	37825701	300	1	8.52E-07	0	0	Sh3kbp1	Signaling
DMRX:43715301	X	43715301	100	1	1.37E-07	0	0		
DMRX:44318101	X	44318101	100	1	6.00E-09	0	0		
DMRX:44325301	X	44325301	600	1	3.87E-08	3	0.5		
DMRX:44431201	X	44431201	400	1	5.44E-10	1	0.25	SNORA32	
DMRX:45111001	X	45111001	1700	3	1.02E-09	17	1	Prkx	Signaling
DMRX:45916201	X	45916201	4800	2	4.01E-07	46	0.958333333		
DMRX:48189301	X	48189301	300	1	3.45E-07	0	0	Da2-19	
DMRX:48197101	X	48197101	400	2	1.46E-08	0	0	Da2-19	
DMRX:50268301	X	50268301	1100	1	2.11E-07	4	0.363636364		
DMRX:52246101	X	52246101	500	1	3.58E-07	0	0	Dmd	Development
DMRX:53662801	X	53662801	1800	1	8.86E-07	8	0.444444444		
DMRX:59018401	X	59018401	500	1	1.18E-08	1	0.2		
DMRX:60499801	X	60499801	2100	1	1.56E-07	6	0.285714286		
DMRX:61151601	X	61151601	1000	2	2.57E-08	9	0.9	Mageb4	
DMRX:61742601	X	61742601	400	1	9.93E-07	0	0		
DMRX:64322501	X	64322501	400	1	1.36E-07	1	0.25	Arhgef9	Signaling
DMRX:64498601	X	64498601	3500	1	9.20E-07	31	0.885714286		
DMRX:68876701	X	68876701	800	1	4.15E-07	3	0.375	Rn50_X_0698.2	
DMRX:72153501	X	72153501	300	1	4.42E-07	0	0	Hdac8	Epigenetic
DMRX:74546401	X	74546401	300	1	4.15E-09	4	1.333333333		
DMRX:76368601	X	76368601	300	1	1.87E-08	0	0		
DMRX:77294701	X	77294701	400	1	4.21E-09	31	7.75	Rn60_X_0773.5;Taf9b	Translation
DMRX:77604801	X	77604801	400	1	4.04E-07	3	0.75	Rn60_X_0775.4	
DMRX:78081001	X	78081001	300	1	2.98E-07	1	0.333333333		
DMRX:79056601	X	79056601	500	1	1.99E-07	8	1.6	RGD1561552	
DMRX:79062501	X	79062501	100	1	1.01E-07	0	0	RGD1561552	
DMRX:81167501	X	81167501	100	1	1.26E-08	0	0		

DMRX:81181501	X	81181501	100	1	3.20E-08	0	0		
DMRX:82127501	X	82127501	400	1	4.28E-07	1	0.25		
DMRX:85600301	X	85600301	200	1	5.93E-07	1	0.5		
DMRX:87298401	X	87298401	2500	2	2.56E-09	28	1.12		
DMRX:87313601	X	87313601	6800	1	2.60E-07	67	0.985294118		
DMRX:89108101	X	89108101	1200	1	1.78E-07	5	0.416666667		
DMRX:92425801	X	92425801	3800	1	1.67E-07	42	1.105263158		
DMRX:95322501	X	95322501	700	1	9.29E-07	1	0.142857143		
DMRX:96277401	X	96277401	300	1	4.53E-07	6	2		
DMRX:96631201	X	96631201	800	1	3.79E-09	2	0.25		
DMRX:97233601	X	97233601	300	1	1.07E-09	0	0		
DMRX:97730901	X	97730901	300	1	1.36E-08	1	0.333333333		
DMRX:101036401	X	1.01E+08	6000	1	2.30E-08	70	1.166666667		
DMRX:101974101	X	1.02E+08	500	1	2.79E-07	4	0.8		
DMRX:102605201	X	1.03E+08	1100	1	1.36E-07	6	0.545454545		
DMRX:102957401	X	1.03E+08	600	1	5.16E-09	5	0.833333333		
DMRX:103938101	X	1.04E+08	1100	1	1.14E-07	6	0.545454545		
DMRX:105446401	X	1.05E+08	700	2	2.01E-07	0	0		
DMRX:106215701	X	1.06E+08	200	1	8.53E-07	1	0.5	LOC100361139	
DMRX:106499201	X	1.06E+08	600	4	7.10E-14	1	0.166666667	Arxes2	Signaling
DMRX:107964101	X	1.08E+08	2200	1	2.42E-07	23	1.045454545		
DMRX:108074401	X	1.08E+08	2400	2	6.47E-11	31	1.291666667		
DMRX:108826201	X	1.09E+08	700	1	3.74E-08	9	1.285714286	Il1rapl2	Receptor
DMRX:109827001	X	1.1E+08	400	2	1.48E-08	0	0	Il1rapl2	Receptor
DMRX:110512501	X	1.11E+08	1600	2	1.43E-07	19	1.1875	Trap1a	
DMRX:111113801	X	1.11E+08	400	1	6.66E-08	2	0.5	Cldn2	Cytoskeleton
DMRX:112383901	X	1.12E+08	1000	2	4.81E-07	11	1.1	Atg4a	Metabolism
DMRX:112386501	X	1.12E+08	8100	6	1.44E-10	57	0.703703704	Atg4a	Metabolism
DMRX:112802701	X	1.13E+08	1900	1	4.01E-08	18	0.947368421	Col4a5	

DMRX:113139301	X	1.13E+08	500	1	2.36E-07	2	0.4		
DMRX:114532801	X	1.15E+08	700	1	6.74E-07	1	0.142857143		
DMRX:114861601	X	1.15E+08	1300	1	2.85E-07	11	0.846153846		
DMRX:115188401	X	1.15E+08	2700	2	4.24E-08	14	0.518518519		
DMRX:117301201	X	1.17E+08	3600	1	7.12E-07	20	0.555555556	AABR07041019.1	
DMRX:117536101	X	1.18E+08	2200	1	5.30E-08	6	0.272727273		
DMRX:119978401	X	1.2E+08	14700	1	2.10E-07	164	1.115646259		
DMRX:120620701	X	1.21E+08	500	1	2.99E-08	1	0.2	Slc6a14	Transport
DMRX:121249101	X	1.21E+08	4100	2	2.33E-07	33	0.804878049		
DMRX:122366201	X	1.22E+08	1800	1	3.86E-09	14	0.777777778		
DMRX:125146601	X	1.25E+08	600	4	5.25E-11	3	0.5		
DMRX:125605401	X	1.26E+08	500	2	4.57E-07	2	0.4		
DMRX:125772201	X	1.26E+08	300	2	1.64E-08	1	0.333333333		
DMRX:131186401	X	1.31E+08	600	1	2.35E-08	0	0		
DMRX:133342801	X	1.33E+08	400	1	3.85E-07	1	0.25		
DMRX:133728301	X	1.34E+08	100	1	1.40E-21	0	0		
DMRX:136411501	X	1.36E+08	400	1	1.16E-07	3	0.75		
DMRX:136953001	X	1.37E+08	600	1	2.32E-11	1	0.166666667		
DMRX:137908301	X	1.38E+08	500	1	4.68E-10	2	0.4		
DMRX:138559401	X	1.39E+08	3100	1	1.92E-09	22	0.709677419		
DMRX:138970301	X	1.39E+08	300	1	3.78E-07	6	2	Hs6st2	Golgi
DMRX:139482101	X	1.39E+08	300	1	1.31E-12	1	0.333333333	AABR07041778.3;AABR07041778.1;Ftl1l1;AABR07041778.2	Unknown
DMRX:142171601	X	1.42E+08	400	1	1.65E-07	2	0.5	Fgf13	Growth Factors & Cytokines
DMRX:142947401	X	1.43E+08	500	1	2.58E-07	4	0.8		
DMRX:144392601	X	1.44E+08	300	1	7.50E-09	8	2.666666667	U6	

DMRX:144794501	X	1.45E+08	400	1	4.93E-09	1	0.25		
DMRX:149094301	X	1.49E+08	1700	1	2.86E-09	12	0.705882353		
DMRX:150232801	X	1.5E+08	1100	1	4.26E-08	5	0.454545455		
DMRX:150464501	X	1.5E+08	400	1	2.11E-08	1	0.25		
DMRX:150542101	X	1.51E+08	1900	3	6.60E-09	8	0.421052632		
DMRX:150615801	X	1.51E+08	1300	1	2.51E-08	7	0.538461538		
DMRX:150618101	X	1.51E+08	1000	1	1.18E-08	6	0.6		
DMRX:150803101	X	1.51E+08	1700	1	3.60E-07	7	0.411764706		
DMRX:151484401	X	1.51E+08	200	2	9.40E-26	1	0.5		
DMRX:152628301	X	1.53E+08	700	2	1.08E-07	0	0	Gabra3	Receptor
DMRX:156934201	X	1.57E+08	300	1	6.59E-07	1	0.333333333	L1cam	Extracellular Matrix
DMRX:157003601	X	1.57E+08	400	1	1.39E-09	0	0	Idh3g;Srpk3;Ssr4	Metabolism;Signaling
DMRX:157220201	X	1.57E+08	300	1	3.53E-07	3	1		
DMRX:157226401	X	1.57E+08	700	2	2.46E-08	1	0.142857143		
DMRX:157437901	X	1.57E+08	1400	1	2.32E-08	9	0.642857143		
DMRX:157886801	X	1.58E+08	400	1	2.42E-07	1	0.25	Fam122b	
DMRX:158383601	X	1.58E+08	1300	1	3.71E-08	6	0.461538462	LOC100909732	
DMRX:159678401	X	1.6E+08	1200	2	1.29E-09	7	0.583333333		
DMRY:638201	Y	638201	100	1	1.41E-07	2	2	Rn60_Y_0007.3;Rn60_Y_0007.4	
DMRY:1122701	Y	1122701	1900	1	8.07E-08	17	0.894736842	Rn60_Y_0010.2	
DMRY:2016301	Y	2016301	2600	1	7.24E-07	25	0.961538462	LOC103694562;AC241722.2;AC241722.1	
DMRY:2234801	Y	2234801	3200	1	8.31E-08	26	0.8125		
DMRY:2377801	Y	2377801	900	4	1.06E-08	2	0.222222222		
