# Supplementary material for: Alterations in sperm DNA methylation, non-coding RNA and histone retention associate with DDT-induced epigenetic transgenerational inheritance of disease
Source: Epigenetics Chromatin. 2018 Feb 27;11:8. doi: 10.1186/s13072-018-0178-0 (PMC5827984; doi:10.1186/s13072-018-0178-0)
Supplement: Supplementary file 5 — Additional file 5: Table S3. F3 DMR p < 1e−06. [file 13072_2018_178_MOESM5_ESM.pdf]

Supplemental Table S3
F3 DMR p<1e-06

DMR Name	Chr	Start	(bp) Length	# SigWin	minP	CpG #	CpG Density	Gene Association	Gene Category
DMR1:2680401	1	2680401	200	1	2.41E-11	1	0.5		
DMR1:8994501	1	8994501	200	1	1.79E-18	0	0		
DMR1:18825001	1	18825001	100	1	3.57E-07	0	0	Lama2	Extracellular Matrix
DMR1:19704301	1	19704301	100	1	9.75E-11	1	1		
DMR1:24657501	1	24657501	300	1	1.32E-08	2	0.666667		
DMR1:29563601	1	29563601	300	1	5.24E-09	2	0.666667		
DMR1:30251701	1	30251701	100	1	1.34E-15	0	0		
DMR1:31939801	1	31939801	1300	2	3.68E-07	12	0.923077	Zdhhc11;AABR07001001.1;Brd9	Unknown;Transcription
DMR1:32666501	1	32666501	3900	1	7.39E-07	24	0.615385	AABR07001006.1	
DMR1:34004301	1	34004301	100	1	1.93E-12	0	0	AABR07001019.1	
DMR1:49105801	1	49105801	200	2	1.76E-10	1	0.5		
DMR1:56584701	1	56584701	100	1	5.35E-13	0	0		
DMR1:62806601	1	62806601	200	2	1.06E-34	0	0	LOC682419	
DMR1:65137301	1	65137301	200	1	1.70E-07	0	0	Vom2r80	Receptor
DMR1:66738001	1	66738001	200	2	8.19E-14	0	0		
DMR1:70975501	1	70975501	1300	1	1.95E-14	18	1.384615		
DMR1:71011601	1	71011601	100	1	9.27E-14	0	0		
DMR1:71016801	1	71016801	200	1	4.48E-15	0	0		
DMR1:71711401	1	71711401	100	1	1.53E-13	0	0	Nlrp4	Immune
DMR1:71835101	1	71835101	1000	1	9.49E-08	12	1.2	Vom1r32	
DMR1:74542201	1	74542201	200	2	4.64E-28	0	0		
DMR1:74984501	1	74984501	200	1	9.35E-07	0	0	Vom2r31	
DMR1:75026201	1	75026201	1100	1	4.08E-08	7	0.636364		
DMR1:79353601	1	79353601	600	3	2.05E-11	1	0.166667	LOC102557244	
DMR1:83210101	1	83210101	1000	3	1.20E-11	6	0.6	Cyp2b3	
DMR1:83767701	1	83767701	100	1	1.94E-15	0	0	Cyp2a1	
DMR1:85782001	1	85782001	800	2	4.81E-17	5	0.625		
DMR1:95742801	1	95742801	1300	1	5.25E-09	13	1		
DMR1:100104501	1	100104501	1500	1	5.09E-07	42	2.8	Klk1	Metabolism
DMR1:103733501	1	103733501	1500	1	4.47E-07	4	0.266667	Mrgprx2	Receptor
DMR1:104484001	1	104484001	100	1	8.54E-09	0	0		
DMR1:105241601	1	105241601	100	1	4.48E-10	0	0		
DMR1:112360501	1	112360501	200	1	3.72E-09	0	0	Luzp2;Gabrg3	Receptor
DMR1:112723001	1	112723001	4000	1	6.77E-10	35	0.875	Luzp2;Gabrg3	Receptor
DMR1:116691701	1	116691701	200	2	1.14E-12	2	1	SNORD115	
DMR1:117459401	1	117459401	100	1	2.45E-15	2	2		
DMR1:120634801	1	120634801	200	1	5.28E-07	2	1		
DMR1:120642501	1	120642501	200	1	3.34E-09	2	1		
DMR1:120872401	1	120872401	100	1	1.10E-12	0	0	AABR07003933.1	
DMR1:121303401	1	121303401	300	1	1.26E-07	1	0.333333		
DMR1:121363601	1	121363601	100	1	2.89E-11	0	0		
DMR1:122052201	1	122052201	100	1	3.04E-11	0	0		
DMR1:124852701	1	124852701	200	1	6.24E-07	1	0.5		
DMR1:131292101	1	131292101	100	1	1.97E-08	0	0		
DMR1:132179001	1	132179001	700	1	6.22E-14	7	1		
DMR1:132905901	1	132905901	200	2	6.56E-17	0	0		
DMR1:134631301	1	134631301	1500	1	1.46E-07	29	1.933333		

DMR1:150011801	1	150011801	500	1	5.34E-07	4	0.8		
DMR1:151997401	1	151997401	1200	1	4.77E-14	4	0.333333	SNORA43;SNORA17	
DMR1:157694001	1	157694001	900	1	5.67E-07	7	0.777778	Ddias;SNORA70;Prcp	Proteolysis
DMR1:157869201	1	157869201	200	1	9.21E-07	2	1		
DMR1:158454801	1	158454801	300	1	7.95E-07	2	0.666667		
DMR1:158677301	1	158677301	200	1	1.20E-08	0	0		
DMR1:158705201	1	158705201	200	1	2.75E-07	0	0		
DMR1:158839101	1	158839101	5100	1	9.46E-08	34	0.666667		
DMR1:158845401	1	158845401	2200	3	4.20E-09	7	0.318182		
DMR1:159566101	1	159566101	1500	2	7.23E-10	12	0.8		
DMR1:165487201	1	165487201	1000	2	5.06E-14	3	0.3	C2cd3;Ucp3	Electron Transport
DMR1:169836601	1	169836601	1600	1	1.41E-07	23	1.4375	Olr179	
DMR1:169916801	1	169916801	200	1	6.37E-07	3	1.5	Olr185;Olr186	
DMR1:171897401	1	171897401	2100	1	1.94E-08	19	0.904762	Ppfibp2	Receptor
DMR1:176384801	1	176384801	100	1	1.31E-15	0	0	Galnt18	Unknown
DMR1:179152401	1	179152401	200	1	1.11E-18	0	0		
DMR1:179669401	1	179669401	100	1	4.06E-12	0	0		
DMR1:179746901	1	179746901	1100	1	1.03E-09	6	0.545455		
DMR1:179922201	1	179922201	800	1	6.00E-09	6	0.75		
DMR1:183276401	1	183276401	100	1	1.20E-11	0	0		
DMR1:183457601	1	183457601	100	1	1.02E-09	0	0		
DMR1:185457001	1	185457001	300	1	8.48E-10	3	1	Plekha7	Signaling
DMR1:187119401	1	187119401	200	2	3.02E-16	0	0		
DMR1:190678501	1	190678501	400	1	3.10E-11	12	3	LOC102547219;AABR07005633.1;Vwa3a	Unknown
DMR1:191223201	1	191223201	200	1	7.27E-07	2	1		
DMR1:192438201	1	192438201	1900	1	1.26E-07	32	1.684211	Prkcb	Signaling
DMR1:198809101	1	198809101	400	1	2.72E-07	3	0.75	AABR07005779.2	
DMR1:204403901	1	204403901	700	2	5.78E-37	1	0.142857		
DMR1:205624901	1	205624901	1700	2	5.32E-11	28	1.647059	Tex36	
DMR1:205664601	1	205664601	200	2	1.09E-10	1	0.5	Tex36	
DMR1:213567401	1	213567401	200	2	6.93E-15	1	0.5	RGD1309350	
DMR1:218109901	1	218109901	100	1	2.62E-21	0	0	Ccnd1	Cell Cycle
DMR1:219322701	1	219322701	1900	2	1.24E-12	17	0.894737	Cabp2	Signaling
DMR1:224030301	1	224030301	1100	1	7.81E-12	8	0.727273		
DMR1:241917401	1	241917401	100	1	9.36E-15	0	0		
DMR1:242334801	1	242334801	1100	1	5.28E-07	21	1.909091	Pip5k1b	Signaling
DMR1:252346101	1	252346101	800	1	2.13E-12	9	1.125	Lipk	
DMR1:257325601	1	257325601	200	2	1.50E-11	0	0	Plce1	Signaling
DMR1:258581201	1	258581201	100	1	1.46E-07	0	0		
DMR1:258702701	1	258702701	100	1	5.95E-09	0	0	Cyp2c12	
DMR1:268757301	1	268757301	100	1	2.40E-07	0	0		
DMR1:270616001	1	270616001	500	1	1.99E-07	8	1.6		
DMR1:271006701	1	271006701	1000	2	1.85E-08	2	0.2		
DMR1:278450301	1	278450301	700	1	6.96E-07	2	0.285714		
DMR1:278725801	1	278725801	1700	1	4.26E-08	7	0.411765	Atrnl1	Signaling
DMR2:4247101	2	4247101	2100	1	4.59E-08	14	0.666667	RGD1560883	Unknown
DMR2:5685601	2	5685601	5600	2	7.42E-14	44	0.785714		
DMR2:5692401	2	5692401	2500	3	5.69E-08	16	0.64		
DMR2:5738701	2	5738701	600	3	8.58E-09	18	3		

DMR2:6079101	2	6079101	8400	1	3.60E-07	52	0.619048		
DMR2:6090901	2	6090901	1900	4	5.40E-09	12	0.631579		
DMR2:6210501	2	6210501	700	2	4.09E-07	7	1		
DMR2:6575501	2	6575501	1600	2	5.81E-07	1	0.0625		
DMR2:7298901	2	7298901	400	1	5.60E-07	4	1		
DMR2:9568201	2	9568201	1700	1	5.21E-12	16	0.941176	Polr3g;Mblac2	Transcription;Metabolism
DMR2:11916701	2	11916701	1100	1	1.30E-18	9	0.818182		
DMR2:12540301	2	12540301	300	1	3.82E-09	2	0.666667	Tmem161b	Unknown
DMR2:12659701	2	12659701	200	2	2.23E-09	2	1		
DMR2:15338701	2	15338701	1100	1	6.14E-07	5	0.454545		
DMR2:15642201	2	15642201	700	2	2.00E-07	4	0.571429		
DMR2:16162601	2	16162601	1100	1	7.39E-07	6	0.545455		
DMR2:20611301	2	20611301	1100	2	4.48E-15	8	0.727273	Ssbp2	Transcription
DMR2:20677401	2	20677401	200	1	2.00E-07	1	0.5	Ssbp2	Transcription
DMR2:35843101	2	35843101	100	1	1.44E-15	0	0	Rnf180	
DMR2:38513801	2	38513801	900	1	1.85E-09	7	0.777778		
DMR2:41283701	2	41283701	2100	2	1.12E-19	15	0.714286	Pde4d	Metabolism
DMR2:42410201	2	42410201	600	1	1.14E-07	6	1		
DMR2:47687301	2	47687301	100	1	3.34E-09	0	0		
DMR2:50438601	2	50438601	400	2	3.46E-11	2	0.5	Hcn1	Signaling
DMR2:51788401	2	51788401	100	1	5.44E-10	0	0		
DMR2:54673101	2	54673101	1300	1	2.70E-10	15	1.153846	Mroh2b	Development
DMR2:55017001	2	55017001	100	1	1.05E-08	0	0		
DMR2:56428001	2	56428001	900	2	2.66E-10	16	1.777778	Lifr	Receptor
DMR2:58323501	2	58323501	600	1	7.61E-10	3	0.5		
DMR2:63601201	2	63601201	700	1	4.48E-10	1	0.142857		
DMR2:64058801	2	64058801	200	2	9.19E-12	0	0		
DMR2:65476201	2	65476201	200	2	2.45E-15	0	0		
DMR2:67827201	2	67827201	100	1	1.05E-08	0	0		
DMR2:69282601	2	69282601	300	1	5.52E-07	3	1		
DMR2:76983501	2	76983501	2000	1	2.50E-07	13	0.65		
DMR2:77025701	2	77025701	100	1	6.69E-07	0	0		
DMR2:85419601	2	85419601	100	1	3.62E-09	0	0	Sema5a	Signaling
DMR2:95743801	2	95743801	100	1	8.19E-11	0	0		
DMR2:97566501	2	97566501	200	2	4.29E-14	0	0		
DMR2:98691301	2	98691301	800	1	1.55E-09	5	0.625		
DMR2:101572001	2	101572001	1800	1	4.84E-07	12	0.666667		
DMR2:104672301	2	104672301	100	1	4.00E-18	0	0		
DMR2:112398901	2	112398901	200	1	8.48E-10	0	0	Spata16	
DMR2:115810301	2	115810301	200	2	8.19E-14	0	0	U1	
DMR2:116961301	2	116961301	100	1	4.04E-16	0	0	Egfem1	
DMR2:123613601	2	123613601	1000	1	1.95E-12	7	0.7	RGD1307100	
DMR2:124014201	2	124014201	500	1	1.14E-07	3	0.6		
DMR2:129802101	2	129802101	100	1	2.51E-07	0	0		
DMR2:132212301	2	132212301	200	1	2.86E-19	1	0.5		
DMR2:138100001	2	138100001	5000	1	6.06E-07	50	1		
DMR2:142368701	2	142368701	1000	2	1.92E-10	14	1.4	Lhfp	Transcription
DMR2:143719401	2	143719401	100	1	2.35E-14	0	0		
DMR2:146672101	2	146672101	1800	2	3.03E-07	14	0.777778		
DMR2:149052901	2	149052901	300	1	6.19E-27	1	0.333333	Clrn1	
DMR2:151153901	2	151153901	100	1	4.80E-21	0	0		
DMR2:155157401	2	155157401	200	1	7.28E-16	1	0.5		

DMR2:157456101	2	157456101	100	1	1.34E-08	0	0	AABR07011085.1; Lekr1	
DMR2:158551001	2	158551001	200	2	3.27E-09	1	0.5		
DMR2:158692401	2	158692401	900	2	1.50E-14	9	1		
DMR2:159304001	2	159304001	100	1	2.00E-07	0	0		
DMR2:161895201	2	161895201	100	1	3.62E-09	0	0		
DMR2:162262201	2	162262201	200	1	4.80E-12	0	0		
DMR2:162971801	2	162971801	1900	1	6.25E-07	11	0.578947		
DMR2:163261401	2	163261401	100	1	6.23E-12	0	0		
DMR2:163594501	2	163594501	100	1	4.38E-14	0	0		
DMR2:163875401	2	163875401	200	2	5.61E-16	0	0		
DMR2:166198301	2	166198301	100	1	1.46E-11	0	0	Ppm1l	Signaling
DMR2:170227501	2	170227501	100	1	2.25E-14	0	0	Si	Metabolism
DMR2:174036501	2	174036501	200	2	1.14E-18	0	0	Serpini1	Proteolysis
DMR2:175546701	2	175546701	200	1	9.35E-07	1	0.5		
DMR2:179758801	2	179758801	200	1	6.69E-07	0	0		
DMR2:184947001	2	184947001	1600	1	4.76E-07	16	1	Fam160a1	
DMR2:190711901	2	190711901	400	3	1.94E-27	0	0		
DMR2:192974001	2	192974001	1900	1	1.50E-10	5	0.263158	AABR07012314.1	
DMR2:201599401	2	201599401	200	1	3.10E-09	3	1.5		
DMR2:202949701	2	202949701	6900	1	9.08E-07	78	1.130435	Man1a2	Metabolism
DMR2:203934801	2	203934801	300	1	6.04E-11	0	0		
DMR2:204423701	2	204423701	100	1	2.75E-07	1	1	Nhlh2	Transcription
DMR2:210658001	2	210658001	600	1	5.81E-07	3	0.5	Eps8l3	Unknown
DMR2:210660801	2	210660801	400	3	7.85E-09	3	0.75	Eps8l3	Unknown
DMR2:215215701	2	215215701	100	1	8.19E-11	0	0		
DMR2:217650601	2	217650601	700	1	4.69E-07	2	0.285714		
DMR2:218148601	2	218148601	200	1	1.50E-11	0	0		
DMR2:220014101	2	220014101	200	2	2.32E-22	0	0	Plppr5	
DMR2:221028701	2	221028701	200	2	9.60E-15	0	0	Snx7	Signaling
DMR2:221680201	2	221680201	400	2	2.38E-11	6	1.5		
DMR2:224665201	2	224665201	1600	2	2.49E-19	22	1.375		
DMR2:225975101	2	225975101	2100	2	1.85E-19	14	0.666667		
DMR2:226194001	2	226194001	500	2	2.40E-07	4	0.8		
DMR2:229593901	2	229593901	100	1	3.17E-10	0	0		
DMR2:229740201	2	229740201	2800	1	2.62E-08	24	0.857143		
DMR2:236336401	2	236336401	1000	1	6.91E-07	20	2	Lef1	Transcription
DMR2:241894301	2	241894301	200	2	3.48E-08	0	0		
DMR2:245163501	2	245163501	1000	1	2.88E-07	23	2.3		
DMR2:245261301	2	245261301	200	2	2.11E-12	0	0		
DMR2:252862801	2	252862801	1400	1	1.08E-08	23	1.642857	AABR07013718.1	
DMR2:254597801	2	254597801	400	1	2.26E-11	6	1.5		
DMR2:254608301	2	254608301	1300	1	4.52E-14	19	1.461538		
DMR2:256967701	2	256967701	200	1	3.59E-17	0	0	Ifi44l	Immune
DMR2:262770901	2	262770901	800	1	2.68E-11	8	1		
DMR2:265633401	2	265633401	2100	1	2.27E-07	8	0.380952		
DMR3:1678101	3	1678101	200	1	2.15E-24	0	0	AABR07051219.1	
DMR3:2223001	3	2223001	100	1	1.62E-16	0	0	Pnpla7	Metabolism
DMR3:14486201	3	14486201	800	2	1.37E-14	12	1.5	Gsn	Cytoskeleton
DMR3:15940501	3	15940501	100	1	8.75E-17	0	0	Olr401	
DMR3:23694001	3	23694001	900	7	5.75E-16	2	0.222222		
DMR3:24675401	3	24675401	100	1	1.10E-12	0	0		
DMR3:26027501	3	26027501	500	1	8.50E-09	2	0.4	Lrp1b	Metabolism

DMR3:26753801	3	26753801	200	1	9.77E-09	0	0		
DMR3:27540201	3	27540201	200	2	1.10E-12	0	0		
DMR3:28256201	3	28256201	1700	3	4.25E-08	27	1.588235		
DMR3:30344601	3	30344601	500	1	1.51E-07	5	1		
DMR3:38282401	3	38282401	200	2	1.95E-11	0	0	Stam2;AABR07052130.1	Transport
DMR3:44482801	3	44482801	1200	2	1.20E-16	16	1.333333	Acvr1	Receptor
DMR3:46587201	3	46587201	200	2	1.57E-13	3	1.5	Pla2r1	Receptor
DMR3:48759401	3	48759401	100	1	3.62E-15	0	0	Kcnh7	Metabolism
DMR3:49379801	3	49379801	900	1	2.32E-11	2	0.222222		
DMR3:49388401	3	49388401	200	2	3.17E-10	0	0		
DMR3:50456101	3	50456101	100	1	1.94E-10	1	1		
DMR3:56955901	3	56955901	400	1	2.26E-18	0	0		
DMR3:57763101	3	57763101	200	2	1.10E-11	0	0	AC107446.2	
DMR3:60031901	3	60031901	3500	1	3.02E-08	43	1.228571	Cir1;Scrn3	Unknown
DMR3:63947901	3	63947901	900	1	2.46E-13	13	1.444444	Ccdc141	Cytoskeleton
DMR3:64142101	3	64142101	6800	1	8.39E-07	89	1.308824	AABR07052587.3	
DMR3:67008501	3	67008501	800	1	1.83E-10	3	0.375		
DMR3:67165101	3	67165101	900	2	7.28E-16	3	0.333333		
DMR3:69438401	3	69438401	200	2	1.34E-08	2	1		
DMR3:69442401	3	69442401	100	1	2.00E-07	0	0		
DMR3:69870101	3	69870101	200	2	2.05E-11	1	0.5		
DMR3:69965101	3	69965101	200	1	7.68E-11	0	0		
DMR3:70315901	3	70315901	3200	1	3.58E-07	20	0.625	Fsip2	
DMR3:71002201	3	71002201	1800	2	1.70E-13	26	1.444444	AABR07052729.1	
DMR3:73258201	3	73258201	1100	1	7.41E-08	9	0.818182	Olr464;Olr465	Receptor
DMR3:74009901	3	74009901	100	1	6.63E-16	0	0	Olr516	
DMR3:74550701	3	74550701	700	1	1.85E-08	15	2.142857	Olr531	
DMR3:76511401	3	76511401	200	2	4.47E-15	0	0	Olr623	Receptor
DMR3:82614001	3	82614001	400	1	3.22E-07	6	1.5	Ext2	Metabolism
DMR3:85064201	3	85064201	100	1	4.70E-14	0	0		
DMR3:89022701	3	89022701	200	2	3.62E-15	1	0.5		
DMR3:89364501	3	89364501	100	1	2.24E-16	0	0		
DMR3:89656701	3	89656701	700	1	1.86E-08	4	0.571429		
DMR3:89957001	3	89957001	800	2	3.34E-09	2	0.25		
DMR3:90005501	3	90005501	100	1	4.61E-10	0	0		
DMR3:91381101	3	91381101	900	2	4.27E-15	9	1		
DMR3:96229101	3	96229101	100	1	9.65E-09	1	1		
DMR3:96670401	3	96670401	1900	1	3.37E-07	24	1.263158		
DMR3:96904101	3	96904101	200	1	4.22E-13	0	0		
DMR3:96987501	3	96987501	100	1	9.75E-11	0	0		
DMR3:97726201	3	97726201	100	1	7.32E-16	0	0	Mpped2	Metabolism
DMR3:101708501	3	101708501	100	1	3.24E-07	0	0		
DMR3:101756101	3	101756101	800	2	1.08E-08	4	0.5		
DMR3:106538301	3	106538301	100	1	1.12E-07	0	0		
DMR3:118193901	3	118193901	1600	2	5.49E-14	18	1.125	Galk2	Signaling
DMR3:118247501	3	118247501	400	3	4.36E-11	2	0.5	Galk2	Signaling
DMR3:118772801	3	118772801	900	1	1.30E-10	7	0.777778	Atp8b4	Transport
DMR3:119031001	3	119031001	1000	2	4.87E-10	28	2.8	Slc27a2	
DMR3:119353801	3	119353801	2100	1	5.42E-07	24	1.142857	LOC100363112;Sppl2a	Signaling
DMR3:127889201	3	127889201	200	2	8.43E-08	1	0.5		
DMR3:137785301	3	137785301	100	1	4.32E-07	0	0	Pcsk2	Proteolysis

DMR3:140718401	3	140718401	1000	1	1.57E-13	13	1.3	Ralgapa2	Signaling
DMR3:149895601	3	149895601	300	2	7.05E-23	1	0.333333	Snta1	Cytoskeleton
DMR3:155931501	3	155931501	500	1	1.12E-07	5	1		
DMR3:161073801	3	161073801	3000	2	2.64E-10	29	0.966667	Wfdc8;Wfdc6b	Signaling
DMR3:162495501	3	162495501	200	1	1.14E-07	1	0.5	Zmynd8	Signaling
DMR3:169140201	3	169140201	100	1	2.89E-10	1	1		
DMR3:170676901	3	170676901	1700	1	1.67E-08	17	1		
DMR3:170978101	3	170978101	800	1	2.34E-07	8	1		
DMR4:741401	4	741401	200	2	4.00E-16	1	0.5	AABR07059002.1	
DMR4:1574901	4	1574901	200	2	1.78E-09	1	0.5	Olr1243;Olr1244	
DMR4:2813901	4	2813901	800	1	3.00E-13	4	0.5		
DMR4:9606801	4	9606801	200	2	5.71E-10	0	0	Reln	Protease
DMR4:13586801	4	13586801	200	2	1.82E-11	0	0		
DMR4:15328901	4	15328901	100	1	5.58E-11	0	0		
DMR4:15874001	4	15874001	1100	2	2.88E-13	12	1.090909	Cacna2d1	Metabolism
DMR4:17007901	4	17007901	200	2	1.89E-15	1	0.5	Pclo	Extracellular Matrix
DMR4:19547901	4	19547901	1000	1	1.70E-13	7	0.7		
DMR4:32721301	4	32721301	500	1	1.63E-08	5	1		
DMR4:32878601	4	32878601	200	2	1.77E-10	0	0		
DMR4:32883901	4	32883901	100	1	2.76E-14	0	0		
DMR4:33775901	4	33775901	1200	2	3.02E-16	3	0.25		
DMR4:36132701	4	36132701	200	1	1.06E-09	1	0.5		
DMR4:36418901	4	36418901	100	1	6.21E-13	1	1		
DMR4:37407401	4	37407401	200	1	3.85E-17	0	0		
DMR4:37544501	4	37544501	100	1	4.79E-13	0	0		
DMR4:37577401	4	37577401	100	1	8.75E-17	0	0		
DMR4:43110301	4	43110301	5400	1	6.55E-07	53	0.981481		
DMR4:45960401	4	45960401	200	1	2.87E-16	0	0		
DMR4:47331401	4	47331401	100	1	1.36E-08	0	0	AABR07059974.1	
DMR4:49089401	4	49089401	200	1	6.69E-07	4	2	Cped1	
DMR4:49517801	4	49517801	1200	1	4.29E-07	11	0.916667		
DMR4:50092101	4	50092101	1200	2	1.57E-13	11	0.916667	Ptprz1	Signaling
DMR4:50841501	4	50841501	100	1	1.08E-09	0	0	Cadps2	Metabolism
DMR4:51768301	4	51768301	100	1	1.03E-16	0	0		
DMR4:54948601	4	54948601	200	2	3.00E-13	0	0	Grm8	Receptor
DMR4:66247801	4	66247801	400	1	7.60E-07	4	1		
DMR4:70271501	4	70271501	100	1	4.32E-07	1	1	Chl1	Extracellular Matrix
DMR4:71948401	4	71948401	900	1	4.60E-21	1	0.111111	Olr804	
DMR4:74063701	4	74063701	400	1	1.28E-07	11	2.75		
DMR4:75952301	4	75952301	200	1	2.93E-08	0	0	Cntnap2	Receptor
DMR4:78409401	4	78409401	300	1	3.46E-16	6	2		
DMR4:83191401	4	83191401	1100	1	1.04E-07	13	1.181818	AABR07060593.2	
DMR4:84063201	4	84063201	900	1	3.59E-17	11	1.222222	Cpvl	Protease
DMR4:87753901	4	87753901	200	1	8.79E-17	0	0	Vom1r73	Receptor
DMR4:87755701	4	87755701	600	1	3.56E-14	1	0.166667	Vom1r73	Receptor
DMR4:87960901	4	87960901	900	1	9.24E-07	4	0.444444		
DMR4:88825201	4	88825201	600	1	9.65E-09	7	1.166667	Abcg2	Receptor
DMR4:96343801	4	96343801	100	1	1.67E-09	0	0		
DMR4:102001601	4	102001601	100	1	9.49E-07	0	0		
DMR4:105996401	4	105996401	1200	2	1.93E-12	2	0.166667		
DMR4:108417001	4	108417001	700	1	3.62E-15	11	1.571429		
DMR4:109180701	4	109180701	700	1	3.12E-13	5	0.714286		
DMR4:110006401	4	110006401	600	1	8.03E-17	1	0.166667		

DMR4:111021901	4	111021901	900	1	2.17E-12	5	0.555556	Lrrtm4	Receptor
DMR4:119181001	4	119181001	900	4	8.31E-10	1	0.111111	Gkn2;Gkn3	
DMR4:121455701	4	121455701	200	1	1.09E-15	0	0	Chchd6	Transcription
DMR4:124948601	4	124948601	100	1	6.75E-13	1	1		
DMR4:127463301	4	127463301	200	2	6.64E-21	0	0		
DMR4:127984401	4	127984401	100	1	3.62E-09	0	0		
DMR4:128371401	4	128371401	200	2	1.01E-19	0	0		
DMR4:128555401	4	128555401	100	1	9.65E-09	0	0		
DMR4:131521201	4	131521201	1100	1	1.76E-10	7	0.636364	Foxp1	Transcription
DMR4:134842401	4	134842401	200	2	2.89E-10	0	0	Cntn3	Cytoskeleton
DMR4:137470001	4	137470001	100	1	1.20E-11	0	0		
DMR4:141515201	4	141515201	100	1	9.49E-07	0	0		
DMR4:142272301	4	142272301	100	1	1.06E-10	1	1		
DMR4:154514201	4	154514201	2600	1	8.67E-07	28	1.076923	Mug1	Immune
DMR4:156844601	4	156844601	200	1	5.95E-09	3	1.5	RGD1307916	
DMR4:158858601	4	158858601	200	1	6.20E-09	1	0.5		
DMR4:162294301	4	162294301	100	1	1.28E-20	0	0	Clec2d	Development
DMR4:162679501	4	162679501	200	2	2.25E-14	0	0	Klra5	Immune
DMR4:163366501	4	163366501	200	1	3.96E-16	0	0	Klrd1	Receptor
DMR4:165314401	4	165314401	200	2	1.76E-10	0	0	Klra5	Immune
DMR4:165596001	4	165596001	100	1	4.61E-10	1	1		
DMR4:166511701	4	166511701	100	1	1.94E-08	0	0		
DMR4:170513201	4	170513201	1700	1	4.88E-09	14	0.823529	Atf7ip	
DMR4:176551701	4	176551701	900	2	3.67E-10	4	0.444444		
DMR5:432401	5	432401	600	1	2.38E-17	4	0.666667		
DMR5:4950701	5	4950701	200	1	9.01E-10	0	0	Xkr9	Unknown
DMR5:21294701	5	21294701	1300	1	1.30E-13	21	1.615385	Car8	Metabolism
DMR5:22222601	5	22222601	200	1	7.68E-11	1	0.5		
DMR5:23237101	5	23237101	100	1	6.34E-19	0	0		
DMR5:28242601	5	28242601	500	2	2.81E-11	2	0.4	Lrrc69	
DMR5:32638601	5	32638601	500	1	1.05E-12	5	1	Cnbd1	
DMR5:33308101	5	33308101	2500	1	7.88E-07	6	0.24	Cngb3	Receptor
DMR5:35445301	5	35445301	2400	1	2.33E-07	23	0.958333		
DMR5:35473501	5	35473501	600	1	2.85E-07	4	0.666667		
DMR5:36441901	5	36441901	200	1	5.19E-08	1	0.5	AABR07047528.1	
DMR5:36846601	5	36846601	500	1	1.40E-10	6	1.2		
DMR5:39989201	5	39989201	200	1	8.14E-07	3	1.5		
DMR5:42314801	5	42314801	100	1	3.44E-11	0	0		
DMR5:42444301	5	42444301	200	2	1.56E-12	0	0		
DMR5:43209801	5	43209801	100	1	5.43E-07	0	0		
DMR5:51485801	5	51485801	100	1	3.25E-08	0	0		
DMR5:53076701	5	53076701	100	1	5.43E-07	0	0		
DMR5:56042101	5	56042101	500	1	4.72E-17	2	0.4		
DMR5:57412401	5	57412401	800	1	9.35E-07	10	1.25		
DMR5:70163201	5	70163201	1000	2	4.55E-11	8	0.8		
DMR5:70822501	5	70822501	100	1	1.94E-08	0	0	SNORA17	
DMR5:71142801	5	71142801	100	1	1.53E-13	0	0		
DMR5:71149301	5	71149301	200	2	8.66E-13	0	0		
DMR5:72973101	5	72973101	200	1	4.52E-08	0	0		
DMR5:77667701	5	77667701	100	1	8.16E-09	0	0	LOC100912565;Rn50_5_0814.4	Immune
DMR5:86979201	5	86979201	100	1	7.43E-11	0	0		
DMR5:88994301	5	88994301	100	1	3.62E-09	0	0		

DMR5:91582801	5	91582801	3000	2	1.37E-17	9	0.3		
DMR5:93048301	5	93048301	100	1	6.87E-07	0	0	Ptprd	Signaling
DMR5:96179701	5	96179701	200	2	5.44E-10	1	0.5		
DMR5:97393801	5	97393801	1400	1	1.77E-07	5	0.357143		
DMR5:97709001	5	97709001	400	1	1.08E-08	4	1		
DMR5:98080101	5	98080101	200	2	7.32E-16	0	0		
DMR5:99070401	5	99070401	200	1	1.91E-09	1	0.5	Pramef5;RGD1306186	EST
DMR5:101771701	5	101771701	6000	20	6.91E-22	61	1.016667	Ccdc171	
DMR5:103390801	5	103390801	200	2	5.40E-11	5	2.5		
DMR5:103558701	5	103558701	600	1	1.89E-08	2	0.333333	Sh3gl2	Signaling
DMR5:108451001	5	108451001	100	1	8.43E-08	0	0		
DMR5:108778901	5	108778901	100	1	3.48E-08	0	0		
DMR5:110874501	5	110874501	2700	2	3.57E-10	20	0.740741		
DMR5:111750401	5	111750401	200	2	2.15E-24	2	1		
DMR5:118559301	5	118559301	200	1	1.39E-14	0	0	Itgb3bp;Efcab7;U6	Receptor;Metabolism
DMR5:119539001	5	119539001	1300	1	2.11E-07	9	0.692308	Cyp2j4	Electron Transport
DMR5:131632001	5	131632001	900	1	2.68E-07	7	0.777778		
DMR5:131938601	5	131938601	200	2	5.73E-13	0	0	Skint1	
DMR5:132006001	5	132006001	200	1	1.85E-09	1	0.5	Skint4	
DMR5:133888201	5	133888201	1200	1	5.74E-13	18	1.5	Tal1;AABR07049701.1;Pdzk1ip1	Transcription
DMR5:137111801	5	137111801	1000	1	1.00E-06	18	1.8	Ptprf	Signaling
DMR5:157172901	5	157172901	1200	1	3.75E-16	15	1.25	Ubxn10;Pla2g2c	Metabolism
DMR5:164305601	5	164305601	700	2	8.19E-14	3	0.428571	U6;LOC500584	
DMR5:166901401	5	166901401	900	2	3.79E-23	18	2	AABR07050484.1	
DMR5:173233801	5	173233801	200	2	6.02E-11	0	0	Tmem88b;Ankrd65	Unknown
DMR6:445401	6	445401	1100	2	7.81E-12	15	1.363636		
DMR6:1203901	6	1203901	100	1	1.76E-10	0	0	Vit	Extracellular Matrix
DMR6:5101401	6	5101401	3800	1	7.25E-07	31	0.815789		
DMR6:5304201	6	5304201	200	2	8.19E-14	0	0		
DMR6:9956601	6	9956601	600	2	8.50E-09	4	0.666667	Prkce	Binding Protein
DMR6:10502201	6	10502201	2000	1	9.08E-07	31	1.55	Atp6v1e2	Transport
DMR6:16218901	6	16218901	1100	1	4.29E-14	7	0.636364		
DMR6:20638901	6	20638901	1000	1	6.69E-07	3	0.3		
DMR6:21941801	6	21941801	200	2	4.58E-22	0	0	Birc6	Unknown
DMR6:29159701	6	29159701	100	1	2.37E-15	0	0	Atad2b	Metabolism
DMR6:30636301	6	30636301	500	1	1.27E-07	15	3	5_8S_rRNA;AABR07063421.1;AABR07063424.1;LOC257642;AABR07063425.2;AABR07063425.1	
DMR6:32111601	6	32111601	200	2	7.77E-22	0	0		
DMR6:32113301	6	32113301	200	1	1.17E-21	0	0		
DMR6:36853801	6	36853801	400	1	6.55E-07	5	1.25	AABR07063601.2	
DMR6:36864401	6	36864401	1000	2	5.31E-09	9	0.9	AABR07063601.2	
DMR6:36884401	6	36884401	500	1	1.33E-08	7	1.4	Msgn1;AABR07063601.1	
DMR6:41054701	6	41054701	300	1	4.33E-08	2	0.666667		
DMR6:41560401	6	41560401	1100	2	5.09E-12	14	1.272727		

DMR6:47179001	6	47179001	200	2	1.01E-07	1	0.5		
DMR6:65102001	6	65102001	1100	2	3.59E-11	4	0.363636	Stxbp6	Receptor
DMR6:71266701	6	71266701	100	1	3.44E-19	0	0	Prkd1	Signaling
DMR6:72308101	6	72308101	300	1	2.61E-10	5	1.666667		
DMR6:74064401	6	74064401	100	1	1.12E-07	0	0		
DMR6:74289301	6	74289301	100	1	1.94E-10	0	0		
DMR6:78129001	6	78129001	200	2	1.42E-13	1	0.5		
DMR6:81207701	6	81207701	500	1	2.65E-07	4	0.8		
DMR6:84161301	6	84161301	200	1	8.19E-11	0	0		
DMR6:85220001	6	85220001	100	1	1.50E-14	0	0		
DMR6:85229301	6	85229301	100	1	1.94E-15	0	0		
DMR6:85586401	6	85586401	200	2	1.33E-09	1	0.5		
DMR6:87937701	6	87937701	100	1	1.10E-12	0	0		
DMR6:92392201	6	92392201	100	1	2.93E-18	0	0	Sav1	Development
DMR6:97271201	6	97271201	1800	1	6.52E-12	18	1		
DMR6:116141701	6	116141701	2300	2	2.11E-12	15	0.652174		
DMR6:116386901	6	116386901	1100	2	2.11E-12	3	0.272727		
DMR6:116920301	6	116920301	100	1	1.14E-18	0	0		
DMR6:117784701	6	117784701	600	1	1.70E-07	7	1.166667		
DMR6:119579301	6	119579301	100	1	1.95E-14	0	0	Flrt2	Cytoskeleton
DMR6:131161301	6	131161301	200	2	6.99E-24	2	1		
DMR6:138251801	6	138251801	100	1	4.06E-12	1	1	Ighm;AABR07065631.2;AABR07065631.1;AABR07065631.3	
DMR6:138525901	6	138525901	700	1	9.06E-07	10	1.428571	Ighm;AABR07065643.1	
DMR6:139069701	6	139069701	5900	1	1.99E-07	40	0.677966	AABR07065656.5	
DMR6:139870501	6	139870501	500	3	2.20E-13	2	0.4	AABR07065714.1	
DMR6:139913301	6	139913301	1400	1	9.21E-07	6	0.428571	AABR07065714.1	
DMR6:140074501	6	140074501	1800	1	7.31E-08	10	0.555556		
DMR6:141274701	6	141274701	1800	1	1.11E-08	11	0.611111		
DMR6:141799001	6	141799001	1000	1	2.73E-08	2	0.2		
DMR6:142440201	6	142440201	100	1	3.43E-10	0	0		
DMR7:2123901	7	2123901	200	2	1.10E-10	0	0		
DMR7:5233301	7	5233301	200	1	3.85E-18	0	0		
DMR7:5952201	7	5952201	100	1	1.77E-08	0	0		
DMR7:9220901	7	9220901	100	1	8.19E-14	1	1		
DMR7:10203701	7	10203701	2100	1	5.21E-08	24	1.142857	AABR07055805.1	
DMR7:13092501	7	13092501	100	1	1.70E-13	0	0		
DMR7:17924101	7	17924101	100	1	1.03E-10	0	0		
DMR7:19747401	7	19747401	100	1	1.70E-07	0	0		
DMR7:20963801	7	20963801	1200	2	1.14E-23	8	0.666667		
DMR7:21060701	7	21060701	2200	2	2.19E-16	19	0.863636		
DMR7:21699401	7	21699401	100	1	3.57E-07	0	0		
DMR7:23226801	7	23226801	300	3	2.91E-26	0	0	AABR07056390.2;AABR07056390.1	
DMR7:32534301	7	32534301	1500	1	4.61E-10	11	0.733333		
DMR7:37438601	7	37438601	900	1	7.21E-08	9	1		
DMR7:37881201	7	37881201	200	1	1.67E-10	0	0		
DMR7:38497601	7	38497601	1400	1	2.57E-07	12	0.857143		
DMR7:40047801	7	40047801	800	1	1.91E-09	1	0.125		

DMR7:44585701	7	44585701	100	1	1.12E-07	1	1		
DMR7:49924401	7	49924401	100	1	1.08E-09	0	0	Ptprq	Receptor
DMR7:51454301	7	51454301	100	1	2.89E-11	1	1	Ppp1r12a	Signaling
DMR7:52342701	7	52342701	200	1	6.08E-09	0	0	Nav3	Development
DMR7:55897001	7	55897001	100	1	5.95E-09	0	0		
DMR7:56018901	7	56018901	200	2	1.37E-10	1	0.5		
DMR7:57318401	7	57318401	100	1	1.66E-11	0	0	Trhde	Signaling
DMR7:57677501	7	57677501	100	1	1.85E-09	0	0	Trhde	Signaling
DMR7:57719601	7	57719601	800	2	5.74E-08	3	0.375		
DMR7:58233701	7	58233701	2000	1	8.91E-10	20	1		
DMR7:59027401	7	59027401	400	1	9.35E-09	0	0		
DMR7:60869501	7	60869501	700	1	3.00E-11	5	0.714286	Rap1b	Signaling
DMR7:63328701	7	63328701	100	1	6.08E-10	0	0		
DMR7:65600501	7	65600501	1100	1	6.29E-09	10	0.909091		
DMR7:65942801	7	65942801	700	2	5.25E-09	8	1.142857		
DMR7:66474601	7	66474601	100	1	3.43E-10	1	1		
DMR7:67403501	7	67403501	200	1	6.85E-23	0	0		
DMR7:67457501	7	67457501	500	1	2.00E-07	3	0.6		
DMR7:78077701	7	78077701	200	1	1.56E-09	0	0		
DMR7:80214501	7	80214501	200	1	7.46E-07	0	0		
DMR7:83091001	7	83091001	200	1	6.08E-10	0	0		
DMR7:85386401	7	85386401	200	2	3.46E-15	1	0.5		
DMR7:86178501	7	86178501	600	2	1.30E-14	4	0.666667		
DMR7:91152801	7	91152801	100	1	6.18E-13	0	0		
DMR7:91873101	7	91873101	200	1	1.50E-11	0	0	Slc30a8	Transport
DMR7:92303501	7	92303501	100	1	6.68E-08	0	0		
DMR7:92975301	7	92975301	400	1	2.67E-07	3	0.75		
DMR7:97937801	7	97937801	100	1	5.58E-11	0	0	RGD1310852;Zhx1	Unknown;Transcription
DMR7:98223101	7	98223101	200	1	3.63E-08	0	0	Anxa13	Signaling
DMR7:106591001	7	106591001	100	1	1.97E-08	0	0	Efr3a	Development
DMR7:108664501	7	108664501	200	2	2.13E-12	1	0.5	Phf20l1	Epigenetic
DMR7:112506601	7	112506601	100	1	2.00E-07	0	0		
DMR7:116971001	7	116971001	600	1	6.15E-08	4	0.666667	Tsta3;Zfp623	Immune;Transcription
DMR7:125149901	7	125149901	300	2	2.65E-10	7	2.333333		
DMR7:125151401	7	125151401	700	1	7.72E-07	4	0.571429		
DMR7:125153501	7	125153501	300	2	1.08E-07	10	3.333333		
DMR7:130114901	7	130114901	200	2	1.03E-15	0	0	Hdac10;Mapk12;Mapk11	Transcription;Signaling
DMR7:133935601	7	133935601	1000	1	1.04E-07	18	1.8	Pdzrn4	
DMR7:134220201	7	134220201	300	1	2.29E-08	4	1.333333		
DMR8:6786001	8	6786001	200	1	6.68E-08	1	0.5		
DMR8:8266201	8	8266201	600	1	2.31E-07	4	0.666667	Cntn5	Extracellular Matrix
DMR8:10643601	8	10643601	100	1	5.25E-09	0	0		
DMR8:14000501	8	14000501	700	2	9.23E-26	8	1.142857		
DMR8:15247401	8	15247401	800	2	1.10E-12	1	0.125		
DMR8:18568801	8	18568801	400	1	2.51E-07	3	0.75		
DMR8:18798201	8	18798201	200	2	1.66E-11	0	0	Olr1124	
DMR8:19972101	8	19972101	400	1	2.25E-08	0	0	Olr1159	Receptor
DMR8:23410401	8	23410401	1200	2	1.58E-14	11	0.916667	RGD1561444	
DMR8:27331601	8	27331601	1200	2	1.09E-10	6	0.5		
DMR8:28895801	8	28895801	1100	4	2.75E-20	16	1.454545		
DMR8:38407101	8	38407101	300	1	7.39E-07	3	1	LOC100360143	Unknown

DMR8:38480601	8	38480601	100	1	1.12E-07	0	0		
DMR8:39937101	8	39937101	100	1	2.23E-09	0	0		
DMR8:41541001	8	41541001	200	2	2.50E-14	1	0.5		
DMR8:46169201	8	46169201	600	1	9.67E-09	1	0.166667		
DMR8:46530001	8	46530001	1200	1	3.28E-07	18	1.5	Sc5d	Metabolism
DMR8:48915001	8	48915001	200	1	6.42E-07	1	0.5		
DMR8:51885501	8	51885501	1500	2	1.61E-11	35	2.333333		
DMR8:52243301	8	52243301	4800	14	3.48E-36	33	0.6875	AABR07070099.1	
DMR8:54881001	8	54881001	1300	3	8.11E-14	11	0.846154	AC141541.1	
DMR8:60664101	8	60664101	500	3	4.14E-25	11	2.2		
DMR8:69522501	8	69522501	1300	2	2.72E-27	2	0.153846	AABR07070416.2	
DMR8:73804601	8	73804601	6300	20	2.63E-13	185	2.936508	Vps13c	
DMR8:82132201	8	82132201	4200	2	1.61E-07	47	1.119048	Myo5a	Cytoskeleton
DMR8:82175601	8	82175601	2200	1	1.53E-11	34	1.545455	Myo5c	Cytoskeleton
DMR8:82196001	8	82196001	1400	1	1.76E-09	16	1.142857	Myo5c	Cytoskeleton
DMR8:88160801	8	88160801	100	1	5.71E-10	0	0		
DMR8:88525301	8	88525301	200	1	4.58E-09	1	0.5		
DMR8:91766601	8	91766601	3000	2	1.10E-16	19	0.633333		
DMR8:94141801	8	94141801	200	2	2.84E-10	0	0	Dopey1	
DMR8:116426101	8	116426101	600	1	2.25E-08	6	1	Slc38a3;Gnat1	Metabolism;Signaling
DMR8:120315301	8	120315301	1600	2	1.77E-10	23	1.4375	Arpp21	
DMR8:125606201	8	125606201	200	2	3.26E-23	0	0	Rbms3	Epigenetic
DMR8:125607601	8	125607601	2400	1	2.51E-07	32	1.333333	Rbms3	Epigenetic
DMR8:126642801	8	126642801	200	2	1.44E-21	2	1	7SK	
DMR8:131240901	8	131240901	200	2	6.18E-10	2	1		
DMR9:6805901	9	6805901	100	1	2.00E-07	0	0		
DMR9:7292801	9	7292801	100	1	7.43E-11	1	1		
DMR9:7555001	9	7555001	200	1	6.29E-09	1	0.5		
DMR9:8457601	9	8457601	6400	1	9.70E-07	43	0.671875		
DMR9:11491601	9	11491601	200	1	1.21E-12	0	0		
DMR9:16677301	9	16677301	900	1	5.74E-08	15	1.666667		
DMR9:17062501	9	17062501	300	1	7.29E-07	6	2	Abcc10;Dlk2	Transport;Extracellular Matrix
DMR9:21131001	9	21131001	1100	2	3.72E-24	10	0.909091	Ptchd4	Unknown
DMR9:21621901	9	21621901	200	1	1.43E-07	0	0		
DMR9:23094501	9	23094501	700	1	2.82E-08	5	0.714286		
DMR9:24256201	9	24256201	200	2	1.01E-07	1	0.5		
DMR9:25376101	9	25376101	5400	1	2.05E-09	57	1.055556		
DMR9:26121001	9	26121001	100	1	1.56E-14	2	2		
DMR9:26263601	9	26263601	200	2	6.04E-13	2	1		
DMR9:27880801	9	27880801	100	1	5.54E-07	0	0		
DMR9:28123001	9	28123001	100	1	2.77E-07	0	0		
DMR9:28290801	9	28290801	200	2	9.36E-15	0	0		
DMR9:29471801	9	29471801	400	1	5.93E-18	0	0		
DMR9:31673201	9	31673201	100	1	1.77E-07	0	0	Adgrb3	
DMR9:32202901	9	32202901	200	1	7.81E-12	1	0.5		
DMR9:32920801	9	32920801	100	1	3.72E-25	0	0		
DMR9:36526301	9	36526301	100	1	1.77E-07	1	1		
DMR9:42047101	9	42047101	300	1	4.31E-12	2	0.666667		
DMR9:46994201	9	46994201	200	2	1.43E-17	0	0	Il1r1	Receptor
DMR9:48311401	9	48311401	900	1	1.01E-08	17	1.888889		
DMR9:49985201	9	49985201	200	2	2.42E-25	0	0		
DMR9:51421201	9	51421201	1900	1	1.62E-20	38	2	Gulp1	Development

DMR9:52108401	9	52108401	200	2	4.00E-16	0	0	Col5a2	Extracellular Matrix
DMR9:55919601	9	55919601	700	1	1.63E-08	2	0.285714		
DMR9:63163801	9	63163801	100	1	6.23E-08	0	0		
DMR9:68869601	9	68869601	600	2	6.45E-08	3	0.5	Pard3b	Cell Junction
DMR9:69037201	9	69037201	1200	1	4.59E-07	12	1	Pard3b	Cell Junction
DMR9:72694901	9	72694901	200	1	3.11E-11	1	0.5		
DMR9:73792601	9	73792601	100	1	5.74E-08	0	0	Kansl1l	
DMR9:83750201	9	83750201	2500	2	1.92E-09	19	0.76		
DMR9:87164801	9	87164801	100	1	2.17E-12	0	0		
DMR9:88140301	9	88140301	2100	2	3.31E-09	7	0.333333	Rhbdd1	Protease
DMR9:94753501	9	94753501	200	2	6.15E-11	1	0.5	Inpp5d	Signaling
DMR9:95831601	9	95831601	1800	1	4.31E-07	23	1.277778		
DMR9:98156701	9	98156701	200	1	4.42E-19	1	0.5		
DMR9:99047101	9	99047101	200	1	1.18E-07	3	1.5	AABR07068327.1;Hdac4	Transcription
DMR9:99374201	9	99374201	100	1	4.61E-10	0	0	RGD1564730	
DMR9:101055301	9	101055301	100	1	1.21E-07	0	0		
DMR9:101563201	9	101563201	100	1	1.10E-10	0	0		
DMR9:104065301	9	104065301	100	1	5.58E-11	0	0		
DMR9:107380401	9	107380401	100	1	9.60E-15	1	1		
DMR9:110320601	9	110320601	1500	1	4.73E-15	23	1.533333		
DMR9:118034501	9	118034501	100	1	2.89E-11	2	2		
DMR10:4577001	10	4577001	100	1	3.49E-18	0	0	Zc3h7a;Txndc11	Transcription;Electron Transport
DMR10:5743601	10	5743601	300	1	1.76E-07	1	0.333333		
DMR10:8968801	10	8968801	200	2	5.94E-11	0	0		
DMR10:12872201	10	12872201	1600	1	1.02E-09	13	0.8125	Gm8225	
DMR10:18595001	10	18595001	1000	1	6.42E-07	11	1.1	Kcnip1	Signaling
DMR10:18601001	10	18601001	1000	2	1.99E-15	2	0.2	Kcnip1	Signaling
DMR10:23316201	10	23316201	1300	1	3.18E-07	7	0.538462		
DMR10:23569701	10	23569701	200	2	3.59E-11	1	0.5		
DMR10:39611501	10	39611501	300	3	5.88E-22	0	0	Csf2;Il3	Signaling
DMR10:51784701	10	51784701	200	2	1.31E-15	0	0	Myocd	Transcription
DMR10:60721001	10	60721001	900	1	1.99E-19	4	0.444444	Olr1501	
DMR10:60765801	10	60765801	100	1	7.41E-07	0	0	Olr1504	
DMR10:61418101	10	61418101	400	1	1.01E-07	1	0.25	Ccdc92b;Rn60_10_0615.3	
DMR10:66206601	10	66206601	100	1	2.89E-10	0	0	LOC497963	Metabolism
DMR10:66296001	10	66296001	200	2	1.55E-09	0	0	LOC497963	Metabolism
DMR10:70688401	10	70688401	200	1	1.55E-10	1	0.5	Mmp28;Taf15	Protease;Transcription
DMR10:73491001	10	73491001	2300	1	4.69E-07	15	0.652174		
DMR10:75179801	10	75179801	600	5	1.19E-17	4	0.666667	Epx;Olr1521	Metabolism;Receptor
DMR10:77053601	10	77053601	1300	1	1.57E-10	18	1.384615		
DMR10:78973501	10	78973501	100	1	3.58E-07	0	0		
DMR10:82942901	10	82942901	1200	1	2.85E-07	12	1	Dlx3	Transcription
DMR10:101053401	10	101053401	300	2	5.74E-08	4	1.333333		
DMR10:101055101	10	101055101	3900	1	7.05E-08	53	1.358974		
DMR10:101831501	10	101831501	400	3	1.83E-11	14	3.5	Slc39a11	Metabolism
DMR10:108142101	10	108142101	200	2	6.92E-17	0	0	Cbx2;Cbx8	Transcription
DMR10:110999101	10	110999101	200	2	4.58E-09	1	0.5		
DMR10:111399301	10	111399301	4000	1	1.23E-10	34	0.85	AABR07030977.1	
DMR11:3865101	11	3865101	100	1	3.27E-07	0	0		
DMR11:6090401	11	6090401	2000	2	3.18E-07	13	0.65		

DMR11:10214001	11	10214001	100	1	1.85E-09	1	1		
DMR11:11623401	11	11623401	100	1	3.58E-07	0	0		
DMR11:13202101	11	13202101	200	1	1.10E-16	4	2		
DMR11:21700801	11	21700801	100	1	1.10E-12	1	1		
DMR11:25409801	11	25409801	1000	2	3.43E-10	12	1.2	Adamts5	Proteolysis
DMR11:25859901	11	25859901	700	1	2.61E-19	2	0.285714		
DMR11:32140501	11	32140501	200	2	1.45E-12	2	1		
DMR11:34790601	11	34790601	100	1	4.48E-11	0	0	Dscr3	Transport
DMR11:38704501	11	38704501	4000	1	8.70E-07	35	0.875		
DMR11:41716701	11	41716701	900	2	1.05E-08	1	0.111111		
DMR11:41996801	11	41996801	800	1	5.09E-12	4	0.5		
DMR11:44646001	11	44646001	1100	1	2.75E-20	5	0.454545		
DMR11:44696401	11	44696401	100	1	1.54E-10	0	0	AABR07033925.1	
DMR11:44774501	11	44774501	500	2	2.99E-17	4	0.8		
DMR11:47310201	11	47310201	200	2	2.85E-09	0	0	U6	
DMR11:54521801	11	54521801	1100	5	7.83E-19	10	0.909091	Retnlg	Development
DMR11:54766401	11	54766401	400	1	1.10E-08	6	1.5	Morc1	
DMR11:55735901	11	55735901	500	1	3.70E-09	4	0.8		
DMR11:55868801	11	55868801	200	1	4.01E-07	1	0.5		
DMR11:57350601	11	57350601	200	2	1.17E-18	0	0	Phldb2	
DMR11:61296801	11	61296801	100	1	1.08E-08	0	0	Spice1	
DMR11:65956501	11	65956501	100	1	2.40E-07	0	0	Ndufb4	Metabolism
DMR11:72678801	11	72678801	500	1	3.26E-23	2	0.4		
DMR11:79084601	11	79084601	2100	1	1.53E-13	16	0.761905	AABR07034573.2	
DMR11:80004101	11	80004101	1000	1	3.80E-16	9	0.9		
DMR11:80612701	11	80612701	100	1	1.91E-09	0	0		
DMR11:84811101	11	84811101	1600	1	4.06E-12	13	0.8125		
DMR11:89455701	11	89455701	700	1	6.38E-08	8	1.142857	Prkdc	Signaling
DMR11:90370001	11	90370001	2700	1	3.19E-07	13	0.481481		
DMR11:90448601	11	90448601	200	2	1.95E-15	0	0		
DMR12:1996501	12	1996501	200	2	2.62E-23	1	0.5	Arhgef18;Pex11g	Signaling
DMR12:5206501	12	5206501	100	1	2.86E-09	0	0	Vom2r58	Receptor
DMR12:5322401	12	5322401	200	2	1.10E-12	1	0.5		
DMR12:6853401	12	6853401	800	1	2.54E-17	2	0.25	Alox5ap	Signaling
DMR12:8713601	12	8713601	1400	1	4.92E-08	24	1.714286		
DMR12:14938201	12	14938201	1100	1	1.85E-08	13	1.181818	Sdk1	Unknown
DMR12:15478601	12	15478601	200	2	5.23E-10	0	0		
DMR12:17596201	12	17596201	200	1	1.24E-12	0	0		
DMR12:20658401	12	20658401	2800	2	6.53E-08	25	0.892857	RGD1561730;RGD1560281	Immune
DMR12:24769601	12	24769601	1400	2	1.31E-10	12	0.857143	Cldn4;Wbscr27	Cytoskeleton;Development
DMR12:28626201	12	28626201	100	1	1.01E-07	0	0	Wbscr17	Development
DMR12:35763601	12	35763601	900	1	4.32E-07	10	1.111111		
DMR12:35943701	12	35943701	100	1	1.85E-08	0	0		
DMR12:39639901	12	39639901	100	1	2.65E-16	0	0	Anapc7	Cell Cycle
DMR13:2039301	13	2039301	100	1	1.93E-12	0	0		
DMR13:2988601	13	2988601	1400	1	1.35E-07	13	0.928571		
DMR13:3289301	13	3289301	100	1	1.34E-08	0	0		
DMR13:11104801	13	11104801	100	1	2.11E-12	0	0		
DMR13:12352201	13	12352201	7900	1	7.59E-10	69	0.873418		
DMR13:13750601	13	13750601	400	2	2.78E-07	1	0.25		
DMR13:15295601	13	15295601	100	1	1.03E-10	0	0	LOC304725	

DMR13:15945701	13	15945701	200	1	1.01E-17	1	0.5		
DMR13:17052701	13	17052701	100	1	2.86E-09	1	1		
DMR13:20086301	13	20086301	100	1	1.74E-26	0	0		
DMR13:29881401	13	29881401	1200	1	1.13E-07	12	1		
DMR13:38898101	13	38898101	100	1	3.43E-10	0	0		
DMR13:42699201	13	42699201	400	1	3.03E-07	4	1	Nckap5	
DMR13:46596301	13	46596301	1300	1	3.84E-12	8	0.615385	Thsd7b	Extracellular Matrix
DMR13:50544701	13	50544701	700	1	7.94E-10	14	2	Kiss1;Golt1a	Golgi
DMR13:53324001	13	53324001	1200	2	1.56E-09	18	1.5	Ddx59	Transcription
DMR13:53512601	13	53512601	700	1	6.08E-08	13	1.857143	Zfp281	Transcription
DMR13:57336601	13	57336601	900	2	1.20E-11	1	0.111111	Kcnt2	Transport
DMR13:60824501	13	60824501	1400	1	1.49E-08	15	1.071429	AABR07021213.1	
DMR13:63336301	13	63336301	100	1	2.29E-08	0	0		
DMR13:64897801	13	64897801	200	2	1.76E-10	0	0		
DMR13:65775301	13	65775301	200	2	1.10E-12	0	0		
DMR13:67511701	13	67511701	1000	1	3.78E-08	10	1	AABR07021355.2	
DMR13:69388401	13	69388401	100	1	1.94E-15	0	0	RGD1309104	Unknown
DMR13:72386701	13	72386701	300	2	2.74E-09	2	0.666667		
DMR13:77864901	13	77864901	100	1	2.16E-16	0	0	Tnn;Rn50_13_0828.3	Extracellular Matrix
DMR13:78036501	13	78036501	100	1	2.76E-14	0	0	Rabgap1l	Signaling
DMR13:82825101	13	82825101	1200	1	1.01E-07	11	0.916667		
DMR13:83352701	13	83352701	300	1	9.39E-07	6	2		
DMR13:83852901	13	83852901	100	1	3.72E-09	0	0	Rcsd1	Unknown
DMR13:86911501	13	86911501	200	2	8.15E-22	2	1		
DMR13:97421301	13	97421301	900	2	4.52E-20	11	1.222222		
DMR13:109131101	13	109131101	100	1	1.08E-09	0	0		
DMR14:3849801	14	3849801	1400	1	7.60E-07	14	1	Cdc7	Signaling
DMR14:7996801	14	7996801	200	1	3.77E-12	3	1.5	LOC108352699	
DMR14:9780701	14	9780701	700	1	6.69E-07	11	1.571429	AABR07014328.1	
DMR14:11472601	14	11472601	300	1	5.54E-07	3	1		
DMR14:12647201	14	12647201	900	1	2.85E-07	9	1		
DMR14:15032401	14	15032401	1400	1	6.55E-07	17	1.214286		
DMR14:15967601	14	15967601	400	3	1.40E-21	0	0		
DMR14:16721701	14	16721701	1800	1	1.09E-10	25	1.388889	Shroom3	Cytoskeleton
DMR14:20826901	14	20826901	800	1	7.28E-16	15	1.875	Slc4a4	Transport
DMR14:21818301	14	21818301	200	1	6.69E-07	0	0	Csn3;Fdcsp	Unknown
DMR14:23604401	14	23604401	200	2	1.85E-09	2	1	Stap1;U1;Cenpc	Signaling;Transcription
DMR14:24477901	14	24477901	1100	1	2.40E-07	9	0.818182		
DMR14:26655201	14	26655201	100	1	7.81E-12	0	0	AABR07014690.1;Tecrl	
DMR14:28858401	14	28858401	200	2	1.40E-21	0	0	Adgrl3	
DMR14:44677901	14	44677901	100	1	6.00E-09	0	0	Rfc1	Transcription
DMR14:44752001	14	44752001	200	1	1.81E-07	1	0.5	Wdr19	Metabolism
DMR14:50841801	14	50841801	100	1	7.45E-31	0	0		
DMR14:53777101	14	53777101	100	1	1.50E-11	0	0	AABR07015394.1	
DMR14:54204801	14	54204801	200	2	8.36E-14	0	0		
DMR14:56518201	14	56518201	200	2	3.00E-13	0	0		
DMR14:56866001	14	56866001	200	2	1.47E-13	0	0	U4	
DMR14:62151301	14	62151301	100	1	1.77E-08	1	1	AABR07015531.1	
DMR14:67831601	14	67831601	100	1	3.70E-09	1	1		
DMR14:68108301	14	68108301	100	1	5.35E-13	0	0		
DMR14:68873101	14	68873101	100	1	5.43E-07	0	0	AABR07015654.1	

DMR14:68900601	14	68900601	200	2	1.03E-09	0	0		
DMR14:69289301	14	69289301	1100	2	1.62E-16	3	0.272727		
DMR14:72790201	14	72790201	100	1	1.94E-08	0	0		
DMR14:74266901	14	74266901	100	1	3.25E-08	0	0		
DMR14:75530701	14	75530701	200	1	2.00E-07	0	0		
DMR14:76609101	14	76609101	200	2	5.61E-16	1	0.5		
DMR14:82211601	14	82211601	400	1	2.23E-09	6	1.5		
DMR14:85908101	14	85908101	100	1	6.69E-07	2	2	Rn50_14_0866.4	
DMR14:86405901	14	86405901	200	2	5.55E-17	1	0.5	Npc1l1;U6;LOC103693780	Receptor
DMR14:95627901	14	95627901	200	1	5.74E-13	0	0		
DMR14:97276001	14	97276001	700	1	8.05E-19	1	0.142857		
DMR14:102086901	14	102086901	200	2	4.48E-15	1	0.5		
DMR14:105524701	14	105524701	100	1	1.72E-08	1	1		
DMR14:109890401	14	109890401	200	2	6.40E-09	0	0		
DMR14:114768001	14	114768001	1300	1	8.70E-09	11	0.846154	U6	
DMR15:5225901	15	5225901	100	1	8.19E-14	0	0		
DMR15:6734001	15	6734001	100	1	2.22E-07	0	0		
DMR15:6786401	15	6786401	100	1	4.58E-09	0	0		
DMR15:10521701	15	10521701	500	1	3.00E-11	4	0.8		
DMR15:13395001	15	13395001	200	2	4.43E-13	0	0		
DMR15:13711401	15	13711401	500	1	4.06E-12	4	0.8		
DMR15:16893701	15	16893701	200	2	1.05E-12	1	0.5		
DMR15:23225401	15	23225401	500	1	2.33E-21	2	0.4		
DMR15:25225201	15	25225201	100	1	9.36E-09	0	0		
DMR15:25797901	15	25797901	1600	1	2.51E-10	17	1.0625		
DMR15:28123001	15	28123001	500	1	8.19E-14	13	2.6	LOC103694853	
DMR15:29540001	15	29540001	7600	1	5.86E-07	38	0.5	AABR07017639.1;	
DMR15:31534901	15	31534901	1300	2	6.30E-11	12	0.923077	AABR07017825.7	
DMR15:31869001	15	31869001	100	1	6.27E-08	0	0	RGD1563780	
DMR15:32383701	15	32383701	200	1	1.74E-11	0	0	AABR07017902.1;AABR07017901.1	
DMR15:40212301	15	40212301	1100	2	4.24E-07	2	0.181818	Atp8a2	Transport
DMR15:50643501	15	50643501	100	1	9.36E-09	0	0		
DMR15:60370901	15	60370901	100	1	3.34E-09	0	0		
DMR15:63648501	15	63648501	100	1	3.17E-10	0	0		
DMR15:66820101	15	66820101	400	1	5.84E-08	1	0.25		
DMR15:67561201	15	67561201	200	2	9.07E-11	4	2	Pcdh17	Cytoskeleton
DMR15:72282001	15	72282001	900	1	3.14E-07	4	0.444444		
DMR15:73028401	15	73028401	100	1	2.11E-12	1	1		
DMR15:74880401	15	74880401	1700	2	1.36E-13	11	0.647059		
DMR15:76243401	15	76243401	1000	1	9.65E-09	2	0.2		
DMR15:78236401	15	78236401	200	2	3.75E-16	0	0		
DMR15:78348001	15	78348001	100	1	6.18E-12	0	0		
DMR15:81158101	15	81158101	200	1	1.01E-07	4	2		
DMR15:84543101	15	84543101	200	1	8.21E-15	0	0	Klf12	Transcription
DMR15:85109601	15	85109601	100	1	4.58E-09	0	0		
DMR15:87920601	15	87920601	200	2	4.27E-15	2	1	Mycbp2	Metabolism
DMR15:92361501	15	92361501	200	1	7.06E-13	0	0	Mycbp2	Metabolism
DMR15:95326701	15	95326701	200	2	3.85E-17	1	0.5		
DMR15:97362101	15	97362101	300	3	1.50E-14	0	0		

DMR15:100076301	15	100076301	200	2	2.80E-29	0	0		
DMR15:101361701	15	101361701	300	1	2.78E-07	1	0.333333		
DMR15:103185301	15	103185301	100	1	5.42E-08	0	0		
DMR15:104420801	15	104420801	1800	2	9.74E-11	28	1.555556		
DMR15:106878701	15	106878701	100	1	8.19E-14	0	0		
DMR15:107028701	15	107028701	2700	1	3.24E-07	25	0.925926		
DMR15:109929401	15	109929401	3400	1	2.50E-08	39	1.147059	Nalcn	Transport
DMR16:5881601	16	5881601	900	2	1.25E-08	16	1.777778		
DMR16:5883501	16	5883501	1100	1	1.59E-08	21	1.909091		
DMR16:6824701	16	6824701	100	1	2.51E-10	0	0	Sfmbt1	Transcription
DMR16:10660801	16	10660801	100	1	2.18E-10	0	0	Fam35a;Glud1	Metabolism
DMR16:11128801	16	11128801	1100	1	1.79E-07	17	1.545455		
DMR16:11895701	16	11895701	100	1	2.00E-07	0	0	Grid1	Receptor
DMR16:11980501	16	11980501	100	1	1.36E-08	0	0		
DMR16:12485901	16	12485901	300	1	8.55E-08	1	0.333333		
DMR16:13041801	16	13041801	1000	3	1.77E-07	5	0.5	AABR07024735.1	
DMR16:13049401	16	13049401	1900	3	2.25E-14	17	0.894737	AABR07024735.1	
DMR16:13642901	16	13642901	400	3	2.45E-15	0	0	SNORA17	
DMR16:24143401	16	24143401	4100	1	1.46E-11	55	1.341463		
DMR16:24572101	16	24572101	900	1	3.05E-21	3	0.333333	Naf1	
DMR16:30197901	16	30197901	3400	2	3.26E-08	44	1.294118		
DMR16:30350301	16	30350301	600	1	1.66E-11	6	1		
DMR16:35654501	16	35654501	800	1	6.29E-09	10	1.25	Galntl6	Metabolism
DMR16:41509701	16	41509701	200	2	5.73E-13	0	0		
DMR16:42545301	16	42545301	1100	2	5.93E-18	3	0.272727		
DMR16:42694101	16	42694101	700	2	5.28E-07	0	0		
DMR16:42883301	16	42883301	100	1	1.94E-15	0	0		
DMR16:43710201	16	43710201	200	2	4.06E-12	0	0		
DMR16:46517301	16	46517301	200	2	5.93E-18	0	0		
DMR16:48532201	16	48532201	1100	1	9.21E-07	13	1.181818		
DMR16:50989501	16	50989501	100	1	9.35E-07	0	0		
DMR16:51239901	16	51239901	400	1	1.05E-07	0	0	U6	
DMR16:51543401	16	51543401	900	2	3.05E-11	7	0.777778		
DMR16:51914101	16	51914101	2100	1	1.50E-11	29	1.380952		
DMR16:52648701	16	52648701	1200	1	2.78E-07	5	0.416667		
DMR16:55467501	16	55467501	1000	1	5.40E-19	4	0.4		
DMR16:56833001	16	56833001	200	1	1.08E-07	1	0.5	Msr1	Receptor
DMR16:57099001	16	57099001	200	2	5.95E-09	1	0.5		
DMR16:59293001	16	59293001	200	2	9.27E-14	0	0		
DMR16:66672201	16	66672201	2900	1	6.27E-08	9	0.310345		
DMR16:72568201	16	72568201	900	2	4.24E-17	4	0.444444		
DMR16:76548301	16	76548301	900	1	6.65E-12	6	0.666667		
DMR16:79853201	16	79853201	200	2	3.93E-18	0	0		
DMR16:80436601	16	80436601	100	1	1.04E-07	0	0		
DMR16:88685201	16	88685201	200	2	3.47E-14	0	0		
DMR17:7006801	17	7006801	1300	1	3.26E-08	14	1.076923	Klhl3	Transcription
DMR17:12668101	17	12668101	100	1	3.57E-07	0	0	Syk	Signaling
DMR17:28525301	17	28525301	500	1	1.70E-07	8	1.6	F13a1	Immune
DMR17:28894901	17	28894901	800	1	1.77E-08	3	0.375	SNORA17	
DMR17:32802001	17	32802001	800	1	2.66E-13	1	0.125		
DMR17:42859901	17	42859901	200	1	5.74E-08	4	2	Prl3d2	Hormone
DMR17:44826501	17	44826501	100	1	5.25E-09	0	0	Hist1h2bd;AC114096.1	

DMR17:53573701	17	53573701	100	1	6.12E-09	0	0	Hecw1	Protease
DMR17:58876801	17	58876801	300	3	1.46E-39	0	0		
DMR17:60107201	17	60107201	1500	1	9.92E-07	13	0.866667	Mpp7	Cytoskeleton
DMR17:60271701	17	60271701	200	1	8.43E-08	2	1		
DMR17:61736601	17	61736601	600	1	1.70E-07	9	1.5		
DMR17:67967801	17	67967801	200	2	1.12E-19	0	0		
DMR17:71350401	17	71350401	2200	1	2.67E-09	30	1.363636		
DMR17:74448601	17	74448601	100	1	2.00E-07	0	0		
DMR17:76513301	17	76513301	800	3	9.53E-40	10	1.25		
DMR17:77414301	17	77414301	100	1	7.39E-13	0	0		
DMR17:83143701	17	83143701	200	1	2.82E-18	0	0		
DMR17:83353001	17	83353001	1000	1	3.12E-13	4	0.4	Plxdc2	Binding Protein
DMR18:9944401	18	9944401	100	1	1.46E-07	0	0		
DMR18:14004001	18	14004001	600	1	5.39E-09	0	0	Nol4	Translation
DMR18:18529901	18	18529901	200	2	4.61E-10	0	0		
DMR18:19780601	18	19780601	100	1	5.25E-10	0	0		
DMR18:22357201	18	22357201	800	1	4.04E-11	6	0.75	AABR07031612.1	
DMR18:33479201	18	33479201	500	1	3.55E-12	6	1.2		
DMR18:41235701	18	41235701	100	1	5.74E-13	0	0		
DMR18:42044701	18	42044701	200	2	4.02E-12	0	0		
DMR18:55146701	18	55146701	4000	1	7.80E-07	48	1.2		
DMR18:60532401	18	60532401	300	3	6.62E-20	5	1.666667	Nedd4l	Protease
DMR18:67270701	18	67270701	500	1	7.72E-07	0	0		
DMR18:67837501	18	67837501	1200	2	1.09E-19	12	1		
DMR18:72048001	18	72048001	100	1	9.35E-07	0	0	Zbtb7c	Transcription
DMR18:72093001	18	72093001	400	1	1.63E-08	2	0.5	Zbtb7c	Transcription
DMR18:72158401	18	72158401	100	1	3.27E-07	0	0	Zbtb7c	Transcription
DMR18:77216401	18	77216401	2500	4	1.53E-14	43	1.72	Nfatc1	Transcription
DMR18:83102801	18	83102801	1100	2	1.57E-13	0	0		
DMR18:84125401	18	84125401	200	2	1.85E-09	0	0		
DMR18:85815001	18	85815001	100	1	4.32E-07	0	0	AABR07032851.1	
DMR18:86277901	18	86277901	200	2	4.52E-14	1	0.5	LOC689166	
DMR19:1933801	19	1933801	800	1	8.05E-09	5	0.625		
DMR19:6420601	19	6420601	100	1	7.60E-08	2	2	Cdh8	Extracellular Matrix
DMR19:8630001	19	8630001	1300	3	1.33E-30	2	0.153846		
DMR19:12835201	19	12835201	400	2	8.36E-14	1	0.25	Large1	
DMR19:14350201	19	14350201	100	1	2.29E-08	0	0	Gm6576;Hmgxb4	
DMR19:14401501	19	14401501	400	2	1.51E-10	10	2.5	Tom1	Signaling
DMR19:15214101	19	15214101	1000	2	4.79E-13	10	1	Ces1d;Ces1f	Metabolism
DMR19:16350901	19	16350901	200	2	3.11E-15	0	0		
DMR19:18575301	19	18575301	300	1	1.53E-07	8	2.666667		
DMR19:21365901	19	21365901	1100	2	2.51E-07	4	0.363636	AABR07043106.1; Siah1	
DMR19:34223201	19	34223201	3200	7	7.79E-10	109	3.40625	Arhgap10	Signaling
DMR19:36446401	19	36446401	7400	1	9.16E-07	66	0.891892		
DMR19:37030301	19	37030301	100	1	1.77E-07	1	1	AABR07072639.2; Ces2g	
DMR19:40530601	19	40530601	800	1	1.92E-10	15	1.875		
DMR19:50419001	19	50419001	200	2	1.63E-10	2	1		
DMR19:56956901	19	56956901	1300	1	8.88E-08	19	1.461538		
DMR19:59640901	19	59640901	200	1	2.41E-11	1	0.5		
DMR19:61995501	19	61995501	1100	2	5.32E-11	5	0.454545		

DMR20:1702601	20	1702601	200	1	4.66E-16	1	0.5	Olr1732-ps;Olr1733	
DMR20:5397801	20	5397801	3500	1	6.93E-07	49	1.4	RT1-A1	Immune
DMR20:12673101	20	12673101	200	2	1.50E-14	1	0.5	Col6a1	Cytoskeleton
DMR20:15104801	20	15104801	200	2	1.68E-22	0	0	Pcdh15	Extracellular Matrix
DMR20:15595701	20	15595701	5200	1	8.58E-09	81	1.557692		
DMR20:15888301	20	15888301	1600	1	3.34E-09	19	1.1875		
DMR20:16504801	20	16504801	100	1	4.79E-13	0	0		
DMR20:17272801	20	17272801	200	1	9.19E-12	0	0		
DMR20:17393001	20	17393001	100	1	2.13E-08	1	1		
DMR20:18563901	20	18563901	1700	1	1.97E-08	18	1.058824	Ube2d1	Metabolism
DMR20:18592101	20	18592101	200	2	1.44E-07	0	0	Tfam	
DMR20:19381901	20	19381901	2800	2	2.33E-09	92	3.285714	Fam13c	
DMR20:21810101	20	21810101	100	1	2.89E-11	0	0	Arid5b	Transcription
DMR20:22489601	20	22489601	200	1	1.94E-10	2	1	AABR07044825.1	
DMR20:26002301	20	26002301	2300	2	3.22E-09	21	0.913043	Lrrtm3	Receptor
DMR20:26015601	20	26015601	1200	1	1.79E-07	5	0.416667	Lrrtm3	Receptor
DMR20:33126201	20	33126201	400	1	8.54E-07	10	2.5	Ros1	Receptor
DMR20:40503001	20	40503001	100	1	1.60E-11	0	0	Hs3st5;AABR07045274.1	Metabolism
DMR20:44098401	20	44098401	1100	1	1.82E-11	9	0.818182	Lama4	Extracellular Matrix
DMR20:46087201	20	46087201	300	1	4.40E-07	14	4.666667	Ak9	
DMR20:47886701	20	47886701	700	1	3.11E-11	13	1.857143	Sobp	
DMR20:50687001	20	50687001	400	2	4.88E-09	6	1.5	Hace1	Translation
DMR20:50933201	20	50933201	900	1	4.58E-09	6	0.666667		
DMR20:51564901	20	51564901	1800	1	4.00E-16	7	0.388889		
DMRX:20917801	X	20917801	100	1	1.10E-12	0	0	FAM120C	
DMRX:24712801	X	24712801	200	2	2.51E-10	0	0		
DMRX:26341901	X	26341901	200	2	8.19E-14	0	0	Arhgap6	Cytoskeleton
DMRX:26696201	X	26696201	400	1	3.74E-07	0	0		
DMRX:27359401	X	27359401	200	1	3.50E-07	0	0		
DMRX:27416501	X	27416501	200	1	2.73E-08	0	0		
DMRX:27783901	X	27783901	1200	2	6.64E-22	4	0.333333		
DMRX:27979301	X	27979301	100	1	3.57E-07	0	0		
DMRX:29786701	X	29786701	200	1	6.12E-09	0	0		
DMRX:31504101	X	31504101	200	2	6.33E-13	0	0		
DMRX:36223301	X	36223301	200	2	5.43E-15	1	0.5		
DMRX:36525101	X	36525101	200	2	4.38E-14	0	0		
DMRX:36968601	X	36968601	100	1	5.95E-09	0	0	Adgrg2	
DMRX:43477201	X	43477201	900	2	1.43E-13	5	0.555556		
DMRX:43551701	X	43551701	200	2	5.71E-10	1	0.5	Acot9	Metabolism
DMRX:43715301	X	43715301	100	1	5.58E-18	0	0		
DMRX:44317601	X	44317601	700	2	1.19E-17	3	0.428571		
DMRX:46026701	X	46026701	100	1	1.26E-14	0	0		
DMRX:46575801	X	46575801	100	1	6.23E-08	0	0		
DMRX:46639901	X	46639901	1200	2	6.15E-11	4	0.333333	AABR07038349.1	
DMRX:49468701	X	49468701	100	1	2.42E-12	0	0		
DMRX:51786901	X	51786901	4400	1	3.89E-07	261	5.931818	Dmd;LOC102549011	Development
DMRX:53490301	X	53490301	200	1	7.43E-11	0	0	Dmd	Development
DMRX:53782501	X	53782501	100	1	2.77E-07	0	0		
DMRX:56819401	X	56819401	200	2	4.72E-17	1	0.5		
DMRX:56850701	X	56850701	100	1	3.47E-13	0	0		

DMRX:57077101	X	57077101	200	2	1.05E-12	0	0		
DMRX:57198801	X	57198801	100	1	5.35E-13	0	0		
DMRX:61777801	X	61777801	100	1	4.36E-11	0	0		
DMRX:62094501	X	62094501	100	1	6.65E-12	1	1		
DMRX:63061301	X	63061301	1000	2	1.43E-28	5	0.5	RGD1564534;LOC367830	
DMRX:79062401	X	79062401	200	2	3.91E-34	1	0.5	RGD1561552	
DMRX:81167501	X	81167501	100	1	6.42E-07	0	0		
DMRX:81181501	X	81181501	1100	1	4.06E-07	4	0.363636		
DMRX:81790901	X	81790901	100	1	1.03E-23	0	0		
DMRX:82180501	X	82180501	200	2	9.30E-21	0	0		
DMRX:83080001	X	83080001	200	2	2.11E-12	1	0.5	Hdx	EST
DMRX:87091401	X	87091401	1500	1	1.20E-08	16	1.066667		
DMRX:87096101	X	87096101	100	1	5.57E-12	1	1		
DMRX:87169701	X	87169701	100	1	2.26E-18	0	0	SCARNA2	
DMRX:87617201	X	87617201	300	1	1.63E-07	2	0.666667		
DMRX:88788001	X	88788001	200	2	3.07E-14	0	0		
DMRX:89419001	X	89419001	200	1	2.75E-08	0	0		
DMRX:90089401	X	90089401	200	2	7.78E-22	0	0		
DMRX:97203301	X	97203301	200	2	8.21E-15	1	0.5		
DMRX:97267801	X	97267801	1700	1	4.48E-10	3	0.176471	Cldn34c4	
DMRX:101301101	X	101301101	200	2	1.53E-13	0	0		
DMRX:101909001	X	101909001	100	1	3.00E-13	0	0		
DMRX:102033501	X	102033501	1200	1	2.29E-08	3	0.25	AABR07040480.1	
DMRX:105741101	X	105741101	100	1	1.02E-09	1	1	SNORA42	
DMRX:108792501	X	108792501	300	1	3.26E-08	0	0	Il1rapl2	Receptor
DMRX:108911001	X	108911001	200	2	1.19E-17	0	0	Il1rapl2	Receptor
DMRX:112095001	X	112095001	1200	1	4.32E-07	3	0.25	Mid2	Cytoskeleton
DMRX:113172801	X	113172801	100	1	1.92E-10	0	0		
DMRX:113249901	X	113249901	200	1	7.41E-07	1	0.5		
DMRX:117028601	X	117028601	100	1	1.26E-14	0	0		
DMRX:117801201	X	117801201	100	1	2.11E-12	0	0		
DMRX:124383501	X	124383501	100	1	1.46E-07	0	0	Zbtb33;Tmem255a	Transcription
DMRX:127178601	X	127178601	200	2	8.19E-14	0	0		
DMRX:131310301	X	131310301	3400	1	9.41E-09	41	1.205882		
DMRX:133728201	X	133728201	200	2	2.61E-30	1	0.5		
DMRX:134226801	X	134226801	200	2	6.08E-10	0	0		
DMRX:136953501	X	136953501	100	1	1.29E-09	0	0		
DMRX:141108601	X	141108601	200	1	9.49E-07	2	1		
DMRX:141134301	X	141134301	100	1	2.98E-20	0	0		
DMRX:143155301	X	143155301	200	1	4.20E-07	0	0	Mcf2	Signaling
DMRX:144641901	X	144641901	200	2	5.06E-14	0	0		
DMRX:145739701	X	145739701	200	2	1.18E-14	0	0		
DMRX:146026101	X	146026101	200	2	1.54E-10	0	0		
DMRX:149104301	X	149104301	100	1	6.08E-10	0	0		
DMRX:149761301	X	149761301	1900	1	1.57E-08	17	0.894737		
DMRX:150463001	X	150463001	700	1	2.11E-12	5	0.714286		
DMRY:1772801	Y	1772801	100	1	1.04E-15	0	0		
DMRY:2691401	Y	2691401	1500	1	7.41E-07	14	0.933333		
DMRY:2732501	Y	2732501	100	1	5.71E-10	0	0		
