# Supplementary material for: Alterations in sperm DNA methylation, non-coding RNA and histone retention associate with DDT-induced epigenetic transgenerational inheritance of disease
Source: Epigenetics Chromatin. 2018 Feb 27;11:8. doi: 10.1186/s13072-018-0178-0 (PMC5827984; doi:10.1186/s13072-018-0178-0)
Supplement: Supplementary file 6 — Additional file 6: Table S4. (A) Site Table F1 lncRNA p<1e-04, (B) F1 sncRNA p < 1e−04. [file 13072_2018_178_MOESM6_ESM.pdf]

Supplemental Table S4A
Site Table F1 lncRNA p<1e-04

Test ID	Gene ID	Chr	Start	Stop	Log 2.fold Change.	Test Stat	minP	q_value	Gene Association	Gene Category
TCONS_00000124	XLOC_000032	1	7658889	7673683	-2.06133	-2.65428	5.00E-05	0.00621615	Fuca2	Metabolism
TCONS_00000553	XLOC_000128	1	23906716	23909566	-2.81626	-3.05162	5.00E-05	0.00621615	Tcf21	Transcription
TCONS_00002401	XLOC_000576	1	81406418	81441730	2.71754	2.30324	5.00E-05	0.00621615	Irgq;Pinlyp;Xrcc1;Zfp575	Transcription
TCONS_00005690	XLOC_001394	1	178636473	179010296	-2.54649	-2.85637	5.00E-05	0.00621615	Spon1;Rras2	Growth Factors & Cytokines;Signaling
TCONS_00005699	XLOC_001396	1	179477523	179620954	#NAME?	NA	5.00E-05	0.00621615		
TCONS_00007138	XLOC_001673	1	214328070	214353461	-2.28883	-2.43995	5.00E-05	0.00621615	Tmem80;Eps8l2	Unknown
TCONS_00007139	XLOC_001674	1	214375514	214388652	-1.63271	-2.36253	5.00E-05	0.00621615	Taldo1;LOC100911365	Metabolism
TCONS_00007549	XLOC_001753	1	220114227	220120248	-2.08196	-2.91238	5.00E-05	0.00621615	U1;Ctsf;Actn3	Proteolysis;Cytoskeleton
TCONS_00007879	XLOC_001834	1	224876731	224876807	Inf	NA	5.00E-05	0.00621615	Chrm1	Receptor
TCONS_00008878	XLOC_002104	1	256035822	256088395	2.58323	2.26886	5.00E-05	0.00621615	Kif11	Cytoskeleton
TCONS_00010847	XLOC_002599	1	47217132	47331589	-2.34319	-2.68941	5.00E-05	0.00621615	Dynlt1;Sytl3;Ezr	Development;Transport;Cytoskeleton
TCONS_00012306	XLOC_002987	1	84118045	84150084	-1.7517	-2.52921	5.00E-05	0.00621615	Ltbp4;Shkbp1	Receptor;Transport
TCONS_00012395	XLOC_003018	1	86832276	86832743	#NAME?	NA	5.00E-05	0.00621615		
TCONS_00013328	XLOC_003238	1	102110583	102191313	-4.25165	-3.30297	5.00E-05	0.00621615	Kcnj11;Abcc8	Transport
TCONS_00013521	XLOC_003306	1	116565789	116567189	-2.92863	-3.29837	5.00E-05	0.00621615	LOC100912195	
TCONS_00014619	XLOC_003626	1	162378597	162385830	1.47629	2.36323	5.00E-05	0.00621615	Ndufc2;Thrsp	Metabolism
TCONS_00015117	XLOC_003773	1	170317112	170318935	-2.58157	-3.26879	5.00E-05	0.00621615	Prkcdbp	Transcription
TCONS_00015396	XLOC_003854	1	176296192	176607466	-2.39848	-2.86502	5.00E-05	0.00621615	Galnt18	Unknown
TCONS_00016539	XLOC_004115	1	211440334	211520699	-3.77928	-3.9244	5.00E-05	0.00621615	Dpysl4;Stk32c	Metabolism;Signaling
TCONS_00017749	XLOC_004346	1	224900743	224921117	-1.67952	-2.44432	5.00E-05	0.00621615	Slc3a2;SNORD25;SNORD26;SNORD27;SNORD28;SNORD29;SNORD30;SNORD31;SNORD22;AC099294.1	Metabolism
TCONS_00018133	XLOC_004462	1	236773118	236900904	-3.37157	-3.21168	5.00E-05	0.00621615	AABR07006480.1	
TCONS_00018558	XLOC_004561	1	252536786	252550395	-3.67202	-4.20828	5.00E-05	0.00621615	Stambpl1;Acta2	Growth Factors & Cytokines;Cytoskeleton
TCONS_00019666	XLOC_004848	1	2219710	2219784	Inf	NA	5.00E-05	0.00621615		
TCONS_00019747	XLOC_004929	1	3067780	3067881	Inf	NA	5.00E-05	0.00621615		
TCONS_00020372	XLOC_005554	1	9081562	9081637	Inf	NA	5.00E-05	0.00621615		
TCONS_00020621	XLOC_005798	1	12679266	12757959	2.20811	2.64624	5.00E-05	0.00621615	AC128394.2	
TCONS_00021074	XLOC_006240	1	21826100	21826176	Inf	NA	5.00E-05	0.00621615		
TCONS_00021147	XLOC_006310	1	22455938	22456034	Inf	NA	5.00E-05	0.00621615	Taar7h;AC128759.2;Taar7g	Receptor
TCONS_00021201	XLOC_006364	1	24090791	24090866	Inf	NA	5.00E-05	0.00621615	AABR07000714.2	
TCONS_00021206	XLOC_006369	1	24334240	24334316	Inf	NA	5.00E-05	0.00621615	U6	
TCONS_00021283	XLOC_006446	1	27232347	27232423	Inf	NA	5.00E-05	0.00621615		
TCONS_00021454	XLOC_006613	1	33324093	33324488	#NAME?	NA	5.00E-05	0.00621615		
TCONS_00021467	XLOC_006626	1	33743466	33743541	Inf	NA	5.00E-05	0.00621615	AABR07001018.3	
TCONS_00021526	XLOC_006683	1	34623968	34624162	Inf	NA	5.00E-05	0.00621615		
TCONS_00021670	XLOC_006827	1	38520673	38520749	Inf	NA	5.00E-05	0.00621615	LOC102551340	Transcription
TCONS_00021750	XLOC_006903	1	39262684	39262985	#NAME?	NA	5.00E-05	0.00621615		
TCONS_00022228	XLOC_007371	1	49011879	49011955	Inf	NA	5.00E-05	0.00621615		
TCONS_00022266	XLOC_007407	1	49163099	49164020	Inf	NA	5.00E-05	0.00621615		
TCONS_00022520	XLOC_007620	1	53909226	53909302	Inf	NA	5.00E-05	0.00621615		
TCONS_00022958	XLOC_008039	1	62543797	62543871	Inf	NA	5.00E-05	0.00621615	AABR07001942.1	
TCONS_00023003	XLOC_008084	1	64836737	64836810	Inf	NA	5.00E-05	0.00621615	AABR07002010.1	
TCONS_00023169	XLOC_008250	1	71745965	71746132	Inf	NA	5.00E-05	0.00621615		

TCONS_00023201	XLOC_008282	1	72634910	72635055	Inf	NA	5.00E-05	0.00621615	AABR07071876.1;Tmem190;Il11;Fam71e2	
TCONS_00023443	XLOC_008515	1	78867339	78867415	Inf	NA	5.00E-05	0.00621615	Pnmal2	Unknown
TCONS_00023455	XLOC_008527	1	78917051	78917478	#NAME?	NA	5.00E-05	0.00621615		
TCONS_00023486	XLOC_008558	1	79285573	79286234	Inf	NA	5.00E-05	0.00621615	AABR07002659.1	
TCONS_00023570	XLOC_008641	1	81363328	81363759	Inf	NA	5.00E-05	0.00621615	Cadm4;Zfp428	Receptor
TCONS_00023629	XLOC_008700	1	83444185	83444261	Inf	NA	5.00E-05	0.00621615	AC142154.1	
TCONS_00023871	XLOC_008942	1	89312158	89312335	Inf	NA	5.00E-05	0.00621615	Ffar3;Ffar1;Cd22	Receptor;Extracellular Matrix
TCONS_00023960	XLOC_009020	1	92300296	92301429	Inf	NA	5.00E-05	0.00621615		
TCONS_00023995	XLOC_009055	1	92532373	92532449	Inf	NA	5.00E-05	0.00621615		
TCONS_00024044	XLOC_009104	1	92824148	92824223	Inf	NA	5.00E-05	0.00621615		
TCONS_00024075	XLOC_009135	1	93956226	93956300	Inf	NA	5.00E-05	0.00621615	Zfp536	Transcription
TCONS_00024227	XLOC_009287	1	98237895	98237971	Inf	NA	5.00E-05	0.00621615	LOC102555672	Transcription
TCONS_00024392	XLOC_009450	1	104403726	104403802	Inf	NA	5.00E-05	0.00621615		
TCONS_00024447	XLOC_009505	1	107500951	107501204	#NAME?	NA	5.00E-05	0.00621615		
TCONS_00024584	XLOC_009641	1	113987503	113988311	Inf	NA	5.00E-05	0.00621615		
TCONS_00024624	XLOC_009680	1	115569894	115570687	Inf	NA	5.00E-05	0.00621615		
TCONS_00024646	XLOC_009702	1	115625979	115626154	Inf	NA	5.00E-05	0.00621615		
TCONS_00024895	XLOC_009946	1	122393294	122393402	Inf	NA	5.00E-05	0.00621615		
TCONS_00025180	XLOC_010231	1	127792931	127793055	Inf	NA	5.00E-05	0.00621615	Adamts17	Proteolysis
TCONS_00025199	XLOC_010249	1	128393309	128393384	Inf	NA	5.00E-05	0.00621615	LOC103691157	
TCONS_00025481	XLOC_010524	1	131642798	131643335	#NAME?	NA	5.00E-05	0.00621615		
TCONS_00025483	XLOC_010526	1	131648731	131649074	#NAME?	NA	5.00E-05	0.00621615		
TCONS_00025739	XLOC_010776	1	140499367	140499443	Inf	NA	5.00E-05	0.00621615		
TCONS_00025945	XLOC_010982	1	145704211	145704287	Inf	NA	5.00E-05	0.00621615		
TCONS_00025974	XLOC_011011	1	146395325	146395401	Inf	NA	5.00E-05	0.00621615	Arnt2	Transcription
TCONS_00026144	XLOC_011175	1	150757928	150758532	Inf	NA	5.00E-05	0.00621615		
TCONS_00026186	XLOC_011217	1	152667091	152667682	Inf	NA	5.00E-05	0.00621615		
TCONS_00026188	XLOC_011219	1	152669042	152669880	Inf	NA	5.00E-05	0.00621615		
TCONS_00026190	XLOC_011221	1	152670314	152672225	Inf	NA	5.00E-05	0.00621615		
TCONS_00026191	XLOC_011222	1	152672535	152674858	Inf	NA	5.00E-05	0.00621615		
TCONS_00026193	XLOC_011224	1	152675870	152676315	Inf	NA	5.00E-05	0.00621615		
TCONS_00026199	XLOC_011230	1	152686862	152687834	Inf	NA	5.00E-05	0.00621615		
TCONS_00026200	XLOC_011231	1	152687917	152693319	Inf	NA	5.00E-05	0.00621615		
TCONS_00026328	XLOC_011359	1	157527006	157527080	Inf	NA	5.00E-05	0.00621615		
TCONS_00026370	XLOC_011401	1	159087505	159087703	Inf	NA	5.00E-05	0.00621615		
TCONS_00026545	XLOC_011576	1	160218429	160219510	Inf	NA	5.00E-05	0.00621615		
TCONS_00026628	XLOC_011659	1	161220151	161220574	#NAME?	NA	5.00E-05	0.00621615	Mir708	
TCONS_00026655	XLOC_011686	1	162447112	162447187	Inf	NA	5.00E-05	0.00621615	Ints4	Signaling
TCONS_00026717	XLOC_011747	1	163624924	163625098	Inf	NA	5.00E-05	0.00621615		
TCONS_00026746	XLOC_011776	1	164167205	164167281	Inf	NA	5.00E-05	0.00621615		
TCONS_00026799	XLOC_011829	1	165162542	165162984	Inf	NA	5.00E-05	0.00621615	Lipt2	
TCONS_00027340	XLOC_012369	1	180973900	180973992	Inf	NA	5.00E-05	0.00621615		
TCONS_00027398	XLOC_012427	1	184800377	184800753	#NAME?	NA	5.00E-05	0.00621615		
TCONS_00027405	XLOC_012434	1	184887789	184888176	#NAME?	NA	5.00E-05	0.00621615		
TCONS_00027638	XLOC_012655	1	192845222	192845358	Inf	NA	5.00E-05	0.00621615		
TCONS_00027660	XLOC_012677	1	193034813	193035310	#NAME?	NA	5.00E-05	0.00621615		
TCONS_00027702	XLOC_012719	1	194339295	194339371	Inf	NA	5.00E-05	0.00621615		
TCONS_00027716	XLOC_012733	1	195347843	195347919	Inf	NA	5.00E-05	0.00621615		
TCONS_00028048	XLOC_013063	1	202038654	202039561	Inf	NA	5.00E-05	0.00621615		
TCONS_00028049	XLOC_013064	1	202039896	202040125	Inf	NA	5.00E-05	0.00621615		
TCONS_00028081	XLOC_013096	1	202854877	202855518	#NAME?	NA	5.00E-05	0.00621615		
TCONS_00028082	XLOC_013097	1	202855977	202856697	#NAME?	NA	5.00E-05	0.00621615		
TCONS_00028089	XLOC_013104	1	202901433	202901509	Inf	NA	5.00E-05	0.00621615	AABR07005838.1	
TCONS_00028125	XLOC_013140	1	203013842	203024151	4.41369	4.11554	5.00E-05	0.00621615		
TCONS_00028242	XLOC_013248	1	208245835	208245910	Inf	NA	5.00E-05	0.00621615		
TCONS_00028255	XLOC_013261	1	208399635	208399709	Inf	NA	5.00E-05	0.00621615		
TCONS_00028271	XLOC_013277	1	208659743	208660717	#NAME?	NA	5.00E-05	0.00621615		
TCONS_00028412	XLOC_013417	1	212086232	212086901	Inf	NA	5.00E-05	0.00621615		
TCONS_00028518	XLOC_013523	1	214212959	214213035	Inf	NA	5.00E-05	0.00621615	Rassf7;Mir3574;Phrf1	
TCONS_00028536	XLOC_013541	1	215019013	215019159	Inf	NA	5.00E-05	0.00621615	U6	
TCONS_00028619	XLOC_013623	1	217911614	217912045	#NAME?	NA	5.00E-05	0.00621615	Ano1	Unknown

TCONS_00028627	XLOC_013631	1	217933951	217934579	#NAME?	NA	5.00E-05	0.00621615	AC095937.1	
TCONS_00028629	XLOC_013633	1	217939220	217939696	#NAME?	NA	5.00E-05	0.00621615	AC095937.1	
TCONS_00028673	XLOC_013677	1	218415391	218415468	Inf	NA	5.00E-05	0.00621615	Tpcn2	Transport
TCONS_00028682	XLOC_013686	1	219062510	219062585	Inf	NA	5.00E-05	0.00621615		
TCONS_00028824	XLOC_013818	1	221390234	221390309	Inf	NA	5.00E-05	0.00621615		
TCONS_00028848	XLOC_013842	1	222081022	222081554	Inf	NA	5.00E-05	0.00621615	Rps6ka4	Signaling
TCONS_00028857	XLOC_013851	1	222124219	222124649	Inf	NA	5.00E-05	0.00621615	Ccdc88b	Signaling
TCONS_00028924	XLOC_013918	1	223337254	223337737	#NAME?	NA	5.00E-05	0.00621615		
TCONS_00028925	XLOC_013919	1	223339791	223340561	#NAME?	NA	5.00E-05	0.00621615		
TCONS_00028932	XLOC_013926	1	223344214	223344956	#NAME?	NA	5.00E-05	0.00621615		
TCONS_00028937	XLOC_013931	1	223348134	223348359	#NAME?	NA	5.00E-05	0.00621615		
TCONS_00028941	XLOC_013935	1	223362719	223362883	#NAME?	NA	5.00E-05	0.00621615		
TCONS_00028944	XLOC_013938	1	223387182	223387359	#NAME?	NA	5.00E-05	0.00621615		
TCONS_00029114	XLOC_014107	1	228071673	228071833	Inf	NA	5.00E-05	0.00621615		
TCONS_00029215	XLOC_014208	1	232576432	232576506	Inf	NA	5.00E-05	0.00621615		
TCONS_00029243	XLOC_014236	1	233270905	233271205	Inf	NA	5.00E-05	0.00621615		
TCONS_00029259	XLOC_014252	1	233334669	233334934	Inf	NA	5.00E-05	0.00621615		
TCONS_00029654	XLOC_014640	1	243623099	243623580	#NAME?	NA	5.00E-05	0.00621615		
TCONS_00029693	XLOC_014679	1	243735446	243736375	#NAME?	NA	5.00E-05	0.00621615		
TCONS_00029700	XLOC_014686	1	243758327	243758678	#NAME?	NA	5.00E-05	0.00621615		
TCONS_00029766	XLOC_014752	1	243998455	243998837	Inf	NA	5.00E-05	0.00621615		
TCONS_00029782	XLOC_014768	1	244956662	244958239	#NAME?	NA	5.00E-05	0.00621615		
TCONS_00029783	XLOC_014769	1	244958291	244958896	#NAME?	NA	5.00E-05	0.00621615		
TCONS_00030095	XLOC_015071	1	249553139	249553681	#NAME?	NA	5.00E-05	0.00621615		
TCONS_00030120	XLOC_015096	1	249597365	249597441	Inf	NA	5.00E-05	0.00621615		
TCONS_00030185	XLOC_015161	1	249682041	249682798	#NAME?	NA	5.00E-05	0.00621615		
TCONS_00030234	XLOC_015210	1	249861942	249862662	#NAME?	NA	5.00E-05	0.00621615		
TCONS_00030293	XLOC_015268	1	249968040	249968677	#NAME?	NA	5.00E-05	0.00621615		
TCONS_00030314	XLOC_015285	1	250005413	250006248	#NAME?	NA	5.00E-05	0.00621615		
TCONS_00030422	XLOC_015379	1	252478473	252478549	Inf	NA	5.00E-05	0.00621615		
TCONS_00030527	XLOC_015484	1	255377458	255378279	#NAME?	NA	5.00E-05	0.00621615	Ppp1r3c	Signaling
TCONS_00030532	XLOC_015489	1	255389156	255390089	#NAME?	NA	5.00E-05	0.00621615		
TCONS_00030673	XLOC_015623	1	261382153	261382983	Inf	NA	5.00E-05	0.00621615	Marveld1	
TCONS_00030748	XLOC_015698	1	262785174	262785250	Inf	NA	5.00E-05	0.00621615		
TCONS_00030786	XLOC_015736	1	264259864	264259940	Inf	NA	5.00E-05	0.00621615	Wnt8b;Sec31b	Signaling;Transport
TCONS_00030931	XLOC_015875	1	267472076	267473163	Inf	NA	5.00E-05	0.00621615	Col17a1;AC096311.1;Sfr1	Extracellular Matrix
TCONS_00031037	XLOC_015981	1	270893477	270894010	Inf	NA	5.00E-05	0.00621615		
TCONS_00031040	XLOC_015984	1	270895530	270896177	Inf	NA	5.00E-05	0.00621615		
TCONS_00031069	XLOC_016013	1	273553864	273554011	Inf	NA	5.00E-05	0.00621615	RGD1561333	
TCONS_00031441	XLOC_016367	1	279573252	279573885	Inf	NA	5.00E-05	0.00621615		
TCONS_00031522	XLOC_016448	1	280733709	280733963	#NAME?	NA	5.00E-05	0.00621615		
TCONS_00127065	XLOC_067008	2	2605995	2616217	-2.02293	-2.62472	5.00E-05	0.00621615	Glrx	Metabolism
TCONS_00128942	XLOC_067532	2	116432690	116433110	4.68064	4.81194	5.00E-05	0.00621615	Terc	
TCONS_00129322	XLOC_067603	2	129293921	129295991	Inf	NA	5.00E-05	0.00621615		
TCONS_00129454	XLOC_067647	2	142494631	142495451	Inf	NA	5.00E-05	0.00621615		
TCONS_00129979	XLOC_067781	2	171294073	171295305	Inf	NA	5.00E-05	0.00621615		
TCONS_00130331	XLOC_067883	2	187737335	187743092	-3.13672	-3.1043	5.00E-05	0.00621615	Smg5;Paqr6;Bglap;Pmf1	Receptor;Extracellular Matrix
TCONS_00130696	XLOC_067931	2	190007087	190008742	-2.21021	-2.40691	5.00E-05	0.00621615	S100a4;S100a5;S100a6	Signaling;Receptor
TCONS_00131767	XLOC_068206	2	225005018	225036188	-1.64304	-2.42041	5.00E-05	0.00621615	Cnn3;AABR07013154.2;Slc44a3	Signaling;Metabolism
TCONS_00131868	XLOC_068231	2	230660663	230662084	#NAME?	NA	5.00E-05	0.00621615	AABR07013255.1	
TCONS_00131926	XLOC_068243	2	232350107	232352867	Inf	NA	5.00E-05	0.00621615		
TCONS_00132136	XLOC_068293	2	240991742	241096066	4.37822	5.64431	5.00E-05	0.00621615	Slc39a8	Transport
TCONS_00132326	XLOC_068349	2	251634430	251766014	-3.02894	-3.32214	5.00E-05	0.00621615	Ddah1	Metabolism
TCONS_00136328	XLOC_069277	2	191291477	191294374	Inf	NA	5.00E-05	0.00621615	RGD1562234	
TCONS_00136571	XLOC_069348	2	197854916	197860741	-2.75143	-3.25332	5.00E-05	0.00621615	Ecm1;Tars2	Cytoskeleton;Translation
TCONS_00136873	XLOC_069413	2	204003741	204048788	-1.48051	-2.2347	5.00E-05	0.00621615	Atp1a1;AABR07012728.1;AABR07012728.2	Metabolism
TCONS_00136908	XLOC_069429	2	206216424	206222248	-2.28898	-2.56175	5.00E-05	0.00621615	Olfml3;Hipk1	Development;Signaling

TCONS_00137221	XLOC_069490	2	211342636	211344489	2.64677	4.14058	5.00E-05	0.00621615	Cox6b1;SCARNA2;LOC108349010	Electron Transport
TCONS_00137980	XLOC_069669	2	250842680	250863940	-2.65469	-2.63138	5.00E-05	0.00621615	Clca4	Transport
TCONS_00138332	XLOC_069822	2	1203772	1206266	-4.1022	-4.53158	5.00E-05	0.00621615		
TCONS_00138333	XLOC_069823	2	1206923	1207303	#NAME?	NA	5.00E-05	0.00621615		
TCONS_00138338	XLOC_069828	2	1872958	1873033	Inf	NA	5.00E-05	0.00621615		
TCONS_00138345	XLOC_069835	2	1961114	1961204	Inf	NA	5.00E-05	0.00621615		
TCONS_00138357	XLOC_069847	2	1978549	1978659	Inf	NA	5.00E-05	0.00621615		
TCONS_00138602	XLOC_070091	2	4653743	4653818	Inf	NA	5.00E-05	0.00621615	RGD1560883	Unknown
TCONS_00138696	XLOC_070185	2	5907192	5907268	Inf	NA	5.00E-05	0.00621615		
TCONS_00138732	XLOC_070221	2	6104678	6104753	Inf	NA	5.00E-05	0.00621615		
TCONS_00138860	XLOC_070349	2	11065506	11065582	Inf	NA	5.00E-05	0.00621615		
TCONS_00139202	XLOC_070690	2	23593156	23593272	Inf	NA	5.00E-05	0.00621615		
TCONS_00139295	XLOC_070783	2	25312773	25312868	Inf	NA	5.00E-05	0.00621615		
TCONS_00139296	XLOC_070784	2	25320877	25323191	Inf	NA	5.00E-05	0.00621615	AABR07007715.1	
TCONS_00139447	XLOC_070935	2	28671407	28671483	Inf	NA	5.00E-05	0.00621615		
TCONS_00139477	XLOC_070965	2	29196292	29196368	Inf	NA	5.00E-05	0.00621615		
TCONS_00139511	XLOC_070999	2	29523189	29525462	Inf	NA	5.00E-05	0.00621615		
TCONS_00139653	XLOC_071141	2	31214727	31214831	Inf	NA	5.00E-05	0.00621615		
TCONS_00140071	XLOC_071554	2	37829608	37829683	Inf	NA	5.00E-05	0.00621615		
TCONS_00140083	XLOC_071563	2	38228211	38228287	Inf	NA	5.00E-05	0.00621615	RGD1564606	
TCONS_00140324	XLOC_071804	2	40072801	40072961	Inf	NA	5.00E-05	0.00621615	Depdc1b	Transcription
TCONS_00140418	XLOC_071898	2	42661778	42662361	#NAME?	NA	5.00E-05	0.00621615		
TCONS_00140424	XLOC_071904	2	42671007	42671083	Inf	NA	5.00E-05	0.00621615		
TCONS_00140510	XLOC_071989	2	44328813	44328912	#NAME?	NA	5.00E-05	0.00621615	Il31ra	Receptor
TCONS_00140700	XLOC_072158	2	49756069	49756145	Inf	NA	5.00E-05	0.00621615		
TCONS_00140722	XLOC_072180	2	51034930	51035225	Inf	NA	5.00E-05	0.00621615	Mrps30	
TCONS_00140859	XLOC_072317	2	54368373	54369994	Inf	NA	5.00E-05	0.00621615	Plcxd3	
TCONS_00140865	XLOC_072323	2	54373077	54374123	Inf	NA	5.00E-05	0.00621615		
TCONS_00140906	XLOC_072364	2	55462936	55464464	#NAME?	NA	5.00E-05	0.00621615		
TCONS_00140911	XLOC_072369	2	55491194	55492361	#NAME?	NA	5.00E-05	0.00621615	SNORA17	
TCONS_00140914	XLOC_072372	2	55493630	55494990	#NAME?	NA	5.00E-05	0.00621615	SNORA17	
TCONS_00140916	XLOC_072374	2	55495699	55496094	#NAME?	NA	5.00E-05	0.00621615	SNORA17	
TCONS_00140918	XLOC_072376	2	55496568	55497764	#NAME?	NA	5.00E-05	0.00621615	SNORA17	
TCONS_00140922	XLOC_072380	2	55502552	55503076	#NAME?	NA	5.00E-05	0.00621615	SNORA17	
TCONS_00140923	XLOC_072381	2	55503149	55503556	#NAME?	NA	5.00E-05	0.00621615	SNORA17	
TCONS_00140925	XLOC_072383	2	55504382	55505457	#NAME?	NA	5.00E-05	0.00621615	SNORA17	
TCONS_00140926	XLOC_072384	2	55505514	55508036	#NAME?	NA	5.00E-05	0.00621615	SNORA17	
TCONS_00140929	XLOC_072387	2	55513338	55513857	#NAME?	NA	5.00E-05	0.00621615		
TCONS_00140933	XLOC_072391	2	55523918	55525449	#NAME?	NA	5.00E-05	0.00621615		
TCONS_00140939	XLOC_072397	2	55539905	55541062	#NAME?	NA	5.00E-05	0.00621615		
TCONS_00140948	XLOC_072406	2	55573279	55574074	#NAME?	NA	5.00E-05	0.00621615		
TCONS_00140952	XLOC_072410	2	55585611	55595386	-3.907	-3.22168	5.00E-05	0.00621615		
TCONS_00141118	XLOC_072575	2	60090432	60090767	#NAME?	NA	5.00E-05	0.00621615		
TCONS_00141190	XLOC_072647	2	62222949	62223067	Inf	NA	5.00E-05	0.00621615	Zfr	Transcription
TCONS_00141228	XLOC_072674	2	62855885	62855961	Inf	NA	5.00E-05	0.00621615		
TCONS_00141239	XLOC_072685	2	63420260	63420335	Inf	NA	5.00E-05	0.00621615		
TCONS_00141245	XLOC_072691	2	63442627	63442703	Inf	NA	5.00E-05	0.00621615		
TCONS_00141254	XLOC_072700	2	63506962	63507037	Inf	NA	5.00E-05	0.00621615		
TCONS_00141261	XLOC_072707	2	63532624	63532700	Inf	NA	5.00E-05	0.00621615		
TCONS_00141265	XLOC_072711	2	63571855	63571950	Inf	NA	5.00E-05	0.00621615		
TCONS_00141273	XLOC_072719	2	63630204	63630280	Inf	NA	5.00E-05	0.00621615		
TCONS_00141294	XLOC_072740	2	65279071	65279233	Inf	NA	5.00E-05	0.00621615		
TCONS_00141557	XLOC_073003	2	73653708	73653784	Inf	NA	5.00E-05	0.00621615		
TCONS_00141574	XLOC_073020	2	73680049	73680430	#NAME?	NA	5.00E-05	0.00621615		
TCONS_00141656	XLOC_073102	2	73987691	73988201	#NAME?	NA	5.00E-05	0.00621615		
TCONS_00141704	XLOC_073150	2	74068430	74068738	#NAME?	NA	5.00E-05	0.00621615		
TCONS_00141724	XLOC_073170	2	74135430	74136176	#NAME?	NA	5.00E-05	0.00621615		
TCONS_00141747	XLOC_073193	2	74196929	74197784	#NAME?	NA	5.00E-05	0.00621615		
TCONS_00141778	XLOC_073224	2	75877426	75877668	Inf	NA	5.00E-05	0.00621615		
TCONS_00141938	XLOC_073384	2	76952975	76953149	#NAME?	NA	5.00E-05	0.00621615		
TCONS_00141943	XLOC_073389	2	76971695	76971897	#NAME?	NA	5.00E-05	0.00621615		
TCONS_00141992	XLOC_073438	2	77559405	77559677	Inf	NA	5.00E-05	0.00621615		
TCONS_00142249	XLOC_073695	2	81853446	81853518	Inf	NA	5.00E-05	0.00621615		
TCONS_00142268	XLOC_073714	2	81955931	81956007	Inf	NA	5.00E-05	0.00621615		

TCONS_00142322	XLOC_073768	2	84260355	84260430	Inf	NA	5.00E-05	0.00621615	SNORA17	
TCONS_00142595	XLOC_074041	2	94686417	94686492	Inf	NA	5.00E-05	0.00621615	Zbtb10	Transcription
TCONS_00142622	XLOC_074064	2	95197986	95198176	-2.96911	-3.06401	5.00E-05	0.00621615		
TCONS_00142686	XLOC_074128	2	96817645	96817988	#NAME?	NA	5.00E-05	0.00621615		
TCONS_00142767	XLOC_074209	2	96985833	96985908	Inf	NA	5.00E-05	0.00621615		
TCONS_00142774	XLOC_074216	2	97001643	97001800	Inf	NA	5.00E-05	0.00621615		
TCONS_00142789	XLOC_074231	2	97055822	97055898	Inf	NA	5.00E-05	0.00621615	U6	
TCONS_00142793	XLOC_074235	2	97072074	97072161	Inf	NA	5.00E-05	0.00621615		
TCONS_00142798	XLOC_074240	2	97081222	97081298	Inf	NA	5.00E-05	0.00621615		
TCONS_00142805	XLOC_074247	2	97134829	97134905	Inf	NA	5.00E-05	0.00621615		
TCONS_00142877	XLOC_074319	2	99763635	99763711	Inf	NA	5.00E-05	0.00621615		
TCONS_00142910	XLOC_074352	2	100426325	100426843	#NAME?	NA	5.00E-05	0.00621615		
TCONS_00142930	XLOC_074372	2	100458913	100459102	#NAME?	NA	5.00E-05	0.00621615		
TCONS_00142955	XLOC_074397	2	100519383	100519942	#NAME?	NA	5.00E-05	0.00621615		
TCONS_00142965	XLOC_074407	2	100538996	100539301	#NAME?	NA	5.00E-05	0.00621615		
TCONS_00142979	XLOC_074421	2	100564663	100565754	#NAME?	NA	5.00E-05	0.00621615		
TCONS_00142995	XLOC_074437	2	100616708	100617495	#NAME?	NA	5.00E-05	0.00621615		
TCONS_00143003	XLOC_074445	2	100631234	100640562	-2.65262	-2.7555	5.00E-05	0.00621615		
TCONS_00143004	XLOC_074446	2	100640682	100642995	-2.75947	-2.69312	5.00E-05	0.00621615		
TCONS_00143061	XLOC_074503	2	102195727	102196238	Inf	NA	5.00E-05	0.00621615		
TCONS_00143094	XLOC_074536	2	102694291	102694451	Inf	NA	5.00E-05	0.00621615	Bhlhe22;Cyp7b1	Transcription;Metabolism
TCONS_00143108	XLOC_074550	2	102897967	102898441	#NAME?	NA	5.00E-05	0.00621615		
TCONS_00143180	XLOC_074622	2	103133678	103134302	#NAME?	NA	5.00E-05	0.00621615		
TCONS_00143326	XLOC_074763	2	104735400	104736157	#NAME?	NA	5.00E-05	0.00621615	Cp	Metabolism
TCONS_00143371	XLOC_074808	2	107214129	107214259	Inf	NA	5.00E-05	0.00621615		
TCONS_00143623	XLOC_074974	2	110434984	110435456	#NAME?	NA	5.00E-05	0.00621615		
TCONS_00144118	XLOC_075466	2	117810602	117811060	#NAME?	NA	5.00E-05	0.00621615		
TCONS_00144145	XLOC_075493	2	117836468	117836544	Inf	NA	5.00E-05	0.00621615		
TCONS_00144251	XLOC_075599	2	118372024	118372100	Inf	NA	5.00E-05	0.00621615		
TCONS_00144319	XLOC_075667	2	120707744	120708718	Inf	NA	5.00E-05	0.00621615		
TCONS_00144468	XLOC_075816	2	120904843	120905325	Inf	NA	5.00E-05	0.00621615		
TCONS_00144513	XLOC_075861	2	121041777	121042096	Inf	NA	5.00E-05	0.00621615		
TCONS_00144623	XLOC_075971	2	124416509	124416582	Inf	NA	5.00E-05	0.00621615		
TCONS_00144947	XLOC_076293	2	133553192	133553678	Inf	NA	5.00E-05	0.00621615		
TCONS_00144950	XLOC_076296	2	133557754	133558250	Inf	NA	5.00E-05	0.00621615		
TCONS_00145084	XLOC_076430	2	139625243	139625765	Inf	NA	5.00E-05	0.00621615		
TCONS_00145367	XLOC_076656	2	144948989	144949244	Inf	NA	5.00E-05	0.00621615		
TCONS_00145578	XLOC_076838	2	148147892	148148073	Inf	NA	5.00E-05	0.00621615		
TCONS_00145596	XLOC_076856	2	148846370	148846471	Inf	NA	5.00E-05	0.00621615	Erich6	
TCONS_00145750	XLOC_076996	2	151553024	151553151	Inf	NA	5.00E-05	0.00621615		
TCONS_00145780	XLOC_077026	2	153244315	153249414	Inf	NA	5.00E-05	0.00621615		
TCONS_00145891	XLOC_077137	2	155573930	155574564	Inf	NA	5.00E-05	0.00621615		
TCONS_00145977	XLOC_077223	2	156266770	156266846	Inf	NA	5.00E-05	0.00621615		
TCONS_00146364	XLOC_077583	2	166750661	166750737	Inf	NA	5.00E-05	0.00621615		
TCONS_00146447	XLOC_077666	2	171204085	171204164	Inf	NA	5.00E-05	0.00621615	Bche	Metabolism
TCONS_00146456	XLOC_077675	2	171219751	171220061	Inf	NA	5.00E-05	0.00621615		
TCONS_00146460	XLOC_077679	2	171232024	171232660	Inf	NA	5.00E-05	0.00621615		
TCONS_00146472	XLOC_077691	2	171263807	171264342	Inf	NA	5.00E-05	0.00621615		
TCONS_00146505	XLOC_077724	2	171511163	171511602	Inf	NA	5.00E-05	0.00621615		
TCONS_00146563	XLOC_077782	2	171600024	171600640	Inf	NA	5.00E-05	0.00621615		
TCONS_00146736	XLOC_077955	2	175583628	175583811	Inf	NA	5.00E-05	0.00621615		
TCONS_00146752	XLOC_077971	2	175677195	175677272	Inf	NA	5.00E-05	0.00621615		
TCONS_00146885	XLOC_078104	2	181793789	181793865	Inf	NA	5.00E-05	0.00621615	7SK	
TCONS_00146948	XLOC_078165	2	181976644	181976720	Inf	NA	5.00E-05	0.00621615		
TCONS_00147260	XLOC_078463	2	186735983	186736298	#NAME?	NA	5.00E-05	0.00621615	Rn60_2_1868.2	
TCONS_00147294	XLOC_078497	2	187194722	187195370	#NAME?	NA	5.00E-05	0.00621615	Rn60_2_1872.3	
TCONS_00147301	XLOC_078504	2	187202611	187202841	#NAME?	NA	5.00E-05	0.00621615	Rn60_2_1872.3	
TCONS_00147477	XLOC_078669	2	193242096	193242172	Inf	NA	5.00E-05	0.00621615		
TCONS_00147581	XLOC_078773	2	195074196	195075299	Inf	NA	5.00E-05	0.00621615		
TCONS_00148026	XLOC_079209	2	203889143	203890364	#NAME?	NA	5.00E-05	0.00621615		
TCONS_00148027	XLOC_079210	2	203890475	203890970	#NAME?	NA	5.00E-05	0.00621615		
TCONS_00148028	XLOC_079211	2	203892696	203893158	#NAME?	NA	5.00E-05	0.00621615		
TCONS_00148029	XLOC_079212	2	203893214	203893740	#NAME?	NA	5.00E-05	0.00621615		
TCONS_00148032	XLOC_079215	2	203897066	203897863	#NAME?	NA	5.00E-05	0.00621615		
TCONS_00148095	XLOC_079278	2	205768570	205768646	Inf	NA	5.00E-05	0.00621615		

TCONS_00148216	XLOC_079399	2	207260646	207260723	Inf	NA	5.00E-05	0.00621615	Fam19a3;Ppm1j	Signaling
TCONS_00148232	XLOC_079415	2	207692617	207692693	Inf	NA	5.00E-05	0.00621615		
TCONS_00148415	XLOC_079598	2	212950764	212951749	#NAME?	NA	5.00E-05	0.00621615		
TCONS_00148435	XLOC_079618	2	213058379	213058991	#NAME?	NA	5.00E-05	0.00621615		
TCONS_00148449	XLOC_079632	2	214768216	214768290	Inf	NA	5.00E-05	0.00621615		
TCONS_00148493	XLOC_079676	2	215315426	215315613	Inf	NA	5.00E-05	0.00621615		
TCONS_00148572	XLOC_079755	2	217345447	217345523	Inf	NA	5.00E-05	0.00621615		
TCONS_00148573	XLOC_079756	2	217345727	217345920	Inf	NA	5.00E-05	0.00621615		
TCONS_00148580	XLOC_079763	2	217359597	217360197	Inf	NA	5.00E-05	0.00621615		
TCONS_00148582	XLOC_079765	2	217364350	217364426	Inf	NA	5.00E-05	0.00621615		
TCONS_00148590	XLOC_079773	2	217389225	217389778	Inf	NA	5.00E-05	0.00621615		
TCONS_00148605	XLOC_079788	2	217408932	217409723	Inf	NA	5.00E-05	0.00621615		
TCONS_00148607	XLOC_079790	2	217411180	217412495	Inf	NA	5.00E-05	0.00621615		
TCONS_00148609	XLOC_079792	2	217413290	217414093	Inf	NA	5.00E-05	0.00621615		
TCONS_00148615	XLOC_079798	2	217420000	217421291	Inf	NA	5.00E-05	0.00621615		
TCONS_00148617	XLOC_079800	2	217423730	217424571	Inf	NA	5.00E-05	0.00621615		
TCONS_00148618	XLOC_079801	2	217424717	217426114	Inf	NA	5.00E-05	0.00621615		
TCONS_00148623	XLOC_079806	2	217431777	217433621	Inf	NA	5.00E-05	0.00621615		
TCONS_00148632	XLOC_079815	2	217446652	217447460	Inf	NA	5.00E-05	0.00621615		
TCONS_00148700	XLOC_079883	2	219489880	219489955	Inf	NA	5.00E-05	0.00621615	U1	
TCONS_00148861	XLOC_080044	2	225415114	225415190	Inf	NA	5.00E-05	0.00621615		
TCONS_00148894	XLOC_080074	2	225548093	225548224	Inf	NA	5.00E-05	0.00621615	Arhgap29	
TCONS_00148968	XLOC_080147	2	230162900	230163402	#NAME?	NA	5.00E-05	0.00621615		
TCONS_00149059	XLOC_080238	2	232140284	232140360	Inf	NA	5.00E-05	0.00621615	Tifa;Ap1ar	Apoptosis
TCONS_00149068	XLOC_080247	2	232352938	232354508	Inf	NA	5.00E-05	0.00621615		
TCONS_00149070	XLOC_080249	2	232355315	232355560	Inf	NA	5.00E-05	0.00621615		
TCONS_00149071	XLOC_080250	2	232355623	232356568	Inf	NA	5.00E-05	0.00621615		
TCONS_00149073	XLOC_080252	2	232358550	232358626	Inf	NA	5.00E-05	0.00621615		
TCONS_00149078	XLOC_080257	2	232362232	232362829	Inf	NA	5.00E-05	0.00621615		
TCONS_00149081	XLOC_080260	2	232365940	232366392	Inf	NA	5.00E-05	0.00621615	AABR07013302.1	
TCONS_00149082	XLOC_080261	2	232367129	232367891	Inf	NA	5.00E-05	0.00621615	AABR07013302.1	
TCONS_00149110	XLOC_080289	2	232485729	232486220	Inf	NA	5.00E-05	0.00621615		
TCONS_00149204	XLOC_080383	2	234756086	234756406	#NAME?	NA	5.00E-05	0.00621615		
TCONS_00149207	XLOC_080386	2	234758501	234759051	#NAME?	NA	5.00E-05	0.00621615		
TCONS_00149208	XLOC_080387	2	234759665	234760415	#NAME?	NA	5.00E-05	0.00621615		
TCONS_00149242	XLOC_080421	2	234814175	234814874	#NAME?	NA	5.00E-05	0.00621615		
TCONS_00149258	XLOC_080437	2	234842094	234842890	#NAME?	NA	5.00E-05	0.00621615		
TCONS_00149263	XLOC_080442	2	234901424	234901499	Inf	NA	5.00E-05	0.00621615		
TCONS_00149622	XLOC_080792	2	240973224	240973702	Inf	NA	5.00E-05	0.00621615		
TCONS_00149800	XLOC_080970	2	247178836	247178911	Inf	NA	5.00E-05	0.00621615		
TCONS_00149908	XLOC_081068	2	249541836	249541912	Inf	NA	5.00E-05	0.00621615		
TCONS_00149909	XLOC_081069	2	249544412	249544633	Inf	NA	5.00E-05	0.00621615		
TCONS_00149915	XLOC_081075	2	249580402	249580477	Inf	NA	5.00E-05	0.00621615		
TCONS_00150040	XLOC_081200	2	251908770	251908846	Inf	NA	5.00E-05	0.00621615	Wdr63	Cytoskeleton
TCONS_00150300	XLOC_081459	2	264905877	264905952	Inf	NA	5.00E-05	0.00621615	Lrrc7	Unknown
TCONS_00150319	XLOC_081478	2	266118980	266119154	Inf	NA	5.00E-05	0.00621615		
TCONS_00150328	XLOC_081487	2	266177851	266177960	Inf	NA	5.00E-05	0.00621615	Rpe65	Metabolism
TCONS_00158324	XLOC_084981	3	8531109	8531726	Inf	NA	5.00E-05	0.00621615	Gle1;Sptan1	Translation
TCONS_00162389	XLOC_086010	3	148046692	148057523	3.0261	3.10688	5.00E-05	0.00621615	Defb19;Defb21;Defb24;AC111428.1	Signaling
TCONS_00162408	XLOC_086015	3	148215539	148216718	-3.47914	-4.69719	5.00E-05	0.00621615	Id1	Transcription
TCONS_00162409	XLOC_086016	3	148234192	148245416	-3.53534	-3.27588	5.00E-05	0.00621615	Cox4i2;AABR07054329.1	Metabolism
TCONS_00162793	XLOC_086094	3	152857591	152864806	-4.32404	-4.05302	5.00E-05	0.00621615	Myl9	Cytoskeleton
TCONS_00164162	XLOC_086389	3	2683807	2689579	-1.70511	-2.76916	5.00E-05	0.00621615	Clic3;RGD1306215;Ptgds	Metabolism
TCONS_00164695	XLOC_086488	3	9035174	9038158	-3.16441	-2.70599	5.00E-05	0.00621615	Ier5l	
TCONS_00166422	XLOC_086865	3	72161188	72171078	-2.92779	-3.75934	5.00E-05	0.00621615	AC096003.1;U6;Serping1	Proteolysis
TCONS_00167793	XLOC_087286	3	114802135	114830655	-1.78965	-2.36118	5.00E-05	0.00621615	Slc30a4	Transport
TCONS_00168112	XLOC_087342	3	122913885	122920684	-2.96808	-2.91467	5.00E-05	0.00621615	Cpxm1;LOC100365450;AABR07053736.1	Protease

TCONS_00168587	XLOC_087426	3	142748673	142752325	-1.92257	-2.446	5.00E-05	0.00621615	Sstr4;Thbd	Receptor;Immune
TCONS_00168905	XLOC_087511	3	148948895	148955664	3.05242	3.14629	5.00E-05	0.00621615		
TCONS_00170162	XLOC_087839	3	1165664	1166510	#NAME?	NA	5.00E-05	0.00621615		
TCONS_00170166	XLOC_087843	3	1394928	1395125	Inf	NA	5.00E-05	0.00621615	Il36rn	Signaling
TCONS_00170221	XLOC_087898	3	2394362	2394438	Inf	NA	5.00E-05	0.00621615	Nrarp	
TCONS_00170319	XLOC_087996	3	5072071	5072923	#NAME?	NA	5.00E-05	0.00621615		
TCONS_00170331	XLOC_088008	3	5101678	5102935	#NAME?	NA	5.00E-05	0.00621615		
TCONS_00170335	XLOC_088012	3	5110869	5111778	#NAME?	NA	5.00E-05	0.00621615	Abo3	
TCONS_00170344	XLOC_088021	3	5380801	5381042	Inf	NA	5.00E-05	0.00621615	Abo2	Metabolism
TCONS_00170541	XLOC_088216	3	9524181	9529689	-2.60757	-2.88727	5.00E-05	0.00621615		
TCONS_00170557	XLOC_088232	3	9580088	9580375	#NAME?	NA	5.00E-05	0.00621615		
TCONS_00170569	XLOC_088244	3	9626420	9626867	#NAME?	NA	5.00E-05	0.00621615	RGD1311084	
TCONS_00170688	XLOC_088361	3	12425704	12426312	Inf	NA	5.00E-05	0.00621615		
TCONS_00170769	XLOC_088438	3	14854308	14854384	Inf	NA	5.00E-05	0.00621615		
TCONS_00171366	XLOC_088997	3	26943405	26944040	Inf	NA	5.00E-05	0.00621615		
TCONS_00171507	XLOC_089138	3	27124160	27124865	Inf	NA	5.00E-05	0.00621615		
TCONS_00171532	XLOC_089163	3	27170022	27171785	Inf	NA	5.00E-05	0.00621615		
TCONS_00171622	XLOC_089244	3	30182923	30183357	Inf	NA	5.00E-05	0.00621615		
TCONS_00171635	XLOC_089257	3	30204877	30206515	Inf	NA	5.00E-05	0.00621615		
TCONS_00171657	XLOC_089279	3	30581725	30582277	#NAME?	NA	5.00E-05	0.00621615		
TCONS_00172087	XLOC_089667	3	40736640	40738808	Inf	NA	5.00E-05	0.00621615		
TCONS_00172138	XLOC_089718	3	41281117	41281262	Inf	NA	5.00E-05	0.00621615		
TCONS_00172162	XLOC_089742	3	43133051	43133127	Inf	NA	5.00E-05	0.00621615		
TCONS_00172175	XLOC_089755	3	43219290	43219366	Inf	NA	5.00E-05	0.00621615	AABR07052247.1	
TCONS_00172243	XLOC_089823	3	43837342	43837560	Inf	NA	5.00E-05	0.00621615		
TCONS_00172246	XLOC_089826	3	43839936	43840495	Inf	NA	5.00E-05	0.00621615		
TCONS_00172260	XLOC_089840	3	43881629	43881705	Inf	NA	5.00E-05	0.00621615		
TCONS_00172264	XLOC_089844	3	43897913	43898113	Inf	NA	5.00E-05	0.00621615		
TCONS_00172265	XLOC_089845	3	43918789	43918864	Inf	NA	5.00E-05	0.00621615		
TCONS_00172267	XLOC_089847	3	43947672	43947750	Inf	NA	5.00E-05	0.00621615		
TCONS_00172374	XLOC_089933	3	47521031	47521107	Inf	NA	5.00E-05	0.00621615	Tank	Signaling
TCONS_00172487	XLOC_090042	3	53732542	53732618	Inf	NA	5.00E-05	0.00621615		
TCONS_00172539	XLOC_090094	3	54249473	54249549	Inf	NA	5.00E-05	0.00621615	B3galt1	Metabolism
TCONS_00172540	XLOC_090095	3	54317445	54317520	Inf	NA	5.00E-05	0.00621615		
TCONS_00172820	XLOC_090372	3	59704930	59705451	Inf	NA	5.00E-05	0.00621615		
TCONS_00173040	XLOC_090576	3	62588257	62588333	Inf	NA	5.00E-05	0.00621615		
TCONS_00173155	XLOC_090691	3	65038449	65038525	Inf	NA	5.00E-05	0.00621615	AABR07052598.1	
TCONS_00173222	XLOC_090757	3	66569328	66569752	#NAME?	NA	5.00E-05	0.00621615		
TCONS_00173711	XLOC_091243	3	82858945	82859489	#NAME?	NA	5.00E-05	0.00621615	RGD1564664	
TCONS_00173717	XLOC_091249	3	82922394	82922579	Inf	NA	5.00E-05	0.00621615	Hsd17b12	Metabolism
TCONS_00173742	XLOC_091274	3	84419975	84420051	Inf	NA	5.00E-05	0.00621615		
TCONS_00173890	XLOC_091422	3	91894709	91894785	Inf	NA	5.00E-05	0.00621615		
TCONS_00173914	XLOC_091445	3	92244554	92244776	Inf	NA	5.00E-05	0.00621615	Trim44	
TCONS_00173982	XLOC_091513	3	94334137	94334213	Inf	NA	5.00E-05	0.00621615		
TCONS_00174095	XLOC_091622	3	100076286	100076364	Inf	NA	5.00E-05	0.00621615		
TCONS_00174145	XLOC_091672	3	102461143	102461218	Inf	NA	5.00E-05	0.00621615	Olr748	
TCONS_00174306	XLOC_091784	3	105473523	105473634	Inf	NA	5.00E-05	0.00621615	Gjd2	
TCONS_00174310	XLOC_091788	3	105478246	105478399	Inf	NA	5.00E-05	0.00621615	Gjd2	
TCONS_00174313	XLOC_091791	3	105481971	105482916	Inf	NA	5.00E-05	0.00621615		
TCONS_00174518	XLOC_091991	3	112421560	112421973	Inf	NA	5.00E-05	0.00621615	Stard9	
TCONS_00174820	XLOC_092243	3	118471913	118472200	Inf	NA	5.00E-05	0.00621615		
TCONS_00174823	XLOC_092246	3	118637139	118637396	Inf	NA	5.00E-05	0.00621615	Atp8b4	Transport
TCONS_00175087	XLOC_092506	3	124181810	124181886	Inf	NA	5.00E-05	0.00621615		
TCONS_00175147	XLOC_092566	3	126189207	126189854	#NAME?	NA	5.00E-05	0.00621615	AABR07053787.2	
TCONS_00175152	XLOC_092571	3	126196713	126197385	#NAME?	NA	5.00E-05	0.00621615		
TCONS_00175154	XLOC_092573	3	126198207	126198831	#NAME?	NA	5.00E-05	0.00621615		
TCONS_00175159	XLOC_092578	3	126203659	126204310	#NAME?	NA	5.00E-05	0.00621615		
TCONS_00175163	XLOC_092582	3	126207873	126209426	#NAME?	NA	5.00E-05	0.00621615		
TCONS_00175164	XLOC_092583	3	126210220	126211276	#NAME?	NA	5.00E-05	0.00621615		
TCONS_00175167	XLOC_092586	3	126230293	126230806	#NAME?	NA	5.00E-05	0.00621615		
TCONS_00175168	XLOC_092587	3	126232404	126233123	#NAME?	NA	5.00E-05	0.00621615		
TCONS_00175200	XLOC_092619	3	127728820	127729144	Inf	NA	5.00E-05	0.00621615		
TCONS_00175546	XLOC_092864	3	130919408	130920236	Inf	NA	5.00E-05	0.00621615		
TCONS_00175564	XLOC_092881	3	130989747	130990206	Inf	NA	5.00E-05	0.00621615		

TCONS_00175885	XLOC_093200	3	135187601	135188125	#NAME?	NA	5.00E-05	0.00621615		
TCONS_00175937	XLOC_093252	3	135270777	135271304	#NAME?	NA	5.00E-05	0.00621615		
TCONS_00175969	XLOC_093284	3	135311035	135311485	#NAME?	NA	5.00E-05	0.00621615		
TCONS_00175985	XLOC_093300	3	135334987	135335643	#NAME?	NA	5.00E-05	0.00621615		
TCONS_00176000	XLOC_093315	3	135356625	135356700	Inf	NA	5.00E-05	0.00621615		
TCONS_00176059	XLOC_093374	3	135882519	135883055	#NAME?	NA	5.00E-05	0.00621615		
TCONS_00176098	XLOC_093413	3	135973492	135974295	#NAME?	NA	5.00E-05	0.00621615		
TCONS_00176119	XLOC_093434	3	136079848	136080921	#NAME?	NA	5.00E-05	0.00621615		
TCONS_00176516	XLOC_093830	3	140780927	140781292	Inf	NA	5.00E-05	0.00621615		
TCONS_00176527	XLOC_093841	3	140795997	140796381	Inf	NA	5.00E-05	0.00621615		
TCONS_00176652	XLOC_093966	3	143225436	143226261	Inf	NA	5.00E-05	0.00621615	Cst3	Proteolysis
TCONS_00176658	XLOC_093972	3	143236990	143237413	Inf	NA	5.00E-05	0.00621615		
TCONS_00176839	XLOC_094153	3	144374895	144375183	Inf	NA	5.00E-05	0.00621615		
TCONS_00176844	XLOC_094158	3	144423847	144424488	Inf	NA	5.00E-05	0.00621615	Cyss	
TCONS_00177095	XLOC_094404	3	147958739	147958813	Inf	NA	5.00E-05	0.00621615	Defb20;Defb22	Unknown
TCONS_00177146	XLOC_094453	3	148941682	148948565	2.62387	2.65849	5.00E-05	0.00621615	Nol4l	
TCONS_00177154	XLOC_094461	3	149028609	149029755	2.47186	2.59994	5.00E-05	0.00621615		
TCONS_00177373	XLOC_094662	3	154727176	154727724	Inf	NA	5.00E-05	0.00621615	AABR07054460.3	
TCONS_00177456	XLOC_094737	3	157166841	157166917	Inf	NA	5.00E-05	0.00621615		
TCONS_00177684	XLOC_094930	3	162883976	162884311	Inf	NA	5.00E-05	0.00621615		
TCONS_00177745	XLOC_094991	3	164367720	164367796	Inf	NA	5.00E-05	0.00621615	LOC679539;Tmem189	Metabolism
TCONS_00177777	XLOC_095023	3	164459062	164459728	Inf	NA	5.00E-05	0.00621615		
TCONS_00177783	XLOC_095029	3	164471428	164472267	Inf	NA	5.00E-05	0.00621615		
TCONS_00177883	XLOC_095125	3	165399965	165400104	Inf	NA	5.00E-05	0.00621615		
TCONS_00178224	XLOC_095419	3	171287193	171287268	Inf	NA	5.00E-05	0.00621615	Zbp1;Pmepa1	
TCONS_00178284	XLOC_095469	3	172051515	172051968	-4.21319	-3.44246	5.00E-05	0.00621615		
TCONS_00178405	XLOC_095590	3	174557452	174557500	Inf	NA	5.00E-05	0.00621615	AABR07054948.1	
TCONS_00178406	XLOC_095591	3	174562395	174562471	Inf	NA	5.00E-05	0.00621615	AABR07054948.1	
TCONS_00178569	XLOC_095754	3	175064243	175064896	Inf	NA	5.00E-05	0.00621615		
TCONS_00179743	XLOC_096192	4	44573263	44580638	-2.29951	-2.6265	5.00E-05	0.00621615	Cav2	Epigenetic
TCONS_00180190	XLOC_096283	4	62897946	62912332	5.88307	5.55443	5.00E-05	0.00621615		
TCONS_00180366	XLOC_096314	4	68395621	68397053	-2.24072	-2.44524	5.00E-05	0.00621615		
TCONS_00180674	XLOC_096413	4	78694446	78715683	-2.49706	-2.66693	5.00E-05	0.00621615	AC120721.1;Gpnmb	Cytoskeleton
TCONS_00181004	XLOC_096504	4	90990087	91038748	4.96218	4.09607	5.00E-05	0.00621615	Mmrn1	Extracellular Matrix
TCONS_00181755	XLOC_096700	4	120122445	120143000	-2.77714	-2.6386	5.00E-05	0.00621615	AABR07061382.2;Gata2	Transcription
TCONS_00182764	XLOC_096932	4	155313670	155336228	-4.43964	-2.97341	5.00E-05	0.00621615	Mfap5	Extracellular Matrix
TCONS_00182819	XLOC_096953	4	157125997	157136829	-2.80589	-3.44128	5.00E-05	0.00621615	C1rl;C1r;C1s	Protease;Immune
TCONS_00183208	XLOC_097045	4	166986349	166987306	Inf	NA	5.00E-05	0.00621615	Tas2r136;Tas2r117;Tas2r109	
TCONS_00183509	XLOC_097114	4	176993954	177014257	-1.72749	-2.57966	5.00E-05	0.00621615	Cmas;RGD1561551	Metabolism
TCONS_00185703	XLOC_097685	4	100061767	100112731	-3.08119	-2.57395	5.00E-05	0.00621615	Atoh8	Transcription
TCONS_00185967	XLOC_097777	4	115215059	115239723	-4.95903	-5.07736	5.00E-05	0.00621615	Dguok;Actg2;AC115473.1;Stambp	Signaling;Cytoskeleton;Receptor
TCONS_00187299	XLOC_098092	4	157977161	158010166	-3.29465	-3.54259	5.00E-05	0.00621615	Cd9	Extracellular Matrix
TCONS_00187744	XLOC_098243	4	170856750	170860225	-3.714	-5.08393	5.00E-05	0.00621615	Mgp	Development
TCONS_00187872	XLOC_098275	4	176701982	176720012	-2.90749	-4.31802	5.00E-05	0.00621615	Ldhb	Metabolism
TCONS_00188274	XLOC_098385	4	1131473	1131548	Inf	NA	5.00E-05	0.00621615		
TCONS_00188416	XLOC_098525	4	3579635	3580098	#NAME?	NA	5.00E-05	0.00621615		
TCONS_00188455	XLOC_098564	4	5091089	5092066	Inf	NA	5.00E-05	0.00621615		
TCONS_00188497	XLOC_098606	4	5355895	5357838	Inf	NA	5.00E-05	0.00621615		
TCONS_00188498	XLOC_098607	4	5357981	5361535	Inf	NA	5.00E-05	0.00621615		
TCONS_00188551	XLOC_098660	4	7055649	7055724	Inf	NA	5.00E-05	0.00621615	AC099360.1	
TCONS_00188720	XLOC_098827	4	8796512	8796795	#NAME?	NA	5.00E-05	0.00621615		
TCONS_00188761	XLOC_098868	4	8855340	8855828	#NAME?	NA	5.00E-05	0.00621615		
TCONS_00188812	XLOC_098919	4	9082056	9083139	Inf	NA	5.00E-05	0.00621615	U7	
TCONS_00189098	XLOC_099202	4	18176062	18176137	Inf	NA	5.00E-05	0.00621615	Sema3a	Growth Factors & Cytokines
TCONS_00189178	XLOC_099282	4	22428700	22429271	#NAME?	NA	5.00E-05	0.00621615	Abcb1a	Transport
TCONS_00189382	XLOC_099486	4	25186946	25187896	#NAME?	NA	5.00E-05	0.00621615		

TCONS_00189384	XLOC_099488	4	25203061	25204327	#NAME?	NA	5.00E-05	0.00621615		
TCONS_00189385	XLOC_099489	4	25205357	25205883	#NAME?	NA	5.00E-05	0.00621615		
TCONS_00189389	XLOC_099493	4	25217034	25217654	#NAME?	NA	5.00E-05	0.00621615		
TCONS_00189397	XLOC_099501	4	25237221	25237900	#NAME?	NA	5.00E-05	0.00621615		
TCONS_00189405	XLOC_099509	4	25249474	25250719	#NAME?	NA	5.00E-05	0.00621615		
TCONS_00189530	XLOC_099622	4	28077936	28078012	Inf	NA	5.00E-05	0.00621615		
TCONS_00189560	XLOC_099652	4	29181543	29181619	Inf	NA	5.00E-05	0.00621615		
TCONS_00189653	XLOC_099734	4	31810791	31810928	Inf	NA	5.00E-05	0.00621615		
TCONS_00189770	XLOC_099851	4	38474943	38475018	Inf	NA	5.00E-05	0.00621615		
TCONS_00189888	XLOC_099969	4	43111134	43111956	Inf	NA	5.00E-05	0.00621615		
TCONS_00189892	XLOC_099973	4	44420934	44421329	#NAME?	NA	5.00E-05	0.00621615		
TCONS_00189904	XLOC_099985	4	44562480	44562804	#NAME?	NA	5.00E-05	0.00621615		
TCONS_00190224	XLOC_100305	4	48524122	48524740	#NAME?	NA	5.00E-05	0.00621615		
TCONS_00190282	XLOC_100363	4	48617961	48618783	#NAME?	NA	5.00E-05	0.00621615		
TCONS_00190344	XLOC_100425	4	51612331	51612407	Inf	NA	5.00E-05	0.00621615	Asb15	Transcription
TCONS_00190436	XLOC_100513	4	57048814	57048888	Inf	NA	5.00E-05	0.00621615	Smo;Ahcyl2	Receptor;Metabolism
TCONS_00190552	XLOC_100622	4	59322793	59322868	Inf	NA	5.00E-05	0.00621615		
TCONS_00190591	XLOC_100661	4	61675386	61675461	Inf	NA	5.00E-05	0.00621615		
TCONS_00190685	XLOC_100751	4	62913988	62914276	Inf	NA	5.00E-05	0.00621615		
TCONS_00190688	XLOC_100754	4	62916537	62917205	Inf	NA	5.00E-05	0.00621615		
TCONS_00190692	XLOC_100758	4	62923699	62924273	Inf	NA	5.00E-05	0.00621615		
TCONS_00190695	XLOC_100761	4	62930581	62931217	Inf	NA	5.00E-05	0.00621615		
TCONS_00190700	XLOC_100766	4	62937159	62938136	Inf	NA	5.00E-05	0.00621615		
TCONS_00190729	XLOC_100795	4	62983184	62983954	Inf	NA	5.00E-05	0.00621615		
TCONS_00190740	XLOC_100806	4	63045160	63045954	Inf	NA	5.00E-05	0.00621615	Mtpn	Development
TCONS_00190952	XLOC_101014	4	67576675	67587188	4.00363	3.90536	5.00E-05	0.00621615		
TCONS_00191188	XLOC_101246	4	72114959	72115035	Inf	NA	5.00E-05	0.00621615	Tcaf1	
TCONS_00191357	XLOC_101413	4	78215042	78215117	Inf	NA	5.00E-05	0.00621615	Lrrc61;Rarres2	
TCONS_00191507	XLOC_101563	4	82011476	82011759	Inf	NA	5.00E-05	0.00621615		
TCONS_00191657	XLOC_101711	4	85615853	85615927	Inf	NA	5.00E-05	0.00621615		
TCONS_00191763	XLOC_101811	4	89448628	89448704	Inf	NA	5.00E-05	0.00621615		
TCONS_00191926	XLOC_101931	4	92054619	92054693	Inf	NA	5.00E-05	0.00621615		
TCONS_00192049	XLOC_102054	4	95971602	95971677	Inf	NA	5.00E-05	0.00621615	Hpgds	Metabolism
TCONS_00192057	XLOC_102062	4	95994576	95994652	Inf	NA	5.00E-05	0.00621615		
TCONS_00192177	XLOC_102177	4	98600993	98605366	3.61002	3.03817	5.00E-05	0.00621615	Rpia	
TCONS_00192219	XLOC_102219	4	99220071	99220432	Inf	NA	5.00E-05	0.00621615		
TCONS_00192374	XLOC_102371	4	100833939	100834273	Inf	NA	5.00E-05	0.00621615		
TCONS_00192402	XLOC_102399	4	101345296	101345909	#NAME?	NA	5.00E-05	0.00621615		
TCONS_00192403	XLOC_102400	4	101345965	101346393	#NAME?	NA	5.00E-05	0.00621615		
TCONS_00192516	XLOC_102513	4	105332496	105332951	Inf	NA	5.00E-05	0.00621615		
TCONS_00192517	XLOC_102514	4	105333485	105333560	Inf	NA	5.00E-05	0.00621615		
TCONS_00192681	XLOC_102678	4	109189978	109190455	Inf	NA	5.00E-05	0.00621615		
TCONS_00192722	XLOC_102719	4	109688703	109688815	Inf	NA	5.00E-05	0.00621615		
TCONS_00192723	XLOC_102720	4	109689007	109689389	Inf	NA	5.00E-05	0.00621615		
TCONS_00192724	XLOC_102721	4	109689685	109690349	Inf	NA	5.00E-05	0.00621615		
TCONS_00192728	XLOC_102725	4	109703852	109704241	Inf	NA	5.00E-05	0.00621615		
TCONS_00192733	XLOC_102730	4	109710116	109710891	Inf	NA	5.00E-05	0.00621615		
TCONS_00192746	XLOC_102743	4	109730140	109730215	Inf	NA	5.00E-05	0.00621615		
TCONS_00192748	XLOC_102745	4	109745685	109746196	Inf	NA	5.00E-05	0.00621615		
TCONS_00192751	XLOC_102748	4	109752385	109752460	Inf	NA	5.00E-05	0.00621615		
TCONS_00192752	XLOC_102749	4	109753216	109753440	Inf	NA	5.00E-05	0.00621615		
TCONS_00192791	XLOC_102788	4	112171600	112171676	Inf	NA	5.00E-05	0.00621615		
TCONS_00193010	XLOC_103001	4	118842602	118842861	Inf	NA	5.00E-05	0.00621615	Nfu1;Gfpt1	Metabolism
TCONS_00193041	XLOC_103032	4	119674381	119674510	Inf	NA	5.00E-05	0.00621615	Gp9	Extracellular Matrix
TCONS_00193141	XLOC_103131	4	121270536	121270612	Inf	NA	5.00E-05	0.00621615		
TCONS_00193208	XLOC_103198	4	123263329	123263790	#NAME?	NA	5.00E-05	0.00621615	SNORA17	
TCONS_00193265	XLOC_103255	4	124376497	124377118	#NAME?	NA	5.00E-05	0.00621615		
TCONS_00193389	XLOC_103364	4	124567172	124567805	#NAME?	NA	5.00E-05	0.00621615		
TCONS_00193459	XLOC_103414	4	125286166	125286242	Inf	NA	5.00E-05	0.00621615		
TCONS_00193845	XLOC_103791	4	142221696	142222008	Inf	NA	5.00E-05	0.00621615		
TCONS_00193869	XLOC_103815	4	144904584	144904907	#NAME?	NA	5.00E-05	0.00621615		
TCONS_00193985	XLOC_103921	4	149817413	149818218	#NAME?	NA	5.00E-05	0.00621615	AABR07061860.1	
TCONS_00193986	XLOC_103922	4	149818616	149819081	#NAME?	NA	5.00E-05	0.00621615	AABR07061860.1	

TCONS_00193988	XLOC_103924	4	149820085	149820328	#NAME?	NA	5.00E-05	0.00621615	AABR07061860.1	
TCONS_00193989	XLOC_103925	4	149820971	149821561	#NAME?	NA	5.00E-05	0.00621615	AABR07061860.1	
TCONS_00194030	XLOC_103966	4	150479073	150479148	Inf	NA	5.00E-05	0.00621615	Bms1;Zfp248;AABR07061874.1	Translation;Transcription
TCONS_00194068	XLOC_104002	4	150888404	150888877	#NAME?	NA	5.00E-05	0.00621615		
TCONS_00194103	XLOC_104034	4	150957608	150973856	-3.32883	-3.30728	5.00E-05	0.00621615		
TCONS_00194127	XLOC_104053	4	151044680	151053369	-3.10958	-3.09677	5.00E-05	0.00621615		
TCONS_00194130	XLOC_104056	4	151065272	151069846	-3.36525	-2.99412	5.00E-05	0.00621615		
TCONS_00194363	XLOC_104276	4	157163657	157166196	-2.88958	-2.75524	5.00E-05	0.00621615	C1s	Immune
TCONS_00194401	XLOC_104314	4	157903492	157903734	#NAME?	NA	5.00E-05	0.00621615	Plekhg6	Signaling
TCONS_00194605	XLOC_104516	4	162530536	162530611	Inf	NA	5.00E-05	0.00621615		
TCONS_00194746	XLOC_104656	4	166991831	166992404	Inf	NA	5.00E-05	0.00621615	Tas2r117;Tas2r109	
TCONS_00194907	XLOC_104809	4	170002167	170002525	Inf	NA	5.00E-05	0.00621615	Grin2b	Receptor
TCONS_00195007	XLOC_104909	4	170328399	170330292	Inf	NA	5.00E-05	0.00621615		
TCONS_00195009	XLOC_104911	4	170331636	170335297	Inf	NA	5.00E-05	0.00621615		
TCONS_00195235	XLOC_105131	4	177663216	177663824	Inf	NA	5.00E-05	0.00621615		
TCONS_00195326	XLOC_105211	4	178557546	178558119	#NAME?	NA	5.00E-05	0.00621615		
TCONS_00195363	XLOC_105234	4	178603150	178603625	#NAME?	NA	5.00E-05	0.00621615		
TCONS_00195547	XLOC_105400	4	179087840	179089152	Inf	NA	5.00E-05	0.00621615		
TCONS_00195549	XLOC_105402	4	179090490	179091379	Inf	NA	5.00E-05	0.00621615		
TCONS_00195553	XLOC_105406	4	179094460	179095312	Inf	NA	5.00E-05	0.00621615		
TCONS_00195652	XLOC_105505	4	182115320	182116356	Inf	NA	5.00E-05	0.00621615		
TCONS_00195665	XLOC_105518	4	182168946	182169397	Inf	NA	5.00E-05	0.00621615		
TCONS_00195684	XLOC_105537	4	182422945	182423441	Inf	NA	5.00E-05	0.00621615		
TCONS_00195686	XLOC_105539	4	182434946	182435482	Inf	NA	5.00E-05	0.00621615		
TCONS_00195687	XLOC_105540	4	182435873	182436275	Inf	NA	5.00E-05	0.00621615		
TCONS_00197292	XLOC_106034	5	79570396	79571036	Inf	NA	5.00E-05	0.00621615	Tnfsf15	Signaling
TCONS_00198764	XLOC_106371	5	140923913	140940905	-2.79067	-2.72706	5.00E-05	0.00621615	Heyl	Transcription
TCONS_00199417	XLOC_106512	5	152559083	152572156	-3.18186	-3.08893	5.00E-05	0.00621615	Slc30a2;Extl1	Metabolism
TCONS_00199536	XLOC_106538	5	154489254	154491348	-2.72642	-3.71729	5.00E-05	0.00621615	U6;Id3	Transcription
TCONS_00199557	XLOC_106541	5	154598757	154634686	2.86877	2.87724	5.00E-05	0.00621615	Asap3;Tcea3	Transcription
TCONS_00199626	XLOC_106555	5	155885985	155913944	-2.88845	-3.27833	5.00E-05	0.00621615	AABR07073181.1;AABR07073181.2;Ldlrad2	Receptor
TCONS_00199760	XLOC_106565	5	156800355	156801890	#NAME?	NA	5.00E-05	0.00621615	Fam43b;AABR07050222.1	
TCONS_00199795	XLOC_106574	5	157282668	157285328	-3.23746	-3.80079	5.00E-05	0.00621615	U1;Pla2g2a	Signaling
TCONS_00200786	XLOC_106792	5	3736288	3737678	Inf	NA	5.00E-05	0.00621615		
TCONS_00201322	XLOC_106912	5	33843518	33892462	-2.36529	-2.56947	5.00E-05	0.00621615	Atp6v0d2	Transport
TCONS_00201676	XLOC_107010	5	58197679	58198782	5.32861	6.02039	5.00E-05	0.00621615	Ccl21	Growth Factors & Cytokines
TCONS_00201734	XLOC_107023	5	58994681	58994953	1.69116	2.42254	5.00E-05	0.00621615	Sit1;RNase_MRP;Ccdc107;Arhgef39	
TCONS_00201739	XLOC_107025	5	59015888	59025631	-3.69041	-4.23637	5.00E-05	0.00621615	Car9;Tpm2;Tln1	Metabolism;Cytoskeleton;Extracellular Matrix
TCONS_00202495	XLOC_107194	5	78283129	78292353	3.06654	2.74053	5.00E-05	0.00621615	Prpf4;Rnf183	Transcription
TCONS_00202817	XLOC_107270	5	104129257	104135898	-2.97838	-2.87792	5.00E-05	0.00621615		
TCONS_00202843	XLOC_107278	5	105209845	105212173	#NAME?	NA	5.00E-05	0.00621615	Rps6	Translation
TCONS_00202844	XLOC_107279	5	105230108	105279777	-3.0991	-2.98954	5.00E-05	0.00621615	Acer2	Metabolism
TCONS_00204545	XLOC_107698	5	152281132	152326789	-2.5734	-3.18851	5.00E-05	0.00621615	Aim1l;Cd52;Ftl1;AC120071.1;Ubxn11	Unknown;Proteolysis
TCONS_00205718	XLOC_107936	5	173216740	173222505	-2.17364	-2.65864	5.00E-05	0.00621615	Atad3a;Vwa1;Tmem88b	Metabolism;Extracellular Matrix;Unknown
TCONS_00205888	XLOC_108025	5	987998	988074	Inf	NA	5.00E-05	0.00621615		
TCONS_00205915	XLOC_108052	5	1629255	1629329	Inf	NA	5.00E-05	0.00621615		
TCONS_00205922	XLOC_108059	5	1651379	1652043	Inf	NA	5.00E-05	0.00621615		
TCONS_00206007	XLOC_108140	5	2518036	2518181	Inf	NA	5.00E-05	0.00621615		
TCONS_00206332	XLOC_108463	5	6917727	6918284	Inf	NA	5.00E-05	0.00621615		
TCONS_00206337	XLOC_108468	5	6923744	6924325	Inf	NA	5.00E-05	0.00621615		

TCONS_00206360	XLOC_108491	5	8217077	8217153	Inf	NA	5.00E-05	0.00621615		
TCONS_00206408	XLOC_108539	5	8346007	8346549	#NAME?	NA	5.00E-05	0.00621615		
TCONS_00206690	XLOC_108818	5	15685373	15685448	Inf	NA	5.00E-05	0.00621615		
TCONS_00206762	XLOC_108890	5	17464928	17465628	Inf	NA	5.00E-05	0.00621615		
TCONS_00206878	XLOC_108997	5	19860628	19860986	Inf	NA	5.00E-05	0.00621615		
TCONS_00206936	XLOC_109055	5	20970221	20970558	Inf	NA	5.00E-05	0.00621615		
TCONS_00206955	XLOC_109074	5	20987288	20987854	Inf	NA	5.00E-05	0.00621615		
TCONS_00206956	XLOC_109075	5	20995540	20996213	Inf	NA	5.00E-05	0.00621615		
TCONS_00206974	XLOC_109093	5	21016245	21016754	Inf	NA	5.00E-05	0.00621615		
TCONS_00207423	XLOC_109530	5	30123166	30123242	Inf	NA	5.00E-05	0.00621615		
TCONS_00207526	XLOC_109633	5	35462718	35463048	Inf	NA	5.00E-05	0.00621615		
TCONS_00208125	XLOC_110217	5	59320603	59321522	#NAME?	NA	5.00E-05	0.00621615	Olr839;Olr840	Receptor
TCONS_00208268	XLOC_110342	5	62036385	62043547	Inf	NA	5.00E-05	0.00621615		
TCONS_00208283	XLOC_110357	5	62788464	62788540	Inf	NA	5.00E-05	0.00621615		
TCONS_00208531	XLOC_110605	5	70133186	70133796	Inf	NA	5.00E-05	0.00621615		
TCONS_00208682	XLOC_110756	5	73359093	73359169	Inf	NA	5.00E-05	0.00621615		
TCONS_00208708	XLOC_110782	5	74228149	74228226	Inf	NA	5.00E-05	0.00621615		
TCONS_00208789	XLOC_110863	5	75769515	75769591	Inf	NA	5.00E-05	0.00621615		
TCONS_00208834	XLOC_110908	5	75846014	75847182	Inf	NA	5.00E-05	0.00621615		
TCONS_00208958	XLOC_111032	5	78090918	78090994	Inf	NA	5.00E-05	0.00621615		
TCONS_00208969	XLOC_111043	5	78293116	78293192	Inf	NA	5.00E-05	0.00621615	Rnf183	
TCONS_00208974	XLOC_111048	5	78327607	78327683	Inf	NA	5.00E-05	0.00621615	Wdr31;Bspry	Unknown;Development
TCONS_00208983	XLOC_111057	5	78708482	78708558	Inf	NA	5.00E-05	0.00621615		
TCONS_00209006	XLOC_111079	5	79921956	79922032	Inf	NA	5.00E-05	0.00621615	AC229945.2	
TCONS_00209072	XLOC_111145	5	83655472	83655969	Inf	NA	5.00E-05	0.00621615	RGD1561195	Translation
TCONS_00209149	XLOC_111222	5	88945656	88945731	Inf	NA	5.00E-05	0.00621615		
TCONS_00209531	XLOC_111604	5	103824550	103829595	-2.74701	-2.60585	5.00E-05	0.00621615		
TCONS_00209570	XLOC_111643	5	103901405	103901962	#NAME?	NA	5.00E-05	0.00621615		
TCONS_00209587	XLOC_111660	5	103934793	103935126	#NAME?	NA	5.00E-05	0.00621615		
TCONS_00209589	XLOC_111662	5	103936583	103937077	#NAME?	NA	5.00E-05	0.00621615		
TCONS_00209663	XLOC_111736	5	104109105	104117307	-2.91578	-2.76574	5.00E-05	0.00621615		
TCONS_00209716	XLOC_111789	5	104240428	104240694	#NAME?	NA	5.00E-05	0.00621615		
TCONS_00209725	XLOC_111798	5	104255215	104255768	#NAME?	NA	5.00E-05	0.00621615		
TCONS_00209759	XLOC_111832	5	104299450	104299835	#NAME?	NA	5.00E-05	0.00621615		
TCONS_00209828	XLOC_111901	5	105220127	105221083	#NAME?	NA	5.00E-05	0.00621615	Rps6;Acer2	Translation;Metabolism
TCONS_00209842	XLOC_111915	5	105287245	105287997	#NAME?	NA	5.00E-05	0.00621615	Acer2	Metabolism
TCONS_00209843	XLOC_111916	5	105288227	105288621	#NAME?	NA	5.00E-05	0.00621615	Acer2	Metabolism
TCONS_00210144	XLOC_112198	5	112305830	112305906	Inf	NA	5.00E-05	0.00621615		
TCONS_00210171	XLOC_112225	5	112413294	112413370	Inf	NA	5.00E-05	0.00621615		
TCONS_00210320	XLOC_112374	5	113409624	113409700	Inf	NA	5.00E-05	0.00621615	7SK	
TCONS_00210481	XLOC_112532	5	116940475	116940551	Inf	NA	5.00E-05	0.00621615	SNORA9	
TCONS_00210551	XLOC_112602	5	118730419	118731080	Inf	NA	5.00E-05	0.00621615	Efcab7	Metabolism
TCONS_00210729	XLOC_112779	5	125190451	125191254	Inf	NA	5.00E-05	0.00621615	U2	
TCONS_00210737	XLOC_112787	5	125202271	125202679	Inf	NA	5.00E-05	0.00621615		
TCONS_00210777	XLOC_112827	5	125614898	125614973	Inf	NA	5.00E-05	0.00621615		
TCONS_00210848	XLOC_112897	5	127115526	127115765	#NAME?	NA	5.00E-05	0.00621615	AABR07049535.2	
TCONS_00210881	XLOC_112930	5	127949347	127950036	#NAME?	NA	5.00E-05	0.00621615		
TCONS_00210887	XLOC_112936	5	127955718	127956149	#NAME?	NA	5.00E-05	0.00621615		
TCONS_00211638	XLOC_113633	5	141978403	141978766	Inf	NA	5.00E-05	0.00621615		
TCONS_00211831	XLOC_113826	5	143869611	143870068	Inf	NA	5.00E-05	0.00621615		
TCONS_00211838	XLOC_113833	5	143880772	143881396	Inf	NA	5.00E-05	0.00621615		
TCONS_00212041	XLOC_114026	5	147021521	147021597	Inf	NA	5.00E-05	0.00621615		
TCONS_00212261	XLOC_114232	5	150626064	150628195	3.11127	3.31083	5.00E-05	0.00621615		
TCONS_00212451	XLOC_114422	5	154479843	154480213	#NAME?	NA	5.00E-05	0.00621615	U6;Id3	Transcription
TCONS_00212454	XLOC_114425	5	154494103	154494600	#NAME?	NA	5.00E-05	0.00621615	Id3	Transcription
TCONS_00212634	XLOC_114604	5	158494113	158494449	Inf	NA	5.00E-05	0.00621615	Igsf21	Immune
TCONS_00212833	XLOC_114800	5	160965671	160965746	Inf	NA	5.00E-05	0.00621615		
TCONS_00213149	XLOC_115113	5	166740647	166740723	Inf	NA	5.00E-05	0.00621615		
TCONS_00213204	XLOC_115162	5	167751136	167751810	#NAME?	NA	5.00E-05	0.00621615		
TCONS_00213613	XLOC_115561	5	172818427	172818503	Inf	NA	5.00E-05	0.00621615	Gabrd;Cfap74	Receptor
TCONS_00213657	XLOC_115604	5	173357825	173357901	Inf	NA	5.00E-05	0.00621615	Acap3	Transcription
TCONS_00215413	XLOC_116004	6	66943662	66944825	Inf	NA	5.00E-05	0.00621615		
TCONS_00215476	XLOC_116027	6	72359662	72374713	-3.12086	-3.37662	5.00E-05	0.00621615	Coch;Strn3	Extracellular Matrix;Unknown

TCONS_00218324	XLOC_116703	6	11280380	11298613	-1.76936	-2.56925	5.00E-05	0.00621615	Msh2;Epcam;U5	Transcription
TCONS_00218802	XLOC_116790	6	27328405	27460038	5.96412	5.95999	5.00E-05	0.00621615	RGD1559683;Otof;Drc1	Transport
TCONS_00218981	XLOC_116835	6	33699320	33739678	-4.15888	-4.33442	5.00E-05	0.00621615	Rhob;Slc7a15	Signaling
TCONS_00219093	XLOC_116878	6	43123865	43126173	-4.18327	-4.34171	5.00E-05	0.00621615		
TCONS_00219857	XLOC_117101	6	91455275	91456703	-1.90628	-2.92861	5.00E-05	0.00621615	Rps29;Rn7sl1;AABR07064716.3;AABR07064716.2;Lrr1	Translation
TCONS_00221204	XLOC_117410	6	128749155	128749430	2.51732	3.39811	5.00E-05	0.00621615	Rpl6-ps1;SCARNA13;Glrx5	Metabolism
TCONS_00221311	XLOC_117435	6	133403787	133408286	Inf	NA	5.00E-05	0.00621615		
TCONS_00221745	XLOC_117614	6	1212873	1213137	Inf	NA	5.00E-05	0.00621615	Vit	Extracellular Matrix
TCONS_00221804	XLOC_117673	6	3211123	3211198	Inf	NA	5.00E-05	0.00621615		
TCONS_00221869	XLOC_117738	6	4081679	4097454	-2.50764	-2.77381	5.00E-05	0.00621615		
TCONS_00221880	XLOC_117749	6	4133549	4142564	-2.98851	-2.82745	5.00E-05	0.00621615		
TCONS_00221892	XLOC_117761	6	4176445	4176759	#NAME?	NA	5.00E-05	0.00621615		
TCONS_00221902	XLOC_117771	6	4215379	4215916	#NAME?	NA	5.00E-05	0.00621615		
TCONS_00221963	XLOC_117832	6	5700469	5701324	#NAME?	NA	5.00E-05	0.00621615		
TCONS_00222104	XLOC_117973	6	6027474	6028099	#NAME?	NA	5.00E-05	0.00621615		
TCONS_00222209	XLOC_118078	6	6267276	6267498	#NAME?	NA	5.00E-05	0.00621615		
TCONS_00222325	XLOC_118192	6	7335059	7335945	#NAME?	NA	5.00E-05	0.00621615		
TCONS_00222505	XLOC_118372	6	9114392	9114665	Inf	NA	5.00E-05	0.00621615		
TCONS_00222508	XLOC_118375	6	9118557	9119204	Inf	NA	5.00E-05	0.00621615		
TCONS_00222526	XLOC_118393	6	9132681	9133466	Inf	NA	5.00E-05	0.00621615		
TCONS_00222538	XLOC_118405	6	9142068	9143328	Inf	NA	5.00E-05	0.00621615		
TCONS_00222544	XLOC_118411	6	9199575	9199651	Inf	NA	5.00E-05	0.00621615		
TCONS_00222621	XLOC_118460	6	9711864	9736025	2.0323	2.55982	5.00E-05	0.00621615		
TCONS_00222804	XLOC_118643	6	17537395	17537470	Inf	NA	5.00E-05	0.00621615		
TCONS_00222930	XLOC_118769	6	20702792	20703672	Inf	NA	5.00E-05	0.00621615		
TCONS_00222951	XLOC_118790	6	20750324	20750936	Inf	NA	5.00E-05	0.00621615		
TCONS_00223058	XLOC_118893	6	23597111	23597618	#NAME?	NA	5.00E-05	0.00621615		
TCONS_00223064	XLOC_118899	6	23712825	23712901	Inf	NA	5.00E-05	0.00621615		
TCONS_00223267	XLOC_119102	6	27144854	27145652	#NAME?	NA	5.00E-05	0.00621615	Kcnk3	Transport
TCONS_00223823	XLOC_119656	6	33752614	33753293	#NAME?	NA	5.00E-05	0.00621615		
TCONS_00223825	XLOC_119658	6	33754956	33755336	#NAME?	NA	5.00E-05	0.00621615		
TCONS_00223830	XLOC_119663	6	33771074	33772113	#NAME?	NA	5.00E-05	0.00621615		
TCONS_00223833	XLOC_119666	6	33774728	33775447	#NAME?	NA	5.00E-05	0.00621615		
TCONS_00223851	XLOC_119684	6	34176905	34178005	#NAME?	NA	5.00E-05	0.00621615		
TCONS_00223857	XLOC_119690	6	34250567	34250642	Inf	NA	5.00E-05	0.00621615		
TCONS_00223872	XLOC_119705	6	34864177	34864253	Inf	NA	5.00E-05	0.00621615		
TCONS_00223949	XLOC_119782	6	36486133	36486209	Inf	NA	5.00E-05	0.00621615		
TCONS_00223954	XLOC_119787	6	36576441	36576517	Inf	NA	5.00E-05	0.00621615		
TCONS_00223976	XLOC_119809	6	37799215	37799756	Inf	NA	5.00E-05	0.00621615		
TCONS_00224023	XLOC_119856	6	38149184	38149718	Inf	NA	5.00E-05	0.00621615		
TCONS_00224027	XLOC_119860	6	38153980	38155300	Inf	NA	5.00E-05	0.00621615		
TCONS_00224041	XLOC_119874	6	38170165	38170882	Inf	NA	5.00E-05	0.00621615		
TCONS_00224160	XLOC_119988	6	39503356	39503432	Inf	NA	5.00E-05	0.00621615	AABR07063649.3	
TCONS_00224341	XLOC_120169	6	41400441	41401485	#NAME?	NA	5.00E-05	0.00621615		
TCONS_00224342	XLOC_120170	6	41404664	41405050	#NAME?	NA	5.00E-05	0.00621615		
TCONS_00224343	XLOC_120171	6	41406422	41406998	#NAME?	NA	5.00E-05	0.00621615		
TCONS_00224347	XLOC_120175	6	41413542	41414005	#NAME?	NA	5.00E-05	0.00621615		
TCONS_00224356	XLOC_120184	6	41427421	41428153	#NAME?	NA	5.00E-05	0.00621615		
TCONS_00224357	XLOC_120185	6	41428608	41429599	#NAME?	NA	5.00E-05	0.00621615		
TCONS_00224361	XLOC_120189	6	41433790	41434720	#NAME?	NA	5.00E-05	0.00621615		
TCONS_00224369	XLOC_120197	6	41449721	41449995	#NAME?	NA	5.00E-05	0.00621615		
TCONS_00224371	XLOC_120199	6	41452008	41453263	#NAME?	NA	5.00E-05	0.00621615		
TCONS_00224376	XLOC_120204	6	41457143	41458487	#NAME?	NA	5.00E-05	0.00621615	AABR07063674.2	
TCONS_00224378	XLOC_120206	6	41462608	41463387	#NAME?	NA	5.00E-05	0.00621615	AABR07063674.2	
TCONS_00224384	XLOC_120212	6	41472155	41472834	#NAME?	NA	5.00E-05	0.00621615	AABR07063674.2	
TCONS_00224390	XLOC_120218	6	41481530	41482677	#NAME?	NA	5.00E-05	0.00621615		

TCONS_00224476	XLOC_120299	6	43952952	43953251	#NAME?	NA	5.00E-05	0.00621615		
TCONS_00224599	XLOC_120421	6	44728912	44728988	Inf	NA	5.00E-05	0.00621615	AABR07063740.2	
TCONS_00224600	XLOC_120422	6	44730729	44730899	Inf	NA	5.00E-05	0.00621615	AABR07063740.2	
TCONS_00224659	XLOC_120481	6	47362341	47364053	Inf	NA	5.00E-05	0.00621615		
TCONS_00224666	XLOC_120488	6	47367999	47369286	Inf	NA	5.00E-05	0.00621615		
TCONS_00224674	XLOC_120496	6	47377702	47378269	Inf	NA	5.00E-05	0.00621615		
TCONS_00224675	XLOC_120497	6	47378341	47379487	Inf	NA	5.00E-05	0.00621615		
TCONS_00224676	XLOC_120498	6	47379978	47381555	Inf	NA	5.00E-05	0.00621615		
TCONS_00224677	XLOC_120499	6	47382229	47383578	Inf	NA	5.00E-05	0.00621615		
TCONS_00224681	XLOC_120503	6	47386796	47387233	Inf	NA	5.00E-05	0.00621615		
TCONS_00224712	XLOC_120534	6	47430691	47430768	Inf	NA	5.00E-05	0.00621615		
TCONS_00224873	XLOC_120695	6	49791651	49793243	Inf	NA	5.00E-05	0.00621615		
TCONS_00224880	XLOC_120702	6	49800196	49802755	Inf	NA	5.00E-05	0.00621615		
TCONS_00224883	XLOC_120705	6	49806699	49807793	Inf	NA	5.00E-05	0.00621615		
TCONS_00224928	XLOC_120750	6	49986764	49987518	Inf	NA	5.00E-05	0.00621615		
TCONS_00225832	XLOC_121610	6	62727385	62727461	Inf	NA	5.00E-05	0.00621615		
TCONS_00225853	XLOC_121631	6	64125278	64125354	Inf	NA	5.00E-05	0.00621615	5S_rRNA	
TCONS_00225966	XLOC_121731	6	65756050	65756680	Inf	NA	5.00E-05	0.00621615		
TCONS_00226017	XLOC_121782	6	66945070	66945146	Inf	NA	5.00E-05	0.00621615		
TCONS_00226261	XLOC_122026	6	72767565	72767641	Inf	NA	5.00E-05	0.00621615		
TCONS_00226301	XLOC_122066	6	74171235	74171309	Inf	NA	5.00E-05	0.00621615		
TCONS_00226307	XLOC_122072	6	74419263	74419338	Inf	NA	5.00E-05	0.00621615		
TCONS_00226652	XLOC_122397	6	86311376	86311452	Inf	NA	5.00E-05	0.00621615		
TCONS_00226736	XLOC_122481	6	87547817	87548095	Inf	NA	5.00E-05	0.00621615		
TCONS_00226774	XLOC_122519	6	89000456	89000534	Inf	NA	5.00E-05	0.00621615		
TCONS_00226821	XLOC_122566	6	89191118	89191198	Inf	NA	5.00E-05	0.00621615		
TCONS_00226874	XLOC_122619	6	89288583	89288658	Inf	NA	5.00E-05	0.00621615		
TCONS_00227004	XLOC_122749	6	92764084	92764406	#NAME?	NA	5.00E-05	0.00621615	Trim9	Unknown
TCONS_00227317	XLOC_123062	6	97313770	97313882	Inf	NA	5.00E-05	0.00621615		
TCONS_00227421	XLOC_123165	6	101167561	101168547	Inf	NA	5.00E-05	0.00621615		
TCONS_00227619	XLOC_123341	6	102892365	102892921	#NAME?	NA	5.00E-05	0.00621615		
TCONS_00227771	XLOC_123486	6	105265762	105266996	-3.08883	-4.03142	5.00E-05	0.00621615	Synj2bp	Receptor
TCONS_00228071	XLOC_123769	6	108271673	108271749	Inf	NA	5.00E-05	0.00621615		
TCONS_00228226	XLOC_123924	6	113206430	113206505	Inf	NA	5.00E-05	0.00621615	Nrxn3	Receptor
TCONS_00228260	XLOC_123958	6	115699107	115699183	Inf	NA	5.00E-05	0.00621615		
TCONS_00228332	XLOC_124030	6	118004719	118004924	Inf	NA	5.00E-05	0.00621615		
TCONS_00228382	XLOC_124080	6	119299448	119299524	Inf	NA	5.00E-05	0.00621615		
TCONS_00228496	XLOC_124194	6	123592393	123592981	#NAME?	NA	5.00E-05	0.00621615		
TCONS_00228627	XLOC_124319	6	126386568	126386644	Inf	NA	5.00E-05	0.00621615	Golga5	Golgi
TCONS_00228674	XLOC_124357	6	127359455	127360037	#NAME?	NA	5.00E-05	0.00621615		
TCONS_00228767	XLOC_124450	6	131492901	131493348	Inf	NA	5.00E-05	0.00621615		
TCONS_00228986	XLOC_124656	6	135426485	135427061	Inf	NA	5.00E-05	0.00621615		
TCONS_00229037	XLOC_124707	6	135916771	135916846	Inf	NA	5.00E-05	0.00621615		
TCONS_00229176	XLOC_124844	6	138051909	138052368	Inf	NA	5.00E-05	0.00621615	Ighm	
TCONS_00230576	XLOC_125327	7	28066634	28129769	-1.94541	-2.79513	5.00E-05	0.00621615	Pah	Metabolism
TCONS_00230851	XLOC_125389	7	38742050	38782401	-3.30646	-4.18579	5.00E-05	0.00621615	Dcn	Cytoskeleton
TCONS_00232685	XLOC_125839	7	120153183	120156289	-2.63011	-3.13866	5.00E-05	0.00621615	Pdxp;Lgals1;Nol12	Signaling
TCONS_00233695	XLOC_126058	7	141237767	141245165	-3.67153	-3.69107	5.00E-05	0.00621615	Aqp2;Aqp5	Metabolism;Transport
TCONS_00233946	XLOC_126112	7	143749220	143754054	-1.86874	-2.71423	5.00E-05	0.00621615	Igfbp6;Soat2	Receptor;Metabolism
TCONS_00234068	XLOC_126136	7	144612325	144631762	-2.63836	-3.2366	5.00E-05	0.00621615	Hoxc8;Hoxc6;Hoxc5;Mir615	Transcription;Development
TCONS_00234072	XLOC_126137	7	144631850	144640222	-2.48234	-2.77525	5.00E-05	0.00621615	Hoxc6;Hoxc5;Mir615;Hoxc4	Transcription;Development
TCONS_00234338	XLOC_126234	7	7310312	7311272	Inf	NA	5.00E-05	0.00621615	Olr1020	
TCONS_00236329	XLOC_126703	7	70846079	70926957	-2.61311	-3.33098	5.00E-05	0.00621615	Nxph4;Lrp1	Signaling;Receptor
TCONS_00236807	XLOC_126820	7	98059812	98098370	-2.16234	-2.49065	5.00E-05	0.00621615	Fbxo32	Transcription
TCONS_00239403	XLOC_127623	7	7201467	7203349	Inf	NA	5.00E-05	0.00621615		
TCONS_00239410	XLOC_127630	7	7210427	7211136	Inf	NA	5.00E-05	0.00621615		
TCONS_00239419	XLOC_127639	7	7216777	7218275	Inf	NA	5.00E-05	0.00621615		
TCONS_00239421	XLOC_127641	7	7219472	7221013	Inf	NA	5.00E-05	0.00621615		
TCONS_00239436	XLOC_127656	7	7243198	7244603	Inf	NA	5.00E-05	0.00621615		
TCONS_00239438	XLOC_127658	7	7248164	7249118	Inf	NA	5.00E-05	0.00621615		

TCONS_00239445	XLOC_127665	7	7257553	7258518	Inf	NA	5.00E-05	0.00621615		
TCONS_00239450	XLOC_127670	7	7260844	7260969	Inf	NA	5.00E-05	0.00621615		
TCONS_00239453	XLOC_127673	7	7264098	7264620	Inf	NA	5.00E-05	0.00621615		
TCONS_00239455	XLOC_127675	7	7266915	7267733	Inf	NA	5.00E-05	0.00621615		
TCONS_00239457	XLOC_127677	7	7272045	7272677	Inf	NA	5.00E-05	0.00621615		
TCONS_00239461	XLOC_127681	7	7277715	7284661	Inf	NA	5.00E-05	0.00621615	AABR07055631.1	
TCONS_00239464	XLOC_127684	7	7293669	7295445	Inf	NA	5.00E-05	0.00621615	AABR07055631.1	
TCONS_00239536	XLOC_127756	7	9883842	9883918	Inf	NA	5.00E-05	0.00621615		
TCONS_00240053	XLOC_128271	7	20169350	20169445	Inf	NA	5.00E-05	0.00621615		
TCONS_00240099	XLOC_128317	7	21753768	21753844	Inf	NA	5.00E-05	0.00621615		
TCONS_00240248	XLOC_128466	7	25304131	25304207	Inf	NA	5.00E-05	0.00621615		
TCONS_00240290	XLOC_128508	7	26860075	26860151	Inf	NA	5.00E-05	0.00621615	Chst11	Metabolism
TCONS_00240340	XLOC_128558	7	28342410	28343298	Inf	NA	5.00E-05	0.00621615		
TCONS_00240424	XLOC_128641	7	32144998	32145424	Inf	NA	5.00E-05	0.00621615		
TCONS_00240429	XLOC_128646	7	32796516	32796592	Inf	NA	5.00E-05	0.00621615		
TCONS_00240728	XLOC_128940	7	38785425	38785848	#NAME?	NA	5.00E-05	0.00621615	Dcn	Cytoskeleton
TCONS_00240914	XLOC_129126	7	44382910	44382985	Inf	NA	5.00E-05	0.00621615		
TCONS_00241698	XLOC_129910	7	60349342	60349734	#NAME?	NA	5.00E-05	0.00621615	Lyz2	Metabolism
TCONS_00242073	XLOC_130282	7	68585154	68585229	Inf	NA	5.00E-05	0.00621615		
TCONS_00242085	XLOC_130294	7	69477675	69477751	Inf	NA	5.00E-05	0.00621615		
TCONS_00242408	XLOC_130597	7	76633041	76633599	#NAME?	NA	5.00E-05	0.00621615		
TCONS_00242409	XLOC_130598	7	76634986	76635490	#NAME?	NA	5.00E-05	0.00621615		
TCONS_00242437	XLOC_130626	7	76910711	76910786	Inf	NA	5.00E-05	0.00621615		
TCONS_00242474	XLOC_130663	7	77130777	77130853	Inf	NA	5.00E-05	0.00621615		
TCONS_00242566	XLOC_130755	7	78998506	78998950	Inf	NA	5.00E-05	0.00621615		
TCONS_00242604	XLOC_130793	7	79509157	79509951	Inf	NA	5.00E-05	0.00621615		
TCONS_00242850	XLOC_131039	7	89082848	89086775	Inf	NA	5.00E-05	0.00621615		
TCONS_00242882	XLOC_131071	7	89244170	89244902	Inf	NA	5.00E-05	0.00621615		
TCONS_00242888	XLOC_131077	7	89254128	89254849	Inf	NA	5.00E-05	0.00621615		
TCONS_00243063	XLOC_131251	7	94071881	94072350	Inf	NA	5.00E-05	0.00621615		
TCONS_00243087	XLOC_131275	7	97131341	97132014	Inf	NA	5.00E-05	0.00621615		
TCONS_00243089	XLOC_131277	7	97132736	97133220	Inf	NA	5.00E-05	0.00621615		
TCONS_00243096	XLOC_131284	7	97140296	97141157	Inf	NA	5.00E-05	0.00621615		
TCONS_00243136	XLOC_131324	7	97201542	97202586	Inf	NA	5.00E-05	0.00621615	AABR07057990.1	
TCONS_00243622	XLOC_131787	7	109028752	109028828	Inf	NA	5.00E-05	0.00621615	Zfat	Transcription
TCONS_00243707	XLOC_131871	7	110219112	110219694	#NAME?	NA	5.00E-05	0.00621615		
TCONS_00243849	XLOC_132011	7	111075240	111075316	Inf	NA	5.00E-05	0.00621615		
TCONS_00243888	XLOC_132050	7	114312987	114313061	Inf	NA	5.00E-05	0.00621615	Trappc9	
TCONS_00243915	XLOC_132077	7	114850632	114850708	Inf	NA	5.00E-05	0.00621615	Slc45a4	Metabolism
TCONS_00244120	XLOC_132282	7	118032086	118033048	Inf	NA	5.00E-05	0.00621615	LOC681282	
TCONS_00244121	XLOC_132283	7	118033422	118034794	Inf	NA	5.00E-05	0.00621615	LOC681282	
TCONS_00244132	XLOC_132294	7	118053359	118053862	Inf	NA	5.00E-05	0.00621615		
TCONS_00244159	XLOC_132321	7	118474804	118474880	Inf	NA	5.00E-05	0.00621615	AABR07058464.1	
TCONS_00244307	XLOC_132468	7	121646015	121647285	Inf	NA	5.00E-05	0.00621615		
TCONS_00244489	XLOC_132650	7	124216823	124216899	Inf	NA	5.00E-05	0.00621615	Pacsin2	Signaling
TCONS_00244493	XLOC_132654	7	124432073	124433224	-3.64358	-3.40738	5.00E-05	0.00621615	Mcat	Metabolism
TCONS_00244523	XLOC_132684	7	125821868	125822166	Inf	NA	5.00E-05	0.00621615		
TCONS_00244687	XLOC_132835	7	130666011	130666086	Inf	NA	5.00E-05	0.00621615		
TCONS_00244707	XLOC_132855	7	131483383	131483460	Inf	NA	5.00E-05	0.00621615		
TCONS_00244790	XLOC_132933	7	134836106	134836554	Inf	NA	5.00E-05	0.00621615		
TCONS_00245078	XLOC_133210	7	138856153	138856229	Inf	NA	5.00E-05	0.00621615		
TCONS_00245343	XLOC_133472	7	144048360	144048697	Inf	NA	5.00E-05	0.00621615	Sp1;Amhr2	Transcription;Receptor
TCONS_00245360	XLOC_133488	7	144398109	144398887	Inf	NA	5.00E-05	0.00621615		
TCONS_00245361	XLOC_133489	7	144399039	144399725	Inf	NA	5.00E-05	0.00621615		
TCONS_00245367	XLOC_133495	7	144407577	144408134	Inf	NA	5.00E-05	0.00621615		
TCONS_00245414	XLOC_133542	7	144641793	144646508	-3.51014	-3.49926	5.00E-05	0.00621615	Hoxc4	Transcription
TCONS_00245999	XLOC_133759	8	22750335	22774903	-1.65302	-2.43014	5.00E-05	0.00621615	Ldlr;Spc24	Receptor
TCONS_00246258	XLOC_133805	8	33504441	33518619	Inf	NA	5.00E-05	0.00621615	Kcnj1	Transport
TCONS_00246807	XLOC_133989	8	52398359	52402953	Inf	NA	5.00E-05	0.00621615		
TCONS_00246891	XLOC_134015	8	55178288	55182545	-3.48764	-3.85471	5.00E-05	0.00621615	LOC689959;Hspb2;Cryab;RGD1564937	Unknown;Protein Binding;Development

TCONS_00247202	XLOC_134062	8	61532464	61567510	-2.8989	-3.09413	5.00E-05	0.00621615	Cspg4	Cytoskeleton
TCONS_00248543	XLOC_134319	8	97436557	97458666	-2.31511	-3.25022	5.00E-05	0.00621615	Ctsh	Protease
TCONS_00249245	XLOC_134453	8	117117429	117118522	-1.86341	-2.66593	5.00E-05	0.00621615	Gpx1;Rhoa;Usp4	Electron Transport;Signaling;Proteolysis
TCONS_00251343	XLOC_134867	8	37079697	37094706	4.05005	2.69683	5.00E-05	0.00621615	Pate-f	
TCONS_00252175	XLOC_135088	8	59164571	59240072	-2.26258	-2.71731	5.00E-05	0.00621615	Idh3a;Acsbg1	Metabolism
TCONS_00252999	XLOC_135240	8	78124653	78278607	1.9181	2.77129	5.00E-05	0.00621615	Cgnl1	Cytoskeleton
TCONS_00254854	XLOC_135726	8	2617628	2617704	Inf	NA	5.00E-05	0.00621615	Casp1	Apoptosis
TCONS_00254886	XLOC_135758	8	3687113	3687187	Inf	NA	5.00E-05	0.00621615		
TCONS_00254967	XLOC_135839	8	6625202	6626093	#NAME?	NA	5.00E-05	0.00621615		
TCONS_00254976	XLOC_135848	8	6677653	6678426	#NAME?	NA	5.00E-05	0.00621615		
TCONS_00255004	XLOC_135876	8	8567065	8567157	#NAME?	NA	5.00E-05	0.00621615		
TCONS_00255413	XLOC_136283	8	19437860	19438321	#NAME?	NA	5.00E-05	0.00621615		
TCONS_00255428	XLOC_136298	8	19469541	19470055	#NAME?	NA	5.00E-05	0.00621615		
TCONS_00255437	XLOC_136307	8	19510047	19510123	Inf	NA	5.00E-05	0.00621615		
TCONS_00255511	XLOC_136381	8	21589696	21589772	Inf	NA	5.00E-05	0.00621615		
TCONS_00255669	XLOC_136539	8	26568665	26568843	Inf	NA	5.00E-05	0.00621615		
TCONS_00255682	XLOC_136552	8	27421643	27422258	#NAME?	NA	5.00E-05	0.00621615		
TCONS_00255807	XLOC_136677	8	32133833	32134016	Inf	NA	5.00E-05	0.00621615		
TCONS_00256149	XLOC_137019	8	37181510	37181585	Inf	NA	5.00E-05	0.00621615		
TCONS_00256182	XLOC_137052	8	38060001	38060606	Inf	NA	5.00E-05	0.00621615		
TCONS_00256187	XLOC_137057	8	38067220	38067494	Inf	NA	5.00E-05	0.00621615		
TCONS_00256245	XLOC_137114	8	39674323	39674399	Inf	NA	5.00E-05	0.00621615		
TCONS_00256292	XLOC_137161	8	43889458	43889906	Inf	NA	5.00E-05	0.00621615		
TCONS_00256488	XLOC_137356	8	47187733	47187809	Inf	NA	5.00E-05	0.00621615		
TCONS_00256775	XLOC_137619	8	52464091	52465175	Inf	NA	5.00E-05	0.00621615		
TCONS_00256776	XLOC_137620	8	52487907	52488008	Inf	NA	5.00E-05	0.00621615		
TCONS_00257023	XLOC_137861	8	56786245	56786488	Inf	NA	5.00E-05	0.00621615		
TCONS_00257038	XLOC_137876	8	57422586	57423397	Inf	NA	5.00E-05	0.00621615		
TCONS_00257047	XLOC_137885	8	57430698	57431505	Inf	NA	5.00E-05	0.00621615		
TCONS_00257048	XLOC_137886	8	57432225	57433070	Inf	NA	5.00E-05	0.00621615		
TCONS_00257063	XLOC_137901	8	57451935	57453006	Inf	NA	5.00E-05	0.00621615	AABR07070198.1	
TCONS_00257064	XLOC_137902	8	57453484	57454104	Inf	NA	5.00E-05	0.00621615	AABR07070198.1	
TCONS_00257102	XLOC_137940	8	57536074	57536545	Inf	NA	5.00E-05	0.00621615		
TCONS_00257105	XLOC_137943	8	57539462	57540774	Inf	NA	5.00E-05	0.00621615		
TCONS_00257110	XLOC_137948	8	57549995	57550249	Inf	NA	5.00E-05	0.00621615		
TCONS_00257112	XLOC_137950	8	57552143	57552509	Inf	NA	5.00E-05	0.00621615		
TCONS_00257116	XLOC_137954	8	57555612	57556302	Inf	NA	5.00E-05	0.00621615		
TCONS_00257117	XLOC_137955	8	57556621	57557007	Inf	NA	5.00E-05	0.00621615		
TCONS_00257121	XLOC_137959	8	57572606	57573533	Inf	NA	5.00E-05	0.00621615		
TCONS_00257123	XLOC_137961	8	57575748	57576408	Inf	NA	5.00E-05	0.00621615		
TCONS_00257126	XLOC_137964	8	57579500	57580758	Inf	NA	5.00E-05	0.00621615		
TCONS_00257127	XLOC_137965	8	57581303	57582197	Inf	NA	5.00E-05	0.00621615		
TCONS_00257555	XLOC_138322	8	66101510	66101788	Inf	NA	5.00E-05	0.00621615		
TCONS_00257559	XLOC_138326	8	66118283	66118655	Inf	NA	5.00E-05	0.00621615		
TCONS_00257591	XLOC_138358	8	66166055	66166834	Inf	NA	5.00E-05	0.00621615		
TCONS_00257712	XLOC_138479	8	67941194	67941751	Inf	NA	5.00E-05	0.00621615		
TCONS_00257780	XLOC_138547	8	69866896	69866970	Inf	NA	5.00E-05	0.00621615		
TCONS_00257819	XLOC_138586	8	70580349	70580424	Inf	NA	5.00E-05	0.00621615	Dpp8	Protease
TCONS_00257954	XLOC_138721	8	72997789	72997919	Inf	NA	5.00E-05	0.00621615		
TCONS_00258080	XLOC_138823	8	73638926	73639002	Inf	NA	5.00E-05	0.00621615		
TCONS_00258256	XLOC_138999	8	77761366	77761655	Inf	NA	5.00E-05	0.00621615		
TCONS_00258257	XLOC_139000	8	77762117	77762561	Inf	NA	5.00E-05	0.00621615		
TCONS_00258258	XLOC_139001	8	77763564	77764049	Inf	NA	5.00E-05	0.00621615		
TCONS_00258535	XLOC_139278	8	81135087	81135272	Inf	NA	5.00E-05	0.00621615		
TCONS_00258793	XLOC_139536	8	89228234	89228879	Inf	NA	5.00E-05	0.00621615	AABR07070873.1	
TCONS_00258997	XLOC_139740	8	95673944	95674020	Inf	NA	5.00E-05	0.00621615		
TCONS_00259262	XLOC_139998	8	102294770	102295530	#NAME?	NA	5.00E-05	0.00621615	Slc9a9	Transport
TCONS_00259266	XLOC_140002	8	102299645	102300031	#NAME?	NA	5.00E-05	0.00621615	Slc9a9	Transport
TCONS_00259371	XLOC_140090	8	105028161	105029240	#NAME?	NA	5.00E-05	0.00621615		
TCONS_00259456	XLOC_140175	8	106491482	106491558	Inf	NA	5.00E-05	0.00621615		
TCONS_00259658	XLOC_140377	8	109751746	109752309	Inf	NA	5.00E-05	0.00621615	5S_rRNA	
TCONS_00259664	XLOC_140383	8	109826699	109826967	Inf	NA	5.00E-05	0.00621615	SNORA17	

TCONS_00259795	XLOC_140514	8	112988919	112988994	Inf	NA	5.00E-05	0.00621615	AABR07071385.1	
TCONS_00259870	XLOC_140589	8	114072392	114072547	Inf	NA	5.00E-05	0.00621615		
TCONS_00260478	XLOC_141178	8	131693428	131693894	Inf	NA	5.00E-05	0.00621615		
TCONS_00260656	XLOC_141298	9	4621424	4624425	5.31253	6.18245	5.00E-05	0.00621615	AABR07066188.1	
TCONS_00261984	XLOC_141635	9	50526810	50548046	-2.38722	-2.65342	5.00E-05	0.00621615	RGD1305645	Cytoskeleton
TCONS_00262821	XLOC_141798	9	80118028	80144789	-2.52579	-3.27136	5.00E-05	0.00621615	Igfbp5;Igfbp2	Receptor
TCONS_00263004	XLOC_141837	9	82556504	82564900	-4.36177	-4.84576	5.00E-05	0.00621615	Des;Speg	Cytoskeleton
TCONS_00263680	XLOC_141941	9	98504849	98513170	-2.86631	-2.93457	5.00E-05	0.00621615	Ilkap;Erfe;Klhl30	Signaling
TCONS_00264118	XLOC_142048	9	117044532	117050371	-2.90875	-3.05838	5.00E-05	0.00621615	Tmem200c	
TCONS_00264275	XLOC_142095	9	4001013	4004297	4.14451	3.92088	5.00E-05	0.00621615		
TCONS_00264277	XLOC_142096	9	4072822	4280794	4.89126	4.29571	5.00E-05	0.00621615	LOC100910057;Sult1c2;AABR07066160.1;Sult1c2a	Metabolism
TCONS_00264292	XLOC_142103	9	4866704	4879755	5.04854	4.73768	5.00E-05	0.00621615	RGD1559960	
TCONS_00265253	XLOC_142338	9	41511717	41529110	3.75912	3.69789	5.00E-05	0.00621615		
TCONS_00265628	XLOC_142412	9	52626850	52638016	-3.44692	-3.55416	5.00E-05	0.00621615		
TCONS_00266425	XLOC_142572	9	82208219	82214440	-4.19626	-3.77876	5.00E-05	0.00621615	Ihh	Development
TCONS_00267493	XLOC_142861	9	2076809	2076885	Inf	NA	5.00E-05	0.00621615		
TCONS_00267602	XLOC_142970	9	2857502	2857577	Inf	NA	5.00E-05	0.00621615		
TCONS_00267614	XLOC_142982	9	4012263	4012583	3.65544	2.99324	5.00E-05	0.00621615		
TCONS_00267626	XLOC_142994	9	4061439	4066707	3.29678	3.84831	5.00E-05	0.00621615	LOC100910057	Metabolism
TCONS_00267664	XLOC_143032	9	4805735	4805853	Inf	NA	5.00E-05	0.00621615	LOC100910526	Metabolism
TCONS_00267798	XLOC_143163	9	8527564	8528105	Inf	NA	5.00E-05	0.00621615		
TCONS_00267831	XLOC_143196	9	8979675	8979917	#NAME?	NA	5.00E-05	0.00621615		
TCONS_00267926	XLOC_143290	9	10906180	10906444	Inf	NA	5.00E-05	0.00621615	Tnfaip8l1;AABR07066532.1	Apoptosis
TCONS_00268078	XLOC_143442	9	13421346	13421859	#NAME?	NA	5.00E-05	0.00621615		
TCONS_00268086	XLOC_143450	9	13537750	13537826	Inf	NA	5.00E-05	0.00621615	AABR07066735.1	
TCONS_00268304	XLOC_143660	9	17035052	17035127	Inf	NA	5.00E-05	0.00621615	Abcc10	Transport
TCONS_00268518	XLOC_143851	9	20042851	20043167	Inf	NA	5.00E-05	0.00621615	Mep1a	Protease
TCONS_00268745	XLOC_144070	9	25388120	25388760	Inf	NA	5.00E-05	0.00621615		
TCONS_00268784	XLOC_144109	9	25753406	25754361	Inf	NA	5.00E-05	0.00621615		
TCONS_00268785	XLOC_144110	9	25754476	25756163	Inf	NA	5.00E-05	0.00621615		
TCONS_00269497	XLOC_144788	9	41462534	41468284	4.10324	3.82425	5.00E-05	0.00621615		
TCONS_00269499	XLOC_144790	9	41471696	41475173	3.50195	3.34733	5.00E-05	0.00621615		
TCONS_00269500	XLOC_144791	9	41475715	41479152	3.97201	3.81193	5.00E-05	0.00621615		
TCONS_00269501	XLOC_144792	9	41479864	41494105	3.49569	3.74022	5.00E-05	0.00621615		
TCONS_00269503	XLOC_144794	9	41496424	41502943	3.60864	3.17184	5.00E-05	0.00621615		
TCONS_00269504	XLOC_144795	9	41503829	41509969	3.07634	2.87453	5.00E-05	0.00621615		
TCONS_00269507	XLOC_144798	9	41544063	41544472	Inf	NA	5.00E-05	0.00621615		
TCONS_00269689	XLOC_144966	9	45874236	45874340	Inf	NA	5.00E-05	0.00621615		
TCONS_00269770	XLOC_145047	9	49234006	49234082	Inf	NA	5.00E-05	0.00621615		
TCONS_00269783	XLOC_145060	9	49281848	49282311	Inf	NA	5.00E-05	0.00621615		
TCONS_00269819	XLOC_145096	9	49421253	49421329	Inf	NA	5.00E-05	0.00621615	AABR07067499.1	
TCONS_00269921	XLOC_145198	9	52295108	52295517	#NAME?	NA	5.00E-05	0.00621615		
TCONS_00269930	XLOC_145207	9	52305607	52305679	Inf	NA	5.00E-05	0.00621615		
TCONS_00269938	XLOC_145215	9	52320790	52321395	#NAME?	NA	5.00E-05	0.00621615		
TCONS_00269981	XLOC_145258	9	52427254	52433923	-3.20559	-3.19799	5.00E-05	0.00621615		
TCONS_00269986	XLOC_145263	9	52443525	52451193	-2.20849	-2.88232	5.00E-05	0.00621615		
TCONS_00269989	XLOC_145266	9	52467805	52477333	-3.87469	-4.45218	5.00E-05	0.00621615		
TCONS_00269990	XLOC_145267	9	52477405	52480665	-3.0156	-2.921	5.00E-05	0.00621615		
TCONS_00269991	XLOC_145268	9	52480742	52486999	-3.09631	-3.77447	5.00E-05	0.00621615		
TCONS_00269994	XLOC_145271	9	52499685	52502051	-2.69842	-2.79348	5.00E-05	0.00621615		
TCONS_00270000	XLOC_145277	9	52641964	52647861	-4.53072	-4.57532	5.00E-05	0.00621615		
TCONS_00270191	XLOC_145468	9	57886436	57886890	Inf	NA	5.00E-05	0.00621615		
TCONS_00270215	XLOC_145492	9	58044262	58044338	Inf	NA	5.00E-05	0.00621615		
TCONS_00270252	XLOC_145529	9	59779400	59783533	-3.20064	-3.96081	5.00E-05	0.00621615		
TCONS_00270463	XLOC_145737	9	67730575	67730648	Inf	NA	5.00E-05	0.00621615		
TCONS_00270613	XLOC_145887	9	70864839	70864915	Inf	NA	5.00E-05	0.00621615		
TCONS_00270938	XLOC_146212	9	76115300	76115738	#NAME?	NA	5.00E-05	0.00621615		
TCONS_00271021	XLOC_146293	9	79548955	79549192	Inf	NA	5.00E-05	0.00621615	Mreg	Metabolism

TCONS_00271131	XLOC_146403	9	82216211	82216287	Inf	NA	5.00E-05	0.00621615	Ihh	Development
TCONS_00271356	XLOC_146628	9	86180854	86180930	Inf	NA	5.00E-05	0.00621615		
TCONS_00271360	XLOC_146632	9	86191644	86191797	Inf	NA	5.00E-05	0.00621615		
TCONS_00271751	XLOC_147020	9	95525139	95526770	Inf	NA	5.00E-05	0.00621615		
TCONS_00271775	XLOC_147044	9	95661497	95661572	Inf	NA	5.00E-05	0.00621615		
TCONS_00271823	XLOC_147092	9	96439645	96439721	Inf	NA	5.00E-05	0.00621615		
TCONS_00272187	XLOC_147456	9	105180844	105180919	Inf	NA	5.00E-05	0.00621615		
TCONS_00272329	XLOC_147598	9	106448606	106450554	-2.86573	-3.18987	5.00E-05	0.00621615		
TCONS_00272347	XLOC_147616	9	107630433	107631450	Inf	NA	5.00E-05	0.00621615		
TCONS_00272593	XLOC_147840	9	112488697	112488773	Inf	NA	5.00E-05	0.00621615		
TCONS_00272723	XLOC_147967	9	114463228	114463720	#NAME?	NA	5.00E-05	0.00621615		
TCONS_00272728	XLOC_147972	9	114473251	114473537	#NAME?	NA	5.00E-05	0.00621615		
TCONS_00272755	XLOC_147999	9	116559242	116559813	#NAME?	NA	5.00E-05	0.00621615		
TCONS_00272868	XLOC_148111	9	121225680	121225756	Inf	NA	5.00E-05	0.00621615		
TCONS_00031661	XLOC_016558	10	764171	904593	-3.72174	-3.43599	5.00E-05	0.00621615	Myh11;Nde1;rno-mir-484;Marf1	Cytoskeleton;Development;Transcription
TCONS_00032742	XLOC_016810	10	29067101	29084035	-2.88269	-2.63607	5.00E-05	0.00621615	C1qtnf2;AABR07029451.1	Immune
TCONS_00033266	XLOC_016952	10	40438355	40450930	-1.69556	-2.41993	5.00E-05	0.00621615	Gm2a	
TCONS_00036016	XLOC_017522	10	86303560	86313069	-3.53521	-4.36817	5.00E-05	0.00621615	Ppp1r1b;Stard3	Signaling;Metabolism
TCONS_00036478	XLOC_017613	10	90376932	90383231	-1.96026	-2.67819	5.00E-05	0.00621615	Grn;Fam171a2	Signaling;Transcription
TCONS_00036482	XLOC_017614	10	90550146	90552057	-2.99309	-3.16363	5.00E-05	0.00621615	Fzd2	Receptor
TCONS_00036743	XLOC_017660	10	94169146	94206536	2.0104	2.72962	5.00E-05	0.00621615	Ace;Ace3;Kcnh6	Metabolism;Transport
TCONS_00037445	XLOC_017800	10	108377299	108413059	-1.69594	-2.72133	5.00E-05	0.00621615	Ccdc40;AABR07030901.1;Gaa;Eif4a3	Metabolism;Transcription
TCONS_00038644	XLOC_018030	10	15119662	15125408	3.5403	3.32924	5.00E-05	0.00621615	Mslnl;Msln	Extracellular Matrix
TCONS_00038914	XLOC_018085	10	19160581	19164505	-1.80761	-2.44655	5.00E-05	0.00621615	Foxi1	Transcription
TCONS_00039040	XLOC_018139	10	31241009	31258419	-2.80527	-2.91819	5.00E-05	0.00621615	Adam19;Nipal4;AABR07029505.1	Development
TCONS_00039419	XLOC_018236	10	40742399	40764185	-1.99916	-3.10982	5.00E-05	0.00621615	Sparc	Cytoskeleton
TCONS_00039505	XLOC_018264	10	43911946	43912900	Inf	NA	5.00E-05	0.00621615	Olr1414	
TCONS_00040566	XLOC_018442	10	58430828	58461992	4.85149	4.28272	5.00E-05	0.00621615	SNORA17	
TCONS_00043371	XLOC_019043	10	104521699	104522241	-1.78893	-2.64003	5.00E-05	0.00621615	Sap30bp;Rps18l1;Itgb4	Transcription;Signaling
TCONS_00043676	XLOC_019099	10	107415222	107424710	-3.44312	-4.77603	5.00E-05	0.00621615	Lgals3bp;Cant1	Cytoskeleton;Metabolism
TCONS_00043945	XLOC_019145	10	110229921	110232843	2.02523	2.66951	5.00E-05	0.00621615	Cd7	Receptor
TCONS_00044420	XLOC_019501	10	6564113	6564188	Inf	NA	5.00E-05	0.00621615		
TCONS_00044452	XLOC_019533	10	7271239	7271341	Inf	NA	5.00E-05	0.00621615	Tmem114	
TCONS_00044546	XLOC_019627	10	10077679	10078010	Inf	NA	5.00E-05	0.00621615		
TCONS_00044662	XLOC_019741	10	11981218	11981294	Inf	NA	5.00E-05	0.00621615		
TCONS_00044691	XLOC_019770	10	12046254	12046447	Inf	NA	5.00E-05	0.00621615	Mefv	Cytoskeleton
TCONS_00044866	XLOC_019940	10	16064111	16064436	Inf	NA	5.00E-05	0.00621615		
TCONS_00044926	XLOC_020000	10	16348552	16349272	#NAME?	NA	5.00E-05	0.00621615		
TCONS_00044950	XLOC_020024	10	16367520	16368074	#NAME?	NA	5.00E-05	0.00621615		
TCONS_00045396	XLOC_020401	10	23462372	23462447	Inf	NA	5.00E-05	0.00621615		
TCONS_00045591	XLOC_020595	10	29828545	29829026	#NAME?	NA	5.00E-05	0.00621615		
TCONS_00045631	XLOC_020635	10	30731360	30731436	Inf	NA	5.00E-05	0.00621615		
TCONS_00045822	XLOC_020817	10	32792064	32792140	Inf	NA	5.00E-05	0.00621615		
TCONS_00046247	XLOC_021241	10	40047666	40047742	Inf	NA	5.00E-05	0.00621615	Rapgef6;Cdc42se2	Signaling;Development
TCONS_00046277	XLOC_021271	10	42165238	42165313	Inf	NA	5.00E-05	0.00621615	AABR07029636.1	
TCONS_00046345	XLOC_021338	10	43916262	43917834	Inf	NA	5.00E-05	0.00621615	Olr1414	
TCONS_00046348	XLOC_021341	10	43919326	43920016	Inf	NA	5.00E-05	0.00621615	Olr1414	
TCONS_00046350	XLOC_021343	10	43920607	43921616	Inf	NA	5.00E-05	0.00621615	Olr1414;Olr1415	
TCONS_00046351	XLOC_021344	10	43922352	43923168	Inf	NA	5.00E-05	0.00621615	Olr1414;Olr1415	
TCONS_00046366	XLOC_021359	10	44825073	44825883	3.32271	2.88266	5.00E-05	0.00621615		
TCONS_00046397	XLOC_021390	10	45857215	45857291	Inf	NA	5.00E-05	0.00621615	Zfp496	Transcription

TCONS_00046405	XLOC_021398	10	46173283	46173358	Inf	NA	5.00E-05	0.00621615	Flcn;Cops3;AC097038.1	Development
TCONS_00046423	XLOC_021416	10	46297101	46297176	Inf	NA	5.00E-05	0.00621615		
TCONS_00046562	XLOC_021555	10	49897624	49897812	Inf	NA	5.00E-05	0.00621615		
TCONS_00046585	XLOC_021577	10	50602426	50602689	Inf	NA	5.00E-05	0.00621615		
TCONS_00046636	XLOC_021628	10	52744448	52744812	#NAME?	NA	5.00E-05	0.00621615	Shisa6	Development
TCONS_00046825	XLOC_021817	10	56972784	56972976	Inf	NA	5.00E-05	0.00621615		
TCONS_00047199	XLOC_022188	10	66498803	66499250	Inf	NA	5.00E-05	0.00621615	RGD1565317	
TCONS_00047329	XLOC_022317	10	69957613	69957737	Inf	NA	5.00E-05	0.00621615		
TCONS_00047344	XLOC_022332	10	70003462	70003538	Inf	NA	5.00E-05	0.00621615		
TCONS_00047358	XLOC_022346	10	70029711	70030057	Inf	NA	5.00E-05	0.00621615		
TCONS_00047436	XLOC_022421	10	72153731	72153807	Inf	NA	5.00E-05	0.00621615	Dhrs11;LOC102553386;Ggnbp2	Metabolism
TCONS_00047719	XLOC_022671	10	77423532	77423608	Inf	NA	5.00E-05	0.00621615		
TCONS_00047771	XLOC_022723	10	78324305	78325047	#NAME?	NA	5.00E-05	0.00621615	SNORA17	
TCONS_00047859	XLOC_022811	10	82330139	82330215	Inf	NA	5.00E-05	0.00621615	Acsf2;AABR07030351.1	Metabolism
TCONS_00047985	XLOC_022933	10	84280525	84280601	Inf	NA	5.00E-05	0.00621615		
TCONS_00048293	XLOC_023238	10	90322328	90322924	Inf	NA	5.00E-05	0.00621615	Slc4a1	Transport
TCONS_00048716	XLOC_023633	10	97272252	97272328	Inf	NA	5.00E-05	0.00621615		
TCONS_00048846	XLOC_023763	10	101331393	101331471	Inf	NA	5.00E-05	0.00621615		
TCONS_00048878	XLOC_023795	10	101615533	101616090	#NAME?	NA	5.00E-05	0.00621615		
TCONS_00048894	XLOC_023811	10	101638425	101639285	#NAME?	NA	5.00E-05	0.00621615		
TCONS_00048903	XLOC_023820	10	101655572	101656183	#NAME?	NA	5.00E-05	0.00621615		
TCONS_00048905	XLOC_023822	10	101657753	101658307	#NAME?	NA	5.00E-05	0.00621615		
TCONS_00048908	XLOC_023825	10	101664362	101665318	#NAME?	NA	5.00E-05	0.00621615		
TCONS_00048917	XLOC_023834	10	101681872	101681947	Inf	NA	5.00E-05	0.00621615		
TCONS_00048919	XLOC_023836	10	101684901	101685760	#NAME?	NA	5.00E-05	0.00621615		
TCONS_00048923	XLOC_023840	10	101697727	101698518	#NAME?	NA	5.00E-05	0.00621615	Mir297	
TCONS_00048936	XLOC_023853	10	102121225	102121301	Inf	NA	5.00E-05	0.00621615		
TCONS_00049045	XLOC_023962	10	103854933	103855321	Inf	NA	5.00E-05	0.00621615	Fads6	
TCONS_00049046	XLOC_023963	10	103861908	103862395	Inf	NA	5.00E-05	0.00621615	Ush1g	
TCONS_00049092	XLOC_024009	10	105491497	105498268	-2.36718	-2.56435	5.00E-05	0.00621615	Sphk1;Ube2o	Signaling;Proteolysis
TCONS_00049125	XLOC_024042	10	105938238	105945751	2.51563	2.74881	5.00E-05	0.00621615	Mgat5b	Metabolism
TCONS_00049191	XLOC_024096	10	106467642	106468210	Inf	NA	5.00E-05	0.00621615		
TCONS_00049244	XLOC_024149	10	107545359	107546505	#NAME?	NA	5.00E-05	0.00621615	Rbfox3	
TCONS_00049253	XLOC_024158	10	107561727	107562380	#NAME?	NA	5.00E-05	0.00621615		
TCONS_00049290	XLOC_024195	10	107610883	107611100	#NAME?	NA	5.00E-05	0.00621615		
TCONS_00049297	XLOC_024202	10	107618511	107621083	#NAME?	NA	5.00E-05	0.00621615		
TCONS_00049298	XLOC_024203	10	107621548	107622455	#NAME?	NA	5.00E-05	0.00621615		
TCONS_00049300	XLOC_024205	10	107623576	107624153	#NAME?	NA	5.00E-05	0.00621615		
TCONS_00049424	XLOC_024329	10	107801223	107802229	#NAME?	NA	5.00E-05	0.00621615		
TCONS_00049447	XLOC_024352	10	107841023	107841702	#NAME?	NA	5.00E-05	0.00621615		
TCONS_00049452	XLOC_024357	10	107845428	107846670	#NAME?	NA	5.00E-05	0.00621615		
TCONS_00049547	XLOC_024452	10	108730927	108731600	Inf	NA	5.00E-05	0.00621615	AABR07030911.1;AABR07030911.2	
TCONS_00049551	XLOC_024456	10	108738256	108738943	Inf	NA	5.00E-05	0.00621615	AABR07030911.2	
TCONS_00049624	XLOC_024529	10	111026922	111026998	Inf	NA	5.00E-05	0.00621615	SNORA62;Ptchd3	Receptor
TCONS_00050083	XLOC_024713	11	33812661	33868530	-5.17918	-4.80048	5.00E-05	0.00621615	LOC102556347;Cbr1;Trub2-ps1;Rn50_11_0375.5;Trub2-ps2;Rn50_11_0375.7	Metabolism
TCONS_00050088	XLOC_024719	11	33887141	33893433	-2.26075	-2.69754	5.00E-05	0.00621615	Rn50_11_0375.8	
TCONS_00050251	XLOC_024740	11	35608193	35619495	3.20657	3.81555	5.00E-05	0.00621615	AABR07033697.2;AABR07033697.1	
TCONS_00050300	XLOC_024752	11	36851037	36912229	-4.1142	-3.93487	5.00E-05	0.00621615	Pcp4	
TCONS_00050983	XLOC_024961	11	68582761	68601469	2.65045	3.25546	5.00E-05	0.00621615	Pdia5;AABR07034404.1;U1	Metabolism
TCONS_00051280	XLOC_025029	11	78028762	78178468	2.70269	2.72956	5.00E-05	0.00621615	P3h2	

TCONS_00051376	XLOC_025066	11	83782458	83785497	Inf	NA	5.00E-05	0.00621615		
TCONS_00051977	XLOC_025232	11	24425004	24641858	-2.33845	-2.65973	5.00E-05	0.00621615	App	Signaling
TCONS_00052298	XLOC_025307	11	34132580	34142753	-5.08964	-4.41615	5.00E-05	0.00621615	Cldn14	Extracellular Matrix
TCONS_00053004	XLOC_025459	11	65782566	65845418	-2.36309	-2.79532	5.00E-05	0.00621615	Fstl1	Signaling
TCONS_00053445	XLOC_025534	11	73676600	73678984	2.20622	2.66574	5.00E-05	0.00621615	Fam43a	Unknown
TCONS_00053811	XLOC_025624	11	84833727	84862371	5.04998	6.21301	5.00E-05	0.00621615	Mir6323;A930003A15Rik	
TCONS_00054184	XLOC_025740	11	2001151	2006855	3.43491	3.44713	5.00E-05	0.00621615		
TCONS_00054290	XLOC_025846	11	3193714	3193798	Inf	NA	5.00E-05	0.00621615		
TCONS_00054314	XLOC_025870	11	3289405	3289481	Inf	NA	5.00E-05	0.00621615		
TCONS_00054737	XLOC_026292	11	15588819	15589429	#NAME?	NA	5.00E-05	0.00621615		
TCONS_00055232	XLOC_026737	11	28445448	28445575	Inf	NA	5.00E-05	0.00621615		
TCONS_00055306	XLOC_026811	11	29740083	29740212	Inf	NA	5.00E-05	0.00621615		
TCONS_00055388	XLOC_026892	11	30688637	30689439	#NAME?	NA	5.00E-05	0.00621615		
TCONS_00055563	XLOC_027046	11	32727806	32728402	#NAME?	NA	5.00E-05	0.00621615		
TCONS_00055662	XLOC_027144	11	35472429	35473033	#NAME?	NA	5.00E-05	0.00621615		
TCONS_00055840	XLOC_027319	11	40426104	40426180	Inf	NA	5.00E-05	0.00621615		
TCONS_00056093	XLOC_027546	11	45641476	45641552	Inf	NA	5.00E-05	0.00621615	AABR07033943.1	
TCONS_00056286	XLOC_027721	11	56497978	56498054	Inf	NA	5.00E-05	0.00621615		
TCONS_00056365	XLOC_027800	11	57804991	57805355	#NAME?	NA	5.00E-05	0.00621615		
TCONS_00056381	XLOC_027816	11	57839544	57853481	-3.56912	-3.5957	5.00E-05	0.00621615		
TCONS_00056382	XLOC_027817	11	57853649	57856867	-3.43495	-2.70488	5.00E-05	0.00621615		
TCONS_00056384	XLOC_027819	11	57860679	57868748	-4.57208	-4.46085	5.00E-05	0.00621615		
TCONS_00056445	XLOC_027880	11	60327050	60327633	#NAME?	NA	5.00E-05	0.00621615	LOC685767	
TCONS_00056485	XLOC_027920	11	60772145	60772221	Inf	NA	5.00E-05	0.00621615		
TCONS_00056488	XLOC_027923	11	60821638	60821983	Inf	NA	5.00E-05	0.00621615	Cd200r1l	Receptor
TCONS_00056668	XLOC_028103	11	68074907	68078321	4.25895	4.89813	5.00E-05	0.00621615	AABR07034393.1	
TCONS_00056886	XLOC_028319	11	72693729	72693840	#NAME?	NA	5.00E-05	0.00621615		
TCONS_00056919	XLOC_028352	11	73389119	73391228	Inf	NA	5.00E-05	0.00621615		
TCONS_00056932	XLOC_028365	11	73611392	73613189	Inf	NA	5.00E-05	0.00621615		
TCONS_00056936	XLOC_028369	11	73614484	73617193	Inf	NA	5.00E-05	0.00621615		
TCONS_00056937	XLOC_028370	11	73617452	73620217	Inf	NA	5.00E-05	0.00621615		
TCONS_00056942	XLOC_028375	11	73626190	73626632	Inf	NA	5.00E-05	0.00621615		
TCONS_00056959	XLOC_028390	11	73895980	73896310	Inf	NA	5.00E-05	0.00621615		
TCONS_00057001	XLOC_028429	11	74415563	74416045	#NAME?	NA	5.00E-05	0.00621615		
TCONS_00057037	XLOC_028465	11	74459625	74460152	#NAME?	NA	5.00E-05	0.00621615		
TCONS_00057042	XLOC_028470	11	74463626	74464314	#NAME?	NA	5.00E-05	0.00621615		
TCONS_00057198	XLOC_028626	11	79125954	79126257	Inf	NA	5.00E-05	0.00621615	AABR07034573.1	
TCONS_00057257	XLOC_028664	11	80424054	80424146	Inf	NA	5.00E-05	0.00621615		
TCONS_00057464	XLOC_028869	11	85492009	85492086	Inf	NA	5.00E-05	0.00621615		
TCONS_00057486	XLOC_028891	11	86083914	86084240	Inf	NA	5.00E-05	0.00621615	LOC100361706	
TCONS_00057719	XLOC_029117	11	90453670	90453815	Inf	NA	5.00E-05	0.00621615		
TCONS_00059444	XLOC_029479	12	40466494	40498950	-1.82323	-2.75289	5.00E-05	0.00621615	Acad10;Aldh2	Metabolism
TCONS_00062260	XLOC_030076	12	46593999	46601339	Inf	NA	5.00E-05	0.00621615		
TCONS_00062310	XLOC_030083	12	46863203	46863344	3.7459	3.57386	5.00E-05	0.00621615	AC097575.2;U4;Sirt4	Transcription
TCONS_00062551	XLOC_030141	12	52093602	52190278	-4.35288	-4.6555	5.00E-05	0.00621615	Galnt9	Metabolism
TCONS_00062706	XLOC_030232	12	935888	935964	Inf	NA	5.00E-05	0.00621615	Kl	Development
TCONS_00062724	XLOC_030250	12	1230381	1230753	#NAME?	NA	5.00E-05	0.00621615		
TCONS_00062870	XLOC_030396	12	5258717	5258860	Inf	NA	5.00E-05	0.00621615		
TCONS_00063415	XLOC_030938	12	16135712	16135787	Inf	NA	5.00E-05	0.00621615	Lfng	Signaling
TCONS_00063457	XLOC_030980	12	16872716	16873115	Inf	NA	5.00E-05	0.00621615		
TCONS_00063703	XLOC_031225	12	24071025	24071198	Inf	NA	5.00E-05	0.00621615		
TCONS_00063947	XLOC_031364	12	30530093	30530167	Inf	NA	5.00E-05	0.00621615	Psph;Gbas	Signaling;Transport
TCONS_00064058	XLOC_031473	12	32789806	32789882	Inf	NA	5.00E-05	0.00621615		
TCONS_00064116	XLOC_031531	12	37094990	37095134	Inf	NA	5.00E-05	0.00621615		
TCONS_00064356	XLOC_031761	12	42832575	42832649	Inf	NA	5.00E-05	0.00621615		
TCONS_00064371	XLOC_031776	12	42885866	42885942	Inf	NA	5.00E-05	0.00621615		
TCONS_00064377	XLOC_031782	12	42911525	42911997	#NAME?	NA	5.00E-05	0.00621615	AABR07036426.1	
TCONS_00064384	XLOC_031789	12	42923535	42924424	#NAME?	NA	5.00E-05	0.00621615		
TCONS_00064390	XLOC_031795	12	43168917	43169133	Inf	NA	5.00E-05	0.00621615		
TCONS_00064438	XLOC_031843	12	44527414	44528740	Inf	NA	5.00E-05	0.00621615	Nos1;Ksr2	Metabolism;Signaling

TCONS_00064491	XLOC_031896	12	46570791	46571549	Inf	NA	5.00E-05	0.00621615		
TCONS_00064493	XLOC_031898	12	46572175	46574082	Inf	NA	5.00E-05	0.00621615		
TCONS_00064497	XLOC_031902	12	46613430	46615466	4.0824	3.64869	5.00E-05	0.00621615	Bicdl1	
TCONS_00064763	XLOC_032165	12	52085065	52085788	#NAME?	NA	5.00E-05	0.00621615	Noc4l	Transcription
TCONS_00065157	XLOC_032296	13	48607307	48627999	3.09721	3.95048	5.00E-05	0.00621615	Slc41a1	Transport
TCONS_00065448	XLOC_032340	13	52147554	52190228	-3.09495	-3.33073	5.00E-05	0.00621615	Lmod1;Shisa4	Cytoskeleton
TCONS_00066338	XLOC_032561	13	87966752	88006314	-3.07973	-3.55487	5.00E-05	0.00621615	Rgs5	Signaling
TCONS_00068278	XLOC_033032	13	70656704	70783533	-1.90417	-2.51796	5.00E-05	0.00621615	Lamc1	Extracellular Matrix
TCONS_00069349	XLOC_033269	13	96769150	97173501	-2.63574	-2.67303	5.00E-05	0.00621615	Kif26b	Cytoskeleton
TCONS_00070060	XLOC_033444	13	2816543	2816708	Inf	NA	5.00E-05	0.00621615		
TCONS_00070072	XLOC_033456	13	3078272	3078454	Inf	NA	5.00E-05	0.00621615	SNORA17	
TCONS_00070079	XLOC_033463	13	3103776	3104618	Inf	NA	5.00E-05	0.00621615		
TCONS_00070109	XLOC_033493	13	3203168	3203482	Inf	NA	5.00E-05	0.00621615		
TCONS_00070111	XLOC_033495	13	3204136	3204508	Inf	NA	5.00E-05	0.00621615		
TCONS_00070112	XLOC_033496	13	3206389	3206465	Inf	NA	5.00E-05	0.00621615		
TCONS_00070311	XLOC_033695	13	25348645	25349019	Inf	NA	5.00E-05	0.00621615		
TCONS_00070362	XLOC_033746	13	26814883	26814958	Inf	NA	5.00E-05	0.00621615	Kdsr	Metabolism
TCONS_00070394	XLOC_033778	13	27702269	27702601	#NAME?	NA	5.00E-05	0.00621615		
TCONS_00070395	XLOC_033779	13	27702771	27703425	#NAME?	NA	5.00E-05	0.00621615		
TCONS_00070442	XLOC_033826	13	30064936	30065011	Inf	NA	5.00E-05	0.00621615		
TCONS_00070593	XLOC_033974	13	37368205	37368282	Inf	NA	5.00E-05	0.00621615		
TCONS_00070688	XLOC_034069	13	40642904	40642980	Inf	NA	5.00E-05	0.00621615		
TCONS_00070817	XLOC_034174	13	46033339	46033415	Inf	NA	5.00E-05	0.00621615		
TCONS_00071110	XLOC_034437	13	52619136	52619212	Inf	NA	5.00E-05	0.00621615	Tnni1	Cytoskeleton
TCONS_00071358	XLOC_034685	13	59954028	59954204	Inf	NA	5.00E-05	0.00621615		
TCONS_00071397	XLOC_034724	13	60034275	60034883	#NAME?	NA	5.00E-05	0.00621615		
TCONS_00071481	XLOC_034808	13	60390842	60390917	Inf	NA	5.00E-05	0.00621615		
TCONS_00071683	XLOC_035010	13	67343008	67343298	#NAME?	NA	5.00E-05	0.00621615	Ptgs2	Metabolism
TCONS_00071856	XLOC_035181	13	70650035	70650111	Inf	NA	5.00E-05	0.00621615	Lamc1	Extracellular Matrix
TCONS_00072113	XLOC_035437	13	79124551	79124698	Inf	NA	5.00E-05	0.00621615		
TCONS_00072129	XLOC_035453	13	79324250	79324804	#NAME?	NA	5.00E-05	0.00621615	Rn50_13_0843.1	
TCONS_00072255	XLOC_035578	13	81481802	81481878	Inf	NA	5.00E-05	0.00621615	AABR07021612.1	
TCONS_00072308	XLOC_035631	13	82770455	82771010	Inf	NA	5.00E-05	0.00621615		
TCONS_00072314	XLOC_035637	13	82853378	82853454	Inf	NA	5.00E-05	0.00621615		
TCONS_00072320	XLOC_035643	13	82896273	82897075	Inf	NA	5.00E-05	0.00621615		
TCONS_00072434	XLOC_035757	13	85088438	85088513	Inf	NA	5.00E-05	0.00621615		
TCONS_00072568	XLOC_035869	13	89438017	89438187	Inf	NA	5.00E-05	0.00621615	Fcgr2b	Immune
TCONS_00072624	XLOC_035925	13	91825705	91826172	Inf	NA	5.00E-05	0.00621615		
TCONS_00072729	XLOC_036030	13	95476136	95477461	Inf	NA	5.00E-05	0.00621615		
TCONS_00072730	XLOC_036031	13	95477628	95478712	Inf	NA	5.00E-05	0.00621615		
TCONS_00072731	XLOC_036032	13	95479121	95480863	Inf	NA	5.00E-05	0.00621615		
TCONS_00072994	XLOC_036281	13	100541333	100541840	#NAME?	NA	5.00E-05	0.00621615	Srp9	Signaling
TCONS_00073249	XLOC_036531	13	106333507	106333807	#NAME?	NA	5.00E-05	0.00621615		
TCONS_00073356	XLOC_036637	13	108992386	108992970	Inf	NA	5.00E-05	0.00621615		
TCONS_00073358	XLOC_036639	13	108998509	108999005	Inf	NA	5.00E-05	0.00621615		
TCONS_00073915	XLOC_037011	14	6994189	7025308	-3.80452	-4.63649	5.00E-05	0.00621615	Sparcl1;LOC100359907	Signaling
TCONS_00073978	XLOC_037027	14	8634978	8668509	3.0996	3.59214	5.00E-05	0.00621615		
TCONS_00074675	XLOC_037174	14	34826856	34847275	4.97537	4.16301	5.00E-05	0.00621615		
TCONS_00077105	XLOC_037691	14	8623782	8634901	3.68375	3.67816	5.00E-05	0.00621615		
TCONS_00077305	XLOC_037765	14	17969218	18076907	2.18654	2.69881	5.00E-05	0.00621615	Parm1	Apoptosis
TCONS_00078120	XLOC_037997	14	61011944	61039917	Inf	NA	5.00E-05	0.00621615		
TCONS_00079623	XLOC_038419	14	3352547	3352622	Inf	NA	5.00E-05	0.00621615	Btbd8;Lpcat2b;Ephx4	Metabolism
TCONS_00079660	XLOC_038456	14	4422688	4423704	Inf	NA	5.00E-05	0.00621615		
TCONS_00079661	XLOC_038457	14	4423851	4424791	Inf	NA	5.00E-05	0.00621615		
TCONS_00079662	XLOC_038458	14	4424853	4425369	Inf	NA	5.00E-05	0.00621615		
TCONS_00079828	XLOC_038618	14	7140487	7140601	Inf	NA	5.00E-05	0.00621615		
TCONS_00080141	XLOC_038904	14	13638766	13638841	Inf	NA	5.00E-05	0.00621615		
TCONS_00080168	XLOC_038931	14	13776706	13776896	Inf	NA	5.00E-05	0.00621615		
TCONS_00080284	XLOC_039041	14	14928678	14928754	Inf	NA	5.00E-05	0.00621615		
TCONS_00080335	XLOC_039092	14	16336713	16336869	Inf	NA	5.00E-05	0.00621615	Ccni	Cell Cycle
TCONS_00080411	XLOC_039168	14	18128240	18129270	Inf	NA	5.00E-05	0.00621615		
TCONS_00080427	XLOC_039184	14	18618635	18618709	Inf	NA	5.00E-05	0.00621615	Epgn	Signaling
TCONS_00080700	XLOC_039453	14	30039438	30040322	#NAME?	NA	5.00E-05	0.00621615		

TCONS_00080883	XLOC_039628	14	34890338	34890414	Inf	NA	5.00E-05	0.00621615		
TCONS_00081253	XLOC_039941	14	43865904	43865980	Inf	NA	5.00E-05	0.00621615		
TCONS_00081731	XLOC_040412	14	59851251	59851327	Inf	NA	5.00E-05	0.00621615		
TCONS_00081871	XLOC_040552	14	65991987	65993352	#NAME?	NA	5.00E-05	0.00621615		
TCONS_00081873	XLOC_040554	14	65994988	65995619	#NAME?	NA	5.00E-05	0.00621615		
TCONS_00081884	XLOC_040565	14	66010133	66012565	#NAME?	NA	5.00E-05	0.00621615		
TCONS_00081893	XLOC_040574	14	66027465	66028626	#NAME?	NA	5.00E-05	0.00621615		
TCONS_00081908	XLOC_040589	14	66059730	66060422	#NAME?	NA	5.00E-05	0.00621615		
TCONS_00082275	XLOC_040955	14	76492001	76492077	Inf	NA	5.00E-05	0.00621615		
TCONS_00082301	XLOC_040981	14	76954528	76954604	Inf	NA	5.00E-05	0.00621615		
TCONS_00082901	XLOC_041571	14	94631229	94631540	#NAME?	NA	5.00E-05	0.00621615		
TCONS_00083309	XLOC_041977	14	106280388	106280667	Inf	NA	5.00E-05	0.00621615		
TCONS_00083340	XLOC_042008	14	106792876	106792950	Inf	NA	5.00E-05	0.00621615		
TCONS_00083417	XLOC_042085	14	108055094	108055170	Inf	NA	5.00E-05	0.00621615	Xpo1	Metabolism
TCONS_00083442	XLOC_042106	14	108550425	108550599	Inf	NA	5.00E-05	0.00621615		
TCONS_00083635	XLOC_042294	14	113520336	113520412	Inf	NA	5.00E-05	0.00621615		
TCONS_00083661	XLOC_042320	14	113941717	113941794	Inf	NA	5.00E-05	0.00621615	Ccdc88a;Prorsd1;Mtif2	Signaling;Transcription
TCONS_00084487	XLOC_042519	15	26300363	26312247	-3.68876	-4.434	5.00E-05	0.00621615	7SK	
TCONS_00084538	XLOC_042540	15	27931910	27937664	6.11855	4.48269	5.00E-05	0.00621615	Rnase10	Transcription
TCONS_00084787	XLOC_042656	15	33102461	33104324	2.08316	2.91246	5.00E-05	0.00621615	Lrp10	Receptor
TCONS_00085824	XLOC_042923	15	61467713	61476673	3.04908	2.97518	5.00E-05	0.00621615		
TCONS_00086086	XLOC_043002	15	90262284	90263086	Inf	NA	5.00E-05	0.00621615	Mycbp2	Metabolism
TCONS_00089525	XLOC_044236	15	7810660	7810930	#NAME?	NA	5.00E-05	0.00621615		
TCONS_00089594	XLOC_044305	15	8441653	8442086	#NAME?	NA	5.00E-05	0.00621615		
TCONS_00089666	XLOC_044375	15	9632128	9632607	#NAME?	NA	5.00E-05	0.00621615		
TCONS_00089690	XLOC_044399	15	9737709	9737916	#NAME?	NA	5.00E-05	0.00621615		
TCONS_00089715	XLOC_044424	15	9793350	9799701	-3.11934	-2.95887	5.00E-05	0.00621615		
TCONS_00089727	XLOC_044436	15	9825357	9825938	#NAME?	NA	5.00E-05	0.00621615		
TCONS_00089728	XLOC_044437	15	9826004	9826407	#NAME?	NA	5.00E-05	0.00621615		
TCONS_00089731	XLOC_044440	15	9875698	9876157	#NAME?	NA	5.00E-05	0.00621615		
TCONS_00089734	XLOC_044443	15	9883524	9884002	#NAME?	NA	5.00E-05	0.00621615		
TCONS_00089736	XLOC_044445	15	9886090	9887255	#NAME?	NA	5.00E-05	0.00621615		
TCONS_00089737	XLOC_044446	15	9890896	9891577	#NAME?	NA	5.00E-05	0.00621615		
TCONS_00089755	XLOC_044463	15	9937074	9937685	#NAME?	NA	5.00E-05	0.00621615		
TCONS_00089771	XLOC_044477	15	9974392	9975010	#NAME?	NA	5.00E-05	0.00621615		
TCONS_00089772	XLOC_044478	15	9975118	9985448	-4.22818	-3.7114	5.00E-05	0.00621615		
TCONS_00089777	XLOC_044483	15	9994529	9995534	#NAME?	NA	5.00E-05	0.00621615		
TCONS_00089797	XLOC_044502	15	10042642	10043068	#NAME?	NA	5.00E-05	0.00621615		
TCONS_00089805	XLOC_044510	15	10056023	10057472	#NAME?	NA	5.00E-05	0.00621615		
TCONS_00089810	XLOC_044515	15	10068876	10069496	#NAME?	NA	5.00E-05	0.00621615		
TCONS_00089817	XLOC_044521	15	10084873	10085898	#NAME?	NA	5.00E-05	0.00621615		
TCONS_00089853	XLOC_044557	15	11095449	11096823	Inf	NA	5.00E-05	0.00621615	AABR07017104.1	
TCONS_00089857	XLOC_044561	15	11103410	11104597	Inf	NA	5.00E-05	0.00621615		
TCONS_00089876	XLOC_044580	15	11131042	11131840	Inf	NA	5.00E-05	0.00621615		
TCONS_00089906	XLOC_044610	15	11512654	11512730	Inf	NA	5.00E-05	0.00621615		
TCONS_00089915	XLOC_044619	15	12036511	12036961	#NAME?	NA	5.00E-05	0.00621615		
TCONS_00090759	XLOC_045325	15	24432731	24432806	Inf	NA	5.00E-05	0.00621615		
TCONS_00090876	XLOC_045442	15	26331217	26338544	-4.97987	-4.16611	5.00E-05	0.00621615		
TCONS_00090877	XLOC_045443	15	26356147	26356612	#NAME?	NA	5.00E-05	0.00621615		
TCONS_00090890	XLOC_045456	15	26429920	26431537	#NAME?	NA	5.00E-05	0.00621615		
TCONS_00090891	XLOC_045457	15	26431596	26432245	#NAME?	NA	5.00E-05	0.00621615		
TCONS_00091035	XLOC_045601	15	32952503	32952904	#NAME?	NA	5.00E-05	0.00621615	LOC102550121	
TCONS_00091403	XLOC_045961	15	43595174	43595250	Inf	NA	5.00E-05	0.00621615	Pnma2	Immune
TCONS_00091603	XLOC_046160	15	49271697	49271773	Inf	NA	5.00E-05	0.00621615		
TCONS_00091734	XLOC_046291	15	51409123	51409198	Inf	NA	5.00E-05	0.00621615	Chmp7;AABR07018321.1	Binding Protein
TCONS_00091758	XLOC_046315	15	52323546	52324384	#NAME?	NA	5.00E-05	0.00621615	Dmtn;Fgf17	Signaling
TCONS_00092078	XLOC_046634	15	56256834	56256913	Inf	NA	5.00E-05	0.00621615		
TCONS_00092202	XLOC_046757	15	58210115	58212663	-3.49936	-3.06079	5.00E-05	0.00621615	Nufip1	Transcription
TCONS_00092206	XLOC_046761	15	58220326	58227781	-3.03289	-3.10585	5.00E-05	0.00621615		
TCONS_00092208	XLOC_046763	15	58230187	58230528	#NAME?	NA	5.00E-05	0.00621615		
TCONS_00092216	XLOC_046771	15	58268394	58269436	#NAME?	NA	5.00E-05	0.00621615		
TCONS_00092218	XLOC_046773	15	58271055	58271669	#NAME?	NA	5.00E-05	0.00621615		
TCONS_00092379	XLOC_046933	15	59504444	59504520	Inf	NA	5.00E-05	0.00621615		
TCONS_00092770	XLOC_047310	15	70892651	70893516	#NAME?	NA	5.00E-05	0.00621615		

TCONS_00092785	XLOC_047325	15	71126598	71126674	Inf	NA	5.00E-05	0.00621615		
TCONS_00092970	XLOC_047510	15	76129929	76130094	Inf	NA	5.00E-05	0.00621615		
TCONS_00093154	XLOC_047694	15	77716032	77716738	#NAME?	NA	5.00E-05	0.00621615		
TCONS_00093165	XLOC_047705	15	77728955	77729473	#NAME?	NA	5.00E-05	0.00621615	Pcdh9	Extracellular Matrix
TCONS_00093430	XLOC_047970	15	87582957	87588573	2.5074	2.80986	5.00E-05	0.00621615	Mycbp2	Metabolism
TCONS_00093588	XLOC_048126	15	91700827	91700903	Inf	NA	5.00E-05	0.00621615	Mycbp2	Metabolism
TCONS_00093668	XLOC_048206	15	95302282	95302357	Inf	NA	5.00E-05	0.00621615		
TCONS_00093728	XLOC_048266	15	98456263	98456356	Inf	NA	5.00E-05	0.00621615		
TCONS_00094043	XLOC_048581	15	104799772	104799848	Inf	NA	5.00E-05	0.00621615	Hs6st3	Metabolism
TCONS_00094083	XLOC_048621	15	104964512	104964588	Inf	NA	5.00E-05	0.00621615	Hs6st3	Metabolism
TCONS_00094134	XLOC_048672	15	105359670	105359743	Inf	NA	5.00E-05	0.00621615	AABR07019435.1	
TCONS_00095408	XLOC_049005	16	26906715	27014813	-2.04876	-2.62059	5.00E-05	0.00621615	Cpe;5S_rRNA	Protease
TCONS_00095532	XLOC_049056	16	47607701	47608424	#NAME?	NA	5.00E-05	0.00621615		
TCONS_00096557	XLOC_049304	16	83522161	83632153	-2.59217	-3.20235	5.00E-05	0.00621615	Col4a1	Cytoskeleton
TCONS_00098446	XLOC_049781	16	73372006	73410777	-2.0183	-2.8262	5.00E-05	0.00621615	Sfrp1	Signaling
TCONS_00098597	XLOC_049819	16	75664985	75668203	5.59767	5.3013	5.00E-05	0.00621615	AC114391.1;Spag11a	Immune
TCONS_00098829	XLOC_049881	16	83386408	83522127	-2.43464	-2.62151	5.00E-05	0.00621615	Rab20;Col4a2;Col4a1	Signaling;Cytoskeleton
TCONS_00099537	XLOC_050550	16	14544316	14544392	Inf	NA	5.00E-05	0.00621615		
TCONS_00099924	XLOC_050934	16	22788385	22788461	Inf	NA	5.00E-05	0.00621615		
TCONS_00099954	XLOC_050964	16	23359125	23359791	Inf	NA	5.00E-05	0.00621615		
TCONS_00099979	XLOC_050989	16	23926578	23927047	#NAME?	NA	5.00E-05	0.00621615		
TCONS_00099981	XLOC_050991	16	23930398	23930768	Inf	NA	5.00E-05	0.00621615		
TCONS_00100031	XLOC_051041	16	24771736	24771936	Inf	NA	5.00E-05	0.00621615	Npy1r	Receptor
TCONS_00100055	XLOC_051065	16	27034084	27034160	Inf	NA	5.00E-05	0.00621615		
TCONS_00100071	XLOC_051081	16	28749404	28749479	Inf	NA	5.00E-05	0.00621615		
TCONS_00100117	XLOC_051125	16	31988524	31988953	#NAME?	NA	5.00E-05	0.00621615		
TCONS_00100129	XLOC_051137	16	32234123	32234677	Inf	NA	5.00E-05	0.00621615	Sh3rf1	
TCONS_00100158	XLOC_051166	16	32724973	32725049	Inf	NA	5.00E-05	0.00621615		
TCONS_00100364	XLOC_051372	16	39568610	39568976	Inf	NA	5.00E-05	0.00621615	Gpm6a	Cytoskeleton
TCONS_00100391	XLOC_051399	16	39608787	39609284	Inf	NA	5.00E-05	0.00621615	Gpm6a;Rn60_16_0397.1	Cytoskeleton
TCONS_00100403	XLOC_051411	16	39620872	39621385	Inf	NA	5.00E-05	0.00621615	Gpm6a;Rn60_16_0397.1	Cytoskeleton
TCONS_00100418	XLOC_051426	16	39644516	39645013	Inf	NA	5.00E-05	0.00621615	Gpm6a	Cytoskeleton
TCONS_00100430	XLOC_051438	16	39656886	39658191	Inf	NA	5.00E-05	0.00621615	Gpm6a	Cytoskeleton
TCONS_00100440	XLOC_051448	16	39673925	39675084	Inf	NA	5.00E-05	0.00621615	Gpm6a	Cytoskeleton
TCONS_00100455	XLOC_051463	16	39690085	39690393	Inf	NA	5.00E-05	0.00621615	Gpm6a	Cytoskeleton
TCONS_00100495	XLOC_051503	16	41063086	41063404	Inf	NA	5.00E-05	0.00621615		
TCONS_00100558	XLOC_051566	16	44987825	44988794	#NAME?	NA	5.00E-05	0.00621615		
TCONS_00100802	XLOC_051792	16	49070706	49076855	-3.86193	-3.86174	5.00E-05	0.00621615	SNORA26	
TCONS_00100805	XLOC_051795	16	49092410	49092695	#NAME?	NA	5.00E-05	0.00621615		
TCONS_00100823	XLOC_051813	16	49277187	49277265	Inf	NA	5.00E-05	0.00621615	Slc25a4;Cfap97	Transport
TCONS_00100979	XLOC_051957	16	53024850	53024924	Inf	NA	5.00E-05	0.00621615		
TCONS_00100984	XLOC_051962	16	53083917	53083991	Inf	NA	5.00E-05	0.00621615		
TCONS_00100997	XLOC_051975	16	53196059	53196454	Inf	NA	5.00E-05	0.00621615		
TCONS_00101017	XLOC_051995	16	53234927	53237639	Inf	NA	5.00E-05	0.00621615		
TCONS_00101033	XLOC_052011	16	53258700	53259684	Inf	NA	5.00E-05	0.00621615		
TCONS_00101039	XLOC_052017	16	53266993	53267335	Inf	NA	5.00E-05	0.00621615		
TCONS_00101041	XLOC_052019	16	53268620	53269349	Inf	NA	5.00E-05	0.00621615		
TCONS_00101044	XLOC_052022	16	53274969	53276092	Inf	NA	5.00E-05	0.00621615		
TCONS_00101049	XLOC_052027	16	53278324	53280768	Inf	NA	5.00E-05	0.00621615		
TCONS_00101054	XLOC_052032	16	53284484	53285053	Inf	NA	5.00E-05	0.00621615		
TCONS_00101056	XLOC_052034	16	53286126	53286671	Inf	NA	5.00E-05	0.00621615		
TCONS_00101057	XLOC_052035	16	53286935	53288307	Inf	NA	5.00E-05	0.00621615		
TCONS_00101061	XLOC_052039	16	53291370	53292469	Inf	NA	5.00E-05	0.00621615		
TCONS_00101068	XLOC_052046	16	53298258	53299095	Inf	NA	5.00E-05	0.00621615		
TCONS_00101199	XLOC_052174	16	58551203	58551278	Inf	NA	5.00E-05	0.00621615		
TCONS_00101610	XLOC_052582	16	72257164	72257668	#NAME?	NA	5.00E-05	0.00621615		
TCONS_00101612	XLOC_052584	16	72291485	72292241	#NAME?	NA	5.00E-05	0.00621615	Ido2	
TCONS_00101651	XLOC_052623	16	72576699	72576775	Inf	NA	5.00E-05	0.00621615		
TCONS_00101685	XLOC_052657	16	73340359	73340435	Inf	NA	5.00E-05	0.00621615		
TCONS_00101911	XLOC_052882	16	81487345	81487696	#NAME?	NA	5.00E-05	0.00621615	LOC688801;Cdc16	Metabolism
TCONS_00101960	XLOC_052930	16	83642113	83642293	Inf	NA	5.00E-05	0.00621615	Col4a1	Cytoskeleton

TCONS_00102013	XLOC_052983	16	84993965	84994040	Inf	NA	5.00E-05	0.00621615		
TCONS_00102062	XLOC_053032	16	90358676	90359209	#NAME?	NA	5.00E-05	0.00621615		
TCONS_00102455	XLOC_053134	17	9109730	9117750	-3.83091	-3.93927	5.00E-05	0.00621615	Cxcl14	Growth Factors & Cytokines
TCONS_00103998	XLOC_053516	17	61035131	61070599	2.16861	2.76145	5.00E-05	0.00621615	SCARNA20	
TCONS_00104927	XLOC_053707	17	5063250	5081098	2.49867	2.93361	5.00E-05	0.00621615		
TCONS_00105177	XLOC_053766	17	10401617	10450489	3.78254	3.8622	5.00E-05	0.00621615	Sncb;Gprin1;Cdhr2;AABR07027007.1;Rnf44	Protein Binding;Extracellular Matrix;Proteolysis
TCONS_00105429	XLOC_053847	17	16690614	16695750	-3.03931	-3.26415	5.00E-05	0.00621615	Id4	Epigenetic
TCONS_00105492	XLOC_053869	17	19543215	19580985	-2.33668	-2.87198	5.00E-05	0.00621615	Atxn1;AABR07027212.1;Gmpr	Transcription;Metabolism
TCONS_00107051	XLOC_054280	17	80954994	81003261	3.44419	3.90301	5.00E-05	0.00621615	St8sia6	Metabolism
TCONS_00107419	XLOC_054416	17	1222121	1222196	Inf	NA	5.00E-05	0.00621615		
TCONS_00107720	XLOC_054709	17	5038759	5047645	2.39534	2.49148	5.00E-05	0.00621615		
TCONS_00107725	XLOC_054714	17	5082846	5089620	2.08524	2.32022	5.00E-05	0.00621615		
TCONS_00107769	XLOC_054757	17	6279343	6279632	#NAME?	NA	5.00E-05	0.00621615		
TCONS_00107820	XLOC_054808	17	6567565	6567640	Inf	NA	5.00E-05	0.00621615		
TCONS_00107876	XLOC_054864	17	7883784	7884525	Inf	NA	5.00E-05	0.00621615	AABR07026971.1	
TCONS_00108033	XLOC_055021	17	11940608	11940772	-2.0715	-2.55073	5.00E-05	0.00621615		
TCONS_00108039	XLOC_055027	17	12475840	12485317	-3.4035	-3.48231	5.00E-05	0.00621615		
TCONS_00108055	XLOC_055043	17	12552294	12552716	#NAME?	NA	5.00E-05	0.00621615		
TCONS_00108093	XLOC_055081	17	13211089	13211165	Inf	NA	5.00E-05	0.00621615		
TCONS_00108425	XLOC_055405	17	17622558	17622634	Inf	NA	5.00E-05	0.00621615		
TCONS_00108494	XLOC_055474	17	18932089	18932405	Inf	NA	5.00E-05	0.00621615		
TCONS_00108540	XLOC_055515	17	19759471	19759547	Inf	NA	5.00E-05	0.00621615		
TCONS_00108558	XLOC_055533	17	19819387	19819463	Inf	NA	5.00E-05	0.00621615		
TCONS_00108573	XLOC_055548	17	19937692	19938282	Inf	NA	5.00E-05	0.00621615		
TCONS_00108578	XLOC_055553	17	19943619	19944187	Inf	NA	5.00E-05	0.00621615		
TCONS_00108579	XLOC_055554	17	19944993	19945553	Inf	NA	5.00E-05	0.00621615		
TCONS_00108731	XLOC_055696	17	23456429	23456755	#NAME?	NA	5.00E-05	0.00621615		
TCONS_00108747	XLOC_055712	17	23550238	23550314	Inf	NA	5.00E-05	0.00621615		
TCONS_00108763	XLOC_055728	17	23565632	23565824	Inf	NA	5.00E-05	0.00621615		
TCONS_00108813	XLOC_055777	17	24328772	24328849	Inf	NA	5.00E-05	0.00621615		
TCONS_00108818	XLOC_055782	17	24338959	24339033	Inf	NA	5.00E-05	0.00621615		
TCONS_00108834	XLOC_055798	17	24399141	24399329	Inf	NA	5.00E-05	0.00621615		
TCONS_00108835	XLOC_055799	17	24399952	24400495	Inf	NA	5.00E-05	0.00621615		
TCONS_00109006	XLOC_055969	17	28263849	28263924	Inf	NA	5.00E-05	0.00621615		
TCONS_00109041	XLOC_056004	17	30404897	30404972	Inf	NA	5.00E-05	0.00621615		
TCONS_00109081	XLOC_056044	17	30735174	30735250	Inf	NA	5.00E-05	0.00621615		
TCONS_00109137	XLOC_056095	17	32239052	32239493	#NAME?	NA	5.00E-05	0.00621615		
TCONS_00109189	XLOC_056147	17	34018455	34018529	Inf	NA	5.00E-05	0.00621615		
TCONS_00109294	XLOC_056252	17	36050670	36050913	Inf	NA	5.00E-05	0.00621615		
TCONS_00109354	XLOC_056311	17	37425201	37425277	Inf	NA	5.00E-05	0.00621615		
TCONS_00109391	XLOC_056346	17	37683510	37683987	Inf	NA	5.00E-05	0.00621615	RGD1563300;AABR07027581.1	
TCONS_00109524	XLOC_056479	17	38368600	38368675	Inf	NA	5.00E-05	0.00621615		
TCONS_00109619	XLOC_056574	17	40544277	40544352	Inf	NA	5.00E-05	0.00621615		
TCONS_00109622	XLOC_056577	17	40569187	40569295	Inf	NA	5.00E-05	0.00621615		
TCONS_00109666	XLOC_056619	17	42033010	42033479	#NAME?	NA	5.00E-05	0.00621615	Dcdc2	Development
TCONS_00110041	XLOC_056992	17	46814203	46814828	Inf	NA	5.00E-05	0.00621615		
TCONS_00110724	XLOC_057634	17	56282187	56282998	Inf	NA	5.00E-05	0.00621615		
TCONS_00110902	XLOC_057810	17	60536821	60536896	Inf	NA	5.00E-05	0.00621615	Mkx	Epigenetic
TCONS_00110956	XLOC_057864	17	61255517	61264690	-2.02794	-2.31406	5.00E-05	0.00621615		
TCONS_00110965	XLOC_057873	17	61775049	61775124	Inf	NA	5.00E-05	0.00621615		
TCONS_00111277	XLOC_058109	17	64473250	64473856	#NAME?	NA	5.00E-05	0.00621615		
TCONS_00111294	XLOC_058126	17	64931627	64931824	Inf	NA	5.00E-05	0.00621615		
TCONS_00111326	XLOC_058158	17	66807331	66807962	Inf	NA	5.00E-05	0.00621615		
TCONS_00111369	XLOC_058201	17	67266311	67266902	Inf	NA	5.00E-05	0.00621615		
TCONS_00111591	XLOC_058420	17	75084268	75084557	#NAME?	NA	5.00E-05	0.00621615		
TCONS_00111612	XLOC_058441	17	75118016	75118558	#NAME?	NA	5.00E-05	0.00621615		
TCONS_00111970	XLOC_058796	17	81015081	81015617	Inf	NA	5.00E-05	0.00621615		
TCONS_00111973	XLOC_058799	17	81018802	81026319	5.10754	4.28648	5.00E-05	0.00621615		
TCONS_00111974	XLOC_058800	17	81026397	81027861	4.69143	3.58206	5.00E-05	0.00621615		
TCONS_00111982	XLOC_058808	17	81063355	81064793	Inf	NA	5.00E-05	0.00621615		

TCONS_00111991	XLOC_058817	17	81094441	81095225	Inf	NA	5.00E-05	0.00621615		
TCONS_00111994	XLOC_058820	17	81101207	81102782	Inf	NA	5.00E-05	0.00621615		
TCONS_00111995	XLOC_058821	17	81102843	81103523	Inf	NA	5.00E-05	0.00621615		
TCONS_00112064	XLOC_058863	17	83206836	83206912	Inf	NA	5.00E-05	0.00621615		
TCONS_00112068	XLOC_058867	17	84071484	84071559	Inf	NA	5.00E-05	0.00621615		
TCONS_00112163	XLOC_058962	17	86794291	86794367	Inf	NA	5.00E-05	0.00621615		
TCONS_00112349	XLOC_059148	17	90790840	90791013	Inf	NA	5.00E-05	0.00621615	7SK	
TCONS_00114066	XLOC_059552	18	62115224	62119363	Inf	NA	5.00E-05	0.00621615		
TCONS_00114560	XLOC_059670	18	80149986	80150902	Inf	NA	5.00E-05	0.00621615		
TCONS_00114924	XLOC_059775	18	16418937	16428109	3.47531	3.30402	5.00E-05	0.00621615	MGC116121	
TCONS_00115933	XLOC_060023	18	59618068	59618540	Inf	NA	5.00E-05	0.00621615		
TCONS_00116170	XLOC_060097	18	70901502	70924708	-2.07907	-2.80106	5.00E-05	0.00621615	Lipg	Metabolism
TCONS_00116596	XLOC_060334	18	3043516	3043829	#NAME?	NA	5.00E-05	0.00621615		
TCONS_00116618	XLOC_060356	18	3095138	3095479	#NAME?	NA	5.00E-05	0.00621615		
TCONS_00116716	XLOC_060454	18	6553017	6553093	Inf	NA	5.00E-05	0.00621615		
TCONS_00116726	XLOC_060464	18	6574731	6574806	Inf	NA	5.00E-05	0.00621615		
TCONS_00116867	XLOC_060604	18	15043347	15043533	Inf	NA	5.00E-05	0.00621615		
TCONS_00116925	XLOC_060662	18	16391644	16402586	3.10888	3.14563	5.00E-05	0.00621615	Mir187	
TCONS_00116926	XLOC_060663	18	16402674	16408952	2.80731	2.73403	5.00E-05	0.00621615		
TCONS_00117124	XLOC_060861	18	23282983	23283059	Inf	NA	5.00E-05	0.00621615		
TCONS_00117365	XLOC_061070	18	28348184	28348260	Inf	NA	5.00E-05	0.00621615	SNORA74;Mir1949;AC135285.5	
TCONS_00117667	XLOC_061368	18	32214221	32214296	Inf	NA	5.00E-05	0.00621615	Arhgap26	Signaling
TCONS_00117670	XLOC_061371	18	32225361	32225444	Inf	NA	5.00E-05	0.00621615		
TCONS_00117760	XLOC_061461	18	33274936	33275011	Inf	NA	5.00E-05	0.00621615		
TCONS_00117801	XLOC_061502	18	35018484	35018560	Inf	NA	5.00E-05	0.00621615		
TCONS_00117802	XLOC_061503	18	35021921	35021997	Inf	NA	5.00E-05	0.00621615		
TCONS_00117815	XLOC_061516	18	36008578	36008806	Inf	NA	5.00E-05	0.00621615		
TCONS_00117816	XLOC_061517	18	36041251	36041471	Inf	NA	5.00E-05	0.00621615		
TCONS_00117858	XLOC_061559	18	36894273	36894407	Inf	NA	5.00E-05	0.00621615	Tcerg1;uc_338;Gpr151	Transcription;Receptor
TCONS_00117937	XLOC_061638	18	39553666	39553742	Inf	NA	5.00E-05	0.00621615		
TCONS_00117940	XLOC_061641	18	39562145	39563093	Inf	NA	5.00E-05	0.00621615		
TCONS_00117942	XLOC_061643	18	39565729	39565805	Inf	NA	5.00E-05	0.00621615		
TCONS_00118063	XLOC_061764	18	39923020	39923096	Inf	NA	5.00E-05	0.00621615		
TCONS_00118194	XLOC_061895	18	41312728	41313509	Inf	NA	5.00E-05	0.00621615		
TCONS_00118283	XLOC_061984	18	43782656	43782730	Inf	NA	5.00E-05	0.00621615		
TCONS_00118310	XLOC_062011	18	44200260	44200375	Inf	NA	5.00E-05	0.00621615		
TCONS_00118312	XLOC_062013	18	44203438	44204097	Inf	NA	5.00E-05	0.00621615		
TCONS_00118317	XLOC_062018	18	44222294	44222858	Inf	NA	5.00E-05	0.00621615		
TCONS_00118319	XLOC_062020	18	44233566	44233912	Inf	NA	5.00E-05	0.00621615		
TCONS_00118328	XLOC_062029	18	44456284	44456359	Inf	NA	5.00E-05	0.00621615		
TCONS_00118329	XLOC_062030	18	44456430	44456506	Inf	NA	5.00E-05	0.00621615		
TCONS_00118415	XLOC_062116	18	46959538	46959921	Inf	NA	5.00E-05	0.00621615		
TCONS_00118429	XLOC_062130	18	48107817	48107893	Inf	NA	5.00E-05	0.00621615		
TCONS_00118532	XLOC_062232	18	52510793	52510869	Inf	NA	5.00E-05	0.00621615		
TCONS_00118650	XLOC_062350	18	57286114	57286190	Inf	NA	5.00E-05	0.00621615	Sh3tc2	Development
TCONS_00118772	XLOC_062472	18	61528998	61529827	Inf	NA	5.00E-05	0.00621615		
TCONS_00119121	XLOC_062807	18	69852335	69852411	Inf	NA	5.00E-05	0.00621615	Mro	
TCONS_00119494	XLOC_063106	18	79882682	79882758	Inf	NA	5.00E-05	0.00621615		
TCONS_00119533	XLOC_063145	18	80108598	80108763	Inf	NA	5.00E-05	0.00621615		
TCONS_00119552	XLOC_063164	18	80783370	80783446	Inf	NA	5.00E-05	0.00621615		
TCONS_00120734	XLOC_063589	19	31524670	31620405	-2.08741	-2.43396	5.00E-05	0.00621615	Hhip;uc_338	Receptor
TCONS_00121597	XLOC_063769	19	52647069	52705131	-2.08616	-2.66778	5.00E-05	0.00621615	Crispld2	
TCONS_00122182	XLOC_063916	19	10079764	10101451	-2.67645	-3.28407	5.00E-05	0.00621615	Mmp15;Usb1	Protease
TCONS_00122360	XLOC_063943	19	11307966	11308740	-3.68968	-4.1577	5.00E-05	0.00621615	AC128848.1;Mt2A	Receptor
TCONS_00122361	XLOC_063944	19	11324697	11326139	3.90815	3.538	5.00E-05	0.00621615	Mt3	
TCONS_00122423	XLOC_063964	19	15542671	15570611	-2.37295	-2.81455	5.00E-05	0.00621615	Lpcat2;Mmp2	Metabolism;Proteolysis
TCONS_00122687	XLOC_064015	19	23178252	23185878	Inf	NA	5.00E-05	0.00621615		
TCONS_00123625	XLOC_064220	19	43545377	43596801	-3.17061	-3.3552	5.00E-05	0.00621615	AABR07043844.1;Fa2h	Metabolism
TCONS_00124066	XLOC_064322	19	56674069	56677084	-4.90517	-4.19642	5.00E-05	0.00621615	Acta1;Nup133	Cytoskeleton
TCONS_00124678	XLOC_064703	19	12411222	12411753	#NAME?	NA	5.00E-05	0.00621615	AABR07042859.1	

TCONS_00124679	XLOC_064704	19	12412059	12412487	#NAME?	NA	5.00E-05	0.00621615	AABR07042859.1	
TCONS_00124855	XLOC_064879	19	17589833	17589953	Inf	NA	5.00E-05	0.00621615		
TCONS_00124870	XLOC_064894	19	17733751	17734313	#NAME?	NA	5.00E-05	0.00621615		
TCONS_00124906	XLOC_064930	19	17993894	17994323	#NAME?	NA	5.00E-05	0.00621615	AABR07043031.1	
TCONS_00124917	XLOC_064941	19	18003237	18003739	#NAME?	NA	5.00E-05	0.00621615		
TCONS_00125027	XLOC_065048	19	20096794	20102355	-3.35485	-3.02997	5.00E-05	0.00621615		
TCONS_00125047	XLOC_065068	19	20131517	20132008	#NAME?	NA	5.00E-05	0.00621615		
TCONS_00125101	XLOC_065122	19	21884400	21885111	#NAME?	NA	5.00E-05	0.00621615	SNORA17	
TCONS_00125258	XLOC_065279	19	24579383	24579790	#NAME?	NA	5.00E-05	0.00621615	Clgn;Scoc	Protein Binding;Golgi
TCONS_00125404	XLOC_065425	19	28448956	28449031	Inf	NA	5.00E-05	0.00621615		
TCONS_00125470	XLOC_065491	19	30706118	30706682	Inf	NA	5.00E-05	0.00621615	Usp38	Protease
TCONS_00125473	XLOC_065494	19	30707510	30708100	Inf	NA	5.00E-05	0.00621615	Usp38	Protease
TCONS_00125508	XLOC_065529	19	31298383	31298541	Inf	NA	5.00E-05	0.00621615		
TCONS_00125540	XLOC_065561	19	31341148	31341384	Inf	NA	5.00E-05	0.00621615		
TCONS_00125633	XLOC_065654	19	32186840	32187435	#NAME?	NA	5.00E-05	0.00621615	AC106702.1;Smad1	Signaling
TCONS_00125636	XLOC_065657	19	32276232	32276316	Inf	NA	5.00E-05	0.00621615		
TCONS_00125673	XLOC_065682	19	32653434	32653510	Inf	NA	5.00E-05	0.00621615		
TCONS_00125728	XLOC_065737	19	34019968	34020042	Inf	NA	5.00E-05	0.00621615	AABR07043652.1	
TCONS_00125781	XLOC_065783	19	34787028	34787104	Inf	NA	5.00E-05	0.00621615		
TCONS_00126135	XLOC_066121	19	45613183	45613258	Inf	NA	5.00E-05	0.00621615		
TCONS_00126335	XLOC_066309	19	47644989	47645065	Inf	NA	5.00E-05	0.00621615		
TCONS_00126518	XLOC_066492	19	52443029	52443330	Inf	NA	5.00E-05	0.00621615		
TCONS_00126566	XLOC_066540	19	53328123	53328295	Inf	NA	5.00E-05	0.00621615		
TCONS_00126621	XLOC_066591	19	54540935	54541011	Inf	NA	5.00E-05	0.00621615	AABR07044063.2	
TCONS_00126626	XLOC_066596	19	54686231	54686862	#NAME?	NA	5.00E-05	0.00621615	Slc7a5	Metabolism
TCONS_00126956	XLOC_066924	19	59765414	59765525	Inf	NA	5.00E-05	0.00621615		
TCONS_00151289	XLOC_081663	20	6923497	6933627	-2.58151	-3.45937	5.00E-05	0.00621615	Pi16;Mtch1	Binding Protein
TCONS_00151749	XLOC_081748	20	12773426	12802997	-2.55716	-3.4796	5.00E-05	0.00621615	Col6a2;Ftcd	Extracellular Matrix
TCONS_00154083	XLOC_082277	20	12805312	12820466	-2.39111	-2.76734	5.00E-05	0.00621615	Col6a2;Ftcd;LOC108348157	Extracellular Matrix
TCONS_00154979	XLOC_082474	20	44377271	44386733	5.1845	4.54389	5.00E-05	0.00621615		
TCONS_00154980	XLOC_082475	20	44387224	44394731	4.61003	4.20503	5.00E-05	0.00621615		
TCONS_00155382	XLOC_082688	20	3537924	3538000	Inf	NA	5.00E-05	0.00621615		
TCONS_00155435	XLOC_082740	20	5487843	5487918	Inf	NA	5.00E-05	0.00621615	Zbtb22;Daxx;AA926063	Transcription
TCONS_00155471	XLOC_082774	20	6259325	6259401	Inf	NA	5.00E-05	0.00621615	Stk38	Signaling
TCONS_00155797	XLOC_083077	20	13792809	13793175	Inf	NA	5.00E-05	0.00621615	Gstt4;Gstt1	Metabolism
TCONS_00156101	XLOC_083377	20	21478436	21478512	Inf	NA	5.00E-05	0.00621615		
TCONS_00156419	XLOC_083671	20	27785363	27808652	1.61856	2.48321	5.00E-05	0.00621615	Dse	
TCONS_00156622	XLOC_083857	20	30926378	30926938	Inf	NA	5.00E-05	0.00621615	Prf1	
TCONS_00156658	XLOC_083893	20	31621610	31622211	#NAME?	NA	5.00E-05	0.00621615		
TCONS_00156962	XLOC_084197	20	42957538	42957615	Inf	NA	5.00E-05	0.00621615	AABR07045321.2;Marcks	
TCONS_00156988	XLOC_084223	20	44332968	44333307	Inf	NA	5.00E-05	0.00621615		
TCONS_00157003	XLOC_084238	20	44353274	44353804	Inf	NA	5.00E-05	0.00621615		
TCONS_00157005	XLOC_084240	20	44355541	44356825	Inf	NA	5.00E-05	0.00621615		
TCONS_00157006	XLOC_084241	20	44357474	44358688	Inf	NA	5.00E-05	0.00621615		
TCONS_00157008	XLOC_084243	20	44360285	44360794	Inf	NA	5.00E-05	0.00621615		
TCONS_00157011	XLOC_084246	20	44362649	44363138	Inf	NA	5.00E-05	0.00621615		
TCONS_00157013	XLOC_084248	20	44364461	44365365	Inf	NA	5.00E-05	0.00621615		
TCONS_00157023	XLOC_084258	20	44396097	44399860	5.28577	4.87584	5.00E-05	0.00621615		
TCONS_00157024	XLOC_084259	20	44399995	44403356	4.90878	4.15607	5.00E-05	0.00621615		
TCONS_00157034	XLOC_084269	20	44655840	44656218	#NAME?	NA	5.00E-05	0.00621615		
TCONS_00157097	XLOC_084330	20	45304530	45305283	Inf	NA	5.00E-05	0.00621615	SNORA69	
TCONS_00157138	XLOC_084371	20	46333481	46334498	#NAME?	NA	5.00E-05	0.00621615		
TCONS_00157394	XLOC_084623	20	49298887	49298962	Inf	NA	5.00E-05	0.00621615		
TCONS_00157411	XLOC_084640	20	49634874	49634949	Inf	NA	5.00E-05	0.00621615		
TCONS_00157412	XLOC_084641	20	49681318	49681394	Inf	NA	5.00E-05	0.00621615		
TCONS_00157538	XLOC_084765	20	51561307	51561388	Inf	NA	5.00E-05	0.00621615		
TCONS_00157555	XLOC_084782	20	51760542	51760618	Inf	NA	5.00E-05	0.00621615		
TCONS_00157634	XLOC_084861	20	55826754	55826829	Inf	NA	5.00E-05	0.00621615		
TCONS_00273299	XLOC_148426	X	6430593	6533534	-2.57676	-3.01647	5.00E-05	0.00621615	Maob	Metabolism

TCONS_00275062	XLOC_148992	X	107634149	107635321	-3.94686	-3.83985	5.00E-05	0.00621615	Zcchc18	
TCONS_00275520	XLOC_149154	X	144445208	144447676	2.82948	3.23578	5.00E-05	0.00621615	RGD1561327	
TCONS_00276068	XLOC_149312	X	10932047	10932768	2.22785	3.06646	5.00E-05	0.00621615	Rn60_X_0110.2	
TCONS_00276310	XLOC_149388	X	18723340	18726732	-2.13812	-3.2259	5.00E-05	0.00621615	Ubqln2	Proteolysis
TCONS_00276664	XLOC_149490	X	40029085	40086882	-3.62445	-2.80548	5.00E-05	0.00621615	Smpx	Development
TCONS_00278350	XLOC_150070	X	157319045	157331204	-2.4414	-3.33266	5.00E-05	0.00621615	Atp2b3;Bgn	Transport;Receptor
TCONS_00278526	XLOC_150181	X	1629685	1630309	Inf	NA	5.00E-05	0.00621615		
TCONS_00278695	XLOC_150337	X	10237142	10237542	Inf	NA	5.00E-05	0.00621615		
TCONS_00278899	XLOC_150541	X	13188912	13189449	#NAME?	NA	5.00E-05	0.00621615		
TCONS_00278965	XLOC_150607	X	13812762	13812838	Inf	NA	5.00E-05	0.00621615		
TCONS_00278999	XLOC_150641	X	14141551	14141998	#NAME?	NA	5.00E-05	0.00621615	Srpx	
TCONS_00279158	XLOC_150795	X	19020547	19020623	Inf	NA	5.00E-05	0.00621615		
TCONS_00279494	XLOC_151127	X	25254325	25254681	Inf	NA	5.00E-05	0.00621615	AABR07037607.1	
TCONS_00279657	XLOC_151275	X	26827068	26833668	-2.24421	-2.43054	5.00E-05	0.00621615		
TCONS_00279893	XLOC_151476	X	31801067	31801143	Inf	NA	5.00E-05	0.00621615	Piga	Metabolism
TCONS_00279918	XLOC_151501	X	33254742	33254866	Inf	NA	5.00E-05	0.00621615		
TCONS_00279928	XLOC_151511	X	33691315	33691958	Inf	NA	5.00E-05	0.00621615		
TCONS_00279998	XLOC_151581	X	34087791	34087866	Inf	NA	5.00E-05	0.00621615		
TCONS_00280203	XLOC_151786	X	34430277	34430717	#NAME?	NA	5.00E-05	0.00621615		
TCONS_00280330	XLOC_151913	X	36780856	36780969	Inf	NA	5.00E-05	0.00621615		
TCONS_00280378	XLOC_151961	X	37158668	37159630	Inf	NA	5.00E-05	0.00621615		
TCONS_00280381	XLOC_151964	X	37161374	37161862	Inf	NA	5.00E-05	0.00621615		
TCONS_00280430	XLOC_152013	X	40026778	40028046	#NAME?	NA	5.00E-05	0.00621615	Smpx	Development
TCONS_00280434	XLOC_152017	X	40092001	40093364	#NAME?	NA	5.00E-05	0.00621615	Smpx	Development
TCONS_00280435	XLOC_152018	X	40093514	40094261	#NAME?	NA	5.00E-05	0.00621615	Smpx	Development
TCONS_00280767	XLOC_152350	X	54415406	54415818	Inf	NA	5.00E-05	0.00621615	RGD1565785	
TCONS_00280781	XLOC_152364	X	56964412	56964488	Inf	NA	5.00E-05	0.00621615		
TCONS_00280868	XLOC_152451	X	61298991	61299507	#NAME?	NA	5.00E-05	0.00621615		
TCONS_00280871	XLOC_152454	X	61302738	61303270	#NAME?	NA	5.00E-05	0.00621615		
TCONS_00280872	XLOC_152455	X	61305182	61305635	#NAME?	NA	5.00E-05	0.00621615		
TCONS_00280874	XLOC_152457	X	61307993	61308590	#NAME?	NA	5.00E-05	0.00621615		
TCONS_00280875	XLOC_152458	X	61309432	61310108	#NAME?	NA	5.00E-05	0.00621615		
TCONS_00280895	XLOC_152478	X	61363508	61365787	#NAME?	NA	5.00E-05	0.00621615		
TCONS_00280908	XLOC_152491	X	61395471	61396139	#NAME?	NA	5.00E-05	0.00621615		
TCONS_00280935	XLOC_152518	X	61438212	61439164	#NAME?	NA	5.00E-05	0.00621615		
TCONS_00280936	XLOC_152519	X	61439532	61440149	#NAME?	NA	5.00E-05	0.00621615		
TCONS_00280951	XLOC_152534	X	61474978	61476410	#NAME?	NA	5.00E-05	0.00621615		
TCONS_00280959	XLOC_152542	X	61486084	61490769	-2.90035	-2.73463	5.00E-05	0.00621615		
TCONS_00280975	XLOC_152558	X	61520047	61531666	-4.72982	-4.40372	5.00E-05	0.00621615		
TCONS_00280977	XLOC_152560	X	61533741	61540125	-3.75296	-3.43668	5.00E-05	0.00621615		
TCONS_00280983	XLOC_152566	X	61549036	61549639	#NAME?	NA	5.00E-05	0.00621615		
TCONS_00280990	XLOC_152573	X	61570519	61580945	-3.63951	-3.09791	5.00E-05	0.00621615		
TCONS_00280993	XLOC_152576	X	61586764	61587199	#NAME?	NA	5.00E-05	0.00621615		
TCONS_00281017	XLOC_152600	X	61653888	61654615	#NAME?	NA	5.00E-05	0.00621615		
TCONS_00281020	XLOC_152603	X	61656490	61663714	-5.05091	-3.74201	5.00E-05	0.00621615		
TCONS_00281022	XLOC_152605	X	61665237	61665554	#NAME?	NA	5.00E-05	0.00621615		
TCONS_00281035	XLOC_152618	X	61714219	61714625	#NAME?	NA	5.00E-05	0.00621615		
TCONS_00281057	XLOC_152640	X	61749884	61750791	#NAME?	NA	5.00E-05	0.00621615		
TCONS_00281065	XLOC_152648	X	61761150	61761656	#NAME?	NA	5.00E-05	0.00621615		
TCONS_00281077	XLOC_152660	X	61774041	61774677	#NAME?	NA	5.00E-05	0.00621615		
TCONS_00281078	XLOC_152661	X	61775825	61776630	#NAME?	NA	5.00E-05	0.00621615		
TCONS_00281180	XLOC_152763	X	64524945	64525082	Inf	NA	5.00E-05	0.00621615	Rn50_X_0655.1	
TCONS_00281181	XLOC_152764	X	64530201	64530276	Inf	NA	5.00E-05	0.00621615	Rn50_X_0655.1	
TCONS_00281488	XLOC_153061	X	71609295	71609370	Inf	NA	5.00E-05	0.00621615	AABR07039203.1;Cxcr3	Receptor
TCONS_00281790	XLOC_153360	X	75606445	75606692	Inf	NA	5.00E-05	0.00621615		
TCONS_00282017	XLOC_153578	X	76325638	76325713	Inf	NA	5.00E-05	0.00621615	Mir325	
TCONS_00282117	XLOC_153678	X	78160626	78161187	Inf	NA	5.00E-05	0.00621615		
TCONS_00282122	XLOC_153683	X	78171400	78172365	Inf	NA	5.00E-05	0.00621615		
TCONS_00282224	XLOC_153785	X	79201215	79203447	4.17564	3.30824	5.00E-05	0.00621615		
TCONS_00282324	XLOC_153874	X	80430076	80430152	Inf	NA	5.00E-05	0.00621615		
TCONS_00282380	XLOC_153930	X	84342529	84342972	Inf	NA	5.00E-05	0.00621615		
TCONS_00282955	XLOC_154493	X	100654141	100654922	Inf	NA	5.00E-05	0.00621615		
TCONS_00283197	XLOC_154733	X	107252407	107252683	Inf	NA	5.00E-05	0.00621615		
TCONS_00283198	XLOC_154734	X	107257915	107257991	Inf	NA	5.00E-05	0.00621615		
TCONS_00283200	XLOC_154736	X	107262004	107262080	Inf	NA	5.00E-05	0.00621615		

TCONS_00283516	XLOC_155019	X	112476547	112477068	#NAME?	NA	5.00E-05	0.00621615	Col4a6	Extracellular Matrix
TCONS_00283517	XLOC_155020	X	112479795	112480162	#NAME?	NA	5.00E-05	0.00621615	Col4a6	Extracellular Matrix
TCONS_00283570	XLOC_155070	X	112612764	112613311	#NAME?	NA	5.00E-05	0.00621615		
TCONS_00283766	XLOC_155239	X	116067885	116067961	Inf	NA	5.00E-05	0.00621615		
TCONS_00283814	XLOC_155287	X	118617375	118617451	Inf	NA	5.00E-05	0.00621615	Lrch2	Unknown
TCONS_00283925	XLOC_155397	X	124039568	124040392	#NAME?	NA	5.00E-05	0.00621615	LOC691272;Rn60_X_1241.1	
TCONS_00283927	XLOC_155399	X	124042078	124042432	#NAME?	NA	5.00E-05	0.00621615	LOC691272;Rn60_X_1241.1	
TCONS_00284026	XLOC_155498	X	127561284	127561360	Inf	NA	5.00E-05	0.00621615	Gria3	Signaling
TCONS_00284502	XLOC_155974	X	144877547	144877619	Inf	NA	5.00E-05	0.00621615		
TCONS_00284655	XLOC_156127	X	153040622	153041157	Inf	NA	5.00E-05	0.00621615	Pnma5;Zfp185	Immune;Transcription
TCONS_00284793	XLOC_156265	X	155492429	155492505	Inf	NA	5.00E-05	0.00621615		

Supplemental Table S4B
F1 sncRNA p<1e-04

Identification	Chr	Start	Stop	baseMean	log2Fold Change	minP	Gene Association	Gene Category
Rattus_norvegicus_chr1.trna12832-LeuTAG:(197697796	1	190773257	190773339	133.618688	4.818283796	3.99E-07	Eef2k	Signaling
piR-rno-62781	1	115716371	115716391	494.566869	-6.169431212	8.74E-09		
piR-rno-62813	1	11974004	11974030	954.748932	-6.044048127	8.77E-12	AABR07000398.1;5_8S_rRNA;AABR07000402.1;AABR07000404.1;LOC100909599	
piR-rno-62895	1	11908598	11908625	128.99895	-6.185245572	4.00E-10	5_8S_rRNA;pRNA	
piR-rno-62901	1	11908429	11908454	82.3763496	-3.044385265	4.09E-07	5_8S_rRNA;pRNA	
piR-rno-62915	1	11968164	11968191	1520.99691	-3.702861987	4.65E-06	pRNA;AABR07000398.1;5_8S_rRNA;AABR07000402.1;AABR07000404.1	
ENSRNOT00000089120.1:ncrna:chromosome:Rnor:6.0:1:1	1	174133709	174133839	39.8787206	5.289230909	2.82E-06	Rpl27a;AABR07005004.1;SNORA3;AABR07005004.2;St5	Translation
ENSRNOT00000090108.1:ncrna:chromosome:Rnor:6.0:1:2	1	216733116	216733243	29.8355834	5.268509676	3.73E-07	Nap1l4;SNORA54	Signaling
Rattus_norvegicus_chr2.trna7585-GlyCCC:(218004617-	2	198516709	198516780	200.897203	3.477627187	1.87E-05	U1	
Rattus_norvegicus_chr2.trna7579-GlyCCC:(218050757-	2	198562804	198562875	66.4543855	4.368127904	1.22E-06		
piR-rno-62762	2	38121642	38121664	168.00359	-3.862103638	2.38E-06	Dimt1	
piR-rno-62764	2	198638949	198638968	1304.36664	-5.892442321	9.01E-10		
ENSRNOT00000087544.1:ncrna:chromosome:Rnor:6.0:3:1	3	122806916	122806986	29.0228306	-4.439241088	6.16E-05	Nop56;SNORD110;SNORA51;SNORD86;SNORD56;SNORD57;Idh3B;RGD1561317	Transcription;Metabolism
ENSRNOT00000077844.1:ncrna:chromosome:Rnor:6.0:3:1	3	163817252	163817343	129.762777	-5.800523503	5.12E-08	Znfx1;SNORD12	Transcription
ENSRNOT00000086225.1:ncrna:chromosome:Rnor:6.0:3:1	3	11243814	11243899	36.0687276	4.052548355	3.37E-05	Prrc2b;SNORD62	
ENSRNOT00000088321.1:ncrna:chromosome:Rnor:6.0:3:4	3	5460452	5460534	11.5197314	-5.387131167	1.90E-05	Surf6;LOC100911917;Rpl7a;SNORD24;SNORD36;Surf4;LOC100912042	Development;Transcription;Translation
Rattus_norvegicus_chr10.trna705-GlyCCC:(15054081-1	4	118207496	118207567	144.516868	8.168667891	5.56E-11	Pcyox1;Tia1	Transcription
rno-miR-141-3p:MIMAT0000846	4	157236361	157236383	27.5012887	5.201721803	1.62E-05	Emg1;Phb2;snoU89;Mir141;Mir3575;Ptpn6	Development;Signaling
Rattus_norvegicus_chr4.trna1517-ArgCCT:(66087628-6	4	66276179	66276252	232.445289	4.511839502	7.85E-09	Fmc1	
Rattus_norvegicus_chr4.trna2752-GlyCCC:(182778746-	4	118207496	118207567	141.154025	6.637055382	1.14E-10	Pcyox1;Tia1	Transcription
piR-rno-41976	4	24253903	24253931	688.590055	-4.151728226	8.63E-06	Metazoa_SRP;AABR07059563.1	
Rattus_norvegicus_chr5.trna476-SerAGA:(28872791-28	5	23358376	23358458	82.2253542	-3.587718145	4.74E-05	4199	EST
piR-rno-41525	5	91134984	91135015	11263.7209	-3.882515395	4.63E-05	pRNA;5_8S_rRNA;AABR07048791.1	
piR-rno-62774	5	143998504	143998534	112765.102	-4.54497261	8.80E-06		
piR-rno-62843	5	91140702	91140730	67.4968605	-4.8366314	8.99E-07	5_8S_rRNA;AABR07048791.1	
piR-rno-40271	6	30633768	30633796	174.491377	-4.443210528	2.13E-07	5_8S_rRNA;AABR07063421.1;AABR07063424.1;LOC257642;AABR07063425.2;AABR07063425.1	
piR-rno-40521	6	30629568	30629599	58.7129843	-5.892284714	1.63E-11	5_8S_rRNA;AABR07063421.1;AABR07063424.1;LOC257642	
piR-rno-40686	6	30628861	30628892	1316.35893	-5.521809843	1.57E-07	5_8S_rRNA;AABR07063421.1;AABR07063424.1;LOC257642	
ENSRNOT00000078315.1:ncrna:chromosome:Rnor:6.0:6:1	6	115336928	115337071	10.8967825	4.600497183	2.27E-05	Gtf2a1;SNORA79	Transcription
Rattus_norvegicus_chr13.trna4025-AspGTC:(93938071-	7	34287931	34288003	225.097785	2.927991626	8.63E-05	Lta4h	Metabolism
ENSRNOT00000078194.1:ncrna:chromosome:Rnor:6.0:7:7	7	712846	712999	1644.94347	-5.077208765	6.06E-07	5_8S_rRNA	
piR-rno-62815	9	112905938	112905962	2956.25421	-3.294718435	5.82E-06	5S_rRNA	
rno-miR-21-5p:MIMAT0000790	10	73902263	73902285	44.4563262	4.819519903	4.19E-05	Mir21;Vmp1	
Rattus_norvegicus_chr10.trna13306-ArgCCT:(12698715	10	12877346	12877419	11.2381397	6.827128035	2.38E-06	Gm8225;Zfp213	Transcription
Rattus_norvegicus_chr10.trna13315-ArgCCT:(12671216	10	12849847	12849920	3.56339036	6.099857449	9.00E-05		
Rattus_norvegicus_chr10.trna2463-SerAGA:(55360576-	10	55617773	55617855	83.8907412	-4.06418392	6.98E-06	Ctc1;AC129753.2;Aurkb	Signaling
piR-rno-16896	10	15241138	15241166	334.228492	5.382641536	9.82E-08	Mcrip2;Mettl26;Wfikkn1;Rab40c	Signaling
piR-rno-62735	10	89570398	89570417	47.8636187	-4.240409	1.38E-06	U2;Arl4d	Translation
ENSRNOT00000089119.1:ncrna:chromosome:Rnor:6.0:11:	11	83915892	83915967	78.5254462	-4.228901696	1.69E-06	Eif4g1;SNORD66	Translation
Rattus_norvegicus_chr12.trna2269-AspGTC:(38501781-	12	36620338	36620410	223.355497	2.911080296	2.69E-05	Dhx37	Apoptosis
Rattus_norvegicus_chr13.trna4004-AspGTC:(94077340-	12	36620338	36620410	222.443499	2.971976554	1.27E-05	Dhx37	Apoptosis

ENSRNOT00000073261.1:ncrna:chromosome:Rnor:6.0:12:	12	1510400	1510518	8.99731142	-5.16271304	2.63E-05	5S_rRNA;AABR07034940.2;Rn5s;AABR07034940.1	
Rattus_norvegicus_chr13.trna2243-AspGTC:(94071463-	13	91233137	91233209	223.393172	2.655914564	7.00E-05	LOC100911825	Receptor
Rattus_norvegicus_chr13.trna3843-AspGTC:(95751497-	13	89403576	89403648	229.274295	2.864631346	4.34E-05	Fcgr2b;Fcgr3a	Immune
Rattus_norvegicus_chr13.trna3853-AspGTC:(95680683-	13	89403576	89403648	229.17478	2.845512892	3.37E-05	Fcgr2b;Fcgr3a	Immune
Rattus_norvegicus_chr13.trna4012-AspGTC:(94042774-	13	91182271	91182343	222.577892	2.857975177	4.67E-05	LOC100911825;LOC108348047	Receptor
Rattus_norvegicus_chr7.trna8266-AspGTC:(34353143-3	13	89449768	89449840	227.857951	2.913780864	3.06E-05		
Rattus_norvegicus_chr13.trna2225-LeuCAG:(93938654-	13	91182925	91183008	189.891044	6.648211677	6.97E-08	LOC100911825;LOC108348047	Receptor
Rattus_norvegicus_chr13.trna2370-LeuCAG:(95701090-	13	91233791	91233874	191.552226	5.930200874	5.38E-07	LOC100911825	Receptor
Rattus_norvegicus_chr13.trna2382-LeuCAG:(95752080-	13	91233791	91233874	191.869958	6.64933212	5.49E-08	LOC100911825	Receptor
Rattus_norvegicus_chr13.trna2365-LeuCAG:(95681267-	13	89467922	89468005	189.891998	7.330444867	1.05E-08		
piR-rno-20562	14	46524754	46524784	1144.2797	-3.604461158	4.87E-05	7642;AABR07015055.2;AABR07015056.1	
piR-rno-38531	14	46522740	46522766	1821.65256	-4.514761468	9.08E-07	AABR07015055.1;LOC257642;AABR07015055.2;AABR07015056.1	
piR-rno-41910	14	46646166	46646196	10030.7755	-3.712281123	7.17E-05	Rn5-8s;AABR07015078.1;AABR07015078.2;AABR07015079.1;AABR07015080.2;LOC257642;AABR07015080.1;AABR07015081.1	
piR-rno-51037	14	46654408	46654438	1466.14141	-4.367761044	3.28E-05	Rn5-8s;AABR07015078.1;AABR07015078.2;AABR07015079.1;AABR07015080.2;LOC257642;AABR07015080.1;AABR07015081.1	
piR-rno-53345	14	46640963	46640992	1106.29914	-4.068787553	8.93E-05	pRNA;Rn5-8s;AABR07015078.1;AABR07015078.2	
piR-rno-55828	14	46683744	46683775	549.82664	-4.721425073	1.84E-06	pRNA;5_8S_rRNA	
piR-rno-56617	14	46643510	46643540	7897.0747	2.765446495	6.46E-05	pRNA;Rn5-8s;AABR07015078.1;AABR07015078.2;AABR07015079.1;AABR07015080.2;LOC257642	
piR-rno-62796	14	46647176	46647201	47.851776	-3.682668026	1.34E-06	Rn5-8s;AABR07015078.1;AABR07015078.2;AABR07015079.1;AABR07015080.2;LOC257642;AABR07015080.1;AABR07015081.1	
piR-rno-62917	14	46644121	46644142	98.2012404	-3.864796061	5.11E-07	Rn5-8s;AABR07015078.1;AABR07015078.2;AABR07015079.1;AABR07015080.2;LOC257642	
piR-rno-62920	14	46641789	46641810	834.501708	-4.94635537	8.64E-08	pRNA;Rn5-8s;AABR07015078.1;AABR07015078.2	
ENSRNOT00000082867.1:ncrna:chromosome:Rnor:6.0:14:	14	2861480	2861612	46.0125396	4.385184217	3.92E-09	Fam69a;Rpl5;SNORA66;SNORD21	Translation
ENSRNOT00000085510.1:ncrna:chromosome:Rnor:6.0:14:	14	2863563	2863695	47.8395171	3.746551881	4.71E-07	Fam69a;Rpl5;SNORA66;SNORD21	Translation
piR-rno-62855	16	68296293	68296315	122.723769	-2.957328559	3.77E-05		
Rattus_norvegicus_chr10.trna11252-GlnCTG:(55454210	17	45633442	45633514	15.015356	5.195550199	1.56E-05	Trim27	Metabolism
Rattus_norvegicus_chr17.trna3680-SerAGA:(58823759-	17	44646969	44647051	83.4445223	-3.651089512	4.43E-05	Zfp184	Transcription
Rattus_norvegicus_chr17.trna3686-SerTGA:(58801093-	17	44669467	44669549	82.3811732	-3.937052321	1.97E-05	AABR07027810.2;AABR07027810.3	
piR-rno-62771	17	44660505	44660535	139.209613	5.792259433	5.19E-08	AABR07027810.2	
piR-rno-42375	18	86602304	86602334	14980.4556	-3.468791494	6.35E-05	Dok6	Signaling
ENSRNOT00000052720.3:ncrna:chromosome:Rnor:6.0:18:	18	27673077	27673196	7.71092785	-5.28599667	1.54E-05	5S_rRNA	
rno-miR-486:MIMAT0037265	NA	NA	NA	12.9645786	-4.410832684	5.50E-06		
piR-rno-40353	NA	NA	NA	749.918353	-3.472959296	7.81E-05		
ENSRNOT00000091903.1:ncrna:scaffold:Rnor:6.0:KL568	NA	NA	NA	43.239007	-5.030684873	6.53E-06		
ENSRNOT00000088091.1:ncrna:scaffold:Rnor:6.0:KL568	NA	NA	NA	7.51120645	-6.289545688	1.80E-05		

ENSRNOT00000069133.1:ncrna:chromosome:Rnor:6.0:MT:	MT	1094	2665	68.9660664	-6.228040247	7.34E-09	AY172581.13;AY172581.9;AY172581.3;AY172581.24;AY172581.14;Mt-nd1;AY172581.4;AY172581.21;AY172581.15;Mt-nd2;AY172581.6;AY172581.22;AY172581.18;AY172581.10;AY172581.7;Mt-co1;AY172581.19;AY172581.12;Mt-co2;AY172581.1;Mt-atp8;Mt-atp6;Mt-cox3;AY172581.5;Mt-nd3;AY172581.16;Mt-nd4l;Mt-nd4;AY172581.23;AY172581.17;AY172581.11;Mt-nd5	
ENSRNOT00000044639.3:ncrna:chromosome:Rnor:6.0:MT:	MT	15348	15416	37.2317314	4.113978479	1.06E-05	Mt-co1;AY172581.19;AY172581.12;Mt-co2;AY172581.1;Mt-atp8;Mt-atp6;Mt-cox3;AY172581.5;Mt-nd3;AY172581.16;Mt-nd4l;Mt-nd4;AY172581.23;AY172581.17;AY172581.11;Mt-nd5;Mt-nd6;AY172581.20;Mt-cyb;AY172581.8;AY1725	
