# Supplementary material for: Alterations in sperm DNA methylation, non-coding RNA and histone retention associate with DDT-induced epigenetic transgenerational inheritance of disease
Source: Epigenetics Chromatin. 2018 Feb 27;11:8. doi: 10.1186/s13072-018-0178-0 (PMC5827984; doi:10.1186/s13072-018-0178-0)
Supplement: Supplementary file 7 — Additional file 7: Table S5. (A) F2 lncRNA p<1e-04, (B) F2 sncRNA p < 1e−04. [file 13072_2018_178_MOESM7_ESM.pdf]

Supplemental Table S5A
F2 lncRNA p<1e-04

Test ID	Gene ID	Gene	Chr	Start	Stop	Log 2.fold Change.	minP	Gene Association	Gene Category
TCONS_00005258	XLOC_001273	AC113925.2	1	168944277	168965566	3.36169	5.00E-05	LOC103694855;LOC103694857;Hbb	Metabolism
TCONS_00015045	XLOC_003734	Hbb	1	168971273	168972725	3.51545	5.00E-05	LOC103694855;Hbb	Metabolism
TCONS_00021679	XLOC_006836	-	1	38595790	38595962	#NAME?	5.00E-05	LOC501406	Transcription
TCONS_00022489	XLOC_007593	-	1	53678139	53678215	Inf	5.00E-05		
TCONS_00025260	XLOC_010310	-	1	130021143	130021254	Inf	5.00E-05		
TCONS_00026817	XLOC_011847	-	1	165236026	165236223	Inf	5.00E-05	Pgm2l1	Metabolism
TCONS_00134530	XLOC_068879	-	2	105272798	105277966	#NAME?	5.00E-05	AABR07009599.2	
TCONS_00136552	XLOC_069343	AABR07072763.1	2	196554035	196568140	#NAME?	5.00E-05	LOC102550012	
TCONS_00143672	XLOC_075023	-	2	110514998	110515197	Inf	5.00E-05		
TCONS_00144408	XLOC_075756	-	2	120815299	120815397	#NAME?	5.00E-05		
TCONS_00146881	XLOC_078100	-	2	181330463	181330588	Inf	5.00E-05	Map9	
TCONS_00146883	XLOC_078102	-	2	181399946	181400103	Inf	5.00E-05		
TCONS_00147408	XLOC_078600	-	2	189747527	189747777	Inf	5.00E-05		
TCONS_00163293	XLOC_086203	-	3	162958903	162959607	Inf	5.00E-05		
TCONS_00185566	XLOC_097642	-	4	88741141	88745766	2.74072	5.00E-05		
TCONS_00192232	XLOC_102232	-	4	99233326	99233402	Inf	5.00E-05	Cd8a	Immune
TCONS_00193138	XLOC_103128	-	4	121156553	121156750	Inf	5.00E-05	5S_rRNA	
TCONS_00206602	XLOC_108730	-	5	13152615	13152691	#NAME?	5.00E-05		
TCONS_00208445	XLOC_110519	-	5	67068714	67068898	Inf	5.00E-05		
TCONS_00217105	XLOC_116418	Serpina5	6	127766469	127772420	#NAME?	5.00E-05	Serpina5;5S_rRNA	EST
TCONS_00221959	XLOC_117828	-	6	5695942	5696017	Inf	5.00E-05		
TCONS_00221991	XLOC_117860	-	6	5754163	5754335	Inf	5.00E-05		
TCONS_00222000	XLOC_117869	-	6	5763630	5763750	Inf	5.00E-05		
TCONS_00222128	XLOC_117997	-	6	6059132	6059336	Inf	5.00E-05	AABR07062751.1	
TCONS_00222967	XLOC_118806	-	6	21163982	21164087	Inf	5.00E-05		
TCONS_00224594	XLOC_120416	-	6	44716478	44716674	Inf	5.00E-05		
TCONS_00226249	XLOC_122014	-	6	72599411	72600446	#NAME?	5.00E-05	Hectd1	Metabolism
TCONS_00226495	XLOC_122240	-	6	80797932	80798008	Inf	5.00E-05		
TCONS_00227884	XLOC_123582	-	6	106398565	106398686	Inf	5.00E-05		
TCONS_00228456	XLOC_124154	-	6	122370020	122370891	#NAME?	5.00E-05		
TCONS_00228457	XLOC_124155	-	6	122400413	122400955	#NAME?	5.00E-05		
TCONS_00228867	XLOC_124537	-	6	132654536	132654618	#NAME?	5.00E-05		
TCONS_00230851	XLOC_125389	Dcn	7	38742050	38782401	2.03528	5.00E-05	Dcn	Cytoskeleton
TCONS_00235832	XLOC_126585	Glipr1l1	7	54828556	54855557	#NAME?	5.00E-05	Glipr1l2;Glipr1l1;Caps2	Development;Signaling
TCONS_00239661	XLOC_127879	-	7	12189339	12190111	2.39285	5.00E-05	Mbd3;Mex3d;AC120291.1	Epigenetic;Transcription
TCONS_00239751	XLOC_127969	-	7	14953125	14953278	Inf	5.00E-05		
TCONS_00241996	XLOC_130205	-	7	66409571	66409647	Inf	5.00E-05		
TCONS_00247267	XLOC_134069	-	8	61869084	61874629	#NAME?	5.00E-05		
TCONS_00252918	XLOC_135226	-	8	76145034	76154895	Inf	5.00E-05		
TCONS_00257177	XLOC_138015	-	8	58807591	58808993	#NAME?	5.00E-05		
TCONS_00259882	XLOC_140601	-	8	114526465	114526545	Inf	5.00E-05	Col6a5	
TCONS_00263315	XLOC_141897	LOC501180	9	93445001	93446420	2.24068	5.00E-05	LOC501180;AC112440.1	
TCONS_00263885	XLOC_141985	-	9	101178292	101179878	Inf	5.00E-05		
TCONS_00268036	XLOC_143400	-	9	13368298	13368584	Inf	5.00E-05		
TCONS_00268578	XLOC_143911	-	9	22054451	22054691	Inf	5.00E-05		
TCONS_00272300	XLOC_147569	-	9	105925158	105925877	#NAME?	5.00E-05		

TCONS_00036168	XLOC_017554	LOC680396	10	87782375	87782936	Inf	5.00E-05	Krtap9-1;LOC680396;LOC100910814;Rn50_10_0876.2	
TCONS_00040605	XLOC_018448	Slc13a5	10	58809971	58834606	1.99536	5.00E-05	Slc13a5	Transport
TCONS_00046610	XLOC_021602	-	10	51823238	51823336	Inf	5.00E-05		
TCONS_00054932	XLOC_026437	-	11	18920987	18921701	#NAME?	5.00E-05	SNORA17	
TCONS_00055280	XLOC_026785	-	11	29106643	29106975	Inf	5.00E-05		
TCONS_00061525	XLOC_029940	Glt1d1	12	32666546	32740732	2.04942	5.00E-05	Glt1d1	
TCONS_00064430	XLOC_031835	-	12	43648840	43648945	Inf	5.00E-05	AABR07036452.1	
TCONS_00067364	XLOC_032835	Gli2	13	34829138	35048444	2.32349	5.00E-05	Gli2	Transcription
TCONS_00068848	XLOC_033156	-	13	83220860	83227817	#NAME?	5.00E-05		
TCONS_00070488	XLOC_033872	-	13	34230337	34230592	#NAME?	5.00E-05	Tsn	Transcription
TCONS_00070540	XLOC_033921	-	13	35549784	35549860	Inf	5.00E-05	Tmem185b;U6	Unknown
TCONS_00072456	XLOC_035779	-	13	86182244	86182499	Inf	5.00E-05		
TCONS_00072681	XLOC_035982	-	13	92836966	92837090	Inf	5.00E-05		
TCONS_00080224	XLOC_038981	-	14	14260342	14260421	Inf	5.00E-05		
TCONS_00080575	XLOC_039328	-	14	26601930	26602006	Inf	5.00E-05		
TCONS_00082526	XLOC_041206	-	14	84918210	84918354	Inf	5.00E-05	Ascc2	
TCONS_00089901	XLOC_044605	-	15	11271517	11271673	Inf	5.00E-05		
TCONS_00092502	XLOC_047042	-	15	64915167	64915242	Inf	5.00E-05		
TCONS_00097541	XLOC_049528	AABR07024932.1	16	21946564	22118486	2.77994	5.00E-05	RGD1563748	
TCONS_00100493	XLOC_051501	-	16	40705137	40705213	#NAME?	5.00E-05		
TCONS_00101178	XLOC_052153	-	16	58055779	58060323	2.08981	5.00E-05		
TCONS_00101450	XLOC_052423	-	16	65007052	65007203	Inf	5.00E-05		
TCONS_00102030	XLOC_053000	-	16	86994203	86994279	Inf	5.00E-05		
TCONS_00110037	XLOC_056988	-	17	46797659	46797889	Inf	5.00E-05	Elmo1	Signaling
TCONS_00110833	XLOC_057741	-	17	58074813	58074981	Inf	5.00E-05		
TCONS_00119538	XLOC_063150	-	18	80151991	80152215	#NAME?	5.00E-05		
TCONS_00124438	XLOC_064464	-	19	5077825	5077974	Inf	5.00E-05		
TCONS_00125711	XLOC_065720	-	19	33566266	33566340	Inf	5.00E-05		
TCONS_00275003	XLOC_148979	AABR07040622.1	X	106518322	106519205	2.17432	5.00E-05	AABR07040622.1;MGC109340	Signaling
TCONS_00276681	XLOC_149504	LOC680489	X	44999724	45000363	#NAME?	5.00E-05	Cldn34e	
TCONS_00277446	XLOC_149770	RGD1562485	X	96458909	96460226	2.52747	5.00E-05	RGD1562485;SNORA17;AABR07040252.1	
TCONS_00279913	XLOC_151496	-	X	32910545	32911221	Inf	5.00E-05		
TCONS_00280665	XLOC_152248	-	X	48808493	48808842	Inf	5.00E-05	Tmem47	Unknown
TCONS_00282456	XLOC_154006	-	X	85487953	85488043	#NAME?	5.00E-05	Dach2	Development
TCONS_00283756	XLOC_155229	-	X	115189201	115189333	Inf	5.00E-05		

Supplemental Table S5B
F2 sncRNA p<1e-04

Identification	Chr	Start	Stop	baseMean	log2FoldChange	minP	Gene Association	Gene Category
Rattus_norvegicus_chr1.trna4916-ProTGG:(169977022-	1	163773949	163774021	14.6924063	4.77215699	5.28E-05		
ENSRNOT00000077255.1:ncrna:chromosome:Rnor:6.0:1:2	1	22759629	22759704	20.8212201	-4.864668	4.80E-05	LOC100910237;Rps12;SNORD101;SNORD100;SNORA33	Translation
ENSRNOT00000090106.1:ncrna:chromosome:Rnor:6.0:2:2	2	251829017	251829133	5.44107328	-5.7589804	8.75E-05	RGD1560065;U5	
piR-rno-62733	4	77804825	77804849	73.1346609	3.40577843	5.25E-05		
piR-rno-62747	8	108122616	108122635	737.568166	4.30567507	1.43E-06		
Rattus_norvegicus_chr10.trna5785-ArgCCG:(94966639-	10	95225812	95225885	430.832856	4.30702751	4.57E-07		
piR-rno-62743	10	55620718	55620745	278.389308	3.80536097	2.98E-05	Ctc1;AC129753.2;Aurkb	Signaling
Rattus_norvegicus_chr13.trna2243-AspGTC:(94071463-	13	91233137	91233209	223.393172	2.66625926	6.29E-05	LOC100911825	Receptor
piR-rno-51037	14	46654408	46654438	1466.14141	5.7397443	5.05E-08	Rn5-8s;AABR07015078.1;AABR07015078.2;AABR07015079.1;AABR07015080.2;LOC257642;AABR07015080.1;AABR07015081.1	
piR-rno-62814	15	58226903	58226930	350.312654	3.81071001	3.66E-07		
piR-rno-62858	15	55460942	55460969	263.018871	4.50220248	4.88E-09	Nudt15;Sucla2	Metabolism
ENSRNOT00000045072.3:ncrna:chromosome:Rnor:6.0:MT:	MT	1026	1094	211.76817	-3.7652673	2.42E-05	AY172581.13;AY172581.9;AY172581.3;AY172581.24;AY172581.14;Mt-nd1;AY172581.4;AY172581.21;AY172581.15;Mt-nd2;AY172581.6;AY172581.22;AY172581.18;AY172581.10;AY172581.7;Mt-co1;AY172581.19;AY172581.12;Mt-co2;AY172581.1;Mt-atp8;Mt-atp6;Mt-cox3;AY172581.5;Mt-nd3;AY172581.16;Mt-nd4l;Mt-nd4	
ENSRNOT00000048050.3:ncrna:chromosome:Rnor:6.0:MT:	MT	9383	9451	24.3501741	-3.9150241	4.14E-05	AY172581.13;AY172581.9;AY172581.3;AY172581.24;AY172581.14;Mt-nd1;AY172581.4;AY172581.21;AY172581.15;Mt-nd2;AY172581.6;AY172581.22;AY172581.18;AY172581.10;AY172581.7;Mt-co1;AY172581.19;AY172581.12;Mt-co2;AY172581.1;Mt-atp8;Mt-atp6;Mt-cox3;AY172581.5;Mt-nd3;AY172581.16;Mt-nd4l;Mt-nd4;AY172581.23;AY172581.17;AY172581.11;Mt-nd5;Mt-nd6;AY172581.20;Mt-cyb;AY172581.8;A	
