# Supplementary material for: Alterations in sperm DNA methylation, non-coding RNA and histone retention associate with DDT-induced epigenetic transgenerational inheritance of disease
Source: Epigenetics Chromatin. 2018 Feb 27;11:8. doi: 10.1186/s13072-018-0178-0 (PMC5827984; doi:10.1186/s13072-018-0178-0)
Supplement: Supplementary file 8 — Additional file 8: Table S6. (A) F3 lncRNA p<1e-04, (B) F3 sncRNA p < 1e−04. [file 13072_2018_178_MOESM8_ESM.pdf]

Supplemental Table S6A
F3 lncRNA p<1e-04

Test ID	Gene ID	Chr	Start	Stop	log2.fold_change.	minP	q_value	Gene Association	Gene Category
TCONS_00000137	XLOC_000037	1	8255761	8266313	4.48738	5.00E-05	0.012978		
TCONS_00002807	XLOC_000658	1	86802912	86813799	-7.03269	5.00E-05	0.012978	AABR07002845.1	
TCONS_00003736	XLOC_000899	1	105263704	105266587	-4.63614	5.00E-05	0.012978		
TCONS_00004292	XLOC_001048	1	141218094	141218277	Inf	5.00E-05	0.012978	AABR07004397.2;AABR07004397.1;LOC691427	
TCONS_00006188	XLOC_001473	1	196737626	196740027	-4.7715	5.00E-05	0.012978	LOC100271845	
TCONS_00008350	XLOC_001972	1	235889686	235895399	#NAME?	5.00E-05	0.012978		
TCONS_00009010	XLOC_002145	1	261229193	261280696	-4.13711	5.00E-05	0.012978	Mms19;U6;Ubtd1;Ankrd2	Transcription;Metabolism
TCONS_00010027	XLOC_002368	1	11693564	11700441	Inf	5.00E-05	0.012978		
TCONS_00011006	XLOC_002649	1	53663366	53666378	-6.33465	5.00E-05	0.012978		
TCONS_00012132	XLOC_002937	1	81291745	81295442	-5.24525	5.00E-05	0.012978	Smg9;Irgc	
TCONS_00012200	XLOC_002961	1	82247674	82266727	-4.80861	5.00E-05	0.012978	Lipe;AC121639.1;Cxcl17	Metabolism;Growth Factors & Cytokines
TCONS_00013475	XLOC_003287	1	111413394	111415685	#NAME?	5.00E-05	0.012978		
TCONS_00015045	XLOC_003734	1	168971273	168972725	-5.45735	5.00E-05	0.012978	LOC103694855;Hbb	Metabolism
TCONS_00015059	XLOC_003748	1	169319199	169321075	-5.41221	5.00E-05	0.012978	RGD1562433	
TCONS_00017734	XLOC_004339	1	223549178	223549904	-3.19061	5.00E-05	0.012978	AABR07006160.1	
TCONS_00017791	XLOC_004358	1	225308640	225330126	-4.84909	5.00E-05	0.012978	Asrgl1	Metabolism
TCONS_00019281	XLOC_004733	1	273619088	273647175	#NAME?	5.00E-05	0.012978	AC129049.1	
TCONS_00019347	XLOC_004743	1	275698913	275714179	2.91487	5.00E-05	0.012978		
TCONS_00019665	XLOC_004847	1	2218883	2219118	#NAME?	5.00E-05	0.012978		
TCONS_00020180	XLOC_005362	1	5880054	5880328	#NAME?	5.00E-05	0.012978		
TCONS_00020268	XLOC_005450	1	7596927	7597563	Inf	5.00E-05	0.012978		
TCONS_00020623	XLOC_005800	1	12774667	12774932	#NAME?	5.00E-05	0.012978	AC128394.3	
TCONS_00020808	XLOC_005974	1	14649777	14650117	#NAME?	5.00E-05	0.012978		
TCONS_00020996	XLOC_006162	1	20755754	20755846	#NAME?	5.00E-05	0.012978		
TCONS_00021370	XLOC_006531	1	29629803	29630462	#NAME?	5.00E-05	0.012978		
TCONS_00021387	XLOC_006548	1	30834076	30834575	#NAME?	5.00E-05	0.012978		
TCONS_00021432	XLOC_006591	1	32782647	32782861	#NAME?	5.00E-05	0.012978		
TCONS_00021614	XLOC_006771	1	37170078	37170624	#NAME?	5.00E-05	0.012978	AABR07001068.1	
TCONS_00021645	XLOC_006802	1	37208221	37208495	#NAME?	5.00E-05	0.012978		
TCONS_00021665	XLOC_006822	1	38350822	38350898	#NAME?	5.00E-05	0.012978		
TCONS_00021707	XLOC_006864	1	38700699	38700774	#NAME?	5.00E-05	0.012978		
TCONS_00021724	XLOC_006881	1	38803702	38803936	#NAME?	5.00E-05	0.012978		
TCONS_00022053	XLOC_007199	1	45791931	45792497	Inf	5.00E-05	0.012978		

TCONS_00022443	XLOC_007547	1	51831409	51831514	#NAME?	5.00E-05	0.012978	AABR07001561.1	
TCONS_00022550	XLOC_007650	1	54322897	54322991	#NAME?	5.00E-05	0.012978	AABR07001623.1	
TCONS_00022748	XLOC_007847	1	59814458	59814534	#NAME?	5.00E-05	0.012978		
TCONS_00022916	XLOC_007997	1	62388062	62389760	Inf	5.00E-05	0.012978		
TCONS_00022932	XLOC_008013	1	62402860	62403669	Inf	5.00E-05	0.012978		
TCONS_00023528	XLOC_008599	1	80370438	80370680	#NAME?	5.00E-05	0.012978	Mark4;U1;AABR07002677.2	Signaling
TCONS_00023571	XLOC_008642	1	81364184	81365018	Inf	5.00E-05	0.012978	Cadm4;Zfp428	Receptor
TCONS_00023578	XLOC_008649	1	81827213	81827454	#NAME?	5.00E-05	0.012978		
TCONS_00023646	XLOC_008717	1	83835381	83835483	#NAME?	5.00E-05	0.012978		
TCONS_00023663	XLOC_008734	1	84303419	84303494	#NAME?	5.00E-05	0.012978	Sertad1;Prx	Transcription
TCONS_00024135	XLOC_009195	1	95174944	95175070	#NAME?	5.00E-05	0.012978		
TCONS_00024555	XLOC_009612	1	112824518	112824690	#NAME?	5.00E-05	0.012978	Luzp2;Gabra5	Receptor
TCONS_00024658	XLOC_009714	1	115713482	115714348	#NAME?	5.00E-05	0.012978		
TCONS_00024730	XLOC_009781	1	118366941	118367047	#NAME?	5.00E-05	0.012978		
TCONS_00024809	XLOC_009860	1	119699214	119699291	#NAME?	5.00E-05	0.012978		
TCONS_00024811	XLOC_009862	1	119715361	119715436	#NAME?	5.00E-05	0.012978		
TCONS_00024819	XLOC_009870	1	120350837	120351140	#NAME?	5.00E-05	0.012978		
TCONS_00024839	XLOC_009890	1	121346885	121346962	#NAME?	5.00E-05	0.012978		
TCONS_00025389	XLOC_010439	1	131183983	131184434	#NAME?	5.00E-05	0.012978		
TCONS_00025673	XLOC_010710	1	138519875	138520105	#NAME?	5.00E-05	0.012978	Agbl1	Signaling
TCONS_00025840	XLOC_010877	1	141970145	141970265	#NAME?	5.00E-05	0.012978	LOC102548889	
TCONS_00025910	XLOC_010947	1	143946052	143946126	#NAME?	5.00E-05	0.012978		
TCONS_00026003	XLOC_011040	1	146625443	146626393	Inf	5.00E-05	0.012978		
TCONS_00026053	XLOC_011084	1	146881513	146882630	Inf	5.00E-05	0.012978		
TCONS_00026054	XLOC_011085	1	146883291	146884207	Inf	5.00E-05	0.012978		
TCONS_00026073	XLOC_011104	1	148909170	148909323	#NAME?	5.00E-05	0.012978		
TCONS_00026423	XLOC_011454	1	160030564	160031660	Inf	5.00E-05	0.012978		
TCONS_00026582	XLOC_011613	1	160467473	160467750	#NAME?	5.00E-05	0.012978		
TCONS_00026636	XLOC_011667	1	161890785	161890873	#NAME?	5.00E-05	0.012978	Tenm4;U6	Signaling
TCONS_00026714	XLOC_011744	1	163618397	163619911	Inf	5.00E-05	0.012978	Emsy	
TCONS_00026854	XLOC_011884	1	166422600	166422846	#NAME?	5.00E-05	0.012978	Atg16l2;Stard10	Unknown;Golgi
TCONS_00027135	XLOC_012165	1	173301431	173301506	#NAME?	5.00E-05	0.012978		
TCONS_00027227	XLOC_012256	1	175983782	175983909	#NAME?	5.00E-05	0.012978		
TCONS_00027417	XLOC_012446	1	185199094	185199198	#NAME?	5.00E-05	0.012978		
TCONS_00027455	XLOC_012484	1	186616222	186616298	#NAME?	5.00E-05	0.012978		
TCONS_00027791	XLOC_012808	1	196815544	196815620	#NAME?	5.00E-05	0.012978		
TCONS_00028353	XLOC_013359	1	210951734	210951987	#NAME?	5.00E-05	0.012978		
TCONS_00028511	XLOC_013516	1	214167689	214167813	#NAME?	5.00E-05	0.012978	Rnh1;U6	Translation
TCONS_00028571	XLOC_013576	1	215905085	215906087	-4.44004	5.00E-05	0.012978	AC099449.1	
TCONS_00028606	XLOC_013610	1	216633781	216633855	#NAME?	5.00E-05	0.012978	Kcnq1	Transport
TCONS_00029062	XLOC_014056	1	226495651	226496514	Inf	5.00E-05	0.012978	Syt7;Lrrc10b	Unknown
TCONS_00029196	XLOC_014189	1	230836515	230836653	#NAME?	5.00E-05	0.012978		
TCONS_00029199	XLOC_014192	1	231064012	231064098	#NAME?	5.00E-05	0.012978		

TCONS_00029205	XLOC_014198	1	232385104	232385180	#NAME?	5.00E-05	0.012978		
TCONS_00029272	XLOC_014265	1	234000327	234000417	#NAME?	5.00E-05	0.012978		
TCONS_00029273	XLOC_014266	1	234224315	234224424	#NAME?	5.00E-05	0.012978		
TCONS_00029321	XLOC_014314	1	236476892	236476967	#NAME?	5.00E-05	0.012978		
TCONS_00030130	XLOC_015106	1	249605837	249607199	Inf	5.00E-05	0.012978		
TCONS_00030181	XLOC_015157	1	249675333	249676452	Inf	5.00E-05	0.012978		
TCONS_00030279	XLOC_015254	1	249949736	249950766	Inf	5.00E-05	0.012978		
TCONS_00030512	XLOC_015469	1	254737726	254737853	#NAME?	5.00E-05	0.012978	Ankrd1	
TCONS_00030678	XLOC_015628	1	261387508	261388395	Inf	5.00E-05	0.012978	Marveld1	
TCONS_00031071	XLOC_016015	1	273648302	273648378	#NAME?	5.00E-05	0.012978		
TCONS_00031206	XLOC_016148	1	275792997	275793891	Inf	5.00E-05	0.012978		
TCONS_00031288	XLOC_016230	1	276755898	276755975	#NAME?	5.00E-05	0.012978		
TCONS_00031395	XLOC_016323	1	278913789	278914073	Inf	5.00E-05	0.012978		
TCONS_00031416	XLOC_016344	1	279061938	279062266	Inf	5.00E-05	0.012978		
TCONS_00031458	XLOC_016384	1	280078135	280079213	Inf	5.00E-05	0.012978	AABR07007086.1	
TCONS_00031585	XLOC_016511	1	281417742	281417819	#NAME?	5.00E-05	0.012978		
TCONS_00031859	XLOC_016602	10	4952567	4954791	-3.6833	5.00E-05	0.012978	Prm1;Prm2;Prm3;Tnp2;Socs1	Transcription;Signaling
TCONS_00032707	XLOC_016796	10	22992003	22992471	#NAME?	5.00E-05	0.012978		
TCONS_00034637	XLOC_017270	10	64360382	64378789	-4.5551	5.00E-05	0.012978	Fam57a;Gemin4;Dbil5;Glod4	Signaling;Metabolism
TCONS_00035783	XLOC_017482	10	83600371	83655182	-4.91316	5.00E-05	0.012978	AABR07030386.2;Phospho1;Abi3;AABR07030386.1;Gngt2	Signaling
TCONS_00036190	XLOC_017566	10	88339906	88372900	-5.67601	5.00E-05	0.012978	Fkbp10;Nt5c3b;Klhl10;Klhl11	Signaling;Metabolism;Cytoskeleton;Transcription
TCONS_00044036	XLOC_019179	10	344326	344816	#NAME?	5.00E-05	0.012978		
TCONS_00044162	XLOC_019298	10	2176630	2177022	#NAME?	5.00E-05	0.012978		
TCONS_00044630	XLOC_019711	10	11762850	11763724	Inf	5.00E-05	0.012978	Trap1;Dnase1	Protein Binding;Transcription
TCONS_00045051	XLOC_020125	10	18030967	18031042	#NAME?	5.00E-05	0.012978		
TCONS_00045539	XLOC_020544	10	27710537	27710613	#NAME?	5.00E-05	0.012978		
TCONS_00046100	XLOC_021094	10	35868837	35869387	#NAME?	5.00E-05	0.012978	Cby3;Hnrnph1	Transcription
TCONS_00046174	XLOC_021168	10	37652386	37653471	Inf	5.00E-05	0.012978	Tcf7	
TCONS_00046197	XLOC_021191	10	39107725	39109010	Inf	5.00E-05	0.012978	AC135771.1;Irf1	Immune
TCONS_00046319	XLOC_021313	10	43432123	43432950	Inf	5.00E-05	0.012978		
TCONS_00046529	XLOC_021522	10	49118902	49119167	#NAME?	5.00E-05	0.012978		
TCONS_00046687	XLOC_021679	10	54055880	54056519	Inf	5.00E-05	0.012978		
TCONS_00047064	XLOC_022053	10	63607272	63607992	Inf	5.00E-05	0.012978		

TCONS_00047539	XLOC_022493	10	74073352	74074024	Inf	5.00E-05	0.012978	Cltc;Dhx40	Cytoskeleton;Transcription
TCONS_00047770	XLOC_022722	10	78303597	78303855	#NAME?	5.00E-05	0.012978		
TCONS_00047964	XLOC_022912	10	83764076	83764353	#NAME?	5.00E-05	0.012978		
TCONS_00048267	XLOC_023212	10	89728769	89728846	#NAME?	5.00E-05	0.012978		
TCONS_00048332	XLOC_023277	10	91514326	91514860	#NAME?	5.00E-05	0.012978		
TCONS_00048724	XLOC_023641	10	97454290	97454366	#NAME?	5.00E-05	0.012978		
TCONS_00048726	XLOC_023643	10	97473374	97473570	#NAME?	5.00E-05	0.012978		
TCONS_00049203	XLOC_024108	10	106591838	106591912	#NAME?	5.00E-05	0.012978		
TCONS_00049674	XLOC_024579	10	112584266	112584938	Inf	5.00E-05	0.012978		
TCONS_00049680	XLOC_024585	10	112596479	112597042	Inf	5.00E-05	0.012978		
TCONS_00051376	XLOC_025066	11	83782458	83785497	Inf	5.00E-05	0.012978		
TCONS_00054142	XLOC_025719	11	1589057	1589454	#NAME?	5.00E-05	0.012978		
TCONS_00054223	XLOC_025779	11	2323478	2323561	#NAME?	5.00E-05	0.012978		
TCONS_00054463	XLOC_026019	11	10377487	10377563	#NAME?	5.00E-05	0.012978		
TCONS_00054936	XLOC_026441	11	18923635	18924594	#NAME?	5.00E-05	0.012978	SNORA17	
TCONS_00055115	XLOC_026620	11	24030672	24030747	#NAME?	5.00E-05	0.012978	AABR07033492.1	
TCONS_00055288	XLOC_026793	11	29113433	29113599	#NAME?	5.00E-05	0.012978		
TCONS_00055872	XLOC_027351	11	40630680	40631261	#NAME?	5.00E-05	0.012978		
TCONS_00055935	XLOC_027414	11	43685748	43686769	Inf	5.00E-05	0.012978	Olr1558	
TCONS_00056192	XLOC_027645	11	52295874	52296280	#NAME?	5.00E-05	0.012978		
TCONS_00056284	XLOC_027719	11	56205380	56205556	#NAME?	5.00E-05	0.012978		
TCONS_00056417	XLOC_027852	11	59371487	59371645	#NAME?	5.00E-05	0.012978		
TCONS_00056441	XLOC_027876	11	59955931	59956033	#NAME?	5.00E-05	0.012978		
TCONS_00056497	XLOC_027932	11	60940410	60941015	Inf	5.00E-05	0.012978	Nepro	
TCONS_00056609	XLOC_028044	11	65929983	65930178	#NAME?	5.00E-05	0.012978		
TCONS_00056642	XLOC_028077	11	67168728	67168875	#NAME?	5.00E-05	0.012978		
TCONS_00056667	XLOC_028102	11	68074095	68074807	Inf	5.00E-05	0.012978	AABR07034393.1	
TCONS_00057320	XLOC_028727	11	83631259	83631344	#NAME?	5.00E-05	0.012978		
TCONS_00057461	XLOC_028866	11	85479356	85483471	-5.81205	5.00E-05	0.012978	AABR07034730.1	
TCONS_00057497	XLOC_028902	11	86450769	86451048	#NAME?	5.00E-05	0.012978		
TCONS_00057710	XLOC_029108	11	90213530	90213606	#NAME?	5.00E-05	0.012978	Efcab1	Receptor
TCONS_00057948	XLOC_029170	12	6544196	6552286	#NAME?	5.00E-05	0.012978	AABR07035190.1	
TCONS_00059704	XLOC_029531	12	45462878	45466566	-4.40966	5.00E-05	0.012978		
TCONS_00060750	XLOC_029745	12	13043040	13067043	-4.61201	5.00E-05	0.012978	Fam220a	
TCONS_00061380	XLOC_029906	12	27976869	28008200	3.11743	5.00E-05	0.012978		
TCONS_00061382	XLOC_029908	12	28098092	28125059	4.15192	5.00E-05	0.012978	5S_rRNA	
TCONS_00061423	XLOC_029921	12	30300888	30304110	-4.23265	5.00E-05	0.012978	Nupr2;Zbed5	
TCONS_00061525	XLOC_029940	12	32666546	32740732	-5.50877	5.00E-05	0.012978	Glt1d1	
TCONS_00062376	XLOC_030106	12	47946608	47990264	-3.23523	5.00E-05	0.012978	Ube3b;Kctd10	Metabolism
TCONS_00062834	XLOC_030360	12	3397684	3397973	#NAME?	5.00E-05	0.012978		
TCONS_00062927	XLOC_030453	12	6134549	6134691	#NAME?	5.00E-05	0.012978		
TCONS_00062954	XLOC_030480	12	6530048	6536418	-4.58096	5.00E-05	0.012978	AABR07035190.1	
TCONS_00063056	XLOC_030582	12	8011466	8011842	Inf	5.00E-05	0.012978		
TCONS_00063248	XLOC_030771	12	13508319	13508395	#NAME?	5.00E-05	0.012978	Rnf216	Metabolism

TCONS_00063303	XLOC_030826	12	15127409	15128246	Inf	5.00E-05	0.012978		
TCONS_00063306	XLOC_030829	12	15135024	15135797	Inf	5.00E-05	0.012978		
TCONS_00063381	XLOC_030904	12	15540947	15542103	Inf	5.00E-05	0.012978		
TCONS_00063628	XLOC_031150	12	21221744	21222570	#NAME?	5.00E-05	0.012978		
TCONS_00064185	XLOC_031591	12	38695406	38696228	Inf	5.00E-05	0.012978	Bcl7a	
TCONS_00064186	XLOC_031592	12	38696600	38697417	Inf	5.00E-05	0.012978	Bcl7a	
TCONS_00064232	XLOC_031637	12	39525471	39526639	Inf	5.00E-05	0.012978		
TCONS_00064280	XLOC_031685	12	41370911	41371806	Inf	5.00E-05	0.012978	Oas2	Transcription
TCONS_00064558	XLOC_031962	12	47867546	47867622	#NAME?	5.00E-05	0.012978		
TCONS_00064856	XLOC_032216	13	26172242	26394505	-3.20585	5.00E-05	0.012978	Phlpp1	Signaling
TCONS_00065526	XLOC_032362	13	53506924	53538136	-4.53326	5.00E-05	0.012978	Zfp281	Transcription
TCONS_00065866	XLOC_032450	13	73118024	73119305	#NAME?	5.00E-05	0.012978		
TCONS_00068134	XLOC_032988	13	64868731	64954679	#NAME?	5.00E-05	0.012978		
TCONS_00070121	XLOC_033505	13	3697350	3697426	#NAME?	5.00E-05	0.012978		
TCONS_00070181	XLOC_033565	13	13659483	13659922	#NAME?	5.00E-05	0.012978		
TCONS_00070190	XLOC_033574	13	16179155	16179270	#NAME?	5.00E-05	0.012978		
TCONS_00070194	XLOC_033578	13	17323177	17323253	#NAME?	5.00E-05	0.012978		
TCONS_00070249	XLOC_033633	13	19844055	19844130	#NAME?	5.00E-05	0.012978		
TCONS_00070260	XLOC_033644	13	21027649	21027895	Inf	5.00E-05	0.012978		
TCONS_00070271	XLOC_033655	13	23787616	23788022	#NAME?	5.00E-05	0.012978		
TCONS_00070339	XLOC_033723	13	26025353	26025467	#NAME?	5.00E-05	0.012978		
TCONS_00070451	XLOC_033835	13	31462312	31462426	#NAME?	5.00E-05	0.012978		
TCONS_00070452	XLOC_033836	13	31496751	31496921	#NAME?	5.00E-05	0.012978		
TCONS_00070602	XLOC_033983	13	37670032	37670454	#NAME?	5.00E-05	0.012978		
TCONS_00070667	XLOC_034048	13	39061021	39061612	Inf	5.00E-05	0.012978		
TCONS_00070955	XLOC_034286	13	50656339	50656437	#NAME?	5.00E-05	0.012978		
TCONS_00071026	XLOC_034357	13	51461436	51462213	Inf	5.00E-05	0.012978	Kdm5b	Transcription
TCONS_00071152	XLOC_034479	13	53627859	53628363	#NAME?	5.00E-05	0.012978		
TCONS_00071257	XLOC_034584	13	57724824	57726542	-5.50154	5.00E-05	0.012978		
TCONS_00071265	XLOC_034592	13	58597202	58597597	#NAME?	5.00E-05	0.012978		
TCONS_00071368	XLOC_034695	13	59969290	59969365	#NAME?	5.00E-05	0.012978		
TCONS_00072229	XLOC_035552	13	80412703	80412792	#NAME?	5.00E-05	0.012978	Mettl13	
TCONS_00072409	XLOC_035732	13	83845327	83846558	Inf	5.00E-05	0.012978	Mpzl1;Rcsd1	Development;Unknown
TCONS_00072553	XLOC_035854	13	87623353	87623429	#NAME?	5.00E-05	0.012978		
TCONS_00072554	XLOC_035855	13	87771616	87771693	#NAME?	5.00E-05	0.012978		
TCONS_00072590	XLOC_035891	13	90026034	90026455	#NAME?	5.00E-05	0.012978	Ly9	Receptor
TCONS_00072942	XLOC_036229	13	99560936	99561932	Inf	5.00E-05	0.012978		
TCONS_00073064	XLOC_036351	13	101776611	101777294	Inf	5.00E-05	0.012978		
TCONS_00073170	XLOC_036457	13	105427096	105427171	#NAME?	5.00E-05	0.012978		
TCONS_00073343	XLOC_036624	13	108223390	108223466	#NAME?	5.00E-05	0.012978		
TCONS_00073398	XLOC_036679	13	109922749	109924548	Inf	5.00E-05	0.012978		
TCONS_00073757	XLOC_036976	14	2380753	2393083	-3.68849	5.00E-05	0.012978	Pde6b	Metabolism
TCONS_00076445	XLOC_037567	14	107784505	107798256	-4.75831	5.00E-05	0.012978	Cct4;Fam161a	Protein Binding
TCONS_00077305	XLOC_037765	14	17969218	18076907	4.67968	5.00E-05	0.012978	Parm1	Apoptosis
TCONS_00077405	XLOC_037807	14	21746294	21748364	-4.54257	5.00E-05	0.012978	Cabs1;2310003L06Rik	

TCONS_00078118	XLOC_037995	14	60958591	60964324	6.32576	5.00E-05	0.012978	Ccdc149;Sod3	Metabolism
TCONS_00079176	XLOC_038215	14	98982878	99031306	#NAME?	5.00E-05	0.012978		
TCONS_00079178	XLOC_038217	14	99468414	99481651	#NAME?	5.00E-05	0.012978		
TCONS_00079564	XLOC_038360	14	2176723	2177312	Inf	5.00E-05	0.012978	Gak	Cell Cycle
TCONS_00079931	XLOC_038694	14	8618453	8619532	Inf	5.00E-05	0.012978		
TCONS_00080432	XLOC_039189	14	18876999	18877084	#NAME?	5.00E-05	0.012978		
TCONS_00080444	XLOC_039201	14	19631951	19632026	#NAME?	5.00E-05	0.012978		
TCONS_00080584	XLOC_039337	14	26960045	26960171	#NAME?	5.00E-05	0.012978		
TCONS_00080955	XLOC_039700	14	37109188	37109583	#NAME?	5.00E-05	0.012978	Spata18;Sgcb	Cytoskeleton
TCONS_00081544	XLOC_040228	14	51498362	51498517	#NAME?	5.00E-05	0.012978		
TCONS_00081845	XLOC_040526	14	63015718	63015960	#NAME?	5.00E-05	0.012978		
TCONS_00081852	XLOC_040533	14	64595610	64595690	#NAME?	5.00E-05	0.012978		
TCONS_00081960	XLOC_040641	14	68523018	68523093	#NAME?	5.00E-05	0.012978		
TCONS_00082259	XLOC_040939	14	76195486	76195562	#NAME?	5.00E-05	0.012978		
TCONS_00082342	XLOC_041022	14	78517700	78518137	#NAME?	5.00E-05	0.012978		
TCONS_00082470	XLOC_041150	14	82697772	82698331	#NAME?	5.00E-05	0.012978	Maea	Receptor
TCONS_00082477	XLOC_041157	14	82805772	82806434	Inf	5.00E-05	0.012978		
TCONS_00083138	XLOC_041808	14	102319222	102319399	#NAME?	5.00E-05	0.012978		
TCONS_00083756	XLOC_042370	15	2245068	2294349	3.22248	5.00E-05	0.012978		
TCONS_00085941	XLOC_042970	15	76324814	76370492	#NAME?	5.00E-05	0.012978		
TCONS_00088223	XLOC_043543	15	52337527	52344629	#NAME?	5.00E-05	0.012978	Fgf17;Npm2	Signaling;Translation
TCONS_00088905	XLOC_043740	15	104117945	104168638	-4.46418	5.00E-05	0.012978	Cldn10;Dzip1	Cell Junction;Transcription
TCONS_00089106	XLOC_043866	15	1387305	1387594	#NAME?	5.00E-05	0.012978		
TCONS_00089265	XLOC_043997	15	1874635	1875294	Inf	5.00E-05	0.012978		
TCONS_00089271	XLOC_044003	15	1891425	1892451	Inf	5.00E-05	0.012978		
TCONS_00089472	XLOC_044193	15	7734946	7735504	Inf	5.00E-05	0.012978		
TCONS_00089548	XLOC_044259	15	7837186	7837740	Inf	5.00E-05	0.012978		
TCONS_00089682	XLOC_044391	15	9685828	9709502	-5.14578	5.00E-05	0.012978		
TCONS_00089824	XLOC_044528	15	10321452	10323949	Inf	5.00E-05	0.012978	Top2b	Transcription
TCONS_00089840	XLOC_044544	15	10609261	10609561	#NAME?	5.00E-05	0.012978		
TCONS_00089860	XLOC_044564	15	11105714	11107904	Inf	5.00E-05	0.012978		
TCONS_00089902	XLOC_044606	15	11296089	11296170	#NAME?	5.00E-05	0.012978	Lrrc3b	Receptor
TCONS_00090145	XLOC_044760	15	13795365	13795441	#NAME?	5.00E-05	0.012978		
TCONS_00090281	XLOC_044896	15	14897133	14898669	Inf	5.00E-05	0.012978		
TCONS_00090416	XLOC_045008	15	16579560	16580329	Inf	5.00E-05	0.012978		
TCONS_00090548	XLOC_045116	15	18268250	18268326	#NAME?	5.00E-05	0.012978		
TCONS_00090570	XLOC_045138	15	18390756	18391870	Inf	5.00E-05	0.012978	Fam107a	
TCONS_00090711	XLOC_045279	15	22764688	22764785	#NAME?	5.00E-05	0.012978		
TCONS_00090743	XLOC_045309	15	24231329	24231408	#NAME?	5.00E-05	0.012978		
TCONS_00090967	XLOC_045533	15	28144145	28144940	Inf	5.00E-05	0.012978	Rnase2	
TCONS_00090977	XLOC_045543	15	28458995	28459111	#NAME?	5.00E-05	0.012978		

TCONS_00091007	XLOC_045573	15	30623453	30870196	Inf	5.00E-05	0.012978	AABR07017748.2;AABR07017763.2;AABR07017763.1;AABR07017765.1;AABR07017768.5;AABR07017768.6;AABR07017768.3;AABR07017768.1;AABR07017768.7;AABR07017768.2;LOC100911282;AABR07017768.4;AABR07017769.1;AABR07017770.1	
TCONS_00091027	XLOC_045593	15	32026746	32027268	Inf	5.00E-05	0.012978	AABR07017868.3	
TCONS_00091340	XLOC_045898	15	41694943	41696025	Inf	5.00E-05	0.012978	Ebpl;Kpna3	Metabolism;Binding Protein
TCONS_00091356	XLOC_045914	15	41951747	41952509	Inf	5.00E-05	0.012978	Kcnrg;AABR07018128.1	Signaling
TCONS_00091441	XLOC_045999	15	45299574	45299683	#NAME?	5.00E-05	0.012978		
TCONS_00091478	XLOC_046035	15	45991245	45991360	#NAME?	5.00E-05	0.012978		
TCONS_00091674	XLOC_046231	15	49740628	49740790	#NAME?	5.00E-05	0.012978		
TCONS_00092009	XLOC_046565	15	55300741	55301265	Inf	5.00E-05	0.012978		
TCONS_00092316	XLOC_046871	15	59017873	59017948	#NAME?	5.00E-05	0.012978		
TCONS_00092566	XLOC_047106	15	66351761	66351836	#NAME?	5.00E-05	0.012978		
TCONS_00092578	XLOC_047118	15	66362166	66362848	#NAME?	5.00E-05	0.012978		
TCONS_00092730	XLOC_047270	15	69746461	69746548	#NAME?	5.00E-05	0.012978		
TCONS_00092917	XLOC_047457	15	75845931	75846068	#NAME?	5.00E-05	0.012978		
TCONS_00092983	XLOC_047523	15	76142154	76142230	#NAME?	5.00E-05	0.012978		
TCONS_00093377	XLOC_047917	15	85046425	85047142	Inf	5.00E-05	0.012978		
TCONS_00093604	XLOC_048142	15	92083259	92083628	Inf	5.00E-05	0.012978	Mycbp2	Metabolism
TCONS_00093622	XLOC_048160	15	92738375	92738452	#NAME?	5.00E-05	0.012978	Mycbp2	Metabolism
TCONS_00093676	XLOC_048214	15	95628316	95629010	#NAME?	5.00E-05	0.012978		
TCONS_00093688	XLOC_048226	15	95887288	95887679	#NAME?	5.00E-05	0.012978	AABR07019243.1	
TCONS_00093918	XLOC_048456	15	103570244	103570569	#NAME?	5.00E-05	0.012978	AABR07019388.1	
TCONS_00093997	XLOC_048535	15	104671375	104671450	#NAME?	5.00E-05	0.012978	Hs6st3	Metabolism
TCONS_00095072	XLOC_048935	16	19992983	20018056	-3.49762	5.00E-05	0.012978	Tmem221;Nxnl1;Slc27a1;Pgls;Fam129c	Metabolism
TCONS_00096244	XLOC_049226	16	73615914	73652384	-4.82629	5.00E-05	0.012978	Gpat4	

TCONS_00097288	XLOC_049466	16	19866021	19872774	-3.84823	5.00E-05	0.012978	Abhd8;Mrpl34;Dda1	Metabolism;Translation
TCONS_00097494	XLOC_049511	16	21272288	21288406	-5.19712	5.00E-05	0.012978	Gatad2a;Tssk6;LOC100911483;Yjefn3;Cilp2	Transcription;Signaling;Electron Transport
TCONS_00099287	XLOC_050316	16	6754093	6754838	Inf	5.00E-05	0.012978	Rft1	
TCONS_00099605	XLOC_050618	16	17471601	17472275	Inf	5.00E-05	0.012978		
TCONS_00099893	XLOC_050903	16	21919258	21921241	#NAME?	5.00E-05	0.012978		
TCONS_00100186	XLOC_051194	16	33837650	33837726	#NAME?	5.00E-05	0.012978		
TCONS_00100497	XLOC_051505	16	41146862	41147297	#NAME?	5.00E-05	0.012978	Neil3	DNA Repair
TCONS_00100524	XLOC_051532	16	41842340	41842574	#NAME?	5.00E-05	0.012978		
TCONS_00100719	XLOC_051712	16	47703826	47705323	Inf	5.00E-05	0.012978		
TCONS_00101178	XLOC_052153	16	58055779	58060323	-5.25523	5.00E-05	0.012978		
TCONS_00101188	XLOC_052163	16	58104454	58104530	#NAME?	5.00E-05	0.012978		
TCONS_00101353	XLOC_052328	16	60920972	60921391	#NAME?	5.00E-05	0.012978	Tnks	Metabolism
TCONS_00101491	XLOC_052464	16	68451149	68451298	#NAME?	5.00E-05	0.012978		
TCONS_00101834	XLOC_052805	16	78443129	78444008	Inf	5.00E-05	0.012978		
TCONS_00101980	XLOC_052950	16	83766252	83766327	#NAME?	5.00E-05	0.012978		
TCONS_00102065	XLOC_053035	16	90360898	90361401	#NAME?	5.00E-05	0.012978		
TCONS_00102072	XLOC_053042	16	90364695	90365025	#NAME?	5.00E-05	0.012978		
TCONS_00102137	XLOC_053062	17	1679488	1703408	-3.39025	5.00E-05	0.012978	Zfp367;Habp4;Cdc14b	Transcription;Unknown;Signaling
TCONS_00105063	XLOC_053740	17	8900035	8925503	#NAME?	5.00E-05	0.012978	Catsper3;Pcbd2	Metabolism
TCONS_00105299	XLOC_053804	17	13910788	13923337	#NAME?	5.00E-05	0.012978		
TCONS_00105716	XLOC_053918	17	27765007	27765754	-3.49829	5.00E-05	0.012978	RGD1563601	Metabolism
TCONS_00106733	XLOC_054186	17	70971913	71105286	-5.13652	5.00E-05	0.012978	Prkcq	Binding Protein
TCONS_00107974	XLOC_054962	17	10300468	10300701	#NAME?	5.00E-05	0.012978		
TCONS_00108021	XLOC_055009	17	11753661	11754499	-4.8869	5.00E-05	0.012978		
TCONS_00108086	XLOC_055074	17	13124273	13124349	#NAME?	5.00E-05	0.012978		
TCONS_00108284	XLOC_055264	17	15593103	15593941	Inf	5.00E-05	0.012978	LOC679342;Rn60_17_0156.3	
TCONS_00108350	XLOC_055330	17	16537757	16537898	#NAME?	5.00E-05	0.012978		
TCONS_00108377	XLOC_055357	17	16885877	16885951	#NAME?	5.00E-05	0.012978		
TCONS_00108450	XLOC_055430	17	18425809	18426155	-5.90586	5.00E-05	0.012978	Fam8a1	
TCONS_00108624	XLOC_055599	17	21265149	21266764	Inf	5.00E-05	0.012978		
TCONS_00108701	XLOC_055666	17	22595094	22595170	#NAME?	5.00E-05	0.012978		
TCONS_00108761	XLOC_055726	17	23564308	23564652	#NAME?	5.00E-05	0.012978		
TCONS_00108958	XLOC_055921	17	27710439	27712452	Inf	5.00E-05	0.012978	AABR07027387.1	
TCONS_00108972	XLOC_055935	17	28037779	28039557	Inf	5.00E-05	0.012978		
TCONS_00109105	XLOC_056067	17	31162238	31162375	#NAME?	5.00E-05	0.012978		
TCONS_00109268	XLOC_056226	17	35610998	35611485	Inf	5.00E-05	0.012978		
TCONS_00109866	XLOC_056818	17	44862243	44862399	#NAME?	5.00E-05	0.012978	Olr1654	
TCONS_00109874	XLOC_056826	17	44901842	44901918	#NAME?	5.00E-05	0.012978		
TCONS_00109963	XLOC_056914	17	45418371	45418941	#NAME?	5.00E-05	0.012978		
TCONS_00109967	XLOC_056918	17	45603773	45605104	Inf	5.00E-05	0.012978	Trim27	Metabolism
TCONS_00110439	XLOC_057387	17	51542361	51542438	#NAME?	5.00E-05	0.012978		

TCONS_00110469	XLOC_057417	17	52719185	52719933	#NAME?	5.00E-05	0.012978		
TCONS_00110839	XLOC_057747	17	58373759	58373834	#NAME?	5.00E-05	0.012978		
TCONS_00111422	XLOC_058254	17	68985894	68985969	#NAME?	5.00E-05	0.012978		
TCONS_00111454	XLOC_058284	17	70382350	70383471	Inf	5.00E-05	0.012978	Ankrd16	
TCONS_00111486	XLOC_058316	17	70969018	70969331	#NAME?	5.00E-05	0.012978	Prkcq	Binding Protein
TCONS_00111533	XLOC_058362	17	72507414	72507801	#NAME?	5.00E-05	0.012978		
TCONS_00111538	XLOC_058367	17	72675683	72676437	#NAME?	5.00E-05	0.012978		
TCONS_00111539	XLOC_058368	17	72832190	72832284	#NAME?	5.00E-05	0.012978		
TCONS_00111623	XLOC_058452	17	75140578	75140655	#NAME?	5.00E-05	0.012978		
TCONS_00111704	XLOC_058533	17	75717640	75717715	#NAME?	5.00E-05	0.012978		
TCONS_00111867	XLOC_058696	17	78986977	78987898	Inf	5.00E-05	0.012978		
TCONS_00114083	XLOC_059560	18	63086688	63108053	#NAME?	5.00E-05	0.012978	Cidea	Unknown
TCONS_00116450	XLOC_060188	18	423272	423481	#NAME?	5.00E-05	0.012978		
TCONS_00116664	XLOC_060402	18	4323791	4323904	Inf	5.00E-05	0.012978	7SK;Impact	
TCONS_00116786	XLOC_060524	18	11333601	11333704	#NAME?	5.00E-05	0.012978		
TCONS_00116809	XLOC_060547	18	12506427	12506584	#NAME?	5.00E-05	0.012978		
TCONS_00116879	XLOC_060616	18	15641951	15644012	Inf	5.00E-05	0.012978	Dsg2;Dsg3	Extracellular Matrix
TCONS_00116880	XLOC_060617	18	15644166	15644755	Inf	5.00E-05	0.012978	Dsg2;Dsg3	Extracellular Matrix
TCONS_00117014	XLOC_060751	18	19688434	19688622	#NAME?	5.00E-05	0.012978		
TCONS_00117082	XLOC_060819	18	22060803	22060879	#NAME?	5.00E-05	0.012978		
TCONS_00117104	XLOC_060841	18	22479476	22479618	#NAME?	5.00E-05	0.012978		
TCONS_00117454	XLOC_061156	18	29872081	29876399	-5.62455	5.00E-05	0.012978		
TCONS_00117787	XLOC_061488	18	34829593	34829939	#NAME?	5.00E-05	0.012978		
TCONS_00117932	XLOC_061633	18	39537174	39537250	#NAME?	5.00E-05	0.012978		
TCONS_00118156	XLOC_061857	18	40676709	40684096	Inf	5.00E-05	0.012978		
TCONS_00118303	XLOC_062004	18	43961757	43961833	#NAME?	5.00E-05	0.012978		
TCONS_00118418	XLOC_062119	18	47164341	47164425	#NAME?	5.00E-05	0.012978		
TCONS_00118586	XLOC_062286	18	55551162	55551602	Inf	5.00E-05	0.012978		
TCONS_00118647	XLOC_062347	18	57268006	57268592	#NAME?	5.00E-05	0.012978		
TCONS_00118711	XLOC_062411	18	58243864	58243954	#NAME?	5.00E-05	0.012978		
TCONS_00119256	XLOC_062941	18	74728109	74728185	#NAME?	5.00E-05	0.012978		
TCONS_00119263	XLOC_062948	18	74806858	74806975	#NAME?	5.00E-05	0.012978		
TCONS_00119418	XLOC_063032	18	77889653	77889941	#NAME?	5.00E-05	0.012978		
TCONS_00124400	XLOC_064426	19	2723441	2723618	#NAME?	5.00E-05	0.012978		
TCONS_00124750	XLOC_064774	19	15386936	15387035	#NAME?	5.00E-05	0.012978	Slc6a2	Metabolism
TCONS_00124762	XLOC_064786	19	16236221	16241390	-4.33895	5.00E-05	0.012978		
TCONS_00124997	XLOC_065020	19	19672117	19672588	Inf	5.00E-05	0.012978	Brd7	Epigenetic
TCONS_00125383	XLOC_065404	19	27763448	27763523	#NAME?	5.00E-05	0.012978		
TCONS_00125429	XLOC_065450	19	29374126	29374202	#NAME?	5.00E-05	0.012978	AABR07043564.1	
TCONS_00125792	XLOC_065794	19	34876620	34876716	#NAME?	5.00E-05	0.012978		
TCONS_00125825	XLOC_065827	19	36829629	36829704	#NAME?	5.00E-05	0.012978		
TCONS_00125945	XLOC_065943	19	40422434	40422910	Inf	5.00E-05	0.012978		
TCONS_00125954	XLOC_065952	19	40703002	40703167	#NAME?	5.00E-05	0.012978		
TCONS_00126004	XLOC_065993	19	42062663	42063357	Inf	5.00E-05	0.012978	Pkd1l3;Dhodh	Metabolism
TCONS_00126016	XLOC_066005	19	42372527	42372587	#NAME?	5.00E-05	0.012978		
TCONS_00126043	XLOC_066032	19	42619691	42619780	#NAME?	5.00E-05	0.012978		
TCONS_00126092	XLOC_066079	19	43749158	43749987	-5.62087	5.00E-05	0.012978	Znrf1	Transcription

TCONS_00126267	XLOC_066241	19	47548208	47549292	Inf	5.00E-05	0.012978		
TCONS_00126301	XLOC_066275	19	47609923	47610589	Inf	5.00E-05	0.012978		
TCONS_00126501	XLOC_066475	19	52116968	52117976	Inf	5.00E-05	0.012978	Necab2;Slc38a8	Receptor
TCONS_00126576	XLOC_066550	19	53703889	53705806	Inf	5.00E-05	0.012978		
TCONS_00126614	XLOC_066584	19	54279176	54279250	#NAME?	5.00E-05	0.012978		
TCONS_00126992	XLOC_066960	19	60789808	60789884	#NAME?	5.00E-05	0.012978		
TCONS_00129056	XLOC_067556	2	120783144	120783938	Inf	5.00E-05	0.012978	SNORD52	
TCONS_00129799	XLOC_067733	2	157127103	157142464	#NAME?	5.00E-05	0.012978		
TCONS_00131575	XLOC_068134	2	210685196	210688272	-4.60496	5.00E-05	0.012978	Eps8l3;NEWGENE_620381;Gstm6l	Unknown
TCONS_00135206	XLOC_069042	2	148386030	148386318	-5.9917	5.00E-05	0.012978	Metazoa_SRP;AABR07010706.1	
TCONS_00136342	XLOC_069290	2	192774591	192780634	-5.2269	5.00E-05	0.012978	Smcp	
TCONS_00136817	XLOC_069403	2	202794024	202816579	-5.03251	5.00E-05	0.012978	Fam46c	
TCONS_00136891	XLOC_069417	2	204847861	204848597	#NAME?	5.00E-05	0.012978		
TCONS_00137216	XLOC_069489	2	211245773	211332327	-4.43576	5.00E-05	0.012978	RGD1310209;U6atac;RGD1309139;Cox6b1	Electron Transport
TCONS_00137947	XLOC_069661	2	250072514	250079416	#NAME?	5.00E-05	0.012978		
TCONS_00138400	XLOC_069890	2	2055353	2057050	Inf	5.00E-05	0.012978		
TCONS_00138771	XLOC_070260	2	7442151	7442227	#NAME?	5.00E-05	0.012978		
TCONS_00138830	XLOC_070319	2	10540836	10540912	#NAME?	5.00E-05	0.012978		
TCONS_00138847	XLOC_070336	2	10973866	10973942	#NAME?	5.00E-05	0.012978		
TCONS_00138900	XLOC_070389	2	11871532	11872846	Inf	5.00E-05	0.012978		
TCONS_00139321	XLOC_070809	2	25630398	25630474	#NAME?	5.00E-05	0.012978		
TCONS_00139548	XLOC_071036	2	30094003	30094079	#NAME?	5.00E-05	0.012978	AABR07007839.1	
TCONS_00139549	XLOC_071037	2	30094161	30094237	#NAME?	5.00E-05	0.012978	AABR07007839.1	
TCONS_00139701	XLOC_071189	2	31364253	31364329	#NAME?	5.00E-05	0.012978		
TCONS_00139750	XLOC_071233	2	32152833	32153089	#NAME?	5.00E-05	0.012978		
TCONS_00140054	XLOC_071537	2	37014325	37014401	#NAME?	5.00E-05	0.012978	AABR07007980.2	
TCONS_00140338	XLOC_071818	2	40181563	40182541	Inf	5.00E-05	0.012978		
TCONS_00140426	XLOC_071906	2	42740285	42740436	#NAME?	5.00E-05	0.012978		
TCONS_00140710	XLOC_072168	2	50757497	50757651	#NAME?	5.00E-05	0.012978		
TCONS_00140859	XLOC_072317	2	54368373	54369994	Inf	5.00E-05	0.012978	Plcxd3	
TCONS_00141283	XLOC_072729	2	64325474	64325611	#NAME?	5.00E-05	0.012978		
TCONS_00141320	XLOC_072766	2	66139127	66140940	-5.63893	5.00E-05	0.012978		
TCONS_00141353	XLOC_072799	2	66437483	66437559	#NAME?	5.00E-05	0.012978		
TCONS_00141446	XLOC_072892	2	69748159	69748235	#NAME?	5.00E-05	0.012978		
TCONS_00141512	XLOC_072958	2	73004450	73005213	#NAME?	5.00E-05	0.012978		
TCONS_00141765	XLOC_073211	2	74759524	74759668	#NAME?	5.00E-05	0.012978		
TCONS_00141922	XLOC_073368	2	76733104	76733532	Inf	5.00E-05	0.012978		
TCONS_00141925	XLOC_073371	2	76734825	76735524	Inf	5.00E-05	0.012978		
TCONS_00142638	XLOC_074080	2	95690892	95691268	#NAME?	5.00E-05	0.012978		
TCONS_00143052	XLOC_074494	2	101958169	101958245	#NAME?	5.00E-05	0.012978		
TCONS_00143384	XLOC_074821	2	107681498	107681574	#NAME?	5.00E-05	0.012978		

TCONS_00143389	XLOC_074826	2	108407635	108408006	#NAME?	5.00E-05	0.012978		
TCONS_00144294	XLOC_075642	2	120159433	120159551	#NAME?	5.00E-05	0.012978		
TCONS_00144599	XLOC_075947	2	123554003	123554079	#NAME?	5.00E-05	0.012978	RGD1307100	
TCONS_00144817	XLOC_076163	2	128978801	128978904	#NAME?	5.00E-05	0.012978		
TCONS_00144887	XLOC_076233	2	131357839	131359410	Inf	5.00E-05	0.012978		
TCONS_00144926	XLOC_076272	2	132830147	132830460	#NAME?	5.00E-05	0.012978		
TCONS_00144962	XLOC_076308	2	133802947	133803145	#NAME?	5.00E-05	0.012978		
TCONS_00145108	XLOC_076454	2	139769679	139771052	Inf	5.00E-05	0.012978		
TCONS_00145276	XLOC_076565	2	141378029	141379329	Inf	5.00E-05	0.012978		
TCONS_00145506	XLOC_076766	2	145993930	145994005	#NAME?	5.00E-05	0.012978		
TCONS_00146062	XLOC_077308	2	157250989	157252504	Inf	5.00E-05	0.012978		
TCONS_00146124	XLOC_077370	2	160059917	160060136	#NAME?	5.00E-05	0.012978		
TCONS_00146304	XLOC_077550	2	165895745	165895867	#NAME?	5.00E-05	0.012978		
TCONS_00146627	XLOC_077846	2	171828850	171829026	#NAME?	5.00E-05	0.012978		
TCONS_00146772	XLOC_077991	2	176625549	176625625	#NAME?	5.00E-05	0.012978		
TCONS_00147033	XLOC_078250	2	183274088	183274773	Inf	5.00E-05	0.012978		
TCONS_00147066	XLOC_078283	2	183586882	183588056	Inf	5.00E-05	0.012978	Arfip1	Signaling
TCONS_00147223	XLOC_078426	2	186337858	186338437	Inf	5.00E-05	0.012978	Cd1d1	Receptor
TCONS_00147467	XLOC_078659	2	192970478	192970566	Inf	5.00E-05	0.012978	AABR07012314.1	
TCONS_00147509	XLOC_078701	2	194077766	194077842	#NAME?	5.00E-05	0.012978	AABR07012342.1	
TCONS_00147520	XLOC_078712	2	194238428	194238840	#NAME?	5.00E-05	0.012978		
TCONS_00147531	XLOC_078723	2	194375039	194375308	#NAME?	5.00E-05	0.012978		
TCONS_00147546	XLOC_078738	2	194760457	194760533	#NAME?	5.00E-05	0.012978		
TCONS_00147641	XLOC_078831	2	195984039	195984720	Inf	5.00E-05	0.012978		
TCONS_00147650	XLOC_078840	2	195993120	195994517	Inf	5.00E-05	0.012978	Pogz	Transcription
TCONS_00147778	XLOC_078964	2	198609331	198609441	#NAME?	5.00E-05	0.012978	AABR07012588.1	
TCONS_00148000	XLOC_079183	2	203066420	203066508	#NAME?	5.00E-05	0.012978		
TCONS_00148086	XLOC_079269	2	205152710	205152838	#NAME?	5.00E-05	0.012978	Tspan2	Signaling
TCONS_00148354	XLOC_079537	2	210641228	210641279	#NAME?	5.00E-05	0.012978		
TCONS_00148463	XLOC_079646	2	214827535	214827611	#NAME?	5.00E-05	0.012978		
TCONS_00148491	XLOC_079674	2	215274395	215274471	#NAME?	5.00E-05	0.012978		
TCONS_00148747	XLOC_079930	2	221665837	221665914	#NAME?	5.00E-05	0.012978	Mir137;AABR07013073.1;AABR07013073.4	
TCONS_00148766	XLOC_079949	2	221686139	221686215	#NAME?	5.00E-05	0.012978		
TCONS_00148776	XLOC_079959	2	221740113	221740211	#NAME?	5.00E-05	0.012978		
TCONS_00148925	XLOC_080105	2	226846307	226846537	#NAME?	5.00E-05	0.012978		
TCONS_00148986	XLOC_080165	2	230521738	230524086	Inf	5.00E-05	0.012978		
TCONS_00148995	XLOC_080174	2	230573712	230574909	Inf	5.00E-05	0.012978		
TCONS_00149376	XLOC_080555	2	236061099	236061294	#NAME?	5.00E-05	0.012978	AABR07013410.4	
TCONS_00149548	XLOC_080723	2	239003850	239005007	Inf	5.00E-05	0.012978		
TCONS_00149553	XLOC_080728	2	239013814	239014684	Inf	5.00E-05	0.012978		
TCONS_00149637	XLOC_080807	2	241545386	241545463	#NAME?	5.00E-05	0.012978	Bank1	Development
TCONS_00149645	XLOC_080815	2	242400837	242400913	#NAME?	5.00E-05	0.012978		
TCONS_00149966	XLOC_081126	2	249867406	249867611	#NAME?	5.00E-05	0.012978		

TCONS_00150101	XLOC_081261	2	254475670	254475766	#NAME?	5.00E-05	0.012978		
TCONS_00150127	XLOC_081287	2	256506714	256506914	#NAME?	5.00E-05	0.012978		
TCONS_00150161	XLOC_081320	2	258709699	258709791	#NAME?	5.00E-05	0.012978		
TCONS_00151430	XLOC_081686	20	8165306	8169555	-4.9238	5.00E-05	0.012978	Pim1	Signaling
TCONS_00153946	XLOC_082248	20	10757853	10844178	-5.52586	5.00E-05	0.012978	Hsf2bp;Rrp1b	
TCONS_00154248	XLOC_082335	20	20020366	20047380	3.69723	5.00E-05	0.012978	AABR07044767.1	
TCONS_00155419	XLOC_082724	20	5158288	5159295	Inf	5.00E-05	0.012978	Prrc2a;SNORA38;Aif1	Signaling
TCONS_00155482	XLOC_082785	20	6512394	6512470	#NAME?	5.00E-05	0.012978	Ppil1	Immune
TCONS_00155610	XLOC_082913	20	10119453	10119529	#NAME?	5.00E-05	0.012978	Pde9a	Signaling
TCONS_00155748	XLOC_083028	20	12151448	12152439	Inf	5.00E-05	0.012978		
TCONS_00155894	XLOC_083174	20	18699505	18700018	Inf	5.00E-05	0.012978		
TCONS_00155984	XLOC_083264	20	19218542	19218617	#NAME?	5.00E-05	0.012978		
TCONS_00156096	XLOC_083372	20	21156750	21165905	#NAME?	5.00E-05	0.012978		
TCONS_00156354	XLOC_083609	20	26754737	26754883	#NAME?	5.00E-05	0.012978	Herc4	Metabolism
TCONS_00156482	XLOC_083731	20	28420285	28421093	Inf	5.00E-05	0.012978		
TCONS_00156486	XLOC_083735	20	28425281	28427311	Inf	5.00E-05	0.012978		
TCONS_00156513	XLOC_083755	20	28468957	28470167	Inf	5.00E-05	0.012978		
TCONS_00156536	XLOC_083774	20	28502352	28503251	Inf	5.00E-05	0.012978		
TCONS_00156676	XLOC_083911	20	31703951	31704336	#NAME?	5.00E-05	0.012978	AABR07045015.1	
TCONS_00157148	XLOC_084381	20	46361312	46361386	#NAME?	5.00E-05	0.012978		
TCONS_00157429	XLOC_084658	20	50004320	50005219	Inf	5.00E-05	0.012978		
TCONS_00157442	XLOC_084671	20	50018829	50018905	#NAME?	5.00E-05	0.012978		
TCONS_00157483	XLOC_084710	20	51043231	51043472	#NAME?	5.00E-05	0.012978		
TCONS_00157490	XLOC_084717	20	51164920	51165612	Inf	5.00E-05	0.012978		
TCONS_00157613	XLOC_084840	20	55578992	55579584	Inf	5.00E-05	0.012978		
TCONS_00157898	XLOC_084908	3	2970089	3211910	#NAME?	5.00E-05	0.012978	Obp2b;AABR07051244.1	Binding Protein
TCONS_00160105	XLOC_085432	3	75024205	75024479	-5.06453	5.00E-05	0.012978	AABR07052780.1;Metazoa_SRP;AABR07052780.2	
TCONS_00161668	XLOC_085832	3	122926487	122932281	-5.79431	5.00E-05	0.012978	Cpxm1;LOC100365450;AABR07053736.1;Pced1a	Protease
TCONS_00164719	XLOC_086497	3	9629099	9637849	-5.33992	5.00E-05	0.012978	RGD1311084;Ntmt1	
TCONS_00169364	XLOC_087640	3	160918577	160922341	-4.45137	5.00E-05	0.012978	Sys1;Tp53tg5	Transport
TCONS_00170135	XLOC_087812	3	290552	290649	#NAME?	5.00E-05	0.012978		
TCONS_00170797	XLOC_088466	3	15996026	15996102	#NAME?	5.00E-05	0.012978	Olr402	
TCONS_00170804	XLOC_088473	3	16452184	16452492	#NAME?	5.00E-05	0.012978		
TCONS_00170838	XLOC_088507	3	18741953	18742033	#NAME?	5.00E-05	0.012978		
TCONS_00170854	XLOC_088523	3	18963095	18963170	#NAME?	5.00E-05	0.012978	AABR07051665.1	
TCONS_00170863	XLOC_088532	3	19193805	19193881	#NAME?	5.00E-05	0.012978		

TCONS_00170889	XLOC_088558	3	21368078	21368153	#NAME?	5.00E-05	0.012978	Olr434;AABR07051787.1	Receptor
TCONS_00171328	XLOC_088959	3	26898010	26898863	Inf	5.00E-05	0.012978		
TCONS_00171396	XLOC_089027	3	26972733	26973709	Inf	5.00E-05	0.012978		
TCONS_00171408	XLOC_089039	3	26987224	26988138	Inf	5.00E-05	0.012978		
TCONS_00171410	XLOC_089041	3	26989634	26990680	Inf	5.00E-05	0.012978		
TCONS_00171498	XLOC_089129	3	27108926	27109597	Inf	5.00E-05	0.012978		
TCONS_00171943	XLOC_089549	3	37538196	37538261	#NAME?	5.00E-05	0.012978	Tnfaip6	Extracellular Matrix
TCONS_00171972	XLOC_089569	3	38420633	38421254	Inf	5.00E-05	0.012978		
TCONS_00172030	XLOC_089611	3	38492059	38492660	Inf	5.00E-05	0.012978		
TCONS_00172316	XLOC_089893	3	45636022	45636201	#NAME?	5.00E-05	0.012978	U6	
TCONS_00172367	XLOC_089926	3	46754933	46754990	#NAME?	5.00E-05	0.012978		
TCONS_00172404	XLOC_089959	3	49083599	49083674	#NAME?	5.00E-05	0.012978		
TCONS_00172455	XLOC_090010	3	51334304	51335527	Inf	5.00E-05	0.012978		
TCONS_00172685	XLOC_090240	3	56800865	56800942	#NAME?	5.00E-05	0.012978	Erich2	
TCONS_00172834	XLOC_090386	3	59746822	59748432	Inf	5.00E-05	0.012978		
TCONS_00173022	XLOC_090558	3	62212941	62213017	#NAME?	5.00E-05	0.012978		
TCONS_00173465	XLOC_090998	3	75022159	75022235	#NAME?	5.00E-05	0.012978	AABR07052780.1;Metazoa_SRP;AABR07052780.2	
TCONS_00173591	XLOC_091124	3	81093980	81094915	Inf	5.00E-05	0.012978		
TCONS_00173618	XLOC_091151	3	81447660	81448242	#NAME?	5.00E-05	0.012978		
TCONS_00173722	XLOC_091254	3	83693153	83693218	#NAME?	5.00E-05	0.012978		
TCONS_00173750	XLOC_091282	3	84921063	84921152	#NAME?	5.00E-05	0.012978		
TCONS_00173769	XLOC_091301	3	87049072	87049366	#NAME?	5.00E-05	0.012978		
TCONS_00173919	XLOC_091450	3	92793182	92793318	#NAME?	5.00E-05	0.012978	Cd44	
TCONS_00174089	XLOC_091616	3	99526094	99526170	#NAME?	5.00E-05	0.012978		
TCONS_00174098	XLOC_091625	3	100167543	100168528	Inf	5.00E-05	0.012978		
TCONS_00174150	XLOC_091677	3	102464359	102464967	#NAME?	5.00E-05	0.012978	Olr748;Olr749	
TCONS_00174484	XLOC_091962	3	111643716	111643792	#NAME?	5.00E-05	0.012978		
TCONS_00174552	XLOC_092025	3	113077324	113077951	Inf	5.00E-05	0.012978	U6	
TCONS_00174553	XLOC_092026	3	113078084	113078997	Inf	5.00E-05	0.012978	U6	
TCONS_00174905	XLOC_092326	3	120807898	120808610	Inf	5.00E-05	0.012978		
TCONS_00175057	XLOC_092476	3	123794592	123795267	Inf	5.00E-05	0.012978	Mavs	
TCONS_00175089	XLOC_092508	3	124290672	124290884	#NAME?	5.00E-05	0.012978		
TCONS_00175093	XLOC_092512	3	124480800	124480926	#NAME?	5.00E-05	0.012978		
TCONS_00175192	XLOC_092611	3	127070243	127070318	#NAME?	5.00E-05	0.012978		
TCONS_00175412	XLOC_092730	3	128072255	128073071	Inf	5.00E-05	0.012978		
TCONS_00175502	XLOC_092820	3	129492265	129492340	#NAME?	5.00E-05	0.012978	Ankef1	
TCONS_00175595	XLOC_092912	3	132450682	132450802	#NAME?	5.00E-05	0.012978		
TCONS_00176037	XLOC_093352	3	135485066	135485191	#NAME?	5.00E-05	0.012978		
TCONS_00176174	XLOC_093488	3	136586726	136587893	Inf	5.00E-05	0.012978	AABR07054025.1	
TCONS_00176831	XLOC_094145	3	144076344	144076655	#NAME?	5.00E-05	0.012978		
TCONS_00177656	XLOC_094902	3	161503175	161503251	#NAME?	5.00E-05	0.012978	Ncoa5	Receptor
TCONS_00177836	XLOC_095079	3	164784517	164785449	Inf	5.00E-05	0.012978		
TCONS_00177893	XLOC_095135	3	165670173	165670248	#NAME?	5.00E-05	0.012978	SNORA17;Zfp93	

TCONS_00177902	XLOC_095144	3	165992698	165993225	#NAME?	5.00E-05	0.012978	LOC108350528	
TCONS_00178153	XLOC_095348	3	168753147	168753233	#NAME?	5.00E-05	0.012978		
TCONS_00178164	XLOC_095359	3	169444280	169444356	#NAME?	5.00E-05	0.012978		
TCONS_00178201	XLOC_095396	3	170874893	170875094	#NAME?	5.00E-05	0.012978	Bmp7	Growth Factors & Cytokines
TCONS_00178238	XLOC_095433	3	171506321	171506412	#NAME?	5.00E-05	0.012978		
TCONS_00178415	XLOC_095600	3	174860152	174861304	Inf	5.00E-05	0.012978		
TCONS_00178560	XLOC_095745	3	175052200	175053909	Inf	5.00E-05	0.012978		
TCONS_00178577	XLOC_095762	3	175073168	175074116	Inf	5.00E-05	0.012978		
TCONS_00179833	XLOC_096227	4	55602549	55609284	6.02166	5.00E-05	0.012978		
TCONS_00179888	XLOC_096238	4	56674831	56704580	-4.72899	5.00E-05	0.012978	Ccdc136;AABR07072995.1;Flnc	Cytoskeleton
TCONS_00180070	XLOC_096264	4	59364134	59371904	-4.83115	5.00E-05	0.012978	LOC689042	
TCONS_00185861	XLOC_097746	4	112310381	112311935	-4.0351	5.00E-05	0.012978		
TCONS_00187699	XLOC_098215	4	169020678	169036976	-4.58119	5.00E-05	0.012978	Fam234b;Gsg1	
TCONS_00187701	XLOC_098216	4	169091840	169093135	-4.79753	5.00E-05	0.012978	Pbp2	
TCONS_00188444	XLOC_098553	4	4778838	4779074	#NAME?	5.00E-05	0.012978		
TCONS_00188473	XLOC_098582	4	5176507	5176644	#NAME?	5.00E-05	0.012978		
TCONS_00188502	XLOC_098611	4	5413621	5413717	#NAME?	5.00E-05	0.012978	AABR07059153.1	
TCONS_00188568	XLOC_098677	4	7614488	7614564	#NAME?	5.00E-05	0.012978		
TCONS_00188592	XLOC_098699	4	8309849	8310711	Inf	5.00E-05	0.012978		
TCONS_00188825	XLOC_098932	4	9790206	9790368	#NAME?	5.00E-05	0.012978		
TCONS_00189058	XLOC_099162	4	14785725	14786686	Inf	5.00E-05	0.012978		
TCONS_00189371	XLOC_099475	4	24537466	24537556	#NAME?	5.00E-05	0.012978		
TCONS_00189462	XLOC_099566	4	26967718	26967794	#NAME?	5.00E-05	0.012978		
TCONS_00189473	XLOC_099577	4	27051053	27051288	#NAME?	5.00E-05	0.012978		
TCONS_00189891	XLOC_099972	4	44409148	44409638	#NAME?	5.00E-05	0.012978		
TCONS_00189922	XLOC_100003	4	44915135	44915908	Inf	5.00E-05	0.012978		
TCONS_00190053	XLOC_100134	4	48349780	48350738	Inf	5.00E-05	0.012978		
TCONS_00190427	XLOC_100504	4	56669359	56669537	#NAME?	5.00E-05	0.012978	Ccdc136;AABR07072995.1	
TCONS_00190468	XLOC_100545	4	57922616	57922732	#NAME?	5.00E-05	0.012978	Cpa5	Protease
TCONS_00190587	XLOC_100657	4	61403951	61404415	Inf	5.00E-05	0.012978		
TCONS_00190756	XLOC_100822	4	63178805	63179110	#NAME?	5.00E-05	0.012978		
TCONS_00191023	XLOC_101085	4	69179225	69179300	#NAME?	5.00E-05	0.012978		
TCONS_00191198	XLOC_101256	4	72611399	72612065	#NAME?	5.00E-05	0.012978	Olr818	Receptor
TCONS_00191282	XLOC_101338	4	77464940	77465016	#NAME?	5.00E-05	0.012978	Y_RNA;Pdia4	Metabolism
TCONS_00191469	XLOC_101525	4	80951686	80953287	-3.58811	5.00E-05	0.012978		
TCONS_00191756	XLOC_101805	4	89042574	89042649	#NAME?	5.00E-05	0.012978		
TCONS_00191917	XLOC_101922	4	92024977	92025053	#NAME?	5.00E-05	0.012978		
TCONS_00192173	XLOC_102173	4	98597848	98598453	Inf	5.00E-05	0.012978	Rpia	
TCONS_00192324	XLOC_102321	4	100341554	100342106	Inf	5.00E-05	0.012978		
TCONS_00193089	XLOC_103079	4	120148528	120150392	#NAME?	5.00E-05	0.012978	Gata2;Dnajb8;AABR07061382.1	Transcription;Protein Binding
TCONS_00193120	XLOC_103110	4	121036805	121036879	#NAME?	5.00E-05	0.012978	Prr20e	

TCONS_00193121	XLOC_103111	4	121037000	121037076	#NAME?	5.00E-05	0.012978	Prr20e	
TCONS_00193336	XLOC_103322	4	124488922	124489988	Inf	5.00E-05	0.012978		
TCONS_00193519	XLOC_103466	4	126752509	126752930	#NAME?	5.00E-05	0.012978		
TCONS_00193646	XLOC_103593	4	130820147	130820562	#NAME?	5.00E-05	0.012978		
TCONS_00193846	XLOC_103792	4	142659933	142660240	#NAME?	5.00E-05	0.012978		
TCONS_00194304	XLOC_104217	4	155177349	155177674	#NAME?	5.00E-05	0.012978	AABR07061962.1	
TCONS_00194406	XLOC_104319	4	157907474	157907841	#NAME?	5.00E-05	0.012978		
TCONS_00194479	XLOC_104392	4	160362495	160368669	-4.19901	5.00E-05	0.012978		
TCONS_00194614	XLOC_104525	4	162857962	162858309	Inf	5.00E-05	0.012978	Clec2e	
TCONS_00194897	XLOC_104799	4	169100954	169101256	#NAME?	5.00E-05	0.012978	Pbp2	
TCONS_00194946	XLOC_104848	4	170059322	170060365	Inf	5.00E-05	0.012978		
TCONS_00195008	XLOC_104910	4	170330513	170331293	Inf	5.00E-05	0.012978		
TCONS_00195067	XLOC_104967	4	171468743	171469690	Inf	5.00E-05	0.012978	Ptpro;Eps8	Signaling
TCONS_00197187	XLOC_106016	5	78073649	78082242	-4.8171	5.00E-05	0.012978	Rn50_5_0814.8	
TCONS_00197204	XLOC_106021	5	78334283	78362065	-5.16548	5.00E-05	0.012978	Bspry;Hdhd3;Rn50_5_0825.2;Alad	Development;Metabolism
TCONS_00199606	XLOC_106548	5	154976164	154980712	-4.90584	5.00E-05	0.012978	LOC102552669	
TCONS_00201011	XLOC_106852	5	17398873	17434282	-5.00962	5.00E-05	0.012978	RGD1563405	
TCONS_00201540	XLOC_106973	5	54480786	54482159	-5.00975	5.00E-05	0.012978	RGD1306195	
TCONS_00201585	XLOC_106985	5	57254100	57267007	-4.35876	5.00E-05	0.012978	Spink4;Bag1;Chmp5	Signaling;Apoptosis;Binding Protein
TCONS_00202696	XLOC_107254	5	100887987	100897591	#NAME?	5.00E-05	0.012978	AABR07049035.1	
TCONS_00204031	XLOC_107601	5	146023695	146069670	-4.91292	5.00E-05	0.012978	LOC682102	
TCONS_00204384	XLOC_107667	5	150683073	150704178	3.40525	5.00E-05	0.012978	Sesn2	Metabolism
TCONS_00205630	XLOC_107913	5	172077281	172078760	-4.9539	5.00E-05	0.012978	Actrt2	Cytoskeleton
TCONS_00205864	XLOC_108001	5	428752	428939	#NAME?	5.00E-05	0.012978		
TCONS_00206245	XLOC_108378	5	4332652	4332727	#NAME?	5.00E-05	0.012978		
TCONS_00206381	XLOC_108512	5	8261628	8261704	#NAME?	5.00E-05	0.012978		
TCONS_00206562	XLOC_108690	5	12668426	12668502	#NAME?	5.00E-05	0.012978	AABR07046961.1	
TCONS_00206581	XLOC_108709	5	13113944	13115335	Inf	5.00E-05	0.012978		
TCONS_00206610	XLOC_108738	5	13598075	13598283	#NAME?	5.00E-05	0.012978		
TCONS_00206714	XLOC_108842	5	16714489	16714646	#NAME?	5.00E-05	0.012978	Rps20;snoU54	Translation
TCONS_00206774	XLOC_108902	5	17856952	17857903	#NAME?	5.00E-05	0.012978		
TCONS_00207135	XLOC_109244	5	24434505	24434581	#NAME?	5.00E-05	0.012978	Ccne2	Cell Cycle
TCONS_00207200	XLOC_109309	5	25869970	25870035	#NAME?	5.00E-05	0.012978		
TCONS_00207375	XLOC_109482	5	30050062	30051394	Inf	5.00E-05	0.012978		
TCONS_00207500	XLOC_109607	5	32456989	32457075	#NAME?	5.00E-05	0.012978		
TCONS_00207515	XLOC_109622	5	34979884	34979960	#NAME?	5.00E-05	0.012978		
TCONS_00207576	XLOC_109683	5	37051433	37051540	#NAME?	5.00E-05	0.012978		
TCONS_00207620	XLOC_109727	5	40841315	40841391	#NAME?	5.00E-05	0.012978	Manea	Golgi
TCONS_00207667	XLOC_109774	5	45694926	45695140	#NAME?	5.00E-05	0.012978		

TCONS_00207772	XLOC_109877	5	48497830	48499140	Inf	5.00E-05	0.012978	Pnrc1	
TCONS_00207912	XLOC_110007	5	53647870	53647945	#NAME?	5.00E-05	0.012978		
TCONS_00208107	XLOC_110199	5	59114223	59115407	Inf	5.00E-05	0.012978	AC121204.3	
TCONS_00208197	XLOC_110271	5	59902995	59903226	#NAME?	5.00E-05	0.012978	LOC100359916	Unknown
TCONS_00208289	XLOC_110363	5	63127406	63128698	Inf	5.00E-05	0.012978	Tgfbr1	Growth Factors & Cytokines
TCONS_00208371	XLOC_110445	5	63927435	63928288	Inf	5.00E-05	0.012978	Stx17;AC142180.1;Erp44	Transport; Metabolism
TCONS_00208444	XLOC_110518	5	67053621	67053696	#NAME?	5.00E-05	0.012978		
TCONS_00208452	XLOC_110526	5	68312729	68312832	#NAME?	5.00E-05	0.012978		
TCONS_00208778	XLOC_110852	5	75742584	75743416	Inf	5.00E-05	0.012978		
TCONS_00208944	XLOC_111018	5	77050423	77050567	#NAME?	5.00E-05	0.012978	Snx30	Transport
TCONS_00209139	XLOC_111212	5	88417138	88417213	#NAME?	5.00E-05	0.012978		
TCONS_00209322	XLOC_111395	5	95161363	95161439	#NAME?	5.00E-05	0.012978		
TCONS_00209349	XLOC_111422	5	96029111	96029187	#NAME?	5.00E-05	0.012978		
TCONS_00209398	XLOC_111471	5	100253627	100253704	#NAME?	5.00E-05	0.012978		
TCONS_00209823	XLOC_111896	5	105021065	105021101	#NAME?	5.00E-05	0.012978		
TCONS_00210013	XLOC_112067	5	108752793	108752869	#NAME?	5.00E-05	0.012978	Zfp353	
TCONS_00210018	XLOC_112072	5	109870385	109870461	#NAME?	5.00E-05	0.012978		
TCONS_00210368	XLOC_112422	5	113465801	113466299	Inf	5.00E-05	0.012978		
TCONS_00210369	XLOC_112423	5	113466398	113467092	Inf	5.00E-05	0.012978		
TCONS_00210446	XLOC_112497	5	115549527	115549675	-3.47394	5.00E-05	0.012978		
TCONS_00210452	XLOC_112503	5	116214387	116214466	#NAME?	5.00E-05	0.012978		
TCONS_00210483	XLOC_112534	5	116976207	116976328	#NAME?	5.00E-05	0.012978	LOC100912024	
TCONS_00210645	XLOC_112695	5	120322234	120322810	Inf	5.00E-05	0.012978	AABR07049389.1	
TCONS_00211312	XLOC_113313	5	132839849	132839990	#NAME?	5.00E-05	0.012978		
TCONS_00211651	XLOC_113646	5	142155371	142156673	Inf	5.00E-05	0.012978		
TCONS_00212069	XLOC_114054	5	147245662	147245826	#NAME?	5.00E-05	0.012978	Rnf19b	
TCONS_00212081	XLOC_114066	5	147452265	147453186	Inf	5.00E-05	0.012978		
TCONS_00212258	XLOC_114229	5	150543392	150544252	Inf	5.00E-05	0.012978	Phactr4	Signaling
TCONS_00212270	XLOC_114241	5	150709512	150710336	Inf	5.00E-05	0.012978	Sesn2;Atpif1	Metabolism
TCONS_00212940	XLOC_114907	5	161942482	161942632	#NAME?	5.00E-05	0.012978	Pdpn	Development
TCONS_00213076	XLOC_115043	5	164664151	164664829	Inf	5.00E-05	0.012978	LOC100911456	
TCONS_00213263	XLOC_115217	5	168827294	168828125	Inf	5.00E-05	0.012978	Camta1	Transcription
TCONS_00213280	XLOC_115228	5	168883172	168884041	Inf	5.00E-05	0.012978	Camta1	Transcription
TCONS_00213432	XLOC_115380	5	171800701	171801378	Inf	5.00E-05	0.012978		
TCONS_00213685	XLOC_115632	5	173632830	173633608	Inf	5.00E-05	0.012978	Isg15;Perm1	Signaling
TCONS_00214029	XLOC_115708	6	10674370	10704977	-3.40018	5.00E-05	0.012978	Socs5	Signaling
TCONS_00214505	XLOC_115805	6	28663601	28666987	Inf	5.00E-05	0.012978	Cenpo;Ptrhd1	
TCONS_00216192	XLOC_116210	6	99808698	99809127	#NAME?	5.00E-05	0.012978	Churc1	
TCONS_00218619	XLOC_116759	6	25666647	26051229	-5.86044	5.00E-05	0.012978	Bre;Rbks	Signaling

TCONS_00219740	XLOC_117043	6	77418095	77421286	#NAME?	5.00E-05	0.012978	Nkx2-1	Transcription
TCONS_00220294	XLOC_117214	6	103932427	103935122	-4.36384	5.00E-05	0.012978	AABR07065010.1;LOC500684	Translation
TCONS_00220824	XLOC_117339	6	122370950	122400277	-4.55642	5.00E-05	0.012978		
TCONS_00221854	XLOC_117723	6	3876604	3876907	#NAME?	5.00E-05	0.012978		
TCONS_00222752	XLOC_118591	6	14706738	14706814	#NAME?	5.00E-05	0.012978	Nrxn1	Receptor
TCONS_00222909	XLOC_118748	6	20657177	20657821	Inf	5.00E-05	0.012978		
TCONS_00222913	XLOC_118752	6	20667974	20668941	Inf	5.00E-05	0.012978		
TCONS_00222951	XLOC_118790	6	20750324	20750936	Inf	5.00E-05	0.012978		
TCONS_00223407	XLOC_119241	6	30307601	30308095	Inf	5.00E-05	0.012978		
TCONS_00223704	XLOC_119538	6	31812876	31812948	#NAME?	5.00E-05	0.012978		
TCONS_00224415	XLOC_120243	6	41736327	41736403	#NAME?	5.00E-05	0.012978		
TCONS_00225185	XLOC_121004	6	54242706	54243249	Inf	5.00E-05	0.012978		
TCONS_00225456	XLOC_121274	6	59963897	59963973	#NAME?	5.00E-05	0.012978		
TCONS_00225846	XLOC_121624	6	63428686	63428998	#NAME?	5.00E-05	0.012978		
TCONS_00226087	XLOC_121852	6	68709744	68709820	#NAME?	5.00E-05	0.012978		
TCONS_00226458	XLOC_122203	6	79241395	79241471	#NAME?	5.00E-05	0.012978	AC098459.2	
TCONS_00226459	XLOC_122204	6	79282321	79282451	#NAME?	5.00E-05	0.012978		
TCONS_00226531	XLOC_122276	6	83445475	83445556	#NAME?	5.00E-05	0.012978	Lrfn5;7SK	Receptor
TCONS_00226622	XLOC_122367	6	85789710	85789827	#NAME?	5.00E-05	0.012978		
TCONS_00226744	XLOC_122489	6	87576220	87576296	#NAME?	5.00E-05	0.012978		
TCONS_00227058	XLOC_122803	6	93278101	93278310	#NAME?	5.00E-05	0.012978	Frmd6	Signaling
TCONS_00227127	XLOC_122872	6	94595152	94596197	Inf	5.00E-05	0.012978		
TCONS_00227206	XLOC_122951	6	95112636	95113235	Inf	5.00E-05	0.012978		
TCONS_00227710	XLOC_123428	6	103892711	103893170	#NAME?	5.00E-05	0.012978		
TCONS_00227721	XLOC_123439	6	104274167	104274312	#NAME?	5.00E-05	0.012978	U6;Erh	Transcription
TCONS_00228328	XLOC_124026	6	117557287	117557363	#NAME?	5.00E-05	0.012978		
TCONS_00228341	XLOC_124039	6	118595758	118595832	#NAME?	5.00E-05	0.012978		
TCONS_00228384	XLOC_124082	6	120655123	120655199	#NAME?	5.00E-05	0.012978	AABR07065312.1	
TCONS_00228431	XLOC_124129	6	122162699	122163639	Inf	5.00E-05	0.012978		
TCONS_00229205	XLOC_124873	6	141255289	141255401	#NAME?	5.00E-05	0.012978		
TCONS_00229224	XLOC_124892	6	143355454	143355655	#NAME?	5.00E-05	0.012978		
TCONS_00231605	XLOC_125584	7	77030669	77031086	#NAME?	5.00E-05	0.012978		
TCONS_00232999	XLOC_125897	7	123296182	123304066	-5.14603	5.00E-05	0.012978	LOC100359574;LOC100362109;Mei1	Transcription
TCONS_00234654	XLOC_126310	7	12282009	12284437	-2.48996	5.00E-05	0.012978	Apc2;Rps15;Dazap1	Cytoskeleton;Translation
TCONS_00235263	XLOC_126444	7	26138884	26144556	-4.97834	5.00E-05	0.012978	Fhl4;AABR07056464.1	
TCONS_00238108	XLOC_127110	7	125853440	125893180	-3.92478	5.00E-05	0.012978	Nup50;RGD1304694;Mir1249	Transport
TCONS_00238326	XLOC_127147	7	130360516	130368819	#NAME?	5.00E-05	0.012978	Odf3b;Klhdc7b;Syce3	Unknown
TCONS_00238480	XLOC_127200	7	136526495	136862420	-6.50691	5.00E-05	0.012978	Nell2	Signaling

TCONS_00239149	XLOC_127372	7	407083	407702	Inf	5.00E-05	0.012978	AABR07055191.1	
TCONS_00239191	XLOC_127414	7	700137	702318	-4.54058	5.00E-05	0.012978	5_8S_rRNA	
TCONS_00239389	XLOC_127609	7	6890573	6890773	#NAME?	5.00E-05	0.012978	AABR07055600.1	
TCONS_00239754	XLOC_127972	7	14956428	14956503	#NAME?	5.00E-05	0.012978		
TCONS_00239761	XLOC_127979	7	15051936	15052011	#NAME?	5.00E-05	0.012978	U1;5S_rRNA	
TCONS_00240167	XLOC_128385	7	22530986	22531349	#NAME?	5.00E-05	0.012978		
TCONS_00241064	XLOC_129276	7	48837741	48837816	#NAME?	5.00E-05	0.012978		
TCONS_00241112	XLOC_129324	7	52421515	52422443	Inf	5.00E-05	0.012978		
TCONS_00241405	XLOC_129617	7	53859217	53859923	Inf	5.00E-05	0.012978		
TCONS_00241858	XLOC_130070	7	62784592	62785560	Inf	5.00E-05	0.012978		
TCONS_00242005	XLOC_130214	7	66576394	66576579	#NAME?	5.00E-05	0.012978		
TCONS_00242086	XLOC_130295	7	69515251	69515368	#NAME?	5.00E-05	0.012978		
TCONS_00242090	XLOC_130299	7	69578637	69578735	#NAME?	5.00E-05	0.012978		
TCONS_00242318	XLOC_130510	7	75402091	75403442	Inf	5.00E-05	0.012978	Pabpc1	Translation
TCONS_00242354	XLOC_130546	7	75837550	75837635	#NAME?	5.00E-05	0.012978		
TCONS_00242355	XLOC_130547	7	75846784	75847444	Inf	5.00E-05	0.012978	LOC102555534	
TCONS_00242432	XLOC_130621	7	76901793	76902079	Inf	5.00E-05	0.012978		
TCONS_00242471	XLOC_130660	7	77029907	77029983	#NAME?	5.00E-05	0.012978		
TCONS_00242532	XLOC_130721	7	77479452	77480090	Inf	5.00E-05	0.012978		
TCONS_00242718	XLOC_130907	7	83994695	83994863	#NAME?	5.00E-05	0.012978	AABR07057683.1	
TCONS_00242764	XLOC_130953	7	87180711	87180786	#NAME?	5.00E-05	0.012978	Csmd3	
TCONS_00242773	XLOC_130962	7	87370172	87370248	#NAME?	5.00E-05	0.012978	Csmd3	
TCONS_00242798	XLOC_130987	7	88876049	88876631	Inf	5.00E-05	0.012978		
TCONS_00242973	XLOC_131162	7	90741745	90741821	#NAME?	5.00E-05	0.012978	AABR07057848.1	
TCONS_00243282	XLOC_131468	7	99274194	99274809	Inf	5.00E-05	0.012978		
TCONS_00243284	XLOC_131470	7	99275866	99276062	Inf	5.00E-05	0.012978		
TCONS_00243502	XLOC_131671	7	104500670	104501007	-5.23472	5.00E-05	0.012978	RGD1359449	
TCONS_00243628	XLOC_131793	7	109211301	109212409	Inf	5.00E-05	0.012978	Zfat	Transcription
TCONS_00243883	XLOC_132045	7	113605724	113606605	Inf	5.00E-05	0.012978		
TCONS_00244216	XLOC_132377	7	119883647	119887165	-4.21958	5.00E-05	0.012978	Elfn2	Receptor
TCONS_00244362	XLOC_132523	7	121926271	121927312	Inf	5.00E-05	0.012978	Fam83f;Tnrc6b	Apoptosis
TCONS_00244641	XLOC_132789	7	129634630	129634792	#NAME?	5.00E-05	0.012978		
TCONS_00244648	XLOC_132796	7	129740657	129741451	Inf	5.00E-05	0.012978	Zbed4	Transcription
TCONS_00244862	XLOC_133004	7	135972125	135972773	Inf	5.00E-05	0.012978		
TCONS_00244878	XLOC_133016	7	136003479	136004491	Inf	5.00E-05	0.012978		
TCONS_00245027	XLOC_133161	7	138205404	138206574	Inf	5.00E-05	0.012978		
TCONS_00245030	XLOC_133164	7	138211874	138213591	Inf	5.00E-05	0.012978		
TCONS_00245231	XLOC_133361	7	142104481	142104995	Inf	5.00E-05	0.012978	Letmd1	Receptor
TCONS_00245250	XLOC_133379	7	142471726	142472753	Inf	5.00E-05	0.012978	Slc4a8	Transport
TCONS_00245358	XLOC_133486	7	144395605	144396792	Inf	5.00E-05	0.012978		
TCONS_00245359	XLOC_133487	7	144396937	144397971	Inf	5.00E-05	0.012978		
TCONS_00245737	XLOC_133686	8	17219722	17220416	#NAME?	5.00E-05	0.012978		

TCONS_00246004	XLOC_133762	8	22918698	22959554	-4.63448	5.00E-05	0.012978	Rab3d;Tmem205;Ccdc159;Plppr2;Swsap1	Signaling;Unknown
TCONS_00246836	XLOC_134000	8	52821955	52832275	-4.73945	5.00E-05	0.012978	Rexo2;Rbm7;RGD1563941	Translation;Transcription
TCONS_00249857	XLOC_134564	8	127255140	127256465	#NAME?	5.00E-05	0.012978	Golga4	Binding Protein
TCONS_00251476	XLOC_134912	8	41442898	41445722	#NAME?	5.00E-05	0.012978	LOC171573	Unknown
TCONS_00252318	XLOC_135126	8	62563255	62563679	Inf	5.00E-05	0.012978	AABR07070270.1;Arid3b	Transcription
TCONS_00254802	XLOC_135674	8	1304709	1305266	#NAME?	5.00E-05	0.012978		
TCONS_00255029	XLOC_135901	8	11161484	11161565	#NAME?	5.00E-05	0.012978		
TCONS_00255096	XLOC_135968	8	12265907	12265983	#NAME?	5.00E-05	0.012978		
TCONS_00255181	XLOC_136053	8	12780547	12781183	Inf	5.00E-05	0.012978		
TCONS_00255305	XLOC_136175	8	16721498	16721572	#NAME?	5.00E-05	0.012978	SNORA44	
TCONS_00255335	XLOC_136205	8	17700357	17700433	#NAME?	5.00E-05	0.012978	AABR07069336.1;SNORA2	
TCONS_00255477	XLOC_136347	8	21396157	21397051	Inf	5.00E-05	0.012978		
TCONS_00255704	XLOC_136574	8	28232796	28232918	#NAME?	5.00E-05	0.012978		
TCONS_00256095	XLOC_136965	8	37073561	37074626	Inf	5.00E-05	0.012978	LOC100362078;Pate-f	
TCONS_00256124	XLOC_136994	8	37146095	37146982	Inf	5.00E-05	0.012978	Gm17689	
TCONS_00256474	XLOC_137342	8	46496840	46496916	#NAME?	5.00E-05	0.012978	AABR07073391.1	
TCONS_00256796	XLOC_137640	8	52650931	52651817	Inf	5.00E-05	0.012978		
TCONS_00256968	XLOC_137810	8	55701868	55703459	Inf	5.00E-05	0.012978	RGD1562914	
TCONS_00257791	XLOC_138558	8	70284692	70284767	#NAME?	5.00E-05	0.012978		
TCONS_00258093	XLOC_138836	8	74277355	74277544	#NAME?	5.00E-05	0.012978	AABR07070532.1	
TCONS_00258119	XLOC_138862	8	75107443	75108332	Inf	5.00E-05	0.012978		
TCONS_00258184	XLOC_138927	8	75495511	75496855	Inf	5.00E-05	0.012978		
TCONS_00258820	XLOC_139563	8	90676594	90677635	Inf	5.00E-05	0.012978		
TCONS_00258942	XLOC_139685	8	93877488	93877607	#NAME?	5.00E-05	0.012978		
TCONS_00259329	XLOC_140052	8	104223611	104224728	-5.53834	5.00E-05	0.012978	Atp1b3	Metabolism
TCONS_00259715	XLOC_140434	8	111408511	111409122	Inf	5.00E-05	0.012978		
TCONS_00260071	XLOC_140786	8	119277652	119278603	Inf	5.00E-05	0.012978	RGD1565653	
TCONS_00260432	XLOC_141132	8	130629494	130630266	Inf	5.00E-05	0.012978		
TCONS_00260828	XLOC_141332	9	10535339	10536528	-5.3584	5.00E-05	0.012978	Znrf4	
TCONS_00261418	XLOC_141475	9	24093987	24103247	-4.6249	5.00E-05	0.012978	Crisp3;Pgk2	Signaling
TCONS_00261520	XLOC_141514	9	32644381	32644870	#NAME?	5.00E-05	0.012978		
TCONS_00261547	XLOC_141532	9	37462692	37490108	#NAME?	5.00E-05	0.012978	Lgsn	Metabolism
TCONS_00263315	XLOC_141897	9	93445001	93446420	-4.45233	5.00E-05	0.012978	LOC501180;AC112440.1	
TCONS_00264982	XLOC_142276	9	25876251	25876440	#NAME?	5.00E-05	0.012978	LOC108351902	
TCONS_00267598	XLOC_142966	9	2779038	2779133	#NAME?	5.00E-05	0.012978		

TCONS_00267693	XLOC_143061	9	5874880	5875146	#NAME?	5.00E-05	0.012978		
TCONS_00267710	XLOC_143078	9	6490214	6490364	#NAME?	5.00E-05	0.012978		
TCONS_00267829	XLOC_143194	9	8972996	8973378	Inf	5.00E-05	0.012978		
TCONS_00268698	XLOC_144023	9	24558305	24559367	Inf	5.00E-05	0.012978		
TCONS_00268890	XLOC_144215	9	26035606	26036693	Inf	5.00E-05	0.012978		
TCONS_00269164	XLOC_144489	9	33688651	33688727	#NAME?	5.00E-05	0.012978		
TCONS_00269173	XLOC_144498	9	34115683	34115759	#NAME?	5.00E-05	0.012978		
TCONS_00269436	XLOC_144761	9	41151278	41151455	#NAME?	5.00E-05	0.012978		
TCONS_00269498	XLOC_144789	9	41471110	41471500	Inf	5.00E-05	0.012978		
TCONS_00269507	XLOC_144798	9	41544063	41544472	Inf	5.00E-05	0.012978		
TCONS_00269517	XLOC_144807	9	41642854	41643710	Inf	5.00E-05	0.012978		
TCONS_00269764	XLOC_145041	9	48817301	48817377	#NAME?	5.00E-05	0.012978		
TCONS_00269836	XLOC_145113	9	49530234	49530338	#NAME?	5.00E-05	0.012978		
TCONS_00269888	XLOC_145165	9	51763033	51763565	#NAME?	5.00E-05	0.012978		
TCONS_00269894	XLOC_145171	9	51765761	51765939	#NAME?	5.00E-05	0.012978		
TCONS_00269958	XLOC_145235	9	52345832	52346931	#NAME?	5.00E-05	0.012978		
TCONS_00270134	XLOC_145411	9	55143070	55143137	#NAME?	5.00E-05	0.012978		
TCONS_00270138	XLOC_145415	9	55326859	55326935	#NAME?	5.00E-05	0.012978		
TCONS_00270156	XLOC_145433	9	56057911	56057987	#NAME?	5.00E-05	0.012978		
TCONS_00270337	XLOC_145613	9	64172829	64172909	#NAME?	5.00E-05	0.012978		
TCONS_00270645	XLOC_145919	9	71659475	71659552	#NAME?	5.00E-05	0.012978	Plekhm3	Signaling
TCONS_00271029	XLOC_146301	9	79575283	79576060	Inf	5.00E-05	0.012978	U2	
TCONS_00271061	XLOC_146333	9	80331200	80331275	#NAME?	5.00E-05	0.012978		
TCONS_00272091	XLOC_147360	9	102981400	102981475	#NAME?	5.00E-05	0.012978		
TCONS_00272375	XLOC_147644	9	108805804	108805898	#NAME?	5.00E-05	0.012978		
TCONS_00272484	XLOC_147731	9	110478350	110479497	Inf	5.00E-05	0.012978		
TCONS_00272855	XLOC_148098	9	120686014	120686090	#NAME?	5.00E-05	0.012978		
TCONS_00274830	XLOC_148923	X	96528835	96532588	-5.70537	5.00E-05	0.012978	RGD1563104	
TCONS_00277410	XLOC_149752	X	82839764	82846307	-5.60439	5.00E-05	0.012978	AABR07039697.1	
TCONS_00277446	XLOC_149770	X	96458909	96460226	-5.02881	5.00E-05	0.012978	RGD1562485;SNORA17;AABR07040252.1	
TCONS_00277507	XLOC_149798	X	105292337	105308554	#NAME?	5.00E-05	0.012978	Taf7l;Drp2	Transcription;Cytoskeleton
TCONS_00278350	XLOC_150070	X	157319045	157331204	5.46322	5.00E-05	0.012978	Atp2b3;Bgn	Transport;Receptor
TCONS_00278446	XLOC_150102	X	658504	659191	Inf	5.00E-05	0.012978		
TCONS_00278550	XLOC_150205	X	2004584	2005480	Inf	5.00E-05	0.012978		
TCONS_00278694	XLOC_150336	X	9752269	9752416	#NAME?	5.00E-05	0.012978		
TCONS_00278737	XLOC_150379	X	10941009	10941655	Inf	5.00E-05	0.012978	Rn60_X_0110.2;Rn60_X_0110.1	
TCONS_00278885	XLOC_150527	X	13075131	13075675	#NAME?	5.00E-05	0.012978		
TCONS_00279225	XLOC_150858	X	19705640	19705953	#NAME?	5.00E-05	0.012978		
TCONS_00279413	XLOC_151046	X	22402203	22403538	Inf	5.00E-05	0.012978	Kantr;Tspyl2	Protein Binding
TCONS_00279751	XLOC_151369	X	29498584	29498659	#NAME?	5.00E-05	0.012978	Egfl6;Tceanc	Signaling;Transcription

TCONS_00279838	XLOC_151421	X	31186424	31187152	Inf	5.00E-05	0.012978	Mospd2	Development
TCONS_00280297	XLOC_151880	X	34987789	34988100	#NAME?	5.00E-05	0.012978		
TCONS_00280335	XLOC_151918	X	36805396	36805948	#NAME?	5.00E-05	0.012978		
TCONS_00280355	XLOC_151938	X	37113599	37114884	Inf	5.00E-05	0.012978		
TCONS_00280464	XLOC_152047	X	41834273	41834453	#NAME?	5.00E-05	0.012978		
TCONS_00280466	XLOC_152049	X	41898741	41898917	#NAME?	5.00E-05	0.012978		
TCONS_00280503	XLOC_152086	X	43729920	43730133	#NAME?	5.00E-05	0.012978		
TCONS_00280652	XLOC_152235	X	47160017	47160140	#NAME?	5.00E-05	0.012978		
TCONS_00280767	XLOC_152350	X	54415406	54415818	Inf	5.00E-05	0.012978	RGD1565785	
TCONS_00281160	XLOC_152743	X	64070686	64071874	Inf	5.00E-05	0.012978		
TCONS_00281666	XLOC_153239	X	72621566	72622877	Inf	5.00E-05	0.012978	Rn50_X_0735.2	
TCONS_00281802	XLOC_153372	X	75626993	75627795	Inf	5.00E-05	0.012978		
TCONS_00281980	XLOC_153543	X	76270708	76271640	Inf	5.00E-05	0.012978		
TCONS_00282036	XLOC_153597	X	76624947	76625024	#NAME?	5.00E-05	0.012978		
TCONS_00282082	XLOC_153643	X	78115096	78115673	Inf	5.00E-05	0.012978		
TCONS_00282094	XLOC_153655	X	78133131	78134172	Inf	5.00E-05	0.012978		
TCONS_00282387	XLOC_153937	X	84521734	84521879	#NAME?	5.00E-05	0.012978	AABR07039773.1	
TCONS_00282388	XLOC_153938	X	84521964	84522241	#NAME?	5.00E-05	0.012978	AABR07039773.1	
TCONS_00282399	XLOC_153949	X	84904925	84905001	#NAME?	5.00E-05	0.012978		
TCONS_00282545	XLOC_154095	X	93015328	93015406	#NAME?	5.00E-05	0.012978		
TCONS_00282799	XLOC_154349	X	100112515	100113065	Inf	5.00E-05	0.012978		
TCONS_00283268	XLOC_154788	X	107592266	107592342	#NAME?	5.00E-05	0.012978	AABR07040686.1	
TCONS_00283732	XLOC_155205	X	114861657	114861876	#NAME?	5.00E-05	0.012978		
TCONS_00283772	XLOC_155245	X	116511146	116511221	#NAME?	5.00E-05	0.012978		
TCONS_00283878	XLOC_155351	X	122006727	122006803	#NAME?	5.00E-05	0.012978		
TCONS_00283916	XLOC_155388	X	123457266	123458182	Inf	5.00E-05	0.012978	RGD1564541	Unknown
TCONS_00283917	XLOC_155389	X	123498896	123500110	Inf	5.00E-05	0.012978	Ube2a;Nkrf	Metabolism;Transcription
TCONS_00283929	XLOC_155401	X	124116790	124116865	#NAME?	5.00E-05	0.012978	AABR07041247.2;AABR07041247.3	
TCONS_00283930	XLOC_155402	X	124135742	124135818	#NAME?	5.00E-05	0.012978		
TCONS_00284049	XLOC_155521	X	128628354	128628898	Inf	5.00E-05	0.012978	Stag2	Transcription
TCONS_00284149	XLOC_155621	X	134818773	134819419	Inf	5.00E-05	0.012978		
TCONS_00284174	XLOC_155646	X	135235947	135236510	Inf	5.00E-05	0.012978	Elf4;Bcorl1	Transcription
TCONS_00284478	XLOC_155950	X	144397483	144397596	#NAME?	5.00E-05	0.012978	U6	
TCONS_00284531	XLOC_156003	X	147307960	147308036	#NAME?	5.00E-05	0.012978		
TCONS_00284630	XLOC_156102	X	152117427	152117503	#NAME?	5.00E-05	0.012978	LOC100362263	
TCONS_00284687	XLOC_156159	X	154836836	154836915	#NAME?	5.00E-05	0.012978	SNORA17	
TCONS_00284755	XLOC_156227	X	155372179	155374127	Inf	5.00E-05	0.012978		
TCONS_00284813	XLOC_156285	X	155528108	155528832	#NAME?	5.00E-05	0.012978		

TCONS_00285113	XLOC_156489	Y	1215553	1216175	Inf	5.00E-05	0.012978	Ddx3;Rn60_Y_0010.2	

Supplemental Table S6B
F3 sncRNA p<1e-04

Identification	Chr	Start	Stop	baseMean	log 2Fold Change	minP	Gene Association	Gene Category
Rattus_norvegicus_chr2.trna7585-GlyCCC:(218004617-	2	198516709	198516780	200.897203	3.37980515	1.72E-05	U1	
Rattus_norvegicus_chr2.trna7579-GlyCCC:(218050757-	2	198562804	198562875	66.4543855	3.86951198	1.90E-06		
Rattus_norvegicus_chr10.trna11254-GlyGCC:(55448680	19	41016835	41016906	1612.89819	4.12179026	3.20E-06	Vac14	Receptor
Rattus_norvegicus_chr13.trna2238-GlyGCC:(94050532-	13	89411405	89411476	1622.68601	4.09453031	2.20E-06	Fcgr2b	Immune
Rattus_norvegicus_chr17.trna1897-GlyGCC:(58619471-	10	55705550	55705621	1608.10313	3.99115524	3.25E-06	Per1;Hes7;Aloxe3	Development; Transcription
Rattus_norvegicus_chr19.trna1527-GlyGCC:(51842838-	17	44846720	44846791	1609.70712	3.80202832	1.21E-05	Hist1h3b;Hist1h2ak;AC114096.2;Olr1654	Epigenetic
Rattus_norvegicus_chr19.trna1528-GlyGCC:(51843600-	17	44846720	44846791	1602.26436	4.04483931	2.37E-06	Hist1h3b;Hist1h2ak;AC114096.2;Olr1654	Epigenetic
Rattus_norvegicus_chr19.trna3490-GlyGCC:(54011276-	19	43186443	43186514	1593.24064	3.97287631	5.10E-06	AC111287.1;Exosc6;Aars	Metabolism;Translation
Rattus_norvegicus_chr3.trna10899-GlyGCC:(48255376-	17	44846720	44846791	1610.4826	4.00868012	2.99E-06	Hist1h3b;Hist1h2ak;AC114096.2;Olr1654	Epigenetic
Rattus_norvegicus_chr13.trna2225-LeuCAG:(93938654-	13	91182925	91183008	189.891044	5.44502039	7.98E-07	LOC100911825;LOC108348047	Receptor
Rattus_norvegicus_chr13.trna2370-LeuCAG:(95701090-	13	91233791	91233874	191.552226	5.75791102	2.45E-07	LOC100911825	Receptor
Rattus_norvegicus_chr13.trna2382-LeuCAG:(95752080-	13	91233791	91233874	191.869958	5.74737536	1.63E-07	LOC100911825	Receptor
Rattus_norvegicus_chr13.trna2365-LeuCAG:(95681267-	13	89467922	89468005	189.891998	6.01486016	3.84E-08		
Rattus_norvegicus_chr1.trna12832-LeuTAG:(197697796	1	190773257	190773339	133.618688	4.04800758	5.89E-06	Eef2k	Signaling
Rattus_norvegicus_chr17.trna1587-SerAGA:(45707665-	17	43851269	43851351	79.0998855	-4.3009319	1.66E-06		
Rattus_norvegicus_chr17.trna3680-SerAGA:(58823759-	17	44646969	44647051	83.4445223	-4.3269586	1.20E-06	Zfp184	Transcription
Rattus_norvegicus_chr10.trna2463-SerAGA:(55360576-	10	55617773	55617855	83.8907412	-4.1242641	4.90E-06	Ctc1;AC129753.2;Aurkb	Signaling
Rattus_norvegicus_chr17.trna1910-SerAGA:(58795943-	5	23358376	23358458	85.5852261	-4.2516808	3.41E-06	AABR07047194.2;MGC94199	EST
Rattus_norvegicus_chr5.trna455-SerAGA:(28086029-28	17	44674536	44674618	81.1042718	-4.2854383	1.41E-06	AABR07027810.2;AABR07027810.3	
Rattus_norvegicus_chr5.trna476-SerAGA:(28872791-28	5	23358376	23358458	82.2253542	-4.0175843	4.83E-06	AABR07047194.2;MGC94199	EST
Rattus_norvegicus_chr17.trna3953-SerTGA:(45691884-	17	43835407	43835489	77.2276456	-4.0452185	1.49E-05	AABR07027754.1	
Rattus_norvegicus_chr17.trna3686-SerTGA:(58801093-	17	44669467	44669549	82.3811732	-3.9058108	2.15E-05	AABR07027810.2;AABR07027810.3	
piR-rno-7167	3	145914122	145914152	8.97275143	-6.0666731	3.72E-06		
piR-rno-10554	12	49067299	49067325	4.29845517	-6.5313022	7.06E-06		
piR-rno-11689	20	6642860	6642890	3.98866653	-7.0272102	2.62E-06	Ppil1;Rn60_20_0067.1	Immune
piR-rno-14708	4	160354176	160354207	3.22670754	-5.5516646	9.96E-05		
piR-rno-16366	10	91536484	91536515	3.60222788	-6.3197117	5.89E-05	AABR07030520.1	
piR-rno-16896	10	15241138	15241166	334.228492	3.94338586	3.85E-05	Mcrip2;Mettl26;Wfikkn1;Rab40c	Signaling
piR-rno-30402	5	91131795	91131814	464.655947	-4.5130528	3.03E-09	pRNA;5_8S_rRNA;AABR07048791.1	
piR-rno-36323	19	26164478	26164507	50.5102845	2.72856404	5.46E-05	Tnpo2;SNORD41;Fbxw9	Metabolism
piR-rno-40353	NA	NA	NA	749.918353	-4.1279187	2.58E-06		

piR-rno-40406	1	11972196	11972224	55.1011726	-3.2858817	1.90E-05	pRNA;AABR07000398.1;5_8S_rRNA;AABR07000402.1;AABR07000404.1	
piR-rno-40407	10	15241137	15241166	14.1625718	-7.6655085	2.91E-10	Mcrip2;Mettl26;Wfikkn1;Rab40c	Signaling
piR-rno-40408	4	118207495	118207525	16.1790208	-6.0637917	1.48E-09	Pcyox1;Tia1	Transcription
piR-rno-40431	5	91133896	91133926	52260.5835	-3.2659573	1.57E-05	pRNA;5_8S_rRNA;AABR07048791.1	
piR-rno-40439	NA	NA	NA	193.323152	-4.2610013	5.96E-07		
piR-rno-40645	NA	NA	NA	19.9160152	-5.6862203	7.06E-08		
piR-rno-41401	1	11972836	11972863	34.3152668	-4.8907189	3.74E-08	pRNA;AABR07000398.1;5_8S_rRNA;AABR07000402.1;AABR07000404.1;LOC100909599	
piR-rno-41402	17	45637304	45637333	31.0664989	-4.9926905	3.75E-09		
piR-rno-41525	5	91134984	91135015	11263.7209	-5.4490735	1.08E-08	pRNA;5_8S_rRNA;AABR07048791.1	
piR-rno-41910	14	46646166	46646196	10030.7755	-5.2710553	1.72E-08	Rn5-8s;AABR07015078.1;AABR07015078.2;AABR07015079.1;AABR07015080.2;LOC257642;AABR07015080.1;AABR07015081.1	
piR-rno-41976	4	24253903	24253931	688.590055	-4.0379004	1.53E-05	Metazoa_SRP;AABR07059563.1	
piR-rno-42375	18	86602304	86602334	14980.4556	-3.7942731	1.21E-05	Dok6	Signaling
piR-rno-42782	13	89403616	89403648	735.071163	3.29430618	7.17E-05	Fcgr2b;Fcgr3a	Immune
piR-rno-49040	4	77879718	77879747	6.6092214	-5.385346	7.93E-06		
piR-rno-49041	3	162914870	162914901	70.1212623	-4.1262494	2.85E-07		
piR-rno-49047	1	262157805	262157835	88.6224337	-5.4213337	6.35E-09		
piR-rno-50516	13	91205685	91205714	19505.4933	-3.6178377	1.01E-06	LOC100911825;LOC108348047	Receptor
piR-rno-50517	13	89442219	89442249	121919.374	-5.3414258	1.31E-09	Fcgr2b	Immune
piR-rno-50518	13	91205685	91205716	177829.826	-3.1931884	2.41E-05	LOC100911825;LOC108348047	Receptor
piR-rno-50564	1	228439662	228439691	27.3488844	-3.9047007	3.03E-07		
piR-rno-50565	10	55711679	55711709	28.7940955	-3.5287841	2.75E-06	Hes7;Aloxe3	Transcription
piR-rno-50566	1	228439662	228439693	256.473201	-5.708103	1.37E-11		
piR-rno-50567	10	55711677	55711709	7601.66994	-4.2902126	3.32E-08	Hes7;Aloxe3	Transcription
piR-rno-50579	7	62174582	62174610	8.29488877	-4.6596738	1.81E-06	Metazoa_SRP;AABR07057233.1	
piR-rno-50642	NA	NA	NA	963.348254	-4.0583536	5.72E-06		
piR-rno-50643	NA	NA	NA	194.871661	-4.5015868	1.73E-07		
piR-rno-50785	15	28015588	28015617	7.54590621	-3.3899742	2.72E-05	Rnase4	Translation
piR-rno-50843	X	107253122	107253152	203.382447	-3.4097027	1.23E-05		
piR-rno-50971	2	78170312	78170342	5.95426343	-5.0490129	2.57E-05	LOC103689968;Metazoa_SRP	
piR-rno-51043	3	106628868	106628899	5089.05836	-4.0082108	9.55E-07		
piR-rno-52964	16	9774416	9774447	20.8761181	-4.4719902	2.11E-05	AABR07024637.1	
piR-rno-57464	NA	NA	NA	2.60795838	-6.5276711	4.25E-05		
piR-rno-58662	1	11967149	11967179	2626.71135	4.89671607	1.70E-06	pRNA;AABR07000398.1;5_8S_rRNA;AABR07000402.1;AABR07000404.1	
piR-rno-62721	8	28280824	28280844	9.03384226	-5.3039344	7.64E-07		
piR-rno-62725	18	27933530	27933557	15.6453966	-4.6189953	1.71E-05	Ctnna1;U6	Signaling
piR-rno-62735	10	89570398	89570417	47.8636187	-4.9194227	5.36E-08	U2;Arl4d	Translation
piR-rno-62736	9	79592783	79592803	36.9620577	-4.9278249	3.85E-09	Pecr;U2	Metabolism
piR-rno-62740	10	89570394	89570417	88.8011724	-3.5738011	5.58E-05	U2;Arl4d	Translation
piR-rno-62762	2	38121642	38121664	168.00359	-3.7154601	5.59E-06	Dimt1	
piR-rno-62764	2	198638949	198638968	1304.36664	-5.8450472	1.22E-09		
piR-rno-62774	5	143998504	143998534	112765.102	-5.446124	1.00E-07		
piR-rno-62781	1	115716371	115716391	494.566869	-7.8115358	3.77E-13		
piR-rno-62793	6	30640778	30640800	165.980757	-4.0820049	1.63E-06	5_8S_rRNA;AABR07063421.1;AABR07063424.1;LOC257642;AABR07063425.2;AABR07063425.1	

piR-rno-62796	14	46647176	46647201	47.851776	-3.4314862	8.89E-06	Rn5-8s;AABR07015078.1;AABR07015078.2;AABR07015079.1;AABR07015080.2;LOC257642;AABR07015080.1;AABR07015081.1	
piR-rno-62813	1	11974004	11974030	954.748932	-4.5180004	3.55E-07	AABR07000398.1;5_8S_rRNA;AABR07000402.1;AABR07000404.1;LOC100909599	
piR-rno-62815	9	112905938	112905962	2956.25421	-3.5103611	1.38E-06	5S_rRNA	
piR-rno-62840	18	27366739	27366763	79.1772514	-3.2915413	4.53E-05	Nme5;LOC100359679	Signaling
piR-rno-62852	14	46640351	46640373	113.97827	-3.2548348	2.39E-06	pRNA;Rn5-8s;AABR07015078.1;AABR07015078.2	
piR-rno-62855	16	68296293	68296315	122.723769	-3.1240736	1.58E-05		
piR-rno-62883	14	46646165	46646191	2083.28244	-4.0546494	2.91E-06	Rn5-8s;AABR07015078.1;AABR07015078.2;AABR07015079.1;AABR07015080.2;LOC257642;AABR07015080.1;AABR07015081.1	
piR-rno-62893	7	17460996	17461023	18.6092739	-3.6632056	4.71E-05	U5	
piR-rno-62895	1	11908598	11908625	128.99895	-4.4630434	7.69E-06	5_8S_rRNA;pRNA	
piR-rno-62898	1	11970092	11970117	2054.07613	-4.0502791	3.35E-06	pRNA;AABR07000398.1;5_8S_rRNA;AABR07000402.1;AABR07000404.1	
piR-rno-62912	14	46646165	46646190	1683.32058	-3.0859354	6.94E-05	Rn5-8s;AABR07015078.1;AABR07015078.2;AABR07015079.1;AABR07015080.2;LOC257642;AABR07015080.1;AABR07015081.1	
piR-rno-62917	14	46644121	46644142	98.2012404	-4.0161089	4.59E-07	Rn5-8s;AABR07015078.1;AABR07015078.2;AABR07015079.1;AABR07015080.2;LOC257642	
piR-rno-62920	14	46641789	46641810	834.501708	-6.8762274	1.20E-13	pRNA;Rn5-8s;AABR07015078.1;AABR07015078.2	
ENSRNOT00000088216.1:ncrna:chromosome:Rnor:6.0:17:	17	47856706	47856842	43.6272466	4.48453614	8.67E-05	5S_rRNA	
ENSRNOT00000069393.1:ncrna:chromosome:Rnor:6.0:16:	16	15660279	15660305	6.46930637	-4.9814727	2.43E-06	5S_rRNA	
ENSRNOT00000069362.2:ncrna:chromosome:Rnor:6.0:14:	2	154154678	154154704	14.3673879	-3.5103933	1.29E-05	AABR07010873.1	
ENSRNOT00000053366.1:ncrna:chromosome:Rnor:6.0:11:	11	83746249	83746370	32120.9605	4.28850111	7.20E-06	5S_rRNA	
ENSRNOT00000083849.1:ncrna:chromosome:Rnor:6.0:2:2	2	260115160	260115241	69.1167761	3.58105754	2.66E-05	Msh4;Rabggtb;SNORD45;Acadm	Transcription;Signaling;Metabolism
ENSRNOT00000091126.1:ncrna:chromosome:Rnor:6.0:20:	20	4872068	4872134	411.377764	3.44180206	8.35E-05	RT1-CE1;RT1-CE4;RT1-CE7;AABR07044408.1;Ltb;AABR07044407.1;LOC102549726;SNORD52;SNORD48;Hspa1b	Immune;Signaling;Protein Binding
ENSRNOT00000091391.1:ncrna:chromosome:Rnor:6.0:18:	18	70970844	70970909	309.047495	3.3208917	4.17E-05	SNORD58;Rpl17;RGD1562987	Translation;EST
ENSRNOT00000083652.1:ncrna:chromosome:Rnor:6.0:1:2	1	22758917	22758991	18.6404372	4.98315081	8.44E-05	LOC100910237;Rps12;SNORD101;SNORD100;SNORA33	Translation
ENSRNOT00000078790.1:ncrna:chromosome:Rnor:6.0:20:	20	4812351	4812426	72.0629558	4.15280601	7.65E-06	RT1-CE1;RT1-CE4;RT1-CE7;Ddx39b;AABR07044405.1;SNORD83;Atp6v1g2	Immune;Transport
ENSRNOT00000089240.1:ncrna:chromosome:Rnor:6.0:5:1	5	136023193	136023272	399.458037	3.64676636	1.79E-07	Best4;Rps8;SNORD38;SNORD46;SNORD39;Kif2c	Transport;Transcription;Cytoskeleton

ENSRNOT00000083337.1:ncrna:chromosome:Rnor:6.0:8:6	8	69123700	69123799	56.2059016	4.78796351	3.94E-05	Zwilch;Rpl4;SNORD18;SNORD16;snoU18;Snapc5	Unknown
ENSRNOT00000090754.1:ncrna:chromosome:Rnor:6.0:8:4	8	44992058	44992144	49.7736169	4.09369771	1.05E-05	Hspa8;SNORD14	Protein Binding
ENSRNOT00000080370.1:ncrna:chromosome:Rnor:6.0:15:	15	57894100	57894230	203.797844	5.8321033	1.47E-08	Tpt1;SNORA31	
ENSRNOT00000083320.1:ncrna:chromosome:Rnor:6.0:18:	18	70970450	70970516	56.1589747	4.42504712	4.65E-05	Slc25a6;SNORD58;Rpl17;RGD1562987	Transport;Translation;EST
ENSRNOT00000089900.1:ncrna:chromosome:Rnor:6.0:7:2	7	2506334	2506406	28.6063691	4.2872093	2.27E-06	Ptges3;Atp5b;SNORD59	Transport
ENSRNOT00000080140.1:ncrna:chromosome:Rnor:6.0:10:	10	13893646	13893729	170.481706	4.81960749	8.96E-08	Traf7;Rab26;SNORD60	Signaling
ENSRNOT00000078900.1:ncrna:chromosome:Rnor:6.0:18:	18	70971277	70971342	740.944852	2.90802775	7.56E-05	SNORD58;Rpl17;RGD1562987	Translation;EST
ENSRNOT00000079066.1:ncrna:chromosome:Rnor:6.0:1:1	1	164436586	164436731	297.890359	4.12894738	2.87E-05	Klhl35;Rps3;SNORD15	Translation
ENSRNOT00000086454.1:ncrna:chromosome:Rnor:6.0:11:	11	67277456	67277537	57.4142755	5.43235519	5.69E-07	SNORD29	
ENSRNOT00000044320.3:ncrna:chromosome:Rnor:6.0:MT:	MT	5010	5079	35.3256941	4.6904578	9.22E-05	AY172581.13;AY172581.9;AY172581.3;AY172581.24;AY172581.14;Mt-nd1;AY172581.4;AY172581.21;AY172581.15;Mt-nd2;AY172581.6;AY172581.22;AY172581.18;AY172581.10;AY172581.7;Mt-co1;AY172581.19;AY172581.12;Mt-co2;AY172581.1;Mt-atp8;Mt-atp6;Mt-cox3;AY172581.5;Mt-nd3;AY172581.16;Mt-nd4l;Mt-nd4;AY172581.23;AY172581.17;AY172581.11;Mt-nd5;Mt-nd6;AY172581.20;Mt-cyb	
ENSRNOT00000041978.3:ncrna:chromosome:Rnor:6.0:MT:	MT	7693	7757	40.4690425	5.1752012	5.47E-06	AY172581.13;AY172581.9;AY172581.3;AY172581.24;AY172581.14;Mt-nd1;AY172581.4;AY172581.21;AY172581.15;Mt-nd2;AY172581.6;AY172581.22;AY172581.18;AY172581.10;AY172581.7;Mt-co1;AY172581.19;AY172581.12;Mt-co2;AY172581.1;Mt-atp8;Mt-atp6;Mt-cox3;AY172581.5;Mt-nd3;AY172581.16;Mt-nd4l;Mt-nd4;AY172581.23;AY172581.17;AY172581.11;Mt-nd5;Mt-nd6;AY172581.20;Mt-cyb;AY172581.8;AY172581.2	
