# Supplementary material for: Alterations in sperm DNA methylation, non-coding RNA and histone retention associate with DDT-induced epigenetic transgenerational inheritance of disease
Source: Epigenetics Chromatin. 2018 Feb 27;11:8. doi: 10.1186/s13072-018-0178-0 (PMC5827984; doi:10.1186/s13072-018-0178-0)
Supplement: Supplementary file 9 — Additional file 9: Table S7. F3 DHR p < 1e−06. [file 13072_2018_178_MOESM9_ESM.pdf]

Supplemental Table S7
F3 DHR p<1e-06

DHR Name	Chr	Start	(bp) Length	# SigWin	minP	Gene Association	Gene Category
DHR1:6643501	1	6643501	300	1	2.78E-07	Utrn	Cytoskeleton
DHR1:9711601	1	9711601	200	1	2.11E-07		
DHR1:10515601	1	10515601	1200	1	7.48E-07		
DHR1:11490301	1	11490301	5300	1	4.24E-07		
DHR1:12159401	1	12159401	900	1	9.63E-07		
DHR1:12394401	1	12394401	500	1	2.13E-07	AABR07000433.1	
DHR1:13037401	1	13037401	600	1	1.37E-07	Heca	
DHR1:16313601	1	16313601	2200	1	2.00E-07		
DHR1:21653101	1	21653101	800	1	3.40E-10	Enpp3	
DHR1:22950601	1	22950601	700	1	7.77E-07		
DHR1:36161401	1	36161401	2400	2	3.04E-07		
DHR1:48345201	1	48345201	2200	1	3.46E-07	Slc22a2	Transport
DHR1:50349201	1	50349201	1600	1	9.59E-07		
DHR1:52899001	1	52899001	4400	1	9.12E-08	T;T2	Transcription
DHR1:55322201	1	55322201	1200	1	2.07E-07	AABR07001700.1	
DHR1:58780701	1	58780701	300	1	7.82E-07	U6	
DHR1:59562601	1	59562601	100	1	4.05E-07		
DHR1:63622201	1	63622201	2800	1	4.98E-08		
DHR1:64723301	1	64723301	800	1	5.03E-08		
DHR1:68223201	1	68223201	100	1	2.42E-07	Vom1r29	Receptor
DHR1:70081001	1	70081001	2700	1	5.07E-07	AABR07002258.1	
DHR1:70250101	1	70250101	3000	1	4.72E-07	Zim1	
DHR1:71924101	1	71924101	5300	1	1.96E-07	AABR07002353.2	
DHR1:74435301	1	74435301	900	1	9.18E-08		
DHR1:81651401	1	81651401	700	2	3.19E-07	LOC103689942	
DHR1:90596301	1	90596301	2900	1	8.37E-07	Chst8	Metabolism
DHR1:94639901	1	94639901	3200	1	1.38E-09		
DHR1:96031701	1	96031701	100	1	7.87E-09		
DHR1:101245501	1	101245501	2400	1	4.53E-07	Cd37;Slc6a16	Immune;Transport
DHR1:109354801	1	109354801	1900	1	4.49E-07		
DHR1:110940801	1	110940801	2400	1	1.54E-08		
DHR1:113218201	1	113218201	200	1	8.24E-07	Luzp2;Gabrb3	Receptor
DHR1:121029301	1	121029301	900	1	6.90E-07		
DHR1:121353901	1	121353901	1200	1	6.30E-08		
DHR1:128349801	1	128349801	2000	1	2.87E-07	Mef2a	Transcription
DHR1:134046001	1	134046001	600	1	4.97E-07		
DHR1:142319901	1	142319901	200	1	3.71E-07	Blm	Transcription
DHR1:147399701	1	147399701	1800	1	9.84E-07	U6	
DHR1:155276801	1	155276801	700	1	7.43E-07	AABR07004765.1	
DHR1:158604501	1	158604501	500	1	1.55E-08		
DHR1:158843201	1	158843201	1900	1	5.07E-07		
DHR1:159332701	1	159332701	1900	1	9.46E-07		
DHR1:163380001	1	163380001	1400	1	4.65E-07		
DHR1:173807501	1	173807501	1200	2	8.89E-07		
DHR1:176306601	1	176306601	4800	1	2.85E-07	Galnt18	Unknown
DHR1:191840601	1	191840601	2100	1	5.36E-09	Scnn1b	Metabolism

DHR1:201391601	1	201391601	3500	1	1.17E-07	Btbd16	
DHR1:201858601	1	201858601	2000	1	4.67E-07	AC123083.1	
DHR1:204320601	1	204320601	6500	1	2.69E-07	Chst15;AABR07005888.2	Metabolism
DHR1:206345301	1	206345301	300	1	8.99E-07	LOC100302465	EST
DHR1:219936901	1	219936901	2500	1	2.89E-07		
DHR1:222007001	1	222007001	2000	1	5.16E-07		
DHR1:230681901	1	230681901	300	1	1.78E-07	Olr379;Olr380	Receptor
DHR1:236843501	1	236843501	2200	1	5.50E-07		
DHR1:270940501	1	270940501	1600	1	1.15E-07		
DHR1:273529501	1	273529501	3100	1	2.97E-07		
DHR1:273815101	1	273815101	4500	1	6.56E-07		
DHR1:274030901	1	274030901	16300	1	2.15E-08	Mxi1	Transcription
DHR1:277953101	1	277953101	3200	1	5.85E-07	Ablim1	Cytoskeleton
DHR1:280120201	1	280120201	2000	1	5.71E-07	Eno4;Shtn1	
DHR1:281382701	1	281382701	1200	1	6.74E-07	Fam204a	
DHR2:239601	2	239601	2400	1	7.92E-07		
DHR2:857901	2	857901	9900	1	5.77E-07		
DHR2:1115601	2	1115601	3500	1	5.73E-07		
DHR2:3828301	2	3828301	700	1	4.49E-07	Mctp1	Unknown
DHR2:5692601	2	5692601	800	2	2.00E-07		
DHR2:7044201	2	7044201	2000	1	8.06E-07		
DHR2:7709401	2	7709401	100	1	6.14E-08		
DHR2:9123401	2	9123401	200	1	3.27E-07	Adgrv1	
DHR2:13035201	2	13035201	1900	1	2.59E-07		
DHR2:15323101	2	15323101	300	1	8.98E-07		
DHR2:15915901	2	15915901	200	1	3.26E-08		
DHR2:17166201	2	17166201	1700	1	2.04E-08		
DHR2:19538201	2	19538201	100	1	6.33E-08		
DHR2:25594001	2	25594001	2100	1	2.78E-07		
DHR2:28168301	2	28168301	1200	1	3.01E-07		
DHR2:29361401	2	29361401	1200	1	4.59E-07		
DHR2:39214301	2	39214301	2300	1	5.91E-07		
DHR2:42357501	2	42357501	200	1	1.20E-07		
DHR2:43010001	2	43010001	400	1	8.07E-07	Gpbp1	
DHR2:43622601	2	43622601	1400	1	8.00E-07		
DHR2:45199501	2	45199501	600	1	8.21E-08	RGD1561161	Unknown
DHR2:49251601	2	49251601	100	1	1.70E-07		
DHR2:49894601	2	49894601	1200	1	2.03E-07	AABR07008259.1	
DHR2:53521701	2	53521701	1200	1	1.56E-07		
DHR2:55287301	2	55287301	1200	1	4.44E-08		
DHR2:58771201	2	58771201	900	1	4.92E-07		
DHR2:62447001	2	62447001	3800	1	2.70E-09	Pdzd2	
DHR2:66543001	2	66543001	3000	1	1.84E-07		
DHR2:71933501	2	71933501	100	1	9.09E-09		
DHR2:72008801	2	72008801	200	1	6.48E-07	Cdh12	Extracellular Matrix
DHR2:74081101	2	74081101	300	1	1.40E-07		
DHR2:74263301	2	74263301	1100	1	1.43E-07		
DHR2:78279201	2	78279201	100	1	9.42E-07	LOC103689968;AABR07008911.1	

DHR2:81318001	2	81318001	3400	1	6.20E-07		
DHR2:83169101	2	83169101	2200	2	1.07E-07		
DHR2:94166001	2	94166001	1000	1	3.58E-07		
DHR2:101628501	2	101628501	1600	1	3.77E-07		
DHR2:103741501	2	103741501	500	1	1.77E-07	AABR07009572.1	
DHR2:106466901	2	106466901	1500	1	1.36E-07		
DHR2:108323601	2	108323601	100	1	3.79E-07		
DHR2:114419801	2	114419801	400	1	8.96E-07	Slc2a2	Transport
DHR2:114565501	2	114565501	100	1	2.27E-10		
DHR2:117804101	2	117804101	3200	1	2.39E-07		
DHR2:120117001	2	120117001	800	1	4.10E-08		
DHR2:123549901	2	123549901	3000	1	4.93E-07	RGD1307100	
DHR2:132344601	2	132344601	1400	1	9.09E-08	AABR07010330.1;AABR07010330.2	
DHR2:140549101	2	140549101	2900	1	8.79E-07	Rab33b	Signaling
DHR2:142820701	2	142820701	500	1	2.09E-07	Frem2	Cytoskeleton
DHR2:143971901	2	143971901	3800	1	9.74E-08	LOC103691556	
DHR2:146697701	2	146697701	1100	1	4.21E-07		
DHR2:158356601	2	158356601	1600	1	4.33E-07		
DHR2:180290901	2	180290901	1200	1	1.27E-07		
DHR2:184494601	2	184494601	1000	1	3.76E-07		
DHR2:185548901	2	185548901	200	1	1.60E-07	Lrba	Signaling
DHR2:186018001	2	186018001	3000	1	9.34E-07	Lrba	Signaling
DHR2:186666301	2	186666301	4300	1	6.46E-07	Fcrl1	Receptor
DHR2:190629101	2	190629101	300	1	2.35E-08	S100vp	
DHR2:191716501	2	191716501	2400	1	8.64E-07		
DHR2:195349901	2	195349901	300	1	4.24E-08		
DHR2:195764201	2	195764201	2900	1	3.22E-07	Snx27	Endocytosis
DHR2:201110301	2	201110301	2200	1	2.21E-07		
DHR2:205288801	2	205288801	800	1	7.90E-07	Sycp1	Cell Cycle
DHR2:207706801	2	207706801	1100	1	8.70E-07		
DHR2:207933401	2	207933401	400	1	9.18E-07	Kcnd3	Transport
DHR2:208004201	2	208004201	700	1	4.83E-07	Kcnd3	Transport
DHR2:211991601	2	211991601	300	1	6.16E-08		
DHR2:214951101	2	214951101	300	1	4.56E-07		
DHR2:216452201	2	216452201	1900	2	1.07E-07	Amy1a	Metabolism
DHR2:217427001	2	217427001	1700	1	6.47E-07		
DHR2:217801901	2	217801901	200	1	4.96E-07		
DHR2:225357401	2	225357401	1300	1	1.20E-07	Abcd3	Transport
DHR2:227431401	2	227431401	600	1	1.74E-07		
DHR2:230310501	2	230310501	1400	1	1.44E-07	Sec24b	Transport
DHR2:231108801	2	231108801	900	1	1.62E-07	Camk2d	Signaling
DHR2:241524301	2	241524301	2100	1	9.19E-07	Bank1	Development
DHR2:241727801	2	241727801	100	1	1.12E-07		
DHR2:244191101	2	244191101	800	1	6.49E-07	Tspan5	Cytoskeleton
DHR2:245868101	2	245868101	1200	1	2.78E-07		
DHR2:246002201	2	246002201	3500	1	6.68E-07		
DHR2:246412701	2	246412701	100	1	2.08E-07		
DHR2:247546601	2	247546601	1200	1	2.06E-07		
DHR2:250290501	2	250290501	1100	1	1.16E-07		

DHR2:253157501	2	253157501	1800	1	2.36E-07		
DHR2:259147701	2	259147701	3300	1	7.90E-07	AABR07013843.1	
DHR2:260751601	2	260751601	4400	1	1.81E-08		
DHR2:265256701	2	265256701	1500	1	3.43E-07	Lrrc7	Unknown
DHR3:44001	3	44001	1900	1	4.89E-07		
DHR3:8080901	3	8080901	3600	1	9.93E-07		
DHR3:8833201	3	8833201	800	2	5.72E-07	Lrrc8a;Phyhd1	Unknown;Metabolism
DHR3:17906601	3	17906601	800	1	1.80E-07		
DHR3:30810301	3	30810301	200	1	6.01E-07		
DHR3:39581501	3	39581501	4100	1	8.31E-07	Rprm	Transcription
DHR3:44325501	3	44325501	200	1	3.03E-07	Acvr1c	
DHR3:47525801	3	47525801	1300	1	1.99E-07		
DHR3:48801301	3	48801301	3400	1	4.84E-07	Kcnh7	Metabolism
DHR3:52348601	3	52348601	2400	1	3.39E-08	Ttc21b	Unknown
DHR3:58937901	3	58937901	1400	1	8.52E-07		
DHR3:63528801	3	63528801	400	1	7.68E-07	AABR07052585.3;Fkbp7;Plekha3	Protein Binding;Transcription
DHR3:65017901	3	65017901	800	1	2.96E-07	AABR07052598.1	
DHR3:70634901	3	70634901	3000	1	3.93E-08		
DHR3:70921401	3	70921401	3100	1	3.57E-07		
DHR3:72684101	3	72684101	2300	1	1.37E-07		
DHR3:77389001	3	77389001	800	1	1.78E-07	Olr657	
DHR3:86319301	3	86319301	4200	1	5.18E-07	Lrrc4c	Extracellular Matrix
DHR3:90557901	3	90557901	3000	1	6.99E-07		
DHR3:90728601	3	90728601	1400	1	5.58E-07	LOC499843	
DHR3:94050301	3	94050301	300	1	1.20E-07		
DHR3:97191101	3	97191101	300	1	5.55E-07		
DHR3:100281801	3	100281801	800	1	1.97E-08	Mettl15	
DHR3:105336601	3	105336601	1700	1	6.52E-07	AABR07053412.1	
DHR3:105547701	3	105547701	700	1	5.18E-07		
DHR3:108291701	3	108291701	1000	1	1.19E-07		
DHR3:110133701	3	110133701	2100	1	2.32E-07	Gpr176	Receptor
DHR3:119851001	3	119851001	1500	1	3.13E-09		
DHR3:124909601	3	124909601	1400	1	3.41E-07	Cds2	Metabolism
DHR3:126316701	3	126316701	900	1	4.92E-07		
DHR3:132757001	3	132757001	2700	1	8.39E-07		
DHR3:138346701	3	138346701	3500	1	9.31E-07		
DHR3:147218101	3	147218101	4200	1	9.44E-07	Tmem74b	
DHR3:148551901	3	148551901	1800	1	1.76E-07	Ccm2l	
DHR3:156431901	3	156431901	4900	1	9.16E-09	AABR07054488.1	
DHR3:160151701	3	160151701	1500	2	4.99E-08		
DHR3:161334101	3	161334101	1700	2	4.68E-08	Pcif1	Transcription
DHR3:161676101	3	161676101	2000	1	6.60E-07	Cdh22	Extracellular Matrix
DHR3:164487001	3	164487001	33500	1	6.68E-07		
DHR3:166172801	3	166172801	2900	1	6.47E-07	U2	
DHR3:166495001	3	166495001	3700	1	2.29E-07		
DHR3:167141901	3	167141901	2800	1	1.23E-07		
DHR3:168069401	3	168069401	9900	1	5.81E-08		
DHR3:170857901	3	170857901	100	1	2.56E-07		
DHR3:176259501	3	176259501	4300	2	7.64E-07		

DHR4:5650801	4	5650801	2000	1	4.26E-07	Actr3b	Cytoskeleton
DHR4:6117001	4	6117001	1300	1	6.32E-09	Kmt2c	Transcription
DHR4:7631401	4	7631401	1600	1	7.28E-07		
DHR4:13456501	4	13456501	200	1	9.30E-07	Gnai1	Signaling
DHR4:14766101	4	14766101	2300	1	9.63E-07		
DHR4:16600901	4	16600901	200	1	6.47E-07	Pclo	Extracellular Matrix
DHR4:19070601	4	19070601	2000	1	1.83E-07		
DHR4:21950101	4	21950101	600	1	1.12E-07	AABR07059495.1	
DHR4:28460901	4	28460901	800	1	6.03E-08	Vps50	
DHR4:29141001	4	29141001	3100	1	8.46E-08		
DHR4:31033101	4	31033101	2000	2	3.24E-07	Dync1i1	Cytoskeleton
DHR4:32623201	4	32623201	1400	1	5.92E-07		
DHR4:34171801	4	34171801	2600	1	7.29E-07	Col28a1	
DHR4:34600601	4	34600601	100	1	5.08E-07	Glcci1	
DHR4:36260301	4	36260301	800	1	1.66E-07		
DHR4:39200001	4	39200001	200	1	8.55E-07		
DHR4:39269301	4	39269301	3600	1	2.40E-08		
DHR4:43744401	4	43744401	1200	1	4.33E-07		
DHR4:49137901	4	49137901	4300	1	1.23E-07	Cped1	
DHR4:50522001	4	50522001	1100	1	2.21E-07	Cadps2	Metabolism
DHR4:50600801	4	50600801	900	1	7.48E-07	Cadps2	Metabolism
DHR4:67843501	4	67843501	300	1	4.69E-07		
DHR4:72109501	4	72109501	1000	1	7.73E-07		
DHR4:76677201	4	76677201	100	1	6.56E-07	Cntnap2	Receptor
DHR4:82386701	4	82386701	4800	3	5.33E-09	AABR07060588.2	
DHR4:84179701	4	84179701	1100	1	3.86E-07		
DHR4:84620601	4	84620601	3600	1	9.45E-07	Wipf3	Receptor
DHR4:86459701	4	86459701	400	1	6.98E-07	Pde1c	Metabolism
DHR4:86595901	4	86595901	4800	1	2.40E-07	Pde1c	Metabolism
DHR4:93787601	4	93787601	1700	1	9.03E-08	AABR07060788.1	
DHR4:115754601	4	115754601	900	1	8.12E-07	Dysf	Transport
DHR4:123515001	4	123515001	1000	1	7.34E-07	Slc41a3;Aldh1l1	Transport;Metabolism
DHR4:123882101	4	123882101	3100	1	6.96E-07	Fgd5	Signaling
DHR4:135593601	4	135593601	300	1	7.48E-07		
DHR4:143560301	4	143560301	1600	1	3.42E-07		
DHR4:153555201	4	153555201	4300	1	5.23E-07	Mical3	
DHR4:160126001	4	160126001	300	1	2.44E-07		
DHR4:163046901	4	163046901	200	1	1.60E-07	Cd69	Receptor
DHR4:170594001	4	170594001	3300	1	2.42E-07	Plbd1	EST
DHR4:175904101	4	175904101	2300	1	2.17E-07		
DHR4:178135501	4	178135501	2200	1	6.28E-07	Sox5	Transcription
DHR4:179456401	4	179456401	4200	1	8.73E-08	Casc1	
DHR5:861101	5	861101	200	1	2.81E-07		
DHR5:7001001	5	7001001	800	1	6.57E-07	RGD1564053	Unknown
DHR5:7557901	5	7557901	1000	1	3.60E-08		
DHR5:9600301	5	9600301	200	1	4.21E-07		
DHR5:13692201	5	13692201	1400	1	3.38E-07		
DHR5:14845301	5	14845301	1900	1	3.87E-07		
DHR5:34733901	5	34733901	400	1	2.28E-08	Nkain3	Transport
DHR5:35431701	5	35431701	5000	1	4.35E-07		

DHR5:35474401	5	35474401	4000	1	3.01E-07		
DHR5:38694701	5	38694701	4600	1	7.22E-07		
DHR5:42362001	5	42362001	1500	1	6.51E-08		
DHR5:48590901	5	48590901	200	1	2.37E-07	Rngtt	Translation
DHR5:50147801	5	50147801	1700	1	1.48E-07	Slc35a1;Cfap206	Transport
DHR5:52539101	5	52539101	7500	1	4.87E-07		
DHR5:54979201	5	54979201	2200	1	7.69E-07		
DHR5:60328001	5	60328001	100	1	4.73E-07	Zcchc7	Transcription
DHR5:61265801	5	61265801	2600	2	9.95E-08		
DHR5:68530101	5	68530101	200	1	4.24E-07		
DHR5:74139401	5	74139401	1400	1	3.36E-08	Epb41l4b	
DHR5:74217701	5	74217701	1500	1	3.90E-07		
DHR5:75925501	5	75925501	400	1	8.99E-07	RGD1306148	
DHR5:84193601	5	84193601	500	1	3.24E-07		
DHR5:84588401	5	84588401	1600	1	2.87E-07		
DHR5:91400801	5	91400801	3400	1	3.37E-07		
DHR5:94309401	5	94309401	3400	1	9.34E-07		
DHR5:96030801	5	96030801	1300	1	3.37E-07		
DHR5:96323301	5	96323301	3100	1	8.55E-07		
DHR5:100315101	5	100315101	5500	1	2.01E-07		
DHR5:101608001	5	101608001	600	2	1.28E-07	SNORA73	
DHR5:102537801	5	102537801	300	1	2.98E-07	Bnc2	Development
DHR5:110643501	5	110643501	1700	1	1.57E-07		
DHR5:113360001	5	113360001	200	1	5.53E-07		
DHR5:126404301	5	126404301	800	1	7.70E-08	Acot11	Metabolism
DHR5:133465301	5	133465301	600	2	1.54E-09		
DHR5:144009201	5	144009201	1500	1	7.38E-07		
DHR5:145897401	5	145897401	500	1	4.55E-07		
DHR5:148943601	5	148943601	900	1	2.69E-07	Sdc3	Signaling
DHR5:151058301	5	151058301	2700	1	1.31E-07	Stx12	Transport
DHR5:151666101	5	151666101	10600	1	6.92E-08	AC118963.2	
DHR5:153222501	5	153222501	2000	2	3.27E-08	Rhd;Tmem50a	Transport
DHR5:158930401	5	158930401	4500	1	5.94E-07		
DHR5:162178701	5	162178701	1900	1	6.11E-07	Oog1;Pramef8	
DHR5:165566801	5	165566801	3400	1	5.61E-07		
DHR5:169096301	5	169096301	3500	1	4.24E-07	Rn60_5_1692.1	
DHR5:171201001	5	171201001	3000	1	2.40E-07		
DHR5:171384801	5	171384801	19700	1	1.18E-09	Tp73	Transcription
DHR6:172301	6	172301	900	1	2.00E-07		
DHR6:490501	6	490501	300	1	9.39E-08		
DHR6:10329801	6	10329801	200	1	5.41E-07	Epas1	Transcription
DHR6:15111601	6	15111601	5200	1	4.33E-07	Nrxn1	Receptor
DHR6:15340201	6	15340201	2500	1	2.23E-07		
DHR6:21564501	6	21564501	200	1	3.90E-08	Ltbp1	Metabolism
DHR6:23770901	6	23770901	500	1	8.47E-08		
DHR6:25103501	6	25103501	2100	1	2.31E-07	Ehd3	Metabolism
DHR6:28971801	6	28971801	2500	1	1.41E-07	Mfsd2b;Ubxn2a	Proteolysis
DHR6:43945201	6	43945201	1400	1	6.91E-07		
DHR6:51200601	6	51200601	3800	1	5.53E-07	Cog5	Golgi
DHR6:62105601	6	62105601	100	1	1.32E-07		

DHR6:62592801	6	62592801	3100	1	3.36E-07		
DHR6:65733801	6	65733801	8300	1	8.79E-08		
DHR6:70363301	6	70363301	2100	1	4.66E-07		
DHR6:72318401	6	72318401	1600	2	4.59E-08		
DHR6:73672101	6	73672101	1500	1	3.99E-07	Akap6	Signaling
DHR6:75179201	6	75179201	400	1	3.99E-07		
DHR6:78101601	6	78101601	1300	2	2.53E-08		
DHR6:83405401	6	83405401	2100	1	8.65E-07	Lrfn5	Receptor
DHR6:89151101	6	89151101	3600	1	9.93E-07		
DHR6:89774801	6	89774801	4600	1	3.78E-07		
DHR6:89868601	6	89868601	1700	1	9.81E-07		
DHR6:91555601	6	91555601	3100	1	9.30E-07	Klhdc1	
DHR6:94845101	6	94845101	4000	1	6.67E-08	Jkamp;Ccdc175	Receptor
DHR6:96708201	6	96708201	3500	1	7.28E-07		
DHR6:98049901	6	98049901	5400	1	1.38E-07	Kcnh5	Metabolism
DHR6:101727601	6	101727601	2200	1	8.88E-07	Gphn	Receptor
DHR6:116654201	6	116654201	1700	1	1.34E-07	U6	
DHR6:120940301	6	120940301	200	1	1.32E-07		
DHR6:123520801	6	123520801	2400	1	6.81E-07	Foxn3	
DHR6:126191901	6	126191901	1800	1	9.41E-07	Rin3	Signaling
DHR6:130606901	6	130606901	2200	1	7.61E-07		
DHR6:131990001	6	131990001	7100	2	3.57E-07		
DHR6:136791701	6	136791701	13300	1	7.20E-07		
DHR6:138619001	6	138619001	3800	1	1.62E-07	Ighm;AABR07065651.3	
DHR6:139907901	6	139907901	800	1	7.48E-07	AABR07065714.1	
DHR6:140270001	6	140270001	6000	1	9.46E-07	AABR07065753.1	
DHR6:141711001	6	141711001	3300	1	2.71E-07	AABR07065798.1	
DHR6:142976101	6	142976101	4500	1	3.30E-07		
DHR6:147382301	6	147382301	4100	1	8.39E-07	Macc1	
DHR6:147396101	6	147396101	100	1	2.78E-07	Macc1	
DHR6:147439201	6	147439201	1400	1	4.74E-07		
DHR7:3325301	7	3325301	200	1	2.18E-07	Gdf11;Cd63	Signaling
DHR7:11617701	7	11617701	16600	1	9.52E-07	Gng7	Signaling
DHR7:13224401	7	13224401	400	1	1.04E-07		
DHR7:23438401	7	23438401	100	1	4.34E-07	Syn3	Development
DHR7:25712301	7	25712301	1600	1	6.88E-07		
DHR7:29010701	7	29010701	100	1	4.48E-07	Gnptab	Transcription
DHR7:31228101	7	31228101	300	1	5.96E-08	Anks1b	Receptor
DHR7:32959101	7	32959101	4800	1	6.90E-07		
DHR7:36759401	7	36759401	1700	2	1.15E-08		
DHR7:37461001	7	37461001	1100	1	4.50E-07		
DHR7:44520801	7	44520801	2000	1	7.42E-07		
DHR7:49211001	7	49211001	4700	1	9.86E-07	Acss3	Metabolism
DHR7:52267801	7	52267801	1200	1	6.30E-08	Nav3	Development
DHR7:52443801	7	52443801	900	1	6.05E-07		
DHR7:53746401	7	53746401	200	1	7.48E-07		
DHR7:56540001	7	56540001	1200	1	2.13E-07		
DHR7:57831001	7	57831001	700	1	1.32E-07		
DHR7:59744401	7	59744401	500	1	2.24E-07		

DHR7:71273101	7	71273101	3200	1	2.29E-07	Uqcrb;Mterf3	
DHR7:71301701	7	71301701	4800	1	1.08E-07	Mterf3;Ptdss1	Metabolism
DHR7:71625101	7	71625101	2000	1	2.29E-07	Sdc2;Metazoa_SRP;AABR07057443.1	Extracellular Matrix
DHR7:76622801	7	76622801	4200	1	4.26E-07		
DHR7:84776201	7	84776201	3000	1	1.18E-07		
DHR7:86937601	7	86937601	400	1	5.04E-07	Csmd3	
DHR7:87688201	7	87688201	1300	1	2.05E-07	Csmd3	
DHR7:89968601	7	89968601	400	1	7.52E-07		
DHR7:90582201	7	90582201	1300	1	4.66E-07		
DHR7:92097001	7	92097001	800	1	9.03E-07		
DHR7:96702601	7	96702601	1100	1	3.27E-07		
DHR7:97464301	7	97464301	500	1	3.14E-07	AABR07057997.1	
DHR7:101472001	7	101472001	300	1	3.42E-09		
DHR7:101543101	7	101543101	2800	1	7.67E-07		
DHR7:108278101	7	108278101	8500	1	9.01E-08		
DHR7:111449201	7	111449201	2500	1	2.16E-07		
DHR7:124633001	7	124633001	2200	1	4.92E-07	AABR07058554.1	
DHR7:124843801	7	124843801	2800	1	4.14E-07	Efcab6	Signaling
DHR7:128595601	7	128595601	6500	1	1.94E-07	Fam19a5	
DHR7:130209501	7	130209501	1200	1	1.03E-07		
DHR7:134932601	7	134932601	1200	1	9.68E-07		
DHR7:143583801	7	143583801	2800	1	9.72E-07	Rn50_7_1414.2;Krt8	Cytoskeleton
DHR8:1311701	8	1311701	2700	1	6.65E-08		
DHR8:4309601	8	4309601	3300	1	2.81E-07	Vom2r18;Vom2r22	
DHR8:5230501	8	5230501	5300	1	7.45E-07	Dync2h1	Cytoskeleton
DHR8:7993401	8	7993401	1900	1	1.49E-07	Cntn5	Extracellular Matrix
DHR8:8987901	8	8987901	5600	1	2.98E-08		
DHR8:9047101	8	9047101	1000	1	2.33E-07		
DHR8:11488801	8	11488801	600	1	3.87E-07		
DHR8:11868301	8	11868301	1700	1	3.11E-08		
DHR8:15757901	8	15757901	1100	1	5.86E-08		
DHR8:19162401	8	19162401	100	1	1.54E-07	LOC100911893;Olr1138	Receptor
DHR8:22587901	8	22587901	3400	1	6.07E-08	Carm1	Metabolism
DHR8:43252801	8	43252801	1500	1	4.15E-07	Olr1305	
DHR8:46616301	8	46616301	1600	1	7.60E-07	Tecta	Extracellular Matrix
DHR8:56663101	8	56663101	1300	1	1.76E-08	Zc3h12c	Transcription
DHR8:60778601	8	60778601	4900	2	2.29E-07	Pstpip1	Cytoskeleton
DHR8:64951901	8	64951901	3800	1	1.17E-07	Rn50_8_0647.2	
DHR8:69254001	8	69254001	100	1	2.15E-07		
DHR8:80038901	8	80038901	2200	1	1.10E-07		
DHR8:88251201	8	88251201	2700	1	3.65E-07		
DHR8:88431401	8	88431401	2500	1	1.32E-07		
DHR8:88687201	8	88687201	500	1	7.48E-07		
DHR8:95173101	8	95173101	900	1	3.65E-07		
DHR8:97737101	8	97737101	2000	1	7.67E-07		
DHR8:104469801	8	104469801	2700	1	7.96E-07	Rasa2	
DHR8:104686501	8	104686501	4300	1	6.91E-08		
DHR8:111025701	8	111025701	2000	1	3.53E-07	Ky	Proteolysis

DHR8:111095201	8	111095201	1700	1	7.22E-07	Cep63	
DHR8:114940801	8	114940801	8900	1	1.27E-07	Alas1	Metabolism
DHR8:120932601	8	120932601	3600	1	8.42E-07	AABR07073455.1	
DHR8:122155701	8	122155701	800	1	4.23E-07	Clasp2	Unknown
DHR9:2504001	9	2504001	800	2	5.52E-09		
DHR9:11218901	9	11218901	900	1	5.30E-07	Uxs1	Metabolism
DHR9:12850801	9	12850801	1500	1	5.75E-07	Rftn1	Unknown
DHR9:13163401	9	13163401	2200	1	4.71E-07	Kif6	Cytoskeleton
DHR9:14001301	9	14001301	1300	1	8.06E-07		
DHR9:15135801	9	15135801	1100	1	1.38E-07		
DHR9:15574201	9	15574201	8900	1	8.12E-07	RGD1561662;LOC100912849	
DHR9:15615301	9	15615301	3700	1	6.92E-08	U6;Guca1a;Guca1b	Signaling
DHR9:15783301	9	15783301	2200	1	6.23E-07		
DHR9:20592701	9	20592701	1600	1	8.56E-07	Tnfrsf21	Apoptosis
DHR9:21057201	9	21057201	3400	1	1.01E-07	Opn5	Receptor
DHR9:26522201	9	26522201	4600	1	8.55E-07		
DHR9:31754301	9	31754301	2600	1	4.37E-07	Adgrb3	
DHR9:34299801	9	34299801	3100	1	1.30E-07		
DHR9:34649301	9	34649301	4100	1	9.90E-07		
DHR9:34669401	9	34669401	1000	1	1.67E-07		
DHR9:35712001	9	35712001	800	1	7.26E-07		
DHR9:37134001	9	37134001	1100	1	2.71E-07	Phf3	
DHR9:40213501	9	40213501	1900	1	3.71E-07		
DHR9:43299601	9	43299601	400	1	5.06E-07	LOC100909859	
DHR9:43767701	9	43767701	4000	1	2.75E-07	Vwa3b	Development
DHR9:44208501	9	44208501	2400	1	1.74E-07	RGD1310819	Unknown
DHR9:44359301	9	44359301	100	1	2.97E-08	Tsga10	Signaling
DHR9:53080801	9	53080801	600	1	8.03E-07	Ankar;Osgepl1	Proteolysis
DHR9:57043401	9	57043401	3800	1	2.02E-07		
DHR9:58212001	9	58212001	200	1	6.58E-07		
DHR9:60927701	9	60927701	1500	1	1.35E-07	AABR07067749.1;Ccdc150	
DHR9:75915001	9	75915001	200	1	3.81E-07		
DHR9:90706701	9	90706701	2300	1	7.48E-07	AABR07068203.1;LOC108351965	
DHR9:94270601	9	94270601	3000	1	1.62E-07	AC098189.1;Prss56	
DHR9:96037001	9	96037001	400	1	7.52E-07		
DHR9:102759301	9	102759301	5400	1	5.49E-07		
DHR9:104203601	9	104203601	1500	1	7.07E-07		
DHR9:110882001	9	110882001	1600	1	8.42E-07	Fbxl17	Proteolysis
DHR9:112651701	9	112651701	1600	1	2.54E-07		
DHR9:115794301	9	115794301	400	1	1.59E-07	AABR07068709.1	
DHR9:119487201	9	119487201	4100	1	8.17E-07		
DHR10:7925601	10	7925601	300	1	2.90E-07		
DHR10:17789001	10	17789001	1700	2	2.73E-08		
DHR10:19431101	10	19431101	400	1	4.07E-08		
DHR10:25351401	10	25351401	400	1	5.55E-07		
DHR10:30006301	10	30006301	500	1	8.50E-07		
DHR10:30719201	10	30719201	200	1	3.28E-08		

DHR10:33962601	10	33962601	300	1	7.20E-08		
DHR10:41271801	10	41271801	900	1	5.88E-07		
DHR10:52762501	10	52762501	1100	1	5.34E-07	Shisa6	Development
DHR10:53387501	10	53387501	2900	1	6.53E-07		
DHR10:63483601	10	63483601	4900	1	5.43E-07	Tusc5	
DHR10:73607101	10	73607101	1900	1	2.39E-07	Brip1	Transcription
DHR10:74196401	10	74196401	3900	1	2.69E-07		
DHR10:74232901	10	74232901	2900	2	3.13E-08	Ypel2	
DHR10:75310401	10	75310401	3200	1	6.33E-07		
DHR10:76407001	10	76407001	4300	1	9.04E-07	Dgke	Signaling
DHR10:80208101	10	80208101	2100	1	9.90E-08		
DHR10:85056001	10	85056001	7200	1	8.90E-07	Tbx21;Tbkbp1	
DHR10:86369901	10	86369901	3900	1	3.29E-08	Erbb2;U6	Receptor
DHR10:87276601	10	87276601	1000	1	1.75E-07	Krt28	Cytoskeleton
DHR10:88076101	10	88076101	2100	2	3.94E-09	Krt14;Rn50_10_0879.7;LOC108352134;Rn50_10_0879.5	Cytoskeleton
DHR10:96898601	10	96898601	400	1	1.80E-07	Cep112	
DHR10:97953701	10	97953701	7600	1	1.66E-07	Prkar1a;Fam20a	Signaling;Unknown
DHR10:99177701	10	99177701	1400	1	1.99E-07		
DHR10:99255401	10	99255401	1200	1	6.67E-07		
DHR10:99498101	10	99498101	600	1	4.56E-07		
DHR10:100194201	10	100194201	900	1	1.89E-07		
DHR10:103608301	10	103608301	4600	1	3.00E-07	Cd300e	Receptor
DHR10:112214601	10	112214601	1300	1	4.19E-08		
DHR11:713401	11	713401	2000	1	7.57E-08	Epha3	Receptor
DHR11:2657201	11	2657201	300	1	6.93E-08	Pou1f1;Chmp2b	Transcription
DHR11:2904201	11	2904201	1100	1	1.56E-07		
DHR11:4976001	11	4976001	1000	1	9.88E-07		
DHR11:16751201	11	16751201	1500	1	1.36E-08		
DHR11:20322901	11	20322901	700	1	6.58E-07		
DHR11:24413001	11	24413001	2500	1	9.23E-08	App	Signaling
DHR11:32201201	11	32201201	4400	2	2.00E-07	Mrps6	
DHR11:37852201	11	37852201	200	1	8.40E-07	Bace2	Metabolism
DHR11:43430801	11	43430801	100	1	7.90E-07	Olr1545	
DHR11:52032301	11	52032301	1600	1	7.69E-07		
DHR11:53120101	11	53120101	800	1	1.98E-07	Bbx	Transcription
DHR11:55342101	11	55342101	6200	1	6.31E-07		
DHR11:58990701	11	58990701	2100	1	3.92E-07	Lsamp	Extracellular Matrix
DHR11:63672601	11	63672601	100	1	6.03E-07		
DHR11:63709801	11	63709801	2700	1	4.21E-07		
DHR11:65476301	11	65476301	500	1	4.35E-07	AABR07034328.1	
DHR11:75080801	11	75080801	3400	2	4.41E-08	Atp13a5	Transport
DHR11:81865301	11	81865301	500	1	9.81E-09		
DHR12:3207201	12	3207201	100	1	6.21E-08	AABR07035008.3;AABR07035008.1;AABR07035008.2	
DHR12:12345301	12	12345301	1700	1	3.95E-08		
DHR12:15518601	12	15518601	300	1	6.35E-07		
DHR12:18874001	12	18874001	200	1	8.61E-07		

DHR12:21155401	12	21155401	800	1	1.28E-07		
DHR12:30870401	12	30870401	5100	2	6.60E-11		
DHR12:31592301	12	31592301	2700	2	4.80E-09	Rimbp2	Unknown
DHR12:33028301	12	33028301	1100	1	1.93E-07		
DHR12:36208201	12	36208201	100	1	5.47E-07	Tmem132b	Unknown
DHR12:39266201	12	39266201	3200	1	8.90E-07	Camkk2	Signaling
DHR12:41347101	12	41347101	9400	1	2.01E-08	Oas2	Transcription
DHR12:44655701	12	44655701	5100	2	3.43E-08	Ksr2	Signaling
DHR12:49132601	12	49132601	800	1	1.77E-08		
DHR12:49999601	12	49999601	1200	1	6.00E-09		
DHR13:3096801	13	3096801	200	1	4.87E-07	SNORA17	
DHR13:6697101	13	6697101	1300	1	8.03E-07	Cntnap5c	
DHR13:20855101	13	20855101	2400	1	8.01E-07		
DHR13:27932701	13	27932701	1000	1	8.82E-07		
DHR13:31773701	13	31773701	1300	1	5.92E-07		
DHR13:35160901	13	35160901	7100	1	5.50E-08		
DHR13:37498301	13	37498301	1900	1	2.29E-07	Htr5b;AC128353.1	Receptor
DHR13:39227301	13	39227301	2400	1	3.69E-07		
DHR13:48015401	13	48015401	9800	1	7.79E-07	Rassf5;Ikbke	Signaling
DHR13:51577601	13	51577601	9500	1	2.91E-07	Syt2;Ppp1r12b	Transport;Signaling
DHR13:52381501	13	52381501	4100	1	6.89E-07	Nav1	
DHR13:52712701	13	52712701	14800	1	3.61E-07	Pkp1	
DHR13:56924801	13	56924801	4100	1	4.66E-07	RGD1564614	
DHR13:58858601	13	58858601	3100	1	3.26E-07		
DHR13:64326101	13	64326101	2000	1	6.56E-07		
DHR13:66047901	13	66047901	700	1	4.50E-07		
DHR13:71526201	13	71526201	100	1	1.75E-07	SNORA17	
DHR13:83093501	13	83093501	2000	1	1.08E-07	Dpt	Development
DHR13:98634901	13	98634901	5800	1	6.00E-08	Itpkb	Signaling
DHR13:105938801	13	105938801	700	1	1.63E-08		
DHR13:107153901	13	107153901	800	1	6.92E-09	Ush2a	Extracellular Matrix
DHR13:108923601	13	108923601	3900	2	6.90E-08		
DHR13:111361501	13	111361501	1500	2	2.87E-07	Hhat	Metabolism
DHR14:2846901	14	2846901	1400	1	9.58E-07	Fam69a	
DHR14:3107401	14	3107401	2000	1	2.15E-07		
DHR14:3283801	14	3283801	1000	1	2.89E-07	LOC689986;Btbd8	
DHR14:3909701	14	3909701	1600	1	5.05E-07	Hfm1	Epigenetic
DHR14:5182201	14	5182201	2700	1	2.01E-07		
DHR14:12569201	14	12569201	400	1	4.47E-09		
DHR14:13769801	14	13769801	1100	1	7.45E-07		
DHR14:16566901	14	16566901	4000	1	4.89E-07		
DHR14:18632401	14	18632401	400	1	9.01E-07	Epgn;Mthfd2l	Signaling;Metabolism
DHR14:27374201	14	27374201	1300	1	9.37E-08		
DHR14:29488501	14	29488501	1300	1	1.81E-07		
DHR14:29840301	14	29840301	2500	1	3.41E-07		
DHR14:31146501	14	31146501	700	1	1.80E-07		
DHR14:32408701	14	32408701	1800	1	4.44E-07		
DHR14:32647601	14	32647601	3600	1	3.49E-07		
DHR14:43748001	14	43748001	4800	1	5.59E-07		
DHR14:45699901	14	45699901	1900	1	8.42E-10	Tbc1d1	Signaling

DHR14:48414001	14	48414001	4300	1	5.82E-08		
DHR14:49013301	14	49013301	5500	1	7.65E-07		
DHR14:66240201	14	66240201	3000	1	8.46E-08		
DHR14:70086301	14	70086301	3100	1	8.29E-07	Fam184b;Med28	
DHR14:73199901	14	73199901	1800	1	4.98E-08		
DHR14:74461401	14	74461401	2100	1	3.74E-07		
DHR14:74573401	14	74573401	700	1	1.05E-07		
DHR14:80250401	14	80250401	10800	1	1.64E-07	Htra3	
DHR14:83665301	14	83665301	1000	1	3.89E-10	Rnf185	Transcription
DHR14:96904601	14	96904601	500	1	3.60E-07	Rn50_14_0967.1	
DHR14:97134501	14	97134501	100	1	7.98E-07		
DHR14:97583201	14	97583201	100	1	9.11E-07		
DHR14:105636801	14	105636801	100	1	3.10E-07		
DHR14:106429401	14	106429401	200	1	5.14E-07	Wdpcp	
DHR14:108584501	14	108584501	4000	1	2.56E-07		
DHR14:110036601	14	110036601	100	1	6.31E-07		
DHR14:110472901	14	110472901	400	1	9.05E-08		
DHR14:112859001	14	112859001	4600	1	9.97E-07	LOC103690141	
DHR15:7567001	15	7567001	300	1	9.71E-07		
DHR15:10016001	15	10016001	3100	1	6.32E-07		
DHR15:12969401	15	12969401	1600	1	2.95E-07	Ptprg	Signaling
DHR15:16826901	15	16826901	1100	1	2.68E-08		
DHR15:19741701	15	19741701	1700	1	6.28E-07	Gnpnat1	Metabolism
DHR15:22093401	15	22093401	900	1	6.85E-07		
DHR15:23225501	15	23225501	3900	1	4.18E-08		
DHR15:24132601	15	24132601	9000	1	7.50E-07	Lgals3	Cytoskeleton
DHR15:24238701	15	24238701	800	1	8.34E-07		
DHR15:29085801	15	29085801	900	1	6.26E-09	AABR07017599.1	
DHR15:33668101	15	33668101	1300	1	7.16E-07	Ngdn	
DHR15:63647301	15	63647301	1000	1	4.49E-07		
DHR15:64112001	15	64112001	100	1	5.68E-07		
DHR15:66192501	15	66192501	3100	1	7.43E-07		
DHR15:66487401	15	66487401	100	1	1.38E-08	SNORA17	
DHR15:71154301	15	71154301	500	1	8.16E-08		
DHR15:73518001	15	73518001	1800	2	1.26E-07		
DHR15:74103601	15	74103601	900	1	1.16E-08		
DHR15:75627601	15	75627601	1100	1	6.61E-07		
DHR15:79064601	15	79064601	200	2	2.63E-07		
DHR15:81382701	15	81382701	900	1	7.13E-07		
DHR15:89645501	15	89645501	2200	1	3.15E-07	Mycbp2	Metabolism
DHR15:92013601	15	92013601	600	1	4.05E-07	Mycbp2	Metabolism
DHR15:97162101	15	97162101	2000	1	5.00E-08		
DHR15:102625301	15	102625301	700	1	4.43E-07	Gpc6	Extracellular Matrix
DHR15:103712701	15	103712701	1100	1	1.63E-07	Abcc4	Receptor
DHR16:1520801	16	1520801	2000	2	2.94E-09		
DHR16:2434801	16	2434801	200	1	4.16E-08	Dnah12	Cytoskeleton
DHR16:12322101	16	12322101	100	1	6.44E-09		
DHR16:17260901	16	17260901	1200	1	8.06E-07		
DHR16:22674501	16	22674501	3000	1	1.06E-07		
DHR16:22977301	16	22977301	4700	1	7.31E-07	Csgalnact1	Metabolism

DHR16:25368101	16	25368101	5000	1	5.91E-07		
DHR16:29248601	16	29248601	1000	1	4.99E-07		
DHR16:30844501	16	30844501	1300	1	5.82E-07		
DHR16:33227901	16	33227901	1000	1	2.98E-07		
DHR16:54006501	16	54006501	1900	1	1.63E-08	Asah1	
DHR16:55385301	16	55385301	300	1	3.56E-07		
DHR16:58498801	16	58498801	700	1	4.65E-07	AABR07025999.1	
DHR16:59560001	16	59560001	2700	1	2.62E-07	Lonrf1	
DHR16:63152101	16	63152101	600	1	3.27E-07		
DHR16:64190101	16	64190101	4300	1	2.96E-07		
DHR16:68572101	16	68572101	1100	1	5.44E-07		
DHR16:68733201	16	68733201	5900	1	8.91E-08		
DHR16:71258201	16	71258201	3100	1	8.97E-07	Letm2;Fgfr1	Signaling;Receptor
DHR16:77797201	16	77797201	1700	1	6.01E-08		
DHR16:77976301	16	77976301	1100	1	2.71E-07		
DHR16:80098901	16	80098901	10700	1	6.47E-07		
DHR16:80659701	16	80659701	6400	2	1.70E-09	Erich1	Unknown
DHR16:81037201	16	81037201	1100	1	6.28E-08	Tmco3;Dcun1d2;U6	Transport;Proteolysis
DHR16:82605001	16	82605001	3000	1	4.35E-07		
DHR16:82959701	16	82959701	5700	1	5.92E-07		
DHR16:83515001	16	83515001	2300	1	7.73E-07	Col4a1	Cytoskeleton
DHR16:83835001	16	83835001	6600	1	8.65E-07	Irs2	Unknown
DHR16:84591101	16	84591101	2300	1	3.59E-07	Myo16	Cytoskeleton
DHR16:85474401	16	85474401	2300	1	4.77E-07		
DHR16:85488501	16	85488501	1500	1	5.85E-08		
DHR16:85510201	16	85510201	1600	1	5.21E-07		
DHR16:89955101	16	89955101	700	1	7.67E-07		
DHR17:5339801	17	5339801	3000	1	9.99E-07	Spata31d1b;Spata31d3	
DHR17:9042601	17	9042601	5800	1	2.74E-07		
DHR17:9672301	17	9672301	3900	1	3.92E-07	Pdlim7;Dbn1	Receptor;Development
DHR17:10907201	17	10907201	2800	1	8.61E-07		
DHR17:12824201	17	12824201	1000	1	3.78E-07		
DHR17:18368201	17	18368201	1000	1	7.68E-07	Nup153	Transcription
DHR17:21678701	17	21678701	2100	1	2.16E-07	Gcnt2	Golgi
DHR17:24287101	17	24287101	6100	1	2.42E-07	Rnf182	
DHR17:24965901	17	24965901	1900	1	5.45E-08		
DHR17:30107501	17	30107501	1000	1	9.41E-08		
DHR17:32154201	17	32154201	100	1	1.01E-07	Nqo2	Metabolism
DHR17:33226501	17	33226501	3200	1	1.10E-07		
DHR17:33394001	17	33394001	5100	1	7.10E-07	Gmds	Metabolism
DHR17:38293801	17	38293801	4000	1	1.92E-07		
DHR17:51697301	17	51697301	2500	1	2.12E-07		
DHR17:55170901	17	55170901	500	1	4.30E-07		
DHR17:60772001	17	60772001	900	1	2.24E-07		
DHR17:61918901	17	61918901	1200	1	2.56E-08		
DHR17:68746201	17	68746201	2900	1	7.70E-07		
DHR17:69916801	17	69916801	2400	2	3.85E-07		
DHR17:71555301	17	71555301	4400	1	7.61E-07		
DHR17:75764701	17	75764701	6100	1	2.49E-07	Usp6nl	Signaling

DHR17:76340101	17	76340101	5900	1	3.79E-07	Dhtkd1	Metabolism
DHR17:80652401	17	80652401	1600	1	1.50E-07	Cubn	Receptor
DHR17:84289101	17	84289101	600	1	2.87E-07	Nebl	Cytoskeleton
DHR17:86450001	17	86450001	100	1	4.25E-07		
DHR17:88432001	17	88432001	900	1	1.79E-08	Gpr158	Receptor
DHR17:89071401	17	89071401	1900	1	6.13E-07	Myo3a	Cytoskeleton
DHR18:6224301	18	6224301	500	1	4.62E-07	Taf4b	Transcription
DHR18:9201001	18	9201001	1200	1	5.27E-07		
DHR18:9444701	18	9444701	1200	1	7.52E-07		
DHR18:17426201	18	17426201	2100	1	5.32E-07	RGD1562608	EST
DHR18:17480301	18	17480301	3300	1	7.48E-07	RGD1562608	EST
DHR18:20041001	18	20041001	700	1	1.90E-07		
DHR18:25095501	18	25095501	1700	1	7.26E-08		
DHR18:27195301	18	27195301	3300	1	4.47E-07	Pkd2l2;AABR07031691.1	Development
DHR18:30744401	18	30744401	1300	1	4.84E-07		
DHR18:37923701	18	37923701	500	1	1.40E-07	Jakmip2	
DHR18:44107301	18	44107301	1500	1	8.99E-09	LOC103694210	
DHR18:46655901	18	46655901	1500	1	2.55E-09		
DHR18:46824301	18	46824301	100	1	3.79E-07		
DHR18:47777701	18	47777701	1700	1	9.71E-08	Sncaip	
DHR18:50384301	18	50384301	2100	1	5.98E-07		
DHR18:51866301	18	51866301	3000	1	7.46E-07	3-Mar	Metabolism
DHR18:53401601	18	53401601	400	1	1.49E-07		
DHR18:53971501	18	53971501	600	1	3.44E-07	Adamts19	Proteolysis
DHR18:54281801	18	54281801	1200	1	3.45E-07	Chsy3	Metabolism
DHR18:54612001	18	54612001	2200	1	7.52E-07		
DHR18:65216501	18	65216501	600	1	9.56E-07	Tcf4	Transcription
DHR18:68183101	18	68183101	1600	1	5.69E-07		
DHR18:71839701	18	71839701	7900	1	7.06E-07	Ctif	
DHR18:71982301	18	71982301	300	1	1.99E-07	AABR07032563.1;AABR07032563.2	
DHR18:74346001	18	74346001	2000	1	5.99E-07	Epg5	
DHR18:75317701	18	75317701	400	2	3.17E-07		
DHR18:84314301	18	84314301	400	1	6.42E-07		
DHR19:692501	19	692501	3200	1	8.23E-07	Terb1;U6	
DHR19:3174201	19	3174201	2800	1	5.36E-08		
DHR19:9255301	19	9255301	600	1	6.58E-07		
DHR19:10323101	19	10323101	4900	1	1.30E-07	Kifc3	Cytoskeleton
DHR19:10543001	19	10543001	6000	1	6.64E-07	Ccdc102a	
DHR19:18134201	19	18134201	2000	1	3.99E-07	Tox3	Transcription
DHR19:30266101	19	30266101	100	1	1.81E-07		
DHR19:34950301	19	34950301	3300	1	7.85E-07		
DHR19:44754801	19	44754801	3700	1	1.16E-07		
DHR19:52418801	19	52418801	1900	1	7.40E-08	Tldc1	Unknown
DHR19:52474301	19	52474301	3300	1	1.44E-07	Cotl1	Cytoskeleton
DHR19:52551601	19	52551601	3300	1	6.30E-08		
DHR19:52900001	19	52900001	4700	1	3.26E-07		
DHR19:57858501	19	57858501	9000	1	7.61E-07	Disc1	
DHR19:59517501	19	59517501	1400	1	9.90E-07		

DHR19:60051001	19	60051001	700	1	9.12E-08	Pard3	Cell Junction
DHR19:60151901	19	60151901	2200	1	4.43E-07	Pard3	Cell Junction
DHR20:34001	20	34001	1400	1	1.38E-07	LOC100910263	Receptor
DHR20:11560301	20	11560301	4500	1	7.96E-07	RGD1561557;LOC100365646	EST
DHR20:13453401	20	13453401	1000	1	8.89E-08	Slc5a4	Transport
DHR20:19232901	20	19232901	1000	2	8.05E-07		
DHR20:23379901	20	23379901	2300	1	4.46E-07		
DHR20:36429901	20	36429901	1500	1	3.07E-07		
DHR20:39524701	20	39524701	3700	1	2.90E-07		
DHR20:45575901	20	45575901	500	1	9.12E-08	Cdk19	Signaling
DHR20:46296001	20	46296001	6300	1	9.39E-07	Ccdc162	
DHR20:48972401	20	48972401	2200	1	2.62E-08	Aim1;AABR07045487.1	Development
DHR20:49220801	20	49220801	700	2	2.57E-07		
DHR20:49649801	20	49649801	600	1	6.05E-07		
DHR20:52944301	20	52944301	300	1	8.08E-07		
DHRX:12002401	X	12002401	400	1	1.81E-07		
DHRX:32687201	X	32687201	800	1	3.76E-07		
DHRX:48904801	X	48904801	200	1	2.70E-07		
DHRX:50851601	X	50851601	100	1	4.07E-07		
DHRX:81088701	X	81088701	2600	1	5.21E-07		
DHRX:90990401	X	90990401	200	1	4.87E-07		
DHRX:114074201	X	114074201	1100	1	6.92E-07	Tmem164	Unknown
DHRX:119277501	X	119277501	100	1	7.81E-07		
DHRX:133210701	X	133210701	200	1	8.29E-07		
DHRX:149618101	X	149618101	300	1	8.80E-07		
