# Supplementary material for: Alterations in sperm DNA methylation, non-coding RNA and histone retention associate with DDT-induced epigenetic transgenerational inheritance of disease
Source: Epigenetics Chromatin. 2018 Feb 27;11:8. doi: 10.1186/s13072-018-0178-0 (PMC5827984; doi:10.1186/s13072-018-0178-0)
Supplement: Supplementary file 10 — Additional file 10: Table S8. F3 H3K27me3 DHR p < 1e−06. [file 13072_2018_178_MOESM10_ESM.pdf]

Supplemental Table S8
F3 H3K27me3 DHR p<1e-06

DHR Name	Chr	Start	Length	num Sig Win	minP	Annotation	Category
DHR1:11906001	1	11906001	5800	1	2.81E-05	5_8S_rRNA;pRNA	
DHR1:23249201	1	23249201	200	1	9.06E-05		
DHR1:24921801	1	24921801	1200	1	2.26E-05	AABR07000740.1	
DHR1:26375101	1	26375101	200	1	7.77E-05		
DHR1:37555101	1	37555101	300	1	6.06E-05	Adcy2	Metabolism
DHR1:38367401	1	38367401	400	1	9.06E-05		
DHR1:54297701	1	54297701	700	1	1.48E-05	AABR07001610.1;LOC102551080	
DHR1:55218101	1	55218101	4200	2	1.33E-06	RGD1561667	Signaling
DHR1:55702201	1	55702201	16300	1	9.24E-05	AABR07001734.1	
DHR1:55730701	1	55730701	10300	1	1.43E-06	AABR07001734.1	
DHR1:56023501	1	56023501	2500	1	5.68E-06		
DHR1:99049101	1	99049101	9000	1	6.67E-05	Vom2r38	Receptor
DHR1:115337701	1	115337701	1100	1	4.31E-05	SNORA17	
DHR1:130465501	1	130465501	1700	1	9.89E-05		
DHR1:152591201	1	152591201	400	1	3.40E-05		
DHR1:157854601	1	157854601	200	1	5.23E-05		
DHR1:159963601	1	159963601	200	1	6.60E-05		
DHR1:169133301	1	169133301	300	1	9.78E-05	Olr136	
DHR1:180785901	1	180785901	7800	1	7.38E-05		
DHR1:181071501	1	181071501	8400	1	5.46E-05		
DHR1:182789701	1	182789701	200	1	6.66E-05		
DHR1:186562001	1	186562001	300	1	1.49E-05		
DHR1:194547001	1	194547001	100	1	9.00E-06		
DHR1:194915601	1	194915601	2800	1	2.21E-05	SNORD116	
DHR1:213109601	1	213109601	200	1	1.98E-06		
DHR1:215516201	1	215516201	100	1	4.21E-05	Ifitm10	
DHR1:221469501	1	221469501	100	1	2.56E-05	LOC100910252;Naaladl1	Protease
DHR1:238842501	1	238842501	100	1	1.61E-05	AABR07006536.1;Zfand5	Transcription
DHR1:252382401	1	252382401	100	1	2.54E-05	Lipn	
DHR1:257008901	1	257008901	200	1	7.05E-05	Lgi1	Receptor
DHR2:1382201	2	1382201	3400	3	9.94E-06		
DHR2:6073501	2	6073501	600	1	7.34E-07		
DHR2:6099401	2	6099401	900	1	2.09E-05		
DHR2:78306201	2	78306201	300	1	7.77E-05		
DHR2:88686701	2	88686701	800	1	9.06E-05		
DHR2:91251101	2	91251101	100	1	2.50E-05		
DHR2:112394501	2	112394501	200	1	5.54E-05	Spata16	
DHR2:128810401	2	128810401	200	1	5.93E-05	5_8S_rRNA	
DHR2:137307501	2	137307501	700	1	7.77E-05	LOC365791	Signaling
DHR2:138134401	2	138134401	1000	1	2.45E-05		
DHR2:150007801	2	150007801	100	1	7.05E-05	AABR07010747.1	
DHR2:156780501	2	156780501	600	1	3.74E-05		
DHR2:158851001	2	158851001	2600	1	8.08E-06		
DHR2:159283601	2	159283601	100	1	2.54E-05		

DHR2:160446401	2	160446401	1000	1	2.99E-05		
DHR2:160685501	2	160685501	1800	1	1.38E-05		
DHR2:160689901	2	160689901	5700	2	4.36E-06		
DHR2:160873201	2	160873201	1500	1	3.74E-05		
DHR2:161558501	2	161558501	500	1	3.38E-06	5_8S_rRNA	
DHR2:161638501	2	161638501	300	1	6.08E-05		
DHR2:171923101	2	171923101	800	1	2.59E-05		
DHR2:183589001	2	183589001	300	1	3.40E-05	Arfip1;Tigd4	Signaling;Transcription
DHR2:197151601	2	197151601	100	1	8.78E-05		
DHR2:198589801	2	198589801	600	2	2.79E-05	AABR07012587.1;U1	
DHR2:215191701	2	215191701	1000	1	7.35E-05	SNORA17	
DHR2:240590901	2	240590901	700	1	2.77E-07	SNORA26;Cisd2	Development
DHR2:244687701	2	244687701	1000	1	1.63E-05	Stpg2	Development
DHR2:253901801	2	253901801	200	1	2.50E-05		
DHR3:18084101	3	18084101	200	1	5.50E-05		
DHR3:63982801	3	63982801	100	1	4.06E-05	Ccdc141	Cytoskeleton
DHR3:70711401	3	70711401	3200	1	1.70E-05	U6	
DHR3:76437101	3	76437101	100	1	7.27E-06		
DHR3:91660301	3	91660301	600	2	2.09E-06	Ldlrad3	Receptor
DHR3:92883401	3	92883401	200	1	1.50E-05		
DHR3:133561201	3	133561201	7500	6	3.17E-05		
DHR3:147438401	3	147438401	100	1	6.90E-05	Angpt4	Signaling
DHR3:167095101	3	167095101	200	1	7.70E-05		
DHR4:12978501	4	12978501	400	1	3.40E-05		
DHR4:63282501	4	63282501	200	1	1.93E-05		
DHR4:63894401	4	63894401	200	1	4.20E-05		
DHR4:70397001	4	70397001	1500	1	3.55E-05		
DHR4:74464701	4	74464701	200	1	6.90E-05		
DHR4:77451901	4	77451901	1500	1	3.32E-05	AABR07060519.1;Y_RNA	
DHR4:100450101	4	100450101	200	1	9.06E-05	Elmod3	Signaling
DHR4:116854901	4	116854901	900	1	1.76E-06	AABR07061333.2	
DHR4:146541501	4	146541501	7300	1	7.91E-06		
DHR4:162266001	4	162266001	2000	1	4.29E-05		
DHR4:174986601	4	174986601	1000	1	3.22E-05		
DHR5:244101	5	244101	500	1	4.69E-05		
DHR5:6372701	5	6372701	2000	20	7.98E-33	AABR07046778.1	
DHR5:7015601	5	7015601	1900	1	4.26E-05	RGD1564053	Unknown
DHR5:28614001	5	28614001	700	1	8.88E-05	Necab1	
DHR5:86596601	5	86596601	1600	1	5.23E-05	Megf9	Extracellular Matrix
DHR5:89412801	5	89412801	300	1	2.33E-05		
DHR5:90524301	5	90524301	300	1	5.73E-05	SNORA17	
DHR5:139783701	5	139783701	200	1	9.06E-05	Rims3	Signaling
DHR5:153641501	5	153641501	100	1	4.76E-05		
DHR5:167331301	5	167331301	100	1	7.88E-07	Rere	Unknown
DHR5:167783001	5	167783001	100	1	6.49E-06		
DHR6:34443601	6	34443601	900	1	2.37E-05		
DHR6:46548301	6	46548301	200	1	6.19E-05		
DHR6:69654801	6	69654801	200	1	6.54E-05		
DHR6:106891501	6	106891501	100	1	8.95E-05	Dpf3	Transcription

DHR6:120698001	6	120698001	300	1	5.81E-05		
DHR6:138245301	6	138245301	100	1	8.25E-05	Ighm;AABR07065631.2;AABR07065631.1;AABR07065631.3	
DHR6:138620901	6	138620901	3500	1	3.26E-05	Ighm;AABR07065651.3	
DHR6:139259901	6	139259901	5200	1	3.39E-05		
DHR6:144073301	6	144073301	100	1	2.54E-05	Wdr60;LOC102547216	Unknown
DHR7:454001	7	454001	3400	1	1.24E-05	AABR07055191.1	
DHR7:749001	7	749001	2900	1	1.84E-05		
DHR7:17343101	7	17343101	2000	1	5.11E-05		
DHR7:19677601	7	19677601	100	1	9.06E-05		
DHR7:19682801	7	19682801	1900	1	9.79E-05		
DHR7:31314401	7	31314401	200	1	3.61E-05	Anks1b	Receptor
DHR7:42604201	7	42604201	900	1	1.93E-05		
DHR7:89347401	7	89347401	300	1	5.65E-05		
DHR7:100303301	7	100303301	2100	1	1.61E-05		
DHR7:124908501	7	124908501	1100	1	1.65E-05	Efcab6	Signaling
DHR8:181201	8	181201	1100	11	1.21E-15		
DHR8:2588101	8	2588101	100	1	6.19E-05		
DHR8:15595001	8	15595001	2900	1	9.39E-05		
DHR8:15925401	8	15925401	100	1	6.19E-05		
DHR8:34057701	8	34057701	100	1	9.61E-05		
DHR8:40207101	8	40207101	200	1	8.21E-05	Rn50_8_0402.2	
DHR8:44677801	8	44677801	300	1	5.64E-06	Vom2r3	Receptor
DHR8:88227001	8	88227001	400	1	3.25E-05		
DHR9:974701	9	974701	100	1	2.08E-05		
DHR9:9116901	9	9116901	2700	1	5.68E-05	MGC116197	Unknown
DHR9:12297001	9	12297001	2000	1	4.56E-05		
DHR9:29186001	9	29186001	200	1	3.40E-05		
DHR9:48489001	9	48489001	800	1	3.31E-06		
DHR9:52918001	9	52918001	200	1	4.24E-05	Slc40a1	Metabolism
DHR9:68375801	9	68375801	1500	1	6.26E-06		
DHR9:83391301	9	83391301	2400	1	5.73E-05	AABR07068094.1	
DHR9:96914901	9	96914901	100	1	3.03E-05	Agap1	Signaling
DHR9:106280401	9	106280401	600	3	1.83E-05		
DHR9:113331601	9	113331601	200	1	3.27E-05	Vapa	Transport
DHR10:3397001	10	3397001	100	1	4.24E-05	5S_rRNA	
DHR10:34546401	10	34546401	200	1	6.60E-05	Mgat1;Olr1387	Metabolism;Receptor
DHR10:41694501	10	41694501	2100	1	2.26E-05		
DHR10:49775701	10	49775701	300	1	3.17E-05		
DHR10:53101701	10	53101701	300	1	5.23E-05		
DHR10:75365201	10	75365201	1200	3	1.85E-08	Vezf1	Transcription
DHR10:90550201	10	90550201	600	1	5.51E-05	Fzd2	Receptor
DHR10:105552801	10	105552801	100	1	2.54E-05	Ube2o;U6	Proteolysis
DHR10:108146901	10	108146901	200	1	6.90E-05	Cbx2;Cbx8	Transcription
DHR11:58209201	11	58209201	700	1	7.37E-05		
DHR11:75552301	11	75552301	200	1	3.54E-05		
DHR11:88400101	11	88400101	300	1	6.07E-05	Igll1;SNORA17	Immune
DHR12:1508501	12	1508501	4700	2	2.99E-06	5S_rRNA;AABR07034940.2;Rn5s;AABR07034940.1	

DHR12:3372701	12	3372701	900	1	1.54E-05		
DHR12:12374601	12	12374601	100	1	6.39E-05	Tecpr1	Development
DHR12:21180601	12	21180601	300	1	8.88E-05		
DHR12:43707201	12	43707201	200	1	5.11E-06		
DHR13:32778901	13	32778901	400	1	2.50E-05		
DHR13:33487601	13	33487601	2400	1	5.29E-05		
DHR13:33527901	13	33527901	600	1	9.40E-05		
DHR13:33534601	13	33534601	400	1	4.92E-05		
DHR13:33579401	13	33579401	500	1	7.44E-05		
DHR13:33584101	13	33584101	11400	1	7.88E-05		
DHR13:33756701	13	33756701	7000	2	3.00E-05		
DHR13:48021801	13	48021801	200	1	2.80E-05	Rassf5;Ikbke	Signaling
DHR13:49969901	13	49969901	200	1	5.57E-06	AABR07020979.1	
DHR14:47953701	14	47953701	1700	1	2.21E-05		
DHR14:83656001	14	83656001	100	1	2.54E-05	Rnf185	Transcription
DHR15:29357901	15	29357901	1800	1	3.86E-05	RGD1563780;LOC688340;AABR07017624.1	
DHR15:29664201	15	29664201	600	1	6.60E-05	AABR07017649.1;LOC100911282	
DHR15:56665201	15	56665201	200	1	1.03E-07	Htr2a	Receptor
DHR15:65020301	15	65020301	900	1	1.39E-05		
DHR15:65172401	15	65172401	200	1	2.74E-05		
DHR15:67662301	15	67662301	100	1	4.24E-05		
DHR15:99767601	15	99767601	200	1	4.24E-05		
DHR15:107466901	15	107466901	900	1	5.81E-05		
DHR16:22043401	16	22043401	1400	1	9.94E-05	RGD1563748	
DHR16:34814601	16	34814601	200	1	6.53E-05		
DHR16:58121701	16	58121701	200	1	4.24E-05		
DHR16:66363601	16	66363601	600	1	6.38E-05	LOC689479	Unknown
DHR16:67363801	16	67363801	700	1	8.82E-05	Unc5d	Receptor
DHR17:54463901	17	54463901	100	1	4.08E-05		
DHR17:89387501	17	89387501	9400	4	4.87E-05		
DHR17:89407901	17	89407901	3700	3	1.56E-05		
DHR17:89415501	17	89415501	2900	3	3.53E-05		
DHR17:89434001	17	89434001	8400	2	1.78E-05		
DHR17:89443501	17	89443501	3100	1	4.21E-05	RGD1561231	
DHR17:89450401	17	89450401	13700	1	3.30E-05	RGD1561231	
DHR18:3317101	18	3317101	800	1	6.19E-05	Cables1;AABR07031182.1	
DHR18:25222001	18	25222001	100	1	9.74E-05	Bin1	Transcription
DHR18:39851701	18	39851701	100	1	9.89E-05		
DHR18:73153501	18	73153501	200	2	1.52E-05	Skor2	
DHR18:86090801	18	86090801	300	1	1.28E-05	Rttn	Development
DHR19:8431801	19	8431801	300	1	6.60E-07		
DHR19:15097601	19	15097601	100	1	5.96E-06	Ces1d;Ces1f	Metabolism
DHR19:29326801	19	29326801	2200	1	9.75E-05	AABR07043564.1;Polr2m	
DHR19:29919501	19	29919501	100	1	2.50E-05	Inpp4b	Signaling
DHR19:54552601	19	54552601	100	1	3.93E-06	Jph3	Cell Junction
DHR20:1729401	20	1729401	500	1	4.51E-07	Olr1734	Receptor

DHR20:4920001	20	4920001	2700	1	3.91E-05	RT1-CE1;RT1-CE4;AABR07044408.1;Rn60_20_0050.1;RT1-CE3	Immune
DHR20:5351801	20	5351801	1900	1	1.50E-05	Rn60_20_0054.5;Rn60_20_0054.3;RT1-A1;RT1-CE9-ps1	Immune
DHR20:15417601	20	15417601	500	1	6.78E-05		
DHR20:19484001	20	19484001	1800	13	4.56E-19	Fam13c	
DHR20:23536201	20	23536201	800	1	2.55E-05	SNORA17	
DHR20:34683301	20	34683301	100	1	4.24E-05	Cep85l	Epigenetic
DHR20:40877601	20	40877601	1200	1	4.06E-05		
DHRMT:1	MT	1	16300	160	1.43E-26	AY172581.13;AY172581.9;AY172581.3;AY172581.24;AY172581.14;Mt-nd1;AY172581.4;AY172581.21;AY172581.15;Mt-nd2;AY172581.6;AY172581.22;AY172581.18;AY172581.10;AY172581.7;Mt-co1;AY172581.19;AY172581.12;Mt-co2;AY172581.1;Mt-atp8;Mt-atp6;Mt-cox3;AY172581.5;Mt-nd3;AY172581.16;Mt-nd4l;Mt-nd4;AY172581.23;AY172581.17;AY172581.11;Mt-nd5;Mt-nd6;AY172581.20;Mt-cyb;AY172581.8;AY172581.2	
DHRX:14951701	X	14951701	3800	1	1.98E-05	SNORA26;Slc38a5	Unknown
DHRX:63744201	X	63744201	1300	1	6.56E-05		
DHRX:64295501	X	64295501	800	1	6.54E-05	Arhgef9	Signaling
DHRX:78632601	X	78632601	2800	1	7.45E-05		
DHRX:84348901	X	84348901	1900	1	3.91E-05		
DHRX:113409801	X	113409801	1600	1	8.56E-05	Gucy2f	Translation
DHRX:137061001	X	137061001	2000	1	3.84E-05		
DHRY:2080301	Y	2080301	300	1	6.39E-05		
