# Supplementary figures and images for: Alterations in sperm DNA methylation, non-coding RNA and histone retention associate with DDT-induced epigenetic transgenerational inheritance of disease
Source: Epigenetics Chromatin. 2018 Feb 27;11:8. doi: 10.1186/s13072-018-0178-0 (PMC5827984; doi:10.1186/s13072-018-0178-0)

Genome browser map of associated DMRs, ncRNA and DHR (Chromosome 22q)

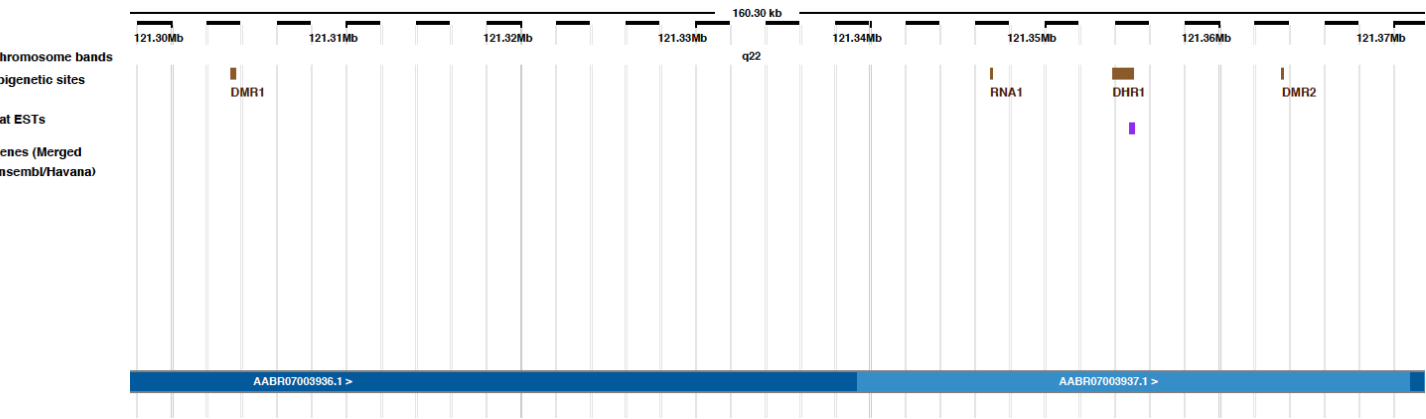

Supplement: Supplementary file 11 — Additional file 11: Fig. S3. Genome Browser representative map of site with two DMRs, one ncRNA and one histone retention site on chromosome 22q. One exposed sequence tag (EST) is present. [file 13072_2018_178_MOESM11_ESM.pdf]

Supplemental Figure S4

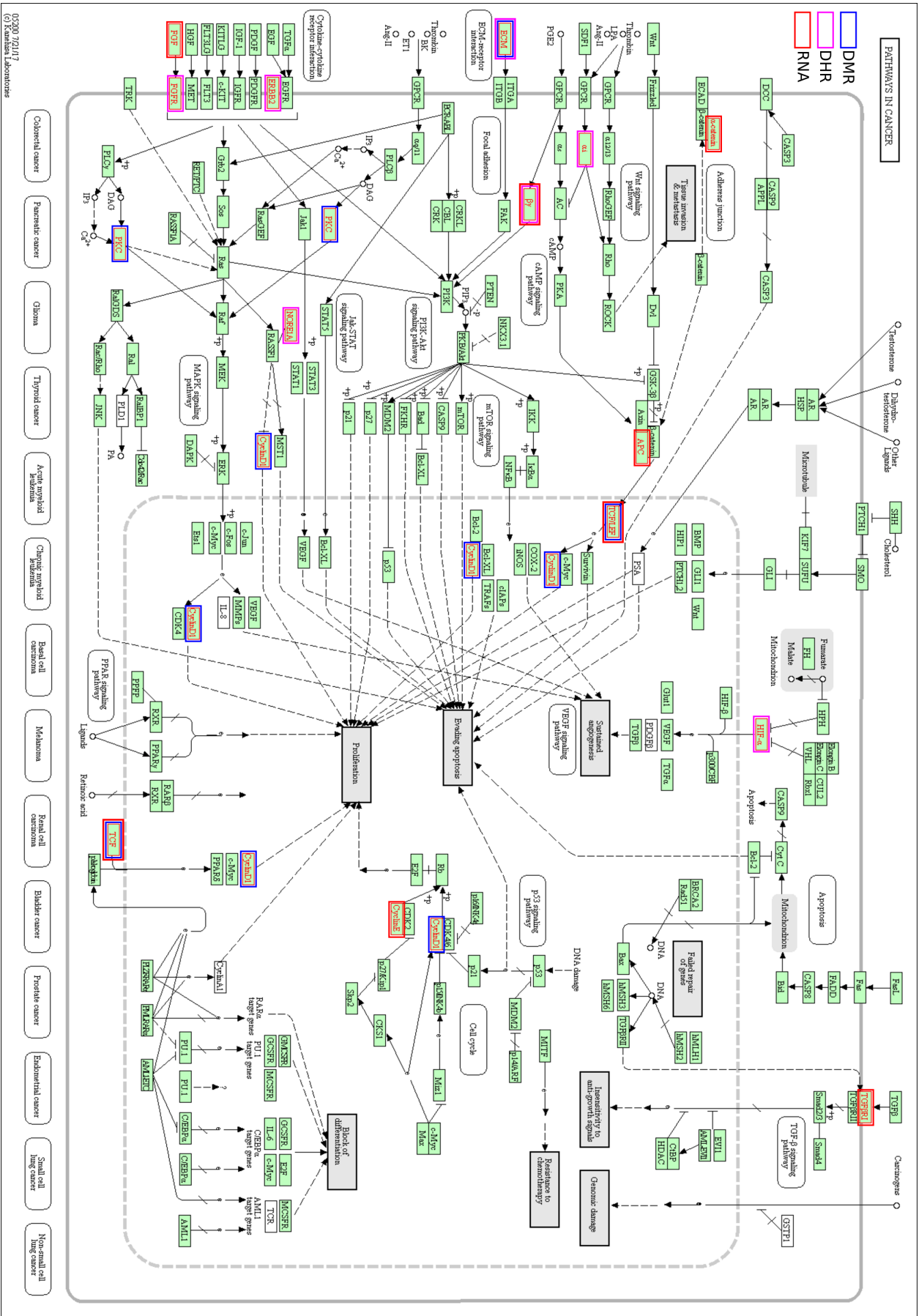

Supplement: Supplementary file 12 — Additional file 12: Fig. S4. Epimutation-associated gene pathway for the pathways in cancer containing 5 DMRs (blue box), 6 ncRNA (red box) and 7 DHRs (pink box). [file 13072_2018_178_MOESM12_ESM.pdf]

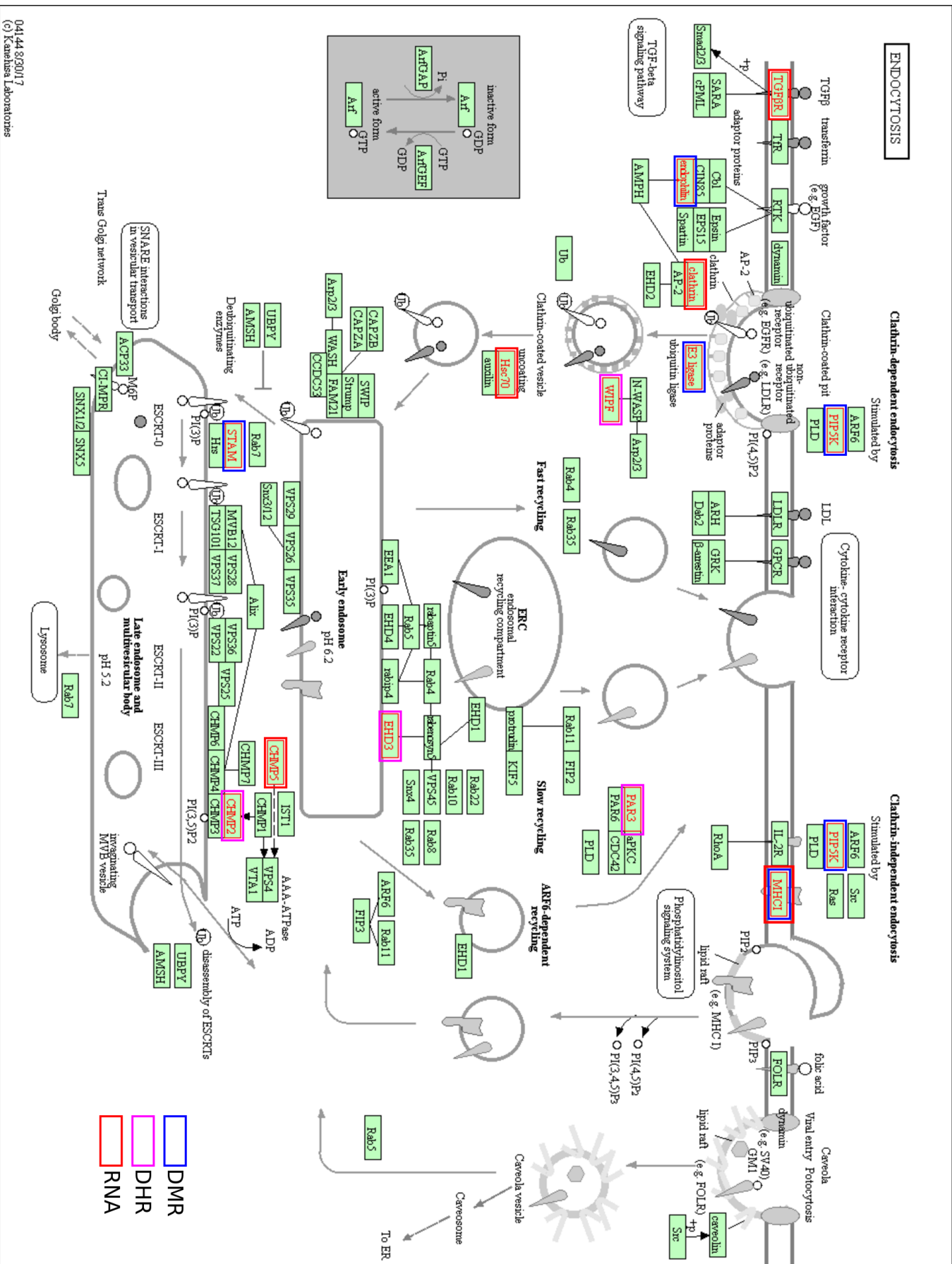

Supplement: Supplementary file 13 — Additional file 13: Fig. S5. Epimutation-associated gene pathways for endocytosis containing 5 DMRs (blue box), 5 ncRNA (red box) and 6 DHRs (pink box). [file 13072_2018_178_MOESM13_ESM.pdf]

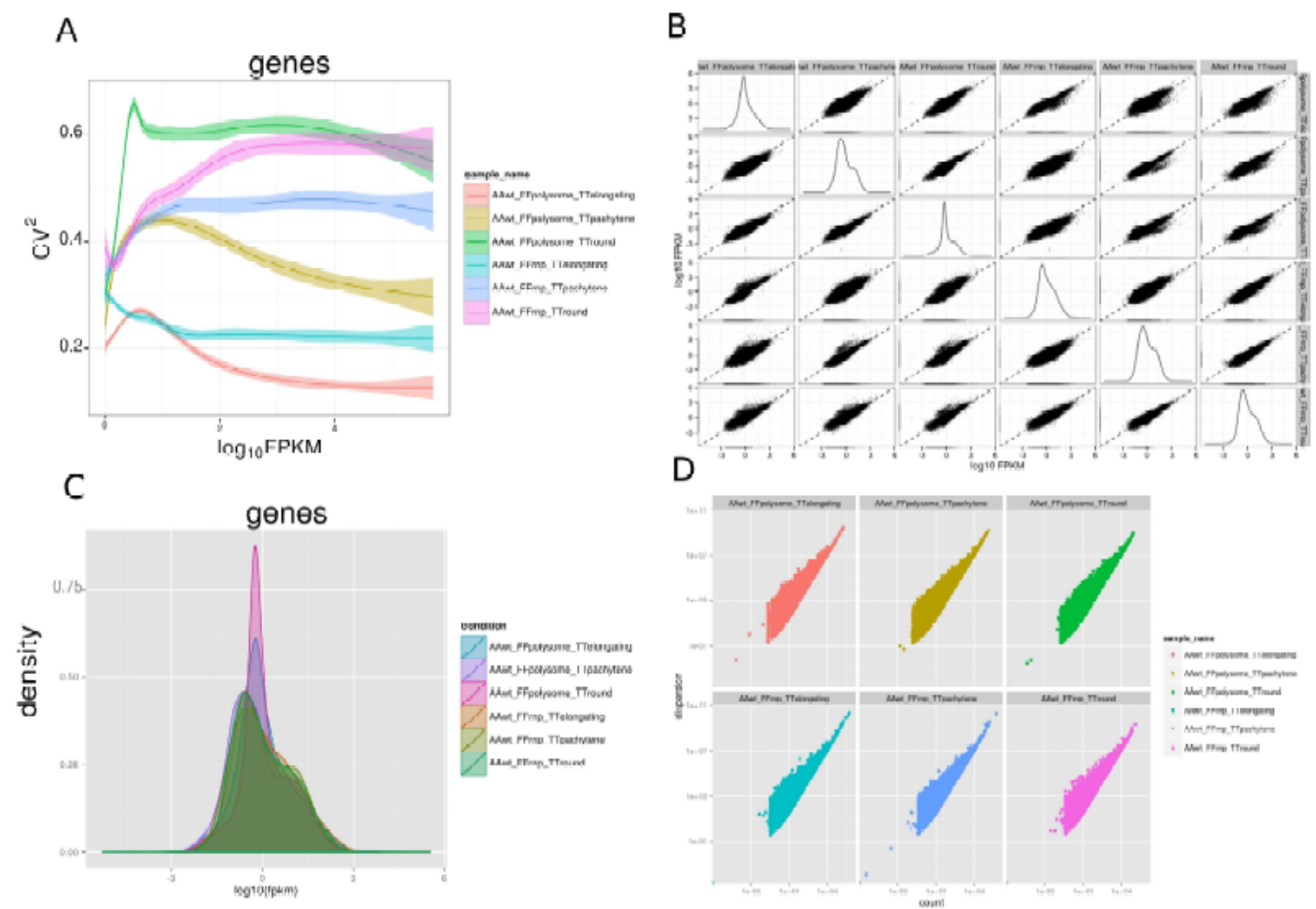

Supplement: Supplementary file 14 — Additional file 14: Fig. S6. Evaluation of the quality of the RNA-Seq data generated in this study. (A) Variations among biological replicates of the six RNA samples (pachytene RNP, pachytene polysome, round spermatid RNP, round spermatid polysome, elongating RNP and elongating polysome). The biological variation is reflected by the coefficient of variation to the power of two (CV2) of FPKM values for each gene. The CV2 represents a normalized measure of cross-replicate variability, which has been widely used for evaluating quality of RNA-Seq data. The data presented here show that the abundance of the genes varied between replicate RNA samples, especially for the ones with lower FPKM values, which is expected. (B) Scatterplot matrix showing the pairwise scatterplots of the log10-normalized FPKM scores across biological replicates of all six RNA samples. (C) Density plots showing the distribution of log10-normalized FPKM scores across biological replicates of all six RNA samples. (D) Overdispersion plots demonstrating the estimated overdispersion for each sample as a quality control measure. [file 13072_2018_178_MOESM14_ESM.pdf]
